# Canadian Association of Gastroenterology Clinical Practice Guideline for Immunizations in Patients With Inflammatory Bowel Disease (IBD)—Part 2: Inactivated Vaccines

**DOI:** 10.1093/jcag/gwab016

**Published:** 2021-07-29

**Authors:** Jennifer L Jones, Frances Tse, Matthew W Carroll, Jennifer C deBruyn, Shelly A McNeil, Anne Pham-Huy, Cynthia H Seow, Lisa L Barrett, Talat Bessissow, Nicholas Carman, Gil Y Melmed, Otto G Vanderkooi, John K Marshall, Eric I Benchimol

**Affiliations:** 1 Department of Medicine and Community Health and Epidemiology, Dalhousie University, Queen Elizabeth II Health Sciences Center, Halifax, Nova Scotia, Canada; 2 Division of Gastroenterology and Farncombe Family Digestive Health Research Institute, McMaster University, Hamilton, Ontario, Canada; 3 Division of Pediatric Gastroenterology, Hepatology and Nutrition, Department of Pediatrics, University of Alberta, Edmonton, Alberta, Canada; 4 Section of Pediatric Gastroenterology, Departments of Pediatrics and Community Health Sciences, University of Calgary, Calgary, Alberta, Canada; 5 Division of Infectious Diseases, Department of Medicine, Dalhousie University, Halifax, Nova Scotia, Canada; 6 Division of Infectious Diseases, Immunology and Allergy, Department of Pediatrics, Children’s Hospital of Eastern Ontario, University of Ottawa, Ottawa, Ontario, Canada; 7 Division of Gastroenterology, Departments of Medicine and Community Health Sciences, University of Calgary, Calgary, Alberta, Canada; 8 Division of Gastroenterology, McGill University Health Centre, Montreal, Quebec, Canada; 9 Department of Pediatrics, University of Ottawa, Ottawa, Ontario, Canada; 14 CHEO Inflammatory Bowel Disease Centre, Division of Gastroenterology, Hepatology and Nutrition, Children’s Hospital of Eastern Ontario, Ottawa, Ontario, Canada; 10 Inflammatory Bowel Disease Center, Cedars-Sinai Medical Center, Los Angeles, California, United States; 11 Section of Infectious Diseases, Departments of Pediatrics, Microbiology, Immunology and Infectious Diseases, Pathology and Laboratory Medicine and Community Health Sciences, University of Calgary, Alberta Children’s Hospital Research Institute, Calgary, Alberta, Canada; 12 Department of Pediatrics and School of Epidemiology and Public Health, University of Ottawa, Ottawa, Ontario, Canada; 15 CHEO Inflammatory Bowel Disease Centre, Division of Gastroenterology, Hepatology and Nutrition, Children’s Hospital of Eastern Ontario and CHEO Research Institute, Ottawa, Ontario, Canada; 16 ICES Ottawa, Ottawa, Ontario, Canada; 13 Department of Paediatrics, University of Toronto, Toronto, Ontario, Canada, SickKids Inflammatory Bowel Disease Centre, Division of Gastroenterology Hepatology and Nutrition, The Hospital for Sick Children, Child Health Evaluative Sciences, SickKids Research Institute, ICES, Toronto, Ontario, Canada

## Abstract

**Background and Aims:**

The effectiveness and safety of vaccinations can be altered by immunosuppressive therapies, and perhaps by inflammatory bowel disease (IBD) itself. These recommendations developed by the Canadian Association of Gastroenterology and endorsed by the American Gastroenterological Association, aim to provide guidance on immunizations in adult and pediatric patients with IBD. This publication focused on inactivated vaccines.

**Methods:**

Systematic reviews evaluating the efficacy, effectiveness, and safety of vaccines in patients with IBD, other immune-mediated inflammatory diseases, and the general population were performed. Critical outcomes included mortality, vaccine-preventable diseases, and serious adverse events. Immunogenicity was considered a surrogate outcome for vaccine efficacy. Certainty of evidence and strength of recommendations were rated according to the GRADE (Grading of Recommendation Assessment, Development, and Evaluation) approach. Key questions were developed through an iterative online platform, and voted on by a multidisciplinary group. Recommendations were formulated using the Evidence-to-Decision framework. Strong recommendation means that most patients should receive the recommended course of action, whereas a conditional recommendation means that different choices will be appropriate for different patients.

**Results:**

Consensus was reached on 15 of 20 questions. Recommendations address the following vaccines: *Haemophilus influenzae* type b, recombinant zoster, hepatitis B, influenza, pneumococcus, meningococcus, tetanus-diphtheria-pertussis, and human papillomavirus. Most of the recommendations for patients with IBD are congruent with the current Centers for Disease Control and Prevention and Canada’s National Advisory Committee on Immunization recommendations for the general population, with the following exceptions. In patients with IBD, the panel suggested *Haemophilus influenzae* type b vaccine for patients older than 5 years of age, recombinant zoster vaccine for adults younger than 50 year of age, and hepatitis B vaccine for adults without a risk factor. Consensus was not reached, and recommendations were not made for 5 statements, due largely to lack of evidence, including double-dose hepatitis B vaccine, timing of influenza immunization in patients on biologics, pneumococcal and meningococcal vaccines in adult patients without risk factors, and human papillomavirus vaccine in patients aged 27–45 years.

**Conclusions:**

Patients with IBD may be at increased risk of some vaccine-preventable diseases. Therefore, maintaining appropriate vaccination status in these patients is critical to optimize patient outcomes. In general, IBD is not a contraindication to the use of inactivated vaccines, but immunosuppressive therapy may reduce vaccine responses.

Patients with inflammatory bowel disease (IBD) may be at increased risk of some vaccine-preventable diseases, but vaccination coverage is low (1). Primary care providers often do not feel comfortable vaccinating patients with IBD (2), and gastroenterologists may assume vaccination is the responsibility of primary care providers (3). This may result in inadequate immunization of patients with IBD. Due to immunosuppressive therapy, the effectiveness and safety of vaccinations can be altered in patients with IBD (4, 5).

These evidence-based recommendations developed by the Canadian Association of Gastroenterology and endorsed by the American Gastroenterological Association, aim to provide guidance on immunizations in patients with inflammatory bowel disease. This publication is the second of 2 articles, and focuses on inactivated vaccines; part 1 is focused on live vaccines (6).

## Methods

The guideline panel used the GRADE (Grading of Recommendation Assessment, Development, and Evaluation) approach, including Evidence-to-Decision frameworks, to appraise evidence and formulate recommendations (7). The overall guideline development process, including panel formation, management of conflicts of interests, internal and external review, and organization approval was guided by Canadian Association of Gastroenterology policies and procedures derived from the Guideline International Network-McMaster Guideline Development Checklist (https://cebgrade.mcmaster.ca/guidelinechecklistonline.html) and was intended to meet standards for trustworthy guidelines by the Institute of Medicine and the Guideline International Network (8, 9). The recommendations were reviewed, commented on, and endorsed by the American Gastroenterological Association. The methods for guideline development were described in detail in part 1 (live vaccines) (6).

## Inactivated Vaccines in Patients With Inflammatory Bowel Disease

The individual recommendation statements are provided and include the strength of recommendation and certainty of evidence (CoE), and the voting result. This is followed by a discussion of the evidence considered for the specific recommendation. A summary of the recommendations is provided in Table 1. See Appendix 3 for the evidence profile tables with detailed CoE assessments (including description of study limitations, inconsistency, indirectness, imprecision, publication bias) and summary of findings, and the Evidence-to-Decision frameworks.

**Table 1:** Summary of Consensus Recommendations for Immunizations in Patients With Inflammatory Bowel Disease

Consensus recommendations
Inactivated vaccines
Hib
Recommendation 8A: In pediatric patients with IBD, 5 years of age and younger, we recommend Hib vaccine be given. GRADE: Strong recommendation, moderate CoE Recommendation 8B: In unimmunized pediatric patients with IBD, older than 5 years of age, we suggest Hib vaccine be given. GRADE: Conditional recommendation, low CoE
Recommendation 9: In unimmunized adult patients with IBD, we suggest Hib vaccine be given. GRADE: Conditional recommendation, very low CoE
HZ
Recommendation 10A: In adult patients with IBD 50 years of age and older, we recommend recombinant zoster vaccine be given. GRADE: Strong recommendation, moderate CoE
Recommendation 10B: In adult patients with IBD younger than 50 years of age, we suggest recombinant zoster vaccine be given. GRADE: Conditional recommendation, low CoE
Hepatitis B
Recommendation 11: In pediatric patients with IBD, we recommend hepatitis B vaccine be given. GRADE: Strong recommendation, moderate CoE
Recommendation 12A: In unimmunized adult patients with IBD with a risk factor for hepatitis B infection, we recommend hepatitis B vaccine be given. GRADE: Strong recommendation, moderate CoE
Recommendation 12B: In unimmunized adult patients with IBD without a risk factor for hepatitis B infection, we suggest hepatitis B vaccine be given. GRADE: Conditional recommendation, low CoE
Influenza
Recommendation 13: In pediatric patients with IBD, we recommend influenza vaccine be given. GRADE: Strong recommendation, moderate CoE
Recommendation 14: In adult patients with IBD, we recommend influenza vaccine be given. GRADE: Strong recommendation, moderate CoE
Pneumococcal vaccine
Recommendation 15: In pediatric patients with IBD, we recommend age-appropriate pneumococcal vaccines be given. GRADE: Strong recommendation, moderate CoE
Recommendation 16A: In adult patients with IBD not on immunosuppressive therapy, with a risk factor for pneumococcal disease, we recommend pneumococcal vaccines be given. GRADE: Strong recommendation, moderate CoE
Recommendation 16B: In adult patients with IBD on immunosuppressive therapy, we suggest pneumococcal vaccines be given. GRADE: Conditional recommendation, low CoE
Meningococcal vaccine
Recommendation 17: In pediatric patients with IBD, we recommend age-appropriate meningococcal vaccine be given. GRADE: Strong recommendation, moderate CoE
Recommendation 18: In adult patients with IBD with a risk factor for invasive meningococcal disease, we recommend meningococcal vaccines be given. GRADE: Strong recommendation, moderate CoE
Diphtheria, tetanus, and pertussis
Recommendation 19: In pediatric patients with IBD, we recommend age-appropriate tetanus, diphtheria, and pertussis-containing vaccines be given. GRADE: Strong recommendation, moderate CoE
Recommendation 20: In adult patients with IBD, we recommend tetanus, reduced diphtheria, and acellular pertussis/tetanus and diphtheria vaccine be given. GRADE: Strong recommendation, moderate CoE
HPV
Recommendation 21: In female patients with IBD aged 9***–***26 years, we recommend HPV vaccine be given. GRADE: Strong recommendation, moderate CoE
Recommendation 22: In male patients with IBD aged 9***–***26 years, we suggest HPV vaccine be given. GRADE: Conditional recommendation, very low CoE
Statements with no recommendations
No Recommendation B: In unimmunized adult patients with IBD on immunosuppressive therapy, the consensus group could not make a recommendation for or against giving double-dose hepatitis B vaccine.
No Recommendation C: In patients with IBD on maintenance biologic therapy, the consensus group could not make a recommendation for or against timing seasonal influenza immunization in relation to the biologic dose.
No Recommendation D: In adult patients with IBD not on immunosuppressive therapy and without a risk factor for pneumococcal disease, the consensus group could not make a recommendation for or against giving pneumococcal vaccines.
No Recommendation E: In adult patients with IBD without a risk factor for IMD, the consensus group could not make a recommendation for or against giving meningococcal vaccines.
No Recommendation F: In female and male patients with IBD aged 27***–***45 years, the consensus group could not make a recommendation for or against giving HPV vaccine.

### Haemophilus influenzae type b

**Risk of Haemophilus influenzae type b infection in people with inflammatory bowel disease compared to people without inflammatory bowel disease. Key evidence:** One cohort study found that adults with IBD had an increased adjusted odds ratio (aOR) of being hospitalized for *Haemophilus influenzae* type b (Hib) pneumonia (aOR, 1.34; 95% confidence interval [CI], 1.16–1.55) compared to a group without IBD (10). There were no significant differences in mortality rates. The CoE was very low, with the evidence being downgraded due to study limitations and indirectness. No studies on the risk of Hib infection in pediatric patients with IBD were identified.

***Recommendation 8A: In pediatric patients with IBD, 5 years of age and younger, we recommend Hib vaccine be given.***

GRADE: Strong recommendation, moderate CoE. Vote on PICO (patient population, intervention, comparator, and outcome) question: yes, 100%

***Recommendation 8B: In unimmunized pediatric patients with IBD, older than 5 years of age, we suggest Hib vaccine be given.***

GRADE: Conditional recommendation, low CoE. Vote on PICO question: yes, 100%

#### Key evidence

No studies assessing Hib vaccine in pediatric patients with IBD were found. A Cochrane systematic review found that Hib conjugate vaccine was safe and effective in reducing the risk of invasive Hib disease in children 5 years of age and younger (relative risk [RR], 0.20; 95% CI, 0.07–0.54) (11). Because there is no evidence to suggest that the Hib vaccines are harmful or less effective in patients with IBD, the evidence was anchored to the general population, and the CoE was assessed as moderate.

No studies were found for children over the age of 5 years in either the general population or with IBD, therefore, the CoE was downgraded to low for indirectness.

#### Discussion

The National Advisory Committee on Immunization (NACI), Public Health Agency of Canada Canadian Immunization Guide (12), and Centers for Disease Control and Prevention (CDC) (13) recommend Hib vaccine for children 5 years and younger. However, in unimmunized children older than 5 years of age (and adults), they recommend the vaccine only for patients with high-risk medical conditions (see Table 2) (12, 13). A World Health Organization (WHO) systematic review found Hib vaccination programs in children to be cost-effective across geographic regions and country income levels, with the incidence of Hib disease being the important determinant of cost-effectiveness (14).

**Table 2:** Risk Factors for Invasive Haemophilus influenzae Type b (12, 13)

Risk factors
Anatomic or functional asplenia (eg, sickle cell disease)
Human immunodeficiency virus infection
Primary immunodeficiency (eg, humoral defects, complement defects)
Receiving chemotherapy or radiation therapy for malignant neoplasms
Recipients of hematopoietic stem cell transplant
Recipients of solid organ transplant
Cochlear implants

The consensus group recommended routine use of Hib vaccine in children 5 years and younger. In children over 5 years, they suggested the vaccine on an individual basis because of the lower risk of invasive Hib, and the uncertain benefits of Hib vaccine, although harms are likely to be low.

***Recommendation 9: In unimmunized adult patients with IBD, we suggest Hib vaccine be given.***

GRADE: Conditional recommendation, very low CoE. Vote on PICO question: yes, 78%; uncertain/neutral, 22%

#### Key evidence

One small, observational study assessed the immune response to Hib vaccine in adults with IBD (15). Among patients who were starting thiopurine therapy, there was a significant increase in antibody titer 3 weeks post-Hib vaccination (15). No vaccine-induced exacerbation of IBD was reported in this study. The CoE was low and was downgraded to very low due to study limitations, indirectness, and imprecision.

#### Discussion

Hib disease is uncommon in adults and children aged over 5 years. The majority of cases in adults are caused by nontypable *Haemophilius influenzae*. In unimmunized adults, NACI and CDC recommend Hib vaccine only for certain high-risk medical conditions (Table 3) (12, 13). There are no published cost-effectiveness studies of Hib vaccine in adults. Patient acceptability can be impacted by cost, and difficulty accessing the vaccine. Given the increased risk of hospitalization for Hib pneumonia in adults with IBD that was found in an observational study (very low CoE) (10), the consensus group suggested shared decision-making regarding administration of the vaccine, especially among patients with risk factors for invasive Hib (Table 2).

**Table 3:** Risk Factors for Hepatitis B Infection (12, 16)

Risk factors
Immigrants from areas where there is a high prevalence of hepatitis B
Populations or communities in which hepatitis B is highly endemic
People with lifestyle risks for infection, including high-risk sexual activities, or injection drug use
People who have household contact with an infected individual
Health care and public safety workers at risk for exposure to blood or body fluids
Residents and staff of facilities for developmentally disabled persons
Persons in correctional facilities
Travelers to regions with increased rates of hepatitis B
People with chronic liver disease, kidney disease, human immunodeficiency virus infection, hepatitis C infection, or diabetes
People receiving repeated transfusions of blood or blood products (eg, hemophiliacs)

### Herpes Zoster

**Risk of herpes zoster in people with inflammatory bowel disease compared to people without inflammatory bowel disease. Key evidence:** Data from 9 cohort studies showed an increased risk of herpes zoster (HZ) in patients with IBD compared to the general population (1.2–1.8 times) (17–25). The CoE was downgraded to low due to study limitations and indirectness. Six cohort studies reported an increased risk of HZ with age (18–21, 23, 25). Among adults with IBD, there was low CoE that those 50 years and older, and very low CoE that those younger than 50 years, are at increased risk of HZ compared to adults without IBD 50 years and older. Data from 5 cohort studies showed that patients with IBD using immunosuppressive mono- and combination therapy had increased risks of HZ compared to patients with IBD not on immunosuppressive therapy or to the general population (18, 19, 23–26). The CoE was very low due to study limitations, imprecision, and inconsistency. Data from 3 single-arm randomized controlled trials (RCTs) provided very low CoE that tofacitinib (27), but not vedolizumab (28) or ustekinumab (29), is associated with an increased incidence of HZ in patients with IBD (27–29).

***Recommendation 10A: In adult patients with IBD 50 years of age and older, we recommend recombinant zoster vaccine be given.***

GRADE: Strong recommendation, moderate CoE. Vote on PICO question: yes, 100%

***Recommendation 10B: In adult patients with IBD younger than 50 years of age, we suggest recombinant zoster vaccine be given.***

GRADE: Conditional recommendation, low CoE. Vote on PICO question: yes, 89%; uncertain/neutral, 11%

#### Key evidence

Note that this section will address both the recombinant HZ vaccine (RZV) and the live attenuated HZ vaccine (LZV). No studies were found on the use of RZV in patients with IBD. The use of LZV was assessed in 4 observational studies in patients with IBD or selected immune-mediated diseases (30–33). The 2 larger studies showed a significant reduction in the risk of HZ (39%–46%) after LZV (30, 31). Among patients on thiopurines, there was no significant reduction in the risk of HZ with LZV (adjusted hazard ratio, 0.63; 95% CI, 0.30–1.33) (30). Patients with IBD could mount an immune response to LZV, but it was lower in those on low-dose immunosuppressive therapy (methotrexate ≤0.4 mg/kg/wk, azathioprine ≤3.0 mg/kg/d, 6-mercaptopurine ≤1.5 mg/kg/d) (33). No serious adverse events were reported (31, 32).

A large study, assessed as high quality by the CDC, demonstrated the efficacy and safety of RZV in immunocompetent adults 50 years and older (34, 35). This evidence was not downgraded for indirectness for IBD patients not on immunosuppressive therapy, because studies of LZV in patients with IBD support the findings in the general population, and data from separate studies, suggest RZV is more effective than LZV (34). It is possible that HZ vaccine may not be as effective in patients with IBD on immunosuppressive therapy (33). Hence, the evidence for efficacy was downgraded for indirectness to moderate for IBD patients on immunosuppressive therapy.

As there is serious imprecision with the estimate of serious adverse events related to the use of HZ vaccine in patients with IBD and all included studies assessed LZV, the evidence for safety for RZV was downgraded to moderate. Overall, there was moderate CoE that RZV is safe and effective in adults with IBD aged 50 years and older regardless of use of immunosuppressive therapy. As there were very few adults with IBD younger than 50 years of age included in these studies, the benefits and risks of the RZV are very uncertain. If the data are extrapolated from older adults, the CoE is downgraded to low due to indirectness.

#### Discussion

NACI and CDC recommend the 2-dose series of RZV as the preferred vaccine for prevention of HZ and related complications in immunocompetent adults 50 years and older (12, 34). NACI also suggests RZV be considered for immunocompromised adults aged 50 years and older on a case-by-case assessment of the benefits and risks (12).

IBD and immunosuppressive therapy can increase the risk of HZ infection, and although immunosuppression may decrease the efficacy of the vaccine, the consensus group recommended RZV in adults with IBD 50 years and older. The vaccination should be administered before initiating immunosuppressive therapy when possible.

For patients with IBD younger than 50 years of age, the evidence of risk and benefit is less compelling. No long-term studies on the duration of vaccine protection have been performed, so it remains unclear whether adults receiving the vaccine before age 50 years will continue to be protected as they age. Studies have shown high variability in acceptability based on patient age and experience with shingles or other complications of HZ infection (36, 37). Cost-effectiveness analyses suggest that RZV is more cost-effective than no vaccination or LZV for adults age 50 years and older (38, 39). Therefore, the consensus group suggested the RZV be discussed with patients younger than 50 years of age, and that patient preferences be considered.

For all patients, RZV is preferred over LZV because of evidence of superior efficacy and safety. However, when availability and access are an issue, LZV may be considered for those who are not immunosuppressed.

### Hepatitis B

**Risk of hepatitis B infection in people with inflammatory bowel disease compared to people without inflammatory bowel disease. Key evidence:** Data were available from 10 cross-sectional studies (40–49). Although older studies in Western countries showed a higher prevalence of past hepatitis B virus (HBV) infection among patients with IBD compared to the general population, this is not reported in more recent studies. In Eastern countries where HBV is endemic, the prevalence rates of past HBV among patients with IBD appeared to be higher than in the general population. The evidence was downgraded due to study limitations, indirectness, and inconsistency. Thus, there was very low CoE that adults with IBD have a comparable (or increased) risk of HBV compared to those without IBD. No studies on the risk of HBV infection in pediatric patients with IBD were identified.

***Recommendation 11: In pediatric patients with IBD, we recommend hepatitis B vaccine be given.***

GRADE: Strong recommendation, moderate CoE. Vote on PICO question: yes, 100%

#### Key evidence

No RCT or observational studies assessing the efficacy of HBV vaccine in pediatric patients with IBD were found. A systematic review of 4 RCTs found that the vaccine reduced the incidence of HBV (RR, 0.28; 95% CI, 0.20–0.40) among infants born to mothers positive for HBV surface antigen compared with placebo or no intervention (50). In 2 large, long-term observational studies, HBV vaccination was associated with decreases in the incidence of hepatocellular carcinoma (60.1%) and mortality due to chronic liver diseases (92.0%) (51, 52).

In 4 cross-sectional studies, vaccine-related seroconversion rates against HBV (hepatitis B surface antibody [anti-HBs] >10 IU/L) ranged from 28% to 71.3% in pediatric patients with IBD (53–56). However, these studies cannot differentiate between lack of primary antibody response and loss of antibody levels over time. One study in children with IBD compared to healthy controls found that the seroconversion rate after primary HBV vaccination was significantly lower (70.2% vs 90%; *P* = .02), but increased to 85.1% after a single-dose booster was given to nonresponders (57). There was no significant association between use of immunosuppressive therapies and vaccination response in these studies (53–57).

A CDC analysis of the safety of HBV vaccine in 6 studies in patients with diabetes reported no serious adverse events (58).

The CoE was anchored to the general population. CoE for effectiveness was downgraded from high to moderate due to indirectness because studies suggested that HBV vaccine may be less immunogenic in patients with IBD. The evidence for safety was downgraded from high to moderate due to indirectness.

#### Discussion

Both CDC and NACI recommend routine HBV vaccine of children (12, 16). The consensus group concluded that the benefits of HBV vaccine far outweigh risks in pediatric patients with IBD. The clinical significance of loss of anti-HBs titers over time in patients with IBD is unknown. Long-term studies performed in different epidemiologic contexts have confirmed that clinical HBV infection rarely occurs among successfully vaccinated people, even though anti-HBs titers decline to <10 IU/L, likely due to a robust anamnestic response in immunocompetent individuals (59). However, clinically significant HBV infection has been documented in immunocompromised responders (human immunodeficiency virus and those undergoing hemodialysis) who do not maintain anti-HBs >10 IU/L, indicating that immune memory may not confer long-term immunity (59, 60). For other immunocompromised patients (eg, IBD patients on immunosuppressive therapy), the need for booster is uncertain.

For immunocompromised individuals, NACI recommends anti-HBs serology, within 1 to 6 months of completion of the series, followed by periodic monitoring based on the severity of the immunocompromised state and the presence of HBV risk factors (12) (see also Recommendation 12).

A cost-effectiveness study found that a strategy of universal HBV vaccination of newborns led to an incremental cost per year of life saved of $3332 (1989 costs) (61). Patient acceptance among students and parents for universal HBV vaccination was high (62).

The consensus group recommends HBV vaccine for all pediatric patients with IBD, with a preference for the 3-dose vaccine.

***Recommendation 12A: In unimmunized adult patients with IBD with a risk factor for hepatitis B infection, we recommend hepatitis B vaccine be given.***

GRADE: Strong recommendation, moderate CoE. Vote on PICO question: yes, 100%

***Recommendation 12B: In unimmunized adult patients with IBD without a risk factor for hepatitis B infection, we suggest hepatitis B vaccine be given.***

GRADE: Conditional recommendation, low CoE. Vote on PICO question: yes, 100%

#### Key evidence

A CDC assessment of evidence for HBV vaccine among adults with diabetes estimated a 63% reduction in risk of HBV infection (RR, 0.37; 95% CI, 0.29–0.48, number needed to treat = 261) (58, 63). The seroprotection rate was 91.6% (95% CI, 87.6%–94.4%).

Among adults with IBD, HBV vaccine immune response (anti-HBs antibody >10 IU/L) occurred in 61% (95% CI, 53%–69%), which appeared to be reduced compared to the general population (64). Younger age at time of vaccination and vaccination during remission were associated with improved serologic response, whereas use of immunosuppressive therapy (corticosteroids, immunomodulators, and anti-tumor necrosis factor [anti-TNF] biologics) was associated with a reduced response. No serious adverse events were reported (64). In a study of adults with IBD initiating anti-TNF therapy, the seroprotection rate after primary vaccination was 43.5% (65). In contrast, an RCT in healthy individuals found that vedolizumab therapy 4 days before HBV vaccination had no effect on immune response (anti-HBs antibody >10 IU/L) compared to placebo (88.5% and 90.3%, respectively) (66).

The overall CoE was anchored to the individuals in the general population at high risk for HBV. However, evidence suggests that the vaccine may not be as immunogenic in adults with IBD, therefore, the CoE for effectiveness and safety were downgraded from high to moderate for adult patients with IBD with a risk factor for HBV. There was no direct evidence for patients who are not at risk of HBV infection, therefore, the evidence was further downgraded to low for that patient population.

#### Discussion

Both CDC and NACI recommend 3-dose HBV vaccine for unvaccinated adults at risk of HBV infection (Table 3) (12, 16). CDC suggests a 2-dose Heplisav-B vaccine be used in persons aged 18 years and older based on immunogenicity data, but long-term safety has yet to be determined (16). The benefits of 2 doses administered over 1 month make this an important option for prevention of HBV in at-risk persons. However, no study has evaluated this vaccine in patients with IBD. Serious adverse effects with HBV vaccines are rare, but include anaphylaxis (16, 67). There have been reports of reactivation of HBV in chronic carriers while on immunosuppressive therapy (68, 69), which was more common with the use of 2 or more medications (69). Based on good evidence for efficacy and safety, the consensus group recommended vaccination in adults with IBD who have risk factors for HBV.

Chronic infection has been shown to develop more frequently in patients who are immunosuppressed (70), and can result in liver cirrhosis, cancer, and failure, as well as death (16). Because patient risk factor status can change over time, IBD and immunosuppressive therapy can reduce the response to HBV vaccination, and long-term outcomes of infection can be life-threatening, the consensus group was in favor of vaccination for unimmunized adults with IBD without risk factors for HBV. However, because of the low CoE, this was a conditional recommendation, meaning that risks for and consequences of HBV infection should be discussed with patients, and use of HBV vaccine should consider patient preferences.

In a cost-effectiveness study, HBV vaccination was less costly and more effective in adult high-risk populations (eg, HBV incidence >5%), and universal vaccination of the general population yielded an incremental cost per year of life saved of $54,524 (1989 costs) (61). An analysis of HBV vaccine in adults with type 1 diabetes (a chronic condition, as is IBD) was moderately cost-effective at $75,094 (2010 costs) per quality-adjusted life-year (QALY) gained (71).

The issues of monitoring serologic titers post primary vaccination, revaccination, and booster doses in adults with IBD were discussed by the consensus group, although they were not predefined PICO questions. There was evidence that although anti-HBs titers can decline to undetectable levels, HBV infection rarely occurs among successfully vaccinated individuals (59, 72). Because protection may be attributable to immunologic memory rather than anti-HBs levels (59, 72), the relevance of serologic testing is not fully known. The potential benefits and harms of measuring anti-HBs titers and giving booster doses when the titer is low are uncertain. In addition, the target anti-HBs titer that would warrant a booster dose among patients with IBD is unknown. Very-low CoE from 4 observational studies of revaccination showed a response rate of about 50% (range, 42%–68%) (65, 72–74). One additional study published outside the literature search showed that in immunocompromised patients with IBD who failed primary HBV vaccination, 3 additional doses were more likely to be seroprotective than 1 or 2 doses (62.9% vs 40.2%; aOR, 1.77; *P* = .01; aOR, 1.9; *P* = .03) (75). However, because HBV infection has been documented in immunocompromised responders who do not maintain anti-HBs levels (60), CDC recommends annual testing and a booster dose when levels decrease to <10 IU/L (13). The consensus group concluded that there were too many unanswered questions around these issues to develop recommendations at this time, including in whom and how often to monitor titers, and threshold titers that warrant revaccination or booster doses in patients with IBD.

***No Recommendation B (see Appendix 3, 5C): In unimmunized adult patients with IBD on immunosuppressive therapy, the consensus group could not make a recommendation for or against giving double-dose hepatitis B vaccine.***

GRADE for PICO: very low CoE. Vote on PICO question: yes, 11%; uncertain/neutral, 67%; no, 22%

#### Key evidence

Two observational studies assessing double-dose vs standard-dose HBV vaccination yielded inconsistent results (76, 77). One study suggested no difference in serologic response between double and standard dose in patients with autoimmune conditions (including IBD) (77), and the other suggested greater serologic response with double dose in patients with IBD (76). In addition, 2 cohort studies in patients with IBD suggested that the serologic response was low with use of an accelerated schedule of double-dose HBV vaccine (65, 72). The overall CoE was very low.

#### Discussion

In light of conflicting results and increased cost, the consensus group concluded that there was insufficient evidence to recommend for or against double-dose HBV vaccine.

### Influenza

**Risk of influenza infection in people with inflammatory bowel disease compared to people without inflammatory bowel disease. Key evidence:** Two cohort studies examined the risk of influenza in patients with IBD (10, 78). One found that patients with IBD had an increased risk for influenza infection (adjusted hazard ratio, 1.28; 95% CI, 1.19–1.37), and a significantly higher 30-day influenza-related hospitalization rate compared with non-IBD controls (5.4% vs 1.85%; *P* < .001) (78). The other found increased odds of hospitalization in a subgroup of low-income patients with UC (aOR, 1.86; 95% CI, 1.46–2.37), but not in the overall IBD group; however, this included inpatient data only (10). The CoE was downgraded from high to low due to study limitations.

***Recommendation 13: In pediatric patients with IBD, we recommend influenza vaccine be given.***

GRADE: Strong recommendation, moderate CoE. Vote on PICO question: yes, 100%

#### Key evidence

A systematic review found that inactivated vaccines reduce the risk of influenza in healthy children from 30% to 11% (RR, 0.36; 95% CI, 0.28–0.48; number needed to treat = 5) (79). Evidence from 4 observational studies assessing trivalent inactivated influenza vaccines suggested that pediatric patients with IBD can mount appropriate immunologic responses to influenza A components, but response may be attenuated to the B component (80–83). Immunosuppressive therapy may further reduce the immunologic response.

A large systematic review found inactivated influenza vaccines to be generally safe with rare serious adverse events in the general population (84). In pediatric patients with IBD, 5 observational studies reported no serious adverse events, including no increased risk of flare of IBD (80–82, 85).

The evidence for efficacy and safety were anchored to the general population. The CoE for efficacy was downgraded from high to moderate because studies suggested that inactivated influenza vaccines may be less immunogenic in patients with IBD. The evidence for safety was downgraded from high to moderate due to indirectness.

#### Discussion

Both CDC and NACI recommend routine annual influenza vaccination of individuals 6 months of age or older, with CDC setting an age cutoff of 59 months (12, 86). The options include inactivated influenza vaccine, recombinant influenza vaccine, or live attenuated influenza vaccine. However, live attenuated influenza vaccine is not recommended to those receiving immunosuppressive therapy or their household contacts.

In a systematic review of economic evaluations, the majority of the studies found that childhood influenza vaccination was cost-effective (87).

The consensus group concluded that there was good evidence to recommend giving influenza vaccine to pediatric patients with IBD.

***Recommendation 14: In adult patients with IBD, we recommend influenza vaccine be given.***

GRADE: Strong recommendation, moderate CoE. Vote on PICO question: yes, 100%

#### Key evidence

In 2 systematic reviews, inactivated influenza vaccines reduced the risk of influenza and influenza-like illness in healthy adults, 65 years and younger (88), and those older than 65 years (89). These systematic reviews concluded that CoE in the younger group was moderate for both outcomes but in the elderly was moderate for the outcome of influenza-like illness and low for the outcome of influenza, because of uncertainty over how influenza was diagnosed in the older trials. However, in our analysis, the CoE was not downgraded for the older population because influenza-like illness was deemed a critical outcome. Symptoms of influenza-like illness have been shown to have a positive predictive value of 79% for influenza (90).

Observational data from 6 cohort studies (91–96) and 4 RCTs (97–100) assessing inactivated influenza vaccines suggested that adults with IBD can mount appropriate immunologic responses (80–83). Immunosuppressive therapy can reduce the immunologic response, particularly when combination therapy is used.

The 2 systematic reviews in healthy adults, and the 10 other studies in adults with IBD showed no serious adverse events associated with the use of inactivated influenza vaccine (88, 89, 91–100).

The evidence for efficacy and safety were anchored to the general population. The CoE for efficacy remained moderate because studies suggesting reduced immunogenicity in patients with IBD showed that the European Union Committee for Medicinal Products for Human Use criteria for effective immunogenicity were met in the majority of patients. The evidence for safety was downgraded from high to moderate due to indirectness.

#### Discussion

CDC and NACI recommend routine annual influenza vaccination of all individuals, particularly those at high risk for influenza-related complications or hospitalization (Table 4) (12, 86).

**Table 4:** Risk Factors for Influenza-Related Complications or Hospitalization (12, 86)

Risk factors
All individuals 6 y or older (NACI) or aged 6–59 mo (Advisory Committee on Immunization Practices)
All adults 65 y or older (NACI) or 50 y or older (Advisory Committee on Immunization Practices)
Individuals who have chronic pulmonary or cardiovascular, renal, hepatic, neurologic, hematologic, or metabolic disorders
Individuals who are immunosuppressed (due to underlying disease and/or therapy)
Women who are or will be pregnant during the influenza season
Children and adolescents who are receiving long-term salicylate-containing medications, because of the risk for Reye syndrome after influenza
Residents of nursing homes and other chronic care facilities
Indigenous peoples
Individuals who are extremely obese (body mass index >40 kg/m^2^)

In a systematic review in adults, the cost-effectiveness of influenza vaccination ranged from $8000 to $39,000 (2015 costs) per QALY (101). In assessments for adults aged 65 years and older the cost-effectiveness ratios were cost-saving in some studies and up to $15,300 per QALY in others (101).

As the CoE and the strength of recommendation were the same for younger (65 years of age and younger) and older (older than 65 years of age) adult patients with IBD, the 2 populations were grouped together, and the consensus group recommend giving influenza vaccine to all adult patients with IBD.

***No Recommendation C (see Appendix 3, 6C): In patients with IBD on maintenance biologic therapy, the consensus group could not make a recommendation for or against timing seasonal influenza immunization in relation to the biologic dose.***

GRADE for PICO: low CoE. Vote on PICO question: uncertain/neutral, 33%; no, 67%

#### Key evidence

One RCT suggested no significant difference in immunogenicity when influenza vaccine was given at the time of biologic infusion (infliximab) compared to midway between infusions (98). No serious adverse events were reported, and changes in disease activity score were not related to timing of the vaccine. The CoE was downgraded from high to low due to study limitations and imprecision. The majority of patients in this study were adults, therefore, the CoE in pediatric patients would be further downgraded to very low.

#### Discussion

There is very limited evidence that the timing of influenza vaccination relative to that of biologic infusion affects the effectiveness and safety of influenza vaccine in patients with IBD. There are pros and cons to each strategy from a practical point of view. If the vaccine and biologic are given at the same time, it may make it difficult to attribute an adverse effect to one or the other. However, in patients with poor access to care, the infusion visit may be the only opportunity to administer the vaccine. The consensus group concluded that there was insufficient data to make a recommendation regarding the timing of influenza vaccination in relation to the biologic dose.

### Pneumococcal Vaccine

**Risk of pneumococcal disease in people with inflammatory bowel disease compared to people without inflammatory bowel disease. Key evidence:** Some data suggest a higher risk of pneumonia and invasive pneumococcal disease (IPD) in patients with immunocompromising conditions and IBD compared to the general population (10, 101–106). In general, these data could not determine whether increased risks were attributable to IBD itself or to immunosuppressive therapy. In a systematic review, the pooled incidence of IPD was 65/100,000 person years in patients with chronic inflammatory diseases (including IBD) compared to 10/100,000 in healthy controls (102). An additional observational study in patients with autoimmune diseases, including Crohn’s disease, reported an increased risk of IPD, which increased with increasing number of comorbid conditions (103). Two observational studies restricted to patients with IBD found a risk of IPD that was about 1.5- to 2-fold higher in patients with IBD compared to those without (104, 105). However, a cohort study failed to show increased odds of hospitalization or mortality due to *Streptococcus pneumoniae* among patients with IBD compared to those without (10).

The CoE was downgraded from high to low due to study limitations.

***Recommendation 15: In pediatric patients with IBD, we recommend age-appropriate pneumococcal vaccines be given.***

GRADE: Strong recommendation, moderate CoE. Vote on PICO question: yes, 100% (n = 8)

#### Key evidence

In a systematic review, pneumococcal vaccines were effective in preventing IPD (RR, 0.20; 95% CI, 0.10–0.42), and clinical pneumonia (RR, 0.94; 95% CI, 0.91–0.98) in healthy children younger than 2 years (107). An observational study using pneumococcal conjugate 13-valent (PCV13) showed that pediatric patients with IBD can mount an appropriate immunologic response, but immunosuppressive therapy may reduce the response (108). Another small study suggested that response to pneumococcal polysaccharide 23-valent (PPSV23) may be impaired in pediatric patients with IBD (majority on immunosuppressive therapy) (109).

In a systematic review, serious adverse events causally related to pneumococcal vaccines were rare in children up to 12 years old (110). No serious adverse events were reported with vaccination in patients with IBD in the observational studies (108, 109).

The CoE for effectiveness and safety was anchored to the general population and downgraded from high to moderate for pediatric patients with IBD.

#### Discussion

NACI recommends routine pneumococcal vaccine for children up to 5 years of age, and those older than 5 years at high risk of IPD due to underlying medical conditions, or due to current or anticipated use of immunosuppressive therapy (12). Similarly, the CDC recommends routine administration of pneumococcal vaccine for all children younger than 2 years, catch-up doses for unimmunized or underimmunized children 2–4 years, and immunization for children older than 2 years with certain medical conditions or using immunosuppressive drugs (111). NACI recommends that individuals with immunocompromising conditions and those anticipating or undergoing immunosuppressive therapy should receive PCV13 and PPSV23 vaccines (12). When both vaccines are required, PCV13 should be given first, followed by PPSV23 at least 8 weeks later.

Comparative data on specific vaccine types and dosing schedules in patients with IBD were not available, therefore, the consensus group was unable to make specific suggestions. CDC and NACI provide guidance for the general population and immunocompromised patients; however, it is unknown whether the recommended schedules are appropriate for patients with IBD.

A global cost-effectiveness modeling analysis found large benefits with the use of PCV in terms of lives saved, disability averted, and cost-effectiveness (112).

Pediatric patients with IBD are often on immunosuppressive therapy or will imminently require such therapy; because this can impact the immune response to pneumococcal vaccines, the consensus group recommends that pediatric patients be administered age-appropriate pneumococcal vaccines as soon as possible.

***Recommendation 16A: In adult patients with IBD not on immunosuppressive therapy, with a risk factor for pneumococcal disease, we *recommend* pneumococcal vaccines be given.***

GRADE: Strong recommendation, moderate CoE. Vote on PICO question: yes, 88%; uncertain/neutral, 12%

***Recommendation 16B: In adult patients with IBD on immunosuppressive therapy, we suggest pneumococcal vaccines be given.***

GRADE: Conditional recommendation, low CoE. Vote on PICO question: yes, 100%

***No Recommendation D (see Appendix 3, 7A.2.2): In adult patients with IBD not on immunosuppressive therapy and without a risk factor for pneumococcal disease, the consensus group could not make a recommendation for or *against* giving pneumococcal vaccines.***

GRADE for PICO: moderate CoE. Vote on PICO question: yes, 12%; uncertain/neutral, 88%

#### Key evidence

A systematic review of 18 RCTs found pneumococcal vaccine to be effective in reducing IPD (OR, 0.26; 95% CI, 0.14–0.45) and all-cause pneumonia (OR, 0.72; 95% CI, 0.56–0.93) in adults in the general population (113). In subgroup analyses, there was evidence of a protective effect against IPD in healthy adults, but not in adults with chronic disease or highly immunosuppressed individuals of any age due to imprecision (113). In patients with IBD, a cross-sectional study found lower 1-year mortality rates among adults who were vaccinated compared to those who were not (2.1% vs 4.5%; *P* < .001) (114). Data from 5 observational studies suggested that pneumococcal vaccine immunogenic response rates in patients with IBD not on immunosuppressive drugs are similar to those seen in the general population (15, 115–118). In a case-controlled study, immune response rates were similar between adults with IBD not on immunosuppressive therapy (80%) and age-matched healthy controls (85%), but lower in patients on combination immunosuppressive therapy (45%) (118). Other studies also suggest that immunosuppressive therapy may reduce the immunologic response (115–117).

No serious adverse events with pneumococcal vaccine were reported in a systematic review of studies in patients with systemic lupus erythematosus (119) or in 4 observational studies in patients with IBD (15, 115–117).

The evidence for effectiveness was anchored to the general population. In patients with IBD, with or without a risk factor, the CoE was moderate and was not downgraded because data suggest that immune response rates are similar in IBD and general populations. For patients on immunosuppressive therapy, the CoE was downgraded to low due to indirectness because therapy may impair the immunogenic response. The CoE for safety of pneumococcal vaccines in adults with IBD was moderate.

#### Discussion

NACI recommends pneumococcal vaccine for adults who are at high risk of IPD (Table 5), those who are residents of long-term care facilities, and those who are 65 years and older regardless of risk (12). Similarly, CDC recommends pneumococcal vaccine for adults 19–64 years with risk factors (Table 5) and all adults 65 years and older (111). Based on moderate CoE, the consensus group made a strong recommendation for pneumococcal vaccines in adult patients with IBD and risk factors, who are not on immunosuppressive therapy.

**Table 5:** Risk Factors for Invasive Pneumococcal Disease (12, 120)

Risk factors
Very young (typically younger than 5 y, especially those attending childcare centers)
Adults 65 y or older
Functional or anatomic asplenia
Cochlear implants
Chronic cerebrospinal fluid leak
Lifestyle factors (eg, cigarette smoking, alcoholism, illicit drug use, homelessness)
Individuals with underlying medical conditions (eg, chronic lung, heart, liver or kidney disease, or diabetes mellitus)
Individuals who are immunosuppressed (due to underlying disease and/or therapy)

For patients who are immunocompromised due to underlying disease or therapy, both NACI and CDC recommend both PCV13 and PPSV23 vaccines as described in Recommendation 15 (12, 111). Although there was low CoE of effectiveness in patients with IBD who are on immunosuppressive therapy, there is a high burden of pneumococcal disease in immunocompromised adults. A large observational study found that rates of all-cause pneumonia and IPD in immunocompromised adults were 5.3 and 10.5 higher than the rates in healthy adults (120).

Therefore, the consensus group suggested pneumococcal vaccines be given to all adults with IBD on immunosuppressive therapy, regardless of other risk factors. Nevertheless, all appropriate vaccinations should be given as soon as possible, and ideally before initiation of immunosuppressive therapy (see Recommendation 2 in part 1 on live vaccines (6)). A cost-effectiveness analysis in immunocompromised individuals found that single-dose PCV13 was cost-effective compared to no vaccination, at $70,937 (2009 costs) per QALY, and more cost-effective than the combination of PCV13 and PPSV23 (121). However, another analysis concluded that the use of both vaccines for immunocompromised adults could potentially be cost-effective (122). Because of the absence of comparative data in patients with IBD, the consensus group was unable to make suggestions regarding specific vaccine types and dosing schedules.

In adult patients with IBD who are not on immunosuppressive therapy and not at high risk because of age or other factors, the consensus group could not make a recommendation for or against giving pneumococcal vaccines. The cost–benefit ratio was uncertain in this group; however, consideration should be given to the need for immunosuppressive therapy in the future.

### Meningococcal Vaccine

**Risk of meningococcal disease in people with inflammatory bowel disease compared to people without inflammatory bowel disease. Key evidence:** There is very low CoE of an increased risk of meningococcal disease in patients with IBD. This is related to a few case reports of a potential association between hyposplenism and IBD based on indirect measurements of splenic function (123–129). However, the prevalence of functional hyposplenism in patients with IBD is uncertain. Hyposplenism has been reported in other gastrointestinal and autoimmune conditions, and asplenia and hyposplenism are risk factors for invasive meningococcal disease (IMD) (12, 13). A decreased ability to mount an anti-polysaccharide response can lead to an increased risk of infection by encapsulated organisms, such as *Neisseria meningitides* (130).

No studies have assessed the risks of meningococcal infection in patients with IBD, or the role of functional hyposplenism. A case of IMD was reported in a patient with hyposplenism (131), and another case in a patient with Crohn’s disease after anti-TNF therapy (132).

There was very low CoE suggesting a higher risk of functional hyposplenism in patients with IBD compared to the general population and, if so, whether this is associated with a higher risk of *N meningitides*.

***Recommendation 17: In pediatric patients with IBD, we recommend age-appropriate meningococcal vaccine be given.***

GRADE: Strong recommendation, moderate CoE. Vote on PICO question: yes, 100%

#### Key evidence

A systematic review of RCTs found the serogroup A vaccine to be 95% (95% CI, 89%–99%) effective against meningococcal A meningitis for the first year in the general population (133). A systematic review of observational studies found meningococcal serogroup C vaccines to be highly immunogenic for preventing meningococcal C meningitis and septicemia (134). Routine immunization programs have led to dramatic reductions in the incidence of meningococcal serogroup C disease (134).

In a WHO assessment of evidence for meningococcal serogroup C conjugate vaccines and quadrivalent meningococcal vaccines in children, the CoE was rated as moderate for efficacy and safety (135). A CDC review of the evidence for serogroup B meningococcal vaccines in adolescents, young adults, and those at high risk rated the CoE for immunogenicity as low (11).

The evidence was anchored at the general population and not downgraded due to indirectness because patients with IBD are likely at similar or increased risk of developing meningococcal infections compared to the general population. In addition, there is no evidence to suggest that the vaccines are less safe or effective in patients with IBD.

#### Discussion

NACI recommends routine childhood meningococcal vaccination according to jurisdictional schedules, and periodic booster doses every 3–5 years for individuals at high risk (Table 6) (12). Routine meningococcal vaccination is recommended for children who are at increased risk for IMD by both CDC and NACI (12, 13).

**Table 6:** Risk Factors for Invasive Meningococcal Disease or Increased Risk of Exposure (12, 13)

Risk factors
Functional or anatomic asplenia
Complement and antibody deficiencies
Persons with human immunodeficiency virus infection
Travel to areas with high rates of endemic meningococcal disease or transmission
Exposure to a confirmed case or during disease outbreak
Risk of occupational exposure to *Neisseria meningitidis* (eg, clinical laboratory personnel)
Military personnel who are at increased risk (eg, recruitment training, deployment)

A US analysis found that routine meningococcal conjugate vaccination of children of different age groups was cost-effective at a cost of $105,000 to $271,000 (2003 costs) per QALY (136). Childhood vaccination would be cost-effective in areas with a high incidence of meningococcal disease. In contrast, universal meningococcal serogroup B vaccine was not shown to be cost-effective in infants or college-aged young adults (137, 138).

Because there is little evidence to suggest that pediatric patients with IBD are substantially different than the general population in terms of risk for developing IMD or responsiveness to meningococcal vaccines, the consensus group recommended that age-appropriate meningococcal vaccines be given according to locally available schedules.

***Recommendation* 18: In adult patients with IBD with a risk factor for invasive meningococcal disease, we recommend meningococcal vaccines be given.**

GRADE: Strong recommendation, moderate CoE. Vote on PICO question: yes, 100%

***No Recommendation E (see Appendix 3, 8A.2): In adult patients with IBD without a risk factor for IMD, the consensus group could not make a recommendation for or against giving meningococcal vaccines.***

GRADE for PICO: moderate CoE. Vote on PICO question: uncertain/neutral, 78%; no, 22%

#### Key evidence

See Recommendation 17.

#### Discussion

NACI recommends routine meningococcal vaccination in childhood and in adolescence (11). The availability and funding for each meningococcal vaccine type will depend on jurisdiction. For other adults, both NACI and CDC recommend meningococcal vaccines only for those with risk factors for IMD (Table 6) (12, 13). Data are mainly from studies in healthy adolescents and young adults, and not individuals with risk factors for meningococcal infections.

A cost-effectiveness analysis, which accounted for herd immunity, found that vaccination was no longer cost-effective when IMD incidence was fewer than 12/100,000 persons, using a threshold of US$100,000/QALY (2012 costs) (139). This analysis assessed vaccination during an outbreak of IMD among men who have sex with men with or without human immunodeficiency virus infection. A 2018 analysis, found that universal serogroup B vaccination was not cost-effective in college-aged young adults (138). The incidence of IMD in Canada was estimated at 0.55 cases per 100,000 persons per years (2006–2011), with the greatest risk being in infants under 1 year of age (140). Therefore, universal vaccination of adults is likely not cost effective.

There is strong evidence of a herd immunity effect with serogroup C meningococcal vaccines (141), and although there is a waning of antibody levels initially, a routine booster vaccination for adolescents or young adults is likely to maintain long-term individual and herd immunity (142). Evidence for herd immunity effect with meningococcal serogroup B vaccines is less certain (143).

The consequences of IMD can be life-threatening. An analysis of Canadian cases of IMD over a decade (2002–2011) reported high rates of mortality (8.4%) and complications (18%), including hearing loss, amputation, renal dysfunction, and seizures (144). In light of this, and the evidence for efficacy and safety, the consensus group recommended vaccination for adults with IBD with risk factors for IMD. However, given the low incidence of IMD, the consensus group was uncertain of the benefit of vaccination of all adults and, thus, could not make a recommendation for or against vaccination for adults with IBD without a risk factor.

### Diphtheria, Tetanus, or Psertussis

**Risk of diphtheria, tetanus, or pertussis in people with inflammatory bowel disease compared to people without inflammatory bowel disease. Key evidence:** No studies on the risk of tetanus, diphtheria, or pertussis infection in adult or pediatric patients with IBD were identified. Diphtheria is rare in North America, but is endemic in many developing countries, and has shown resurgence in countries with low vaccine coverage (12). Tetanus is relatively uncommon in most developed countries. Annual rates are low, with an average of 4 per year in Canada and a total of 33 in 2017 in the United States (12, 145).

Pertussis is endemic worldwide, even in regions with high vaccination coverage. Although North America has experienced a decline since the introduction of vaccination programs, infants and children remain at the highest risk for disease (12, 146). Pertussis peaks continue to occur at 2- to 5-year intervals (12, 146).

***Recommendation 19: In pediatric patients with IBD, we recommend age-appropriate tetanus, diphtheria, and pertussis-containing vaccines be given.***

GRADE: Strong recommendation, moderate CoE. Vote on PICO question: yes, 100%

***Recommendation 20: In adult patients with IBD, we recommend tetanus, reduced diphtheria, and acellular pertussis/tetanus and diphtheria vaccine be given.***

GRADE: Strong recommendation, moderate CoE. Vote on PICO question: yes, 100%

#### Key evidence

A WHO assessment of evidence for effectiveness of multicomponent diphtheria, tetanus, and acellular pertussis vaccines in healthy children and adults rated the CoE as high (135). The analysis for pertussis included a systematic review of 6 RCTs showing the vaccine was 84%–85% effective in preventing pertussis (147). In addition, strong evidence from observational studies supported the effectiveness of diphtheria and tetanus toxoid vaccination (135).

Most observational studies have shown no significant differences in immunogenic response between pediatric or adult patients with IBD irrespective of immunosuppressive therapy, or healthy controls (15, 53, 148–151). One study suggested that adults with IBD may have lower diphtheria and pertussis antibody concentrations compared to healthy subjects, with those on anti-TNF therapy having lower concentrations compared to those on thiopurine monotherapy (151). However, the clinical significance of these findings is uncertain, given that anamnestic response was not assessed.

The WHO assessment of evidence for safety included a systematic review that found no significant risk of serious adverse events with acellular pertussis vaccines (134, 147). No serious adverse events related to the diphtheria and tetanus toxoids, and acellular pertussis (DTaP) vaccines were reported to the Vaccine Adverse Event Reporting System (152).

The evidence was anchored to the general population. The CoE was not downgraded for efficacy because there is no evidence that the vaccines are less effective in patients with IBD. The evidence for safety was downgraded from high to moderate due to imprecision.

#### Discussion

CDC and NACI recommend a routine 5-dose series of a vaccine containing DTaP and inactivated poliomyelitis vaccine for infants and young children, with 1 adolescent booster dose of tetanus, reduced diphtheria, and pertussis vaccine (12, 13, 146). A tetanus, reduced diphtheria, and pertussis vaccine should be administered to adolescents, adults who did not receive a pertussis-containing vaccine in adulthood, and pregnant women for every pregnancy regardless of immunization history, with ongoing tetanus and diphtheria booster vaccines every 10 years.

A routine childhood immunization program with 9 vaccines, including DTaP, reported a net savings of US$13.5 billion in direct cost and US$68.8 billion in total societal costs (2009 costs) (153). In another analysis, DTaP vaccine resulted in net savings of more than $22.5 million in societal costs (1997 costs) (154). A program to increase the uptake of several vaccines, including DTaP, among adults at high risk of complications was cost-effective from a societal perspective (155).

Based on evidence supporting efficacy, safety, and cost-effectiveness of DTaP, the consensus group recommended that both pediatric and adult patients with IBD maintain full immunization status, including booster doses as needed.

### Human Papillomavirus

**Risk of human papillomavirus in people with inflammatory bowel disease compared to people without inflammatory bowel disease. Key evidence:** Because cervical cancer is almost exclusively caused by human papillomavirus (HPV) infection, the risk of developing cervical cancer among patients with IBD was assessed. Data were available from 12 observational studies (156–160) (8 of which were included in a systematic review (156)). Outcomes included cervical abnormalities (atypical squamous cells of undetermined significance, cervical intraepithelial neoplasia (CIN) 1 or worse, or cervical cancer), abnormal Pap smears, low grade or high-grade dysplasia, and cancer. Overall, the data were conflicting as to whether patients with IBD have an increased risk of developing cervical dysplasia and cancer; however, the risk may be increased in those on immunosuppressive therapy (eg, corticosteroids, immunomodulators, anti-TNF agents).

The CoE was downgraded from high to very low due to study limitations, inconsistency, and indirectness. Most studies did not adjust for known risk factors of cervical cancer. In addition, frequent physician visits may lead to a higher rate of detection of cervical abnormalities in patients with IBD compared to the general population.

Cases of anal squamous cell carcinoma have been described in patients with IBD, and although its incidence may be raised in patients with Crohn’s disease compared to the general population, it is a very rare disease (161).

***Recommendation 21: In female patients with IBD aged 9–26 years, we recommend HPV vaccine be given.***

GRADE: Strong recommendation, moderate CoE. Vote on PICO question: yes, 100%

#### Key evidence

A CDC GRADE assessment of evidence for quadrivalent (4vHPV) and 9-valent (9vHPV) vaccines was conducted for females in various age groups (63). Trials included RCTs with the 9vHPV and HPV4 vaccines, as well as data demonstrating non-inferior immunogenicity of 9vHPV compared with HPV4. The review showed that HPV vaccine was effective for the prevention of CIN ≥2 in females aged 9–26 years.

One before-and-after study showed that post-vaccination titers with HPV4 vaccine in female patients with IBD aged 9–26 years on immunosuppressive therapy were comparable to those seen in healthy controls (162). The titers decreased over time, and the seroresponse to HPV type 18 may be lower in patients with IBD, but the clinical significance of this is unknown.

The CoE for effectiveness was anchored to the general population and was not downgraded for patients with IBD because of the study showing comparable immunogenicity with healthy controls (162). The evidence for safety was downgraded from high to moderate due to indirectness.

The relationship between CIN ≥2 and cervical cancer is not clear, because many of these lesions in women younger than 30 years regress spontaneously. Because CIN ≥2 was used as a surrogate outcome in these studies, there is moderate CoE for reducing the risks of CIN ≥2, but low CoE for the outcome of cervical cancer.

#### Discussion

See discussion under No Recommendation F.

***Recommendation 22: In male patients with IBD aged 9–26 years, we suggest HPV vaccine be given.***

GRADE: Conditional recommendation, very low CoE. Vote on PICO question: yes, 100%

#### Key evidence

The CDC GRADE assessment of evidence for HPV vaccine in males included 1 RCT with the 4vHPV vaccine, and data showing comparable immunogenicity of 9vHPV in males and females (63). The review concluded that HPV vaccine was effective for the prevention of genital warts, and anal intraepithelial neoplasia.

The CoE for effectiveness was anchored to the general population and was downgraded from moderate to low for patients with IBD because the immunogenicity data included female patients with IBD only (162). The evidence for safety was downgraded from moderate to very low because there are no data in males with IBD.

#### Discussion

See discussion under No Recommendation F.

***No Recommendation F (see Appendix 3, 10C): In female and male patients with IBD aged 27–45 years, the consensus group could not make a recommendation for or against giving HPV vaccine.***

GRADE for PICO: low CoE for female patients and very low CoE for male patients. Vote on PICO question: yes, 22%; uncertain/neutral, 78%

#### Key evidence

The CDC GRADE assessment of evidence for the use of catch-up HPV vaccine in adults was based on data from RCTs of the 4vHPV vaccine in this age group, and data showing comparable immunogenicity of 9vHPV and 4vHPV (63). The review concluded that HPV vaccine was effective for the prevention of HPV infections, anogenital warts, and CIN ≥1.

The CoE was anchored to the general population and the rating started at moderate for female and low for male. It was not downgraded for females with IBD because of the immunogenicity study in females (162), but was downgraded for males with IBD. The evidence for safety was downgraded from moderate to low due to indirectness in females with IBD, and to very low for males with IBD.

#### Discussion

NACI recommends HPV vaccines be used routinely for male and female patients aged 9–26 years, and may be used in adults older than 26 years (12). CDC recommends routine HPV vaccination for male and female patients at ages 11–12 years (can be started at age 9 years), and catch-up vaccination through age 26 years (13). They do not recommend catch-up vaccination of adults aged 27–45 years, but suggest patient preferences be considered in adults at risk.

A systematic review of the cost-effectiveness of HPV vaccination programs included 29 studies of bivalent and 4vHPV vaccines (163). Routine vaccination of adolescent girls was consistently cost-effective compared with cervical screening alone. Including boys in a program was generally not cost-effective. However, the incremental cost per QALY gained by vaccinating adults through age 30 years exceeded $300,000 in 4 of 5 economic models in the United States, as reviewed by the CDC (13). In systematic reviews assessing acceptability of HPV vaccination, the recommendation of a health care professional was one of the most important factors in getting vaccinated (164, 165). Other factors included cost, concerns regarding sexual activity, and low perceived risks of HPV infection.

Based on the evidence for efficacy, safety, and cost-effectiveness, HPV vaccine is recommended for females and suggested for males aged 9–26 years with IBD. Due to insufficient evidence, the consensus group could not make a recommendation for or against HPV vaccine for adults aged 27–45 years with IBD. Patients who are immunosuppressed may have an increased risk of cervical dysplasia and cancer (156–160). In addition, NACI recommends HPV vaccine for adults who are immunocompromised (eg, use of immunosuppressive therapy, or underlying medical conditions) (12). In adults with IBD, current vaccine status, risks, and patient preferences should be considered.

## Summary

Previous guidelines on immunization in patients with IBD were developed through traditional expert consensus-based methodology (166, 167). This is the first guideline on immunization in patients with IBD that considers not only the certainty of evidence of vaccine safety and effectiveness in IBD populations, but also the ample evidence available in the general population and in other immune-mediated inflammatory diseases. The recommendations were developed using the rigorous GRADE methodology and the Evidence-to-Decision framework with consideration of all factors that are important for decision-making including the balance of benefits and harms, patient values and preferences, and resources (cost-effectiveness). As a result, the decision-making process was much more structured, systematic, and transparent than previous guidelines. The evidence profile tables and the Evidence-to-Decision framework (Appendix 3) that determine the direction and strength of a recommendation will enable decision-makers in different settings to adopt recommendations or decisions, or adapt them to their context. Appendix 4 summarizes the immunization recommendations of this guideline in comparison to the European Crohn’s and Colitis Organization, the American College of Gastroenterology, and the CDC (13, 166, 167).

This guideline should help optimize immunization strategies to reduce the risk of vaccine-preventable infections in patients with IBD. However, many questions remain unanswered. Further research is needed to assess whether accelerated vaccination schedule may be safe and effective in patients requiring urgent immunosuppressive therapy. Given that patients with IBD on immunosuppressive therapy may have lower immune response to vaccine, further research will be needed to assess the safety and effectiveness of high-dose vs standard-dose vaccination strategy. In addition, most studies used immunogenicity as a surrogate end point for vaccine efficacy in patients with IBD. Immunogenicity may be a valid end point to predict vaccine efficacy in the general population, but further research is needed to determine whether the results are generalizable to patients with IBD, particularly those on immunosuppressive therapy. More research is also needed to address the optimal timing of vaccination in relation to the dosing of biologics. Finally, there is a need for more studies to assess the safety and effectiveness of live and inactivated vaccines in patients with IBD on different types of immunosuppressive therapies.

These guidelines will be updated as appropriate when new evidence becomes available. As new vaccines are developed, such as the vaccines to SARS-CoV-2 (severe acute respiratory syndrome coronavirus 2), a similar process of evidence evaluation, consensus building, and agreement would be required to add them to future revisions to these guidelines. Unfortunately, at the moment, vaccines to SARS-CoV-2 have not been studied in the IBD population sufficiently to include recommendations in the current formal clinical practice guideline, although the Canadian Association of Gastroenterology recently released a communiqué recommending COVID-19 vaccines in IBD patients (168).

## Supplementary Material

Note: To access the supplementary material accompanying this article, visit the online version of *Gastroenterology* at www.gastrojournal.org, and at http://doi.org/10.1053/j.gastro.2021.04.034.

gwab016_suppl_Supplementary_Appendix_1

gwab016_suppl_Supplementary_Appendix_3

gwab016_suppl_Supplementary_Appendix_4

## Abbreviations

anti-HBs: hepatitis B surface antibody
anti-TNF: anti-tumor necrosis factor
aOR: adjusted odds ratio
CDC: Centers for Disease Control and Prevention
CIN: cervical intraepithelial neoplasia
CoE: certainty of evidence
CI: confidence interval
DTaP: diphtheria and tetanus toxoids, acellular pertussis
GRADE: Grading of Recommendation Assessment, Development and Evaluation
HBV: hepatitis B virus
Hib: *Haemophilus influenzae* type b
HPV: human papillomavirus virus
HZ: herpes zoster
IBD: inflammatory bowel disease
IMD: invasive meningococcal disease
IPD: invasive pneumococcal disease
LZV: live attenuated *Herpes zoster* vaccine
NACI: National Advisory Committee on Immunization
PCV13: pneumococcal conjugate 13-valent
PPSV23: pneumococcal polysaccharide 23-valent
QALY: quality-adjusted life-year
RCT: randomized controlled trial
RR: relative risk
RZV: recombinant *Herpes zoster* vaccine
4vHPV: quadrivalent human papillomavirus vaccine
9vHPV: 9-valent human papillomavirus vaccine
WHO: World Health Organization

## Canadian Association of Gastroenterology Statement

This clinical practice guideline (CPG) on immunizations in patients with IBD was developed under the direction of Dr Eric I Benchimol and Dr Jennifer L. Jones, in accordance with the policies and procedures of the Canadian Association of Gastroenterology (CAG) and under the direction of CAG Clinical Affairs. It has been reviewed by the CAG Clinical Affairs Committee and the CAG Board of Directors. The CPG was developed following a thorough consideration of medical literature and the best available evidence and clinical experience. It represents the consensus of a Canadian and US panel composed of experts on this topic. The CPG aims to provide a reasonable and practical approach to care for specialists and allied health professionals charged with the duty of providing optimal care to patients and families, and can be subject to change as scientific knowledge and technology advance and as practice patterns evolve. The CPG is not intended to be a substitute for physicians using their individual judgment in managing clinical care in consultation with the patient, with appropriate regard to all the individual circumstances of the patient, diagnostic and treatment options available, and available resources. Adherence to these recommendations will not necessarily produce successful outcomes in every case.
