# Supplementary material for: Canadian Association of Gastroenterology Clinical Practice Guideline for Immunizations in Patients With Inflammatory Bowel Disease (IBD)—Part 2: Inactivated Vaccines
Source: J Can Assoc Gastroenterol. 2021 Jul 29;4(4):e72–91. doi: 10.1093/jcag/gwab016 (PMC8407486; doi:10.1093/jcag/gwab016)
Supplement: gwab016_suppl_Supplementary_Appendix_1 [file gwab016_suppl_Supplementary_Appendix_1.pdf]

APPENDIX 1: DISCLOSURES AND MANAGEMENT OF CONFLICTS OF INTEREST

CAG policy guided disclosures and the management of conflicts of interest. The full methods are presented in detail in Supplementary Appendix 1. In accordance with CAG policy, the guideline co-chairs (EIB, JJ) and the GRADE methodologists (FT, MC) had no or minimal relevant COIs, and the majority (>50%) of the guideline panel were free of significant COIs.

The CAG adheres to the nine principles developed by the Guidelines International Network (G-I-N) for disclosing interests and managing conflicts of interests (COIs).[1] As per CAG policy, all individuals who were intellectually involved in this guideline (co-chairs, panel members, GRADE methodologists, moderators) disclosed all conflicts of interests (COI) by using a standardized declaration of interest (DOI) form. The CAG has defined current interests as those that have arisen during the 2 years preceding the invitation to participate on the guideline panel and during the guideline development process. The COI disclosures were then reviewed by the CAG Practice Affairs Lead prior to the individuals being accepted as panel members and also after the PICO questions were finalized. To ensure transparency and objectivity in this process, the Significance Scale (developed by the American Thoracic Society) was used as a guide in assessing the significance of COIs and in determining the level of management needed.[2] Based upon the assessment, panelists were considered as either having no or minimal relevant COIs, having significant COIs that requirement management, or having disqualifying COIs that must be terminated in order to serve as a panel member. CAG Practice Affairs discussed the results of the COI assessment and potential management strategies with co-chairs (EIB, JJ) and CAG Clinical Affairs. CAG Practice Affairs then prepared a COI grid for all panel members to inform eligibility to participate in discussion and/or voting for each PICO question. The COI grid was reviewed with all panel members at the face-to-face meeting, where everyone was given an opportunity to disclose any new and relevant COIs before the content discussion began. All disclosed COIs are included with this manuscript.

For this guideline, any declared interest that is related to vaccines (eg, research funding for vaccine-related work from a vaccine company; participation in industry-funded research, scientific advisory committees, consulting roles, speaking engagements, or expert testimony on matters related to vaccines) is considered a significant COI. Intellectual conflicts, such as a prior publication or scientific presentation on vaccines, were recognized as important and were

required to be disclosed, but because they were ubiquitous, intellectual conflicts were not counted as conflicted.

The guideline co-chairs (EIB, JJ) and the GRADE methodologists (FT, MC) had no or minimal relevant COIs, and the majority of the guideline panel had minimal COIs. A declared interest is considered minimal if it is unlikely to affect, or be reasonably perceived to affect, the expert's judgment. Individuals with minimal COIs were allowed to participate in the discussion and vote on all recommendations (except the GRADE methodologists [FT, MC] and the moderator [JKM] who were non-voting members of the panel). Two panel members (GM, SM) were considered to have significant COIs requiring management. Both panel members were permitted to participate in discussions about the evidence, but were recused from decision making, including formulating, voting on, grading, and writing recommendations for all statements. One panel member (OV) was considered to have significant COIs for PICO questions pertaining to pneumococcal and rotavirus vaccines, and was permitted to participate in discussions about the evidence, but was recused from voting on those statements.

1. Schunemann HJ, Al-Ansary LA, Forland F, et al. Guidelines International Network: Principles for disclosure of interests and management of conflicts in guidelines. Ann Intern Med 2015;163:548-53.
2. Schunemann HJ, Osborne M, Moss J, et al. An official American Thoracic Society Policy statement: managing conflict of interest in professional societies. Am J Respir Crit Care Med 2009;180:564-80.

COI for Committee Members

	EIB	FT	MCC	JD	SAM	APH	CS
**Perceived risk**	No	No	Low	Low	High*	No	Low
**Discussion**	OK	OK	OK	OK	OK	OK	OK
**Voting**	OK	NA	NA	OK	No	OK	OK
**Description of COI**	None	None	Biologic therapies	Biologic therapies, Merck	GSK, Merck, Pfizer, Sanofi - honoraria, research grants, contracts, clinical trials	None	Biologic therapies, Pfizer
**Potential vaccines with financial COI**	None	None	None	None	Dtap, Hep B, Hib, HPV, MENB, Meningococcal Conj, MMR, Varicella, Pneumococcal 13, Pneumococcal 23, Rotavirus, Zoster recombinant, Influenza	None	None

	LLB	TB	NC	GYM	OGV	JKM	JLJ
**Perceived risk**	Low	Low	No	High*	Low	No	Low
**Discussion**	OK	OK	OK	OK	OK	OK	OK
**Voting**	OK	OK	OK	OK	OK	NA	OK
**Description of COI**	Merck	Biologic therapies, Merck, Pfizer	None	Pfizer, GSK	Pfizer	NA	Biologic therapies, Pfizer
**Potential vaccines with financial COI**	None	None	None	Zoster (Shingrix), MENB, DTaP, Hib, Pneumococcal 13, Rotavirus, Hep B, Quadrivalent influenza	None	NA	None

* A researcher has received a research grant and/or honoraria from a non-profit sponsor that is related to exploring the efficacy of a medication (vaccine) that will be discussed by a CPG panel. The CPG panel may make recommendations for its use.

Eric I. Benchimol (EIB), Frances Tse (FT), Matthew C. Carroll (MCC), Jennifer deBruyn (JD), Shelly A. McNeil (SAM), Anne Pham-Huy (APH), Cynthia H-T. Seow (CS), Lisa L. Barrett (LLB), Talat Bessissow (TB), Nicholas Carman (NC), Gil Y. Melmed (GYM), Otto G. Vanderkooi (OGV), John K. Marshall (JKM), Jennifer L. Jones (JLJ)
