# Supplementary material for: Canadian Association of Gastroenterology Clinical Practice Guideline for Immunizations in Patients With Inflammatory Bowel Disease (IBD)—Part 2: Inactivated Vaccines
Source: J Can Assoc Gastroenterol. 2021 Jul 29;4(4):e72–91. doi: 10.1093/jcag/gwab016 (PMC8407486; doi:10.1093/jcag/gwab016)
Supplement: gwab016_suppl_Supplementary_Appendix_3 [file gwab016_suppl_Supplementary_Appendix_3.pdf]

GRADE assessment for
The Canadian Association of Gastroenterology Clinical Practice Guideline for Immunizations in Patients with Inflammatory Bowel Disease (IBD)

Prepared by: Frances Tse and Matthew W. Carroll

INTRODUCTION

Why is immunization in inflammatory bowel disease an important problem?

Immunosuppression increases the risk of people developing various vaccine-preventable infections (VPIs). Therefore, Centers for Disease Control and Prevention (CDC) recommend that immunosuppressed patients be brought up to date against (VPIs). However, the response to immunizations (effectiveness) in IBD patients is unclear. Immunosuppression from medical therapy may alter vaccine response and clinical protection from infection. As well, the effect of underlying immune dysregulation inherent to IBD may impair response to vaccine. Furthermore, response to immunization may vary depending on age, disease severity and activity, specific immunosuppressive drug or regimen administered, and other factors. From the safety point of view, there are concerns of potential adverse effects related to the administration of vaccines (e.g. exacerbation of underlying disease) due to a number of case reports suggesting an effect of vaccination on immune-mediated inflammatory disease onset or course.[1] Hence, a systematic review of the available evidence and assessment of the quality (certainty) of evidence of the benefits and harms of immunizations in IBD patients are needed to inform evidence-based recommendations.

This clinical practice guideline is limited to common vaccine preventable illnesses and vaccines and is inclusive of both adult and pediatric populations with inflammatory bowel disease (IBD). The recommendations are not meant to be extrapolated to special patient subgroups or special situations (e.g. very early onset IBD, travelers etc).

Definition of PICO questions to inform recommendations:

An essential part of the recommendation development process is defining the information that will influence the direction and strength of a recommendation. In the case of vaccines in inflammatory bowel disease (IBD), there are many important factors to consider including the burden of VPI, effectiveness and safety, cost and cost-effectiveness, as well as patient preferences and values. We used the PICO (patient/intervention/comparator/outcome) framework in defining the questions for this guideline. For each vaccine, we first divided the IBD patient population into adult and pediatric subgroups *a priori*. For certain vaccines, we further subdivided the patient populations into important subgroups depending on disease burden including age-specific mortality and morbidity and certainty of evidence as recommendations may differ across subgroups of patients at different baseline risks of VPI with different immunological response to vaccines, as well as varying certainty of evidence in the safety and effectiveness of vaccines.

Critical and important outcomes:

How well a vaccine works can be measured through different types of studies. *Efficacy* of a vaccine is measured in a randomized, placebo-controlled study (RCT). However, depending on the type of vaccine, the certainty of evidence, as well as the balance of benefits and harms supporting its use in the general population, it may be unethical to offer a placebo instead of vaccine in patients with IBD after a recommendation for vaccination of the general population has been issued. This is because withholding the vaccine from people recommended to receive it would place them at risk for infection and possibly serious complications including death. *Effectiveness* of a vaccine is measured as an epidemiological effect from observational studies. These studies are also uncommon in IBD because of the prevalence of rare outcomes (mortality, VPI) in patients with a chronic relatively rare condition, such as IBD. *Immunogenicity* refers to the ability of a vaccine to induce humoral and/or cell-mediated immune responses in a vaccinated individual. The efficacy of a vaccine may be *indirectly* predicted when the protecting level of antibodies is known from previous epidemiological studies in the general population. Hence, ***Immunogenicity* is a surrogate outcome for vaccine efficacy**. In general, the necessity to substitute a surrogate outcome for patient-important outcomes may lead to rating down the certainty of evidence because of indirectness.

Outcomes considered critical were the primary factors influencing a recommendation and were used to determine the overall certainty of evidence supporting a recommendation. Surrogate outcomes were considered when evidence about population-

important outcomes was lacking. When this is the case, the surrogates that were used to substitute for the population-important outcomes were specified. The critically important outcomes included mortality, VPI, and serious adverse events (SAEs). Immunogenicity was considered a surrogate outcome which may be important for decision making.

**Critical outcomes (for decision making):**
• Mortality<br>• Vaccine preventable illness (VPI)<br>• Serious adverse events (SAEs)
**Important outcomes:**
• Immunogenicity is a surrogate outcome for vaccine efficacy.

Direct vs. indirect evidence

A systematic literature search was conducted to collect direct evidence of vaccine safety and efficacy in patients with IBD. As well, a literature search for large case-control or cohort studies for prognostic evidence in determining the baseline risk estimates for vaccine-preventable illnesses (e.g. risk of herpes zoster infection in IBD populations) was conducted for each vaccine to inform decision-making. Determining the typical risk of a vaccine-preventable illness in IBD populations and how particular characteristics of IBD patients (e.g. age, immunosuppressants, disease severity) influence this risk would help guideline panel estimate the absolute effect of a vaccine. As an example, if the risk of having a vaccine-preventable illness is very low, the possible absolute benefits of vaccine will inevitably be low and serious adverse effects related to the vaccine, even if rare, will loom large in any decision. If instead, the risk of a vaccine-preventable illness is high and the consequences are severe, the impact of an effective vaccine may be large and patients may be ready to accept vaccine related adverse effects.

When there was paucity of direct evidence in IBD populations, a systematic search of the literature for vaccine safety and efficacy in other immune-mediated inflammatory diseases (e.g. rheumatoid arthritis, psoriasis, psoriatic arthritis, systematic lupus erythematosus) was conducted for indirect evidence. Although individuals with HIV, solid organ transplant and cancer may also receive treatment with biologics, immunosuppressants, and/or corticosteroids, we excluded these conditions in our systematic review for indirect evidence because the mechanisms of immunosuppression is distinct in each of these diseases and thus it is very likely that the background risks of vaccine preventable illnesses and response to vaccination in terms of efficacy and safety are very different than that of patients with IBD.

Much of the evidence for the efficacy and safety of vaccines was available from RCTs and observational studies conducted in the general population. CDC and the World Health Organization (WHO) vaccine recommendations are developed using the GRADE approach with systematic review of the evidence to generate evidence-based recommendations with consideration of the balance of benefits and harms, type or certainty of evidence, values and preferences of the people affected, and health economic analyses. When available, **the CDC or WHO GRADE evidence profile tables and analyses for the use of vaccines in the general population** were reviewed and incorporated into the GRADE assessment for this guideline. In addition, a systematic search for **high quality systematic reviews and meta-analysis assessing the efficacy and safety of each vaccine in the general population** (healthy children and adults) was conducted. **For each vaccine, the evidence for its safety and efficacy/effectiveness in the general population was used as an anchor.** In some cases, the certainty of evidence for effectiveness was downgraded for indirectness when there was evidence (RCTs, observational studies, immunogenicity studies) suggesting that the vaccines may be less immunogenic/effective in IBD populations. However, if there were studies done in IBD populations (even observational in nature with immunogenicity as outcomes) that supported the findings of effectiveness/efficacy in the general population, the evidence was not downgraded for indirectness. In most cases, the certainty of evidence for safety was downgraded because small sample sizes of IBD studies with short-term follow-up cannot detect rare adverse events. And if there was no study done in IBD populations (even observational in nature), studies that were done in the general population would serve as the evidentiary base and the evidence was not downgraded for indirectness. The rationale for not downgrading the certainty of evidence in this case is that there is no reason to suspect that IBD patients are at lower risks for developing vaccine-preventable illness than the general population. On the contrary, there is reason to suspect that IBD patients may be at similar or higher risks for developing vaccine-preventable illness due to underlying immune dysregulation or immunosuppressive medications. Therefore, unless there are concerns about safety or effectiveness in IBD populations (even observational in nature), the evidence was not downgraded for indirectness. Under these circumstances, there is no compelling reason to deviate from country-specific immunization guidelines for the general population with protocols based on local epidemiologic, programmatic, resource, policy, disease control objectives and strategies. However, we would encourage more studies to be done in IBD populations under research priorities.

Rating the certainty of Evidence (CoE)

Studies enter into the GRADE system at a particular level based initially on their study design. For PICO questions related to treatment or intervention, a body of randomized controlled trials (RCTs) begins as high certainty evidence and a body of observational evidence as low quality. In contrast, prognostic studies (observational studies that answer the question whether

certain characteristics of patients – prognostic factors, within a population, increase or decrease the risk of an event) start out as high certainty evidence (e.g. IBD as a prognostic factor for developing a VPI). Evidence about prognosis may also originate from single arms of RCTs, as these could be conceptualized as two single arm observational studies (one being the intervention group, and the other control group). When no comparison is made – that is, when rates measured in one or the other arm, rather than the comparison, is the matter of interest – the distinction between the two designs loses much of its relevance. In general, we have more confidence of estimates of prognosis from observational studies than from RCTs because eligibility criteria for RCTs usually include filters (e.g. age, comorbidity, severity of disease) that exclude patients who are relevant to the prognostic question of interest. Furthermore, eligible patients may decline to participate in RCTs, and their reasons for declining may be related to their prognosis.

The following tools were used to assess risk of bias based on study designs:
RCTs – Cochrane risk-of-bias tool
Non-randomized studies of interventions – ROBINS-I with adaptation for cohort, case-control, and before-and-after study designs
Prognosis Studies – Quality in Prognosis Study (QUIPS)

The following table outlines the criteria for down- and up-grading the certainty of evidence. Studies may be downgraded by 1 or 2 points if there are serious or very serious issues with risk of bias, inconsistency, indirectness, imprecision, and publication bias. Examples of indirectness may include surrogate outcomes (e.g. immunogenicity), indirect comparisons, and problems with generalizability to the population of interest. Certainty of evidence can be raised based on large effect, dose-response gradient, and antagonistic bias and confounding only if the certainty has not already been lowered based on the previous 5 domains (except indirectness).

Certainty of evidence	Certainty rating first assigned based on study design	Certainty is lowered if	Certainty is raised only if the certainty level is not already lowered
High	Randomized controlled trials (RCTs)<br><br>Prognostic studies	Risk of bias<br>-1 serious<br>-2 very serious<br><br>Inconsistency	Large effect:<br>+1 RR or OR > 2 (or < 0.5) in 2+ studies<br>+2 RR or OR > 5 (or < 0.2) in 2+ studies<br><br>Dose response gradient (population-based):

<table>
<tr><td></td><td>(observational evidence and single arms of RCTs)</td><td rowspan="4">-1 serious
-2 very serious

Indirectness
-1 serious
-2 very serious

Imprecision
-1 serious
-2 very serious

Publication bias
-1 likely
-2 very likely</td><td rowspan="4">+1 Evidence of decreased risk with increased vaccine coverage including evidence of reversal at population level (disease returns when vaccine coverage is decreased)
+2 Very strong evidence of decreased risk with increased coverage

All plausible confounding:
+1 would have reduced the effect
+1 would have suggested a spurious effect when results showed no effect</td></tr>
<tr><td>Moderate</td><td></td></tr>
<tr><td>Low</td><td>Observational studies</td></tr>
<tr><td>Very Low</td><td>Case reports or Case series</td></tr>
</table>

Footnotes:
-1 move down 1 grade
-2 move down 2 grades

**Certainty of evidence**	**Definitions**
High	We are very confident that the true effect lies close to that of the estimate of the effect.
Moderate	We are moderately confident in the effect estimate. The true effect is likely to be close to the estimate of the effect, but there is a possibility that it is substantially different.
Low	Our confidence in the effect estimate is limited. The true effect may be substantially different from the estimate of the effect.
Very low	We have very little confidence in the effect estimate. The true effect is likely to be substantially different from the estimate of the effect.

Moving from evidence to recommendations

When formulating recommendations, CAG uses the GRADE **Evidence to Decision (EtD) framework** which encompasses criteria that are important for decision making in a structured, explicit, and transparent way in the context of clinical recommendations: certainty of evidence (in relation to benefits and harms), values and preferences (in relation to outcomes), balance of benefits and harms, resource implications (cost-effectiveness), acceptability, and feasibility. As this clinical practice guideline focuses on an individual clinician-patient encounter, we did not consider the criteria of equity. Whenever available, research evidence from systematic reviews or single studies were used to inform judgments for all criteria. The source of the evidence summarized in the framework was referenced. If no evidence was found, this was noted. Based on the overall assessment across criteria, panel reached a conclusion about the direction of the recommendation (for or against the intervention) and the strength of the recommendation. The conclusion was reached by discussion followed by voting.

In GRADE, recommendations can be either strong or conditional/weak. Generally, strong recommendations are restricted to high or moderate certainty evidence. Low or very low certainty evidence almost invariably mandates a weak recommendation unless one of the five paradigmatic situations is encountered.

The implications of a strong recommendation are:

- For patients – most people in your situation would want the recommended course of action and only a small proportion would not; request discussion if the intervention is not offered
- For clinicians – most patients should receive the recommended course of action
- For policy makers – the recommendation can be adopted as a policy in most situations.

The implications of a weak recommendation are:

- For patients – most people in your situation would want the recommended course of action, but many would not
- For clinicians – you should recognize that different choices will be appropriate for different patients and that you must help each patient to arrive at a management decision consistent with his or her values and preferences
- For policy makers – policy maker will require substantial debate and involvement of many stakeholders.

PICO 1 – Review of patient's history of immunization and vaccine preventable illnesses

PICO 1	**In all patients with IBD, should a complete review of the patient's history of immunization and vaccine preventable illnesses be done at diagnosis and updated at regular intervals by IBD care provider?**
**Population**	All IBD patients (all ages)
**Intervention**	Complete review of the patient's history of immunization and vaccine preventable illnesses at diagnosis and update at regular intervals
**Comparator**	No complete review of the patient's history of immunization and vaccine preventable illnesses at diagnosis and update at regular intervals
**Outcome**	Mortality, VPI, SAEs, Immunogenicity

Statement 1: In all patients with IBD, a complete review of the patient's history of immunization and vaccine preventable illnesses should be done at diagnosis and updated at regular intervals by IBD care provider. (*Ungraded good practice statement)*

This PICO question can be considered a **good practice statement** based on the following 5 criteria:[1]

1. Is the statement clear and actionable? Yes.

2. Is the message really necessary in regard to actual health care practice? Yes.

   Vaccine utilization remains suboptimal in IBD patients.[2-5] Among IBD patients, a lack of awareness, perceived lack of benefit, concerns about adverse events, and insufficient counseling by providers are the most common reasons for non-immunization.[2-5] An internet-based cohort study of 958 IBD patients performed by the Crohn's and Colitis Foundation of American Partners Program showed that vaccinations and counselling rates (3.5-19.5% for various live vaccines) were exceedingly low in this setting.[6] Interesting, 59.5% individuals thought that patients should be responsible for keeping track of their vaccines, whereas 44.7% placed responsibility on their gastroenterologist and 62.1% on their primary care physician.[6] Only 44.9% recalled their gastroenterologists had previously taken a vaccination history.[6] Another survey revealed that 52% of gastroenterologists asked their IBD patients immunization history most or all of the time, and the majority believed that the primary care physicians should determine which vaccinations to give (64%) and administer the vaccine (83%).[7] Yet, a survey of primary care physicians noted that only 30% of them were comfortable with vaccination of IBD patients.[8] This

ambiguity as to the role of the gastroenterologists vs primary care physicians in assuming responsibility for immunizations in this particular population and the lack of knowledge of the proper vaccination schedules in IBD may be the main reasons underlying this relevant problem. Previous studies have demonstrated that provider recommendation is the strongest predictor for receipt of preventative health services including vaccination.[6,9,10] Therefore, gastroenterologists should take an active role in obtaining a vaccination history, providing recommendations to the primary care clinician for the appropriate vaccines to be administered, and assuring that their IBD patients are appropriately immunized.

3. After consideration of all relevant outcomes and potential downstream consequences, will implementing the good practice statement result in large net positive consequences? Yes.

   Patients with IBD are not considered to have systemic immunodeficiency at diagnosis, but subsequently may become immunocompromised due to immunosuppressive medications used to treat their underlying inflammatory condition. Relative immunosuppression may vary during the course of disease. Severe vaccine preventable illnesses have been reported in patients with IBD. Therefore, the ideal timing to review patient's immunization record and history of vaccine preventable illnesses is at the diagnosis of the disease and at regular intervals during follow-up because the course of IBD and its treatment may vary over time, and an immunocompromised status may reduce the efficacy and/or possibility to perform all necessary vaccinations. Therefore, implementing this good practice statement will result in more optimal use of vaccination which in turn may lead to a reduction in the risks of vaccine preventable illnesses.

4. Is collecting and summarizing the evidence a poor use of a guideline panel's limited time and energy (opportunity cost is large)? Yes.

   There have been no randomized trials or observational studies that have directly compared reviewing or not reviewing immunization status among IBD patients or among any populations. However, there is a large and compelling body of indirect evidence that strongly supports the net benefit of the recommended action. We could collect all the reports of how vaccinations reduce the risk of vaccine preventable illnesses. We could also collect the evidence that supports the usefulness of reviewing immunization record and history of vaccine preventable illness in assessing immunization status for each vaccine. We could link these bodies of evidence to make the case for high level of certainty regarding the net benefits of reviewing immunization status among IBD patients. The case for a good practice statement rather than a GRADEd recommendation is the poor use of time in collecting and summarizing the relevant evidence.

5. Is there a well-documented clear and explicit rationale connecting the indirect evidence? Yes.

   Patients with chronic, immune-mediated conditions such as IBD are often treated with immunosuppressive therapies, potentially increasing their risks of developing serious infections including vaccine-preventable illnesses. Vaccinations have been shown to reduce disease, disability, and death from a variety of infectious diseases.[11] Without a complete review of the patient's immunization record and history of vaccine preventable illnesses, optimal use of vaccination will not be possible.

References:

1. Guyatt GH, Schünemann HJ, Djulbegovic B, Akl EA. J Clin Epidemiol. 2015 May;68(5):597-600. Guideline panels should not GRADE good practice statements.
2. Melmed GY, Ippoliti AF, Papadakis KA, Tran TT, Birt JL, Lee SK, Frenck RW, Targan SR, Vasiliauskas EA. Patients with inflammatory bowel disease are at risk for vaccine-preventable illnesses. Am J Gastroenterol. 2006 Aug;101(8):1834-40
3. Khan N, Trivedi C, Kavani H, Lewis J, Yang YX. Frequency of Herpes Zoster Vaccination Among Inflammatory Bowel Disease Patients. Inflamm Bowel Dis. 2019 Jan 10;25(2):345-351.
4. Pham HV, Hasan I, Udaltsova N, Pham K, Abramson O, Armstrong MA, Postlethwaite D, Li D. Rates and Predictors of Vaccinations Among Inflammatory Bowel Disease Patients Receiving Anti-Tumor Necrosis Factor Agents. Dig Dis Sci. 2018 Jan;63(1):209-217.
5. Huth K, Benchimol EI, Aglipay M, Mack DR. Strategies to Improve Influenza Vaccination in Pediatric Inflammatory Bowel Disease Through Education and Access. Inflamm Bowel Dis. 2015 Aug;21(8):1761-8.
6. Wasan SK, Calderwood AH, Long MD, Kappelman MD, Sandler RS, Farraye FA. Immunization rates and vaccine beliefs among patients with inflammatory bowel disease: an opportunity for improvement. Inflamm Bowel Dis. 2014 Feb;20(2):246-50.
7. Wasan SK, Coukos JA, Farraye FA. Vaccinating the inflammatory bowel disease patient: deficiencies in gastroenterologists knowledge. Inflamm Bowel Dis. 2011 Dec;17(12):2536-40.
8. Selby L, Hoellein A, Wilson JF. Are primary care providers uncomfortable providing routine preventive care for inflammatory bowel disease patients? Dig Dis Sci. 2011;56:819–24.
9. Malhi G, Rumman A, Thanabalan R, Croitoru K, Silverberg MS, Hillary Steinhart A, Nguyen GC. Vaccination in inflammatory bowel disease patients: attitudes, knowledge, and uptake. J Crohns Colitis. 2015 Jun;9(6):439-44.
10. Loubet P, Verger P, Abitbol V, Peyrin-Biroulet L, Launay O. Pneumococcal and influenza vaccine uptake in adults with inflammatory bowel disease in France: Results from a web-based study. Dig Liver Dis. 2018 Jun;50(6):563-567.
11. Roush SW, Murphy TV; Vaccine-Preventable Disease Table Working Group. Historical comparisons of morbidity and mortality for vaccine-preventable diseases in the United States. JAMA. 2007 Nov 14;298(18):2155-63.

PICO 2 - Timing of vaccination

PICO 2	**In patients with IBD, should vaccination vs. no vaccination be given prior to the use of immunosuppressive therapy?**
**Population**	All IBD patients (all ages)
**Intervention**	Vaccination against VPI to which they lack immunity or have questionable immunity prior to the use of immunosuppressive therapy
**Comparator**	Vaccination against VPI to which they lack immunity or have questionable immunity after starting immunosuppressive therapy
**Outcome**	Mortality, VPI, SAEs, Immunogenicity
**Time**	Prior to (vs. after) starting immunosuppressive therapy

This PICO question actually contains **2 patient populations**. The first patient population includes patients with newly diagnosed moderate-severe IBD who require urgent immunosuppressive therapy and therefore cannot wait to optimize vaccination status prior to initiating immunosuppression. Delaying immunosuppressive therapy to optimize vaccination status in this setting will most likely lead to more harms than benefits. The second patient population includes patients with newly diagnosed mild-moderate IBD who in the judgement of the treating physicians do not require urgent immunosuppressive therapy. For this second patient population, the benefits of optimizing vaccination status prior to (vs. after) initiating immunosuppressive will most likely outweigh harms.

Statement 2: In patients with IBD, all appropriate vaccinations should be given as soon as possible, and ideally prior to initiation of immunosuppressive therapy. (*Ungraded good practice statement)*

Statement 3: In patients with IBD who require urgent immunosuppressive therapy, treatment should not be delayed in order to provide vaccinations. (*Ungraded good practice statement)*

Both PICO questions can be considered **good practice statements** based on the following 5 criteria:[1]

1. Are the statements clear and actionable? Yes.

2. Are the messages really necessary in regard to actual health care practice? Yes.

Vaccine utilization remains suboptimal in IBD patients.[2-5] Among IBD patients, a lack of awareness, perceived lack of benefit, concerns about adverse events, and insufficient counseling by providers are the most common reasons for non-immunization.[2-5] Previous studies have demonstrated that provider recommendation is the strongest predictor for receipt of preventative health services including vaccination.[6-8] Therefore, gastroenterologists play an important role in providing recommendations to the primary care clinician for the appropriate vaccines to be administered.

Patients with IBD are not considered to have systemic immunodeficiency at diagnosis, but subsequently may become immunocompromised due to immunosuppressive medications used to treat their underlying inflammatory condition. Relative immunosuppression may vary during the course of disease, and an immunocompromised status may reduce the efficacy and/or possibility to perform all necessary vaccinations. Therefore, the ideal timing to administer appropriate routine vaccinations should be prior to initiation of immunosuppressive therapy. However, in patients who require urgent immunosuppressive therapy due to moderate or severe disease, delaying therapy to provide vaccination will most certainly lead to harms.

3. After consideration of all relevant outcomes and potential downstream consequences, will implementing the good practice statements result in large net positive consequences? Yes.

   There are general principles that should be followed when providing immunization to immunocompetent persons who might be anticipating initiation of immunosuppressive treatments. In general, immunizations for vaccine preventable infections (VPI) should be provided at a time with maximum benefits and expected immunogenicity along with minimum adverse effects and harm. For a patient with IBD, this time is generally at diagnosis and prior to starting immunosuppressive therapy. Indeed, observational studies have shown that IBD patients on immunosuppressive therapy have a significantly lower serological response to routine vaccinations, and the greatest effect is seen among patients on anti-TNF and combination immunosuppressive therapy.[9] However, delaying treatment in patients with moderate or severe disease who require urgent immunosuppressive therapy will most certainly lead to harms. Therefore, implementing the good practice statements will result in optimizing timing of vaccination (relative to immunosuppression) which in turn may lead to improved immunogenicity and safety of vaccines, and thereby a reduction in the risks of VPI.

4. Is collecting and summarizing the evidence a poor use of a guideline panel's limited time and energy (opportunity cost is large)? Yes.

There have been no randomized trials that have directly compared administering vaccinations prior to vs. after initiating immunosuppressive therapy in IBD patients. There are observational studies comparing serological response in IBD patients who are on immunosuppressive therapies vs. those who are not on immunosuppressive therapies.[9] However, these studies often did not account for confounding factors such as disease severity, activity and extent, comorbidities, and nutritional factors. Nevertheless, **the alternative of delaying vaccination until immunosuppressive therapy has been started in patients not requiring urgent immunosuppressive therapy is absurd or illogical as serological response to vaccination is expected to be either the same or reduced (not improved) during immunosuppressive therapy. Also, the alternative of delaying treatment to provide vaccinations in patients who require urgent immunosuppressive therapy is absurd or illogical.** If we GRADE both PICO questions, the certainty of evidence would be very low due to the observational designs of these studies. The very low certainty evidence would warrant a conditional / weak recommendation. If the alternative action is illogical, making a weak/conditional recommendation for an obvious course of action is counterintuitive.

There is a large and compelling body of indirect evidence that strongly supports the net benefit of the recommended action. We could collect all the reports of how vaccinations reduce the risk of vaccine preventable illnesses. We also have evidence that the immunological response to vaccines is suboptimal during immunosuppression from many observational studies. We could link these bodies of evidence to make the case for high level of certainty regarding the net benefits of administering vaccinations prior to initiating immunosuppression among newly diagnosed IBD patients. The case for a good practice statement rather than a GRADEd recommendation is the poor use of time in collecting and summarizing the relevant evidence.

5. Is there a well-documented clear and explicit rationale connecting the indirect evidence? Yes.

   Patients with IBD are often treated with immunosuppressive therapies, potentially reducing their response to vaccines. In patients who do not require urgent immunosuppressive therapy, all appropriate routine vaccinations should be given prior to initiation of immunosuppressive therapy whenever possible to optimize immunogenicity and minimize adverse events or harms. In contrast, patients who require urgent immunosuppressive therapy, delaying treatment in order to provide vaccinations will most certainly lead to harms.

The answers to all questions should be yes to proceed with a good practice statement.

References

1. Guyatt GH, Schünemann HJ, Djulbegovic B, Akl EA. J Clin Epidemiol. 2015 May;68(5):597-600. Guideline panels should not GRADE good practice statements
2. Melmed GY, Ippoliti AF, Papadakis KA, Tran TT, Birt JL, Lee SK, Frenck RW, Targan SR, Vasiliauskas EA. Patients with inflammatory bowel disease are at risk for vaccine-preventable illnesses. Am J Gastroenterol. 2006 Aug;101(8):1834-40
3. Khan N, Trivedi C, Kavani H, Lewis J, Yang YX. Frequency of Herpes Zoster Vaccination Among Inflammatory Bowel Disease Patients. Inflamm Bowel Dis. 2019 Jan 10;25(2):345-351.
4. Pham HV, Hasan I, Udaltsova N, Pham K, Abramson O, Armstrong MA, Postlethwaite D, Li D. Rates and Predictors of Vaccinations Among Inflammatory Bowel Disease Patients Receiving Anti-Tumor Necrosis Factor Agents. Dig Dis Sci. 2018 Jan;63(1):209-217.
5. Huth K, Benchimol EI, Aglipay M, Mack DR. Strategies to Improve Influenza Vaccination in Pediatric Inflammatory Bowel Disease Through Education and Access. Inflamm Bowel Dis. 2015 Aug;21(8):1761-8.
6. Wasan SK, Calderwood AH, Long MD, Kappelman MD, Sandler RS, Farraye FA. Immunization rates and vaccine beliefs among patients with inflammatory bowel disease: an opportunity for improvement. Inflamm Bowel Dis. 2014 Feb;20(2):246-50.
7. Malhi G, Rumman A, Thanabalan R, Croitoru K, Silverberg MS, Hillary Steinhart A, Nguyen GC. Vaccination in inflammatory bowel disease patients: attitudes, knowledge, and uptake. J Crohns Colitis. 2015 Jun;9(6):439-44.
8. Loubet P, Verger P, Abitbol V, Peyrin-Biroulet L, Launay O. Pneumococcal and influenza vaccine uptake in adults with inflammatory bowel disease in France: Results from a web-based study. Dig Liver Dis. 2018 Jun;50(6):563-567.
9. Nguyen DL, Nguyen ET, Bechtold ML. Effect of Immunosuppressive Therapies for the Treatment of Inflammatory Bowel Disease on Response to Routine Vaccinations: A Meta-Analysis. Dig Dis Sci. 2015 Aug;60(8):2446-53.

Risk of Bias Table

SR of Prognostic studies							
**Study**	**Study sample adequately represents the population of interest**	**Study data available adequately represent the study sample (>80% follow-up)**	**Prognostic factor measured in a similar and valid way for all participants**	**Outcome of interest is measured in a similar and valid way for all participants**	**Important potential confounding factors are appropriately accounted for**	**Statistical analysis is appropriate, and all primary outcomes are reported**	**Comments**
Nguyen 2015	OK	OK	Unclear. Mostly based on chart review of medication	Definition of seroresponse or seroprotection is variable among studies	Potential confounding factors (e.g. disease severity / extent,	Inappropriate to pool these studies together in a MA given the heterogeneity	• SR of 9 cohort studies (n = 1474) comparing IBD patients on IS (anti-TNF, IM,

			use (dose and duration were either not reported or reported as for at least certain duration)	even for the same vaccine	nutritional status, dose of medications etc) were not accounted for.	in study designs, control groups used (HC vs. IBD patients not on IS), interventions (different vaccines), and different definitions of outcomes (seroresponse or seroprotection for the same vaccine and also among different vaccines)	and/or prednisone ≥ 20mg/day) vs IBD patients not on IS or HC<br>• Different vaccines were included: Hep B (2), Hep A (1), Influenza (2), Pneumococcal (4)<br>• **IBD patients on IS have a lower chance of achieving adequate seroprotection vs. HC or IBD patients not on IS (OR 0.41, 95% CI 0.30-0.55)**

HC: Healthy controls
IM: immunomodulators
IS: immunosuppressive therapies

LIVE VACCINES

Measles, Mumps, Rubella

Background

Measles, mumps and rubella (MMR) are serious viral diseases that can result in significant morbidity and even mortality. All 3 diseases are vaccine preventable.

Measles is a highly contagious paramyxovirus with only one antigenic type. The primary site of infection is the respiratory epithelium with a primary viremia two to three days later in the reticuloendothelial system. A second viremia occurs 5-7 days later with spread to other organs. The typical clinical presentation is of coryza, cutaneous maculopapular rash and often oral mucosal rash (Koplik spots). Approximately 30% of infections develop complications, with increased frequency in children less than 5 years and adults older than 20 years. Diarrhea, otitis media and pneumonia are common. Acute encephalitis occurs in approximately 0.1% of reported cases and death in 0.2%. Pneumonia accounts for about 60% of deaths. Measles in an immunocompromised patient may be severe with a prolonged course.[1,2] As a result of high 2-dose vaccine coverage, Canada achieved elimination of endemic measles transmission in 1998. However, transported cases from other endemic regions still occurs and secondary spread to at risk individuals causes sporadic outbreaks. At risk individuals include the unimmunized and those who have only received one dose of vaccine.[3]

Mumps virus is a paramyxovirus in the same group as parainfluenza. The virus is acquired via respiratory droplets and replicates in the nasopharynx and regional lymph nodes. An acute viremia occurs 12-25 days later with spread to tissues including the meninges, salivary glands, pancreas, testes and ovaries. Parotitis is the most common manifestation. Orchitis is the most common complication in post-pubertal males with reported rates of 12-66% in pre-vaccination era. Prior to vaccination, mumps was one of the most common causes of aseptic meningitis and sensorineural deafness in childhood.[1,2]

Rubella is classified as a togavirus and has one antigenic type. Following respiratory transmission, replication occurs in the nasopharynx and regional lymph nodes. A viremia occurs 5-7 days later with spread of the virus throughout the body. Acquired rubella (as opposed to congenitally acquired disease) typically manifests as a maculopapular rash. In older children and adults, fever, malaise and lymphadenopathy are common as are arthritis and arthralgia in adults. Complications are not common, but encephalitis is reported one in 6000 cases. Hemorrhagic manifestations are estimated to occur 1 per 3000 cases. Congenitally acquired infection may affect all organs and is associated with a variety of congenital defects. Transplacental fetal infection is most severe in near gestation and may lead to death, spontaneous abortion and preterm delivery.[1,2]

Both NACI and the CDC recommend routine childhood vaccination against measles, mumps and rubella (MMR) with a first dose at 12-15 months of age and a second dose at 18 months of age or anytime thereafter, but should be given no later than around school entry (4-6 years).[4,5] This is also the recommendation for varicella. Catch-up vaccination is recommended for children and adolescents aged 12 months to less than 13 years who are previously unimmunized.[4,5] For susceptible adults born in or after 1970 (susceptible

health care workers, military personnel, susceptible travelers to destinations outside of North America, susceptible students in post-secondary educational settings), 1-2 doses or MMR vaccine is also recommended.[4,5] Adults born before 1970 in Canada or 1957 in the US are generally presumed to have acquired natural immunity to measles; however, some of these individuals may be susceptible.[4,5]

In Canada, there exists both live attenuated trivalent MMR vaccines including M-M-R-®II and Priorix® and a combined quadrivalent MMR-varicella (MMRV) vaccines including Priorix-Tetra™ and ProQuad™. In the US, M-M-R-®II and ProQuad™ are approved for use. Monovalent vaccines against measles, mumps and rubella are no longer commercially available. As per NACI guidelines, MMRV may be used in place of individual MMR plus varicella vaccines.[5] In 12 month old children, a single dose of MMRV vaccine results in similar seroconversion rates as those achieved after concomitant administration of MMR vaccine and univalent varicella vaccine.[2] A study of children receiving 2 doses of MMRV vaccine during the second year of life noted seropositivity for measles, mumps, rubella and varicella of 99%, 97.4%, 100% and 99.4% respectively by the third year post-vaccination.[2] The efficacy of a single dose of MMR vaccine is 93% effective against measles, 78% effective against mumps, and 97% effective against rubella.[1] Two doses of MMR vaccine are 97% effective against measles and 88% effective against mumps.[1] In the US, national immunization programs with MMR led to elimination of measles in 2000, rubella in 2004, and a 96% reduction in mumps.[6] The live MMR vaccine is believed to confer lifelong immunity to measles and rubella. In contrast, vaccination against mumps does not lead to sustained protection. The ACIP has recently recommended administration of a third dose of the MMR vaccine for at-risk groups during mumps outbreaks. There are no data regarding the long-term effectiveness of MMRV vaccine which is not recommended for individuals older than 13.

As per CDC ACIP and NACI, MMR vaccines are contraindicated in persons with impaired immune function and pregnancy because of the theoretical risk to the fetus.

Serologic testing is not routinely recommended before vaccination if a person has other acceptable evidence of immunity to these diseases. Similarly, post vaccination serologic testing to verify an immune response is not recommended. The ACIP emphasizes that MMR serologies may be falsely negative in previously vaccinated persons and considers the immunization record of MMR to be a reliable surrogate of immunity. However, serologic testing may be used for situations where there is no record of prior immunization or infection. ACIP recommends against giving additional doses of MMR vaccine for individuals with negative serologies and proof of age-appropriate immunization.

References:

1. Centers for Disease Control and Prevention. Epidemiology and Prevention of Vaccine-Preventable Diseases. Hamborsky J, Kroger A, Wolfe S, eds. 13th ed. Washington D.C. Public Health Foundation, 2015.

2. Public Health Agency of Canada Accessed on line Sept 27 2019 at https://www.canada.ca/en/public-health/services/publications/healthy-living/canadian-immunization-guide-part-4-active-vaccines.html
3. McLean HQ, Fiebelkorn AP, Temte JL et al. Prevention of Measles, Rubella, Congenital Rubella Syndrome, and Mumps, 2013: Summary Recommendations of the Advisory Committee on Immunization Practices (ACIP). Morbid and Mortal Weekly Report. 2013; 62(RR04):1-34.
4. Public Health Agency of Canada. Accessed on line Sept 27 2019 at https://www.canada.ca/en/public-health/services/diseases/measles/health-professionals-measles.html#s9
5. Public Health Agency of Canada. An Advisory Committee Statement (ACS) National Advisory Committee on Immunization (NACI) Statement on Measles-Mumps-Rubella-Varicella Vaccine. 2010. Accessed online Sept 27 2019 at https://www.canada.ca/en/public-health/services/reports-publications/canada-communicable-disease-report-ccdr/monthly-issue/2010-36/canada-communicable-disease-report-14.html
6. Roush SW, Murphy TV; Vaccine-Preventable Disease Table Working Group. Historical comparisons of morbidity and mortality for vaccine-preventable diseases in the United States. JAMA. 2007 Nov 14;298(18):2155-63.

Risk of infection with Measles, Mumps, Rubella in IBD patients

PICO: What is the risk of measles, mumps and rubella in people with IBD compared to people without IBD?

Summary – Adult and Pediatric

The literature search did not identify any study on the risk of measles, mumps and rubella in adult or pediatric IBD patients.

Effectiveness and Safety of MMR Vaccine in IBD Patients

Summary – Pediatric

**PICO 4A**	**In MMR-susceptible pediatric patients with IBD not on immunosuppressive therapy, should vaccination vs. no vaccination against mumps, measles, rubella (MMR) be given?**
**Population**	**MMR-susceptible pediatric patients with IBD not on immunosuppressive therapy**
**Intervention**	Vaccination against MMR

**Comparator**	No vaccination against MMR
**Outcome**	Mortality, VPI (MMR infection), SAEs, Immunogenicity

**PICO 4B**	**In MMR-susceptible pediatric patients with IBD on immunosuppressive therapy, should vaccination vs. no vaccination against mumps, measles, rubella (MMR) be given?**
**Population**	**MMR-susceptible pediatric patients with IBD on immunosuppressive therapy**
**Intervention**	Vaccination against MMR
**Comparator**	No vaccination against MMR
**Outcome**	Mortality, VPI (MMR infection), SAEs, Immunogenicity

There was no RCT comparing MMR vaccines with placebo in patients with IBD to address this PICO question.

One Cochrane systematic review assessed the evidence on the effectiveness of MMR vaccines in healthy children and reported one MMR vaccine dose to be at least 95% effective in preventing clinical measles and 92% effective in preventing secondary cases among household contacts.[1] Effectiveness of at least 1 dose of MMR in preventing clinical mumps in children is estimated to be between 69-81% depending on the vaccine strain.[1] No studies were identified for effectiveness of rubella[1]. The World Health Organization (WHO) assessed the evidence on the effectiveness of measles and rubella vaccines in healthy children in 2017 and 2011, respectively. In the WHO evidence profile, they also included 2 RCTs on clinical efficacy in measles and rubella.[2,3] Overall, the WHO rated the quality of evidence as **high** for MMR vaccination in immunocompetent healthy children in preventing measles, mumps and rubella.

One systematic review included 40 observational studies (mostly cohort studies and case series/reports) in patients with immune-mediated diseases (2852/20,556 were IBD patients) on 22 different immunosuppressive medications.[4] The immunosuppressive medications used by IMID patients included prednisone 2.5-35mg/day, methotrexate 5-27mg/week, 6MP, biologic monotherapy, and combination therapy with biologic and immunomodulator.[4] The results are somewhat inconsistent in that some studies showed reduced antibody titer when vaccinated while on immunosuppressive medications, while other studies showed no significant differences in humoral responses between patients with and without methotrexate or biologics after MMR vaccination.[4] Four cross-sectional studies (1 in pediatric patients, and 3 in adult IBD patients who received MMR vaccines in their childhood) assessed the serologic status of MMR in IBD patients with the majority of patients on immunosuppressive medications.[5-8] Among pediatric IBD

patients who received the complete 2-dose MMR series, serologic protection was present for 67.6% for measles, 63.3% for mumps, and 81.4% for rubella.[6] In a cross-sectional study of adults with confirmed vaccination records of 2- dose MMR prior to IBD diagnosis (as children), there was no difference in pre-set antibody threshold for presumptive immunity for measles (87% vs. 75%) and rubella (74% vs. 85%), and no difference in antibody concentration for measles, mumps and rubella between IBD patients vs. healthy controls or among the IBD treatment groups (AZA/6MP, anti-TNF monotherapy, combination treatment).[5] It is important to note that these cross-sectional studies used different cut-off values for antibody titer for immunity or seroprotection, and cannot distinguish between waning titers over time vs. primary vaccination failure. There is no definitive serologic correlate of specific antibody levels with clinical protection against each virus (although the USA Food and Drug Administration accepted IgG antibody concentration ≥ 200 mIU/mL for anti-measles, ≥ 10 EU/mL for anti-mumps, and ≥ 10 IU/mL for anti-rubella as offering "clinical benefit"). As well, there has been no clear definition with supporting evidence that low titers of antibody cause more infection among vaccinated populations. In fact, the ACIP emphasizes that MMR serologies may be falsely negative in previously vaccinated persons and considers the immunization record of MMR to be a reliable surrogate of immunity. However, serologic testing may be used for situations where there is no record of prior immunization or infection. ACIP recommends against giving additional doses of MMR vaccine for individuals with negative serologies and proof of age-appropriate immunization. The GRADE rating started as low due to observational nature of these studies. The evidence was further downgraded to **very low** due to study limitations (residual confounding and selection bias) and indirectness (surrogate outcomes).

The certainty of evidence for effectiveness was anchored to the general population (healthy children), and started as high. When the evidence was applied to IBD patients, the evidence was downgraded to **moderate** due to indirectness as observational studies suggested that MMR vaccines may be less immunogenic in IBD patients. In summary, there is **moderate** certainty evidence that MMR vaccines are effective in reducing the risks of MMR in pediatric IBD patients.

The Cochrane systematic review also assessed the safety of MMR vaccines in healthy children.[1] MMR was associated with a lower incidence of upper respiratory tract infections, a higher incidence of irritability, and a similar incidence of other adverse events compared to placebo.[1] MMR vaccine was likely to be associated with aseptic meningitis (mumps) using Urabe strain-containing MMR, parotitis, joint and limb complaints, febrile seizures and benign thrombocytopenic purpura within 2 weeks of vaccination.[1] Exposure to MMR was unlikely to be associated with Crohn's disease, ulcerative colitis, autism or aseptic meningitis (mumps) using Jeryl-Lynn strain-containing MMR.[1] The WHO also assessed the evidence on the safety of measles and rubella vaccines in healthy children incorporating the findings of the Cochrane systematic review. The certainty of evidence was rated as **moderate** due to study limitations (inadequate reporting of adverse events).

One systematic review included 40 observational studies (mostly cohort studies and case series/reports) in patients with immune-mediated diseases (2852/20,556 were IBD patients) on 22 different immunosuppressive medications.[4] The administration of live vaccines was safe in most studies of IMID patients on immunosuppressive medications (including prednisone 2.5-35mg/day, methotrexate 5-27mg/week, 6MP, biologic monotherapy, and combination therapy with biologic and immunomodulator). Serious adverse events were rare (0.05%, 11/20,556 IMID patients). Infections through the vaccine strain were also rare, occurring in 0.06% (12/20,556 IMID patients) for all live vaccines, and 0.2% IMID patients through MMR vaccine. In most cases, infection was mild. However, two patients had fatal infection: a patient with RA/SLE overlap who started methotrexate/dexamethasone treatment 4 days after the yellow fever vaccine developed vaccine-associated viscerotropic disease and died. One infant whose mother was under infliximab treatment during pregnancy received the BCG vaccine at the age of 3 months and developed disseminated BCG infection and died. The certainty of evidence started as low due to the observational designs of these studies. The evidence was downgraded to **very low** due to study limitations and indirectness (patient population).

The certainty of evidence for safety was anchored to the general population (healthy children), and started as **moderate**. When the evidence is applied to **pediatric IBD patients not on immunosuppressive medications**, the evidence was not downgraded for indirectness. However, the evidence was downgraded to **very low** due to serious indirectness when applied to **pediatric IBD patients on immunosuppressive medications**. Although the risk of vaccine-induced infection with MMR seemed to be very rare in patients with immune-mediated diseases (including IBD), fatal outcomes did occur following the administration of other live vaccines. As well, the small number of IBD patients on immunosuppressive medications included in the systematic review may not be large enough to detect rare adverse events.

Overall, there is moderate certainty evidence that MMR is safe and effective in pediatric IBD patients not on immunosuppressive medications. There is very low certainty evidence that MMR is safe and effective in pediatric IBD patients on immunosuppressive medications.

Risk of Bias Table – Pediatric and Adults

SR of RCTs (Observational Data) and Observational Studies		
**Study**	**Quality Assessment**	**Comments**

Croce (2017) IMID patients including children and adults	• Risk of bias was judged to be high in the majority of studies	• SR of 64 articles (8 RCTs, 39 observational studies and 17 case series / reports) on live vaccinations in patients with IMIDs or SOT on immunosuppressive therapy as well as BMT patients who received a live vaccine < 2 years after BMT.<br>• **40 studies were conducted in IMID patients (n = 20,556 with 2852 IBD) on 22 different IS medications**<br>• Vaccines in IMID: YF (n = 8 adults, n = 1 children and adults), **MMR (n = 1 adults, n = 5 children),** varicella (n = 3 adults, n = 9 children, n = 1 children and adults)**,** HZ (n = 8 adults), polio (n = 1 adult), BCG (n = 2 adults, n = 2 children), live typhoid (n = 1 adult), smallpox (n = 1 adult)<br>• **IS medications used by IMID patients: prednisone 2.5-35mg/day, MTX 5-27mg/week, biological monotherapy, biological + IM**<br>• **In most studies, the administration of live vaccines was safe.**<br>• SAEs were reported in 11 IMID patients under steroid treatment who received HZV (as frequent as the placebo group). No SAEs with MMR, polio, small pox, live typhoid, BCG.<br>• **12/20,556 IMID (0.06%) patients developed an infection through the vaccine strain**. **0.2% IMID patients (1/474) developed infection through MMR vaccine strain.** One juvenile idiopathic arthritis patient with MTX had fever and rash 20 days after vaccination. In most cases, the infection was mild. However, 2 patients had fatal infection: RA/SLE on MTX/dexamethasone received YF. Infant whose mother was on IFX during treatment received BCG.<br>• **No increase flares of autoimmune disease** in most studies<br>• High seroconversion rates in IMID group with VV and YFV. **MTX and anti-TNF appeared to reduce immune response to VV and HZ, but not to MMR and YF revaccination.**<br>• Although live vaccines were safe and sufficiently immunogenic in most studies, some serious reactions and vaccine-related infections were reported in immunosuppressed IMID patients.<br>• Until further data are available, live vaccinations under most immunosuppressive treatments should only be administered after a careful risk benefit assessment
Demicheli 2016 (Healthy children up to 15 years of age)	• Risk of bias assessment provided for included studies and overall risk of bias high due to study limitations, missing data, high risk for selection bias, detection, reporting and attrition bias.<br>• Design and reporting of safety outcomes in MMR vaccine studies, both pre- and post-marketing, are largely inadequate.	• Cochrane SR of 5 RCTs, 1 controlled clinical trial, 27 cohort studies, 17 case-control studies, 5 time-series, 1 case cross-over trial, 2 ecological studies, 6 self-controlled case series (14,700,000 healthy children)<br>• Descriptive review. No meta-analysis due to diversity of exposure, outcomes, and length of follow-up.<br>• **1 MMR vaccine dose is at least 95% effective in preventing clinical measles and 92% effective in preventing secondary cases among household contacts**<br>• **At least 1 dose of MMR in preventing clinical mumps in children is estimated to be 69-81% for the vaccine prepared with Jeryl Lynn mumps strain and between 70-75% for**

		**the vaccine containing the Urabe strain**.<br>• **No studies on effectiveness of MMR in preventing rubella.**<br>• MMR vaccine was associated with aseptic meningitis, febrile seizure and thrombocytopenic purpura.<br>• Exposure to MMR vaccine was unlikely to be associated with autism, asthma, leukemia, hay fever, type 1 diabetes, gait disturbance, Crohn's disease, demyelinating diseases, bacterial or viral infections.

**Cohort studies**							
**Study**	**Valid methods to ascertain exposure**	**Prognostic factors (other than exposure of interest) similar among cohorts – or cohorts were adjusted adequately for confounders**	**Demonstration that outcome of interest was not present at the start of the study**	**Outcome detection methods valid and similar among cohorts**	**Follow-up complete and similar among cohorts**	**Free of other bias**	**Comments**
Caldera 2019 (US) IBD patients - receipt of MMR as pediatric patients, titers were measured as adults	Documentation of a two-dose series of MMR vaccine in the Wisconsin Immunization Registry, an internet immunization information system tracking immunization dates of Wisconsin residents.	Not adjusted for disease activity / severity, nutritional factors, and other confounding factors.	No<br><br>Uncertain titer prior to vaccination	No certain serologic correlate of protection is accepted for mumps.<br><br>For measles, microneutralization titers > 120 mIU/ML give protection against disease.<br><br>For rubella, immunoprecipitation titer > 10-15 mIU/mL has been proposed	OK	Possible selection bias. Patients attending a tertiary referral center may differ systematically from other patients. Patients who agreed to participate in a study where serologic assays were measured	• Cross-sectional study comparing antibody concentrations following 2-dose MMR **(sustained antibody response**) among 46 IBD patients (received 2 MMR prior to diagnosis) vs 20 healthy controls<br>• IBD groups stratified by IS therapy (AZA/6MP, anti-TNF monotherapy, combination)

				as a useful correlate.		may be different than patients who did not agree to participate.	• Age and time of receipt of MMR similar in both groups<br>• Median age when titers were measured: 26<br>• Antibody threshold for protection: measles at 255mIU/ML; Rubella > 12 mIU/mL; No specific mumps antibody<br>• **No differences in antibody concentration of measles, mumps or rubella** between IBD patients vs. healthy controls or among the IBD treatment groups<br>• **No differences in pre-set antibody threshold for presumptive immunity to measles (80% vs. 75%) and rubella (74% vs 85%)**
deBruyn 2018 (Canada) Pediatric IBD	Vaccination records and baseline serology were used to determine immunity against vaccine preventable diseases including	IBD subtype, current immunosuppressive medication use, age at diagnosis, and age at serum collection were adjusted for in a multivariate analysis.<br><br>Disease activity at time	OK	Serologic protection was defined for qualitative assays as positive detection of measles IgG, mumps IgG. For quantitative assays, serologic protection was defined by rubella	OK	Possible selection bias. Patients attending a tertiary referral center may differ systematically from other	• Cross sectional study in children examining the serologic status of childhood vaccinatable diseases<br>• 156 children with IBD at a Canadian tertiary referral IBD unit.<br>• **Among 143 subjects**

	Varicella.	of vaccination, duration of disease and nutritional status were not accounted for.		IgG titer $\geq$ 15 IU/mL		patients. Patients who agreed to participate in a study where serologic assays were measured may be different than patients who did not agree to participate.	**who received the complete 2-dose MMR series, serologic protection was present for 67.6% for measles, 63.3% for mumps, and 81.4% for rubella.**<br>• Current IS therapy and age at enrollment were not associated with serologic protection.<br>• IBD type of UC and age at diagnosis were associated with increased odds of serologic protection for rubella (OR 3.15, 95% CI 1.03-8.63; OR 1.18, 95% CI 1.02-1.36), but not for measles or mumps
Abu-Elyazeed 2018 (US, Estonia, Slovakia) Healthy adults	OK	OK	OK	OK	OK	OK	• Observational data from both arms of a RCT MMR-RIT vaccine (Priorix) vs. MMRII in > 7 years old who had received $\geq$ 1 previous dose of MMR vaccine (n = 869)<br>• Most of the participants (64.1%) adults aged $\geq$ 18 (mean age 25.7)

							• Defined seroresponse as anti-measles IgG $\geq$ 200 mIU/mL, $\geq$ 10 IU/mL for anti-rubella, $\geq$ 10 EU/ML for anti-mumps<br>• Seroresponse rates of 98.8% for measles, 98.4% mumps, and 99.5% rubella after MMR-RIT. Non-inferior to MMRII<br>• No serious adverse events
Cleveland 2016 (US) IBD patients - receipt of MMR as pediatric patients, titers were measured as adults	"Recall of vaccination history was not predictive of immune status"<br><br>No immunization record	Disease activity at time of vaccination, duration of disease and nutritional status were not accounted for.	No	Antibody index - not an accepted correlate of protection	OK	Possible selection bias. Patients attending a tertiary referral center may differ systematically from other patients. Patients who agreed to participate in a study where serologic assays were measured may be different than patients who did not agree to participate.	• Cross-sectional study of 122 patients with IBD<br>• 47% on IM, 43% on anti-TNF, 15% anti-integrin<br>• 70% patients reported childhood vaccinations to measles<br>• Measles antibodies $\leq$ 0.8 antibody index = negative immunity, 0.9-1.1 = equivocal, $\geq$ 1.2 AI = positive immunity<br>• **13.1% lacked detectable immunity to measles, 3% had equivocal immunity, 83.6% immune**<br>• IS was not associated with "immunity" (75%

							of non-immune patients on IS vs. 64% of immune patients (OR 1.7, 95% CI 0.5-5.9)<br>• **Disease ≥ 10 years and age ≥ 50 were associated with significant lower titers**
Naganuma 2013 (Japan) IBD patients - receipt of MMR / infection as pediatric patients, titers were measured as adults	No immunization records.<br><br>Relied on patients' recall for history of vaccination / infection.	Did not adjust for any confounding factors	No<br><br>Uncertain titer prior to vaccination	No certain serologic correlate of protection is accepted for mumps.<br><br>For measles, microneutralization titers > 120 mIU/ML give protection against disease.<br><br>For rubella, immunoprecipitation titer > 10-15 mIU/mL has been proposed as a useful correlate.	OK	Possible selection bias. Patients attending a tertiary referral center may differ systematically from other patients. Patients who agreed to participate in a study where serologic assays were measured may be different than patients who did not agree to participate.	• Cross-sectional study of 139 IBD outpatients<br>• ELISA were used as serological tests for measles. Anti-Rubella < 10 IU/mL, anti-measles IgG < 16 IU/mL, and anti-mumps/varicella zoster IgG < 4 IU/mL as seronegative<br>• **34%, 37%, 30% were seronegative for measles, mumps, rubella**<br>• About 40% of IBD patients did not remember whether they had been previously infected<br>• 1/3 did not remember whether they had been vaccinated<br>• Proportions of patients with seropositive

							antibodies were significantly higher in patients who had a history of vaccination than in those who had no history or an unknown history of vaccination.<br>• 54% treated with IS had seronegative antibodies specific for at least one of the viruses.

6MP – 6 mercaptopurine
AZA - azathioprine
IS – immunosuppressive therapy

Evidence Profile Table – Pediatric

**Certainty Assessment**								**Summary of Findings**	
Studies	Risk of bias	Inconsistency	Indirectness	Imprecision	Other considerations	Certainty of Evidence	Overall Certainty of evidence	Study Event Rates	Relative Effect (95% CI)
**VPI (clinical and/or confirmed cases of MMR) - CRITICAL**							⊕⊕⊕⊖<br>MODERATE		
1 systematic review[1] of 5 RCTs, 1 controlled clinical trial, 27 cohort studies, 17 case-control studies, 5 time-series trials, 1 case cross-over trial, 2 ecological studies, 6 self-controlled case series studies	Not serious[a]	Not serious	Serious[b]	Not Serious	None	⊕⊕⊕⊖<br>MODERATE	For pediatric IBD patients not on IS<br><br>⊕⊖⊖⊖	• One MMR vaccine dose is at least 95% effective in preventing clinical **measles** and 92% effective in preventing secondary cases among household contacts.[1,3]<br>• One dose of MMR in preventing clinical **mumps** in children is estimated to be between 69-81% for the vaccine prepared with Jeryl Lynn mumps strain and between 70-75% for the vaccine containing the Urabe strain.[3]<br>• 1 RCT on the protective efficacy of rubella vaccine against clinical rubella (198 children in China) during a rubella outbreak. Protection against **rubella** was achieved in > 95%	

2 RCT[2,3]<br><br>**Healthy Children**<br><br>*Adapted from WHO Evidence Profile Table*							VERY LOW<br><br>For pediatric IBD patients on IS	of those who received the vaccine.[2]
**Immunogenicity (MMR antibody titer) - IMPORTANT**								
1 SR of 40 Observational Studies[4]<br><br>**20,556 IMID patients with 2852 IBD patients**	Serious[c]	Serious[d]	Serious[e]	Not serious	None	⊕⊖⊖⊖<br>VERY LOW		• Some studies showed reduced antibody titer when vaccinated while on IS. Other studies showed no significant differences in humoral responses between patients with and without MTX or biologics after MMR vaccination.
4 Observational studies[5-8]<br><br>**1 Pediatric IBD population**<br><br>**3 Adult IBD populations with receipt of MMR vaccine as pediatric patients**	Serious[f]	Serious[g]	Serious[h]	Serious[i]	None	⊕⊖⊖⊖<br>VERY LOW		• Among pediatric IBD patients who received the complete 2-dose MMR series, serologic protection was present for 67.6% for measles, 63.3% for mumps, and 81.4% for rubella.[5] Cannot distinguish waning titer vs. primary vaccination failure.<br>• In the 1 cross-sectional study of adults with confirmed vaccination records of 2- dose MMR prior to IBD diagnosis (as children), there was no difference in pre-set antibody threshold for presumptive immunity or antibody concentration for measles, mumps and rubella between IBD patients vs. healthy controls or among the IBD treatment groups (AZA/6MP, anti-TNF monotherapy, combination treatment).[4]
**Adverse events – CRITICAL**								
1 systematic review[1] of 5 RCTs, 1 controlled clinical trial, 27 cohort studies, 17- case-control studies, 5 time-series trials, 1 case cross-over trial, 2 ecological studies, 6 self-controlled case series studies<br><br>**Healthy Children**<br><br>*Adapted from WHO Evidence Profile Table*	Serious[a]	Not serious	Not serious[j]<br>Not on IS<br><br>Very serious[j]<br>On IS	Not Serious	None	⊕⊕⊕⊖<br>MODERATE<br>Not on IS<br><br>⊕⊖⊖⊖<br>VERY LOW<br>On IS		• MMR was associated with a lower incidence of upper respiratory tract infections, a higher incidence of irritability, and a similar incidence of other adverse events compared to placebo.<br>• MMR vaccine was likely to be associated with aseptic meningitis (mumps) using Urabe strain-containing MMR, parotitis, joint and limb complaints, febrile seizures and benign thrombocytopenic purpura within 2 weeks of vaccination.<br>• Exposure to MMR was unlikely to be associated with Crohn's disease, ulcerative colitis, autism or aseptic meningitis (mumps) using Jeryl-Lynn strain-containing MMR.
1 SR of 40 Observational Studies[4]<br><br>**20,556 IMID patients with**	Serious[c]	Not serious	Serious[e]	Not serious	None	⊕⊖⊖⊖<br>VERY LOW		• The administration of live vaccines was safe in most studies of IMID patients on immunosuppressive medications<br>• Serious adverse events were reported in 11/20,556 (0.05%) IMID patients

**2852 IBD patients**								• Overall, 0.06% patients had infection through the vaccine strain. Infection was mild in most cases. However, 2 patients had fatal infection (yellow fever vaccine, BCG vaccine). **Mild infection through the vaccine strain in 0.2% IMID patients through MMR vaccine.**<br>• No increase flares of autoimmune diseases in most studies

6MP – 6 mercaptopurine
AZA – azathioprine
IS – immunosuppressive medications
MTX – methotrexate

Footnotes:

a. Grading of evidence for vaccine effectiveness of measles based on the study by Anonymous (1968), a RCT of 21,653 UK children. Numerous observational studies underlined the high vaccine effectiveness. In the Cochrane Systematic Review by Demicheli et al, most of the included observational studies were characterized by poor reporting or missing information about comparability between exposed or non-exposed groups, reporting and attrition bias; the composition of MMR vaccine was sometimes not reported. The design and reporting of safety outcomes in MMR vaccine studies, both pre- and post-marketing, were largely inadequate. The evidence of adverse events following vaccination with the MMR vaccine cannot be separated from its role in preventing the target diseases.
b. Downgraded for indirectness. Patient population is healthy children (not IBD patients). Observational studies suggested that MMR vaccines may be less immunogenic in IBD patients with or without immunosuppressive medications.
c. Downgraded for study limitations. Residual confounding cannot be ruled out given the observational nature of these studies (e.g. comorbidities, concurrent illnesses, disease activity and duration, nutritional status, and other factors which may affect the risk of MMR) as well as selection bias.
d. Downgraded for inconsistency in results.
e. Downgraded due to indirectness (population and surrogate outcomes). Only 14% (32852/20,556) IMID patients were patients with IBD (majority were children). Different immunosuppressive medications were used across studies. Surrogate outcomes were used for vaccine effectiveness.
f. Downgraded for study limitations. Exposure to MMR vaccines / wild-type infection in 2/4 studies were based on patients' recall of vaccination history or infection. Possible selection bias. Patients attending a tertiary referral center may differ systematically from other patients. Patients who agreed to participate in a study where serologic assays were measured may be different than patients who did not agree to participate. Residual confounding factors not adjusted (disease activity / severity, duration of disease nutritional status, comorbidities).
g. Downgraded for inconsistency in results. One study showed no differences in antibody concentration of measles, mumps or rubella between IBD patients and controls. Other studies (with no control group) showed a reduced serologic protection among IBD patients. However, given the cross-sectional nature of these studies, they cannot determine whether the reduced serologic protection is due to waning titers over time vs. primary vaccination failure.
h. Downgraded for indirectness. Surrogate outcomes of antibody titer were used for vaccine effectiveness. However, the ACIP emphasizes that MMR serologies may be falsely negative in previously vaccinated persons and considers the immunization record of MMR to be a reliable surrogate of immunity. Low antibody concentrations are not necessarily associated with susceptibility to infection. No definitive cut-off levels to protect against each virus. No serologic correlate of protection is accepted for mumps. Included studies have used different assays and different cut-offs for serologic correlates of protection for each virus.
i. Downgraded for imprecision. Small sample sizes.

j. Not downgraded for indirectness in pediatric IBD patients not on immunosuppressive. Downgraded for very serious indirectness in pediatric IBD patients on immunosuppressive medications (low risk of vaccine-induced infection, and very rare fatal outcomes with other live vaccines). Small number of IBD patients included in the systematic review, and sample size may not be large enough to detect rare adverse events.

References:

1. Demicheli V, Rivetti A, Debalini MG, Di Pietrantonj C. Vaccines for measles, mumps and rubella in children. Cochrane Database Syst Rev. 2012 Feb 15;(2):CD004407.
2. Anonymous. Vaccination against measles: clinical trial of live measles vaccine given alone and live vaccine preceded by killed vaccine. Second report to the medical research council by the measles vaccines committee. BMJ 1968;449-454.
3. Beasley RP, Detels R, Kim KS, Gale JL, Lin TL, Grayston JT. Prevention of rubella during an epidemic on Taiwan. HPV-77 and RA 27-3 rubella vaccines administered subcutaneously and intranasally HPV-77 vaccine mixed with mumps and-or measles vaccines. Am J Dis Child. 1969 Aug;118(2):301-6.
4. Croce E, Hatz C, Jonker EF, Visser LG, Jaeger VK, Bühler S. Safety of live vaccinations on immunosuppressive therapy in patients with immune-mediated inflammatory diseases, solid organ transplantation or after bone-marrow transplantation - A systematic review of randomized trials, observational studies and case reports. Vaccine. 2017 Mar 1;35(9):1216-1226.
5. Caldera F, Misch EA, Saha S, Wald A, Zhang Y, Hubers J, Megna B, Ley D, Reichelderfer M, Hayney MS. Immunosuppression Does Not Affect Antibody Concentrations to Measles, Mumps, and Rubella in Patients with Inflammatory Bowel Disease. Dig Dis Sci. 2019 Jan;64(1):189-195.
6. deBruyn JCC, Soon IS, Fonseca K, Feng S, Purtzki M, Goedhart C, Kuhn S, Vanderkooi OG, Wrobel I. Serologic Status of Routine Childhood Vaccines, Cytomegalovirus, and Epstein-Barr Virus in Children With Inflammatory Bowel Disease. Inflamm Bowel Dis. 2019 Jun 18;25(7):1218-1226.
7. Cleveland NK, Rodriquez D, Wichman A, Pan I, Melmed GY, Rubin DT. Many Inflammatory Bowel Disease Patients Are Not Immune to Measles or Pertussis. Dig Dis Sci. 2016 Oct;61(10):2972-2976.
8. Naganuma M, Nagahori M, Fujii T, Morio J, Saito E, Watanabe M. Poor recall of prior exposure to varicella zoster, rubella, measles, or mumps in patients with IBD. Inflamm Bowel Dis. 2013 Feb;19(2):418-22.
9. Wichmann A, Krugliak Cleveland N, Rubin DT. Safety and Efficacy of Live Measles Vaccine Administered to a Crohn's Disease Patient Receiving Vedolizumab. Am J Gastroenterol. 2016 Apr;111(4):577.

Evidence to Decision Table - Pediatric

**PICO 4A**	**In MMR-susceptible pediatric patients with IBD not on immunosuppressive therapy, should vaccination vs. no vaccination against mumps, measles, rubella (MMR) be given?**
**Population**	**MMR-susceptible pediatric patients with IBD not on immunosuppressive therapy**

**Intervention**	Vaccination against MMR
**Comparator**	No vaccination against MMR
**Outcome**	Mortality, VPI (MMR infection), SAEs, Immunogenicity

**PICO 4B**	**In MMR-susceptible pediatric patients with IBD on immunosuppressive therapy, should vaccination vs. no vaccination against mumps, measles, rubella (MMR) be given?**
**Population**	**MMR-susceptible pediatric patients with IBD on immunosuppressive therapy**
**Intervention**	Vaccination against MMR
**Comparator**	No vaccination against MMR
**Outcome**	Mortality, VPI (MMR infection), SAEs, Immunogenicity

	Judgement	Research evidence	Additional considerations
**Desirable Effects**	**How substantial are the desirable anticipated effects?**<br><br>○ Trivial<br>○ Small<br>○ Moderate<br>○ Large On and not on IS<br><br>○ Varies<br>○ Don't know	**Risk of MMR in IBD patients**<br><br>The literature search did not identify any study on the risk of measles, mumps and rubella in adult or pediatric IBD patients.<br><br>**Effectiveness and Safety of MMR Vaccine in IBD patients**<br><br>There was no RCT comparing MMR vaccines with placebo in patients with IBD to address this PICO question.<br><br>One Cochrane systematic review assessed the evidence on the effectiveness of MMR vaccines in healthy children and reported one MMR vaccine dose to be at least 95% effective in preventing clinical measles and 92% effective in preventing secondary cases among household contacts.[1] Effectiveness of at least 1 dose of MMR in preventing clinical mumps in children is estimated to be between 69-81% depending on the vaccine strain.[1] No studies were identified for effectiveness of rubella[1]. The World Health Organization (WHO) assessed the evidence on the effectiveness of measles and rubella vaccines in healthy children in 2017 and 2011, respectively. In the WHO evidence profile, they also included 2 RCTs on clinical efficacy in measles and rubella.[2,3] Overall, the WHO rated the quality of evidence as high for MMR vaccination in immunocompetent healthy children in preventing measles, mumps and rubella.	
**Undesirable Effects**	**How substantial are the undesirable anticipated effects?**<br><br>○ Large<br>○ Moderate<br>○ Small On IS<br>○ Trivial Not on IS<br><br>○ Varies		

	○ Don't know	One systematic review included 40 observational studies (mostly cohort studies and case series/reports) in patients with immune-mediated diseases (2852/20,556 were IBD patients) on 22 different immunosuppressive medications.[4] The immunosuppressive medications used by IMID patients included prednisone 2.5-35mg/day, methotrexate 5-27mg/week, 6MP, biologic monotherapy, and combination therapy with biologic and immunomodulator.[4] The results are somewhat inconsistent in that some studies showed reduced antibody titer when vaccinated while on immunosuppressive medications, while other studies showed no significant differences in humoral responses between patients with and without methotrexate or biologics after MMR vaccination.[4] Four cross-sectional studies (1 in pediatric patients, and 3 in adult IBD patients who received MMR vaccines in their childhood) assessed the serologic status of MMR in IBD patients with the majority of patients on immunosuppressive medications.[5-8] Among pediatric IBD patients who received the complete 2-dose MMR series, serologic protection was present for 67.6% for measles, 63.3% for mumps, and 81.4% for rubella.[6] In a cross-sectional study of adults with confirmed vaccination records of 2- dose MMR prior to IBD diagnosis (as children), there was no difference in pre-set antibody threshold for presumptive immunity for measles (87% vs. 75%) and rubella (74% vs. 85%), and no difference in antibody concentration for measles, mumps and rubella between IBD patients vs. healthy controls or among the IBD treatment groups (AZA/6MP, anti-TNF monotherapy, combination treatment).[5] It is important to note that these cross-sectional studies used different cut-off values for antibody titer for immunity or seroprotection, and cannot distinguish between waning titers over time vs. primary vaccination failure. There is no definitive serologic correlate of specific antibody levels with clinical protection against each virus (although the USA Food and Drug Administration accepted IgG antibody concentration $\geq$ 200 mIU/mL for anti-measles, $\geq$ 10 EU/mL for anti-mumps, and $\geq$ 10 IU/mL for anti-rubella as offering "clinical benefit"). As well, there has been no clear definition with supporting evidence that low titers of antibody cause more infection among vaccinated populations. In fact, the ACIP emphasizes that MMR serologies may be falsely negative in previously vaccinated persons and considers the immunization record of MMR to be a reliable surrogate of immunity. However, serologic testing may be used for situations where there is no record of prior immunization or infection. ACIP recommends against giving additional doses of MMR vaccine for individuals with negative serologies and proof of age-appropriate immunization. The GRADE rating started as low due to observational nature of these studies. The evidence was further downgraded to **very low** due to study limitations (residual confounding and selection bias) and indirectness (surrogate outcomes).<br><br>**The certainty of evidence for effectiveness was anchored to the general population (healthy children**), and started as high. When the evidence is applied to IBD patients, the evidence was downgraded to **moderate** due to indirectness as observational studies suggested that MMR vaccines may be less immunogenic in IBD patients. In summary, there is moderate certainty evidence that MMR vaccines are effective in reducing the risks of MMR in pediatric IBD patients.<br><br>The Cochrane systematic review also assessed the safety of MMR vaccines in healthy children.[1] MMR was associated with a lower incidence of upper respiratory tract	

		infections, a higher incidence of irritability, and a similar incidence of other adverse events compared to placebo.[1] MMR vaccine was likely to be associated with aseptic meningitis (mumps) using Urabe strain-containing MMR, parotitis, joint and limb complaints, febrile seizures and benign thrombocytopenic purpura within 2 weeks of vaccination.[1] Exposure to MMR was unlikely to be associated with Crohn's disease, ulcerative colitis, autism or aseptic meningitis (mumps) using Jeryl-Lynn strain-containing MMR.[1] The WHO also assessed the evidence on the safety of measles and rubella vaccines in healthy children incorporating the findings of the Cochrane systematic review. The certainty of evidence was rated as moderate due to study limitations (inadequate reporting of adverse events).<br><br>One systematic review included 40 observational studies (mostly cohort studies and case series/reports) in patients with immune-mediated diseases (2852/20,556 were IBD patients) on 22 different immunosuppressive medications.[4] The administration of live vaccines was safe in most studies of IMID patients on immunosuppressive medications (including prednisone 2.5-35mg/day, methotrexate 5-27mg/week, 6MP, biologic monotherapy, and combination therapy with biologic and immunomodulator). Serious adverse events were rare (0.05%, 11/20,556 IMID patients). Infections through the vaccine strain were also rare, occurring in 0.06% (12/20,556 IMID patients) for all live vaccines, and 0.2% IMID patients through MMR vaccine. In most cases, infection was mild. However, two patients had fatal infection: a patient with RA/SLE overlap who started methotrexate/dexamethasone treatment 4 days after the yellow fever vaccine developed vaccine-associated viscerotropic disease and died. One infant whose mother was under infliximab treatment during pregnancy received the BCG vaccine at the age of 3 months and developed disseminated BCG infection and died. The certainty of evidence started as low due to the observational designs of these studies. The evidence was downgraded to **very low** due to study limitations and indirectness (patient population).<br><br>**The certainty of evidence for safety was anchored to the general population (healthy children)**, and started as **moderate**. When the evidence is applied to **pediatric IBD patients not on immunosuppressive medications**, the evidence was not downgraded for indirectness. However, the evidence was downgraded to <u>**very low**</u> due to serious indirectness when applied to **pediatric IBD patients on immunosuppressive medications**. Although the risk of vaccine-induced infection with MMR seemed to be very rare in patients with immune-mediated diseases (including IBD), very rare fatal outcomes did occur following the administration of other live vaccines. As well, the small number of IBD patients on immunosuppressive medications included in the systematic review may not be large enough to detect rare adverse events.<br><br>**Overall, there is <u>moderate</u> certainty evidence that MMR is safe and effective in pediatric IBD patients not on immunosuppressive medications. There is <u>very low</u> certainty evidence that MMR is safe and effective in pediatric IBD patients on immunosuppressive medications.**	

**Certainty of evidence**	**What is the overall certainty of the evidence of effects?**<br><br>○ Very low pediatric IBD patients on IS<br>○ Low<br>○ Moderate pediatric IBD patients not on IS<br>○ High<br><br>○ No included studies		
**Values and Preferences**	**Is there important uncertainty about or variability in how much people value the main outcomes?**<br><br>○ Important uncertainty or variability<br>○ Possibly important uncertainty or variability<br>○ Probably no important uncertainty or variability<br>○ No important uncertainty or variability	Patients likely value patient-important outcomes (mortality, VPI, adverse effects) more than surrogate outcomes (immunogenicity).	
**Balance of effects**	**Does the balance between desirable and undesirable effects favor the intervention or the comparison?**<br><br>○ Favors the comparison<br>○ Probably favors the comparison<br>○ Does not favor either the intervention or the comparison<br>○ Probably favors the intervention On IS<br>○ Favors the intervention Not on IS<br><br>○ Varies<br>○ Don't know		
**Resources required**	**How large are the resource requirements (costs)?**<br><br>○ Large costs<br>○ Moderate costs<br>○ Negligible costs and savings<br>○ Moderate savings<br>○ Large savings<br><br>○ Varies<br>○ Don't know	CDC vaccine price list last reviewed/updated: July 1, 2019<br><br>(see table below)	

Brandname	CDC cost/dose	Private sector cost/dose
MMR: M-M-R®II	$21.22	$78.68
MMRV: ProGuad®	$131.40	$224.94

**Certainty of Evidence of Required Resources**	**What is the certainty of the evidence of resource requirements (costs)?**<br><br>o Very low<br>o Low<br>o Moderate<br>o High<br><br>o No included studies	The costs of delivering routine immunization services may vary widely across countries and different health system settings. See Immunization Costing Action Network (ICAN) Immunization Delivery Cost Catalogue. http://immunizationeconomics.org/ican-idcc.	
**Cost effectiveness**	**Does the cost-effectiveness of the intervention favor the intervention or the comparison?**<br><br>o Favors the comparison<br>o Probably favors the comparison<br>o Does not favor either the intervention or the comparison<br>o Probably favors the intervention<br>o Favors the intervention On and not on IS<br><br>o Varies<br>o No included studies	There is no published study of cost-effectiveness of MMR vaccine in pediatric IBD patients.<br><br>An economic analysis showed that the national 2-dose MMR vaccination program in the US is highly cost-beneficial and results in substantial cost savings from both the direct cost and societal perspectives compared with the absence of MMR vaccination under even the most conservative assumptions.[9] All costs were estimated for a hypothetical US birth cohort of 3803295 infants born in 2001. The 2-dose MMR vaccination program was cost-saving from both the direct cost and societal perspectives compared with the absence of MMR vaccination, with net savings (net present value) from the direct cost and societal perspectives of US dollars 3.5 billion and US dollars 7.6 billion, respectively. The direct and societal benefit-cost ratios for the MMR vaccination program were 14.2 and 26.0. Analysis of the incremental benefit-cost of the second dose showed that direct and societal benefit-cost ratios were 0.31 and 0.49, respectively. Varying the proportion of vaccines purchased and administered in the public versus the private sector had little effect on the results.	
**Acceptability**	**Is the intervention acceptable to key stakeholders?**<br><br>o No<br>o Probably no<br>o Probably yes<br>o Yes Not on IS<br><br>o Varies On IS<br>o Don't know	In a survey study assessing parent's attitudes regarding their choice to postpone or abstain from MMR vaccination in Sweden, the most common reason for non-vaccination was fear of side effects.[10]<br><br>Analyses of findings from the 2013 Childhood National Immunization Coverage Survey data in Canada, non-receipt of minimum age-specific vaccination dosages for MMR was associated with concerns about side effects and lower perceived importance of immunizing a child with MMR.[11]<br><br>In a systematic review of the determinants of European parents' decision on the vaccination of their children against MMR, the following factors were associated with lower MMR vaccine uptake: misleading knowledge, beliefs and perceptions on vaccines (OR 0.57, CI 0.37-0.87); negative attitudes and behaviors toward vaccination (OR 0.71, CI 0.52-0.98); demographic characteristics, such as different ethnicity in Southern populations (OR 0.44, CI 0.31-0.61), higher child's age (OR 0.80, CI 0.76-0.85); low socio-economic status (OR 0.64, CI 0.51-0.80), especially low income (OR 0.39, CI 0.25-0.60) and education (OR 0.64, CI 0.48-0.84), high number of children (OR 0.54, CI 0.42-0.69),	

		irregular marital status (OR 0.80, CI 0.66-0.96).[12]	
**Feasibility**	**Is the intervention feasible to implement?**<br><br>○ No<br>○ Probably no<br>○ Probably yes<br>○ Yes On and not on IS<br><br>○ Varies<br>○ Don't know		

References:

1. Demicheli V, Rivetti A, Debalini MG, Di Pietrantonj C. Vaccines for measles, mumps and rubella in children. Cochrane Database Syst Rev. 2012 Feb 15;(2):CD004407.
2. Anonymous. Vaccination against measles: clinical trial of live measles vaccine given alone and live vaccine preceded by killed vaccine. Second report to the medical research council by the measles vaccines committee. BMJ 1968;449-454.
3. Beasley RP, Detels R, Kim KS, Gale JL, Lin TL, Grayston JT. Prevention of rubella during an epidemic on Taiwan. HPV-77 and RA 27-3 rubella vaccines administered subcutaneously and intranasally HPV-77 vaccine mixed with mumps and-or measles vaccines. Am J Dis Child. 1969 Aug;118(2):301-6.
4. Croce E, Hatz C, Jonker EF, Visser LG, Jaeger VK, Bühler S. Safety of live vaccinations on immunosuppressive therapy in patients with immune-mediated inflammatory diseases, solid organ transplantation or after bone-marrow transplantation - A systematic review of randomized trials, observational studies and case reports. Vaccine. 2017 Mar 1;35(9):1216-1226.
5. Caldera F, Misch EA, Saha S, Wald A, Zhang Y, Hubers J, Megna B, Ley D, Reichelderfer M, Hayney MS. Immunosuppression Does Not Affect Antibody Concentrations to Measles, Mumps, and Rubella in Patients with Inflammatory Bowel Disease. Dig Dis Sci. 2019 Jan;64(1):189-195.
6. deBruyn JCC, Soon IS, Fonseca K, Feng S, Purtzki M, Goedhart C, Kuhn S, Vanderkooi OG, Wrobel I. Serologic Status of Routine Childhood Vaccines, Cytomegalovirus, and Epstein-Barr Virus in Children With Inflammatory Bowel Disease. Inflamm Bowel Dis. 2019 Jun 18;25(7):1218-1226.
7. Cleveland NK, Rodriquez D, Wichman A, Pan I, Melmed GY, Rubin DT. Many Inflammatory Bowel Disease Patients Are Not Immune to Measles or Pertussis. Dig Dis Sci. 2016 Oct;61(10):2972-2976.
8. Naganuma M, Nagahori M, Fujii T, Morio J, Saito E, Watanabe M. Poor recall of prior exposure to varicella zoster, rubella, measles, or mumps in patients with IBD. Inflamm Bowel Dis. 2013 Feb;19(2):418-22.
9. Zhou F, Reef S, Massoudi M, Papania MJ, Yusuf HR, Bardenheier B, Zimmerman L, McCauley MM. An economic analysis of the current universal 2-dose measles-mumps-rubella vaccination program in the United States. J Infect Dis. 2004 May 1;189 Suppl 1:S131-45.
10. Dannetun E, Tegnell A, Hermansson G, Giesecke J. Parents' reported reasons for avoiding MMR vaccination. A telephone survey. Scand J Prim Health Care. 2005 Sep;23(3):149-53.
11. Carpiano RM, Polonijo AN, Gilbert N, Cantin L, Dubé E. Socioeconomic status differences in parental immunization attitudes and child immunization in Canada: Findings from the 2013 Childhood National Immunization Coverage Survey (CNICS). Prev Med. 2019 Jun;123:278-287.

12. Tabacchi G, Costantino C, Napoli G, Marchese V, Cracchiolo M, Casuccio A, Vitale F; The Esculapio Working Group. Determinants of European parents' decision on the vaccination of their children against measles, mumps and rubella: A systematic review and meta-analysis. Hum Vaccin Immunother. 2016 Jul 2;12(7):1909-23.

Conclusion - Pediatric

PICO 4A: **In measles-susceptible pediatric patients with IBD not on immunosuppressive therapy, should vaccination vs. no vaccination against mumps, measles, rubella (MMR) be given?**
Moderate certainty of evidence
Direction – yes (100%)
Strength – Strong (100%)

**Type of recommendation**	**Strong recommendation against the intervention**<br>○	**Conditional recommendation against the intervention**<br>○	**Conditional recommendation for either the intervention or the comparison**<br>○	**Conditional recommendation for the intervention**<br>○	**Strong recommendation for the intervention**<br>○
**Recommendation**	**Statement 4A: In measles-susceptible pediatric patients with IBD not on immunosuppressive therapy, we recommend MMR vaccine be given.**				
**Justification**					
**Subgroup considerations**	• The severity or degree of immunosuppression likely varies with severity or activity of disease, nutritional status, comorbidities, types and dosages of medications used.				
**Implementation considerations**					

**Monitoring and evaluation**	• Ongoing monitoring of serious adverse events associated with MMR vaccines in IBD patients
**Research priorities**	• Observational studies to determine the risks of MMR infection in pediatric IBD patients compared to the general population<br>• RCTs or observational studies to determine the clinical effectiveness and immunogenicity of MMR vaccines in pediatric IBD patients with assessment of patient-important outcomes (i.e. MMR infection)

PICO 4B: **In MMR-susceptible pediatric patients with IBD on immunosuppressive therapy, should vaccination vs. no vaccination against mumps, measles, rubella (MMR) be given?**
Very low certainty of evidence
Direction – Yes (), Uncertain (), No (100%)

Type of recommendation	Strong recommendation against the intervention	Conditional recommendation against the intervention	Conditional recommendation for either the intervention or the comparison	Conditional recommendation for the intervention	Strong recommendation for the intervention
	○	○	○	○	○
Recommendation	**Statement 4B: In MMR-susceptible pediatric patients with IBD on immunosuppressive therapy, we suggest against giving MMR vaccine.**				
Justification					

Subgroup considerations	• The severity or degree of immunosuppression likely varies with severity or activity of disease, nutritional status, comorbidities, types and dosages of medications used.
Implementation considerations	
Monitoring and evaluation	• Ongoing monitoring of serious adverse events associated with MMR vaccines in IBD patients
Research priorities	• Observational studies to determine the risks of MMR infection in pediatric IBD patients compared to the general population<br>• RCTs or observational studies to determine the clinical effectiveness and immunogenicity of MMR vaccines in pediatric IBD patients with assessment of patient-important outcomes (i.e. MMR infection)

Summary – Adult

**PICO 5A**	**In MMR-susceptible adult patients with IBD not on immunosuppressive therapy, should vaccination vs. no vaccination against mumps, measles, rubella (MMR) be given?**
**Population**	**MMR-susceptible adult patients with IBD not on immunosuppressive therapy**
**Intervention**	Vaccination against MMR
**Comparator**	No vaccination against MMR
**Outcome**	Mortality, VPI (MMR infection), SAEs, Immunogenicity

**PICO 5B**	**In MMR-susceptible adult patients with IBD on immunosuppressive therapy, should vaccination vs. no vaccination against mumps, measles, rubella (MMR) be given?**

Population	**MMR-susceptible adult patients with IBD on immunosuppressive therapy,**
**Intervention**	Vaccination against MMR
**Comparator**	No vaccination against MMR
**Outcome**	Mortality, VPI (MMR infection), SAEs, Immunogenicity

There was no RCT or observational studies comparing MMR vaccine with placebo or no treatment in adult patients with IBD to address this PICO question.

There are scarce data in the literature on the immunogenicity, clinical effectiveness, and safety of MMR vaccines administered outside of the recommended schedule (one dose at 12-15 months of age and a second dose typically at 4-6 years of age). One RCT compared 2 MMR vaccines (MMR-RIT vs. M-M-R II) in individuals aged > 7-year-olds who had received > 1 previous dose of MMR vaccine. Most of the participants (64.1%) were adults aged > 18 (mean age 25.7 years).[9] Seroresponse rates were defined as IgG antibody concentration > 200 mIU/mL for measles, > 10 EU/mL for mumps, and > 10IU/mL for rubella as per the thresholds used by the USA Food and Drug Administration as offering clinical benefit. The seroresponse rates of MMR-RIT were found to be non-inferior compared to M-M-R II (98.8% for measles, 98.4% for mumps, 99.5% for rubella).[9] It is important to note that the results of this study represent secondary / anamnestic immune response rather than primary immune response to MMR vaccines in adults. No serious adverse events were noted in both arms.[9] Nevertheless, the immunogenicity and safety data of both arms of this study supported the immunogenicity findings in the pediatric population. Hence, the evidence was not downgraded for indirectness (pediatric vs. adult population) as the evidence would be anchored to the pediatric population.

A case report details safe administration of MMR vaccine in a 26-year-old female with Crohn's ileocolitis who was receiving vedolizumab and methotrexate. Methotrexate was discontinued and after a 2 week wash out period the MMR vaccine was administered.[10] The methotrexate was recommenced four weeks after vaccination. Measles antibody index was positive 8 weeks after vaccination and at 3 months follow up there had been no adverse sequelae.[10] This case report was not included in the evidence profile.

As in pediatric population, there is **moderate** certainty evidence that MMR vaccines are effective in reducing the risks of MMR in adult IBD populations. There is **moderate** certainty evidence that MMR vaccines are safe in adult IBD populations not on immunosuppressive medications). There is **very low** certainty evidence that MMR vaccines are safe in adult IBD populations on immunosuppressive medications).

Overall, there is <u>moderate</u> certainty evidence MMR vaccines are safe and effective in adult IBD patients not on immunosuppressive medications. There is <u>very low</u> certainty evidence that MMR vaccines are safe and effective in adult IBD populations on immunosuppressive medications.

In Adults, CDC currently recommends MMR vaccines only be administered in susceptible individuals in high risk groups (e.g. college students, health-care workers, military personnel), in immigrants or travelers without proper vaccination, and in outbreak settings.

Evidence Profile Table - Adults

Certainty Assessment								Summary of Findings	
Studies	Risk of bias	Inconsistency	Indirectness	Imprecision	Other considerations	Certainty of Evidence	Overall Certainty of evidence	Study Event Rates	Relative Effect (95% CI)
**VPI (clinical and/or confirmed cases of MMR) - CRITICAL**							⊕⊕⊕⊖ MODERATE<br><br>For adult IBD patients not on IS<br><br>⊕⊖⊖⊖ VERY LOW<br><br>For adult IBD patients on IS		
1 systematic review[1] of 5 RCTs, 1 controlled clinical trial, 27 cohort studies, 17 case-control studies, 5 time-series trials, 1 case cross-over trial, 2 ecological studies, 6 self-controlled case series studies<br><br>2 RCT[2,3]<br><br>**Healthy Children**<br><br>*Adapted from WHO Evidence Profile Table*	Not serious[a]	Not serious	Serious[b]	Not Serious	None	⊕⊕⊕⊖ MODERATE		• One MMR vaccine dose is at least 95% effective in preventing clinical measles and 92% effective in preventing secondary cases among household contacts.[1,3]<br>• One dose of MMR in preventing clinical mumps in children is estimated to be between 69-81% for the vaccine prepared with Jeryl Lynn mumps strain and between 70-75% for the vaccine containing the Urabe strain.[3]<br>• 1 RCT on the protective efficacy of rubella vaccine against clinical rubella (198 children in China) during a rubella outbreak. Protection against rubella was achieved in > 95% of those who received the vaccine.[2]	
**Immunogenicity (MMR antibody titer) - IMPORTANT**									
1 SR of 40 Observational Studies[4]	Serious[c]	Serious[d]	Serious[e]	Not serious	None	⊕⊖⊖⊖ VERY LOW		• Some studies showed reduced antibody titer when vaccinated while on IS. Other studies showed no significant differences in humoral responses between patients with and without MTX or	

**20,556 IMID patients with 2852 IBD patients**								biologics after MMR vaccination.
4 Observational studies[5-8]<br><br>**1 Pediatric IBD populations**<br><br>**3 Adult IBD populations with receipt of MMR vaccine as pediatric patients**	Serious[f]	Serious[g]	Serious[h]	Serious[i]	None	⊕⊖⊖⊖<br>VERY LOW		• Among pediatric IBD patients who received the complete 2-dose MMR series, serologic protection was present for 67.6% for measles, 63.3% for mumps, and 81.4% for rubella.[5] Cannot distinguish waning titer vs. primary vaccination failure.<br>• In the 1 cross-sectional study of adults with confirmed vaccination records of 2- dose MMR prior to IBD diagnosis (as children), there was no difference in pre-set antibody threshold for presumptive immunity or antibody concentration for measles, mumps and rubella between IBD patients vs. healthy controls or among the IBD treatment groups (AZA/6MP, anti-TNF monotherapy, combination treatment).[4]
1 RCT[9]<br>(Observational data from 2 arms of RCT)<br><br>**Mostly healthy adult populations**	Not serious	Not serious	Serious[j]	Not serious	None	⊕⊖⊖⊖<br>VERY LOW		• Among individuals $\geq$ 7-year-olds who received $\geq$ 1 previous dose of MMR vaccine, the seroresponse rates to MMR-RIT and MMRII vaccines were comparable (98.8% vs. 99.1% against measles, 98.4% vs. 99.5% against mumps, 99.5% vs. 99.8% against rubella)
**Adverse events - CRITICAL**								
1 systematic review[1] of 5 RCTs, 1 controlled clinical trial, 27 cohort studies, 17- case-control studies, 5 time-series trials, 1 case cross-over trial, 2 ecological studies, 6 self-controlled case series studies<br><br>**Healthy Children**<br><br>*Adapted from WHO Evidence Profile Table*	Serious[a]	Not serious	Not serious[k]<br>Not on IS<br><br>Very serious[k]<br>On IS	Not Serious	None	⊕⊕⊕⊖<br>MODERATE<br>Not on IS<br><br>⊕⊖⊖⊖<br>VERY LOW<br>On IS		• MMR was associated with a lower incidence of upper respiratory tract infections, a higher incidence of irritability, and a similar incidence of other adverse events compared to placebo.<br>• MMR vaccine was likely to be associated with aseptic meningitis (mumps) using Urabe strain-containing MMR, parotitis, joint and limb complaints, febrile seizures and benign thrombocytopenic purpura within 2 weeks of vaccination.<br>• Exposure to MMR was unlikely to be associated with Crohn's disease, ulcerative colitis, autism or aseptic meningitis (mumps) using Jeryl-Lynn strain-containing MMR.
1 SR of 40 Observational Studies[4]<br><br>**20,556 IMID patients with 2852 IBD patients**	Serious[c]	Not serious	Serious[e]	Not serious	None	⊕⊖⊖⊖<br>VERY LOW		• The administration of live vaccines was safe in most studies of IMID patients on immunosuppressive medications<br>• Serious adverse events were reported in 11/20,556 (0.05%) IMID patients<br>• Overall, 0.06% patients had infection through the vaccine strain. Infection was mild in most cases. However, 2 patients had fatal infection (yellow fever vaccine, BCG vaccine). **Mild infection through the vaccine strain in 0.2% IMID patients**

								**through MMR vaccine.**<br>• No increase flares of autoimmune diseases in most studies
1 RCT[9]<br>(Observational data from 2 arms of RCT)<br><br>**Mostly healthy adult populations**	Not serious	Not serious	Serious[j]	Not serious	None	⨁⊖⊖⊖<br>VERY LOW		• No serious adverse events

Footnotes:

a. Grading of evidence for vaccine effectiveness of measles based on the study by Anonymous (1968), a RCT of 21,653 UK children. Numerous observational studies underlined the high vaccine effectiveness. In the Cochrane Systematic Review by Demicheli et al, most of the included observational studies were characterized by poor reporting or missing information about comparability between exposed or non-exposed groups, reporting and attrition bias; the composition of MMR vaccine was sometimes not reported. The design and reporting of safety outcomes in MMR vaccine studies, both pre- and post-marketing, were largely inadequate. The evidence of adverse events following vaccination with the MMR vaccine cannot be separated from its role in preventing the target diseases.

b. Downgraded for indirectness. Patient population is healthy children (not adult IBD patients). Observational studies suggested that MMR vaccines may be less immunogenic in IBD patients with or without immunosuppressive medications.

c. Downgraded for study limitations. Residual confounding cannot be ruled out given the observational nature of these studies (e.g. comorbidities, concurrent illnesses, disease activity and duration, nutritional status, and other factors which may affect the risk of MMR) as well as selection bias.

d. Downgraded for inconsistency in results.

e. Downgraded due to indirectness (population and surrogate outcomes). Only 14% (32852/20,556) IMID patients were patients with IBD (majority were children). Different immunosuppressive medications were used across studies. Surrogate outcomes were used for vaccine effectiveness.

f. Downgraded for study limitations. Exposure to MMR vaccines / wild-type infection in 2/4 studies were based on patients' recall of vaccination history or infection. Possible selection bias. Patients attending a tertiary referral center may differ systematically from other patients. Patients who agreed to participate in a study where serologic assays were measured may be different than patients who did not agree to participate. Residual confounding factors not adjusted (disease activity / severity, duration of disease nutritional status, comorbidities).

g. Downgraded for inconsistency in results. One study showed no differences in antibody concentration of measles, mumps or rubella between IBD patients and controls. Other studies (with no control group) showed a reduced serologic protection among IBD patients. However, given the cross-sectional nature of these studies, they cannot determine whether the reduced serologic protection is due to waning titers over time vs. primary vaccination failure.

h. Downgraded for indirectness. Surrogate outcomes of antibody titer were used for vaccine effectiveness. However, the ACIP emphasizes that MMR serologies may be falsely negative in previously vaccinated persons and considers the immunization record of MMR to be a reliable surrogate of immunity. Low antibody concentrations are not necessarily associated with susceptibility to infection. No definitive cut-off levels to protect against each virus. No serologic correlate of protection is accepted for mumps. Included studies have used different assays and different cut-offs for serologic correlates of protection for each virus.

i. Downgraded for imprecision. Small sample sizes.

j. Downgraded for indirectness. Patient population is healthy adults (not IBD patients). Surrogate outcomes of immunogenicity were used and the results were based on secondary (anamnestic response) rather than primary immune response to MMR vaccines.
k. Not downgraded for indirectness in adult IBD patients not on immunosuppressive. Downgraded for very serious indirectness in adult IBD patients on immunosuppressive medications (low risk of vaccine-induced infection, and very rare fatal outcomes with other live vaccines). Small number of IBD patients included in the systematic review, and sample size may not be large enough to detect rare adverse events.

References:

1. Demicheli V, Rivetti A, Debalini MG, Di Pietrantonj C. Vaccines for measles, mumps and rubella in children. Cochrane Database Syst Rev. 2012 Feb 15;(2):CD004407.
2. Anonymous. Vaccination against measles: clinical trial of live measles vaccine given alone and live vaccine preceded by killed vaccine. Second report to the medical research council by the measles vaccines committee. BMJ 1968;449-454.
3. Beasley RP, Detels R, Kim KS, Gale JL, Lin TL, Grayston JT. Prevention of rubella during an epidemic on Taiwan. HPV-77 and RA 27-3 rubella vaccines administered subcutaneously and intranasally HPV-77 vaccine mixed with mumps and-or measles vaccines. Am J Dis Child. 1969 Aug;118(2):301-6.
4. Croce E, Hatz C, Jonker EF, Visser LG, Jaeger VK, Bühler S. Safety of live vaccinations on immunosuppressive therapy in patients with immune-mediated inflammatory diseases, solid organ transplantation or after bone-marrow transplantation - A systematic review of randomized trials, observational studies and case reports. Vaccine. 2017 Mar 1;35(9):1216-1226.
5. Caldera F, Misch EA, Saha S, Wald A, Zhang Y, Hubers J, Megna B, Ley D, Reichelderfer M, Hayney MS. Immunosuppression Does Not Affect Antibody Concentrations to Measles, Mumps, and Rubella in Patients with Inflammatory Bowel Disease. Dig Dis Sci. 2019 Jan;64(1):189-195.
6. deBruyn JCC, Soon IS, Fonseca K, Feng S, Purtzki M, Goedhart C, Kuhn S, Vanderkooi OG, Wrobel I. Serologic Status of Routine Childhood Vaccines, Cytomegalovirus, and Epstein-Barr Virus in Children With Inflammatory Bowel Disease. Inflamm Bowel Dis. 2019 Jun 18;25(7):1218-1226.
7. Cleveland NK, Rodriquez D, Wichman A, Pan I, Melmed GY, Rubin DT. Many Inflammatory Bowel Disease Patients Are Not Immune to Measles or Pertussis. Dig Dis Sci. 2016 Oct;61(10):2972-2976.
8. Naganuma M, Nagahori M, Fujii T, Morio J, Saito E, Watanabe M. Poor recall of prior exposure to varicella zoster, rubella, measles, or mumps in patients with IBD. Inflamm Bowel Dis. 2013 Feb;19(2):418-22.
9. Abu-Elyazeed R, Jennings W, Severance R, Noss M, Caplanusi A, Povey M, Henry O. Immunogenicity and safety of a second dose of a measles-mumps-rubella vaccine administered to healthy participants 7 years of age or older: A phase III, randomized study. Hum Vaccin Immunother. 2018;14(11):2624-2631.
10. Wichmann A, Krugliak Cleveland N, Rubin DT. Safety and Efficacy of Live Measles Vaccine Administered to a Crohn's Disease Patient Receiving Vedolizumab. Am J Gastroenterol. 2016 Apr;111(4):577.

Evidence to Decision Table - Adults

**PICO 5A**	**In MMR-susceptible adult patients with IBD not on immunosuppressive**

	therapy, should vaccination vs. no vaccination against mumps, measles, rubella (MMR) be given?
**Population**	**Measles-susceptible adult patients with IBD not on immunosuppressive therapy**
**Intervention**	Vaccination against MMR
**Comparator**	No vaccination against MMR
**Outcome**	Mortality, VPI (MMR infection), SAEs, Immunogenicity

**PICO 5B**	**In MMR-susceptible adult patients with IBD on immunosuppressive therapy, should vaccination vs. no vaccination against mumps, measles, rubella (MMR) be given?**
**Population**	**Measles-susceptible adult patients with IBD on immunosuppressive therapy,**
**Intervention**	Vaccination against MMR
**Comparator**	No vaccination against MMR
**Outcome**	Mortality, VPI (MMR infection), SAEs, Immunogenicity

	Judgement	Research evidence	Additional considerations
**Desirable Effects**	**How substantial are the desirable anticipated effects?**<br><br>o Trivial<br>o Small<br>o Moderate<br>o Large on and not on IS<br><br>o Varies<br>o Don't know	**Risk of MMR in IBD patients**<br><br>The literature search did not identify any study on the risk of measles, mumps and rubella in adult or pediatric IBD patients.<br><br>**Effectiveness and Safety of MMR Vaccine in IBD patients**<br><br>There was no RCT or observational studies comparing MMR vaccine with placebo or no treatment in adult patients with IBD to address this PICO question.<br><br>There are scarce data in the literature on the immunogenicity, clinical effectiveness, and safety of MMR vaccines administered outside of the recommended schedule (one dose at 12-15 months of age and a second dose typically at 4-6 years of age). One RCT	In Adults, CDC currently recommends MMR vaccines be administered in susceptible individuals in high risk groups (e.g. college students, health-care workers, military personnel), in immigrants or travelers without proper vaccination, and in outbreak settings. The panel will need to decide whether all adult IBD patients are considered “susceptible individuals in high risk groups” as there is no evidence in the literature to suggest these individuals are at higher risk for having MMR compared to the general population, or only selected adult populations considered as high risk by CDC.

**Undesirable Effects**	**How substantial are the undesirable anticipated effects?**<br><br>○ Large<br>○ Moderate<br>○ Small On IS<br>○ Trivial not on IS<br><br>○ Varies<br>○ Don't know	compared 2 MMR vaccines (MMR-RIT vs. M-M-R II) in individuals aged $\geq$ 7-year-olds who had received $\geq$ 1 previous dose of MMR vaccine. Most of the participants (64.1%) were adults aged $\geq$ 18 (mean age 25.7 years).[9] Seroresponse rates were defined as IgG antibody concentration $\geq$ 200 mIU/mL for measles, $\geq$ 10 EU/mL for mumps, and $\geq$ 10IU/mL for rubella as per the thresholds used by the USA Food and Drug Administration as offering clinical benefit. The seroresponse rates of MMR-RIT were found to be non-inferior compared to M-M-R II (98.8% for measles, 98.4% for mumps, 99.5% for rubella).[9] It is important to note that the results of this study represent secondary / anamnestic immune response rather than primary immune response to MMR vaccines in adults. No serious adverse events were noted in both arms.[9] Nevertheless, the immunogenicity and safety data of both arms of this study supported the findings in the pediatric population. Hence, the evidence was not downgraded for indirectness (pediatric vs. adult population) as the evidence would be anchored to the pediatric population.<br><br>A case report details safe administration of MMR vaccine in a 26-year-old female with Crohn's ileocolitis who was receiving vedolizumab and methotrexate. Methotrexate was discontinued and after a 2 week wash out period the MMR vaccine was administered.[10] The methotrexate was recommenced four weeks after vaccination. Measles antibody index was positive 8 weeks after vaccination and at 3 months follow up there had been no adverse sequelae.[10] This case report was not included in the evidence profile.<br><br>As in pediatric population, there is **moderate** certainty evidence that MMR vaccines are effective in reducing the risks of MMR in adult IBD populations. There is **moderate** certainty evidence that varicella vaccines are safe in adult IBD populations not on immunosuppressive medications). There is **very low** certainty evidence that MMR vaccines are safe in adult IBD populations on immunosuppressive medications).<br><br>**Overall, there is moderate certainty evidence MMR vaccines are safe and effective in adult IBD patients not on immunosuppressive medications. There is very low certainty evidence that MMR vaccines are safe and effective in adult IBD populations on immunosuppressive medications.**	
**Certainty of evidence**	**What is the overall certainty of the evidence of effects?**<br><br>○ Very low adult IBD patients on IS<br>○ Low<br>○ Moderate IBD patients not on IS<br>○ High<br><br>○ No included studies		
**s and Prefe rence**	**Is there important uncertainty about or variability**	Patients likely value patient-important outcomes (mortality, VPI, adverse effects) more	

	**in how much people value the main outcomes?**<br><br>○ Important uncertainty or variability<br>○ Possibly important uncertainty or variability<br>○ Probably no important uncertainty or variability<br>○ No important uncertainty or variability	than surrogate outcomes (immunogenicity).	
**Balance of effects**	**Does the balance between desirable and undesirable effects favor the intervention or the comparison?**<br><br>○ Favors the comparison<br>○ Probably favors the comparison<br>○ Does not favor either the intervention or the comparison<br>○ Probably favors the intervention On IS<br>○ Favors the intervention not on IS<br><br>○ Varies<br>○ Don't know		
**Resources required**	**How large are the resource requirements (costs)?**<br><br>○ Large costs<br>○ Moderate costs<br>○ Negligible costs and savings<br>○ Moderate savings<br>○ Large savings<br><br>○ Varies<br>○ Don't know	CDC vaccine price list last reviewed/updated: July 1, 2019<br><br>**Brandname** / **CDC cost/dose** / **Private sector cost/dose**<br>MMR: M-M-R®II / $21.22 / $78.68<br>MMRV: ProGuad® / $131.40 / $224.94	
**Certainty of Evidence of Required Resources**	**What is the certainty of the evidence of resource requirements (costs)?**<br><br>○ Very low<br>○ Low<br>○ Moderate<br>○ High<br><br>○ No included studies	The costs of delivering routine immunization services may vary widely across countries and different health system settings. See Immunization Costing Action Network (ICAN) Immunization Delivery Cost Catalogue. http://immunizationeconomics.org/ican-idcc.	

Brandname	CDC cost/dose	Private sector cost/dose
MMR: M-M-R®II	$21.22	$78.68
MMRV: ProGuad®	$131.40	$224.94

**Cost effectiveness**	**Does the cost-effectiveness of the intervention favor the intervention or the comparison?**<br><br>○ Favors the comparison<br>○ Probably favors the comparison<br>○ Does not favor either the intervention or the comparison<br>○ Probably favors the intervention<br>○ Favors the intervention<br><br>○ Varies<br>○ No included studies	There is no published study of cost-effectiveness of MMR vaccine in adult patients (IBD or general population).	
**Acceptability**	**Is the intervention acceptable to key stakeholders?**<br><br>○ No<br>○ Probably no<br>○ Probably yes<br>○ Yes not on IS<br><br>○ Varies on IS<br>○ Don't know		
**Feasibility**	**Is the intervention feasible to implement?**<br><br>○ No<br>○ Probably no<br>○ Probably yes<br>○ Yes<br><br>○ Varies<br>○ Don't know		

References:

1. Demicheli V, Rivetti A, Debalini MG, Di Pietrantonj C. Vaccines for measles, mumps and rubella in children. Cochrane Database Syst Rev. 2012 Feb 15;(2):CD004407.
2. Anonymous. Vaccination against measles: clinical trial of live measles vaccine given alone and live vaccine preceded by killed vaccine. Second report to the medical research council by the measles vaccines committee. BMJ 1968;449-454.

3. Beasley RP, Detels R, Kim KS, Gale JL, Lin TL, Grayston JT. Prevention of rubella during an epidemic on Taiwan. HPV-77 and RA 27-3 rubella vaccines administered subcutaneously and intranasally HPV-77 vaccine mixed with mumps and-or measles vaccines. Am J Dis Child. 1969 Aug;118(2):301-6.
4. Croce E, Hatz C, Jonker EF, Visser LG, Jaeger VK, Bühler S. Safety of live vaccinations on immunosuppressive therapy in patients with immune-mediated inflammatory diseases, solid organ transplantation or after bone-marrow transplantation - A systematic review of randomized trials, observational studies and case reports. Vaccine. 2017 Mar 1;35(9):1216-1226.
5. Caldera F, Misch EA, Saha S, Wald A, Zhang Y, Hubers J, Megna B, Ley D, Reichelderfer M, Hayney MS. Immunosuppression Does Not Affect Antibody Concentrations to Measles, Mumps, and Rubella in Patients with Inflammatory Bowel Disease. Dig Dis Sci. 2019 Jan;64(1):189-195.
6. deBruyn JCC, Soon IS, Fonseca K, Feng S, Purtzki M, Goedhart C, Kuhn S, Vanderkooi OG, Wrobel I. Serologic Status of Routine Childhood Vaccines, Cytomegalovirus, and Epstein-Barr Virus in Children With Inflammatory Bowel Disease. Inflamm Bowel Dis. 2019 Jun 18;25(7):1218-1226.
7. Cleveland NK, Rodriquez D, Wichman A, Pan I, Melmed GY, Rubin DT. Many Inflammatory Bowel Disease Patients Are Not Immune to Measles or Pertussis. Dig Dis Sci. 2016 Oct;61(10):2972-2976.
8. Naganuma M, Nagahori M, Fujii T, Morio J, Saito E, Watanabe M. Poor recall of prior exposure to varicella zoster, rubella, measles, or mumps in patients with IBD. Inflamm Bowel Dis. 2013 Feb;19(2):418-22.
9. Abu-Elyazeed R, Jennings W, Severance R, Noss M, Caplanusi A, Povey M, Henry O. Immunogenicity and safety of a second dose of a measles-mumps-rubella vaccine administered to healthy participants 7 years of age or older: A phase III, randomized study. Hum Vaccin Immunother. 2018;14(11):2624-2631.
10. Wichmann A, Krugliak Cleveland N, Rubin DT. Safety and Efficacy of Live Measles Vaccine Administered to a Crohn's Disease Patient Receiving Vedolizumab. Am J Gastroenterol. 2016 Apr;111(4):577.

Conclusion - Adults

PICO 5A: In MMR-susceptible adult patients with IBD not on immunosuppressive therapy, should vaccination vs. no vaccination against mumps, measles, rubella (MMR) be given?
Moderate certainty of evidence
Direction – Yes (100%)
Strength – Strong (100%)

Type of recommendation	Strong recommendation against the intervention	Conditional recommendation against the intervention	Conditional recommendation for either the intervention or the comparison	Conditional recommendation for the intervention	Strong recommendation for the intervention

	○	○	○	○	○

**Recommendation**	**Statement 5A: In MMR-susceptible adult patients with IBD not on immunosuppressive therapy, we recommend MMR vaccine be given.**
**Justification**	
**Subgroup considerations**	• Contraindicated in pregnancy – theoretical risk to the fetus. Only smallpox vaccine has been shown to cause fetal injury. However, since the theoretical possibility exists, live vaccines should not be administered to women known to be pregnant.<br>• The severity of immunosuppression likely varies with severity or activity of disease, nutritional status, comorbidities, types and dosages of medications used.
**Implementation considerations**	
**Monitoring and evaluation**	• Ongoing monitoring of serious adverse events associated with MMR vaccines in IBD patients
**Research priorities**	• Observational studies to determine the risks of MMR infection in adult IBD patients compared to the general population<br>• RCTs or observational studies to determine the clinical effectiveness and immunogenicity of MMR vaccines in adult IBD patients with assessment of patient-important outcomes (i.e. MMR infection)

PICO 5B: In MMR-susceptible adult patients with IBD on immunosuppressive therapy, should vaccination vs. no vaccination against mumps, measles, rubella (MMR) be given?
Very low certainty evidence
Direction – No (100%)
Strength – conditional

Type of recommendation	Strong recommendation against the intervention	Conditional recommendation against the intervention	Conditional recommendation for either the intervention or the comparison	Conditional recommendation for the intervention	Strong recommendation for the intervention
	○	○	○	○	○

**Recommendation**	**Statement 5B: In measles-susceptible adult patients with IBD on immunosuppressive therapy, we suggest against giving MMR vaccine.**
**Justification**	
**Subgroup considerations**	• Contraindicated in pregnancy – theoretical risk to the fetus. Only smallpox vaccine has been shown to cause fetal injury. However, since the theoretical possibility exists, live vaccines should not be administered to women known to be pregnant.<br>• The severity of immunosuppression likely varies with severity or activity of disease, nutritional status, comorbidities, types and dosages of medications used.
**Implementation considerations**	
**Monitoring and evaluation**	• Ongoing monitoring of serious adverse events associated with MMR vaccines in IBD patients
**Research priorities**	• Observational studies to determine the risks of MMR infection in adult IBD patients compared to the general population<br>• RCTs or observational studies to determine the clinical effectiveness and immunogenicity of MMR vaccines in adult IBD patients with assessment of patient-important outcomes (i.e. MMR infection)

Varicella

Background

Primary varicella infection (chickenpox) is an acute infectious disease caused by the herpesvirus varicella zoster virus (VZV). Reactivation of latent infection is known as herpes zoster or shingles. VZV enters via the respiratory tract and conjunctiva and replicates in the nasopharynx and local lymph nodes. A primary viremia follows 4-6 days later which disseminates the virus to other organs such as the liver, spleen and sensory ganglia. Further replication occurs in the viscera, which is followed by a second viremia with involvement of the skin and the typical vesicular rash that is the hallmark of chickenpox. Breakthrough varicella is defined as a case of varicella due to infection with wild-type VZV occurring more than 42 days after varicella vaccination. Varicella in vaccinated persons is typically less severe and of a shorter duration than in unvaccinated persons.

The clinical course in children is usually mild and characterized by malaise, rash, pruritis and fever. Adults may have more severe disease and a higher rate of complications. Immunocompromised individuals may also have a more severe, prolonged illness. Acute varicella is typically self-limited, but it may be associated with complications. Secondary bacterial infection of skin lesions; pneumonia (viral or secondary bacterial); central nervous system disease ranging from aseptic meningitis to encephalitis and cerebella ataxia are more commonly seen. Rarer complications include transverse myelitis, Guillain-Barré syndrome, thrombocytopenia, hemorrhagic varicella, purpura fulminans, glomerulonephritis, myocarditis, arthritis, orchitis, uveitis, iritis, and hepatitis.

The risk of complications varies with age, occurring more frequently in patients over 15 years of age or less than 1 year of age. Complications are infrequent among healthy children. Since introduction of the varicella vaccination program in the US, varicella morbidity (cases and hospitalizations) and mortality has decreased by more than 90%.[1] Prior to the introduction of varicella vaccination, the fatality rates for varicella were approximately 1 per 100,000 cases among children 1-14 years of age, 2.7 per 100,000 cases among persons 15-19 years of age, and 25.2 per 100,000 cases among adults 30-49 years of age.[1] Adults accounted for only 5% of reported cases of varicella but approximately 35% of mortality.[1] Immunocompromised patients without evidence of immunity to varicella, such as people with leukemia or lymphoma, people on medications that suppress the immune system, such as high-dose systemic steroids or chemotherapeutic agents, and people with cellular immune-deficiencies or other immune system problems have a higher risk of severe varicella with disseminated disease and multi-organ involvement.[1]

VZV vaccine is a live-attenuated viral vaccine derived from the Oka strain of VZV. In the US, two VZV vaccines are licensed for use: a single antigen varicella vaccine (Varivax®) and a combination measles-mumps-rubella-varicella (MMRV) vaccine (ProQuad®). In Canada, four VZV vaccines are available for use: two single antigen varicella vaccines (Varivax®III and Varilrix®) and two combination MMRV vaccine (ProQuad™ and Priorix-Tetra®). The VZV vaccine is approved for children 12 months and older whilst the combined MMRV is approved for children 12 months to 12 years. However, MMRV is not approved for individuals older than 12.

Both CDC ACIP and NACI recommend two 0.5mL doses of VZV vaccine administered subcutaneously for children aged >12 months, adolescents, and adults (<50 years) without evidence of immunity.[1,2] As per NACI, evidence of immunity to varicella includes documented evidence of immunization with 2 doses of varicella-containing vaccine or laboratory evidence of immunity. Additionally in the US, evidence of immunity may also include birth in the US before 1980 (except in healthcare personnel, pregnant women, and immunocompromised people) and diagnosis of a history of varicella or herpes zoster by a healthcare provider.[1] In healthy children 12 months to 12 years of age, a single univalent varicella vaccine results in a seroconversion rate of 98% at 4 to 6 weeks after vaccination, with antibodies persisting in 98% at 5 years and 96% at 7 years after vaccination.[2] A second dose of a univalent varicella vaccine in children produces an improved immunologic response that is correlated with improved protection.[2] In adults and adolescents 13 years of age and older, 2 vaccine doses administered 4 to 8 weeks apart result in seroconversion rates of 99% at 4 to 6 weeks after the second dose, with persistence of antibodies 5 years later in 97% of vaccine recipients.[2] The estimated vaccine effectiveness 10 years following the receipt of 2 doses of univalent varicella vaccine is estimated at over 98% against any varicella disease and 100% against severe varicella.[2]

Serologic testing prior to or post- immunization is not recommended for healthy individuals. Commercially available varicella antibody tests (ELISA and LA) can be used to assess disease-induced immunity, but they lack sensitivity to detect antibody after vaccination. Previously vaccinated individuals who are inadvertently tested are likely to be immune to varicella, even if there is no detectable antibody.

As per NACI, individuals with autoimmune disease not treated with immunosuppressed drugs are not considered significantly immunocompromised and should receive varicella immunization.[2] Individuals who are immunocompromised, either due to underlying conditions or immunosuppressive agents, are more susceptible to infections including varicella. They may be more likely to experience more severe disease and complications. The safety and effectiveness of varicella vaccine is determined by the type of immunodeficiency and degree of immunosuppression.[2] **In general, immunocompromised people should not receive live vaccines because of the risk of disease caused by the vaccine strains**.[2] **As per CDC, people receiving prolonged, high dose systemic immunosuppressive therapy (≥ 2 weeks), including large doses of oral steroids (> 2mg/kg body weight or a total of 20mg/day of**

prednisone or its equivalent for people who weigh > 10 kg) should not receive varicella vaccine.[1] As well, people with "moderate or severe concurrent illness" should not receive varicella vaccine.[1]

ACG recommends adults with IBD should be assessed for prior exposure to varicella and vaccinated if naive before initiation of immunosuppressive therapy when possible (conditional recommendation, very low level of evidence).[3] The European Crohn's and Colitis Organization (ECCO) recommends susceptible IBD patients should be vaccinated with a 2-dose course at least 3 weeks prior to commencing immunomodulator therapy. Subsequent immunisation can only be administered after a 3–6 month cessation of all immunosuppressive therapy.[4] **IDSA recommends varicella be administered to patients with chronic inflammatory diseases without evidence of varicella immunity ≥ 4 weeks prior to initiation of immunosuppression if treatment initiation can be safely delayed**.[5] As well, **varicella vaccine should be considered for patients without evidence of immunity being treated for chronic inflammatory diseases with long-term, low-level immunosuppression (weak recommendation, very low quality evidence)**.[5] In the recently published vaccination guidelines for patients with immune-mediated disorders on immunosuppressive therapies, it was suggested that live attenuated vaccines be administered when individual benefits outweigh the perceived risks (conditional recommendation, low level evidence).[6]

References:

1. Centers for Disease Control and Prevention. https://www.cdc.gov/flu/professionals/vaccination/index.htm
2. Public Health Agency of Canada. https://www.canada.ca/en/public-health/services/publications/healthy-living/canadian-immunization-guide-part-4-active-vaccines/page-24-varicella-chickenpox-vaccine.html#p4c23a6e
3. Farraye FA, Melmed GY, Lichtenstein GR, Kane SV. ACG Clinical Guideline: Preventive Care in Inflammatory Bowel Disease. Am J Gastroenterol. 2017 Feb;112(2):241-258.
4. Rahier JF, Magro F, Abreu C, Armuzzi A, Ben-Horin S, Chowers Y, Cottone M, de Ridder L, Doherty G, Ehehalt R, Esteve M, Katsanos K, Lees CW, Macmahon E, Moreels T, Reinisch W, Tilg H, Tremblay L, Veereman-Wauters G, Viget N, Yazdanpanah Y, Eliakim R, Colombel JF; European Crohn's and Colitis Organisation (ECCO). Second European evidence-based consensus on the prevention, diagnosis and management of opportunistic infections in inflammatory bowel disease. J Crohns Colitis. 2014 Jun;8(6):443-68.
5. Rubin LG, Levin MJ, Ljungman P, Davies EG, Avery R, Tomblyn M, Bousvaros A, Dhanireddy S, Sung L, Keyserling H, Kang I; Infectious Diseases Society of America. 2013 IDSA clinical practice guideline for vaccination of the immunocompromised host. Clin Infect Dis. 2014 Feb;58(3):e44-100.
6. Papp KA, Haraoui B, Kumar D, Marshall JK, Bissonnette R, Bitton A, Bressler B, Gooderham M, Ho V, Jamal S, Pope JE, Steinhart AH, Vinh DC, Wade J. Vaccination Guidelines for Patients With Immune-Mediated Disorders on Immunosuppressive Therapies. J Cutan Med Surg. 2019 Jan/Feb;23(1):50-74.

Risk of Varicella infection in IBD patients

PICO: What is the risk of varicella infection in people with IBD compared to people without IBD?

Summary

Adult

Varicella zoster virus (VZV) predominantly affects children in temperate countries, with near-universal seroconversion occurring by late childhood.[1,2] However, in tropical regions, VZV infection is a less common childhood infection, and up to 50% of adults in these areas may have no history of primary infection.[3,4] Unfortunately, primary VZV infection is often more severe in adults than in children. In contrast to primary VZV infection, reactivation of the VZV (herpes zoster - HZ) tend to occur mainly in older adults (age > 50) and in those who are immunosuppressed.

Literature search did not identify any study on the risk of primary varicella infection in adult IBD patients. There are a number of case reports of primary varicella infection in immunosuppressed IBD patients, with severe disease course and fatalities reported. In a review article by Cullen et al in 2012, there were 20 reported cases of primary varicella infection IBD patients with five deaths.[5] Sixteen of the reported cases occurred in individuals age ≥ 18, with 3 cases resulting in death.[5] Thirteen of the cases involved organs other than the skin.[5] Nine of the 20 cases involved anti-TNF therapy; seven of these 9 were on combination immunosuppression.[5] Thirteen patients were on steroids and 12 were on either a thiopurine or methotrexate.[5]

For the risk of HZ in IBD patients, please see under Herpes Zoster.

Pediatric

One observational study addressed this PICO question.[6] A retrospective cross-sectional inpatient study in the US used an inpatient database of pediatric hospitalizations representing approximately 95.6% of all US pediatric hospitalizations. Using ICD-9 codes, cases of VZV and HSV were identified as the coded primary diagnosis in hospitalizations. IBD cases were identified using IBD (Crohn Disease and Ulcerative Colitis) as the secondary diagnoses. These groups were compared to a non-IBD cohort as well as to a third group of children with immunocompromising conditions (secondary diagnosis of malignancy, HIV, or a disorder of immunity). After adjusting for ethnicity, age, sex, geographic region and location and payer type, the authors found a strong association between IBD and VZV and HZ hospitalizations. Children and adolescents with IBD accounted for an increasing proportion of VZV-related hospitalizations during the study period 1997-2012 despite a decreasing temporal trend in VZV hospitalizations amongst all groups studied.

The GRADE rating started as high as this was considered a prognostic study (providing evidence about the likelihood of VZV infection in patients with IBD). The rating was downgraded to **very low** due to study limitations (detection and admission bias, residual confounding factors, misclassification bias) and indirectness (admitted IBD patients with a primary diagnosis of VZV infection and not all IBD patients). In particular, patients with IBD may be more likely to be diagnosed and admitted due to VZV or HZ than non-IBD controls thus creating an overestimate of the prevalence of VZV and HZ in IBD patients. **Overall, there is very low certainty evidence that pediatric patients with IBD are at higher risk of primary varicella infection or herpes zoster compared to the general population.**

Risk of Bias Table

Prognostic studies								
**Study**	**Study sample adequately represents the population of interest**	**Study data available adequately represent the study sample (>80% follow-up)**	**Prognostic factor measured in a similar and valid way for all participants**	**Outcome of interest is measured in a similar and valid way for all participants**	**Important potential confounding factors are appropriately accounted for**	**Statistical analysis is appropriate, and all primary outcomes are reported**	**Comments**
Adams 2016 (US) Pediatric	Cases were selected from the triennial Healthcare Cost and Utilization Project Kids' Inpatient Database, an inpatient database of pediatric hospitalizations in the US with data available from the years 1997, 2000, 2003, 2006, 2009, and 2012. The database now represents approximately 95.6% of all US pediatric hospitalization<br><br>Study included only	OK	Data were reliant on administrative claim codes. Possible misclassification errors due to errors of miscoding, and the codes have not been previously validated.	Data were reliant on administrative claim codes. Possible misclassification errors due to errors of miscoding, and the codes have not been previously validated.<br><br>Detection bias and admission rate bias: patients with IBD and skin rash may be more likely to be tested and admitted for VZV or HZ than controls, thus creating an overestimate of the prevalence of VZV or HZ among admitted IBD	Adjusted for age, sex, race/ethnicity, payer status, and geographic region.<br><br>Residual confounding: Did not adjust for medication exposure or dosing, disease activity or duration, or healthcare utilization. Immunization rates or status of children not able to de determined from claims database.	OK	• Retrospective, cross-sectional inpatient study (1997-2012)<br>• Cases were identified as patients 5-20 years of age who had a primary diagnosis of VZV (4434) or HSV (4,488) and a secondary diagnosis of IBD (CD 19,920; UC 11,367).<br>• VZV-related hospitalizations were compared to 2 control groups; children without IBD, and to children with immunocompromising conditions (535,147) defined as those hospitalized with a secondary diagnosis of malignancy, HIV, or a disorder of immunity.<br>• CD is associated with hospitalization for varicella (OR 12.75, 95%

	hospitalized patients, and did not capture patients treated as outpatients. Prevalence-incidence (Neyman) bias: Exclusion of individuals with severe (fatal prior to admission) or mild disease (not requiring admission) may result in a systematic error in the estimated association or effect of IBD on the risk of hospitalization for VZV or Zoster.			patients. As well, IBD patients may be preferentially hospitalized out of concern for their immunocompromising conditions and potential for serious complications. This is supported by the finding that there were no deaths in the IBD group, and they had a similar LOS to those without IBD (i.e. disease course did not appear to be more severe than children without IBD)			CI 8.30-19.59) and HZ (OR 7.91, 95% CI 5.6-11.18).<br>• UC is associated with hospitalization for varicella (OR 4.25, 95% CI 1.98-9.12) and HZ (OR 3.90, 95% CI 1.98-7.67).<br>• No deaths among children with IBD hospitalized for varicella or HZ.<br>• 9 deaths among children without IBD or an immunocompromising condition

Evidence Profile Table

Risk of Varicella and Zoster in Pediatric Patients with IBD

Certainty Assessment								Summary of Findings	
Studies	Risk of bias	Inconsistency	Indirectness	Imprecision	Other considerations	Certainty of Evidence	Overall Certainty of evidence	Study Event Rates	Relative Effect (95% CI)
**VPI (Mortality) - Critical**							⊕⊖⊖⊖ VERY LOW		
1 Retrospective Cross Sectional Study[6] (Pediatric)	Vey Serious[a]	Not Serious	Serious[b]	Not serious	None	⊕⊖⊖⊖ VERY LOW		0 out of 31,287 hospitalizations for VZV / HZ infection and IBD	
**VPI (Hospitalization for Varicella) - Critical**									
1 Retrospective Cross Sectional Study[6] (Pediatric)	Vey Serious[a]	Not Serious	Serious[b]	Not serious	None	⊕⊖⊖⊖ VERY LOW		CD: AOR 12.75 (8.30-19.59)<br>UC: AOR 4.25 (1.98-9.12)	
**VPI (Hospitalization for Zoster) – Critical**									
1 Retrospective Cross Sectional Study[6] (Pediatric)	Vey Serious[a]	Not Serious	Serious[b]	Not serious	None	⊕⊖⊖⊖ VERY LOW		CD: AOR 7.91 (5.60-11.18)<br>UC: AOR 3.90 (1.98-7.67)	

Footnotes:

a. Downgraded 2 levels due to study limitations: Detection and admission bias: patients with IBD and skin rash may be more likely to be tested and admitted for VZV or HZ than controls, thus creating an overestimate of the prevalence of VZV or HZ among admitted IBD patients. As well, IBD patients may be preferentially hospitalized out of concern for their immunocompromising conditions and potential for serious complications. This is supported by the finding that there were no deaths in the IBD group, and they had a similar LOS to those without IBD (i.e. disease course did not appear to be more severe than children without IBD). Residual confounding factors such as medication use (immunomodulators or biologics), varicella vaccination status, IBD disease activity, and healthcare utilization which may over-estimated the risk of hospitalization for VZV or HZ in IBD patients compared to controls). Data were based on discharge diagnostic codes. At risk of misclassification bias due to errors in coding and the use of non-validated codes.

b. Downgraded due to indirectness. Study included only a highly selected population (hospitalized patients), and did not capture VZV or HZ infection treated as outpatients. Hence, the risk of VZV or HZ among all IBD patients (population of interest) vs. non-IBD patients is unknown.

References:
1. Arvin AM. Varicella-zoster virus. Clin Microbiol Rev. 1996 Jul;9(3):361-81.
2. Bollaerts K, Riera-Montes M, Heininger U, Hens N, Souverain A, Verstraeten T, Hartwig S. A systematic review of varicella seroprevalence in European countries before universal childhood immunization: deriving incidence from seroprevalence data. Epidemiol Infect. 2017 Oct;145(13):2666-2677.
3. Hussey H, Abdullahi L, Collins J, Muloiwa R, Hussey G, Kagina B. Varicella zoster virus-associated morbidity and mortality in Africa - a systematic review. BMC Infect Dis. 2017 Nov 14;17(1):717.
4. Fatha N, Ang LW, Goh KT. Changing seroprevalence of varicella zoster virus infection in a tropical city state, Singapore. Int J Infect Dis. 2014 May;22:73-7.
5. Cullen G, Baden RP, Cheifetz AS. Varicella zoster virus infection in inflammatory bowel disease. Inflamm Bowel Dis. 2012 Dec;18(12):2392-403.
6. Adams DJ, Nylund CM. Hospitalization for Varicella and Zoster in Children with Inflammatory Bowel Disease. J Pediatr. 2016 Apr;171:140-5.

Effectiveness and Safety of Varicella vaccine in IBD patients

Summary - Pediatric

**PICO 6A**	**In varicella-susceptible pediatric patients with IBD not on immunosuppressive therapy, should vaccination vs. no vaccination against varicella (chickenpox) be given?**
**Population**	**Varicella-susceptible pediatric patients with IBD not on immunosuppressive therapy**
**Intervention**	Vaccination against varicella
**Comparator**	No vaccination against varicella
**Outcome**	Mortality, VPI (varicella infection), SAEs, Immunogenicity

**PICO 6B**	**In varicella-susceptible pediatric patients with IBD on immunosuppressive therapy, should vaccination vs. no vaccination against varicella (chickenpox) be given?**
**Population**	**Varicella-susceptible pediatric patients with IBD on immunosuppressive therapy**
**Intervention**	Vaccination against varicella

**Comparator**	No vaccination against varicella
**Outcome**	Mortality, VPI (varicella infection), SAEs, Immunogenicity

There was no RCT comparing varicella vaccines with placebo in patients with IBD to address this PICO question.

The World Health Organization (WHO) assessed the evidence on the effectiveness of varicella vaccines in healthy children in 2013. The WHO evidence profile included 3 systematic reviews.[1-3] One other systematic review was published recently in 2016.[4] In the most recent systematic review, 42 observational studies were included.[4] The pooled 1-dose vaccine was moderately effective in preventing all varicella with pooled vaccine effectiveness (VE) of 81% (95% CI 78-84%) and highly effective in preventing moderate / severe varicella with VE of 98% (95% CI 97-99%) in healthy children.[4] The second dose adds improved protection against all varicella with pooled VE of 92% (88-95%) in healthy children.[4] Overall, the WHO rated the quality of evidence for varicella vaccination in immunocompetent healthy children (9 months to 12 years of age) in preventing severe varicella to be **high.**

One systematic review included 40 observational studies (mostly cohort studies and case series/reports) in patients with immune-mediated diseases (2852/20,556 were IBD patients) on 22 different immunosuppressive medications.[5] The immunosuppressive medications used by IMID patients included prednisone 2.5-35mg/day, methotrexate 5-27mg/week, 6MP, biologic monotherapy, and combination therapy with biologic and immunomodulator.[5] The seroconversion rates in IMID group were high with varicella vaccines.[5] However, methotrexate and anti-TNF therapy appeared to reduce the seroconversion rates.[5] No subgroup data was provided for IBD patients.[5] Two cross-sectional studies assessed the serologic status of pediatric IBD patients (one at diagnosis of IBD, and one after diagnosis of IBD with the majority of patients exposed to immunosuppressive mediations).[6,7] Among pediatric IBD patients with a history of varicella vaccination or chickenpox infection, about 70% demonstrated serologic protection.[6,7] In one study, pediatric IBD patients with varicella vaccination were less likely to mount serologic protection compared to those with past chickenpox infection (32.8% vs. 95.2%, $P < 0.001$).[6] It is important to note that these 2 cross-sectional studies cannot distinguish between waning titers over time vs. primary vaccination failure. As well, the commercially available varicella antibody tests (ELISA and LA) can be used to assess disease-induced immunity, but they lack sensitivity to detect antibody after vaccination. As per CDC, previously vaccinated individuals who are tested are likely to be immune to varicella, even if there is no detectable antibody. The GRADE rating started as low due to observational nature of these studies. The evidence was further downgraded to **very low** due to study limitations (residual confounding and selection bias) and indirectness (surrogate outcomes).

The certainty of evidence for effectiveness was anchored to the general population (healthy children), and started as high. When the evidence was applied to IBD patients, the evidence was downgraded to **<u>moderate</u>** due to indirectness as observational studies

suggested that varicella vaccines may be less immunogenic in IBD patients. In summary, there is **<u>moderate</u>** certainty evidence that varicella vaccines are effective in pediatric IBD patients.

The WHO also assessed the evidence for safety of varicella vaccines in healthy children in 2013. The WHO evidence profile included 7 RCTs and 5 observational studies.[8-19] There were few reports and low incidence of serious adverse events in RCTs, observational studies and post-marketing surveillance data.[8-19] The certainty of evidence was rated as moderate due to imprecision.

One systematic review included 40 observational studies (mostly cohort studies and case series/reports) in patients with immune-mediated diseases (2852/20,556 were IBD patients) on 22 different immunosuppressive medications.[5] The administration of live vaccines was safe in most studies of IMID patients on immunosuppressive medications (including prednisone 2.5-35mg/day, methotrexate 5-27mg/week, 6MP, biologic monotherapy, and combination therapy with biologic and immunomodulator). Serious adverse events were rare (0.05%, 11/20,556 IMID patients). Infections through the vaccine strain were also rare, occuring in 0.06% (12/20,556 IMID patients). In most cases, infection was mild. However, two patients had fatal infection: a patient with RA/SLE overlap who started methotrexate/dexamethasone treatment 4 days after the yellow fever vaccine developed vaccine-associated viscerotropic disease and died. One infant whose mother was under infliximab treatment during pregnancy received the BCG vaccine at the age of 3 months and developed disseminated BCG infection and died. The certainty of evidence started as low due to the observational designs of these studies. The evidence was downgraded to **very low** due to study limitations and indirectness (patient population).

A large safety analysis was just published (outside of our literature review parameters) containing 22 years of post-marketing adverse event data.[20] Spontaneous, voluntary reporting of adverse events and non-interventional study reports submitted by health care providers was the basis for the review which spanned 1995 – 2017. During this time, >212 million doses of varicella vaccines were distributed globally. Reported rates were calculated based on total doses distributed and the assumption that each patient received 1 dose of the vaccine. Disseminated disease caused by the vaccine strain was confirmed by PCR in 39 cases.[20] 28 cases occurred in immunocompromised individuals and/or who reported concomitant use of immunosuppressive therapies (including patients with rheumatoid arthritis on prednisone and Methotrexate, SLE on pulse steroids, IBD with protein losing enteropathy and hypogammaglobulinemia on multiple immunosuppressive therapy). 86 cases of death (0.002%) were reported after vaccination with 26 occurring in immunocompromised patients (congenital syndromes, malignancies, OTC deficiency, HIV/AIDS; no reported fatal case in IBD patients).[20] It should be noted that these events were temporally associated with varicella vaccination, but may not have been causally associated. 25% of reports contained insufficient data to establish the cause of death.[20]

The certainty of evidence for safety was anchored to the general population (healthy children), and started as **moderate**. When the evidence is applied to **pediatric IBD patients not on immunosuppressive medications**, the evidence was not downgraded for indirectness. However, the evidence was downgraded to **very low** due to serious indirectness when applied to **pediatric IBD patients on immunosuppressive medications**. Although the risk of vaccine-induced infection with varicella vaccines seemed to be very rare in patients with immune-mediated diseases (including IBD), fatal outcomes did occur following the administration of other live vaccines. As well, the small number of IBD patients on immunosuppressive medications included in the systematic review may not be large enough to detect rare adverse events.

Overall, there is moderate certainty evidence that varicella vaccine is safe and effective in pediatric IBD patients not on immunosuppressive medications. There is very low certainty evidence that varicella vaccine is safe and effective in pediatric IBD patients on immunosuppressive medications.

Vaccinations with live vaccine has the potential to revert to the original pathogenic form and to induce infection by uncontrolled replication, particularly in immunocompromised individuals. Serious infections with the vaccine strain and even deaths have occurred in patients with HIV, leukemia and inherited immunodeficiencies.[21,22] **Thus far, no fatal infection has been reported after vaccination with varicella vaccines in IBD patients. On the other hand, disseminated wild-type varicella infection has been reported in an IBD patient treated with anti-TNF therapy.**[23] The dilemma in an immunosuppressed patient on whether to administer a live vaccine is related to the risks of vaccine-preventable illnesses. On the other hand, the live vaccine itself may pose important risks to the immunosuppressed patient including vaccine-induced infection, vaccine-related side effects and exacerbation of the underlying disease. As well, the vaccines may be less effective in the setting of immunosuppression.

Risk of Bias Table – Pediatric and Adults

**SR of Observational Studies**		
**Study**	**Quality Assessment**	**Comments**
Croce (2017) IMID	• Risk of bias was judged to be high in the majority of studies	• SR of 64 articles (8 RCTs,39 observational studies and 17 case series / reports) on live vaccinations in patients with IMIDs or SOT on immunosuppressive therapy as well as BMT patients who received a live vaccine < 2 years after BMT.<br>• **40 studies were conducted in IMID patients (n = 20,556 with 2852 IBD) on 22 different IS medications**

		• Vaccines in IMID: YF (n = 8 adults, n = 1 children and adults), MMR (n = 1 adults, n = 5 children), **varicella (n = 3 adults, n = 9 children, n = 1 children and adults),** HZ (n = 8 adults), polio (n = 1 adult), BCG (n = 2 adults, n = 2 children), live typhoid (n = 1 adult), smallpox (n = 1 adult)<br>• **IS medications used by IMID patients: prednisone 2.5-35mg/day, MTX 5-27mg/week, biological monotherapy, biological + IM**<br>• **In most studies, the administration of live vaccines was safe.**<br>• SAEs were reported in 11 IMID patients under steroid treatment who received HZV (as frequent as the placebo group). No SAEs with MMR, polio, small pox, live typhoid, BCG.<br>• **12/20,556 IMID (0.06%) patients developed an infection through the vaccine strain**. In most cases, the infection was mild. However, 2 patients had fatal infection: RA/SLE on MTX/dexamethasone received YF. Infant whose mother was on IFX during treatment received BCG.<br>• **No increase flares of autoimmune disease** in most studies<br>• **High seroconversion rates in IMID group with VV** and YFV. **MTX and anti-TNF appeared to reduce immune response to VV and HZ,** but not to MMR and YF revaccination.<br>• Although live vaccines were safe and sufficiently immunogenic in most studies, some serious reactions and vaccine-related infections were reported in immunosuppressed IMID patients.<br>• Until further data are available, live vaccinations under most immunosuppressive treatments should only be administered after a careful risk benefit assessment<br>• **Included Lu 2010 – case series of 6 children of IBD receiving IS therapy (4 patient on 6MP +/- 5ASA, 1 patient on IFX monotherapy, 1 patient on IFX + 6MP), who were injected with varicella vaccine, no SAEs, and developed antibodies to the varicella.**

BMT – bone marrow transplantation
IFX - infliximab
IMID – immune-mediated inflammatory disease
IM - immunomodulators
IS – immunosuppressive medication
MTX - methotrexate
SOT – solid organ transplantation
VV – varicella vaccine

YF – yellow fever

Cohort studies							
**Study**	**Valid methods to ascertain exposure**	**Prognostic factors (other than exposure of interest) similar among cohorts – or cohorts were adjusted adequately for confounders**	**Demonstration that outcome of interest was not present at the start of the study**	**Outcome detection methods valid and similar among cohorts**	**Follow-up complete and similar among cohorts**	**Free of other bias**	**Comments**
deBruyn 2018 (Canada) Pediatric IBD	Vaccination records and baseline serology were used to determine immunity against vaccine preventable diseases including Varicella.	IBD subtype, current immunosuppressive medication use, age at diagnosis, and age at serum collection were adjusted for in a multivariate analysis.<br><br>Disease activity at time of vaccination, duration of disease and nutritional status were not accounted for.	OK	Commercially available varicella antibody tests (ELISA and LA) can be used to assess disease-induced immunity, but they lack sensitivity to detect antibody after vaccination. Previously vaccinated individuals who are tested are likely to be immune to varicella, even if there is no detectable antibody.	OK	Possible selection bias. Patients attending a tertiary referral center may differ systematically from other patients. Patients who agreed to participate in a study where serologic assays were measured may be different than patients who did not agree to participate.	• Cross sectional study in children examining the serologic status of childhood vaccinatable diseases<br>• 156 children with IBD at a Canadian tertiary referral IBD unit.<br>• **Among 146 subjects with a history of varicella vaccination or chickenpox infection**, **70.5% demonstrated serologic protection.**<br>• Current IS therapy, IBD type, age at diagnosis were not associated with serologic protection.<br>• Older age at serum collection was associated with serologic protection (OR 1.69, 95% CI 1.33-2.15). This may be due to a **higher proportion of subjects with past chickenpox infection mounting serologic proection compared with**

							**subjects with varicella vaccination (95.2% vs 32.8%, P < 0.001)**<br>• Subjects with past chickenpox infection mounted higher titers of varicella IgG than subjects with varicella vaccination (1693.8 IU/L vs. 61.6 IU/L, P < 0.0001)
Ansari 2011 (US) Pediatric IBD	Chart review of vaccination history, immunization records, vaccination titers at the time of diagnosis. Vaccination history and titers were routinely collected at the time of diagnosis of IBD.	No adjustment of any prognostic factors	OK	"Varicella-Zoster IgG ELISA has sensitivity of 96.3% and specificity of 94.4% for individuals who have had varicella disease. Performance characteristics with individuals vaccinated with varicella have not been established"	No information was available for 12% of patients	Possible selection bias. Patients attending a tertiary referral center may differ systematically from other patients.	• Retrospective cohort study of 163 newly diagnosed IBD patients, mean age 12 years (1-19 years)<br>• 66% had a history of varicella disease or had received at least 1 vaccination (52% had vaccination, 14% had disease)<br>• **77% of patients had measurable titers at the time of diagnosis.**

Before-After (Pre-Post) Studies									
**Study**	**Was there a <u>concurrent</u> comparator group that did not receive the**	**If a concurrent comparator group was used, was it <u>similar</u> to the**	**If <u>no</u> concurrent comparator group was used**		**Outcome detection methods valid and similar among**	**Incomplete outcome data assessed**	**Selective outcome reporting**	**Other bias**	**Comments**
			**If each participant served as**	**If two different consecutive cohorts of**					

	intervention	intervention group (or adequately adjusted) for prognostic factors	his/her own control (assessed before vs. after the intervention), are there compelling arguments that the outcome was not influenced by historic events / underlying secular trends	participants were assessed (before vs. after implementation of the intervention), are there (a) compelling arguments that the outcome was not influenced by historic events / underlying secular trends and (b) evidence that the two groups were similar (or adequately adjusted) for prognostic factors	compared groups / periods				
Kuter 1995 (US and Canada) Healthy adolescents and adults	No	NA	OK	NA	OK	OK	OK	Healthy vaccinee effect	• Cohort study of 757 healthy adolescents and adults 13-54 years<br>• Randomized 2 doses of varicella 4 vs. 8 weeks<br>• Seroconversion rates 72% and 99% vs. 78% and 99% after 1st and 2nd dose<br>• 2/757 (0.26%) patients developed varicella

									• 2/46 (4.3%) exposed developed varicella<br>• No serious adverse events
Gershon 1988 (US) Healthy varicella-susceptible adults	No	NA	OK	NA	OK	OK	OK	Healthy vaccinee effect	• Cohort study of 187 healthy varicella-susceptible adults (healthcare personnel or parents of young children)<br>• 81% received 2 doses live varicella vaccine, 18% 1 dose, 1% 3 doses<br>• Seroconversion 82% after 1st dose and 94% after 2nd doses<br>• Antibodies persist for at least 6 years in > 70%<br>• 12/187 (6.4%) mild chickenpox (mean 30 mos)<br>• **Vaccine efficacy was 51% assuming 90% attack rate in susceptible subjects**<br>• No serious adverse effects
Gershon 1986 (US) Healthy adults	No	NA	OK	NA	OK	OK	OK	Healthy vaccinee effect	• Cohort study of children with acute-lymphocytic leukemia in remission x 9 mos or 86 healthy adults (42 healthcare professionals)

									• 1-2 doses of varicella vaccine<br>• Seroconversion 58% after 1st dose and 92% after 2nd dose<br>• Mild varicella in 6/86 (7%) mean 20 mos<br>• No serious adverse effects
Ndumbe 1985 (UK) Adults Healthy Adults	No	NA	OK	NA	OK	OK	OK	Healthy vaccinee effect	• Cohort study 34 nurses with no previous history of chickenpox and seronegative to VZV<br>• Received 1 dose live varicella vaccine<br>• F/U 36 mos<br>• 94% seroconverted at 1 year, 64% detectable antibody at 3 years<br>• 2/34 (5.8%) developed chickenpox<br>• No major vaccine reactions

Evidence Profile Table - Pediatric

Effectiveness and Safety of Varicella Vaccine in Pediatric IBD Patients

Certainty Assessment								Summary of Findings	
Studies	Risk of bias	Inconsistency	Indirectness	Imprecision	Other considerations	Certainty of Evidence	Overall Certainty of evidence	Study Event Rates	Relative Effect (95% CI)
**VPI (Varicella infection) - CRITICAL**							⊕⊕⊕⊖ MODERATE Pediatric IBD patients not on IS<br><br>⊕⊕⊖⊖ VERY LOW Pediatric IBD patients on IS		
4 SR of 42 Observational Studies[1-4]<br><br>**Healthy Children**<br><br>*Adapted from WHO Evidence Profie Table*	Not serious	Not serious	Serious[a]	Not serious	Upgraded by 2 levels as strong evidence from observational studies of vaccine effectiveness of 80% or higher with no major residual confounders. In addition to effectiveness on an individual level, decline in incidence in all age groups over time, not only age-group targeted by vaccination programs, suggests induction of community protection.	⊕⊕⊕⊖ MODERATE		• Pooled 1-dose vaccine effectiveness 81% (78-84%) against all varicella and 98% (97-99%) against moderate/severe varicella in Healthy Children<br>• Pooled 2-dose vaccine effectiveness against all varicella was 92% (88-95%) in Healthy Children	
**Immunogenicity (Varicella antibody titer) - IMPORTANT**									
1 SR of 40 Observational Studies[5]<br><br>**20,556 IMID patients with 2852 IBD patients**	Serious[b]	Not serious	Serious[c]	Not serious	None	⊕⊕⊖⊖ VERY LOW		• High seroconversion rates against varicella vaccines in IMID patients on immunosuppressive medications<br>• MTX and anti-TNF appeared to reduce immune response to varicella vaccines.	
2 Observational Studies[6,7]	Serious[d]	Not serious	Serious[e]	Serious[f]	None	⊕⊕⊖⊖ VERY LOW		• Among pediatric IBD patients with a history of varicella vaccination or chickenpox infection, about 70% demonstrated serologic protection (? Waning titers vs.	

**Pediatric IBD populations**								primary vaccination failure)<br>• Lower proportion of pediatric IBD patients with varicella vaccination mounting serologic protection compared with pediatric IBD patients with past chickenpox infection (32.8% vs. 95.2%, P < 0.001)[6]
**Serious Adverse Events - CRITICAL**								
7 RCTs and 5 observational studies[8-19]<br><br>**Healthy Children**<br><br>*Adapted from WHO Evidence Profile Table*	Not serious	Not serious	Not serious[g]<br>Not on IS<br><br>Very serious[g]<br>On IS	Serious[h]	None	⊕⊕⊕⊖<br>MODERATE<br>Not on IS<br><br>⊕⊖⊖⊖<br>VERY LOW<br>On IS		• Few reports and low incidence of serious adverse events in RCTs, observational studies and post-marketing surveillance data.
1 SR of 40 Observational Studies[5]<br><br>**20,556 IMID patients with 2852 IBD patients**	Serious[b]	Not serious	Serious[c]	Not serious	None	⊕⊕⊖⊖<br>VERY LOW		• The administration of live vaccines was safe in most studies of IMID patients on immunosuppressive medications<br>• Serious adverse events were reported in 11/20,556 (0.05%) IMID patients<br>• Infection through the vaccine strain in 12/20,556 (0.06%) IMID patients. Infection was mild in most cases. However, 2 patients had fatal infection (yellow fever vaccine, BCG vaccine)<br>• No increase flares of autoimmune diseases in most studies

Footnotes:

a. Downgraded for indirectness. Patient population is healthy children (not IBD patients). Observational studies suggested that varicella vaccines may not be as immunogenic (and therefore less effective) as in healthy children.
b. Downgraded for study limitations. Residual confounding cannot be ruled out given the observational nature of these studies (e.g. comorbidities, concurrent illnesses, disease activity and duration, nutritional status, and other factors which may affect the risk of varicella infection) as well as selection bias.
c. Downgraded due to indirectness (population and surrogate outcomes). Only 14% (32852/20,556) IMID patients were patients with IBD. Surrogate outcomes were used for vaccine effectiveness.
d. Downgraded for study limitations. Residual confounding cannot be ruled out as disease activity at time of vaccination, duration of disease and nutritional status were not accounted for. Possible selection bias. Patients attending a tertiary referral center may differ systematically from other patients.
e. Downgraded due to indirectness (surrogate outcomes). The cross-sectional studies cannot distinguish between waning titers over time vs. primary vaccination failure. As well, the commercially available varicella antibody tests (ELISA and LA) can be used to assess disease-induced immunity, but they lack sensitivity to detect antibody after vaccination. Previously vaccinated individuals who are tested are likely to be immune to varicella, even if there is no detectable antibody.
f. Downgraded due to imprecision (small sample sizes).

g. Not downgraded for indirectness in pediatric IBD patients not on immunosuppressive. Downgraded for very serious indirectness in pediatric IBD patients on immunosuppressive medications (low risk of vaccine-induced infection, and very rare fatal outcomes with other live vaccines). Small number of IBD patients included in the systematic review, and sample size may not be large enough to detect rare adverse events.
h. Downgraded for imprecision. Small sample sizes to detect rare serious adverse events.

References:

1. World Health Organization. Systematic review of available evidence on effectiveness and duration of protection of varicella vaccines (through October 2013. http://www.who.int/immunization/sage/meetings/2014/april/4_Systematic_review_on_effectiveness_and_duration_of_protection_of_varicella_vaccines.pdf.
2. Seward JF, Marin M, Vázquez M. Varicella vaccine effectiveness in the US vaccination program: a review. J Infect Dis. 2008 Mar 1;197 Suppl 2:S82-9.
3. Bayer O, Heininger U, Heiligensetzer C, von Kries R. Metaanalysis of vaccine effectiveness in varicella outbreaks. Vaccine. 2007 Sep 17;25(37-38):6655-60.
4. Marin M, Marti M, Kambhampati A, Jeram SM, Seward JF. Global Varicella Vaccine Effectiveness: A Meta-analysis. Pediatrics. 2016 Mar;137(3):e20153741.
5. Croce E, Hatz C, Jonker EF, Visser LG, Jaeger VK, Bühler S. Safety of live vaccinations on immunosuppressive therapy in patients with immune-mediated inflammatory diseases, solid organ transplantation or after bone-marrow transplantation - A systematic review of randomized trials, observational studies and case reports. Vaccine. 2017 Mar 1;35(9):1216-1226.
6. deBruyn JCC, Soon IS, Fonseca K, Feng S, Purtzki M, Goedhart C, Kuhn S, Vanderkooi OG, Wrobel I. Serologic Status of Routine Childhood Vaccines, Cytomegalovirus, and Epstein-Barr Virus in Children With Inflammatory Bowel Disease. Inflamm Bowel Dis. 2019 Jun 18;25(7):1218-1226.
7. Ansari F, Baker RD, Patel R, Baker SS. Varicella immunity in inflammatory bowel disease. J Pediatr Gastroenterol Nutr. 2011 Oct;53(4):386-8.
8. Ferrera G, Gajdos V, Thomas S, Tran C, Fiquet A. Safety of a refrigerator-stable varicella vaccine (VARIVAX) in healthy 12- to 15-month-old children: A randomized, double-blind, cross-over study. Hum Vaccin. 2009 Jul;5(7):455-60.
9. Gatchalian S, Leboulleux D, Desauziers E, Bermal N, Borja-Tabora C. Immunogenicity and safety of a varicella vaccine, Okavax, and a trivalent measles, mumps and rubella vaccine, MMR-II, administered concomitantly in healthy Filipino children aged 12-24 months. Southeast Asian J Trop Med Public Health. 2003 Sep;34(3):589-97.
10. Lau YL, Vessey SJ, Chan IS, Lee TL, Huang LM, Lee CY, Lin TY, Lee BW, Kwan K, Kasim SM, Chan CY, Kaplan KM, Distefano DJ, Harmon AL, Golie A, Hartzel J, Xu J, Li S, Matthews H, Sadoff JC, Shaw A. A comparison of safety, tolerability and immunogenicity of Oka/Merck varicella vaccine and VARILRIX in healthy children. Vaccine. 2002 Jul 26;20(23-24):2942-9.
11. Parment PA, Svahn A, Rudén U, Bråkenhielm G, Storsaeter J, Akesson L, Linde A. Immunogenicity and reactogenicity of a single dose of live attenuated varicella vaccine and a booster dose of measles-mumps-rubella vaccine given concomitantly at 12 years of age. Scand J Infect Dis. 2003;35(10):736-42.
12. Ramkissoon A, Coovadia HM, Jugnundan P, Haffejee IE, Meurice F, Vandevoorde D. Immunogenicity and safety of a live attenuated varicella vaccine in healthy Indian children aged 9-24 months. S Afr Med J. 1995 Dec;85(12):1295-8.
13. Shinefield HR, Black SB, Staehle BO, Matthews H, Adelman T, Ensor K, Li S, Chan I, Heyse J, Waters M, Chan CY, Vessey SJ, Kaplan KM, Kuter BJ; Kaiser Permanente Medical Team for Varivax. Vaccination with measles, mumps and rubella vaccine and varicella vaccine: safety, tolerability, immunogenicity, persistence of antibody and duration of protection against varicella in healthy children. Pediatr Infect Dis J. 2002 Jun;21(6):555-61.

14. Weibel RE, Neff BJ, Kuter BJ, Guess HA, Rothenberger CA, Fitzgerald AJ, Connor KA, McLean AA, Hilleman MR, Buynak EB, et al. Live attenuated varicella virus vaccine. Efficacy trial in healthy children. N Engl J Med. 1984 May 31;310(22):1409-15.
15. Black S, Shinefield H, Ray P, Lewis E, Hansen J, Schwalbe J, Coplan P, Sharrar R, Guess H. Postmarketing evaluation of the safety and effectiveness of varicella vaccine. Pediatr Infect Dis J. 1999 Dec;18(12):1041-6.
16. Chaves SS, Haber P, Walton K, Wise RP, Izurieta HS, Schmid DS, Seward JF. Safety of varicella vaccine after licensure in the United States: experience from reports to the vaccine adverse event reporting system, 1995-2005. J Infect Dis. 2008 Mar 1;197 Suppl 2:S170-7.
17. Ozaki T, Nishimura N, Kajita Y. Experience with live attenuated varicella vaccine (Oka strain) in healthy Japanese subjects; 10-year survey at pediatric clinic. Vaccine. 2000 May 8;18(22):2375-80.
18. Galea SA, Sweet A, Beninger P, Steinberg SP, Larussa PS, Gershon AA, Sharrar RG. The safety profile of varicella vaccine: a 10-year review. J Infect Dis. 2008 Mar 1;197 Suppl 2:S165-9.
19. Sharrar RG, LaRussa P, Galea SA, Steinberg SP, Sweet AR, Keatley RM, Wells ME, Stephenson WP, Gershon AA. The postmarketing safety profile of varicella vaccine. Vaccine. 2000 Nov 22;19(7-8):916-23.
20. Woodward M, Marko A, Galea S, Eagel B, Straus W. Varicella Virus Vaccine Live: A 22-Year Review of Postmarketing Safety Data. Open Forum Infect Dis. 2019 Aug 1;6(8).
21. Alexander KE, Tong PL, Macartney K, Beresford R, Sheppeard V, Gupta M. Live zoster vaccination in an immunocompromised patient leading to death secondary to disseminated varicella zoster virus infection. Vaccine. 2018 Jun 22;36(27):3890-3893
22. Denham JD, Lee DH, Castro M, Pandya S, Aslam S, Nanjappa S, Greene JN. Two cases of disseminated infection following live organism anti-cancer vaccine administration in cancer patients. Int J Infect Dis. 2018 Jul;72:1-2.
23. Leung VS, Nguyen MT, Bush TM. Disseminated primary varicella after initiation of infliximab for Crohn's disease. Am J Gastroenterol. 2004 Dec;99(12):2503-4.

Evidence to Decision Table – Pediatric

**PICO 6A**	**In varicella-susceptible pediatric patients with IBD not on immunosuppressive therapy, should vaccination vs. no vaccination against varicella (chickenpox) be given?**
**Population**	**Varicella-susceptible pediatric patients with IBD not on immunosuppressive therapy**
**Intervention**	Vaccination against varicella
**Comparator**	No vaccination against varicella
**Outcome**	Mortality, VPI (varicella infection), SAEs, Immunogenicity

**PICO 6B**	**In varicella-susceptible pediatric patients with IBD on immunosuppressive**

	**therapy, should vaccination vs. no vaccination against varicella (chickenpox) be given?**
**Population**	**Varicella-susceptible pediatric patients with IBD on immunosuppressive therapy**
**Intervention**	Vaccination against varicella
**Comparator**	No vaccination against varicella
**Outcome**	Mortality, VPI (varicella infection), SAEs, Immunogenicity

	Judgement	Research evidence	Additional considerations
**Desirable Effects**	**How substantial are the desirable anticipated effects?**<br><br>○ Trivial<br>○ Small<br>○ Moderate<br>○ Large Not on and on IS<br><br>○ Varies<br>○ Don't know	**Risks of Varicella Infection in Pediatric IBD Patients**<br><br>One observational study addressed this PICO question.[1] A retrospective cross-sectional inpatient study in the US used an inpatient database of pediatric hospitalizations representing approximately 95.6% of all US pediatric hospitalizations.[1] Using ICD-9 codes, cases of VZV and HSV were identified as the coded primary diagnosis in hospitalizations. IBD cases were identified using IBD (Crohn Disease and Ulcerative Colitis) as the secondary diagnoses.[1] These groups were compared to a non-IBD cohort as well as to a third group of children with immunocompromising conditions (secondary diagnosis of malignancy, HIV, or a disorder of immunity). After adjusting for ethnicity, age, sex, geographic region and location and payer type, the authors found a strong association between IBD and VZV and HZ hospitalizations. Children and adolescents with IBD accounted for an increasing proportion of VZV-related hospitalizations during the study period 1997-2012 despite a decreasing temporal trend in VZV hospitalizations amongst all groups studied.[1]<br><br>The GRADE rating started as high as this was considered a prognostic study (providing evidence about the likelihood of VZV infection in patients with IBD). The rating was downgraded to **very low** due to study limitations (detection and admission bias, residual confounding factors, misclassification bias) and indirectness (admitted IBD patients with a primary diagnosis of VZV infection and not all IBD patients). In particular, patients with IBD may be more likely to be diagnosed and admitted due to VZV or HZ than non-IBD controls thus creating an overestimate of the prevalence of VZV and HZ in IBD patients. **Overall, there is very low certainty evidence that pediatric patients with IBD are at higher risk of primary varicella infection or herpes zoster compared to the general population.**<br><br><br>**Effectiveness and Safety of Varicella Vaccine in Pediatric IBD Patients**	
**Undesirable Effects**	**How substantial are the undesirable anticipated effects?**<br><br>○ Large<br>○ Moderate<br>○ Small On IS<br>○ Trivial Not on IS<br><br>○ Varies<br>○ Don't know		

		There was no RCT comparing varicella vaccines with placebo in patients with IBD to address this PICO question.<br><br>The World Health Organization (WHO) assessed the evidence on the effectiveness of varicella vaccines in healthy children in 2013. The WHO evidence profile included 3 systematic reviews.[2-4] One other systematic review was published recently in 2016.[5] In the most recent systematic review, 42 observational studies were included.[5] The pooled 1-dose vaccine was moderately effective in preventing all varicella with pooled vaccine effectiveness (VE) of 81% (95% CI 78-84%) and highly effective in preventing moderate / severe varicella with VE of 98% (95% CI 97-99%) in healthy children.[5] The second dose adds improved protection against all varicella with pooled VE of 92% (88-95%) in healthy children.[5] Overall, the WHO rated the quality of evidence for varicella vaccination in immunocompetent healthy children (9 months to 12 years of age) in preventing severe varicella to be high.<br><br>One systematic review included 40 observational studies (mostly cohort studies and case series/reports) in patients with immune-mediated diseases (2852/20,556 were IBD patients) on 22 different immunosuppressive medications.[6] The immunosuppressive medications used by IMID patients included prednisone 2.5-35mg/day, methotrexate 5-27mg/week, 6MP, biologic monotherapy, and combination therapy with biologic and immunomodulator.[6] The seroconversion rates in IMID group were high with varicella vaccines.[6] However, methotrexate and anti-TNF therapy appeared to reduce the seroconversion rates.[6] No subgroup data was provided for IBD patients.[6] Two cross-sectional studies assessed the serologic status of pediatric IBD patients (one at diagnosis of IBD, and one after diagnosis of IBD with the majority of patients exposed to immunosuppressive mediations).[7,8] Among pediatric IBD patients with a history of varicella vaccination or chickenpox infection, about 70% demonstrated serologic protection.[7,8] In one study, pediatric IBD patients with varicella vaccination were less likely to mount serologic protection compared to those with past chickenpox infection (32.8% vs. 95.2%, P < 0.001).[7] It is important to note that these 2 cross-sectional studies cannot distinguish between waning titers over time vs. primary vaccination failure. As well, the commercially available varicella antibody tests (ELISA and LA) can be used to assess disease-induced immunity, but they lack sensitivity to detect antibody after vaccination. As per CDC, previously vaccinated individuals who are tested are likely to be immune to varicella, even if there is no detectable antibody. The GRADE rating started as low due to observational nature of these studies. The evidence was further downgraded to **very low** due to study limitations (residual confounding and selection bias) and indirectness (surrogate outcomes).<br><br>**The certainty of evidence for effectiveness was anchored to the general population (healthy children**), and started as high. When the evidence was applied to IBD patients, the evidence was downgraded to **<u>moderate</u>** due to indirectness as observational studies suggested that varicella vaccines may be less immunogenic in IBD patients. In summary, there is **<u>moderate</u>** certainty evidence that varicella vaccines are effective in pediatric IBD patients.<br><br>The WHO also assessed the evidence for safety of varicella vaccines in healthy children	

		in 2013. The WHO evidence profile included 7 RCTs and 5 observational studies.[9-20] There were few reports and low incidence of serious adverse events in RCTs, observational studies and post-marketing surveillance data.[9-20] The certainty of evidence was rated as moderate due to imprecision.<br><br>One systematic review included 40 observational studies (mostly cohort studies and case series/reports) in patients with immune-mediated diseases (2852/20,556 were IBD patients) on 22 different immunosuppressive medications.[6] The administration of live vaccines was safe in most studies of IMID patients on immunosuppressive medications (including prednisone 2.5-35mg/day, methotrexate 5-27mg/week, 6MP, biologic monotherapy, and combination therapy with biologic and immunomodulator). Serious adverse events were rare (0.05%, 11/20,556 IMID patients). Infections through the vaccine strain were also rare, occuring in 0.06% (12/20,556 IMID patients). In most cases, infection was mild. However, two patients had fatal infection: a patient with RA/SLE overlap who started methotrexate/dexamethasone treatment 4 days after the yellow fever vaccine developed vaccine-associated viscerotropic disease and died. One infant whose mother was under infliximab treatment during pregnancy received the BCG vaccine at the age of 3 months and developed disseminated BCG infection and died. The certainty of evidence started as low due to the observational designs of these studies. The evidence was downgraded to **very low** due to study limitations and indirectness (patient population).<br><br>A large safety analysis was just published (outside of our literature review parameters) containing 22 years of post-marketing adverse event data.[21] Spontaneous, voluntary reporting of adverse events and non-interventional study reports submitted by health care providers was the basis for the review which spanned 1995 – 2017. During this time, >212 million doses of varicella vaccines were distributed globally. Reported rates were calculated based on total doses distributed and the assumption that each patient received 1 dose of the vaccine. Disseminated disease caused by the vaccine strain was confirmed by PCR in 39 cases.[21] 28 cases occurred in immunocompromised individuals and/or who reported concomitant use of immunosuppressive therapies (including patients with rheumatoid arthritis on prednisone and Methotrexate, SLE on pulse steroids, IBD with protein losing enteropathy and hypogammaglobulinemia on multiple immunosuppressive therapy). 86 cases of death (0.002%) were reported after vaccination with 26 occurring in immunocompromised patients (congenital syndromes, malignancies, OTC deficiency, HIV/AIDS; no reported fatal case in IBD patients).[21] It should be noted that these events were temporally associated with varicella vaccination, but may not have been causally associated. 25% of reports contained insufficient data to establish the cause of death.[21]<br><br>**The certainty of evidence for safety was anchored to the general population (healthy children)**, and started as **moderate**. When the evidence is applied to **pediatric IBD patients not on immunosuppressive medications**, the evidence was not downgraded for indirectness. However, the evidence was downgraded to **<u>very low</u>** due to serious indirectness when applied to **pediatric IBD patients on immunosuppressive medications**. Although the risk of vaccine-induced infection with varicella vaccines seemed to be very rare in patients with immune-mediated diseases (including IBD),	

		fatal outcomes did occur following the administration of other live vaccines. As well, the small number of IBD patients on immunosuppressive medications included in the systematic review may not be large enough to detect rare adverse events.<br><br>**Overall, there is moderate certainty evidence that varicella vaccine is safe and effective in pediatric IBD patients not on immunosuppressive medications. There is very low certainty evidence that varicella vaccine is safe and effective in pediatric IBD patients on immunosuppressive medications.**<br><br>Vaccinations with live vaccine has the potential to revert to the original pathogenic form and to induce infection by uncontrolled replication, particularly in immunocompromised individuals. Serious infections with the vaccine strain and even deaths have occurred in patients with HIV, leukemia and inherited immunodeficiencies.[22,23] **Thus far, no fatal infection has been reported after vaccination with varicella vaccines in IBD patients. On the other hand, disseminated wild-type varicella infection has been reported in an IBD patient treated with anti-TNF therapy.**[23] The dilemma in an immunosuppressed patient on whether to administer a live vaccine is related to the risks of vaccine-preventable illnesses. On the other hand, the live vaccine itself may pose important risks to the immunosuppressed patient including vaccine-induced infection, vaccine-related side effects and exacerbation of the underlying disease. As well, the vaccines may be less effective in the setting of immunosuppression.	
**Certainty of evidence**	**What is the overall certainty of the evidence of effects?**<br><br>○ Very low IBD patients on IS<br>○ Low<br>○ Moderate IBD patients not on IS<br>○ High<br><br>○ No included studies		
**Values and Preferences**	**Is there important uncertainty about or variability in how much people value the main outcomes?**<br><br>○ Important uncertainty or variability<br>○ Possibly important uncertainty or variability<br>○ Probably no important uncertainty or variability<br>○ No important uncertainty or variability	Patients likely value patient-important outcomes (mortality, VPI, adverse effects) more than surrogate outcomes (immunogenicity).	

<table>
<tr>
<td>Balance of effects</td>
<td>Does the balance between desirable and undesirable effects favor the intervention or the comparison?

o Favors the comparison
o Probably favors the comparison
o Does not favor either the intervention or the comparison
o Probably favors the intervention on IS
o Favors the intervention Not on IS

o Varies
o Don't know</td>
<td></td>
<td></td>
</tr>
<tr>
<td>Resources required</td>
<td>How large are the resource requirements (costs)?

o Large costs
o Moderate costs
o Negligible costs and savings
o Moderate savings
o Large savings

o Varies
o Don't know</td>
<td>CDC vaccine price list last reviewed/updated: July 1, 2019

<table><tr><th>Brandname</th><th>CDC cost/dose</th><th>Private sector cost/dose</th></tr><tr><td>Varivax®</td><td>$104.09 (children)

$84.88 (adults)</td><td>$135.73</td></tr></table>
Varivax is given as a 2-dose vaccine.</td>
<td></td>
</tr>
<tr>
<td>Certainty of Evidence of Required Resources</td>
<td>What is the certainty of the evidence of resource requirements (costs)?

o Very low
o Low
o Moderate
o High

o No included studies</td>
<td>The costs of delivering routine immunization services may vary widely across countries and different health system settings. See Immunization Costing Action Network (ICAN) Immunization Delivery Cost Catalogue. http://immunizationeconomics.org/ican-idcc.</td>
<td></td>
</tr>
</table>

**Cost effectiveness**	**Does the cost-effectiveness of the intervention favor the intervention or the comparison?**<br><br>○ Favors the comparison<br>○ Probably favors the comparison<br>○ Does not favor either the intervention or the comparison<br>○ Probably favors the intervention Not on IS<br>○ Favors the intervention<br><br>○ Varies<br>○ No included studies on IS	There is no published study of cost-effectiveness of Varicella vaccine in pediatric IBD patients.<br><br>An economic analysis of the universal varicella program in the US found that both the 1-dose program and 2-dose program were estimated to be cost saving from the societal perspective compared to no vaccination.[24] The incremental cost-effectiveness ratio for the second dose was $343 per case prevented, or approximately $109,000 per quality-adjusted life-year saved, and these results were sensitive to assumptions about vaccine effectiveness and prices.[24] The study concluded, "compared to the one-dose program, the two-dose program may not be cost effective".[24]<br><br>Since introduction of the universal varicella vaccination program, there has been a dramatic decline of varicella incidence. However, there has also been a dramatic rise in the incidence of adult shingles cases since HZ was added to the surveillance in 2000 (? detection bias with increased identification of HZ vs. real increase in incidence). There continues to be debate as to whether universal varicella vaccination program leads to unintended increase in HZ incidence.[25,26] Prior to the universal varicella vaccination program, 95% of adults experienced natural chickenpox – these cases were usually benign. In the prelicensure era, the periodic exogenous boosting that adults received from those shedding VZV resulted in long-term immunity, thereby reducing the risks of developing HZ (Hope-Simpson's exogeneous boosting hypothesis). As part of this hypothesis, Hope-Simpson postulated that reactivation of VZV was under immunologic control, and that this control could be boosted "endogeneously" due to reactivation of latent VZV, and "exogeneously" due to exposure to varicella. It is hypothesized that this high percentage of seropositive individuals and their long-term immunity may be compromised by the universal varicella vaccination of children which provides about 70-90% protection that is temporary and of unknown duration – shifting chickenpox to a more vulnerable adult populations which carries more risks of death and hospitalization. Therefore, it is highly controversial whether universal varicella vaccination is cost-effective as increased HZ morbidity may have disproportionately offset cost savings associated with reductions in varicella disease.[25,26] In part because of these concerns, a number of countries, including the UK and many European countries, did not implement universal varicella vaccination in children. Additional data will be needed to assess the impact of varicella vaccination on HZ.<br><br>Goldman did a cost-benefit analysis of universal varicella vaccination in the US taking into account the closely related HZ epidemiology.[27] This computer model reported that universal varicella vaccination had the impact of an additional 14.6 million HZ cases (or 42% increase) among adults aged <50 years during a 50-year period at a substantial medical cost burden of $4.1 billion or $80 million annually utilizing a very conservative estimated mean healthcare provider cost of $280 per HZ case.[27]	

**Acceptability**	**Is the intervention acceptable to key stakeholders?**<br><br>○ No<br>○ Probably no<br>○ Probably yes<br>○ Yes No on IS<br><br>○ Varies On IS<br>○ Don't know	No published studies to address patients or parents' acceptability of varicella vaccines in IBD patients<br><br>In a questionnaire survey study conducted in Israel, a total of 1474 parents completed the questionnaire.[28] Of the 624 children without a history of chicken pox, 34.1% were immunized against varicella.[28] Immunization rates were significantly lower in families with lower parental education and in patients with lower socioeconomic ranking. The main reasons for not being vaccinated were related to insufficient information about the vaccine itself, fear of adverse effects and waning immunity, preference of natural illness over immunization, and financial limitations.[28]	Varies acceptability due to outbreaks and herd immunity
**Feasibility**	**Is the intervention feasible to implement?**<br><br>○ No<br>○ Probably no<br>○ Probably yes<br>○ Yes<br><br>○ Varies<br>○ Don't know		

References:

1. Adams DJ, Nylund CM. Hospitalization for Varicella and Zoster in Children with Inflammatory Bowel Disease. J Pediatr. 2016 Apr;171:140-5.
2. World Health Organization. Systematic review of available evidence on effectiveness and duration of protection of varicella vaccines (through October 2013. http://www.who.int/immunization/sage/meetings/2014/april/4_Systematic_review_on_effectiveness_and_duration_of_protection_of_varicella_vaccines.pdf.
3. Seward JF, Marin M, Vázquez M. Varicella vaccine effectiveness in the US vaccination program: a review. J Infect Dis. 2008 Mar 1;197 Suppl 2:S82-9.
4. Bayer O, Heininger U, Heiligensetzer C, von Kries R. Metaanalysis of vaccine effectiveness in varicella outbreaks. Vaccine. 2007 Sep 17;25(37-38):6655-60.
5. Marin M, Marti M, Kambhampati A, Jeram SM, Seward JF. Global Varicella Vaccine Effectiveness: A Meta-analysis. Pediatrics. 2016 Mar;137(3):e20153741.
6. Croce E, Hatz C, Jonker EF, Visser LG, Jaeger VK, Bühler S. Safety of live vaccinations on immunosuppressive therapy in patients with immune-mediated inflammatory diseases, solid organ transplantation or after bone-marrow transplantation - A systematic review of randomized trials, observational studies and case reports. Vaccine. 2017 Mar 1;35(9):1216-1226.
7. deBruyn JCC, Soon IS, Fonseca K, Feng S, Purtzki M, Goedhart C, Kuhn S, Vanderkooi OG, Wrobel I. Serologic Status of Routine Childhood Vaccines, Cytomegalovirus, and Epstein-Barr Virus in Children With Inflammatory Bowel Disease. Inflamm Bowel Dis. 2019 Jun 18;25(7):1218-1226.
8. Ansari F, Baker RD, Patel R, Baker SS. Varicella immunity in inflammatory bowel disease. J Pediatr Gastroenterol Nutr. 2011 Oct;53(4):386-8.

9. Ferrera G, Gajdos V, Thomas S, Tran C, Fiquet A. Safety of a refrigerator-stable varicella vaccine (VARIVAX) in healthy 12- to 15-month-old children: A randomized, double-blind, cross-over study. Hum Vaccin. 2009 Jul;5(7):455-60.
10. Gatchalian S, Leboulleux D, Desauziers E, Bermal N, Borja-Tabora C. Immunogenicity and safety of a varicella vaccine, Okavax, and a trivalent measles, mumps and rubella vaccine, MMR-II, administered concomitantly in healthy Filipino children aged 12-24 months. Southeast Asian J Trop Med Public Health. 2003 Sep;34(3):589-97.
11. Lau YL, Vessey SJ, Chan IS, Lee TL, Huang LM, Lee CY, Lin TY, Lee BW, Kwan K, Kasim SM, Chan CY, Kaplan KM, Distefano DJ, Harmon AL, Golie A, Hartzel J, Xu J, Li S, Matthews H, Sadoff JC, Shaw A. A comparison of safety, tolerability and immunogenicity of Oka/Merck varicella vaccine and VARILRIX in healthy children. Vaccine. 2002 Jul 26;20(23-24):2942-9.
12. Parment PA, Svahn A, Rudén U, Bråkenhielm G, Storsaeter J, Akesson L, Linde A. Immunogenicity and reactogenicity of a single dose of live attenuated varicella vaccine and a booster dose of measles-mumps-rubella vaccine given concomitantly at 12 years of age. Scand J Infect Dis. 2003;35(10):736-42.
13. Ramkissoon A1, Coovadia HM, Jugnundan P, Haffejee IE, Meurice F, Vandevoorde D. Immunogenicity and safety of a live attenuated varicella vaccine in healthy Indian children aged 9-24 months. S Afr Med J. 1995 Dec;85(12):1295-8.
14. Shinefield HR, Black SB, Staehle BO, Matthews H, Adelman T, Ensor K, Li S, Chan I, Heyse J, Waters M, Chan CY, Vessey SJ, Kaplan KM, Kuter BJ; Kaiser Permanente Medical Team for Varivax. Vaccination with measles, mumps and rubella vaccine and varicella vaccine: safety, tolerability, immunogenicity, persistence of antibody and duration of protection against varicella in healthy children. Pediatr Infect Dis J. 2002 Jun;21(6):555-61.
15. Weibel RE, Neff BJ, Kuter BJ, Guess HA, Rothenberger CA, Fitzgerald AJ, Connor KA, McLean AA, Hilleman MR, Buynak EB, et al. Live attenuated varicella virus vaccine. Efficacy trial in healthy children. N Engl J Med. 1984 May 31;310(22):1409-15.
16. Black S, Shinefield H, Ray P, Lewis E, Hansen J, Schwalbe J, Coplan P, Sharrar R, Guess H. Postmarketing evaluation of the safety and effectiveness of varicella vaccine. Pediatr Infect Dis J. 1999 Dec;18(12):1041-6.
17. Chaves SS, Haber P, Walton K, Wise RP, Izurieta HS, Schmid DS, Seward JF. Safety of varicella vaccine after licensure in the United States: experience from reports to the vaccine adverse event reporting system, 1995-2005. J Infect Dis. 2008 Mar 1;197 Suppl 2:S170-7.
18. Ozaki T, Nishimura N, Kajita Y. Experience with live attenuated varicella vaccine (Oka strain) in healthy Japanese subjects; 10-year survey at pediatric clinic. Vaccine. 2000 May 8;18(22):2375-80.
19. Galea SA, Sweet A, Beninger P, Steinberg SP, Larussa PS, Gershon AA, Sharrar RG. The safety profile of varicella vaccine: a 10-year review. J Infect Dis. 2008 Mar 1;197 Suppl 2:S165-9.
20. Sharrar RG, LaRussa P, Galea SA, Steinberg SP, Sweet AR, Keatley RM, Wells ME, Stephenson WP, Gershon AA. The postmarketing safety profile of varicella vaccine. Vaccine. 2000 Nov 22;19(7-8):916-23.
21. Woodward M, Marko A, Galea S, Eagel B, Straus W. Varicella Virus Vaccine Live: A 22-Year Review of Postmarketing Safety Data. Open Forum Infect Dis. 2019 Aug 1;6(8).
22. Alexander KE, Tong PL, Macartney K, Beresford R, Sheppeard V, Gupta M. Live zoster vaccination in an immunocompromised patient leading to death secondary to disseminated varicella zoster virus infection. Vaccine. 2018 Jun 22;36(27):3890-3893
23. Denham JD, Lee DH, Castro M, Pandya S, Aslam S, Nanjappa S, Greene JN. Two cases of disseminated infection following live organism anti-cancer vaccine administration in cancer patients. Int J Infect Dis. 2018 Jul;72:1-2.
24. Leung VS, Nguyen MT, Bush TM. Disseminated primary varicella after initiation of infliximab for Crohn's disease. Am J Gastroenterol. 2004 Dec;99(12):2503-4.
25. Zhou F, Ortega-Sanchez IR, Guris D, Shefer A, Lieu T, Seward JF. An economic analysis of the universal varicella vaccination program in the United States. J Infect Dis. 2008 Mar 1;197 Suppl 2:S156-64.

26. Harpaz R, van Hoek AJ. Point-Counterpoint: The Hope-Simpson Hypothesis and Its Implications Regarding an Effect of Routine Varicella Vaccination on Herpes Zoster Incidence. J Infect Dis. 2018 Sep 22;218(suppl_2):S57-S62.
27. Goldman GS, King PG. Review of the United States universal varicella vaccination program: Herpes zoster incidence rates, cost-effectiveness, and vaccine efficacy based primarily on the Antelope Valley Varicella Active Surveillance Project data. Vaccine. 2013 Mar 25;31(13):1680-94.
28. Adler A, Herring E, Babilsky H, Gazala E, Cohen A, Levy I. Parent-dependent barriers to varicella immunization in Israel: the importance of adequate information. Acta Paediatr. 2007 Mar;96(3):428-31.

Conclusion – Pediatric

PICO 6A: In varicella-susceptible pediatric patients with IBD not on immunosuppressive therapy, should vaccination vs. no vaccination against varicella (chickenpox) be given?

Moderate certainty of evidence
Direction – Yes (100%)
Strength – Strong (100%)

**Type of recommendation**	**Strong recommendation against the intervention**	**Conditional recommendation against the intervention**	**Conditional recommendation for either the intervention or the comparison**	**Conditional recommendation for the intervention**	**Strong recommendation for the intervention**
	○	○	○	○	○ (selected)
**Recommendation**	**Statement 6A: In susceptible pediatric patients with IBD not on immunosuppressive therapy, we recommend varicella vaccine be given.**				
**Justification**					
**Subgroup considerations**					

**Implementation considerations**	
**Monitoring and evaluation**	• Ongoing monitoring of serious adverse events associated with varicella vaccine in IBD patients
**Research priorities**	• Observational or RCTs to determine the safety and clinical effectiveness of varicella vaccine in IBD patients on immunosuppressive therapy with assessment of patient-important outcomes (i.e. varicella infection, HZ etc.)<br>• Observational studies to establish correlates of seroprotection against varicella disease in IBD patients

PICO 6B: In varicella-susceptible pediatric patients with IBD on immunosuppressive therapy, should vaccination vs. no vaccination against varicella (chickenpox) be given?

Very low certainty of evidence

Direction – No (78%)

Strength – conditional

**Type of recommendation**	**Strong recommendation against the intervention**	**Conditional recommendation against the intervention**	**Conditional recommendation for either the intervention or the comparison**	**Conditional recommendation for the intervention**	**Strong recommendation for the intervention**
	○	● (highlighted)	○	○	○
**Recommendation**	**Statement 6B: In varicella-susceptible pediatric patients with IBD on immunosuppressive therapy,**				

	**we suggest against giving varicella vaccine.**
**Justification**	
**Subgroup considerations**	
**Implementation considerations**	
**Monitoring and evaluation**	• Ongoing monitoring of serious adverse events associated with varicella vaccine in IBD patients
**Research priorities**	• Observational or RCTs to determine the safety and clinical effectiveness of varicella vaccine in IBD patients on immunosuppressive therapy with assessment of patient-important outcomes (i.e. varicella infection, HZ etc.)<br>• Observational studies to establish correlates of seroprotection against varicella disease in IBD patients

Summary – Adults

**PICO 7A**	**In varicella-susceptible adult patients with IBD not on immunosuppressive therapy, should vaccination vs. no vaccination against varicella (chickenpox) be given?**
**Population**	**Varicella-susceptible adult patients with IBD not on immunosuppressive therapy**
**Intervention**	Vaccination against varicella

**Comparator**	No vaccination against varicella
**Outcome**	Mortality, VPI (varicella infection), SAEs, Immunogenicity

**PICO 7B**	**In varicella-susceptible adult patients with IBD on immunosuppressive therapy, should vaccination vs. no vaccination against varicella (chickenpox) be given?**
**Population**	**Varicella-susceptible adult patients with IBD on immunosuppressive therapy**
**Intervention**	Vaccination against varicella
**Comparator**	No vaccination against varicella
**Outcome**	Mortality, VPI (varicella infection), SAEs, Immunogenicity

There was no RCT comparing varicella vaccines with placebo in patients with IBD to address this PICO question.

There are 3 observational studies and observational data from 1 RCT comparing 4 vs. 8 weeks of a 2-dose varicella vaccine that assessed the effectiveness of varicella vaccines in healthy adults with a maximum duration of follow-up of 6 years.[1-4] The studies included mostly healthy adults considered susceptible to varicella infection (i.e. healthcare workers or parents of young children).[1-4] In total, 0.26-7% of included patients developed mild varicella infection.[1-4] In one study, the vaccine efficacy (attack rate unvaccinated – attack rate vaccinated / attack rate unvaccinated x 100) was estimated to be 51% assuming a 90% attack rate in susceptible subjects (varicella exposed household contacts).[3] This is in contrast to reported vaccine effectiveness of 92% (88-95%) in healthy children.[5] The seroconversion rates were however very high with 2-dose varicella vaccine (92-99%) in healthy adults. No serious adverse events were reported by any of the studies.[1-4] The certainty of evidence started as low due to observational designs of these studies. The certainty of evidence is downgraded to **very low** due to indirectness (when applied to IBD patients) and imprecision (relatively rare events of varicella infection with sample size < 2000).

One systematic review included 40 observational studies (mostly cohort studies and case series/reports) in patients with immune-mediated diseases (2852/20,556 were IBD patients, mostly children) on 22 different immunosuppressive medications.[6] The immunosuppressive medications used by IMID patients included prednisone 2.5-35mg/day, methotrexate 5-27mg/week, 6MP, biologic monotherapy, and combination therapy with biologic and immunomodulator.[6] The seroconversion rates in IMID group were high with varicella vaccines.[6] However, methotrexate and anti-TNF therapy appeared to reduce the seroconversion rates.[6] No subgroup data was provided for IBD patients.[6] The GRADE rating started as low due to observational designs of these studies. The

evidence was further downgraded to very low due to study limitations (residual confounding and selection bias) and indirectness (surrogate outcomes, IMID patients, mostly children).

Overall, there is <u>very low</u> certainty evidence that varicella vaccine is effective in adult IBD patients.

Safety of varicella vaccine in healthy adults was assessed by the 4 observational studies.[1-4] No serious adverse events was reported by any of the studies.[1-4] The GRADE rating started as low, and the evidence was downgraded to **very low** due to imprecision and indirectness (not IBD patients). One systematic review included 40 observational studies in patients with immune-mediated diseases (2852/20,556 were IBD patients) on 22 different immunosuppressive medications.[6] The administration of live vaccines was safe in most studies of IMID patients on immunosuppressive medications (including prednisone 2.5-35mg/day, methotrexate 5-27mg/week, 6MP, biologic monotherapy, and combination therapy with biologic and immunomodulator). Serious adverse events were rare (0.05%, 11/20,556 IMID patients).[6] Infections through the vaccine strain were also rare, occurring in 0.06% (12/20,556 IMID patients).[6] In most cases, infection was mild. However, two patients had fatal infection: a patient with RA/SLE overlap who started methotrexate/dexamethasone treatment 4 days after the yellow fever vaccine developed vaccine-associated viscerotropic disease and died. One infant whose mother was under infliximab treatment during pregnancy received the BCG vaccine at the age of 3 months and developed disseminated BCG infection and died. The certainty of evidence started as low due to the observational designs of these studies. The evidence was downgraded to very low due to study limitations and indirectness (patient population).

Overall, there is <u>very low</u> certainty evidence that varicella vaccines are safe in adult IBD populations.

A large safety analysis was just published (outside of our literature review parameters) containing 22 years of post-marketing adverse event data.[7] Spontaneous, voluntary reporting of adverse events and non-interventional study reports submitted by health care providers was the basis for the review which spanned 1995 – 2017. During this time, >212 million doses of varicella vaccines were distributed globally. Reported rates are calculated based on total doses distributed and the assumption that each patient received 1 dose of the vaccine. 46855 adverse event reports were received. Disseminated disease caused by the vaccine strain was confirmed by PCR in 39 cases.[7] 28 cases occurred in immunocompromised individuals and/or who reported concomitant use of immunosuppressive therapies (including patients with rheumatoid arthritis on prednisone and Methotrexate, SLE on pulse steroids, IBD with protein losing enteropathy and hypogammaglobulinemia on multiple immunosuppressive therapy).[7] 86 cases of death (0.002%) were reported after vaccination with 26 occurring in immunocompromised patients (no reported fatal case in IBD patients).[7] It should be noted that these events were temporally associated with varicella vaccination, but may not have been causally associated. 25% of reports contained insufficient data to establish the cause of death.[7]

Overall, there is <u>very low</u> certainty evidence that varicella vaccines are safety and effective in adult IBD patients (on or not on immunosuppressive medications).

Evidence Profile Table – Adults

Effectiveness and Safety of Varicella Vaccine in Adult IBD Patients

Certainty Assessment								Summary of Findings	
Studies	Risk of bias	Inconsistency	Indirectness	Imprecision	Other considerations	Certainty of Evidence	Overall Certainty of evidence	Study Event Rates	Relative Effect (95% CI)
**VPI (Varicella infection) - CRITICAL**							⊕⊖⊖⊖ VERY LOW		
4 Observational studies[1-4] **Healthy Adults**	Not serious	Not serious	Serious[a]	Serious[b]	None	⊕⊖⊖⊖ VERY LOW		• Vaccine efficacy was estimated to be 51% assuming a 90% attack rate in susceptible subjects (or 45% assuming an 80% attack rate in susceptible subjects in 1 study).[3]	
**Immunogenicity (Varicella antibody titer) - IMPORTANT**									
4 Observational studies[1-4] **Healthy Adults**	Not serious	Not serious	Serious[c]	Serious[b]	None	⊕⊖⊖⊖ VERY LOW		• High seroconversion rates (92-99%) against varicella vaccines in healthy adults after 2 doses	
1 SR of 40 Observational Studies[6] **20,556 IMID patients with 2852 IBD patients**	Serious[d]	Not serious	Serious[e]	Not serious	None	⊕⊖⊖⊖ VERY LOW		• High seroconversion rates against varicella vaccines in IMID patients on immunosuppressive medications<br>• MTX and anti-TNF appeared to reduce immune response to varicella vaccines.	
**Serious Adverse Events - CRITICAL**									
4 Observational studies[1-4] **Healthy Adults**	Not serious	Not serious	Serious[a]	Serious[b]	None	⊕⊖⊖⊖ VERY LOW		• No serious adverse events.	

1 SR of 40 Observational Studies[6]<br><br>**20,556 IMID patients with 2852 IBD patients**	Serious[d]	Not serious	Serious[e]	Not serious	None	⊕⊖⊖⊖<br>VERY LOW		• The administration of live vaccines was safe in most studies of IMID patients on immunosuppressive medications<br>• Serious adverse events were reported in 11/20,556 (0.05%) IMID patients<br>• Infection through the vaccine strain in 12/20,556 (0.06%) IMID patients. Infection was mild in most cases. However, 2 patients had fatal infection (yellow fever vaccine, BCG vaccine)<br>• No increase flares of autoimmune diseases in most studies

Footnotes:

a. Downgraded for indirectness. Patient population is healthy adults (not IBD patients).
b. Downgraded for imprecision. Rare events and total sample size < 2000.
c. Downgraded for indirectness. Patient population is healthy adults (not IBD patients). Surrogate outcomes were used for vaccine effectiveness.
d. Downgraded for study limitations. Residual confounding cannot be ruled out given the observational nature of these studies (e.g. comorbidities, concurrent illnesses, disease activity and duration, nutritional status, and other factors which may affect the risk of varicella infection) as well as selection bias.
e. Downgraded due to indirectness (population and surrogate outcomes). Only 14% (32852/20,556) IMID patients were patients with IBD, and most were children. Surrogate outcomes were used for vaccine effectiveness.

References:

1. Ndumbe PM, Cradock-Watson JE, MacQueen S, Dunn H, Holzel H, André F, Davies EG, Dudgeon JA, Levinsky RJ. Immunisation of nurses with a live varicella vaccine. Lancet. 1985 May 18;1(8438):1144-7.
2. Gershon AA, Steinberg SP, Gelb L. Live attenuated varicella vaccine use in immunocompromised children and adults. Pediatrics. 1986 Oct;78(4 Pt 2):757-62.
3. Gershon AA, Steinberg SP, LaRussa P, Ferrara A, Hammerschlag M, Gelb L. Immunization of healthy adults with live attenuated varicella vaccine. J Infect Dis. 1988 Jul;158(1):132-7.
4. Kuter BJ, Ngai A, Patterson CM, Staehle BO, Cho I, Matthews H, Provost PJ, White CJ. Safety, tolerability, and immunogenicity of two regimens of Oka/Merck varicella vaccine (Varivax) in healthy adolescents and adults. Oka/Merck Varicella Vaccine Study Group. Vaccine. 1995 Aug;13(11):967-72.
5. Marin M, Marti M, Kambhampati A, Jeram SM, Seward JF. Global Varicella Vaccine Effectiveness: A Meta-analysis. Pediatrics. 2016 Mar;137(3):e20153741.
6. Croce E, Hatz C, Jonker EF, Visser LG, Jaeger VK, Bühler S. Safety of live vaccinations on immunosuppressive therapy in patients with immune-mediated inflammatory diseases, solid organ transplantation or after bone-marrow transplantation - A systematic review of randomized trials, observational studies and case reports. Vaccine. 2017 Mar 1;35(9):1216-1226.

7. Woodward M, Marko A, Galea S, Eagel B, Straus W. Varicella Virus Vaccine Live: A 22-Year Review of Postmarketing Safety Data. Open Forum Infect Dis. 2019 Aug 1;6(8).

Evidence to Decision Table – Adults

**PICO 7A**	**In varicella-susceptible adult patients with IBD not on immunosuppressive therapy, should vaccination vs. no vaccination against varicella (chickenpox) be given?**
**Population**	**Varicella-susceptible adult patients with IBD not on immunosuppressive therapy**
**Intervention**	Vaccination against varicella
**Comparator**	No vaccination against varicella
**Outcome**	Mortality, VPI (varicella infection), SAEs, Immunogenicity

**PICO 7B**	**In varicella-susceptible adult patients with IBD on immunosuppressive therapy, should vaccination vs. no vaccination against varicella (chickenpox) be given?**
**Population**	**Varicella-susceptible adult patients with IBD on immunosuppressive therapy**
**Intervention**	Vaccination against varicella
**Comparator**	No vaccination against varicella
**Outcome**	Mortality, VPI (varicella infection), SAEs, Immunogenicity

Judgement	Research evidence	Additional considerations

<table>
<tr>
<td>Desirable Effects</td>
<td>How substantial are the desirable anticipated effects?

○ Trivial
○ Small
○ Moderate not on IS
○ Large

○ Varies
○ Don't know on IS</td>
<td rowspan="2">Risks of Varicella Infection in Adult IBD Patients

Varicella zoster virus (VZV) predominantly affects children in temperate countries, with near-universal seroconversion occurring by late childhood.[1,2] However, in tropical regions, VZV infection is a less common childhood infection, and up to 50% of adults in these areas may have no history of primary infection.[3,4] Unfortunately, primary VZV infection is often more severe in adults than in children. In contrast to primary VZV infection, reactivation of the VZV (herpes zoster - HZ) tend to occur mainly in older adults (age > 50) and in those who are immunosuppressed.
Literature search did not identify any study on the risk of primary varicella infection in adult IBD patients. There are a number of case reports of primary varicella infection in immunosuppressed IBD patients, with severe disease course and fatalities reported. In a review article by Cullen et al in 2012, there were 20 reported cases of primary varicella infection IBD patients with five deaths.[5] Sixteen of the reported cases occurred in individuals age ≥ 18, with 3 cases resulting in death.[5] Thirteen of the cases involved organs other than the skin.[5] Nine of the 20 cases involved anti-TNF therapy; seven of these 9 were on combination immunosuppression.[5] Thirteen patients were on steroids and 12 were on either a thiopurine or methotrexate.[5]

For the risk of HZ in IBD patients, please see under Herpes Zoster. There is <u>very low</u> certainty evidence that adult IBD patients younger than age 50 have an increased risk of HZ compared to non-IBD patients older than age 50. There is <u>very low</u> certainty evidence that steroids, combination therapy (steroids + thiopurines or anti-TNF, steroids + thiopurines + anti-TNF, thiopurines + anti-TNF), thiopurines alone, and anti-TNF alone were associated with increased risks of HZ among IBD patients.

Effectiveness and Safety of Varicella Vaccine in Adult IBD Patients

There was no RCT comparing varicella vaccines with placebo in patients with IBD to address this PICO question.

There are 3 observational studies and observational data from 1 RCT comparing 4 vs. 8 weeks of a 2-dose varicella vaccine that assessed the effectiveness of varicella vaccines in healthy adults with a maximum duration of follow-up of 6 years.[6-9] The studies included mostly healthy adults considered susceptible to varicella infection (i.e. healthcare workers or parents of young children).[6-9] In total, 0.26-7% of included patients developed mild varicella infection.[6-9] In one study, the vaccine efficacy (attack rate unvaccinated – attack rate vaccinated / attack rate unvaccinated x 100) was estimated to be 51% assuming a 90% attack rate in susceptible subjects (varicella exposed household contacts).[8] This is in contrast to reported vaccine effectiveness of 92% (88-95%) in healthy children.[10] The seroconversion rates were however very high with 2-dose varicella vaccine (92-99%) in healthy adults. No serious adverse events were reported by any of the studies.[6-9] The certainty of evidence started as low due to observational designs of these studies. The certainty of evidence is downgraded to very low due to indirectness (when applied to IBD patients) and imprecision (relatively rare events of varicella infection with sample size < 2000).</td>
<td></td>
</tr>
<tr>
<td>Undesirable Effects</td>
<td>How substantial are the undesirable anticipated effects?

○ Large
○ Moderate
○ Small on IS
○ Trivial Not on IS

○ Varies
○ Don't know</td>
<td></td>
</tr>
</table>

		One systematic review included 40 observational studies (mostly cohort studies and case series/reports) in patients with immune-mediated diseases (2852/20,556 were IBD patients, mostly children) on 22 different immunosuppressive medications.[11] The immunosuppressive medications used by IMID patients included prednisone 2.5-35mg/day, methotrexate 5-27mg/week, 6MP, biologic monotherapy, and combination therapy with biologic and immunomodulator.[11] The seroconversion rates in IMID group were high with varicella vaccines.[11] However, methotrexate and anti-TNF therapy appeared to reduce the seroconversion rates.[11] No subgroup data was provided for IBD patients.[11] The GRADE rating started as low due to observational designs of these studies. The evidence was further downgraded to **very low** due to study limitations (residual confounding and selection bias) and indirectness (surrogate outcomes, IMID patients, mostly children).<br><br>**Overall, there is <u>very low</u> certainty evidence that varicella vaccine is effective in adult IBD patients.**<br><br>Safety of varicella vaccine in healthy adults was assessed by the 4 observational studies.[6-9] No serious adverse events was reported by any of the studies.[6-9] The GRADE rating started as low, and the evidence was downgraded to **very low** due to imprecision and indirectness (not IBD patients). One systematic review included 40 observational studies in patients with immune-mediated diseases (2852/20,556 were IBD patients) on 22 different immunosuppressive medications.[11] The administration of live vaccines was safe in most studies of IMID patients on immunosuppressive medications (including prednisone 2.5-35mg/day, methotrexate 5-27mg/week, 6MP, biologic monotherapy, and combination therapy with biologic and immunomodulator). Serious adverse events were rare (0.05%, 11/20,556 IMID patients).[11] Infections through the vaccine strain were also rare, occurring in 0.06% (12/20,556 IMID patients).[11] In most cases, infection was mild. However, two patients had fatal infection: a patient with RA/SLE overlap who started methotrexate/dexamethasone treatment 4 days after the yellow fever vaccine developed vaccine-associated viscerotropic disease and died. One infant whose mother was under infliximab treatment during pregnancy received the BCG vaccine at the age of 3 months and developed disseminated BCG infection and died. The certainty of evidence started as low due to the observational designs of these studies. The evidence was downgraded to very low due to study limitations and indirectness (patient population).<br><br>**Overall, there is <u>very low</u> certainty evidence that varicella vaccines are safe in adult IBD populations.**<br><br>A large safety analysis was just published (outside of our literature review parameters) containing 22 years of post-marketing adverse event data.[12] Spontaneous, voluntary reporting of adverse events and non-interventional study reports submitted by health care providers was the basis for the review which spanned 1995 – 2017. During this time, >212 million doses of varicella vaccines were distributed globally. Reported rates are calculated based on total doses distributed and the assumption that each patient received 1 dose of the vaccine. 46855 adverse event reports were received. Disseminated disease caused by the vaccine strain was confirmed by PCR in 39 cases.[12]	

		28 cases occurred in immunocompromised individuals and/or who reported concomitant use of immunosuppressive therapies (including patients with rheumatoid arthritis on prednisone and Methotrexate, SLE on pulse steroids, IBD with protein losing enteropathy and hypogammaglobulinemia on multiple immunosuppressive therapy).[7] 86 cases of death (0.002%) were reported after vaccination with 26 occurring in immunocompromised patients (no reported fatal case in IBD patients).[12] It should be noted that these events were temporally associated with varicella vaccination, but may not have been causally associated. 25% of reports contained insufficient data to establish the cause of death.[12]<br><br>**Overall, there is very low certainty evidence that varicella vaccines are safety and effective in adult IBD patients (on or not on immunosuppressive medications).**	
**Certainty of evidence**	**What is the overall certainty of the evidence of effects?**<br><br>○ Very low IBD patients on or not on IS<br>○ Low<br>○ Moderate<br>○ High<br><br>○ No included studies		
**Values and Preferences**	**Is there important uncertainty about or variability in how much people value the main outcomes?**<br><br>○ Important uncertainty or variability<br>○ Possibly important uncertainty or variability<br>○ Probably no important uncertainty or variability<br>○ No important uncertainty or variability	Patients likely value patient-important outcomes (mortality, VPI, adverse effects) more than surrogate outcomes (immunogenicity).	
**Balance of effects**	**Does the balance between desirable and undesirable effects favor the intervention or the comparison?**<br><br>○ Favors the comparison<br>○ Probably favors the comparison<br>○ Does not favor either the intervention or the comparison<br>○ Probably favors the intervention<br>○ Favors the intervention not on IS<br><br>○ Varies		

	○ Don't know on IS		
**Resources required**	**How large are the resource requirements (costs)?**<br><br>○ Large costs<br>○ Moderate costs<br>○ Negligible costs and savings<br>○ Moderate savings<br>○ Large savings<br><br>○ Varies<br>○ Don't know	CDC vaccine price list last reviewed/updated: July 1, 2019<br><br>**Brandname**: Varivax® — **CDC cost/dose**: $104.09 (children); $84.88 (adults) — **Private sector cost/dose**: $135.73<br><br>Varivax is given as a 2-dose vaccine.	
**Certainty of Evidence of Required Resources**	**What is the certainty of the evidence of resource requirements (costs)?**<br><br>○ Very low<br>○ Low<br>○ Moderate<br>○ High<br><br>○ No included studies	The costs of delivering routine immunization services may vary widely across countries and different health system settings. See Immunization Costing Action Network (ICAN) Immunization Delivery Cost Catalogue. http://immunizationeconomics.org/ican-idcc.	
**Cost effectiveness**	**Does the cost-effectiveness of the intervention favor the intervention or the comparison?**<br><br>○ Favors the comparison<br>○ Probably favors the comparison<br>○ Does not favor either the intervention or the comparison<br>○ Probably favors the intervention<br>○ Favors the intervention	There is no published study of cost-effectiveness of Varicella vaccine in adult IBD patients.<br><br>Merrett conducted a cost-effectiveness analysis to identify the optimal vaccination strategy for adult immigrants and refugees arriving in industrialized countries, such as Canada.[13] Routine serological testing of young adult immigrants in Montreal without a self-reported history of varicella, followed by vaccination of individuals found to be susceptible to varicella, would prevent an estimated 37% of cases and would be the most cost-saving intervention from a societal perspective (relative to no intervention,	Need to consider whether adult IBD patients are "susceptible" adults for varicella infection.

Embedded table (Resources required row):

Brandname	CDC cost/dose	Private sector cost/dose
Varivax®	$104.09 (children)<br><br>$84.88 (adults)	$135.73

	○ Varies<br>○ No included studies	vaccination of all individuals, vaccination of individuals with a negative or uncertain history of varicella, serological testing of all individuals and vaccination of those with results indicating susceptibility to varicella).[13] **However, if the annual varicella attack rate was <3.8% among susceptible adults or if <5% of the population was susceptible to varicella (i.e., if>95% of the population had varicella antibody), then selective serological testing was no longer cost-saving. This is consistent with other cost-effectiveness analyses in healthy susceptible individuals**.[13] Chodick et al concluded that serological testing of workers with a negative or uncertain history of varicella with subsequent vaccination of those individuals found to be susceptible to varicella was the most cost-effective strategy for health care workers, preventing an estimated 43% of cases, compared with no intervention.[13]<br><br>However, varicella transmission dynamics are likely to be different in the era of universal childhood vaccination. On one hand, the circulation of varicella in the population may be lower, resulting in fewer opportunities for exposure and perhaps a lower risk of contracting varicella, even among susceptible adults. On the other hand, there is concern about the possible accumulation of young adults with partial or complete primary vaccine failure occurring in conjunction with reduced opportunities for natural boosting from the community. This growing pool of susceptible individuals may place young adults at higher risk of disease from outbreaks.	
**Acceptability**	**Is the intervention acceptable to key stakeholders?**<br><br>○ No<br>○ Probably no<br>○ Probably yes<br>○ Yes not on IS<br><br>○ Varies on IS<br>○ Don't know	No published studies to address patients or parents' acceptability of varicella vaccines in IBD patients.	
**Feasibility**	**Is the intervention feasible to implement?**<br><br>○ No<br>○ Probably no<br>○ Probably yes<br>○ Yes<br><br>○ Varies<br>○ Don't know		

References:

1. Arvin AM. Varicella-zoster virus. Clin Microbiol Rev. 1996 Jul;9(3):361-81.

2. Bollaerts K, Riera-Montes M, Heininger U, Hens N, Souverain A, Verstraeten T, Hartwig S. A systematic review of varicella seroprevalence in European countries before universal childhood immunization: deriving incidence from seroprevalence data. Epidemiol Infect. 2017 Oct;145(13):2666-2677.
3. Hussey H, Abdullahi L, Collins J, Muloiwa R, Hussey G, Kagina B. Varicella zoster virus-associated morbidity and mortality in Africa - a systematic review. BMC Infect Dis. 2017 Nov 14;17(1):717.
4. Fatha N, Ang LW, Goh KT. Changing seroprevalence of varicella zoster virus infection in a tropical city state, Singapore. Int J Infect Dis. 2014 May;22:73-7.
5. Cullen G, Baden RP, Cheifetz AS. Varicella zoster virus infection in inflammatory bowel disease. Inflamm Bowel Dis. 2012 Dec;18(12):2392-403.
6. Ndumbe PM, Cradock-Watson JE, MacQueen S, Dunn H, Holzel H, André F, Davies EG, Dudgeon JA, Levinsky RJ. Immunisation of nurses with a live varicella vaccine. Lancet. 1985 May 18;1(8438):1144-7.
7. Gershon AA, Steinberg SP, Gelb L. Live attenuated varicella vaccine use in immunocompromised children and adults. Pediatrics. 1986 Oct;78(4 Pt 2):757-62.
8. Gershon AA, Steinberg SP, LaRussa P, Ferrara A, Hammerschlag M, Gelb L. Immunization of healthy adults with live attenuated varicella vaccine. J Infect Dis. 1988 Jul;158(1):132-7.
9. Kuter BJ, Ngai A, Patterson CM, Staehle BO, Cho I, Matthews H, Provost PJ, White CJ. Safety, tolerability, and immunogenicity of two regimens of Oka/Merck varicella vaccine (Varivax) in healthy adolescents and adults. Oka/Merck Varicella Vaccine Study Group. Vaccine. 1995 Aug;13(11):967-72.
10. Marin M, Marti M, Kambhampati A, Jeram SM, Seward JF. Global Varicella Vaccine Effectiveness: A Meta-analysis. Pediatrics. 2016 Mar;137(3):e20153741.
11. Croce E, Hatz C, Jonker EF, Visser LG, Jaeger VK, Bühler S. Safety of live vaccinations on immunosuppressive therapy in patients with immune-mediated inflammatory diseases, solid organ transplantation or after bone-marrow transplantation - A systematic review of randomized trials, observational studies and case reports. Vaccine. 2017 Mar 1;35(9):1216-1226.
12. Woodward M, Marko A, Galea S, Eagel B, Straus W. Varicella Virus Vaccine Live: A 22-Year Review of Postmarketing Safety Data. Open Forum Infect Dis. 2019 Aug 1;6(8).
13. Merrett P, Schwartzman K, Rivest P, Greenaway C. Strategies to prevent varicella among newly arrived adult immigrants and refugees: a cost-effectiveness analysis. Clin Infect Dis. 2007 Apr 15;44(8):1040-8.
14. Chodick G, Ashkenazi S, Livni G, Lerman Y. Cost-effectiveness of varicella vaccination of healthcare workers. Vaccine. 2005 Oct 17;23(43):5064-72.

Conclusion – Adults

PICO 7A: In varicella-susceptible adult patients with IBD not on immunosuppressive medications, should varicella vaccine be given?

Very low certainty of evidence
Direction – Yes (100%)
Strength – conditional

Type of recommendation	Strong recommendation against the intervention	Conditional recommendation against the intervention	Conditional recommendation for either the intervention or the comparison	Conditional recommendation for the intervention	Strong recommendation for the intervention
	o	o	o	o	o

**Recommendation**	**Statement 7A: In varicella-susceptible adult patients with IBD not on immunosuppressive therapy, we suggest varicella vaccine be given.**
**Justification**	
**Subgroup considerations**	
**Implementation considerations**	
**Monitoring and evaluation**	• Ongoing monitoring of serious adverse events associated with varicella vaccine in IBD patients
**Research priorities**	• Observational or RCTs to determine the safety and clinical effectiveness of varicella vaccine in IBD patients on immunosuppressive therapy with assessment of patient-important outcomes (i.e. varicella infection, HZ etc.)<br>• Observational studies to establish correlates of seroprotection against varicella disease in IBD patients

PICO 7B: In susceptible adult patients with IBD on immunosuppressive medications, should varicella vaccine be given?
Direction – No (100%)
Strength – conditional

**Type of recommendation**	**Strong recommendation against the intervention**<br>○	**Conditional recommendation against the intervention**<br>○	**Conditional recommendation for either the intervention or the comparison**<br>○	**Conditional recommendation for the intervention**<br>○	**Strong recommendation for the intervention**<br>○
**Recommendation**	**Statement 7B: In susceptible adult patients with IBD on immunosuppressive therapy, we suggest against giving varicella vaccine.**				
**Justification**					
**Subgroup considerations**					
**Implementation considerations**					
**Monitoring and evaluation**	• Ongoing monitoring of serious adverse events associated with varicella vaccine in IBD patients				
**Research priorities**	• Observational or RCTs to determine the safety and clinical effectiveness of varicella vaccine in IBD patients on immunosuppressive therapy with assessment of patient-important outcomes (i.e. varicella infection, HZ etc.)<br>• Observational studies to establish correlates of seroprotection against varicella disease in IBD patients				

Live Vaccines in Infants Born of Mother with IBD Using Biological Therapies

Background

Live vaccines contain live attenuated microorganisms which are still capable of replicating within the host (vaccinee). These include measles, mumps, rubella, rotavirus, smallpox, chickenpox, yellow fever, and Bacillus Calmette-Guerin (BCG) vaccines. Because live vaccines may cause disease by uncontrolled replication, **CDC considers "severe immunosuppression" as a contraindication to live vaccines.**[1] Severe immunosuppression can be due to a variety of conditions, including congenital immunodeficiency, human immunodeficiency virus (HIV) infection, leukemia, lymphoma, generalized malignancy or therapy with alkylating agents, antimetabolites, radiation, or large amounts of corticosteroids. For some of these conditions, all affected persons will be severely immunocompromised; for others, such as HIV infection, the spectrum of disease severity due to disease or treatment stage will determine the degree to which the immune system is compromised. As per CDC, **the responsibility for determining whether a patient is severely immunocompromised ultimately lies with the physician.**[1]

In North America, live vaccines such as measles, mumps, rubella (MMR) vaccines and varicella are routinely given at and/or after 12 months of age. Under special circumstances for international travellers, MMR vaccine may be given to infants at age 6 – 11 months followed by a second dose at 12-15 months. In contrast, rotavirus vaccine is usually given before 15 weeks of age. In some countries, infants may be exposed to other live vaccines in the first 6 months including BCG, oral polio and smallpox.

Rotavirus is a common cause of gastroenteritis in children with varying presentation, including asymptomatic infection, mild disease, severe dehydration, and very rarely death in developed countries. It is associated with considerable health care resource utilization. Most unimmunized children are infected by 5 years of age. Rotavirus vaccine efficacy against diarrhea of any severity in developed world settings is 74-87%. It is usually well tolerated, but there is a small increased risk of intussusception. **Both CDC and NACI recommend routine rotavirus vaccine before 15 weeks of age, except in those who are known or suspected to have severe combined immunodeficiency (SCID) or other significant "immunocompromising conditions"**.[1,2] CDC recommends practitioners to consider the potential risks and benefits of administering rotavirus vaccine to infants with known or suspected altered

immunocompetence; consultation with an immunologist or infectious diseases specialist is advised. Children and adults who are immunocompromised because of congenital immunodeficiency, hematopoetic transplantation, or solid organ transplantation sometimes experience severe or prolonged rotavirus gastroenteritis. **However, no safety or efficacy data are available for the administration of rotavirus vaccine to infants who are immunocompromised or potentially immunocompromised**, including 1) infants with primary and acquired immunodeficiency states, cellular immunodeficiencies, and hypogammaglobulinemic and dysgammaglobulinemic states; 2) infants with blood dyscrasias, leukemia, lymphomas, or other malignant neoplasms affecting the bone marrow or lymphatic system; 3) infants on immunosuppressive therapy (including high-dose systemic corticosteroids); and 4) infants who are HIV-exposed or infected.[1] In response to reported cases of vaccine-acquired rotavirus infection in infants with severe combined immunodeficiency (SCID) following rotavirus vaccine administration, CDC recommends not to give rotavirus vaccine to infants diagnosed with SCID.[1]

The Toronto consensus guideline recommended against administration of live vaccinations within the first 6 months of life to newborns of women who were on anti-TNF therapy during pregnancy (strong recommendation, very low-quality evidence).[3] Despite very low-quality evidence, the recommendation was strong, based on the potential for catastrophic harm associated with early use of live vaccines. If vaccinations are absolutely necessary because of childcare regulations, imminent travel, or exposure to a high-risk area, then it may be prudent to measure anti-TNF serum levels in the infant to help inform decisions.[3] If anti-TNF was stopped after the second trimester to limit transfer to infant, then live vaccination should still be deferred to 6 months of age when possible or blood levels in the infant should be assessed, because the impact of discontinuing therapy on drug levels in infant has not been systematically assessed.[3]

Two Rotavirus vaccines are authorized for use in North America. Rotarix® is a live, oral, monovalent, attenuated, human rotavirus vaccine and Rotateq® is a live, oral, pentavalent vaccine.

References:

1. Centers for Disease Control and Prevention. https://www.cdc.gov/vaccines/vpd/rotavirus/hcp/index.html
2. Public Health Agency of Canada. https://www.canada.ca/en/public-health/services/publications/healthy-living/canadian-immunization-guide-part-4-active-vaccines/page-19-rotavirus-vaccine.html
3. Nguyen GC, Seow CH, Maxwell C, Huang V, Leung Y, Jones J, Leontiadis GI, Tse F, Mahadevan U, van der Woude CJ; IBD in Pregnancy Consensus Group; Canadian Association of Gastroenterology. The Toronto Consensus Statements for the Management of Inflammatory Bowel Disease in Pregnancy. Gastroenterology. 2016 Mar;150(3):734-757

Summary

PICO	**In infants born of mother using biological therapies, should live vaccines vs. no live vaccines be given in the first 6 months of life?**
**Population**	Infants born of mother using biological therapies
**Intervention**	Administration of live vaccines in the first 6 months of life
**Comparator**	No administration of live vaccines in the first 6 months of life
**Outcome**	Mortality, VPI, SAEs, Immunogenicity

Because live vaccines may cause disease by uncontrolled replication in immunocompromised persons, the literature review for this PICO question focused on **safety outcomes.**

Serious adverse events to live vaccines in biologic-exposed infants

Literature review found 7 observational studies (small cohort and case series) of infants exposed to biologic agents in utero who were given live vaccines (56 infants received rotavirus vaccine at less than 6 months of age, 74 received BCG vaccine within 6 months of age, 52 received MMR or rubella vaccine at 15 months of age).[1-7] The most common biologics used during these pregnancies were anti-TNF agents (infliximab followed by adalimumab and certolizumab). Vedolizumab and Ustekinumab were used in a small minority of cases. Most of these biologics were stopped either in the second or third trimester. No serious adverse events were experienced by any of the infants except a death attributed to disseminated BCG infection after administration of the BCG vaccine to a 3-month old infant exposed in utero to infliximab.[7] The GRADE rating started at low due to the observational designs of these studies. **The certainty of evidence was downgraded to <u>very low</u> due to study limitations, inconsistency, indirectness and imprecision.** (See **Summary of observational studies assessing immunologic or clinical outcomes of infants exposed to biologics in utero who were given live vaccines)**

Anti-TNF drug clearance in exposed infants

Anti-TNF agents are IgG1 antibodies that cross the placenta by active transport starting at the end of the second trimester but mainly in the third trimester. It has therefore been common practice to discontinue infliximab and adalimumab during the second or third trimester to reduce the transplacental transfer of these drugs to the fetus. In contrast, certolizumab pegol, by virtue of being a

PEG (polyethylene glycolylated) FaB immunoglobulin, does not bind to the neonatal FcY receptor responsible for active transplacental transfer.

Three observational studies have shown that both infliximab and adalimumab can be detected in the cord blood at delivery even when these drugs were stopped in the second or third trimester, usually in levels higher than those in maternal serum (about 1.5 to 2-fold higher).[8-10] Additionally, Julsgaard et al. demonstrated that infliximab concentration could persist for up to 12 months (in 1/80 biologic exposed infants) and adalimumab for up to 6 months after birth.[10] The average half-life of anti-TNF in exposed infants was longer than in adult non-pregnant patients: adalimumab was 26 days (95% CI 23-29 days) and infliximab was 33 days (95% CI 30-37 days).[10] The mean time to drug clearance in infants was 4.0 months for adalimumab (95% CI 2.9-5.0 and 7.3 months for infliximab (95% CI 6.2-8.3). Certolizumab was detachable at minimal levels in infant or cord blood likely due to passive diffusion.[9] It is important to note that previous studies have found insignificant amounts of anti-TNF and vedolizumab in breast milk of nursing IBD patients, and are unlikely to result in systemic immunosuppression of the infant.[11-14] In the study by Julsgaard et al, no statistically significant associations between drug half-life and maternal breastfeeding was found.[10] The GRADE rating started at low due to the observational designs of these studies. Anti-TNF level detected in exposed infants is a surrogate outcome for immunosuppression which in turn is a surrogate outcome for potential adverse events related to administration of live vaccines. **The certainty of evidence was downgraded to <u>very low</u> due to study limitations, indirectness and imprecision.**

Immunophenotyping in anti-TNF exposed infants

Finally, immunophenotyping studies have shown that anti-TNF exposed infants had more immature B- and helper T phenotype at birth[5] that normalized by 12 months.[5,15] A decreased response after mycobacterial challenge was noted in 1 study.[5] Observational studies have found that infants exposed to anti-TNF in utero have appropriate response to inactivated vaccines with no serious adverse events (See **Summary of observational studies assessing immunologic or clinical outcomes of infants exposed to biologics in utero who were given inactivated vaccines)**.[3,4,6,8,16-18] Immunophenotyping is a surrogate outcome for immunosuppression which in turn is a surrogate outcome for potential adverse events related to administration of live vaccines. **The certainty of evidence was downgraded to <u>very low</u> due to study limitations, indirectness and imprecision.**

Live vaccines are first encountered by infants in the US and Canada at 1 year of age (varicella, mumps-measles-rubella) at which point infliximab or adalimumab concentrations should be undetectable. However, rotavirus live vaccine is given orally within 15 weeks of age. Despite its mode of administration and being significantly attenuated, there is very little data on the safety of this vaccine in this setting especially if either infliximab or adalimumab concentrations may be present. In other countries, infants may be exposed to other live vaccines in the first 6 months including BCG, oral polio and small pox. **Because it is not possible to predict**

the necessary anti-TNF concentration associated with an adverse immune reaction to a live vaccine in anti-TNF exposed infants (as this has not been systematically assessed), the data suggests that live vaccinations should be postponed until 6 months of age or after documented clearance of the drug in the child. As detectable certolizumab levels in the newborn are minimal and there is no detectable transfer in breast milk, live vaccines may be given to certolizumab exposed infant on schedule. There is no data on the clearance of other biologics (e.g. ustekinumab or vedolizumab) in exposed infants.

Risk of Bias Table

Cohort studies							
**Study**	**Valid methods to ascertain exposure**	**Prognostic factors (other than exposure of interest) similar among cohorts – or cohorts were adjusted adequately for confounders**	**Demonstration that outcome of interest was not present at the start of the study**	**Outcome detection methods valid and similar among cohorts**	**Follow-up complete and similar among cohorts**	**Free of other bias**	**Comments**
Moens 2019 (Belgium)	OK	No	OK	OK	OK	Possible selection bias as 9/20 received Rotavirus vaccination.	• Retrospective cohort study of 23 live births exposed to **vedolizumab** in utero<br>• Complications were observed in 25% of pregnancies and in 35% of infants (but confounded by disease activity and no control group)<br>• **9/20 old enough newborns received Rotavirus vaccination with no adverse reactions**.
Duricova 2019 (Czech Republic)	OK	No	OK	No<br><br>Recall bias<br><br>Different follow-ups of exposed	No<br><br>Serologic response to non-live and live vaccines was assessed	Possible selection bias	• Retrospective cohort study comparing 72 children (> 12 mos of age) born to mothers with IBD treated with anti-TNF during pregnancy vs. 69 unexposed children of non-IBD mothers

				children and controls due to inclusion of the control group later during the study course.<br><br>The control group with assessment of serologic response was more than twice as old as the exposed group.	in 68.1% and 51.4% exposed children at a median of 34.0 and 38.7 months. 23.2% controls had measurement of post-vaccine antibody titers to both non-live and live vaccines at a median age of 80.4 months.		• > 95% of exposed children have adequate serologic response to vaccination, except of Hib and mumps<br>• No children received Rotavirus vaccine<br>• **15 received BCG < 1 week old – no SAEs**
De Lima 2018 (Netherlands)	OK	No	OK	OK	OK	Possible selections as low participation rate – healthy volunteer effect	• Retrospective cohort study comparing 15 children born to mothers with IBD receiving anti-TNF vs. 12 children born to mothers with IBD not on anti-TNF<br>• No difference in seroprotection to Hep B between groups
Beaulieu 2018 (PIANO registry in US)	OK	No	OK	OK	OK	Possible selection bias – healthy volunteer effect	• Retrospective cohort study comparing 42 children born to women with IBD on biologics (27 IFX, 7 ADA, 3 certolizumab, 2 natalizumab, 2 ustekinumab, 1 vedolizumab) in the PIANO registry vs 8 children born to women not on biologic (IM alone or no IM)<br>• Serologic response to tetanus or HiB vaccines similar

							between exposed and unexposed infants, but overall rates lower than historical healthy control<br>• **39 Infants born to women on biologics received Rotavirus vaccine, no SAEs, 17.5% reaction with fever/diarrhea**. **No correlation between infant drug concentration at birth and likelihood of reaction to rotavirus vaccine.**
Julsgaard 2016 (Denmark, Australia and New Zealand)	OK	OK. Accounted for factors influencing drug concentration at the time of birth: weeks since last anti-TNF used, duration of anti-TNF, mesalamine use, thiopurine use, maternal weight before pregnancy, child weight, gestational week of birth, type of IBD, use of a second anti-TNF.	OK	OK	OK	OK	• Prospective cohort study of 80 mothers with IBD who received ADA and IFX during pregnancy and their 80 infants<br>• Pregnant mothers with IBD: 55% IFX and 45% ADA. 49% concurrent thiopurine. Last dose anti-TNF was at median GW 35 (14-41) for ADA and GW 30 (8-37) for IFX.<br>• **ADA exposed infants**: 8(22%) had undetectable drug level at birth in cord blood. **Mean time to clearance 4 mos (2.9-5.0 mos). All had undetectable level at 9 mos. Mean half life 26 d (23-29 d)**<br>• **IFX exposed infants:** all had detectable drug level at birth in cord blood. **Mean time to clearance 7.3 mos (6.2-8.3)** showing a 26% slower clearance of anti-TNF in IFX exposed vs. ADA exposed infants. **5 (11%) had**

							**detectable drug level at 9 mos. 1 had detectable drug (0.03ug/mL) at 12 mos. Mean half-life 33 d (30-37**)<br>• No association between drug half-life and birth weight, cord blood concentration, or maternal breastfeeding.
Sheibani 2016 (PIANO registry in US)	OK	No	OK	OK	OK	Unclear how the infants were selected (the denominator)	• Prospective cohort study of 12 infants exposed to anti-TNF (10 IFX, 2 ADA)<br>• Adequate response to both tetanus and Hib vaccines in 92%
Bortlik 2014 (Czech Republic)	OK	No	OK	OK	OK	Possible selection bias. Consecutive children exposed to anti-TNF in utero for maternal IBD treated with biologics were included. But inly 60% received BCG and serological response was assessed in 60%.	• Cohort study of 25 children aged $\geq$ 12 mos exposed to anti-TNFs prenatally for maternal IBD<br>• 60% received **BCG**, no serious adverse events<br>• 60% received other vaccines (including **mumps)** with serological response to mumps, tetanus, S. Pneumoniae, diphtheria, rubella, morbilli, and parotitis
Mahadevan 2013 (PIANO registry in US)	OK	No	OK	OK	OK	Possible selection bias. Mothers who agreed to participate	• Cohort study of 31 pregnant women with CD receiving IFX (11), ADA (10), or CZP (10) and their 33 infants

						may systematically different from those who did not agree to participate in the study.	• IFX: median ratio of cord to maternal drug level 160% (87-400%), took 2 – 7 mos to become undetectable<br>• ADA: median ratio of cord to maternal drug level 179% (98-293%), detectable for at least 11 weeks from birth<br>• CZP: drug < 2ug/mL in infants, median ratio of cord to maternal drug level 3.9% (1.5-24).

ADA: Adalimumab
CZP: Certolizumab
IFX: infliximab
IM: immunomodulators
SAEs: serious adverse events

Evidence Profile Table

Certainty Assessment								Summary of Findings	
Studies	Risk of bias	Inconsistency	Indirectness	Imprecision	Other considerations	Certainty of Evidence	Overall Certainty of evidence	Study Event Rates	Relative Effect (95% CI)
**Serious adverse events - CRITICAL**							⨁⊖⊖⊖ VERY LOW		
7 Observational studies[1-7] (cohort studies and case series)	Serious[a]	Not serious[b]	Serious[c]	Very Serious[d]	None	⨁⊖⊖⊖ **VERY LOW**		• See **Summary of observational studies assessing immunologic or clinical outcomes of infants exposed to biologics in utero who were given live vaccines**	
**Anti-TNF drug clearance in exposed infants - IMPORTANT**									
3 Observational studies[8-10] (cohort studies)	Serious[e]	Not serious	Very serious[f]	Serious[d]	None	⨁⊖⊖⊖ **VERY LOW**		• Median ratio of infant: mother drug concentration at birth 1.21 (0.94-1.49) for ADA and 1.97 (1.50-2.43) for IFX.[10] Median ratio of cord to maternal CZP level was 3.9%[9]	

								• Mean time to drug clearance 4.0 mos (2.9-5.0) for ADA and 7.3 mos (6.2-8.3) for IFX[10]<br>• Drugs were not detected in infants after 12 mos of age[10]<br>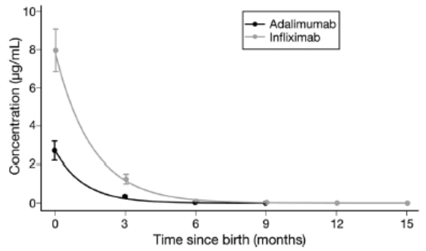<br><br>**Figure 4.** Mean (±SEM) and fitted neonatal clearance of adalimumab and infliximab.<br>From Julsgaard 2016
**Immunophenotyping in anti-TNF exposed infants - IMPORTANT**								
2<br>Observational studies[5,15]<br>(cohort studies)	Serious[e]	Not serious	Very serious[g]	Serious[d]	None	⊕⊖⊖⊖<br>**VERY LOW**		• Anti-TNF exposed infants had more immature B- and helper T phenotype at birth[5] that normalized by 12 months.[5,15]<br>• Decreased response after mycobacterial challenge[5]

ADA: Adalimumab
CZP: Certolizumab
IFX: Infliximab

Footnotes:

a. Downgraded for study limitations. Included studies were either case series or small cohort studies with no control group or adjustment of confounding factors (e.g. disease severity, other medication use etc). Possible selection bias as it was unclear how some biologic-exposed infants were given live vaccines despite recommendations against the practice. Presumably, "healthier" infants were selectively given live vaccines.
b. Not downgraded for inconsistency as most studies showed no serious adverse events except 1 reported death after administration of BCG vaccine to a 3-month old infant.
c. Downgraded for indirectness given a variety of live vaccines and different biologics (mostly anti-TNF) were included. The infants were also diverse in terms of baseline characteristics (e.g. pre-term birth weight, complications experienced during pregnancy and delivery, and other risk factors) that may predispose them to experience vaccine-related adverse effects.
d. Downgraded for imprecision (very small sample size).
e. Downgraded for study limitations. Included studies were either case series or small cohort studies with no control group or adjustment of confounding factors (e.g. disease severity, other medication use etc). Possible selection bias as mothers who agreed to participate in the study may be systematically different than those who did not agree to participate.

f. Downgraded for indirectness. Anti-TNF level detected in exposed infants is a surrogate outcome for immunosuppression which in turn is a surrogate outcome for potential adverse events related to administration of live vaccines.
g. Downgraded for indirectness. Immunophenotyping in exposed infants is a surrogate outcome for immunosuppression and therefore potential adverse events related to administration of live vaccines.

Summary of observational studies assessing immunologic or clinical outcomes of infants exposed to biologics in utero who were given live vaccines

Study	Number of infants who received live vaccines	Types of anti-TNF Used by mothers	Last dose of biologic given	Vaccination response				
				BCG	Rotavirus	Mumps	Measles	Rubella
Moens 2019 (Belgium) Cohort	9 Rotavirus	VED	First or second trimester	-	No adverse reactions	-	-	-
Lee 2019 (Korea) Case series	4 BCG < 6 mos<br><br>**4 Rotavirus < 6 mos**<br><br>(7 received live vaccines)	89% IFX<br><br>11% ADA	22 to 32 weeks of GA	No specific side effects	No specific side effects	-	-	-
Duricova 2019 (Czech Republic) Cohort	15 BCG < 1st week<br><br>37 MMR first dose at 15th month, second dose 6 – 10 mos later.	75% IFX<br><br>25% ADA	Median 29 weeks of GA	No serious adverse events	-	75% had adequate serologic response<br><br>(no difference than healthy controls)<br><br>No serious adverse events	100% had adequate serologic response<br><br>(no difference than healthy controls)<br><br>No serious adverse events	100% had adequate serologic response<br><br>(no difference than healthy controls)<br><br>No serious adverse events

Beaulieu 2018 (US) Cohort	**39 Rotavirus 2, 4, and 6 mos**	48.7% IFX 17.9% ADA 30.8% CZP 2.6% UST		-	No serious adverse events<br><br>17.5% mild reaction (fever, diarrhea) IFX and ADA<br><br>Comparable to rates seen in GP	-	-	-
Esteve-Sole 2017 (Spain) Case series	**4 Rotavirus**	ADA or IFX	≤ 7 days before delivery	-	No serious adverse events	-	-	-
Bortlik 2014 (Czech Republic) Cohort	15 BCG < 1 week<br><br>15 rubella 15 mos	IFX	Mean 23 weeks of GA	No serious adverse events<br><br>Large local reaction in 20%	-	-	-	100% had detectable antibodies, no infection
Cheent 2010 (UK)	1 BCG 3 mos	IFX	-	Died of disseminated mycobacterial infection	-	-	-	-

ADA: Adalimumab
BCG: Bacillus Calmette-Guerin vaccine
CZP: Certolizumab
GA: gestational age
GP: general population
IFX: Infliximab
NAT: Natalizumab
UST: Ustekinumab
VED: Vedolizumab

Summary of observational studies assessing immunologic or clinical outcomes of infants exposed to biologics in utero who were given inactivated vaccines

Study	Number of infants who received vaccines	Types of anti-TNF Used by mothers	Last dose of biologic given	Vaccination response				
				Hep B	Hib	Tetanus	Diphtheria	Streptococcus pneumoniae
Lee 2019 (Korea) Case series	12 Hep B 0, 1, 6 mos of life	89% Infliximab<br><br>11% Adalimumab	22 to 32 weeks of GA	66.7% Seroconversion<br><br>100% seroconversion after 1 booster	-	-	-	-
Duricova 2019 (Czech Republic) Cohort	72 hexavalent non-live vaccine (Hep B, Hib, diphtheria, tetanus, pertussis, and inactivated polio) within the 1st year of life, booster between 12-18 mos	75% Infliximab<br><br>25% Adalimumab	Median 29 weeks of GA	-	65.3% adequate serologic response<br><br>(higher than healthy control 12.5%)	95.9% adequate serologic response<br><br>(higher than healthy control 62.5%)	98.0% adequate serologic response<br><br>(no different than healthy control 87.5%)	97.8% adequate serologic response<br><br>(higher than healthy controls 68.8%)
De Lima 2018 (Netherlands) Cohort	15 HBV 6 weeks, 3, 4 and 11 mos	53% Infliximab 47% Adalimumab	Median 25 weeks of GA for infliximab<br><br>Median 23 weeks of GA	100% seroconversion<br><br>No SAEs	-	-	-	-

			for Adalimumab					
Beaulieu 2018 (US) Cohort	46 Hib 2, 4 and 6 mos<br><br>49 tetanus 2, 4, and 6 mos	64% IFX 17% ADA 7% CZP 5% NAT 5% UST 2% VED	-	-	71% adequate antibody titers<br><br>(no different than unexposed infants born to IBD mothers 50%, but lower than historical healthy controls 90-100%)	80% adequate antibody titers<br><br>(no different than unexposed infants born to IBD mothers 75%, but lower than historical healthy controls 90-100%)	-	-
Sheibani 2016 (US)	12 Hib, tetanus 0 to 6 mos	83% IFX 17% ADA	-	-	92% adequate serologic response<br><br>(compared to 95% standard seroprotection rates)	92% adequate serologic response<br><br>(compared to 100% standard seroprotection rates)	-	-
Bortlik 2014 (Czech Republic) Cohort	15 tetanus, S. pneumoniae, diphtheria, rubella, morbilli, and parotitis 2-18 mos	88% IFX 12% ADA	Mean 26 weeks GA	-	60% adequate serological response	Detectable antibodies	Detectable antibodies	Detectable antibodies
Zelinkova 2011 (Netherlands) Case series	4 diphtheria, tetanus, pertusus, and polio, Hib, pneumococcus age 2, 3 and 4	IFX	21-30 weeks GA	-	100% seroprotection	-	-	100% seroprotection

	mos (seroresponse tested in 2/3 children born with detectable IFX levels)							

References:

1. Moens A, van Hoeve K, Humblet E, Rahier JF, Bossuyt P, Dewit S, Franchimont D, Macken E, Nijs J, Posen A, Strubbe B, Van Hootegem A, Van Moerkercke W, Vermeire S, Ferrante M; Belgian IBD Research and Development group (BIRD). Outcome of Pregnancies in Female Patients With Inflammatory Bowel Diseases Treated With Vedolizumab.
2. Lee KE, Jung SA, Park SH, Moon CM, Shim SY, Kim ES, Cho SJ, Kim SE, Cho KB, Yang SK. Influence of anti-tumor necrosis factor-alpha therapy to pregnant inflammatory bowel disease women and their children's immunity. Intest Res. 2019 Apr;17(2):237-243.
3. Duricova D, Dvorakova E, Hradsky O, Mitrova K, Durilova M, Kozeluhova J, Kohout P, Zarubova K, Bronsky J, Hradska N, Bronska E, Adamcova M, Machkova N, Hruba V, Bortlik M, Lukas M, Malickova K, Lukas M. Safety of Anti-TNF-Alpha Therapy During Pregnancy on Long-term Outcome of Exposed Children: A Controlled, Multicenter Observation. Inflamm Bowel Dis. 2019 Mar 14;25(4):789-796.
4. Beaulieu DB, Ananthakrishnan AN, Martin C, Cohen RD, Kane SV, Mahadevan U. Use of Biologic Therapy by Pregnant Women With Inflammatory Bowel Disease Does Not Affect Infant Response to Vaccines. Clin Gastroenterol Hepatol. 2018 Jan;16(1):99-105.
5. Esteve-Solé A, Deyà-Martínez À, Teixidó I, Ricart E, Gompertz M, Torradeflot M, de Moner N, Gonzalez EA, Plaza-Martin AM, Yagüe J, Juan M, Alsina L. Immunological Changes in Blood of Newborns Exposed to Anti-TNF-α during Pregnancy. Front Immunol. 2017 Sep 21;8:1123.
6. Bortlik M, Duricova D, Machkova N, Kozeluhova J, Kohout P, Hrdlicka L, Durilova M, Mitrova K, Hradsky O, Bronsky J, Malickova K, Lukas M. Impact of anti-tumor necrosis factor alpha antibodies administered to pregnant women with inflammatory bowel disease on long-term outcome of exposed children. Inflamm Bowel Dis. 2014 Mar;20(3):495-501.
7. Cheent K, Nolan J, Shariq S, Kiho L, Pal A, Arnold J. Case Report: Fatal case of disseminated BCG infection in an infant born to a mother taking infliximab for Crohn's disease. J Crohns Colitis. 2010 Nov;4(5):603-5.
8. Zelinkova Z, de Haar C, de Ridder L, Pierik MJ, Kuipers EJ, Peppelenbosch MP, van der Woude CJ. High intra-uterine exposure to infliximab following maternal anti-TNF treatment during pregnancy. Aliment Pharmacol Ther. 2011 May;33(9):1053-8.
9. Mahadevan U, Wolf DC, Dubinsky M, Cortot A, Lee SD, Siegel CA, Ullman T, Glover S, Valentine JF, Rubin DT, Miller J, Abreu MT. Placental transfer of anti-tumor necrosis factor agents in pregnant patients with inflammatory bowel disease. Clin Gastroenterol Hepatol. 2013 Mar;11(3):286-92.
10. Julsgaard M, Christensen LA, Gibson PR, Gearry RB, Fallingborg J, Hvas CL, Bibby BM, Uldbjerg N, Connell WR, Rosella O, Grosen A, Brown SJ, Kjeldsen J, Wildt S, Svenningsen L, Sparrow MP, Walsh A, Connor SJ, Radford-Smith G, Lawrance IC, Andrews JM, Ellard K, Bell SJ. Concentrations of Adalimumab and Infliximab in Mothers and Newborns, and Effects on Infection. Gastroenterology. 2016 Jul;151(1):110-9.
11. Ben-Horin S, Yavzori M, Kopylov U, Picard O, Fudim E, Eliakim R, Chowers Y, Lang A. Detection of infliximab in breast milk of nursing mothers with inflammatory bowel disease. J Crohns Colitis. 2011 Dec;5(6):555-8.

12. Ben-Horin S, Yavzori M, Katz L, Picard O, Fudim E, Chowers Y, Lang A. Adalimumab level in breast milk of a nursing mother. Clin Gastroenterol Hepatol. 2010 May;8(5):475-6
13. Lahat A, Shitrit AB, Naftali T, Milgrom Y, Elyakim R, Goldin E, Levhar N, Selinger L, Zuker T, Fudim E, Picard O, Yavzori M, Ben-Horin S. Vedolizumab Levels in Breast Milk of Nursing Mothers With Inflammatory Bowel Disease. J Crohns Colitis. 2018 Jan 5;12(1):120-123.
14. Julsgaard M, Kjeldsen J, Bibby BM, Brock B, Baumgart DC. Vedolizumab Concentrations in the Breast Milk of Nursing Mothers With Inflammatory Bowel Disease. Gastroenterology. 2018 Feb;154(3):752-754.e1.
15. Kattah MG, Milush JM, Burt T, McCabe RP Jr, Whang MI, Ma A, Mahadevan U. Anti-TNF and thiopurine therapy in pregnant IBD patients does not significantly alter a panel of B-cell and T-cell subsets in 1-year-old infants. Clin Transl Gastroenterol. 2018 Apr 3;9(4):143.
16. Lee KE, Jung SA, Park SH, Moon CM, Shim SY, Kim ES, Cho SJ, Kim SE, Cho KB, Yang SK. Influence of anti-tumor necrosis factor-alpha therapy to pregnant inflammatory bowel disease women and their children's immunity. Intest Res. 2019 Apr;17(2):237-243.
17. de Lima A, Kanis SL, Escher JC, van der Woude CJ. Hepatitis B Vaccination Effective in Children Exposed to Anti-Tumour Necrosis Factor Alpha in Utero. J Crohns Colitis. 2018 Jul 30;12(8):948-953.
18. Sheibani S, Cohen R, Kane S, Dubinsky M, Church JA, Mahadevan U. The Effect of Maternal Peripartum Anti-TNFα Use on Infant Immune Response. Dig Dis Sci. 2016 Jun;61(6):1622-7.

Evidence to Decision Table

PICO	In infants born of mother using biological therapies, should live vaccines vs. no live vaccines be given in the first 6 months of life?
Population	Infants born of mother using biological therapies
Intervention	Administration of live vaccines in the first 6 months of life
Comparator	No administration of live vaccines in the first 6 months of life
Outcome	Mortality, VPI, SAEs, Immunogenicity

	Judgement	Research evidence	Additional considerations
Desirable Effects	**How substantial are the desirable anticipated effects?**<br><br>○ Trivial<br>○ Small<br>○ Moderate<br>○ Large	Because live vaccines may cause disease by uncontrolled replication in immunocompromised persons, the literature review for this PICO question focused on **safety outcomes.**<br><br>**Serious adverse events to live vaccines in biologic-exposed infants**	Panel can make recommendations for either live vaccines in general or rotavirus only as the evidence was reviewed for all live vaccines.

	○ Varies<br>○ Don't know	Literature review found 7 observational studies (small cohort and case series) of infants exposed to biologic agents in utero who were given live vaccines (56 infants received rotavirus vaccine at less than 6 months of age, 74 received BCG vaccine within 6 months of age, 52 received MMR or rubella vaccine at 15 months of age).[1-7] The most common biologics used during these pregnancies were anti-TNF agents (infliximab followed by adalimumab and certolizumab). Vedolizumab and Ustekinumab were used in a small minority of cases. Most of these biologics were stopped either in the second or third trimester. No serious adverse events were experienced by any of the infants except a death attributed to disseminated BCG infection after administration of the BCG vaccine to a 3-month old infant exposed in utero to infliximab.[7] The GRADE rating started at low due to the observational designs of these studies. **The certainty of evidence was downgraded to <u>very low</u> due to study limitations, inconsistency, indirectness and imprecision.** (See **Summary of observational studies assessing immunologic or clinical outcomes of infants exposed to biologics in utero who were given live vaccines)**<br><br>**Anti-TNF drug clearance in exposed infants**<br><br>Anti-TNF agents are IgG1 antibodies that cross the placenta by active transport starting at the end of the second trimester but mainly in the third trimester. It has therefore been common practice to discontinue infliximab and adalimumab during the second or third trimester to reduce the transplacental transfer of these drugs to the fetus. In contrast, certolizumab pegol, by virtue of being a PEG (polyethylene glycolylated) FaB immunoglobulin, does not bind to the neonatal FcY receptor responsible for active transplacental transfer.<br><br>Three observational studies have shown that both infliximab and adalimumab can be detected in the cord blood at delivery even when these drugs were stopped in the second or third trimester, usually in levels higher than those in maternal serum (about 1.5 to 2-fold higher).[8-10] Additionally, Julsgaard et al. demonstrated that infliximab concentration could persist for up to 12 months (in 1/80 biologic exposed infants) and adalimumab for up to 6 months after birth.[10] The average half-life of anti-TNF in exposed infants was longer than in adult non-pregnant patients: adalimumab was 26 days (95% CI 23-29 days) and infliximab was 33 days (95% CI 30-37 days).[10] The mean time to drug clearance in infants was 4.0 months for adalimumab (95% CI 2.9-5.0 and 7.3 months for infliximab (95% CI 6.2-8.3). Certolizumab was detachable at minimal levels in infant or cord blood likely due to passive diffusion.[9] It is important to note that previous studies have found insignificant amounts of anti-TNF and vedolizumab in breast milk of nursing IBD patients, and are unlikely to result in systemic immunosuppression of the infant.[11-14] In the study by Julsgaard et al, no statistically significant associations between drug half-life and maternal breastfeeding was found.[10] The GRADE rating started at low due to the observational designs of these studies. Anti-TNF level detected in exposed infants is a surrogate outcome for immunosuppression which in turn is a surrogate outcome for potential adverse events related to administration of live vaccines. **The certainty of evidence was downgraded to <u>very low</u> due to study limitations, indirectness and imprecision.**	
**Undesirable Effects**	**How substantial are the undesirable anticipated effects?**<br><br>○ Large<br>○ Moderate<br>○ Small<br>○ Trivial<br><br>○ Varies depending on jurisdictions<br>○ Don't know		

		**Immunophenotyping in anti-TNF exposed infants**<br>Finally, immunophenotyping studies have shown that anti-TNF exposed infants had more immature B- and helper T phenotype at birth[5] that normalized by 12 months.[5,15] A decreased response after mycobacterial challenge was noted in 1 study.[5] Observational studies have found that infants exposed to anti-TNF in utero have appropriate response to inactivated vaccines with no serious adverse events (See **Summary of observational studies assessing immunologic or clinical outcomes of infants exposed to biologics in utero who were given inactivated vaccines)**.[3,4,6,8,16-18] Immunophenotyping is a surrogate outcome for immunosuppression which in turn is a surrogate outcome for potential adverse events related to administration of live vaccines. **The certainty of evidence was downgraded to <u>very low</u> due to study limitations, indirectness and imprecision.**<br><br>Live vaccines are first encountered by infants in the US and Canada at 1 year of age (varicella, mumps-measles-rubella) at which point infliximab or adalimumab concentrations should be undetectable. However, rotavirus live vaccine is given orally within 15 weeks of age. Despite its mode of administration and being significantly attenuated, there is very little data on the safety of this vaccine in this setting especially if either infliximab or adalimumab concentrations may be present. In other countries, infants may be exposed to other live vaccines in the first 6 months including BCG, oral polio and small pox. **Because it is not possible to predict the necessary anti-TNF concentration associated with an adverse immune reaction to a live vaccine in anti-TNF exposed infants (as this has not been systematically assessed), the data suggests that live vaccinations should be postponed until (panel to decide 6 or 12 months of age after assessing the Julsgaard 2016 study), or after documented clearance of the drug in the child.** As detectable certolizumab levels in the newborn are minimal and there is no detectable transfer in breast milk, live vaccines may be given to certolizumab exposed infant on schedule. There is no data on the clearance of other biologics (e.g. ustekinumab or vedolizumab) in exposed infants.	
**Certainty of evidence**	**What is the overall certainty of the evidence of effects?**<br><br>○ Very low<br>○ Low<br>○ Moderate<br>○ High<br><br>○ No included studies		
**and Prefere nces**	**Is there important uncertainty about or variability in how much people value the main outcomes?**	Parents likely value the main outcomes of adverse events (potential mortality) related to administration of live vaccines more than other surrogate outcomes (drug clearance	

	o Important uncertainty or variability<br>o Possibly important uncertainty or variability<br>o Probably no important uncertainty or variability<br>o No important uncertainty or variability	or immunophenotyping) or potential effectiveness of the vaccines.	
**Balance of effects**	**Does the balance between desirable and undesirable effects favor the intervention or the comparison?**<br><br>o Favors the comparison<br>o Probably favors the comparison<br>o Does not favor either the intervention or the comparison<br>o Probably favors the intervention<br>o Favors the intervention<br><br>o Varies<br>o Don't know		
**Resources required**	**How large are the resource requirements (costs)?**<br><br>o Large costs<br>o Moderate costs<br>o Negligible costs and savings<br>o Moderate savings<br>o Large savings<br><br>o Varies<br>o Don't know	CDC vaccine price list last reviewed/updated: July 1, 2019<br><br><table><tr><th>Brand name</th><th>CDC cost/dose</th><th>Private sector cost/dose</th></tr><tr><td>Rotateq®</td><td>$70.49</td><td>$84.53</td></tr><tr><td>Rotarix®</td><td>$94.69</td><td>$120.95</td></tr><tr><td>MMR®</td><td>$21.22</td><td>$78.68</td></tr><tr><td>ProQuad®</td><td>$131.40</td><td>$224.94</td></tr><tr><td>Varivax®</td><td>$104.09</td><td>$135.73</td></tr></table>	
**Certainty of Evidence of Required Resources**	**What is the certainty of the evidence of resource requirements (costs)?**<br><br>o Very low<br>o Low<br>o Moderate<br>o High<br><br>o No included studies	The costs of delivering routine immunization services may vary widely across countries and different health system settings. See Immunization Costing Action Network (ICAN) Immunization Delivery Cost Catalogue. http://immunizationeconomics.org/ican-idcc.	

**Cost effectiveness**	**Does the cost-effectiveness of the intervention favor the intervention or the comparison?**<br><br>○ Favors the comparison<br>○ Probably favors the comparison<br>○ Does not favor either the intervention or the comparison<br>○ Probably favors the intervention<br>○ Favors the intervention<br><br>○ Varies<br>○ No included studies	Rotavirus is the most common cause of gastroenteritis among children younger than 5 years of age and results in significant morbidity and resource utilization, although mortality due to rotavirus is rare in Canada or in the US.<br><br>A cost effectiveness analysis was conducted through a Markov model that followed a cohort of children from birth to 5 years of age in Canada.[19] **Because the majority of rotavirus infections do not require emergency department visits or hospital admission, from a health care system perspective, a routine vaccination program for rotavirus would not be considered cost-effective in Canada.** The incremental cost per QALY gained from the health care system perspective was $122,000 for RotaTeq and $108,000 for Rotarix. From a societal perspective, a cost effectiveness analysis found a universal vaccination program against rotavirus in Canada to be both cost-saving and more effective than no vaccination.<br><br>A cost-effectiveness analysis assessed the impact of a national rotavirus immunization program in the US by using a hypothetical US birth cohort of 4,010.000 children.[20] Routine rotavirus immunization would prevent 13 deaths, 44,000 hospitalizations, 137,000 emergency department visits, 256,000 office visits, and 1,100,000 episodes requiring only home care. It concluded that **routine rotavirus vaccination would unlikely be cost-saving in the US.**[20]<br><br>A systematic review of global economic evaluations of rotavirus vaccine found that **mass vaccination against rotavirus was generally cost-effective (cost-saving to highly cost-effective), particularly in low- and middle-income setting**s according to the external subsidization of vaccine price.[21] On the other hand, it **may not be a cost-effective intervention at market price in some high-income settings**.[21]<br><br>**A critical literature review of 68 health economic evaluations of rotavirus vaccination found rotavirus vaccination to be cost-effective in developing countries, while conclusions varied between studies in developed countries.**[22] Many studies found that vaccination was likely to be cost-effective under some scenarios, such as lower prices, inclusion of herd protection, and/or adoption of societal perspective. Other reasons for variability included uncertainty around healthcare visits incidence and lack of consensus on quality of life valuation for infants and caregivers.	

**Acceptability**	**Is the intervention acceptable to key stakeholders?**<br><br>○ No<br>○ Probably no<br>○ Probably yes<br>○ Yes<br><br>○ Varies<br>○ Don't know	In a qualitative study of factors influencing acceptance of rotavirus vaccine among healthcare providers and consumers, acceptability was variable among consumers.[23] Parents generally deemed the vaccine to be acceptable and most of them reported that they would rely on their health care provider's recommendation. However, many parents did not consider the disease to be a high-priority health issue for children.[23] Commonly expressed concerns about the vaccine included the administration of a live-vaccine to "young, vulnerable" infants, the potential for adverse events, and the narrow window of age when the vaccine is recommended.[23]<br><br>In a Canadian study assessing the determinants of parents' decision to vaccinate their children against rotavirus, more than 70% of parents held very positive general attitudes about vaccination.[24] However, only 35% had a very strong intention to have their child vaccinated against rotavirus.[24] This could be explained, at least partially, by the fact that rotavirus vaccines were relatively new at that time (2008-09) and that most parents perceived rotavirus gastroenteritis as only a moderately severe disease.[24]	
**Feasibility**	**Is the intervention feasible to implement?**<br><br>○ No<br>○ Probably no<br>○ Probably yes<br>○ Yes<br><br>○ Varies<br>○ Don't know		

References:

1. Moens A, van Hoeve K, Humblet E, Rahier JF, Bossuyt P, Dewit S, Franchimont D, Macken E, Nijs J, Posen A, Strubbe B, Van Hootegem A, Van Moerkercke W, Vermeire S, Ferrante M; Belgian IBD Research and Development group (BIRD). Outcome of Pregnancies in Female Patients With Inflammatory Bowel Diseases Treated With Vedolizumab.
2. Lee KE, Jung SA, Park SH, Moon CM, Shim SY, Kim ES, Cho SJ, Kim SE, Cho KB, Yang SK. Influence of anti-tumor necrosis factor-alpha therapy to pregnant inflammatory bowel disease women and their children's immunity. Intest Res. 2019 Apr;17(2):237-243.
3. Duricova D, Dvorakova E, Hradsky O, Mitrova K, Durilova M, Kozeluhova J, Kohout P, Zarubova K, Bronsky J, Hradska N, Bronska E, Adamcova M, Machkova N, Hruba V, Bortlik M, Lukas M, Malickova K, Lukas M. Safety of Anti-TNF-Alpha Therapy During Pregnancy on Long-term Outcome of Exposed Children: A Controlled, Multicenter Observation. Inflamm Bowel Dis. 2019 Mar 14;25(4):789-796.
4. Beaulieu DB, Ananthakrishnan AN, Martin C, Cohen RD, Kane SV, Mahadevan U. Use of Biologic Therapy by Pregnant Women With Inflammatory Bowel Disease Does Not Affect Infant Response to Vaccines. Clin Gastroenterol Hepatol. 2018 Jan;16(1):99-105.
5. Esteve-Solé A, Deyà-Martínez À, Teixidó I, Ricart E, Gompertz M, Torradeflot M, de Moner N, Gonzalez EA, Plaza-Martin AM, Yagüe J, Juan M, Alsina L. Immunological Changes in Blood of Newborns Exposed to Anti-TNF-α during Pregnancy. Front Immunol. 2017 Sep 21;8:1123.

6. Bortlik M, Duricova D, Machkova N, Kozeluhova J, Kohout P, Hrdlicka L, Durilova M, Mitrova K, Hradsky O, Bronsky J, Malickova K, Lukas M. Impact of anti-tumor necrosis factor alpha antibodies administered to pregnant women with inflammatory bowel disease on long-term outcome of exposed children. Inflamm Bowel Dis. 2014 Mar;20(3):495-501.
7. Cheent K, Nolan J, Shariq S, Kiho L, Pal A, Arnold J. Case Report: Fatal case of disseminated BCG infection in an infant born to a mother taking infliximab for Crohn's disease. J Crohns Colitis. 2010 Nov;4(5):603-5.
8. Zelinkova Z, de Haar C, de Ridder L, Pierik MJ, Kuipers EJ, Peppelenbosch MP, van der Woude CJ. High intra-uterine exposure to infliximab following maternal anti-TNF treatment during pregnancy. Aliment Pharmacol Ther. 2011 May;33(9):1053-8.
9. Mahadevan U, Wolf DC, Dubinsky M, Cortot A, Lee SD, Siegel CA, Ullman T, Glover S, Valentine JF, Rubin DT, Miller J, Abreu MT. Placental transfer of anti-tumor necrosis factor agents in pregnant patients with inflammatory bowel disease. Clin Gastroenterol Hepatol. 2013 Mar;11(3):286-92.
10. Julsgaard M, Christensen LA, Gibson PR, Gearry RB, Fallingborg J, Hvas CL, Bibby BM, Uldbjerg N, Connell WR, Rosella O, Grosen A, Brown SJ, Kjeldsen J, Wildt S, Svenningsen L, Sparrow MP, Walsh A, Connor SJ, Radford-Smith G, Lawrance IC, Andrews JM, Ellard K, Bell SJ. Concentrations of Adalimumab and Infliximab in Mothers and Newborns, and Effects on Infection. Gastroenterology. 2016 Jul;151(1):110-9.
11. Ben-Horin S, Yavzori M, Kopylov U, Picard O, Fudim E, Eliakim R, Chowers Y, Lang A. Detection of infliximab in breast milk of nursing mothers with inflammatory bowel disease. J Crohns Colitis. 2011 Dec;5(6):555-8.
12. Ben-Horin S, Yavzori M, Katz L, Picard O, Fudim E, Chowers Y, Lang A. Adalimumab level in breast milk of a nursing mother. Clin Gastroenterol Hepatol. 2010 May;8(5):475-6
13. Lahat A, Shitrit AB, Naftali T, Milgrom Y, Elyakim R, Goldin E, Levhar N, Selinger L, Zuker T, Fudim E, Picard O, Yavzori M, Ben-Horin S. Vedolizumab Levels in Breast Milk of Nursing Mothers With Inflammatory Bowel Disease. J Crohns Colitis. 2018 Jan 5;12(1):120-123.
14. Julsgaard M, Kjeldsen J, Bibby BM, Brock B, Baumgart DC. Vedolizumab Concentrations in the Breast Milk of Nursing Mothers With Inflammatory Bowel Disease. Gastroenterology. 2018 Feb;154(3):752-754.e1.
15. Kattah MG, Milush JM, Burt T, McCabe RP Jr, Whang MI, Ma A, Mahadevan U. Anti-TNF and thiopurine therapy in pregnant IBD patients does not significantly alter a panel of B-cell and T-cell subsets in 1-year-old infants. Clin Transl Gastroenterol. 2018 Apr 3;9(4):143.
16. Lee KE, Jung SA, Park SH, Moon CM, Shim SY, Kim ES, Cho SJ, Kim SE, Cho KB, Yang SK. Influence of anti-tumor necrosis factor-alpha therapy to pregnant inflammatory bowel disease women and their children's immunity. Intest Res. 2019 Apr;17(2):237-243.
17. de Lima A, Kanis SL, Escher JC, van der Woude CJ. Hepatitis B Vaccination Effective in Children Exposed to Anti-Tumour Necrosis Factor Alpha in Utero. J Crohns Colitis. 2018 Jul 30;12(8):948-953.
18. Sheibani S, Cohen R, Kane S, Dubinsky M, Church JA, Mahadevan U. The Effect of Maternal Peripartum Anti-TNFα Use on Infant Immune Response. Dig Dis Sci. 2016 Jun;61(6):1622-7.
19. Coyle D, Coyle K, Bettinger JA, Halperin SA, Vaudry W, Scheifele DW, Le Saux N. Cost effectiveness of infant vaccination for rotavirus in Canada. Can J Infect Dis Med Microbiol. 2012 Summer;23(2):71-7.
20. Widdowson MA, Meltzer MI, Zhang X, Bresee JS, Parashar UD, Glass RI. Cost-effectiveness and potential impact of rotavirus vaccination in the United States. Pediatrics. 2007 Apr;119(4):684-97.
21. Kotirum S, Vutipongsatorn N, Kongpakwattana K, Hutubessy R, Chaiyakunapruk N. Global economic evaluations of rotavirus vaccines: A systematic review.
22. Aballéa S, Millier A, Quilici S, Caroll S, Petrou S, Toumi M. A critical literature review of health economic evaluations of rotavirus vaccination. Hum Vaccin Immunother. 2013 Jun;9(6):1272-88.
23. Patel MM, Janssen AP, Tardif RR, Herring M, Parashar UD. A qualitative assessment of factors influencing acceptance of a new rotavirus vaccine among health care providers and consumers. BMC Pediatr. 2007 Oct 18;7:32.

24. Dubé E, Bettinger JA, Halperin B, Bradet R, Lavoie F, Sauvageau C, Gilca V, Boulianne N. Determinants of parents' decision to vaccinate their children against rotavirus: results of a longitudinal study. Health Educ Res. 2012 Dec;27(6):1069-80.

Conclusion

PICO: In infants born of mother using biological therapies, should live vaccines be given in the first 6 months of life?
Very low certainty evidence
Direction – Uncertain (67%), No (33%)
No consensus

**Type of recommendation**	**Strong recommendation against the intervention**<br>○	**Conditional recommendation against the intervention**<br>○	**Conditional recommendation for either the intervention or the comparison**<br>○	**Conditional recommendation for the intervention**<br>○	**Strong recommendation for the intervention**<br>○
**Recommendation**	**No recommendation. In infants born of mothers using biological therapies, the consensus group could not make a recommendation for or against giving live vaccines in the first 6 months of life.**				
**Justification**					
**Subgroup considerations**					
**Implementation considerations**					

**Monitoring and evaluation**	• Ongoing monitoring of serious adverse events associated with administration of live vaccines in biologic exposed infants (by types of biologic)
**Research priorities**	• More studies to determine the immunogenicity, efficacy, and safety of live vaccines (e.g. rotavirus vaccine) in biologic exposed infants (by types of biologic) with undetected drug level

INACTIVATED VACCINES

Haemophilus Influenzae type b (Hib)

Background

Prior to the introduction of *Haemophilus Influenzae* type b (Hib) vaccine in the United States (US), Hib was the leading cause of bacterial meningitis and a common cause of other invasive diseases (e.g. epiglottitis, pneumonia, septic arthritis, cellulitis, purulent pericarditis, and bacteremia) among US children aged < 5 years. Between 3% to 6% of Hib cases in children are fatal. Up to 20% of patients who survive Hib meningitis have permanent hearing loss or other severe permanent neurological sequelae. As a result of the introduction of routine childhood Hib vaccination and sustained high vaccine coverage, the annual incidence of invasive Hib disease in children aged < 5 years decreased by 99%, to less than one case per 100,000 during 1989-2000.[1] Clinical efficacy of Hib vaccination has been estimated at 95% to 100% in the general population.[2] The duration of immunity following vaccination is unknown, but data suggest that protection is long lasting.[2] Studies have suggested that long-term protection from invasive Hib disease is correlated with the presence of anti-purified polyribosylribitol phosphate (PRP) levels ≥ 0.15 ug/mL in unvaccinated children and anti-PRP levels ≥ 1.0 ug/mL in vaccinated children.[3]

Hib disease is uncommon in adults and in children aged > 5 years. In the US, adults aged > 65 years now account for the largest proportion of *Haemophilus influenzae* disease. The majority of cases in adults are caused by non-typeable *Haemophilus influenzae*

with an overall case fatality ratio (CER) of 19.5%. Persons with certain immunocompromising conditions are considered at risk for invasive Hib disease including those with functional or anatomic asplenia, HIV infection, immunoglobulin deficiency, early component complement deficiency, receipt of a hematopoietic stem cell transplant, or receipt of chemotherapy or radiation therapy for malignant neoplasms.[1]

Centers for Disease Control and Prevention (CDC) Advisory Committee on Immunization Practices (ACIP) recommends routine administration of Hib vaccine series beginning at age 2 months through 6 months of age with either a 3 dose-series of Hib PRP-T as ActHib®, Hiberix®, or Pentacel® or a 2-dose series of Hib PRP-OMP as PedvaxHib®.[1] A booster dose of conjugate Hib vaccine is recommended at age 12 through 15 months. Catch-up vaccination until 5 years of age is also recommended.

In unimmunized adults and children older than 5 years of age, Hib vaccine is recommended only for high-risk medical conditions for invasive Hib disease including those with anatomic or functional asplenia (e.g. sickle cell disease), HIV infection, immunoglobulin deficiency, early component complement deficiency, elective splenectomy, recipients of hematopoietic stem cell transplant, and those prior to receiving chemotherapy or radiation therapy for malignant neoplasms.[1] Hib vaccination is not recommended routinely for unvaccinated adults and children aged more than 5 years because, in the pre-vaccination era, invasive Hib disease affected almost exclusively children aged less than 5 years.[4] Healthy, unvaccinated adults have protective immunity against Hib due to natural anti-Hib antibodies that may have been induced by exposure to some common environmental bacteria that carry antigens cross-reacting with PRP.[5]

References:

1. Centers for Disease Control and Prevention (CDC). Prevention and Control of Haemophilus influenzae Type b Disease: Recommendations of the Advisory Committee on Immunization Practices (ACIP). MMWR 2014; 63 (No. 1)
2. Public Health Agency of Canada. https://www.canada.ca/en/public-health/services/publications/healthy-living/canadian-immunization-guide-part-4-active-vaccines/page-5-haemophilus-influenzae-type-b-vaccine.html
3. Käyhty H, Peltola H, Karanko V, Mäkelä PH. The protective level of serum antibodies to the capsular polysaccharide of Haemophilus influenzae type b. J Infect Dis. 1983 Jun;147(6):1100.
4. Peltola H. Worldwide Haemophilus influenzae type b disease at the beginning of the 21st century: global analysis of the disease burden 25 years after the use of the polysaccharide vaccine and a decade after the advent of conjugates. Clin Microbiol Rev. 2000 Apr;13(2):302-17.
5. Nix EB1, Hawdon N, Gravelle S, Biman B, Brigden M, Malik S, McCready W, Ferroni G, Ulanova M. Risk of invasive Haemophilus influenzae type b (Hib) disease in adults with secondary immunodeficiency in the post-Hib vaccine era. Clin Vaccine Immunol. 2012 May;19(5):766-71

Risk of Hib infection in IBD patients

PICO: What is the risk of Hib infection in people with IBD compared to people without IBD?

Summary

Adults

Only one observational study addressed this PICO question.[1] This was a cross-sectional case-control study that used an administrative database (Nationwide Inpatient Sample) to compare the risks of hospitalization for Hib pneumonia among adult IBD patients vs. non-IBD controls. It is important to note that Hib pneumonia patients treated as outpatients were excluded. After adjusting for various factors including comorbidities, risk factors for pneumonia, as well as patient and hospital characteristics, IBD patients had increased odds of being admitted for Hib pneumonia (aOR 1.34; CI 1.16-1.55) when compared to the non-IBD control group.[1] Mortality during these admissions among IBD patients was not significantly higher than the control population.[1] The GRADE rating started at high as it was considered a prognostic study (providing evidence about the likelihood of Hib pneumonia in patients with IBD). The rating was further downgraded to **very low** due to study limitations (residual confounding factors, detection bias, admission bias, and misclassification bias) and indirectness (admitted IBD patients with a primary diagnosis of Hib pneumonia, and not all IBD patients with Hib pneumonia). In particular, patients with IBD and pneumonia (or respiratory symptoms) may be more likely to be tested and admitted for Hib than non-IBD controls, thus creating an overestimate of the prevalence of Hib pneumonia among admitted IBD patients. **In summary, there is very low certainty evidence that adult IBD patients have an increased risk of Hib infection compared to non-IBD patients.**

Please also see meningococcal section for functional asplenia in IBD patients.

Pediatric

Literature search did not identify any study on the risk of Hib infection in pediatric IBD patients.

Risk of Bias Table

Prognostic studies							
**Study**	**Study sample adequately represents the population of interest**	**Study data available adequately represent the study sample (>80% follow-up)**	**Prognostic factor measured in a similar and valid way for all participants**	**Outcome of interest is measured in a similar and valid way for all participants**	**Important potential confounding factors are appropriately accounted for**	**Statistical analysis is appropriate, and all primary outcomes are reported**	**Comments**
Stobaugh 2013 (US)	Study included only hospitalized patients, and did not capture Hib pneumonia patients treated as outpatients. Prevalence-incidence (Neyman) bias: Exclusion of individuals with severe (fatal prior to admission) or mild HiB (not requiring admission) may result in a systematic error in the estimated association or effect of IBD on the risk of hospitalization for Hib.	OK	Data were reliant on administrative discharge diagnoses. Possible misclassification errors due to errors of miscoding, and the codes have not been previously validated.	Data were reliant on administrative discharge diagnoses. Possible misclassification errors due to errors of miscoding, and the codes have not been previously validated.<br><br>Detection bias and admission rate bias: patients with IBD and pneumonia may be more likely to be tested and admitted for Hib than controls, thus creating an overestimate of the prevalence of Hib pneumonia	Case-mix adjustment was performed using the updated Elixhauser Agency for Health-care Research and Quality-Web ICD-9-CM comorbidity algorithms, well-described risk factors for pneumonia, as well as patient and hospital characteristics.<br><br>Possible residual confounding factors: medication use, Hib vaccination status, severity and activity of underlying disease (e.g. sicker IBD patients on immunosuppressives may be more likely to be admitted than less sick IBD patients).	OK	• Cross-sectional case-control study (6-year analysis) on the Nationwide Inpatient Sample to assess the risk of hospitalizations for vaccine preventable pneumonias (HiB) among IBD patients vs. non-IBD patients<br>• Cases: All adult patients hospitalized with a secondary diagnosis of IBD<br>• Control: random sample of hospitalized adult patients without a primary or secondary diagnosis of IBD<br>• **IBD patients had**

				among admitted IBD patients.			**increased odds of being admitted for Hib pneumonia (AOR 1.34; CI 1.16-1.55) vs. non-IBD control.**<br>• UC patients had equal adjusted odds of being admitted for Hib compared to CD patients (AOR 1.42; CI 1.13-1.79 and AOR 1.28; CI 1.06-1.54).

Evidence Profile Table

**Certainty Assessment**								**Summary of Findings**	
Studies	Risk of bias	Inconsistency	Indirectness	Imprecision	Other considerations	Certainty of Evidence	Overall Certainty of evidence	Study Event Rates	Relative Effect (95% CI)
**Mortality – CRITICAL**							⊕⊖⊖⊖ VERY LOW		
1 Observational study[1]	Very serious[a]	Not serious	Serious[b]	Not serious	None	⊕⊖⊖⊖ VERY LOW		5 deaths during hospitalization for Hib among all patients	No difference in mortality among IBD patients vs. non-IBD control
**VPI (Admission for Hib pneumonia) - CRITICAL**									

1 observational study[1]	Very serious[a]	Not serious	Serious[b]	Not serious	None	⨁⊖⊖⊖ VERY LOW		Prevalence: 19.2/100,000 in IBD patients vs. 14.0/100,000 in non-IBD control	aOR 1.34 (1.16-1.55)

Footnotes:

a. Downgraded two levels for study limitations. Possible residual confounding factors including medication use (e.g. immunosuppressives or biologics), Hib vaccination status, as well as severity and activity of IBD may over-estimate the risk of hospitalization for Hib in IBD patients compared to controls. High risk for detection and admission bias as patients with IBD and pneumonia may be more likely to be tested and admitted for Hib than controls, thus creating an overestimate of the prevalence of Hib pneumonia among admitted IBD patients. Data were reliant on administrative discharge diagnoses. Possible misclassification errors due to errors of miscoding, and the codes have not been previously validated.

b. Downgraded for indirectness. Study included only a highly selected population (hospitalized patients with a secondary diagnosis of IBD), and did not capture Hib pneumonia patients treated as outpatients. Hence, the risk of Hib infection among all IBD patients (population of interest) vs. non-IBD patients is unknown.

References:

1. Stobaugh DJ, Deepak P, Ehrenpreis ED. Hospitalizations for vaccine preventable pneumonias in patients with inflammatory bowel disease: a 6-year analysis of the Nationwide Inpatient Sample. Clin Exp Gastroenterol. 2013 May 6;6:43-9.

Effectiveness and Safety of Hib vaccine in IBD patients

Summary - Pediatric

**PICO 8**	**In pediatric patients with IBD, should vaccination vs. no vaccination against Haemophilus Influenzae type b (Hib) be given?**
**Population**	Pediatric patients with IBD with documented or presumed lack of immunity against Hib
**Intervention**	Vaccination against Hib
**Comparator**	No vaccination against Hib
**Outcome**	Mortality, VPI (Hib infection), SAEs, Immunogenicity
**Perspective**	Population

There was no RCT or observational studies comparing Hib vaccine with placebo or no treatment in pediatric patients with IBD to address this PICO question. Literature search also did not identify any studies assessing the immunogenicity, clinical effectiveness or safety of Hib vaccine in pediatric patients with IBD.

A Cochrane systematic review has shown that Hib conjugate vaccines to be safe and effective in reducing the risk of invasive Hib disease in children under five years of age (RR 0.20; 95% CI 0.07-0.54).[1] No serious adverse events were reported in any of the trials.[1] Because of the large beneficial effects of conjugate Hib vaccine on invasive Hib disease and the lack of vaccine-related serious adverse effects, the World Health Organization (WHO) recommends Hib vaccination be included in all routine infant immunization programs all over the world. The GRADE rating started at high. The evidence was downgraded due to heterogeneity (significant variation in the estimates of effect in different trials). The evidence was not downgraded due to indirectness related to patient population (general population vs. IBD patients). Patient population included children less than 5 years old in the general population. Yet, there is no reason to suspect that pediatric IBD patients are at lower risks for developing Hib infection than non-IBD patients. On the contrary, there is reason to suspect that pediatric IBD patients may be at higher risks for developing Hib infection due to immune dysregulation and/or the use of immunosuppressive medications. There is also no evidence to suggest that the Hib vaccines are harmful or less effective in IBD patients. Therefore, the evidence was anchored with the general population since there is no reason to deviate from country-specific immunization guidelines for the general population with protocols based on local epidemiologic, programmatic, resource, policy, disease control objectives and strategies. **In summary, there is moderate certainty evidence that Hib vaccine is safe and effective in reducing the risk of invasive Hib disease in pediatric IBD patients under five years of age.**

In unimmunized children older than 5 years of age (and adults), ACIP and NACI recommends Hib vaccine only for high-risk medical conditions for invasive Hib disease including those with anatomic or functional asplenia (e.g. sickle cell disease), HIV infection, immunoglobulin deficiency, early component complement deficiency, elective splenectomy, recipients of hematopoietic stem cell transplant, and those prior to receiving chemotherapy or radiation therapy for malignant neoplasms. Literature search did not identify any study that assessed the safety and effectiveness of Hib vaccine in pediatric patients over the age of 5 (general population or patients with IBD). Therefore, **the benefits of Hib vaccine are more uncertain in pediatric IBD patients over the age of 5, although harms of Hib vaccine are likely to be very low. If the data is extrapolated from pediatric patients under 5 years of age, the certainty of evidence would need to be downgraded to low for indirectness (lower risks of Hib and infections may be more likely to be caused by non-typeable *Haemophilus influenzae* which may reduce the effectiveness of the vaccines; paucity of safety data in this population)**.

Risk of Bias Table – Pediatric

SR of RCTs							
**Study**	**Adequate sequence generation**	**Allocation concealment**	**Blinding**	**Incomplete outcome data assessed**	**Free of selective reporting**	**Free of other bias**	**Comments**
Swingler 2007 (4 RCTs)	OK Except 1 trial	OK Except 1 trial	OK Except 1 trial	Unclear<br><br>Outcomes were measured by case detection	OK	OK	• SR of 6 RCTs (4 included in meta-analysis, n = 162,140 patients) of conjugate Hib vaccine in preventing Hib disease or death in children under 5 years of age<br>• RR for invasive Hib disease RR 0.20 (0.07-0.54)<br>• RR Hib-related mortality 0.29 (0.07-1.20)

Evidence Profile Table - Pediatric

**Certainty Assessment**								**Summary of Findings**				**Comments**
								No of patients (ITT)		Effect		
Studies	Risk of bias	Inconsistency	Indirectness	Imprecision	Other considerations	Certainty of Evidence	Overall certainty of evidence	Conjugate Hib vaccine	Control	Relative (95%CI)	Absolute (95%CI)	
**Mortality (Hib-related) - CRITICAL**							⊕⊕⊕⊖<br>MODERATE<br>Age ≤ 5<br><br>⊕⊕⊖⊖<br>LOW					
1 SR[1] of 2 RCTs<br><br>**Children ≤ age 5**	Not serious	Serious[a]	Not serious[b]	Not serious[c]	Publication bias cannot be assessed (< 10 studies)	⊕⊕⊕⊖<br>MODERATE		2/24,078 (0.0%)	8/23,960 (0.0%)	RR 0.29 (0.07 to 1.20)	Not calculable	

**VPI (All invasive Hib disease) - CRITICAL**							Age > 5[d]					
1 SR[1] of 4 RCTs<br><br>**Children ≤ age 5**	Not serious	Serious[a]	Not serious[b]	Not serious[c]	Publication bias cannot be assessed (< 10 studies)	⊕⊕⊕⊖ MODERATE		29/83,132 (0.0%)	149/79,008 (0.2%)	RR 0.20 (0.07 to 0.54)	2 fewer per 1,000 (from 2 fewer to 1 fewer)	
**Serious adverse effects - CRITICAL**												
1 SR[1] of 6 RCTs<br><br>**Children ≤ age 5**	Not serious	Not serious	Not serious[b]	Not serious[c]	Publication bias cannot be assessed (< 10 studies)	⊕⊕⊕⊕ HIGH		No serious adverse events were reported in any of the trials, involving a total of 257,000 infants				

Footnotes:

a. Downgraded for inconsistency (statistical heterogeneity).
b. Not downgraded for indirectness. Patient population included children less than 5 years old in the general population. Yet, there is no reason to suspect that IBD patients have lower baseline risks of developing Hib infection and Hib-related mortality than the general population. On the contrary, there is reason to suspect that pediatric IBD patients may be at higher risks for developing Hib infection due to immune dysregulation or the use of immunosuppressive medications. There is also no evidence to suggest that the Hib vaccines are harmful or less effective in IBD patients. Therefore, the evidence was anchored at the general population since there is no reason to deviate from country-specific immunization guidelines for the general population with protocols based on local epidemiologic, programmatic, resource, policy, disease control objectives and strategies.
c. Low event rates, but very large sample size. Therefore, evidence was not downgraded for imprecision.
d. Downgraded for indirectness if data from children ≤ age 5 is extrapolated to children age > 5. In children age > 5, risk of Hib is lower and infections may be more likely to be caused by non-typeable Haemophilus influenzae which may reduce the effectiveness of the vaccines. Paucity of safety data in this population.

References:

1. Swingler GH, Michaels D, Hussey GG. Conjugate vaccines for preventing Haemophilus influenzae type B infections. Cochrane Database Syst Rev. 2009 Oct 7;(4):CD001729.

Evidence to Decision Table – Pediatric (5 years of age and under)

**PICO 8A**	**In pediatric patients with IBD (5 years of age and younger), should vaccination vs. no vaccination against Haemophilus Influenzae type b (Hib) be given?**
**Population**	Pediatric patients with IBD (5 years of age and younger) with documented or presumed lack of

	immunity against Hib
**Intervention**	Vaccination against Hib
**Comparator**	No vaccination against Hib
**Outcome**	Mortality, VPI (Hib infection), SAEs, Immunogenicity
**Perspective**	Population

	**Judgement**	**Research evidence**	**Additional considerations**
**Desirable Effects**	**How substantial are the desirable anticipated effects?**<br><br>o Trivial<br>o Small<br>o Moderate<br>o Large<br><br>o Varies<br>o Don't know	See Evidence Profile Table<br><br>**Risk of Hib Infection in Pediatric IBD Patients**<br><br>Literature search did not identify any study on the risk of Hib infection in pediatric IBD patients.<br><br>**Effectiveness and Safety of Hib Vaccine in Pediatric IBD Patients**<br><br>There was no RCT or observational studies comparing Hib vaccine with placebo or no treatment in pediatric patients with IBD to address this PICO question. Literature search also did not identify any studies assessing the immunogenicity, clinical effectiveness or safety of Hib vaccine in pediatric patients with IBD.<br><br>A Cochrane systematic review has shown that Hib conjugate vaccines to be safe and effective in reducing the risk of invasive Hib disease in children under five years of age (RR 0.20; 95% CI 0.07-0.54).[1] No serious adverse events were reported in any of the trials.[1] Because of the large beneficial effects of conjugate Hib vaccine on invasive Hib disease and the lack of vaccine-related serious adverse effects, the World Health Organization (WHO) recommends Hib vaccination be included in all routine infant immunization programs all over the world. The GRADE rating started at high. The evidence was downgraded due to heterogeneity (significant variation in the estimates of effect in different trials). The evidence was not downgraded due to indirectness related to patient population (general population vs. IBD patients). Patient population included children less than 5 years old in the general population. Yet, there is no reason to suspect that pediatric IBD patients are at lower risks for developing Hib infection than non-IBD patients. On the contrary, there is reason to suspect that pediatric IBD patients may be at higher risks for developing Hib infection due to immune dysregulation and/or the use of immunosuppressive medications. There is also no evidence to suggest that the Hib vaccines are harmful or less effective in IBD patients. Therefore, the evidence was anchored at the general population since there is no reason to deviate from country-specific immunization guidelines for the general	
**Undesirable Effects**	**How substantial are the undesirable anticipated effects?**<br><br>o Large<br>o Moderate<br>o Small<br>o Trivial<br><br>o Varies<br>o Don't know		

		population with protocols based on local epidemiologic, programmatic, resource, policy, disease control objectives and strategies. **In summary, there is <u>moderate</u> certainty evidence that Hib vaccine is safe and effective in reducing the risk of invasive Hib disease in pediatric IBD patients under five years of age.**	
Certainty of evidence	**What is the overall certainty of the evidence of effects?**<br><br>o Very low<br>o Low<br>o Moderate (age ≤ 5)<br>o High<br><br>o No included studies		
Values and Preferences	**Is there important uncertainty about or variability in how much people value the main outcomes?**<br><br>o Important uncertainty or variability<br>o Possibly important uncertainty or variability<br>o Probably no important uncertainty or variability<br>o No important uncertainty or variability	Patients likely value patient-important outcomes (mortality, VPI, adverse effects) more than surrogate outcomes (immunogenicity).	
Balance of effects	**Does the balance between desirable and undesirable effects favor the intervention or the comparison?**<br><br>o Favors the comparison<br>o Probably favors the comparison<br>o Does not favor either the intervention or the comparison<br>o Probably favors the intervention<br>o Favors the intervention<br><br>o Varies<br>o Don't know		

<table>
<tr>
<td>Resources required</td>
<td>How large are the resource requirements (costs)?

○ Large costs
○ Moderate costs
○ Negligible costs and savings
○ Moderate savings
○ Large savings

○ Varies
○ Don't know</td>
<td>CDC vaccine price list last reviewed/updated: July 1, 2019

<table><tr><th>Brandname</th><th>CDC cost/dose</th><th>Private sector cost/dose</th></tr><tr><td>PedvaxHIB®</td><td>$13.21</td><td>$26.23</td></tr><tr><td>ActHIB®</td><td>$9.484</td><td>$16.51</td></tr><tr><td>Hiberix®</td><td>$9.46</td><td>$10.85</td></tr></table></td>
<td></td>
</tr>
<tr>
<td>Certainty of Evidence of Required Resources</td>
<td>What is the certainty of the evidence of resource requirements (costs)?

○ Very low
○ Low
○ Moderate
○ High

○ No included studies</td>
<td>The costs of delivering routine immunization services may vary widely across countries and different health system settings. See Immunization Costing Action Network (ICAN) Immunization Delivery Cost Catalogue. http://immunizationeconomics.org/ican-idcc.

According to a systematic review of economic evaluations of Hib vaccine with inclusion of 26 studies, the costs of vaccine ranged from USD 0.3 to 22.5.[2] The required costs of vaccine delivery ranged from USD 0.26 to 20.[2]</td>
<td></td>
</tr>
<tr>
<td>Cost effectiveness</td>
<td>Does the cost-effectiveness of the intervention favor the intervention or the comparison?

○ Favors the comparison
○ Probably favors the comparison
○ Does not favor either the intervention or the comparison
○ Probably favors the intervention
○ Favors the intervention

○ Varies
○ No included studies</td>
<td>A World Health Organization (WHO) commissioned systematic review of economic evaluations of Hib vaccine found Hib vaccination programs in children to be cost-effective across geographic regions and country income levels, and Hib vaccination is recommended for inclusion into all national immunization programs.[2] The incidence rate of Hib disease was the most influential determinant of cost-effectiveness.[2]</td>
<td></td>
</tr>
</table>

**Acceptability**	**Is the intervention acceptable to key stakeholders?**<br><br>o No<br>o Probably no<br>o Probably yes<br>o Yes<br><br>o Varies<br>o Don't know		
**Feasibility**	**Is the intervention feasible to implement?**<br><br>o No<br>o Probably no<br>o Probably yes<br>o Yes<br><br>o Varies<br>o Don't know		

References:

1. Swingler GH, Michaels D, Hussey GG. Conjugate vaccines for preventing Haemophilus influenzae type B infections. Cochrane Database Syst Rev. 2009 Oct 7;(4):CD001729.
2. Chongmelaxme B, Hammanee M, Phooaphirak W, Kotirum S, Hutubessy R, Chaiyakunapruk N. Economic evaluations of Haemophilus influenzae type b (Hib) vaccine: a systematic review. J Med Econ. 2017 Oct;20(10):1094-1106.

Conclusion – Pediatric (5 years of age and under)

PICO 8A: In pediatric patients with IBD (5 years of age and younger), should vaccination vs. no vaccination against Haemophilus Influenzae type b (Hib) be given?

Moderate certainty of evidence
Direction – Yes (100%)

Strength of recommendation – Strong (100%)

Type of recommendation	Strong recommendation against the intervention	Conditional recommendation against the intervention	Conditional recommendation for either the intervention or the comparison	Conditional recommendation for the intervention	Strong recommendation for the intervention
	○	○	○	○	○

**Recommendation**	**Statement 8A: In pediatric patients with IBD (5 years of age and younger), we recommend *Haemophilus influenza type b (Hib)* vaccine be given.**
**Justification**	
**Subgroup considerations**	
**Implementation considerations**	
**Monitoring and evaluation**	• Ongoing monitoring of safety of Hib vaccine in pediatric IBD patients
**Research priorities**	• Before-and-after study to determine Hib vaccine immunogenicity and safety among pediatric IBD patients (stratified by the use of immunosuppressive medications) by evaluating postimmunization anti-PRP level.

Evidence to Decision Table – Pediatric (older than 5 years of age)

PICO 8B	In pediatric patients with IBD (older than 5 years of age), should vaccination vs. no vaccination against Haemophilus Influenzae type b (Hib) be given?
Population	Pediatric patients with IBD (older than 5 years of age) with documented or presumed lack of immunity against Hib
Intervention	Vaccination against Hib
Comparator	No vaccination against Hib
Outcome	Mortality, VPI (Hib infection), SAEs, Immunogenicity
Perspective	Population

	Judgement	Research evidence	Additional considerations
Desirable Effects	**How substantial are the desirable anticipated effects?**<br><br>○ Trivial<br>○ Small<br>○ Moderate<br>○ Large<br><br>○ Varies<br>○ Don't know	See Evidence Profile Table<br><br>**Risk of Hib Infection in Pediatric IBD Patients**<br><br>Literature search did not identify any study on the risk of Hib infection in pediatric IBD patients.<br><br>**Effectiveness and Safety of Hib Vaccine in Pediatric IBD Patients**<br><br>There was no RCT or observational studies comparing Hib vaccine with placebo or no treatment in pediatric patients with IBD to address this PICO question. Literature search also did not identify any studies assessing the immunogenicity, clinical effectiveness or safety of Hib vaccine in pediatric patients with IBD.<br><br>A Cochrane systematic review has shown that Hib conjugate vaccines to be safe and effective in reducing the risk of invasive Hib disease in children under five years of age (RR 0.20; 95% CI 0.07-0.54).[1] No serious adverse events were reported in any of the trials.[1] Because of the large beneficial effects of conjugate Hib vaccine on invasive Hib disease and the lack of vaccine-related serious adverse effects, the World Health Organization (WHO) recommends Hib vaccination be included in all routine infant immunization programs all over the world. The GRADE rating started at high. The evidence was downgraded due to heterogeneity (significant variation in the estimates of effect in different trials). The evidence was not downgraded due to indirectness	
Undesirable Effects	**How substantial are the undesirable anticipated effects?**<br><br>○ Large<br>○ Moderate<br>○ Small<br>○ Trivial<br><br>○ Varies		

	○ Don't know	related to patient population (general population vs. IBD patients). Patient population included children less than 5 years old in the general population. Yet, there is no reason to suspect that pediatric IBD patients are at lower risks for developing Hib infection than non-IBD patients. On the contrary, there is reason to suspect that pediatric IBD patients may be at higher risks for developing Hib infection due to immune dysregulation and/or the use of immunosuppressive medications. There is also no evidence to suggest that the Hib vaccines are harmful or less effective in IBD patients. Therefore, the evidence was anchored at the general population since there is no reason to deviate from country-specific immunization guidelines for the general population with protocols based on local epidemiologic, programmatic, resource, policy, disease control objectives and strategies. **In summary, there is moderate certainty evidence that Hib vaccine is safe and effective in reducing the risk of invasive Hib disease in pediatric IBD patients under five years of age.**	
**Certainty of evidence**	**What is the overall certainty of the evidence of effects?**<br><br>○ Very low<br>○ Low (age > 5)<br>○ Moderate<br>○ High<br><br>○ No included studies		
**Values and Preferences**	**Is there important uncertainty about or variability in how much people value the main outcomes?**<br><br>○ Important uncertainty or variability<br>○ Possibly important uncertainty or variability<br>○ Probably no important uncertainty or variability<br>○ No important uncertainty or variability	Patients likely value patient-important outcomes (mortality, VPI, adverse effects) more than surrogate outcomes (immunogenicity).	
**Balance of effects**	**Does the balance between desirable and undesirable effects favor the intervention or the comparison?**<br><br>○ Favors the comparison<br>○ Probably favors the comparison<br>○ Does not favor either the intervention or the comparison<br>○ Probably favors the intervention<br>○ Favors the intervention<br><br>○ Varies		

<table>
<tr><td></td><td>○ Don't know</td><td></td><td></td></tr>
<tr><td>Resources required</td><td>How large are the resource requirements (costs)?

○ Large costs
○ Moderate costs
○ Negligible costs and savings
○ Moderate savings
○ Large savings

○ Varies
○ Don't know</td><td>CDC vaccine price list last reviewed/updated: July 1, 2019

<table><tr><th>Brandname</th><th>CDC cost/dose</th><th>Private sector cost/dose</th></tr><tr><td>PedvaxHIB®</td><td>$13.21</td><td>$26.23</td></tr><tr><td>ActHIB®</td><td>$9.484</td><td>$16.51</td></tr><tr><td>Hiberix®</td><td>$9.46</td><td>$10.85</td></tr></table></td><td></td></tr>
<tr><td>Certainty of Evidence of Required Resources</td><td>What is the certainty of the evidence of resource requirements (costs)?

○ Very low
○ Low
○ Moderate
○ High

○ No included studies</td><td>The costs of delivering routine immunization services may vary widely across countries and different health system settings. See Immunization Costing Action Network (ICAN) Immunization Delivery Cost Catalogue. http://immunizationeconomics.org/ican-idcc.

According to a systematic review of economic evaluations of Hib vaccine with inclusion of 26 studies, the costs of vaccine ranged from USD 0.3 to 22.5.[2] The required costs of vaccine delivery ranged from USD 0.26 to 20.[2]</td><td></td></tr>
</table>

**Cost effectiveness**	**Does the cost-effectiveness of the intervention favor the intervention or the comparison?**<br><br>o Favors the comparison<br>o Probably favors the comparison<br>o Does not favor either the intervention or the comparison<br>o Probably favors the intervention<br>o Favors the intervention<br><br>o Varies<br>o No included studies	A World Health Organization (WHO) commissioned systematic review of economic evaluations of Hib vaccine found Hib vaccination programs in children (under age 5) to be cost-effective across geographic regions and country income levels, and Hib vaccination is recommended for inclusion into all national immunization programs.[2] The incidence rate of Hib disease was the most influential determinant of cost-effectiveness.[2]	
**Acceptability**	**Is the intervention acceptable to key stakeholders?**<br><br>o No<br>o Probably no<br>o Probably yes<br>o Yes<br><br>o Varies<br>o Don't know	Depends on the cost to the patients and parents.	
**Feasibility**	**Is the intervention feasible to implement?**<br><br>o No<br>o Probably no<br>o Probably yes<br>o Yes<br><br>o Varies<br>o Don't know	Access can be an issue in some jurisdictions; in Canada, provincial access varies in children over age 5 years, making it difficult to give the vaccine to patients at high risk. Patient acceptability can vary, since in the absence of reimbursement cost can be an issue.	

Conclusion – Pediatric (older than 5 years of age)

PICO 8B: In pediatric patients with IBD (older than 5 years of age), should vaccination vs. no vaccination against Haemophilus Influenzae type b (Hib) be given?

Low certainty of evidence
Direction – Yes (100%)
Strength of recommendation – conditional (default)

**Type of recommendation**	**Strong recommendation against the intervention**	**Conditional recommendation against the intervention**	**Conditional recommendation for either the intervention or the comparison**	**Conditional recommendation for the intervention**	**Strong recommendation for the intervention**
	○	○	○	○	○
**Recommendation**	**Statement 8B: In unimmunized pediatric patients with IBD (older than 5 years of age), we suggest *Haemophilus influenza type b (Hib)* vaccine be given.**				
**Justification**					
**Subgroup considerations**					
**Implementation considerations**					
**Monitoring and evaluation**	• Ongoing monitoring of safety of Hib vaccine in pediatric IBD patients				
**Research priorities**	• Before-and-after study to determine Hib vaccine immunogenicity and safety among pediatric IBD patients (stratified by use of immunosuppressive medications) by evaluating postimmunization anti-PRP level				

Summary - Adults

PICO 9	**In unimmunized adult patients with IBD, should vaccination vs. no vaccination against Haemophilus Influenzae type b (Hib) be given?**
**Population**	Adult patients with IBD with documented or presumed lack of immunity against Hib
**Intervention**	Vaccination against Hib
**Comparator**	No vaccination against Hib
**Outcome**	Mortality, VPI (Hib infection), SAEs, Immunogenicity

There was no RCT or observational studies comparing Hib vaccine with placebo or no treatment in adult patients with IBD to address this PICO question.

One small prospective observational study indirectly addressed this PICO question.[1] This was a before-after (pre-post) study which included adult IBD patients who were starting on thiopurine.[1] Hib vaccine was administered to these patients at week 24. The study reported a significant increase in antibody titer 3 weeks post-vaccination.[1] However, it is uncertain whether a seroprotective response (as defined by anti-PRP levels > 1.0 ug/mL) was achieved as the results were reported as a "significant increase" in a different unit of measurement (IU/mL). The GRADE rating started at low. The rating was further downgraded to **very low** due to study limitations (incomplete outcome data, selective outcome reporting), indirectness (surrogate outcome of immunogenicity, patient population), and imprecision. **In summary, there is very low certainty evidence that thiopurine does not affect humoral responses to Hib vaccine in IBD patients. However, the evidence does not directly address the question whether Hib vaccine is effective in adult IBD patients.**

No vaccine-induced exacerbation of disease was reported in this study (**very low** certainty evidence).[1]

Overall, the certainty of evidence is anchored to safety. There is very low certainty evidence that Hib vaccine is safe and effective in adult IBD patients (lower risks of Hib compared to pediatric patients; infections may be more likely to be caused by non-typeable *Haemophilus influenzae* which may reduce the effectiveness of the vaccines; paucity of safety data in this population).

Risk of Bias Table - Adults

Before-After (Pre-Post) Studies									
			If no concurrent comparator group was used						
Study	Was there a concurrent comparator group that did not receive the intervention	If a concurrent comparator group was used, was it similar to the intervention group (or adequately adjusted) for prognostic factors	If each participant served as his/her own control (assessed before vs. after the intervention), are there compelling arguments that the outcome was not influenced by historic events / underlying secular trends	If two different consecutive cohorts of participants were assessed (before vs. after implementation of the intervention), are there (a) compelling arguments that the outcome was not influenced by historic events / underlying secular trends and (b) evidence that the two groups were similar (or adequately adjusted) for prognostic factors	Outcome detection methods valid and similar among compared groups / periods	Incomplete outcome data assessed	Selective outcome reporting	Other bias	Comments
Dotan 2012 (US and Israel)	No – but this does not affect the risk of bias as the	No – but this does not affect the risk of bias as the	OK	OK	OK	19% (10/53) either withdrew due to	Defined response to Hib vaccine as > 2-fold	OK	• Prospective cohort study of 53 IBD patients (35 CD, 15 UC, 3 IC) who were

	only explanation for increase in Hib titer is the vaccine (no other confounding factors)	only explanation for increase in Hib titer is the vaccine (no other confounding factors)				thiopurine side effects or were lost to follow-up. Reported outcomes only on 36% (19/53) who were started on thiopurine. Unclear if the other patients received Hib vaccine or not	increase in antibody titre (with at least a GMT > 1ug/mL, considered a protective level), but reported outcome as "significant increase" in titre and the unit was changed to IU/mL instead of ug/mL		starting on thiopurine treatment<br>• Patients were administered Hib vaccine at week 24<br>• Post-therapy average 6-MP dose: 1.05 +/- 0.30mg/kg<br>• Response to Hib was defined as > 2-fold increase in antibody titre (with at least a GMT > 1 ug/mL, considered a protective level)<br>• 19 patients were given Hib vaccine following 24 weeks of thiopurine (IM-treated): significant increase in Hib antibody titer: 4.72 +/- 3.29 pre vs. 6.97 +/- 3.19 IU/mL post at 27 weeks (P = 0.009)<br>• A subgroup of 9 patients had previously Hib vaccine (IM-naive): significant increase in Hib antibody titer: 3.63 +/- 3.4 pre vs. 7.62 +/- 3.7 IU/mL post at 3 weeks (P = 0.008)<br>• No vaccine induced disease exacerbation

IC – indeterminate colitis

Evidence Profile Table - Adults

Certainty Assessment								Summary of Findings	
Studies	Risk of bias	Inconsistency	Indirectness	Imprecision	Other considerations	Certainty of Evidence	Overall Certainty of evidence	Study Event Rates	Relative Effect (95% CI)
**Immunogenicity (Hib antibody titre) - IMPORTANT**							⊕⊖⊖⊖ VERY LOW		
1 Observational Study[1] **IBD patients**	Serious[a]	Not serious	Serious[b]	Serious[c]	None	⊕⊖⊖⊖ VERY LOW		• 19 patients were given Hib vaccine following 24 weeks of thiopurine (IM-treated): significant increase in Hib antibody titer: 4.72 +/- 3.29 pre- vs. 6.97 +/- 3.19 IU/mL post- at 27 weeks (P = 0.009)<br>• A subgroup of 9 patients had previous Hib vaccine (IM-naive): significant increase in Hib antibody titer: 3.63 +/- 3.4 pre- vs. 7.62 +/- 3.7 IU/mL post- at 3 weeks (P = 0.008)	
**Adverse events (Disease exacerbation) - CRITICAL**									
1 Observational Study[1] **IBD patients**	Serious[a]	Not serious	Serious[d]	Serious[c]	None	⊕⊖⊖⊖ VERY LOW		• No vaccine induced disease exacerbation	

Footnotes:

a. Downgraded for study limitations. Incomplete outcome data. Reported Hib titer only on 36% (19/53) patients who were started on thiopurine. Unclear if other patients received Hib vaccine or not. 19% (10/53) either withdrew or were lost to follow-up. Selective outcome reporting. Defined response to Hib vaccine as > 2-fold increase in antibody titre (with at least a GMT > 1ug/mL, considered a protective level), but reported outcome as "significant increase" in titre and the unit was changed to IU/mL instead of ug/mL.
b. Downgraded for indirectness. Immunogenicity is a surrogate outcome for vaccine efficacy. It is uncertain whether the standard antibody correlate in healthy population is the same as in IBD population. Included patients who were starting on thiopurine. Patient population may not be representative of IBD patients who are on other medications or no medications.
c. Downgraded for imprecision. Small sample size (28 patients with reported outcomes).
d. Downgraded for indirectness. Included patients who were starting on thiopurine. Patient population may not be representative of IBD patients who are on other medications or no medications.

References:

1. Dotan I, Werner L, Vigodman S, Agarwal S, Pfeffer J, Horowitz N, Malter L, Abreu M, Ullman T, Guzner-Gur H, Halpern Z, Mayer L. Normal response to vaccines in inflammatory bowel disease patients treated with thiopurines. Inflamm Bowel Dis. 2012 Feb;18(2):261-8.

Evidence to Decision Table - Adults

PICO 9	**In unimmunized adult patients with IBD, should vaccination vs. no vaccination against Haemophilus Influenzae type b (Hib) be given?**
**Population**	Adult patients with IBD with documented or presumed lack of immunity against Hib
**Intervention**	Vaccination against Hib
**Comparator**	No vaccination against Hib
**Outcome**	Mortality, VPI (Hib infection), SAEs, Immunogenicity
**Perspective**	Population

	Judgement	Research evidence	Additional considerations
**Desirable Effects**	**How substantial are the desirable anticipated effects?**<br><br>○ Trivial<br>○ Small<br>○ Moderate<br>○ Large<br><br>○ Varies<br>○ Don't know	See Evidence Profile Tables.<br><br>**Risk of Hib infection in adult IBD patients**<br><br>Only one observational study directly addressed this PICO question.[1] This was a cross-sectional case-control study that used an administrative database (Nationwide Inpatient Sample) to compare the risks of hospitalization for Hib pneumonia among adult IBD patients vs. non-IBD controls. It is important to note that Hib pneumonia patients treated as outpatients were excluded. After adjusting for various factors including comorbidities, risk factors for pneumonia, as well as patient and hospital characteristics, IBD patients had increased odds of being admitted for Hib pneumonia **(aOR 1.34; CI 1.16-1.55)** when compared to the non-IBD control group. The GRADE rating started at high as it was considered a prognostic study (providing evidence about the likelihood of Hib pneumonia in patients with IBD). The rating was further downgraded to very low due to study limitations (residual confounding factors, detection bias, admission bias, and misclassification bias) and indirectness (admitted IBD patients with a primary diagnosis of Hib pneumonia, and not all IBD patients). In particular, patients with IBD and pneumonia may be more likely to be tested and admitted for Hib than non-IBD controls, thus creating an overestimate of the prevalence of Hib pneumonia among admitted IBD patients. **In summary, there is very low certainty evidence that adult IBD patients have an**	Most adult patients born after 1985 in the US and after 1992 in Canada would have received Hib vaccine when it was first introduced.<br><br>Hib disease is uncommon in adults and in children aged > 5 years. The majority of *Haemophilus influenzae* infections in adults are caused by non-typeable Haemophilus influenzae with an overall case fatality ratio (CER) of 19.5%.<br><br>In unimmunized adults and children older than 5 years of age, Hib vaccine is recommended only for high-

<table>
<tr>
<td></td>
<td></td>
<td rowspan="2">increased risk of Hib infection compared to non-IBD patients.

Effectiveness and safety of Hib in adult IBD patients

Only one small prospective observational study directly addressed this PICO question.[2] This was a before-after (pre-post) study which included adult IBD patients who were starting on thiopurine. Hib vaccine was administered to these patients at week 24. The study reported a significant increase in antibody titer 3 weeks post-vaccination. However, it is uncertain whether a seroprotective response (as defined by anti-PRP levels > 1.0 ug/mL) was achieved as the results were reported as a “significant increase” in a different unit of measurement (IU/mL). The GRADE rating started at low. The rating was further downgraded to very low due to study limitations (incomplete outcome data, selective outcome reporting), indirectness (surrogate outcome of immunogenicity, patient population), and imprecision. In summary, there is <u>very low</u> certainty evidence that thiopurine does not affect humoral responses to Hib vaccine in adult IBD patients. However, the evidence does not address the question whether Hib vaccine is effective in adult IBD patients.

No vaccine-induced exacerbation of disease was reported in this study (very low certainty evidence).[2]

Overall, the certainty of evidence is anchored to safety. There is <u>very low</u> certainty evidence that Hib vaccine is safe and effective in adult IBD patients,</td>
<td>risk medical conditions for invasive Hib disease including those with anatomic or functional asplenia (e.g. sickle cell disease), HIV infection, immunoglobulin deficiency, early component complement deficiency, elective splenectomy, recipients of hematopoietic stem cell transplant, and those prior to receiving chemotherapy or radiation therapy for malignant neoplasms.

Hib vaccination is not recommended routinely for unvaccinated adults and children aged more than 5 years because, in the pre-vaccination era, invasive Hib disease affected almost exclusively children aged less than 5 years. Healthy, unvaccinated adults have protective immunity against Hib due to natural anti-Hib antibodies that may have been induced by exposure to some common environmental bacteria that carry antigens cross-reacting with PRP.</td>
</tr>
<tr>
<td>Undesirable Effects</td>
<td>How substantial are the undesirable anticipated effects?

○ Large
○ Moderate
○ Small
○ Trivial

○ Varies</td>
<td></td>
</tr>
</table>

	○ Don't know		
**Certainty of evidence**	**What is the overall certainty of the evidence of effects?**<br><br>○ Very low<br>○ Low<br>○ Moderate<br>○ High<br><br>○ No included studies		
**Values and Preferences**	**Is there important uncertainty about or variability in how much people value the main outcomes?**<br><br>○ Important uncertainty or variability<br>○ Possibly important uncertainty or variability<br>○ Probably no important uncertainty or variability<br>○ No important uncertainty or variability	Patients likely value patient-important outcomes (mortality, VPI, adverse effects) more than surrogate outcomes (immunogenicity).	
**Balance of effects**	**Does the balance between desirable and undesirable effects favor the intervention or the comparison?**<br><br>○ Favors the comparison<br>○ Probably favors the comparison<br>○ Does not favor either the intervention or the comparison<br>○ Probably favors the intervention<br>○ Favors the intervention<br><br>○ Varies<br>○ Don't know		

**Resources required**	**How large are the resource requirements (costs)?**<br><br>○ Large costs<br>○ Moderate costs<br>○ Negligible costs and savings<br>○ Moderate savings<br>○ Large savings<br><br>○ Varies<br>○ Don't know	CDC vaccine price list last reviewed/updated: July 1, 2019<br><br><table><tr><th>Brandname</th><th>CDC cost/dose</th><th>Private sector cost/dose</th></tr><tr><td>PedvaxHIB®</td><td>$13.21</td><td>$26.23</td></tr><tr><td>ActHIB®</td><td>$9.484</td><td>$16.51</td></tr><tr><td>Hiberix®</td><td>$9.46</td><td>$10.85</td></tr></table>	Vaccination rates for Hib in Canada and US are quite high (60-98% and 81.8% respectively). Most adult IBD patients in North America would have received Hib vaccination as children. However, immigrants may not have received Hib vaccination as children.
**Certainty of Evidence of Required Resources**	**What is the certainty of the evidence of resource requirements (costs)?**<br><br>○ Very low<br>○ Low<br>○ Moderate<br>○ High<br><br>○ No included studies	The costs of delivering routine immunization services may vary widely across countries and different health system settings. See Immunization Costing Action Network (ICAN) Immunization Delivery Cost Catalogue. http://immunizationeconomics.org/ican-idcc.<br><br>According to a systematic review of economic evaluations of Hib vaccine with inclusion of 26 studies, the costs of vaccine ranged from USD 0.3 to 22.5.[3] The required costs of vaccine delivery ranged from USD 0.26 to 20.[3]	
**Cost effectiveness**	**Does the cost-effectiveness of the intervention favor the intervention or the comparison?**<br><br>○ Favors the comparison<br>○ Probably favors the comparison<br>○ Does not favor either the intervention or the comparison<br>○ Probably favors the intervention<br>○ Favors the intervention<br><br>○ Varies<br>○ No included studies	No published study of cost-effectiveness of Hib vaccine in adult IBD patients.	

**Acceptability**	**Is the intervention acceptable to key stakeholders?**<br><br>o No<br>o Probably no<br>o Probably yes<br>o Yes<br><br>o Varies<br>o Don't know	Cost may be a factor as this is currently not covered for individuals > age 5 without risk factors for Hib infection.	
**Feasibility**	**Is the intervention feasible to implement?**<br><br>o No<br>o Probably no<br>o Probably yes<br>o Yes<br><br>o Varies<br>o Don't know		

References:

1. Stobaugh DJ, Deepak P, Ehrenpreis ED. Hospitalizations for vaccine preventable pneumonias in patients with inflammatory bowel disease: a 6-year analysis of the Nationwide Inpatient Sample. Clin Exp Gastroenterol. 2013 May 6;6:43-9.
2. Dotan I, Werner L, Vigodman S, Agarwal S, Pfeffer J, Horowitz N, Malter L, Abreu M, Ullman T, Guzner-Gur H, Halpern Z, Mayer L. Normal response to vaccines in inflammatory bowel disease patients treated with thiopurines. Inflamm Bowel Dis. 2012 Feb;18(2):261-8.
3. Chongmelaxme B, Hammanee M, Phooaphirak W, Kotirum S, Hutubessy R, Chaiyakunapruk N. Economic evaluations of Haemophilus influenzae type b (Hib) vaccine: a systematic review. J Med Econ. 2017 Oct;20(10):1094-1106.

Conclusion – Adults

PICO 9: In unimmunized adult patients with IBD, should vaccination vs. no vaccination against Haemophilus Influenzae type b (Hib) be given?

Very low certainty of evidence

Direction – Yes (78%), Uncertain (22%)
Strength – Conditional

Type of recommendation	Strong recommendation against the intervention	Conditional recommendation against the intervention	Conditional recommendation for either the intervention or the comparison	Conditional recommendation for the intervention	Strong recommendation for the intervention
	○	○	○	○	○

**Recommendation**	**Statement 9: In unimmunized adult patients with IBD, we suggest Haemophilus influenzae type b (Hib) vaccine be given.**
**Justification**	
**Subgroup considerations**	
**Implementation considerations**	
**Monitoring and evaluation**	• Ongoing monitoring of safety of Hib vaccine in adult IBD patients
**Research priorities**	• Population-based study to determine the risks and predictors of invasive Hib infection in adult IBD patients<br>• Before-and-after study to determine Hib vaccine immunogenicity among adult IBD patients on immunosuppressives by evaluating postimmunization anti-PRP level (with seroprotective response as defined by $\geq$1.0 ug/mL)

Herpes Zoster

Background

Herpes zoster is a manifestation of reactivation of the varicella-zoster virus (VZV) within the dorsal ganglion. Only people who had natural infection with wild-type VZV or had varicella vaccination can develop herpes zoster. The infection is characterized by painful, unilateral vesicular rash, usually in a single dermatomal distribution. The most common complication of herpes zoster is post-hepatic neuralgia (PNH) in 10-50% of cases with potential long-term sequelae. VZV reactivation can also lead to a variety of neurologic and ocular disorders, including herpes zoster ophthalmicus, herpes zoster oticus, necrotizing retinitis, cranial and peripheral nerve palsies, myelopathy, meningoencephalitis, cerebellitis, and visceral involvement including pneumonitis, hepatitis, and acute retinal necrosis. The risk of mortality from VZV-associated disease is low.

More than 90% of the population has serologic evidence of VZV infection, and the lifetime risk of herpes zoster infection has been estimated to be as high as 30% (1 in 3 persons) in the general population in the US. In the US, the incidence rate (IR) of herpes zoster in the unvaccinated general population 50 years or older is estimated to be 7.0 cases per 1,000 person-years. Herpes zoster occurs most frequently among older adults and immunocompromised individuals. Age is the most important risk factor for development of herpes zoster with over two-thirds of cases occurring in individuals over 50 years of age. The severity of illness associated with herpes zoster and the risk of complications, including PHN and hospitalization, also increases with age. Children who get the varicella vaccine appear to have a lower risk of herpes zoster compared with people who were infected with wide-type VZV. The rate of herpes zoster in US children has been declining since the routine varicella vaccination program started.

Since 2008, a one-dose herpes zoster live-attenuated vaccine (LZV, Zostavax®) has been recommended by the ACIP for the prevention of herpes zoster in immunocompetent adults aged 60 years and older. Since 2017, a two-dose recombinant zoster vaccine (RZV, Shingrix®) has been recommended by ACIP as the preferred vaccine for prevention of herpes zoster and related complications in immunocompetent adults aged 50 years and older because of its higher efficacy across all age groups compared to the LZV.[1] The RZV is recommended for all individuals for whom the vaccine is indicated regardless of whether the person has a history of varicella infection.[1] NACI also suggests RZV be considered for immunocompromised adults (either due to underlying

conditions or immunosuppressive agents) aged 50 years and older based on a case-by-case assessment of the benefits vs. risks.[2] When indicated, the vaccine is recommended to be administered at least 14 days before initiating immunosuppressive treatment.

The American College of Gastroenterology (ACG) in 2017 recommends that all IBD patients older than 50 should be vaccinated, whereas European Crohn's and Colitis Organization in 2014 recommends that all IBD patients older than 60 years should be vaccinated.[3,4] The American College of Rheumatology in 2016 made a conditional recommendation to use herpes zoster immunization with live-attenuated vaccine (LZV, Zostavax®) at age 50 and older prior to starting biologics in patients with rheumatoid arthritis, considering the higher infection risk due to the condition and its treatments.

There are currently no established humoral and/or cellular correlates of protection following immunization against herpes zoster (immunogenicity).

Due to the low incidence of HZ infection in pediatric population, the PICO question of "in pediatric patients with IBD who are immune to VZV, should vaccination vs. no vaccination against HZ (recombinant zoster vaccine) be given" was deemed to be not clinically relevant or important for inclusion in this guideline by the guideline steering committee. As well, live-attenuated vaccine (LZV, Zostavax®) was not included for this guideline as current guidelines for general population recommend recombinant zoster vaccine as the preferred vaccine.

References:

1. Dooling KL, Guo A, Patel M, et al. Recommendations of the Advisory Committee on Immunization Practices for Use of Herpes Zoster Vaccines. MMWR Morb Mortal Wkly Rep 2018;67:103–108. DOI: http://dx.doi.org/10.15585/mmwr.mm6703a5
2. Public Health Agency of Canada. https://www.canada.ca/en/public-health/services/publications/healthy-living/canadian-immunization-guide-part-4-active-vaccines/page-8-herpes-zoster-(shingles)-vaccine.html
3. Farraye FA, Melmed GY, Lichtenstein GR, Kane SV. ACG Clinical Guideline: Preventive Care in Inflammatory Bowel Disease. Am J Gastroenterol. 2017 Feb;112(2):241-258.
4. Rahier JF, Magro F, Abreu C, Armuzzi A, Ben-Horin S, Chowers Y, Cottone M, de Ridder L, Doherty G, Ehehalt R, Esteve M, Katsanos K, Lees CW, Macmahon E, Moreels T, Reinisch W, Tilg H, Tremblay L, Veereman-Wauters G, Viget N, Yazdanpanah Y, Eliakim R, Colombel JF; European Crohn's and Colitis Organisation (ECCO). Second European evidence-based consensus on the prevention, diagnosis and management of opportunistic infections in inflammatory bowel disease. J Crohns Colitis. 2014 Jun;8(6):443-68.
5. Singh JA, Saag KG, Bridges SL Jr, Akl EA, Bannuru RR, Sullivan MC, Vaysbrot E, McNaughton C, Osani M, Shmerling RH, Curtis JR, Furst DE, Parks D, Kavanaugh A, O'Dell J, King C, Leong A, Matteson EL, Schousboe JT, Drevlow B, Ginsberg S, Grober J, St Clair EW, Tindall E, Miller AS, McAlindon T. 2015 American College of Rheumatology Guideline for the Treatment of Rheumatoid Arthritis. Arthritis Care Res (Hoboken). 2016 Jan;68(1):1-25.

Risk of zoster infection in IBD patients

PICO: What is the risk of zoster infection in people with IBD compared to people without IBD?

Summary - Adults

IBD patients vs. non-IBD controls

Nine case-control studies (considered as prognostic studies) addressed this PICO question.[1-9] All nine studies found an increased risk of herpes zoster (HZ) in IBD patients compared to the general population (1.2-1.8 times). The GRADE rating started at high as these studies provided prognostic evidence about the likelihood of HZ in patients with IBD when compared to non-IBD controls. The rating was eventually downgraded to low due to study limitations (detection bias, residual confounding factors) and indirectness (HZ as main outcome). In particular, IBD patients may have more frequent outpatient visits and/or hospitalization than non-IBD patients. This may lead to over-estimation of the risk of HZ in IBD patients related to increased health care utilization (detection bias). As well, all studies reported HZ as the main outcome, but did not report on more severe complications related to HZ (e.g. post-hepatic neuralgia, herpes zoster ophthalmicus, herpes zoster oticus, necrotizing retinitis, cranial and peripheral nerve palsies, myelopathy, meningoencephalitis, cerebellitis, and visceral involvement including pneumonitis, hepatitis, and acute retinal necrosis), which are more important to patients than a self-limited rash (shingles). **In summary, there is low certainty evidence that adult IBD patients have an increased risk of HZ infection compared to non-IBD patients.**

IBD patients stratified by age

Six case-control studies (considered as prognostic studies) provided data on the risks of HZ stratified by age.[2-5,7,9] All studies showed an increased risk of HZ with age. The only low risk of bias study (Khan 2018) suggested that the incidence of HZ among the younger IBD patients (age < 50) exceeded that of older control patients (age > 50) for whom the recombinant zoster vaccine (RZV, Shingrix®) is currently recommended by ACIP as the preferred vaccine for prevention of HZ and related complications. The rating of evidence for this subgroup analysis was further downgraded to very low due to imprecision since only a small number of IBD patients age < 50 had HZ. **In summary, there is low certainty evidence that adult IBD patients older than age 50 have an increased risk of HZ infection compared to non-IBD patients older than age 50. There is very low certainty evidence that adult IBD patients younger than age 50 have an increased risk of HZ infection compared to non-IBD patients older than age 50.**

IBD patients stratified by medication groups

Five case-control studies and one systematic review of RCTs and observational studies (considered as prognostic studies) provided data on the risks of HZ stratified by medication groups.[2-3,7-9,13] The use of steroids, thiopurines, anti-TNF, and combination therapy were associated with increased risks of HZ among IBD patients compared to IBD patients on no treatment / 5ASA or to the general population. The rating of evidence for this subgroup analysis was further downgraded to very low due to imprecision of effect estimates for anti-TNF and thiopurines with wide CIs, inconsistency with some studies suggesting no increased risks for HZ with anti-TNF and thiopurines, and residual confounding with no adjustment for disease flare or severity in most studies. **In summary, there is very low certainty evidence that steroids, combination therapy (steroids + thiopurines or anti-TNF, steroids + thiopurines + anti-TNF, thiopurines + anti-TNF), thiopurines alone, and anti-TNF alone were associated with increased risks of HZ among IBD patients.**

Three single-arm RCTs (considered as prognostic studies) provided data on the incidence of HZ associated with the use of vedolizumab, ustekinumab, and tofacinib.[10-12] There is **very low certainty evidence that tofacinib is associated with an increased incidence of HZ in IBD patients, but vedolizumab and ustekinumab are not.**

There are a number of case reports of HZ in IBD patients complicated by involvement of organs other than the skin, including the central nervous system (CNS), eyes, and lungs.[14] All reported patients were on immunosuppressive medications (steroids, immunomodulators, anti-TNF monotherapy, combination of immunomodulators and anti-TNF, combination of immunomodulators, steroids and anti-TNF). Due to lack of comparison groups and high risk for selection bias, these studies were not included in the evidence profile.

Risk of Bias Table - Adults

**Prognostic studies**							
**SR of single arms RCT and observational studies**							
**Study**	**Study sample adequately represents the population of interest**	**Study data available adequately represent the study sample**	**Prognostic factor measured in a similar and valid way for all**	**Outcome of interest is measured in a similar and valid way for all**	**Important potential confounding factors are appropriately**	**Statistical analysis is appropriate, and all primary outcomes are**	**Comments**

		(>80% follow-up)	participants	participants	accounted for	reported	
Marra 2016 (immune-mediated diseaess)	Highly selected patient populations in RCTs. But observational studies are representative of population of interest.	Dropout rates > 20% in 18 of 40 RCTs. Greater dropouts in the placebo arm due to lack of efficacy, which could have resulted in lower HZ events in placebo arm.	Not across different study designs.	HZ was not a pre-defined endpoint in the studies. Outcome either reported as a serious adverse event or an adverse event in RCTs, and rather than being reported as a separate entity, it was often reported under other categories such as skin infection.	Confounding factors not accounted for in the analysis of RCT data.<br><br>Eligible observational studies were those providing adjusted or propensity score-matched associations. But confounding factors accounted for were highly variable among studies.	OK	• SR of 40 RCTs (20, 136 patients) and 19 observational studies (810,939 patients) assessing the risk of HZ in individuals on biologics, disease-modifying antirheumatic drugs (DMARDs), or steroids for autoimmune diseases (rheumatoid arthritis, psoriasis, psoriatic arthritis, systemic lupus erythematosus, **IBD**)<br>• 4 RCTs (n = 2176) and 3 observational studies in IBD (n = 17,361)<br>• **Biologics**: 28 RCTs (n = 12,272) and 6 observational studies (n = 132,647). **Biologics were associated with an increased risk of HZ in RCTs (OR 1.71; 95% CI 1.11-2.64) and in observational studies (OR 1.58, 95% CI 1.39-1.81).** Increased risks with non-TNF blockers (OR 2.19, 95% CI 1.2-4.02), but not anti-TNF blockers (OR 1.28, 95% CI 0.69-2.40) in RCTs.<br>• **Non-biological DMARDs**: 16 RCTs and 6 observational studies. Increased risks of HZ with DMARDs (OR 1.21, 95% CI 1.15-1.28) in observational studies, but not in RCTs (OR 1.61, 95% CI 0.84-3.10)<br>• **Tofacitinib**: increased risks with 10mg BID dose (OR 3.01, 95% CI 1.15-7.87) in RCTs, but few

							studies examining other doses<br>• **Steroids**: increased risks with steroids (OR 1.73, 95% CI 1.57-1.89) in observational studies. No RCTs.
**Cohort – nested case control studies**							
**Study**	**Study sample adequately represents the population of interest**	**Study data available adequately represent the study sample (>80% follow-up)**	**Prognostic factor measured in a similar and valid way for all participants**	**Outcome of interest is measured in a similar and valid way for all participants**	**Important potential confounding factors are appropriately accounted for**	**Statistical analysis is appropriate, and all primary outcomes are reported**	**Comments**
Nugent 2019 (Canada)	Both IBD cases and non-IBD controls were identified from the University of Manitoba IBD Epidemiology Database. The database includes all IBD patients in Manitoba, and unaffected controls drawn from the Manitoba Health database matched 10:1 to every IBD case by date of diagnosis, age, sex, and geographic	OK	OK	Data were reliant on administrative claim codes. Possible misclassification errors due to errors of miscoding, and the codes have not been previously validated.	Accounted for HZ vaccination.<br><br>Not adjusted for comorbidities may lead to overestimation of the risk of HZ in IBD.<br><br>Detection bias: Patients with IBD may have more outpatient visits and hospitalization than non-IBD controls. This may lead to overestimation of the risk of HZ in IBD patients.	OK	• Population-based case-control study in Manitoba (4998 IBD patients, 34,186 non-IBD controls) from 1984-2016<br>• **Increased risk of HZ infection in IBD patients vs. non-IBD controls (HR 1.42, 95% CI 1.30-1.55 before diagnosis; HR 1.52, 95% CI 1.41-1.63 after diagnosis).**<br>• **HZ rates prior to 2009 (before availability of vaccine): 9.2/1000 person-years in IBD vs. 7.2/1000 person-years in controls (P < 0.0001)**

	residence.						
Khan 2018 (US)	Cohort 1: Non-IBD controls were from Corporate Data Warehouse matched 1:4 to every IBD case by geographic location, year of first outpatient receipt of care in the VA, age, and gender.<br><br>Cohort 2: from the same national VA data in VA informatics and Computing Infrastructure.	OK	OK	Data were from administrative claim data, but validated by performing manual review of records of 200 randomly selected patients who had HZ and IBD diagnostic codes (diagnosis confirmed in 91% and 94.5% respectively).	Cohort 1: adjusted for geographic location, health care use (# of outpatient and inpatient visits), race, and many baseline comorbid conditions.<br><br>Cohort 2: adjusted for age at index date, intensity of prednisone use, oral prednisone use within 30 days before index date, a proxy for flare (hospitalization, iv steroid, CT, stool Clostridium difficile toxin testing), geographic location, IBD diagnosis, health care use, race, gender, baseline comorbid conditions.<br><br>Censored for herpes zoster vaccination.	OK	• 2 retrospective cohort studies among patients in the VA system (**older male population**) from 2000-2016<br>• Cohort 1 (13,001 IBD patients on 5ASA, no steroids vs. 35, 510 Non-IBD controls)<br>• **Increased risk of HZ in IBD patients on 5ASA alone vs. non-IBD controls (AHR 1.72, 95% CI 1.51-1.96).** No difference between UC vs. CD (AHR 1.81, 95% CI 1.56-2.11 vs. AHR 1.56, 95% CI 1.28-1.91).<br>• HZ IR 7.55/1000 person years in IBD vs. 3.22/1000 person-years in controls. **Incidence of HZ among the youngest age group (< 50) of patients with IBD exceeded that of oldest group (>60) of control patients (8.62 per 1000 person-years vs. 3.3 per 1000 person-years)** (See table below)<br>• Cohort 2 (50,962 IBD patients on 5ASA vs. 13,174 IBD patients on thiopurines; 6653 on anti-TNF; 2534 on thiopurines + anti-TNF; 167 on vedolizumab)<br>• **Compared to 5ASA alone, increased risk of HZ with thiopurines (AHR 1.47, 95% CI 1.31-1.65), thiopurines + anti-**

							**TNF (AHR 1.65, 95 % CI 1.22-2.23), cumulative (AHR 1.02, 95% CI 1.01-1.03) and short term (AHR 1.27, 95% CI 1.10-1.48) steroid use, but not anti-TNF alone (AHR 1.15, 95% CI 0.96-1.38)** (See table below)<br>• Increased risk of HZ infection with increased age (AHR 1.01, 95% CI 1.01-1.02) and disease flare (AHR 3.69, 95% CI 3.22-3.43)<br>• **Incidence rates of HZ in all age groups and all IBD medication subgroups were substantially higher than that in the oldest group of patients without IBD (age > 60).**<br>• No HZ infection with vedolizumab
Chang 2018 Korea	Population-based study: Both IBD and non-IBD controls were drawn from the Korean national health insurance claims database.<br><br>Nested case control study: IBD controls without HZ were matched to IBD patients	OK	Population based study: data were reliant on administrative claim codes. Possible misclassification errors due to errors of miscoding, and the codes have not been previously validated.	Population based study: data were reliant on administrative claim codes. Possible misclassification errors due to errors of miscoding, and the codes have not been previously validated.	Population based study: Did not adjust for health care use (# of outpatient and inpatient visits) and baseline comorbid conditions. This may lead to overestimation of the risk of HZ in IBD patients.<br><br>Detection bias: Patients with IBD may have more outpatient visits and hospitalization	OK	• Population-based study and hospital-based, nested case control study in Korea from 2009-2013<br>• Population-based case control study: 38,039 IBD patients vs. entire Korean population<br>• **Increased risk of HZ in IBD patients vs. general population (SIR 1.48, 95% CI 1.42-1.54).**<br>• **The incidence rate of HZ in IBD patients (IR 18.34/1000 person-years, 95% CI 17.60-19.09) vs. general population (IR 11.29/1000 person-years, 95% CI 11.27-11.30)**

	with HZ 3:1 by sex, IBD subtype, age at IBD diagnosis, calendar year of IBD diagnosis.				than non-IBD controls. This may lead to over-estimation of the risk of HZ in IBD patients.<br><br><u>Nested case control study:</u> adjusted for disease location, phenotype, surgery, hospitalization with 1 year before HZ diagnosis, and medication use. Did not adjust for disease flare or severity.		• **The age-specific IR of HZ in IBD patients increased with age, but age-specific SIR of zoster in IBD patients was higher in younger patients and decreased with age.** See Table<br>• <u>Nested case control study</u> in a tertiary care center: 300 IBD patients with HZ vs. 895 IBD patients with no HZ<br>• **Use of steroid was associated with HZ in UC (AOR 2.44, 95% CI 1.18-5.05) and CD (AOR 2.70, 95% CI 1.25-5.83), but not anti-TNF or thiopurines**
Yun 2016 US	Both cases and controls were from a national multi-payer claims database that incorporated public and private data.	OK	OK	Data were from administrative claim data, but validated with high sensitivity and positive predictive values (>85%) for identifying incident HZ.	Adjusted for age, gender, race, and vaccination used.<br><br><u>Detection bias</u> when compared with healthy controls as patients with autoimmune diseases may have more outpatient visits and hospitalization. This may lead to over-estimation of the risk of HZ in IBD patients. Less likely	OK	• Population-based case control study using a national multi-payer claims database from 2007-2010<br>• <u>Cases:</u> **7858 IBD**, 8320 systematic lupus erythematosus, 50,269 rheumatoid arthritis, 2609 psoriasis, 4272 psoriatic arthritis, 1011 ankylosing spondylitis)<br>• <u>Controls:</u> 212,806 diabetes, 328,580 no autoimmune disease controls<br>• **The age-specific rate HZ for systematic lupus erythematosus, IBD and**

					to have detection bias when compared with diabetes control.<br><br>Did not adjust for health care use (# of outpatient and inpatient visits) and baseline comorbid conditions. This may lead to overestimation of the risk of HZ in IBD patients.		**rheumatoid arthritis in their 20s, 30s, and 40s, was comparable or substantially higher than the corresponding rate in adults without autoimmune disease aged ≥ 60** (See table below).
Tsai 2015 Taiwan	Both IBD cases and non-IBD controls were identified from the Taiwan National Health Insurance Research Database, a compulsory and universal health insurance programme. Controls were matched 4:1 to every IBD case by age, sex, and year of IBD diagnosis.	Ok	Data were reliant on administrative claim codes. Possible misclassification errors due to errors of miscoding, and the codes have not been previously validated.	Data were reliant on administrative claim codes. Possible misclassification errors due to errors of miscoding, and the codes have not been previously validated.	Adjusted for age, sex, comorbidities of depression, diabetes, obesity, renal disease, rheumatoid arthritis and malignancy.<br><br>Not adjusted for vaccination use.<br><br>Detection bias: Patients with IBD may have more outpatient visits and hospitalization than non-IBD controls. This may lead to over-estimation of the risk of HZ in IBD patients. The study	OK	• Population-based case-control study in Taiwan (7055 newly diagnosed IBD patients, 28,220 non-IBD controls) from 2000-2010<br>• **Increased risk of HZ among IBD patients vs. non-IBD controls (AHR 1.42, 95% CI 1.27-1.60).**<br>• HZ IR 8.23/1000 person years in IBD vs. 5.74/1000 person-years in controls<br>• Age-specific analysis showed that IBD patients had a higher risk of developing HZ than that of the non-IBD controls, except for the 20-34 age group.

					showed that the overall risk of developing HZ positively correlated with the frequency of medical visits.		
Forbes 2014 UK	Both cases and controls were identified from the UK Clinical Practice Research Datalink (GPRD), a primary care database, containing data on approximately 7% of the UK population, broadly representative of patients' and practices' characteristics in the UK. Controls were matched 4:1 by practice, sex, and age.	Multiple imputation by chained equations to account for missing data (11% had missing data for alcohol and smoking)	Possible misclassification errors as no chart validation.	Possible misclassification errors as no chart validation.	Adjusted for age, sex, practice, immunosuppressive treatments, inhaled steroids, socioeconomic status, and comorbidities.<br><br>Detection bias: patients with IBD may have more frequent outpatient visits than non-IBD patients. This may lead to over-estimation of the risk of HZ in IBD patients.	OK	• Case-control study in the UK (base study population consisted of all patients aged 18 or over with no previous HZ; 144, 959 patients with incident HZ, 549,336 controls with no previous history of HZ) from 2000-2011<br>• Approximately 45% of HZ occurred in patients under 60 years, 27% under 50 years, 7% under 30 years<br>• **Increased risk of HZ among IBD patients vs. controls (AHR 1.28, 95% CI 1.18-1.38).** (See Table)<br>• **The relative risks of HZ increase with decreasing age with AOR 1.18, 1.30, 1.40, 1.73 among patients aged ≥ 70 years, 60-69 years, 50-59 years, < 50 years. Yet, the rates remained low among patients aged 18-49 years (3.59 per 1000 person years, 95% CI 2.56-5.04)** (See Table)
Long 2013 US	Case-control study: Both cases and controls were identified from	OK	OK	Data were from administrative claim data, but validated with high sensitivity	Case-control study: Adjusted for health care utilization and comorbidities.	OK	• Two case-control studies in the US using a private administrative claim database from 1997-2009

	the IMS LifeLink Information Assets-Health Plan Claims Database. Non-IBD controls were matched to IBD patients 4:1 by US census region, sex, and age.<br><br>Nested case-control study : IBD controls without HZ were matched to IBD patients with HZ 4:1 by geographic region, sex, age, disease type, and duration of follow-up.			(98%) and positive predictive values (93%) for identifying HZ.	Nested case-control study: Did not adjust for disease severity or flare.<br><br>Not adjusted for vaccination use.<br><br>Detection bias when compared with non-IBD controls as IBD patients may have more outpatient visits and hospitalization. This may lead to over-estimation of the risk of HZ in IBD patients.		(representative of the national commercially insured population) aged ≤ 65<br>• Case-control study: IBD patients (108,604) vs. non-IBD controls (434,416)<br>• **Increased risk of HZ for IBD patients compared to non-IBD controls (AHR 1.49, 95% CI 1.42-1.57). Increasing incidence of HZ within each strata of age with the highest incidence in the 60+ age strata.** See Figure.<br>• Nested case-control study: IBD patients with HZ (2,659) vs. IBD patients without HZ (10,470)<br>• **Use of thiopurine (AOR 1.85, 95% CI 1.61-2.13), steroid (AOR 1.73, 95% CI 1.51-1.99), and anti-TNF (AOR 1.81, 95% CI 1.48-2.21) were independently associated with HZ. Combination therapy with thiopurine and anti-TNF has the highest risk (AOR 3.29, 95% CI 2.33-4.65). 5-ASA use was not associated with HZ (AOR 1.08, 95% CI 0.97-1.19).**
Zhang 2012 US	Both cases and controls were from Medicare medical and pharmacy claims data.	OK	OK	Positive predictive value of the HZ diagnosis code alone to identify incident case of HZ (using medical review as a gold standard) has	Adjusted for gender, race, immune-mediated disease, time-varying concurrent medications, and health care utilization	OK	• Retrospective cohort study among Medicare beneficiaries diagnosed with immune-mediated diseases including rheumatoid arthritis (292,169), psoriatic arthritis, psoriasis (11,030), ankylosing spondylitis

				been shown to range between 80% and 100%.	(hospitalization and physician visits).<br><br>Did not adjust for disease severity or flare.<br><br><u>Detection bias</u>: did not adjust for the number of outpatient and inpatient visit.		(4,026), or **IBD (66,751)** aged ≥ 60 from 2006-2009<br>• Median duration of follow-up 2.0 (0.8-3.0) years<br>• 4% (18,683) received the **HZ vaccine (LZV, Zostavax)** vs. unvaccinated (444,858)<br>• **Among the unvaccinated group, exposure to oral steroids was associated with a 1.1-2.0-fold greater risk of HZ; the increase was significant for most medication groups (anti-TNF, DMARDs without biologics)** (See Table)
Marehbian 2009 US	Both cases and controls were identified from private health-care insurance administrative claim data in the US. Non-IBD controls were matched to IBD patients 5:1 by age, gender, health plan, availability of follow-up, CD patients' index date.	OK	Possible <u>misclassification errors</u> due to errors of miscoding, and the codes have not been previously validated.	Possible <u>misclassification errors</u> due to errors of miscoding, and the codes have not been previously validated.	Adjusted for age, gender, health plan, region, and year.<br><br>Did not adjust for comorbidities, disease severity or flare.<br><br><u>Detection bias:</u> Patients with IBD may have more outpatient visits and hospitalization than non-IBD controls. This may lead to over-estimation of the risk of HZ in IBD patients.	OK	• Case-control study and longitudinal cohort study (nested case-control study) in the US from 2002-2005<br>• <u>Case-control study</u>: 22, 310 CD patients vs. 111,550 non-CD controls<br>• **Increased risk of HZ for IBD patients compared to non-IBD controls (RR 1.83, 95% CI 1.65-2.04).**<br>• **Increased risk of HZ for IBD patients compared to non-IBD controls among all treatment and no treatment subgroups:** steroids (RR 5.53, 95% CI 3.83-7.99), IS (RR 2.54, 95% CI 1.86-3.46), anti-TNF (RR 2.90, 95% CI 1.72-4.89), anti-TNF + steroids (RR 7.23, 95% CI 2.51-20.84), anti-TNF + IS (RR 4.15, 95% CI 2.24-7.71),

							steroids + IS (RR 7.01, 95% CI 4.31-11.38), anti-TNF + IS + steroids (RR 7.07, 95 CI 2.45-20.38), no treatment (RR 1.57, 95% CI 1.39-1.77)<br>• Nested case-control study: 8581 CD patients with no CD diagnosis for at least 1 year before the index date<br>• **Compared to IBD patients on no therapy, increased risk of HZ with steroids (HR 3.11, 95% CI 1.57-6.17) and combination with any 2 or more (HR 3.68, 95% CI 1.82-7.46), but not with IS (HR 1.04, 95% CI 0.46-2.36) or anti-TNF (HR 1.33, 95% CI 0.42-4.25).**
Gupta 2006 UK	Both cases and controls were identified from the UK Clinical Practice Research Datalink (GPRD), a primary care database, containing data on approximately 6% of the UK population, broadly representative of patients' and practices' characteristics	OK	Possible misclassification errors as no chart validation.	Possible misclassification errors as no chart validation.	Adjusted for smoking, alcohol, cancer, and depression.<br><br>Did not adjust for disease severity or flare, or other comorbidities.<br><br>Detection bias: patients with IBD may have more frequent outpatient visits than non-IBD patients. This may lead to over-estimation of the risk of HZ in IBD patients.	OK	• Two case-control studies in the UK from 1988-1997<br>• Case control study: IBD patients (7823 CD and 11,930 UC) vs. non-IBD controls (79,563)<br>• **Increased risk of HZ for both CD (IRR 1.6, 95% CI 1.4-1.9) and UC (IRR 1.2, 95% CI 1.1-1.4) compared to non-IBD controls.** The significant differences in IRs were limited to patients with CD in the age groups 15-44, 45-64, and > 65.<br>• Nested case-control study: IBD patients with HZ (451) vs. IBD patients without HZ (1787)<br>• **Use of steroid (AOR 1.5, 95%**

	in the UK.<br><br><u>Case-control study:</u> Non-IBD controls were matched to IBD patients 4:1 by practice, sex, and age.<br><br><u>Nested case-control study</u>: IBD controls without HZ were matched to IBD patients with HZ 4:1 by practice, sex, and age.						**CI 1.1-2.2) and AZA/6MP (AOR 3.1, 95% CI 1.7-5.6) were associated with the risk of HZ.** Mesalamine was not associated with the risk of HZ (AOR 0.9, 95% CI 0.7-1.2). Did not examine biologics.
**Single arms RCTs**							
**Study**	**Study sample adequately represents the population of interest**	**Study data available adequately represent the study sample (>80% follow-up)**	**Prognostic factor measured in a similar and valid way for all participants**	**Outcome of interest is measured in a similar and valid way for all participants**	**Important potential confounding factors are appropriately accounted for**	**Statistical analysis is appropriate, and all primary outcomes are reported**	**Comments**
Hanauer 2019	Highly selected patient populations in RCTs. Included patients who tolerated and responded to ustekinumab.	OK	OK	HZ was not a pre-defined endpoint in the studies. Outcome either reported as a serious adverse event or an adverse event in RCTs, and rather than being reported as a	Confounding factors not accounted for in the analysis of RCTs data.	OK<br><br>Reported serious infections including anal abscess, peri-rectal abscess, cellulitis, gastroenteritis, peri-rectal	• Observational data from **ustekinumab** Phase III/open label long term extension CD clinical trials<br>• Average duration of follow-up 141 weeks in Ustekinumab patients<br>• **0/567 (0%) patients treated with ustekinumab developed HZ**

				separate entity, it was often reported under other categories such as skin infection.		abscess, pyelonephritis, and sepsis. Did not specifically report on HZ.	
Winthrop 2018	Highly selected patient populations in RCTs. Included patients who tolerated and responded to tofacitinib.	OK	OK	HZ events were graded as serious infection events if they met serious adverse event criteria. Serious adverse events, or those reported to involve > 1 dermatome were sent to an independent, blinded, external adjudication committee.	Confounding factors not accounted for in the analysis of RCT data.	OK	• Observational data from **tofacitinib** Phase II/III/open-label, long term extension UC clinical trials<br>• Open label long-term extension study with treatment up to 47 months<br>• 65 (5.6%) / 1157 patients treated with tofacitinib developed HZ: majority were single dermatomes, 11 had multi-dermatomal, 1 encephalitis.<br>• **HZ in the overall tofacitinib cohort: IR 4.07 per 100 patient-years of exposure (95% CI 3.14-5.19) vs. IR 0.97 per 100 patient years (0.02-5.42) in the placebo maintenance cohort**<br>• IRs highest in patients age ≥ 65, Asian patients, patients with prior anti-TNF failure, and tofacinitib 10mg bid dose.
Columbel 2017	Highly selected patient populations in RCTs. Included patients who tolerated and responded to	OK	OK	HZ was not a pre-defined endpoint in the studies. It may be reported as skin infection or under other categories.	Confounding factors not accounted for in the analysis of RCT data.	OK	• Observational data from 6 **vedolizumab** UC or CD trials (2 phase 2 and 4 phase 3 studies including open-label trials)<br>• Vedolizumab exposure median 365 days<br>• 35/2830 (1.2%) who received

	vedolizumab						vedolizumab<br>• 2/504 (0.4%) received placebo (but may have previously received vedolizumab)<br>• **HZ in the overall vedolizumab cohort: IR 0.7 per 100 patient-years of exposure (95% CI 0.5-1.0) vs. IR 0.9 per 100 patient years (0-2.2) in the placebo**

Evidence Profile Table - Adults

Certainty Assessment								Summary of Findings	
Studies	Risk of bias	Inconsistency	Indirectness	Imprecision	Other considerations	Certainty of Evidence	Overall Certainty of evidence	Study Event Rates	Relative Effect (95% CI)
**VPI (Herpes Zoster) - CRITICAL**							⨁⨁⊖⊖<br>LOW		
9<br>Case-control studies (prognostic studies)[1-9]<br><br>**IBD patients**	Serious[a]	Not serious	Serious[b]	Not serious	None	⨁⨁⊖⊖<br>LOW		• See **Summary of case-control studies assessing the risk of HZ in IBD vs. non-IBD patients** and **Summary of case-control studies assessing the factors associated with increased risk of herpes zoster.** All studies found an increased risk of HZ in IBD patients (1.2-1.8 times) compared to the general population (non-IBD controls). Overall certainty of evidence is anchored to this comparison - **Low**<br>• See **Summary of case-control studies assessing the risks of herpes zoster infection (HZ) in IBD vs. non-IBD patients stratified by age.** All studies showed an increased risk of HZ with age. The only low risk of bias study (Khan 2018) suggested that the Incidence of HZ among the youngest IBD patients (age <50) exceeded that of older groups of control patients (age > 50). Certainty of evidence downgraded to **very low** due to imprecision as small number of IBD patients age < 50 had HZ (n = 35).<br>• See **Summary of case-control studies assessing the risks of**	

								**herpes zoster infection (HZ) in IBD patients stratified by medication groups.** Steroids, thiopurines, anti-TNF, and combination therapy were associated with increased risks of HZ among IBD patients compared to no treatment or to the general population. Certainty of evidence downgraded to **very low** due to imprecision of effect estimates of anti-TNF and thiopurines (wide CIs), inconsistency with some studies suggesting no increased risks for HZ with anti-TNF and thiopurines, and residual confounding with no adjustment for disease severity or flare for most studies.
3<br>Single arm RCTs<br>(prognostic studies)[10-12]<br><br>**IBD patients**	Serious[c]	Not serious	Serious[d]	Serious[e]	None	⊕⊖⊖⊖<br>VERY LOW		• Ustekinumab: 0/567 (0%) patients treated with ustekinumab developed HZ<br>• Vedolizumab: IR 0.7 per 100 patient-years (95% CI 0.5-1.0) in the Vedolizumab cohort vs. IR 0.9 per 100 patient-years (95% CI 0-2.2) in the placebo<br>• Tofacitinib: IR 4.07 per 100 patient-years (95% CI 3.14-5.19) in the Tofacitinib cohort vs. IR 0.97 per 100 patient-years (95% CI 0.02-5.42) in the placebo cohort
1 SR of RCTs and observational studies (40 RCTs and 19 observational studies) (prognostic studies)[13]<br><br>**Patients with autoimmune diseases including IBD**	Serious[f]	Serious[g]	Serious[h]	Not serious	None	⊕⊖⊖⊖<br>VERY LOW		• Biologics: Biologics were associated with an increased risk of HZ than control in RCTs (OR 1.71; 95% CI 1.11-2.64) and in observational studies (OR 1.58, 95% CI 1.39-1.81). Increased risks with non-TNF blockers (OR 2.19, 95% CI 1.2-4.02), but not anti-TNF blockers (OR 1.28, 95% CI 0.69-2.40) in RCTs.<br>• Non-biological DMARDs: Increased risks of HZ with DMARDs (OR 1.21, 95% CI 1.15-1.28) in observational studies, but not in RCTs (OR 1.61, 95% CI 0.84-3.10)<br>• Tofacitinib: increased risks with 10mg BID dose (OR 3.01, 95% CI 1.15-7.87) in RCTs, but few studies examining other doses<br>• Steroids: increased risks with steroids (OR 1.73, 95% CI 1.57-1.89) in observational studies. No RCTs.

Footnotes:

a. Downgraded for study limitations. High risk for detection bias for all except 1 study (Khan 2018) as patients with IBD may have more frequent outpatient visits and/or hospitalization than non-IBD patients (general population). This may lead to over-estimation of the risk of HZ in IBD patients. Most studies did not adjust for health care utilization and comorbidities which are potential confounders for the association between IBD and HZ.

b. Downgraded for indirectness related to outcome. All studies reported HZ as the main outcome, but did not report on more severe complications related to HZ (e.g. post-hepatic neuralgia, herpes zoster ophthalmicus, herpes zoster oticus, necrotizing retinitis, cranial and peripheral nerve palsies, myelopathy, meningoencephalitis, cerebellitis, and visceral involvement including pneumonitis, hepatitis, and acute retinal necrosis), which are more important to patients than a self-limited rash (shingles).

c. Downgraded for study limitations. HZ was not a predefined endpoint for most studies. Outcome either reported as a serious adverse event or an adverse event in RCTs, and rather than being reported as a separate entity, it was often reported under other categories such as skin infection. In

open label extension cohort of these studies, the outcome assessors were not blinded to treatment. Confounding factors were also not accounted for in the analysis of RCT data.

d. Downgraded for indirectness. Highly selected patient populations who tolerated and responded to biologic treatments.
e. Downgraded for imprecision. Low event rates.
f. Downgraded for study limitations. Dropout rates > 20% in 18 of 40 RCTs. Greater dropouts in the placebo arm due to lack of efficacy, which could have resulted in lower HZ events in the placebo arm. Confounding factors not accounted for in the analysis of RCT data. Eligible observational studies were those providing adjusted or propensity score-matched associations. But confounding factors accounted for were highly variable among observational studies. HZ is not a predefined endpoint for most RCTs. Outcome either reported as a serious adverse event or an adverse event in RCTs, and rather than being reported as a separate entity, it was often reported under other categories such as skin infection.
g. Downgraded for inconsistency of some findings between RCTs and observational studies.
h. Downgraded for indirectness. Studies included different autoimmune diseases (rheumatoid arthritis, psoriasis, psoriatic arthritis, systemic lupus erythematosus, IBD).

Summary of case-control studies assessing the risk of herpes zoster infection (HZ) in IBD vs. non-IBD patients

Study	IBD patients	Non-IBD control	Incidence rates of HZ in IBD patients	Incidence rates of HZ in non-IBD controls	Adjusted Incidence rate ratios (IRR) / Hazards Ratio (HR) for HZ in IBD patients (95% CI)
Nugent 2019[1] Canada	27,283 person-years	191,205 person-years	9.2 / 1000 person-years	7.2 / 1000 person-years	Increased risk of HZ in IBD patients: HR 1.42 (1.30-1.55) before IBD diagnosis HR 1.52 (1.41-1.63) after IBD diagnosis
Khan 2018[2] US	45,510 person-years	334,017 person-years	7.6 / 1000 person-years	3.2 / 1000 person-years	Increased risk of HZ in IBD patients: AHR 1.72 (1.51-1.96)
Chang 2018[3] Korean	127,621 person-years	250,552,299 person-years	18.3 / 1000 Person-years	11.3 / 1000 person-years	Increased risk of HZ in IBD patients: SIR 1.48 (1.42-1.54)
Yun 2016[4] US	7858 patients	328,580 controls	13 / 1000 person-years	5.3 / 1000 person-years	Increased risk of HZ in IBD patients: No IRR/HR provided
Tsai 2015[5] Taiwan	7055 patients	28,220 controls	8.23 / 1000 person-years	5.74 / 1000 person-years	Increased risk of HZ in IBD patients: AHR 1.42 (1.27-1.60)
Long 2013[7] US	364,533 person-years	992,273 Person-years	734 / 100,000 person-years	437 / 100,000 person-years	Increased risk of HZ in IBD patients: AHR 1.49 (1.42-1.57)
Marehbian 2009[8] US	22,310 patients	111,550 controls	89 / 10,000 person-years	48 / 10,000 person-years	Increased risk of HZ in CD patients: RR 1.83 (1.65-2.04)
Gupta 2006[9] UK	19,753 patients	79,563 controls	-	-	Increased risk of HZ in IBD patients: CD: IRR 1.6 (1.4-1.9) UC: IRR 1.2 (1.1-1.4)

Summary of case-control studies assessing the factors associated with increased risk of herpes zoster

Study	Cases with HZ	Controls without HZ	AOR (99% CI)
Forbes 2014[6]	1851 /144,959 (1.3%) IBD patients	5118/549,336 controls (0.9%) IBD patients	1.28 (1.18-1.38)<br>IBD is a risk factor for zoster

Summary of case-control studies assessing the risks of herpes zoster infection (HZ) in IBD vs. non-IBD patients stratified by age

ACIP recommends two-dose recombinant zoster vaccine (RZV, Shingrix®) at age 50 for the general population

Study	Incidence rates of HZ in IBD patients Age < 50	Incidence rates of HZ in IBD patients Age 50-60	Incidence rates of HZ in IBD patients Age > 60	Incidence rates of HZ in non-IBD controls Age 50-60	Incidence rates of HZ in non-IBD controls Age > 60	Comments
Khan 2018[2]<br>US	8.6 / 1000 person-years	9.0 / 1000 person-years	7.2 / 1000 person-years	3.4 / 1000 person-years	3.3 / 1000 person-years	Incidence of HZ among the youngest IBD patients (age <50) exceeded that of older groups of control patients (age > 50)
Chang 2018[3]<br>Korean	Age 0-19: 8.87 / 1000 person-years<br>Age 20-29: 11.32 / 1000 person-years<br>Age 30-39: 13.19 / 1000 person-years<br>Age 40-49: 16.81 / 1000 person-years	27.93 / 1000 person-years	Age 60-69: 28.38 / 1000 person-years<br>Age ≥ 70: 29.75 / 1000 person-years	19.0 / 1000 person-years	23.68 / 1000 person-years	Incidence of HZ among IBD patients (age 50-60) exceeded that of control patients (age 50-60). But the IRs of HZ in non-IBD controls are higher than that expected in western populations. In fact, the IR of HZ among IBD patients at any age exceeded that of the IR of HZ at age 50 in the US (7/1000 person-years).
Yun 2016[4]	Age 21-30: 11.6	11.7 / 1000	Age 61-70: 19.0 / 1000	5.8 / 1000	Age 61-70: 8.5 / 1000	Incidence rates of HZ among IBD patients

US	/ 1000 person-years	person-years	person-years	person-years	person-years	of all age groups are either comparable or substantially higher than that of older groups of control patients (age > 50).
	Age 31-40: 5.6 / 1000 person-years		Age > 70: 23.8 / 1000 person-years		Age > 70: 10.6 / 1000 person-years	
	Age 41-50: 10.4 / 1000 person-years					
Tsai 2015[5] Taiwan	Age 20-34: 2.76 / 1000 person-years	Age 54-64: 12.7 / 1000 person-years	Age ≥ 65: 16.0 / 1000 person-years	Age 54-64: 8.79 / 1000 person-years	Age > 65: 10.7 / 1000 person-years	Incidence rates of HZ among IBD patients (age 45-54) exceeded that of older groups of control patients (age 54-64)
	Age 35-44: 5.30 / 1000 person-years					
	Age 45-54: 9.24 / 1000 person-years					
Long 2013[7] US	Age 0-10	Age 51-60	CD: 1502/100,000 person-years	650/100,000 person-years	850/100,000 person-years	Incidence rates were provided in Figure only (with no exact numbers). Incidence of HZ among IBD patients (age 41-50) exceeded that of older groups of control patients (age 50-60).
	Age 11-20					
	Age 21-30					
	Age 31-40					
	Age 41-50					
Gupta 2006[9] UK	Age 0 – 4: 0 / 100,000 person-years	Age 45-64: 664-856 / 100,000 person-years	Age ≥ 65: 1143-1291 / 100,000 person-years	Age 45-64: 534-557 / 100,000 person-years	Age ≥ 65: 906-959 / 100,000 person-years	The significant differences in IRs were limited to patients with CD in the age groups 15-44, 45-64, and 65 and older.
	Age 5-14: 218-321 / 100,000 person-years					
	Age 15-44: 302-432 / 100,000 person-years					

Compared to healthy people aged 50-60, rates were classified as significantly higher or comparable (yellow shading).

Summary of case-control studies assessing the risks of herpes zoster infection (HZ) in IBD patients stratified by medication groups

**Study**	**5ASA alone** Adjusted Incidence rate ratios (IRR) / Hazards Ratio / OR	**Thiopurines** Adjusted Incidence rate ratios (IRR) / Hazards Ratio / OR	**Anti-TNF** Adjusted Incidence rate ratios (IRR) / Hazards Ratio / OR	**Thiopurines + Anti-TNF** Adjusted Incidence rate ratios (IRR) / Hazards Ratio / OR	**Steroids** Adjusted Incidence rate ratios (IRR) / Hazards Ratio / OR	**Steroids + Thiopurines** Adjusted Incidence rate ratios (IRR) / Hazards Ratio / OR	**Steroids + Anti-TNF** Adjusted Incidence rate ratios (IRR) / Hazards Ratio / OR	**Steroids + Anti-TNF + Thiopurines** Adjusted Incidence rate ratios (IRR) / Hazards Ratio / OR	**Comments**
Khan 2018[2] US	Reference	AHR: 1.47 (1.31-1.65)	AHR: 1.15 (0.96-1.38)	AHR: 1.65 (1.22-2.23)	Short term: AHR 1.27 (1.10-1.48) Cumulative: AHR 1.02 (1.01-1.03)	NA	NA	NA	Compared to 5ASA alone, increased risk of HZ with thiopurines, thiopurines + anti-TNF, cumulative and short-term steroid use, but not anti-TNF alone.
Chang 2018[3] Korean	NA	NA	CD: NA UC: AOR 2.08 (0.91-4.73)	NA	CD: AOR 2.70 (1.25-5.83) UC: AOR 2.44 (1.18-5.05)	NA	NA	NA	Use of steroid was associated with HZ in UC and CD, but not anti-TNF or thiopurines.
Long 2013[7] US	AOR 1.08 (0.97-1.19)	AOR 1.85 (1.61-2.13)	AOR 1.81 (1.48-2.21)	AOR 3.29 (2.33-4.65)	AOR 1.73 (1.51-1.99)	NA	NA	NA	Use of thiopurine, anti-TNF, steroid, and combination therapy was associated with HZ, but not 5ASA.
Marehbian 2009[8] US	NA	RR 2.54[a] (1.86-3.46)	RR 2.90 (1.72-4.89)	RR 4.15[a] (2.24-7.71)	RR 5.53 (3.83-7.99)	RR 7.01[a] (4.31-11.38)	RR 7.23 (2.51-20.84)	RR 7.07[a] (2.45-20.38)	Compared to the general population, increased risk of HZ for IBD patients with

									all medications. Compared to IBD patients on no therapy, increased risk of HZ with steroids (HR 3.11, 1.57-6.17), and combination therapy (HR 3.68, 1.82-7.46), but not with immunosuppressants alone (HR 1.04, 0.46-2.36) or anti-TNF alone (HR 1.33, 95% CI 0.42-4.25).
Gupta 2006[9]	AOR 0.9 (0.7-1.2)	AOR 3.1 (1.7-5.6)	NA	NA	AOR 1.5 (1.1-2.2)	NA	NA	NA	Use of steroid or thiopurine was associated with increased risk of HZ. Mesalamine was not associated with the risk of HZ. Did not examine biologics.

Compared to IBD patients on no treatment / 5ASA or general population, rates were classified as significantly higher (yellow shading).

Footnotes:

a. RR for immunosuppressants including thiopurines (mostly) and methotrexate

References:

1. Nugent Z, Singh H, Targownik LE, Bernstein CN. Herpes Zoster Infection and Herpes Zoster Vaccination in a Population-Based Sample of Persons With IBD: Is There Still an Unmet Need? Inflamm Bowel Dis. 2019 Feb 21;25(3):532-540.
2. Khan N, Patel D, Trivedi C, Shah Y, Lichtenstein G, Lewis J, Yang YX. Overall and Comparative Risk of Herpes Zoster With Pharmacotherapy for Inflammatory Bowel Diseases: A Nationwide Cohort Study. Clin Gastroenterol Hepatol. 2018 Dec;16(12):1919-1927.
3. Chang K, Lee HS, Kim YJ, Kim SO, Kim SH, Lee SH, Song EM, Hwang SW, Park SH, Yang DH, Ye BD, Byeon JS, Myung SJ, Yang SK. Increased Risk of Herpes Zoster Infection in Patients With Inflammatory Bowel Diseases in Korea. Clin Gastroenterol Hepatol. 2018 Dec;16(12):1928-1936.

4. Yun H, Yang S, Chen L, Xie F, Winthrop K, Baddley JW, Saag KG, Singh J, Curtis JR. Risk of Herpes Zoster in Autoimmune and Inflammatory Diseases: Implications for Vaccination. Arthritis Rheumatol. 2016 Sep;68(9):2328-37.
5. Tsai SY, Yang TY, Lin CL, Tsai YH, Kuo CF, Kao CH. Increased risk of varicella zoster virus infection in inflammatory bowel disease in an Asian population: a nationwide population-based cohort study. Int J Clin Pract. 2015 Feb;69(2):228-34.
6. Forbes HJ, Bhaskaran K, Thomas SL, Smeeth L, Clayton T, Langan SM. Quantification of risk factors for herpes zoster: population based case-control study. BMJ. 2014 May 13;348:g2911.
7. Long MD, Martin C, Sandler RS, Kappelman MD. Increased risk of herpes zoster among 108 604 patients with inflammatory bowel disease. Aliment Pharmacol Ther. 2013 Feb;37(4):420-9.
8. Marehbian J, Arrighi HM, Hass S, Tian H, Sandborn WJ. Adverse events associated with common therapy regimens for moderate-to-severe Crohn's disease. Am J Gastroenterol. 2009 Oct;104(10):2524-33.
9. Gupta G, Lautenbach E, Lewis JD. Incidence and risk factors for herpes zoster among patients with inflammatory bowel disease. Clin Gastroenterol Hepatol. 2006 Dec;4(12):1483-90.
10. Hanauer SB, Sandborn WJ, Feagan BG, Gasink C, Jacobstein D, Zou B, Johanns J, Adedokun OJ, Sands BE, Rutgeerts P, de Villiers WJS, Colombel JF, Ghosh S. IM-UNITI: 3 Year Efficacy, Safety, and Immunogenicity of Ustekinumab Treatment of Crohn's Disease. J Crohns Colitis. 2019 Jun 3.
11. Winthrop KL, Melmed GY, Vermeire S, Long MD, Chan G, Pedersen RD, Lawendy N, Thorpe AJ, Nduaka CI, Su C. Herpes Zoster Infection in Patients With Ulcerative Colitis Receiving Tofacitinib. Inflamm Bowel Dis. 2018 Sep 15;24(10):2258-2265.
12. Colombel JF, Sands BE, Rutgeerts P, Sandborn W, Danese S, D'Haens G, Panaccione R, Loftus EV Jr, Sankoh S, Fox I, Parikh A, Milch C, Abhyankar B, Feagan BG. The safety of vedolizumab for ulcerative colitis and Crohn's disease. Gut. 2017 May;66(5):839-851.
13. Marra F, Lo E, Kalashnikov V, Richardson K. Risk of Herpes Zoster in Individuals on Biologics, Disease-Modifying Antirheumatic Drugs, and/or Corticosteroids for Autoimmune Diseases: A Systematic Review and Meta-Analysis. Open Forum Infect Dis. 2016 Sep 28;3(4):ofw205.
14. Cullen G, Baden RP, Cheifetz AS. Varicella zoster virus infection in inflammatory bowel disease. Inflamm Bowel Dis. 2012 Dec;18(12):2392-403.

Effectiveness and Safety of recombinant zoster vaccine in IBD patients

Summary - Adults

**PICO 10A**	**In adult patients with IBD (50 years of age and older), should vaccination vs. no vaccination against herpes zoster (recombinant zoster vaccine) be given?**
**Population**	Adult patients with IBD (50 years of age and older)
**Intervention**	Vaccination against herpes zoster (RZV, Shingrix®)
**Comparator**	No vaccination against herpes zoster
**Outcome**	Mortality, VPI (herpes zoster infection and complications), SAEs, Immunogenicity

PICO 10B	**In adult patients with IBD (younger than 50 years of age), should vaccination vs. no vaccination against herpes zoster (recombinant zoster vaccine) be given?**
**Population**	Adult patients with IBD (50 years of age and older)
**Intervention**	Vaccination against herpes zoster (RZV, Shingrix®)
**Comparator**	No vaccination against herpes zoster
**Outcome**	Mortality, VPI (herpes zoster infection and complications), SAEs, Immunogenicity

There was no RCT comparing recombinant (or live attenuated) herpes zoster vaccine (HZV) with placebo or no treatment in adult patients with IBD to address this PICO question.

One case-control study in IBD patients, two case-control studies in patients with selected immune-mediated diseases including IBD, and a before-and-after study in IBD patients addressed this PICO question.[1-4] All assessed the use of live attenuated herpes zoster vaccine (not recombinant herpes zoster vaccine).[1-4] The two larger case-control studies[1,2] that included a larger number of vaccinated patients showed a significant reduction in the risk of HZ (39-46%) following vaccination with the live-attenuated zoster vaccine (LZV, Zostavax®). In a large case-control study, no significant risk reduction of herpes zoster infection was seen among patients vaccinated while on thiopurines compared with patients not vaccinated while on thiopurines (AHR 0.63, 95% CI 0.30-1.33).[1] There were too few patients who were vaccinated while on anti-TNF alone or in combination with a thiopurine to assess the association of their use with effectiveness of the vaccine.[1] The before-and-after study showed that IBD patients can mount an immune response to the live-attenuated zoster vaccine (LZV, Zostavax®) with a significant increase in HZ immunoglobulin G 2 weeks post vaccination, but the response was lower in patients on low dose immunomodulators (methotrexate $\leq$ 0.4mg/kg/week, azathioprine $\leq$ 3.0mg/kg/d, 6MP $\leq$ 1.5mg/kg/d).[4] It is uncertain if this blunted response is clinically relevant and would still afford seroprotection. No serious adverse events were reported with the live attenuated herpes zoster vaccine.[3] The GRADE rating of these studies started as low due to their observational designs. The rating was downgraded to **very low** due to study limitations (selection bias, residual confounding) and indirectness (patient population, intervention, outcome). In particular, HZV may be selectively given to healthier patients (healthy vaccinee effect). This may have led to over-estimation of the protective effect of the vaccine and underestimation of adverse effects. As well, the PICO question pertains to recombinant zoster vaccine (not live attenuated zoster vaccine which was assessed in these studies). The studies reported HZ as the main outcome, but did not report on more severe complications related to HZ (e.g. post-hepatic neuralgia, herpes zoster ophthalmicus, herpes zoster oticus, necrotizing retinitis,

cranial and peripheral nerve palsies, myelopathy, meningoencephalitis, cerebellitis, and visceral involvement including pneumonitis, hepatitis, and acute retinal necrosis), which are more important to patients than a self-limited rash (shingles). The incidence of HZ within 42 days following vaccination with live attenuated zoster vaccine in IBD patients was very low.

The CDC ACIP recommends herpes zoster recombinant vaccine in immunocompetent adults aged 50 years and older for the prevention of HZ and related complications based on **high certainty** of evidence for both safety and effectiveness.[6,7] Since there were studies on clinical effectiveness, safety, and immunogenicity on live attenuated zoster vaccines in age-specific IBD populations that supported the findings in the general populations, the evidence was not downgraded for indirectness related to patient population (general population vs. IBD population). As per CDC, recombinant zoster vaccine is preferred over live attenuated vaccine due to higher effectiveness. Therefore, the evidence was also not downgraded for indirectness related to intervention. The evidence of adult IBD patients aged 50 years and older is therefore anchored to the general population. However, it is possible that herpes zoster vaccine may not be as effective in IBD patients on immunosuppressive medications based on the above evidence. As well, only a small number of IBD patients on different types of immunosuppressive medications were included in the studies.[1-4] Therefore, the evidence was downgraded 1 level to moderate for adult IBD patients on immunosuppressive medications. **Overall, there is <u>high</u> certainty evidence that herpes zoster vaccine is effective in adult IBD patients aged 50 years and older not on immunosuppressive medications. There is <u>moderate</u> certainty evidence that herpes zoster is effective in adult IBD patients aged 50 years and older on immunosuppressive medications. As there is serious imprecision with the estimate of serious adverse events related to use of zoster vaccine in IBD patients and all included studies assessed live attenuated vaccines (not recombinant vaccines), the evidence was downgraded to <u>moderate</u> for safety. Overall, there is <u>moderate</u> certainty evidence that recombinant zoster vaccine is safe and effective in adult IBD patients aged 50 and older on or not on immunosuppressives.**

As there were very few younger IBD patients (age < 50) who received the herpes zoster vaccine in the included studies, the benefits vs. risks of the recombinant zoster vaccine in this patient population are very uncertain. As well, no long-term studies on the duration of vaccine protection have been performed for patients younger than 50 years old. It remains unclear whether adults receiving the vaccine before age 50 will continue to be protected as they age. **If the data is extrapolated from adults age > 50, the evidence would be downgraded to low due to indirectness (absolute risk of HZ in adults age < 50 is lower than adults age > 50; uncertain duration of protection).**

References:

1. Khan N, Trivedi C, Kavani H, Medvedeva E, Lewis J, Yang YX. Efficacy of Live Attenuated Herpes Zoster Vaccine in Patients With Inflammatory Bowel Diseases. Clin Gastroenterol Hepatol. 2019 Jun;17(7):1341-1347.

2. Zhang J, Xie F, Delzell E, Chen L, Winthrop KL, Lewis JD, Saag KG, Baddley JW, Curtis JR. Association between vaccination for herpes zoster and risk of herpes zoster infection among older patients with selected immune-mediated diseases. JAMA. 2012 Jul 4;308(1):43-9.
3. Zhang J, Delzell E, Xie F, Baddley JW, Spettell C, McMahan RM, Fernandes J, Chen L, Winthrop K, Curtis JR. The use, safety, and effectiveness of herpes zoster vaccination in individuals with inflammatory and autoimmune diseases: a longitudinal observational study. Arthritis Res Ther. 2011;13(5):R174.
4. Wasan SK, Zullow S, Berg A, Cheifetz AS, Ganley-Leal L, Farraye FA. Herpes Zoster Vaccine Response in Inflammatory Bowel Disease Patients on Low-dose Immunosuppression. Inflamm Bowel Dis. 2016 Jun;22(6):1391-6.
5. Khan N, Shah Y, Trivedi C, Lewis JD. Safety of herpes zoster vaccination among inflammatory bowel disease patients being treated with anti-TNF medications. Aliment Pharmacol Ther. 2017 Oct;46(7):668-672.
6. Lal H, Cunningham AL, Godeaux O, Chlibek R, Diez-Domingo J, Hwang SJ, Levin MJ, McElhaney JE, Poder A, Puig-Barberà J, Vesikari T, Watanabe D, Weckx L, Zahaf T, Heineman TC; ZOE-50 Study Group. Efficacy of an adjuvanted herpes zoster subunit vaccine in older adults. N Engl J Med. 2015 May 28;372(22):2087-96.
7. Cunningham AL, Lal H, Kovac M, Chlibek R, Hwang SJ, Díez-Domingo J, Godeaux O, Levin MJ, McElhaney JE, Puig-Barberà J, Vanden Abeele C, Vesikari T, Watanabe D, Zahaf T, Ahonen A, Athan E, Barba-Gomez JF, Campora L, de Looze F, Downey HJ, Ghesquiere W, Gorfinkel I, Korhonen T, Leung E, McNeil SA, Oostvogels L, Rombo L, Smetana J, Weckx L, Yeo W, Heineman TC; ZOE-70 Study Group. Efficacy of the Herpes Zoster Subunit Vaccine in Adults 70 Years of Age or Older. N Engl J Med. 2016 Sep 15;375(11):1019-32.

Risk of Bias Table – Adults

**Case Control Studies**						
**Study**	**Cases and controls similar for risk of exposure (or adjusted adequately for confounders)**	**Methods to determine exposure valid and similar for cases and controls**	**Methods to ascertain outcome of interest valid and similar for cases and controls**	**Missing data**	**Other bias**	**Comments**
Khan 2019 US	Adjusted for age at index date, intensity of prednisone use, oral prednisone use within 30 days before index date, a proxy for flare (hospitalization, iv steroid, CT, stool Clostridium difficile toxin testing, lower	Receipt of zoster vaccine outside the VA was not accounted for, but the chances of this creating a bias is less likely because previous reports have indicated that veterans have	Data were from administrative claim data, but validated by performing manual review of records of 200 randomly selected patients who had herpes zoster infection and IBD diagnostic codes (diagnosis confirmed in	OK	<u>Selection bias:</u> zoster vaccine may be selectively given to healthier patients. This may have led to over-estimation of the protective effect	• Retrospective case control study among patients in the VA system (older male population) from 2000-2016<br>• 32,813 IBD patients who had not received the **HZ vaccine (LZV, Zostavax®)** by age 60 vs. 7170 (17.9% of cohort) IBD patients who had received the vaccine<br>• **HZ vaccination was associated with a significantly lower risk of herpes zoster**

	bowel endoscopy), geographic location, IBD diagnosis, health care use, race, gender, baseline comorbid conditions.	very good adherence to the VA pharmacy, seeking care at the VA and vaccinations are covered in the VA.	91% and 94.5% respectively).		of the vaccine and underestimation of adverse effects.	**infection compared with no vaccination (AHR 0.54, 95% CI 0.44-0.68).**<br>• HZ infection rates: 6.97 per 1000 person-years in unvaccinated vs. 4.09 per 1000 person-years vaccinated<br>• Increased risk of herpes zoster infection with thiopurines, prednisone (cumulative and within 30 days), age, disease flare, and number of healthcare utilization visits<br>• Among patients vaccinated while on thiopurines compared with patients not vaccinated while on thiopurines, no significant risk reduction of herpes zoster infection (AHR 0.63, 95% CI 0.30-1.33). Results also similar for patients vaccinated > 60 days before thiopurines compared with patients not vaccinated and exposed to thiopurines (AHR 0.57, 95% CI 0.18-1.76)<br>• Too few patients and events for patients who were vaccinated while on anti-TNF alone or in combination with thiopurine, or on vedolizumab<br>• Cannot evaluate efficacy and safety of HZ vaccine in younger IBD patients because only 227 patients (age < 60) were vaccinated
Zhang 2012 US	Adjusted for gender, race, immune-mediated disease, time-varying concurrent medications, and health care utilization (hospitalization and	Administration of zoster vaccine was identified by Current Procedural Terminology code, or a combination of the National	Positive predictive value of the herpes zoster diagnosis code alone to identify incident case of herpes zoster (using medical review as a gold standard) has been shown to range	Actual vaccine administration dates were unknown for 59% of patients which resulted in	Selection bias: zoster vaccine may be selectively given to healthier patients. This may have led to over-estimation	• Retrospective cohort study among Medicare beneficiaries diagnosed with immune-mediated diseases including rheumatoid arthritis (292,169), psoriatic arthritis, psoriasis (11,030), ankylosing spondylitis (4,026), or **IBD (66,751**) aged ≥ 60 from 2006-2009<br>• Median duration of follow-up 2.0 (0.8-

	physician visits). Did not adjust for disease severity or flare.	Drug Code for the zoster vaccine and Health Care Common Procedure Code in the subsequent 7 days (representing its administration)	between 80% and 100%.	exclusion of these patients from safety (but not the effectiveness) analyses.	of the protective effect of the vaccine and under-estimation of adverse effects.	3.0) years<br>• 4% (18,683) received the **herpes zoster vaccine (LZV, Zostavax®)**<br>• <u>**Safety**</u>**: Among 7780 vaccinated patients, IR of HZ within 42 days (7.8 cases per 1000 person years, 95% CI 3.7-16.5). Among 633 patients exposed to biologics (551 anti-TNF), no cases of varicella or HZ occurred within 42 days following vaccination (95% CI 0-5.4 per 1000 person years among anti-TNF and 0-4.7 per 1000 among biologic users).**<br>• <u>**Effectiveness**</u>**: Reduced risk of HZ infection after 42 days with vaccination (AHR 0.61, 95% CI 0.52-0.71). Among the vaccinated, lower rates of HZ with vaccination (6.7 cases per 1000 person years, 95% CI 5.7-7.9) vs. unvaccinated (11.6 cases per 1000 person years, 95% CI 11.4-11.9). Lower rates in all subgroups of patients categorized by medication exposure.** See Table
Zhang 2011 US	Applied age- and sex-specific incidence rates of the unvaccinated to age and sex-specific vaccinated person-time to derive the expected number of herpes zoster cases among the vaccinated and calculated the standardized incidence-rate ratio (SIR) as the observed divided by the expected number of cases. Did not adjust for disease severity or	Administration of zoster vaccine was identified by Current Procedural Terminology code.	Cases of herpes zoster were identified by the first herpes zoster claim that was preceded or followed by a prescription for anti-viral medications within 30 days of the claim date.	OK	<u>Selection bias:</u> zoster vaccine may be selectively given to healthier patients. This may have led to over-estimation of the protective effect of the vaccine and under-estimation of adverse effects. Younger and healthier	• Retrospective cohort study among a private insurance health plan diagnosed with immune-mediated diseases including rheumatoid arthritis (19,326), psoriatic arthritis (867), psoriasis (10,712), ankylosing spondylitis (633), or IBD (8,639) aged $\geq$ 50 from 2006-2009<br>• 1.2% (551) received the **HZ vaccine (LZV, Zostavax®)**<br>• <u>**Effectiveness:**</u> **similar incidence rates of HZ in vaccinated (crude IR 9.97 per 1000 person-years) vs. unvaccinated patients (crude IR 8.61 per 1000 person-years) (SIR 0.99, CI 0.29-3.43)**

	flare, immune-mediated disease, time-varying concurrent medications, and health care utilization (hospitalization and physician visits).				patients were more likely to be vaccinated.	• **Safety: Only 1/551 out of all vaccinated patients developed HZ within 42 days of vaccination**. 6% (47) received vaccine while using anti-TNF, no HZ within 1 month

**Before-After (Pre-Post) Studies**									
			**If no concurrent comparator group was used**						
**Study**	**Was there a concurrent comparator group that did not receive the intervention**	**If a concurrent comparator group was used, was it similar to the intervention group (or adequately adjusted) for prognostic factors**	**If each participant served as his/her own control (assessed before vs. after the intervention), are there compelling arguments that the outcome was not influenced by historic events / underlying secular trends**	**If two different consecutive cohorts of participants were assessed (before vs. after implementation of the intervention), are there (a) compelling arguments that the outcome was not influenced by historic events / underlying secular trends and (b) evidence that the two groups were similar (or**	**Outcome detection methods valid and similar among compared groups / periods**	**Incomplete outcome data assessed**	**Selective outcome reporting**	**Other bias**	**Comments**

				**adequately adjusted) for prognostic factors**					
Wasan 2016 US	No – but this does not affect the risk of bias as the only explanation for increase in herpes zoster immunoglobulin G level is the vaccine (no other confounding factors)	No – but this does not affect the risk of bias as the only explanation for increase in herpes zoster immunoglobulin G level is the vaccine (no other confounding factors)	OK	OK	OK	OK	OK	Should have a healthy control group to compare vaccination response. Possible both groups had blunted response to vaccination compared to healthy control.	• Prospective cohort study of 39 IBD patients (14 on low dose immunomodulators, 25 on 5ASA or no therapy)<br>• **HZ vaccine (LZV, Zostavax[illegible])** was administered<br>• Immune responses were assessed at baseline and 2 weeks post vaccination<br>• HZ immunoglobulin G increased significantly in both groups, but response was lower in the immunosuppressed group. Unclear if this blunted response is clinically relevant and would afford protection<br>• **<u>Safety:</u> no serious adverse events in both groups within 1 year after vaccination**

Cohort studies							
**Study**	**Valid methods to ascertain exposure**	**Prognostic factors (other than exposure of interest) similar among cohorts – or cohorts were adjusted adequately for confounders**	**Demonstration that outcome of interest was not present at the start of the study**	**Outcome detection methods valid and similar among cohorts**	**Follow-up complete and similar among cohorts**	**Free of other bias**	**Comments**
Khan 2017<br>US	Administration of herpes zoster vaccine was identified by Current Procedural Terminology (CPT) code and validated by chart reviews.	Selection bias. Patients who were given herpes zoster vaccine may be systematically different from those who were not given the vaccine in terms of prognostic factors.	OK<br>Chart reviews	OK<br>Chart reviews	OK<br>Chart reviews, but may have missed patients who received care outside the VA. Median number of follow up visits in the 42 days post vaccination were 2.	OK	• Retrospective cohort study among patients in the VA system (older male population) from 2000-2016<br>• 59 IBD patients (median age 64.9 years, 95% had Charlson Comorbidity index ≥2) on anti-TNF (infliximab, adalimumab, Golimumab, Certolizumab) when they were given HZ vaccine (**LZV, Zostavax**), 20% were also on thiopurine<br>• **Safety: No case of HZ infection was found within 0-42 days of vaccination**

Evidence Profile Table – Adults

Herpes Zoster Vaccine (HZV) in the IBD Population

Certainty Assessment								Summary of Findings				Comments
								No of patients (ITT)		Effect		
Studies	Risk of bias	Inconsistency	Indirectness	Imprecision	Other considerations	Certainty of Evidence	Overall certainty of evidence	HZV vaccine	Control	Relative (95%CI)	Absolute (95%CI)	
**VPI (Herpes Zoster) - CRITICAL**												
1 RCT[1]<br>**Immunocom-petent adults aged > 50**<br>*Adapted from CDC evidence profile table*	Not serious	Not serious	Not serious (patients not on IS)<br>Serious[a] (patients on IS)	Not serious	None	⊕⊕⊕⊕ HIGH (patients not on IS)<br>⊕⊕⊕⊖ MODERATE (patients on IS)		6/7698 (0.078%)	210/7713 (2.72%)	Vaccine efficacy: 97.2% (93.7-99.0)		RZV vaccine
1 Case-control study[2]<br>**IBD populations**	Serious[b]	Not serious	Very serious[c]	Not serious		⊕⊖⊖⊖ VERY LOW	⊕⊕⊕⊖ MODERATE Age > 50<br>⊕⊕⊖⊖ LOW Age < 50[r]	92/7,170 (1.3%)	1,776/32,813 (5.4%)	**HR 0.54 (0.44 to 0.68)**	25 fewer per 1,000 (from 30 fewer to 17 fewer)	See **Summary of case-control studies assessing the efficacy of LZV in IBD patients**
1 Case-control study[3]<br>**immune-mediated diseases including IBD**	Serious[d]	Not serious	Very serious[e]	Not serious	Publication bias cannot be assessed (< 10 studies)	⊕⊖⊖⊖ VERY LOW		138/7,780 (1.8%)	9,960/444,858 (2.2%)	**HR 0.61 (0.52 to 0.71)**	9 fewer per 1000 (from 11 fewer to 6 fewer)	See **Summary of case-control studies assessing the efficacy of LZV in patients with immune-mediated diseases**
1 Case-control study[4]<br>**immune-mediated diseases including IBD**	Serious[f]	Not serious	Very serious[g]	Serious[h]		⊕⊖⊖⊖ VERY LOW		5/551 (0.9%)	756/43,564 (1.7%)	SIR 0.9 (0.29-3.43)	0 fewer per 1000 (from 12 fewer to 42 more)	
**Serious adverse effects - CRITICAL**												
1 RCT[1,7]	Not	Not serious	Serious[i]	Not serious	None	⊕⊕⊕⊖		No differences in serious adverse events between vaccinated				RZV vaccine

**Immunocom-petent adults aged > 50**<br><br>*Adapted from CDC evidence profile table*	serious					MODERATE		and placebo groups.<br><br>No serious adverse events related to vaccination found.	
1<br>Cohort study[3]<br><br>**immune-mediated diseases including IBD**	Serious[d]	Not serious	Very serious[j]	Not serious	Publication bias cannot be assessed (< 10 studies)	⊕⊖⊖⊖<br>VERY LOW		11/7780 (0.14%) cases of HZ within 42 days after vaccination<br>Overall: IR 7.8/1000 person-years (3.7-16.5)<br>Exposed to biologics: IR 0/1000 person-years (0-4.7)<br>Exposed to anti-TNF: IR 0/1000 person-years (0-5.4)<br>0 case of hospitalized meningitis or encephalitis<br>1 case of primary varicella	LZV vacccine
1<br>Cohort study[4]<br><br>**immune-mediated diseases including IBD**	Serious[f]	Not serious	Very serious[k]	Serious[l]		⊕⊖⊖⊖<br>VERY LOW		1/551 (0.18%) cases of HZ within 42 days after vaccination, no hospitalization.<br>Exposed to biologics: 0/47<br>Exposed to methotrexate: 0/79<br>Exposed to steroids: 0/81	LZV vacccine
1<br>Cohort study[5]<br><br>**IBD populations**	Serious[m]	Not serious	Very serious[n]	Serious[l]		⊕⊖⊖⊖<br>VERY LOW		0/59 cases of HZ within 42 days after vaccination<br>All patients were on anti-TNF. 20% were also on thiopurine.	LZV vacccine
1<br>Before-after study[6]<br><br>**IBD populatons**	Not serious	Not serious	Serious[o]	Serious[p]		⊕⊖⊖⊖<br>VERY LOW		no serious adverse events within 1 year after vaccination	LZV vacccine
**Immunogenicity (HZ immunoglobulin G level at baseline and 2 weeks post HZV) - IMPORTANT**									
1<br>Before-after study[6]<br><br>**IBD populations**	Not serious	Not serious	Very serious[q]	Not serious	Publication bias cannot be assessed (< 10 studies)	⊕⊖⊖⊖<br>VERY LOW		HZ immunoglobulin G increased significantly 2 weeks post vaccination in both groups (low dose immunomodulators, 5ASA or no therapy), but response was lower in the immunosuppressed group. Unclear if this blunted response is clinically relevant and would afford protection	LZV vacccine

HZV – herpes zoster vaccine
IS - immunosuppressive medications
LZV – live attenuated HZV

Footnotes:

a. Not downgraded for indirectness for IBD patients not on immunosuppressive medications. Downgraded for indirectness for IBD patients on immunosuppressive medications as observational studies suggested herpes zoster vaccine may be less immunogenic and effective among IBD patients on immunosuppressive medications.
b. Downgraded for study limitations. Selection bias: HZV may be selectively given to healthier patients. Only 18% of patients received HZV. This may have led to over-estimation of the protective effect of the vaccine and underestimation of adverse effects.
c. Downgraded for indirectness related to patient population, intervention, and outcome. The study included patients in the VA system (predominantly older, white males) who had not received the HZV by an age of 60 years. Only 227 patients younger than age 60 were vaccinated. Results may not be generalizable to the whole IBD population. Also, the PICO question pertains to recombinant zoster vaccine (not live attenuated zoster vaccine). The study reported HZ as the main outcome, but did not report on more severe complications related to HZ (e.g. post-hepatic neuralgia, herpes zoster ophthalmicus, herpes zoster oticus, necrotizing retinitis, cranial and peripheral nerve palsies, myelopathy, meningoencephalitis, cerebellitis, and visceral involvement including pneumonitis, hepatitis, and acute retinal necrosis), which are more important to patients than a self-limited rash (shingles).
d. Downgraded for study limitations. Selection bias: HZV may be selectively given to healthier patients. Only 4% of patients received HZV. This may have led to over-estimation of the protective effect of the vaccine and underestimation of adverse effects. Actual vaccine administration dates were unknown for 59% of patients which resulted in exclusion of these patients from safety (but not the effectiveness) analyses.
e. Downgraded for indirectness related to patient population, intervention, and outcome. The study included patients with selected immune-mediated diseases including rheumatoid arthritis (292,169), psoriatic arthritis (11,030), psoriasis (89,565), ankylosing spondylitis (4,026), or IBD (66,751) aged > 60. Results were reported for the entire cohort (no subgroup data for IBD patients). Also, the PICO question pertains to recombinant zoster vaccine (not live attenuated zoster vaccine). The study reported HZ as the main outcome, but did not report on more severe complications related to HZ (e.g. post-hepatic neuralgia, herpes zoster ophthalmicus, herpes zoster oticus, necrotizing retinitis, cranial and peripheral nerve palsies, myelopathy, meningoencephalitis, cerebellitis, and visceral involvement including pneumonitis, hepatitis, and acute retinal necrosis), which are more important to patients than a self-limited rash (shingles).
f. Downgraded for study limitations. Selection bias: HZV may be selectively given to healthier patients. Only 1.2% of patients received HZV. This may have led to over-estimation of the protective effect of the vaccine and underestimation of adverse effects. Residual confounding factors: did not adjust for disease severity or flare and health care utilization (hospitalization and physician visits).
g. Downgraded for indirectness related to patient population, intervention, and outcome. The study included patients with selected immune-mediated diseases including rheumatoid arthritis (19,326), psoriatic arthritis (867), psoriasis (10,712), ankylosing spondylitis (633), or IBD (8639) aged $\geq$ 50. Results were reported for the entire cohort (no subgroup data for IBD patients). Also, the PICO question pertains to recombinant zoster vaccine (not live attenuated zoster vaccine). The study reported HZ as the main outcome, but did not report on more severe complications related to HZ (e.g. post-hepatic neuralgia, herpes zoster ophthalmicus, herpes zoster oticus, necrotizing retinitis, cranial and peripheral nerve palsies, myelopathy, meningoencephalitis, cerebellitis, and visceral involvement including pneumonitis, hepatitis, and acute retinal necrosis), which are more important to patients than a self-limited rash (shingles).
h. Downgraded for imprecision. Wide confidence interval consistent with either significant decrease or increase in the risk of HZ with vaccination.
i. Downgraded for indirectness. Very small number of IBD patients were assessed for safety outcomes, although no serious adverse events were noted. Only live attenuated zoster vaccines (not recombinant zoster vaccine) were evaluated in IBD patients.
j. Downgraded for indirectness related to patient population, intervention, and outcome. The study included patients with selected immune-mediated diseases including rheumatoid arthritis (292,169), psoriatic arthritis, psoriasis (11,030), ankylosing spondylitis (4,026), or IBD (66,751) aged > 60.

Results were reported for the entire cohort (no subgroup data for IBD patients). Also, the PICO question pertains to recombinant zoster vaccine (not live attenuated zoster vaccine). Adverse effects outcome included only HZ incidence rate within 42 days following vaccination.

k. Downgraded for indirectness related to patient population, intervention, and outcome. The study included patients with selected immune-mediated diseases including rheumatoid arthritis (19,326), psoriatic arthritis (867), psoriasis (10,712), ankylosing spondylitis (633), or IBD (8639) aged $\geq$ 50. Results were reported for the entire cohort (no subgroup data for IBD patients). Also, the PICO question pertains to recombinant zoster vaccine (not live attenuated zoster vaccine). Adverse effects outcome included only HZ incidence rate within 42 days following vaccination.
l. Downgraded for imprecision given small sample size and low event rates for the vaccinated group.
m. Downgraded for study limitation. Selection bias: patients who were given HZV may be systematically different from those who were not given the vaccine in terms of prognostic factors.
n. Downgraded for indirectness related to patient population, intervention, and outcome. The study included patients in the VA system (predominantly older, white males) who were treated with anti-TNF. Also, the PICO question pertains to recombinant zoster vaccine (not live attenuated zoster vaccine). Adverse effects outcome included only HZ incidence rate within 42 days following vaccination.
o. Downgraded for indirectness related to patient population, intervention, and outcome. The study included patients who were on low-dose immunomodulators, 5-aminosalicylic acid or no IBD therapy. Results may not be generalizable to the whole IBD population. Also, the PICO question pertains to recombinant zoster vaccine (not live attenuated zoster vaccine).
p. Downgraded for imprecision due to small sample size (39)
q. Downgraded for indirectness related to patient population, intervention, and outcome. The study included patients who were on low-dose immunomodulators, 5-aminosalicylic acid or no IBD therapy. Results may not be generalizable to the whole IBD population. Also, the PICO question pertains to recombinant zoster vaccine (not live attenuated zoster vaccine). Surrogate outcome was used.
r. Downgraded for indirectness as most data was from adults age > 50 (lower absolute risks of HZ in adults age < 50, and no data on long term protection).

Summary of case-control studies assessing the efficacy of live attenuated herpes zoster vaccine (HZV) in IBD patients

Study	IBD patients who had received the HZV (Vaccinated)	IBD patients who had not received the HZV (Unvaccinated)	Incidence rates of HZ in IBD patients who had received the HZV (Vaccinated)	Incidence rates of HZ in IBD patients who had not received the HZV (Unvaccinated)	Adjusted Incidence rate ratios (IRR) / Hazards Ratio (HR) for HZ (95% CI)
Khan 2019[2]	22,486.05 person-years	254,889.2 person-years	4.09 / 1000 person-years	6.97 / 1000 person-years	Lower risk of HZ with vaccination: AHR 0.54 (0.44-0.68)
Zhang 2012[3]	1891.0 person-years	115,372.2 person-years	-	-	Lower risk of HZ with vaccination. No difference in risk of HZ with vaccination in IBD patients compared to patients with rheumatoid arthritis (reference): AHR 1.03 (0.97-1.10)

Summary of case-control studies assessing the efficacy of live attenuated herpes zoster vaccine (HZV) in patients with immune-mediated diseases

Study	Number of IBD patients (%)	Patients with immune-mediated diseases who had received the HZV (Vaccinated)	Patients with immune-mediated diseases who had not received the HZV (Unvaccinated)	Incidence rates of HZ in patients with immune-mediated diseases who had received the HZV (Vaccinated)	Incidence rates of HZ in patients with immune-mediated diseases who had not received the HZV (Unvaccinated)	Adjusted Incidence rate ratios (IRR) / Hazards Ratio (HR) for HZ (95% CI)
Zhang 2012[3]	66,751 (14.4%)	52,436 person-years	855,226 person-years	6.7 / 1000 person-years	11.6 / 1000 person-years	Lower risk of HZ with vaccination:<br>AHR 0.61 (0.52-0.71)<br><br>Lower IR (per 1000 person-years) of HZ with vaccination in all subgroups of patients categorized by medication exposure.<br>Biologics: IR 8.5 (5.1-14.4) vs. 16.0 (15.2-16.8)<br>Anti-TNF: IR 8.5 (4.8-15.0) vs. 15.9 (15.1-16.8)<br>DMARDs: 7.0 (4.7-10.3) vs. 13.6 (13.1-14.2)<br>Steroids alone: 10.3 (6.7-15.8) vs. 17.2 (16.5-17.9)
Zhang 2011[4]	8639 (19.6%)	551 patients	43,564 patients	9.97 / 1000 person-years	8.61 / 1000 person-years	No difference in risk of HZ with vaccination vs. no vaccination<br>SIR 0.99 (0.29-3.43)

DMARDS: disease modifying antirheumatic drugs
IR: incidence rates

References:

1. Lal H, Cunningham AL, Godeaux O, Chlibek R, Diez-Domingo J, Hwang SJ, Levin MJ, McElhaney JE, Poder A, Puig-Barberà J, Vesikari T, Watanabe D, Weckx L, Zahaf T, Heineman TC; ZOE-50 Study Group. Efficacy of an adjuvanted herpes zoster subunit vaccine in older adults. N Engl J Med. 2015 May 28;372(22):2087-96

2. Khan N, Trivedi C, Kavani H, Medvedeva E, Lewis J, Yang YX. Efficacy of Live Attenuated Herpes Zoster Vaccine in Patients With Inflammatory Bowel Diseases. Clin Gastroenterol Hepatol. 2019 Jun;17(7):1341-1347.
3. Zhang J, Xie F, Delzell E, Chen L, Winthrop KL, Lewis JD, Saag KG, Baddley JW, Curtis JR. Association between vaccination for herpes zoster and risk of herpes zoster infection among older patients with selected immune-mediated diseases. JAMA. 2012 Jul 4;308(1):43-9.
4. Zhang J, Delzell E, Xie F, Baddley JW, Spettell C, McMahan RM, Fernandes J, Chen L, Winthrop K, Curtis JR. The use, safety, and effectiveness of herpes zoster vaccination in individuals with inflammatory and autoimmune diseases: a longitudinal observational study. Arthritis Res Ther. 2011;13(5):R174.
5. Khan N, Shah Y, Trivedi C, Lewis JD. Safety of herpes zoster vaccination among inflammatory bowel disease patients being treated with anti-TNF medications. Aliment Pharmacol Ther. 2017 Oct;46(7):668-672.
6. Wasan SK, Zullow S, Berg A, Cheifetz AS, Ganley-Leal L, Farraye FA. Herpes Zoster Vaccine Response in Inflammatory Bowel Disease Patients on Low-dose Immunosuppression. Inflamm Bowel Dis. 2016 Jun;22(6):1391-6.
7. Cunningham AL, Lal H, Kovac M, Chlibek R, Hwang SJ, Díez-Domingo J, Godeaux O, Levin MJ, McElhaney JE, Puig-Barberà J, Vanden Abeele C, Vesikari T, Watanabe D, Zahaf T, Ahonen A, Athan E, Barba-Gomez JF, Campora L, de Looze F, Downey HJ, Ghesquiere W, Gorfinkel I, Korhonen T, Leung E, McNeil SA, Oostvogels L, Rombo L, Smetana J, Weckx L, Yeo W, Heineman TC; ZOE-70 Study Group. Efficacy of the Herpes Zoster Subunit Vaccine in Adults 70 Years of Age or Older. N Engl J Med. 2016 Sep 15;375(11):1019-32

Evidence to Decision Table – Adults (50 years of age and older)

**PICO 10A**	**In adult patients with IBD (50 years of age and older), should vaccination vs. no vaccination against herpes zoster (recombinant zoster vaccine) be given?**
**Population**	Adult patients with IBD (50 years of age and older)
**Intervention**	Vaccination against herpes zoster (RZV, Shingrix®)
**Comparator**	No vaccination against herpes zoster
**Outcome**	Mortality, VPI (herpes zoster infection and complications), SAEs, Immunogenicity

**Judgement**	**Research evidence**	**Additional considerations**

**Desirable Effects**	**How substantial are the desirable anticipated effects?**<br><br>○ Trivial<br>○ Small<br>○ Moderate<br>○ Large<br><br>○ Varies<br>○ Don't know	See Evidence Profile Tables.<br><br>**Risk of HZ in adult IBD patients**<br>Nine case-control studies (considered as prognostic studies) addressed this PICO question.[1-9] All nine studies found an increased risk of herpes zoster (HZ) in IBD patients compared to the general population (1.2-1.8 times). The GRADE rating started at high as these studies provided prognostic evidence about the likelihood of HZ in patients with IBD when compared to non-IBD controls. The rating was eventually downgraded to **low** due to study limitations (detection bias, residual confounding factors) and indirectness (HZ as main outcome). In particular, IBD patients may have more frequent outpatient visits and/or hospitalization than non-IBD patients. This may lead to over-estimation of the risk of HZ in IBD patients. As well, all studies reported HZ as the main outcome, but did not report on more severe complications related to HZ (e.g. post-hepatic neuralgia, herpes zoster ophthalmicus, herpes zoster oticus, necrotizing retinitis, cranial and peripheral nerve palsies, myelopathy, meningoencephalitis, cerebellitis, and visceral involvement including pneumonitis, hepatitis, and acute retinal necrosis), which are more important to patients than a self-limited rash (shingles). **In summary, there is low certainty evidence that adult IBD patients have an increased risk of HZ infection compared to non-IBD patients.**<br><br>Six case-control studies (considered as prognostic studies) provided data on the risks of HZ stratified by age.[2-5,7,9] The only low risk of bias study (Khan 2018) suggested that the incidence of HZ among the younger IBD patients (age < 50) exceeded that of older control patients (age > 50) for whom the recombinant zoster vaccine (RZV, Shingrix?) is recommended by ACIP as the preferred vaccine for prevention of HZ and related complications. The rating of evidence for this subgroup analysis was further downgraded to very low due to imprecision since only a small number of IBD patients age < 50 had HZ. **In summary, there is low certainty evidence that adult IBD patients older than age 50 have an increased risk of HZ infection compared to non-IBD patients older than age 50. There is very low certainty evidence that adult IBD patients younger than age 50 have an increased risk of HZ infection compared to non-IBD patients older than age 50.**<br><br>Five case-control studies and one systematic review of RCTs and observational studies (considered as prognostic studies) provided data on the risks of HZ stratified by medication groups.[2-3,7-9,13] The use of steroids, thiopurines, anti-TNF, and combination therapy were associated with increased risks of HZ among IBD patients compared to IBD patients on no treatment / 5ASA or to the general population. The rating of evidence for this subgroup analysis was further downgraded to **very low** due to imprecision of effect estimates for anti-TNF and thiopurines with wide CIs, inconsistency with some studies suggesting no increased risks for HZ with anti-TNF and thiopurines, and residual confounding with no adjustment for disease flare or severity in most studies. **In summary, there is very low certainty evidence that steroids, combination therapy (steroids + thiopurines or anti-TNF, steroids + thiopurines + anti-TNF, thiopurines + anti-TNF), thiopurines alone, and anti-TNF alone were associated with increased risks of HZ among IBD patients.**	
**Undesirable Effects**	**How substantial are the undesirable anticipated effects?**<br><br>○ Large<br>○ Moderate<br>○ Small<br>○ Trivial<br><br>○ Varies<br>○ Don't know		

		Three single-arm RCTs (considered as prognostic studies) provided data on the incidence of HZ associated with the use of vedolizumab, ustekinumab, and tofacinib.[10-12] **There is <u>very low</u> certainty evidence that tofacinib is associated with an increased incidence of HZ in IBD patients, but vedolizumab and ustekinumab are not.**<br><br>**Effectiveness and safety of HZV in adult IBD patients**<br>There was no RCT comparing recombinant (or live attenuated) herpes zoster vaccine (HZV) with placebo or no treatment in adult patients with IBD to address this PICO question.<br><br>One case-control study in IBD patients, two case-control studies in patients with selected immune-mediated diseases including IBD, and a before-and-after study in IBD patients addressed this PICO question.[14-17] The two larger case-control studies[14,15] that included a larger number of vaccinated patients showed a significant reduction in the risk of HZ (39-46%) following vaccination with the live-attenuated zoster vaccine (LZV, Zostavax®). In a large case-control study, no significant risk reduction of herpes zoster infection was seen among patients vaccinated while on thiopurines compared with patients not vaccinated while on thiopurines (AHR 0.63, 95% CI 0.30-1.33).[14] There were too few patients who were vaccinated while on anti-TNF alone or in combination with a thiopurine to assess the association of their use with effectiveness of the vaccine.[14] The before-and-after study showed that IBD patients can mount an immune response to the live-attenuated zoster vaccine (LZV, Zostavax®) with a significant increase in HZ immunoglobulin G 2 weeks post vaccination, but the response was lower in patiens on low dose immunomodulators (methotrexate $\leq$ 0.4mg/kg/week, azathioprine $\leq$ 3.0mg/kg/d, 6MP $\leq$ 1.5mg/kg/d).[17] It is uncertain if this blunted response is clinically relevant and still would afford seroprotection. No serious adverse events were reported with the live attenuated herpes zoster vaccine.[17] The GRADE rating of these studies started as low due to their observational designs. The rating was downgraded to **<u>very low</u>** due to study limitations (selection bias, residual confounding) and indirectness (patient population, intervention, outcome). In particular, HZV may be selectively given to healthier patients (healthy vaccinee effect). This may have led to over-estimation of the protective effect of the vaccine and underestimation of adverse effects. As well, the PICO question pertains to recombinant zoster vaccine (not live attenuated zoster vaccine which was assessed in these studies). The studies reported HZ as the main outcome, but did not report on more severe complications related to HZ (e.g. post-hepatic neuralgia, herpes zoster ophthalmicus, herpes zoster oticus, necrotizing retinitis, cranial and peripheral nerve palsies, myelopathy, meningoencephalitis, cerebellitis, and visceral involvement including pneumonitis, hepatitis, and acute retinal necrosis), which are more important to patients than a self-limited rash (shingles). The incidence of HZ within 42 days following vaccination with live attenuated zoster vaccine in IBD patients was very low.<br><br>The CDC ACIP recommends herpes zoster recombinant vaccine in immunocompetent adults aged 50 years and older for the prevention of HZ and related complications based on **<u>high level</u>** of evidence for both safety and effectiveness. Since there were studies on clinical effectiveness, safety, and immunogenicity on live attenuated zoster	

		vaccines in age-specific IBD populations that supported the findings in the general populations, the evidence was not downgraded for indirectness related to patient population (general population vs. IBD population). As per CDC, recombinant zoster vaccine is preferred over live attenuated vaccine due to higher effectiveness. Therefore, the evidence is also not downgraded for indirectness related to intervention. The evidence of adult IBD patients aged 50 years and older is therefore anchored to the general population. However, it is possible that herpes zoster vaccine may not be as effective in IBD patients on immunosuppressive medications. As well, only a small number of IBD patients on different types of immunosuppressive medications were included in these studies. Therefore, the evidence was downgraded 1 level to moderate for adult IBD patients on immunosuppressive medications. **Overall, there is high certainty evidence that herpes zoster vaccine is effective in adult IBD patients aged 50 years and older not on immunosuppressive medications. There is moderate certainty evidence that herpes zoster is effective in adult IBD patients aged 50 years and older on immunosuppressive medications. As there is serious imprecision with the estimate of serious adverse events related to use of zoster vaccine in IBD patients and all included studies assessed live attenuated vaccines (not recombinant vaccines), the evidence was downgraded to moderate for safety.**[15-18]<br><br>**Overall, there is moderate certainty evidence that recombinant zoster vaccine is safe and effective in adult IBD patients aged 50 and older on or not on immunosuppressives.**<br><br>As there were very few **younger IBD patients (age < 50)** who received the herpes zoster vaccine in the included studies, the benefits vs. risks of the recombinant zoster vaccine in this patient population are very uncertain. As well, no long-term studies on the duration of vaccine protection have been performed for patients younger than 50 years old. It remains unclear whether adults receiving the vaccine before age 50 will continue to be protected as they age. **If we extrapolate the evidence from older IBD patients (age > 50), the evidence would have to be downgraded to low for both safety and effectiveness.**	
Certainty of evidence	**What is the overall certainty of the evidence of effects?**<br><br>○ Very low<br>○ Low (for patients age < 50 no or not on IS)<br>○ Moderate (for patients age ≥ 50 on or not on IS)<br>○ High<br><br>○ No included studies		
Values and Preferences	**Is there important uncertainty about or variability in how much people value the main outcomes?**<br><br>○ Important uncertainty or variability	A survey was conducted to evaluate willingness to pay (WTP) for a quality-adjusted life-year (QALY) based on community member and patient preferences for temporary health states associated with herpes zoster.[19] The study showed that patients and community members gave mean WTP per QALY values that **varied significantly** based	

	○ Possibly important uncertainty or variability<br>○ Probably no important uncertainty or variability<br>○ No important uncertainty or variability	on age, sex, socioeconomic status, experience with shingles and duration of the health state evaluated. The WTP per QALY ranged from a trimmed mean of $US 26,000 to US 45,000 (year 2005 values). In multivariate analyses, the mean WTP per QALY was higher among respondents who were younger, male or had higher educational or income levels. After adjusting for these demographic variables, patients who had experienced shingles gave responses with the highest WTP per QALY values. Patients who had experienced PHN gave the lowest values, and community members gave values intermediate to the shingles and PHN groups. In multivariate models that evaluated the effects of pain and duration of the hypothetical zoster scenario, lower duration was associated with higher WTP per QALY. This effect appeared to be due to people increasing the amounts of time they would be willing to trade as duration increased, without proportional increases in the amounts of money they would be willing to pay. **The high variability in responses underscored the fact that preferences at the individual level may vary substantially.**<br><br>A discrete choice experiment was conducted to determine the relative importance of vaccine and disease specific characteristics and acceptance for Dutch older adults (age > 50).[20] The results suggest that older adults are most likely to accept pneumococcal vaccination of the 4 vaccines evaluated (pneumococcal, herpes zoster, pertussis, influenza). Potential vaccination rates of older adults were estimated at 58.1% for herpes zoster.	
**Balance of effects**	**Does the balance between desirable and undesirable effects favor the intervention or the comparison?**<br><br>○ Favors the comparison<br>○ Probably favors the comparison<br>○ Does not favor either the intervention or the comparison<br>○ Probably favors the intervention<br>○ Favors the intervention<br><br>○ Varies<br>○ Don't know		
**Resources required**	**How large are the resource requirements (costs)?**<br><br>○ Large costs<br>○ Moderate costs<br>○ Negligible costs and savings<br>○ Moderate savings<br>○ Large savings	CDC vaccine price list last reviewed/updated: July 1, 2019<br><br>**Brandname** | **CDC cost/dose** | **Private sector cost/dose**<br><br>Shingrix is given as a 2-dose vaccine.	

	○ Varies<br>○ Don't know	Shingrix® | $102.90 | $144.20	
Certainty of Evidence of Required Resources	**What is the certainty of the evidence of resource requirements (costs)?**<br><br>○ Very low<br>○ Low<br>○ Moderate<br>○ High<br><br>○ No included studies	The costs of delivering routine immunization services may vary widely across countries and different health system settings. See Immunization Costing Action Network (ICAN) Immunization Delivery Cost Catalogue. http://immunizationeconomics.org/ican-idcc.	
Cost effectiveness	**Does the cost-effectiveness of the intervention favor the intervention or the comparison?**<br><br>○ Favors the comparison<br>○ Probably favors the comparison<br>○ Does not favor either the intervention or the comparison<br>○ Probably favors the intervention<br>○ Favors the intervention<br><br>○ Varies<br>○ No included studies	A cost-effectiveness analysis compared vaccination with recombinant herpes zoster vaccine (RZV) vs. zoster vaccine live (ZVL) vs. no vaccination at age 50 or older.[21] **For vaccination with RZV compared with no vaccination, ICERs ranged by age from $10,000 to $47,000 per quality-adjusted life-year (QALY)**, using a societal perspective and assuming 100% completion of the 2-dose RZV regimen. For persons aged 60 years or older, ICERs were less than $60,000 per QALY. Vaccination with ZVL was dominated by vaccination with RZV for all age groups 60 years or older. **For those aged 50 to 59 years, RZV had an economically attractive ICER of $46,000 per QALY compared with no vaccination.**<br><br>A cost-utility analysis suggested that recombinant herpes zoster vaccine in persons aged ≥60 would be cost effective in the Canadian population compared with no vaccination and vaccination with live attenuated herpes zoster vaccine with an ICER of $28,360 per QALY.[22]	Most decision makers in the US consider cost < $50,000 to $60,000 per QALY gained as reasonably efficient.
Acceptability	**Is the intervention acceptable to key stakeholders?**<br><br>○ No<br>○ Probably no<br>○ Probably yes<br>○ Yes<br><br>○ Varies<br>○ Don't know		
Feasibility	**Is the intervention feasible to implement?**<br><br>○ No	In the US, demands exceed the supplies for Zoster vaccine.	

	○ Probably no<br>○ Probably yes<br>○ Yes<br><br>○ Varies<br>○ Don't know		

References:

1. Nugent Z, Singh H, Targownik LE, Bernstein CN. Herpes Zoster Infection and Herpes Zoster Vaccination in a Population-Based Sample of Persons With IBD: Is There Still an Unmet Need? Inflamm Bowel Dis. 2019 Feb 21;25(3):532-540.
2. Khan N, Patel D, Trivedi C, Shah Y, Lichtenstein G, Lewis J, Yang YX. Overall and Comparative Risk of Herpes Zoster With Pharmacotherapy for Inflammatory Bowel Diseases: A Nationwide Cohort Study. Clin Gastroenterol Hepatol. 2018 Dec;16(12):1919-1927.
3. Chang K, Lee HS, Kim YJ, Kim SO, Kim SH, Lee SH, Song EM, Hwang SW, Park SH, Yang DH, Ye BD, Byeon JS, Myung SJ, Yang SK. Increased Risk of Herpes Zoster Infection in Patients With Inflammatory Bowel Diseases in Korea. Clin Gastroenterol Hepatol. 2018 Dec;16(12):1928-1936.
4. Yun H, Yang S, Chen L, Xie F, Winthrop K, Baddley JW, Saag KG, Singh J, Curtis JR. Risk of Herpes Zoster in Autoimmune and Inflammatory Diseases: Implications for Vaccination. Arthritis Rheumatol. 2016 Sep;68(9):2328-37.
5. Tsai SY, Yang TY, Lin CL, Tsai YH, Kuo CF, Kao CH. Increased risk of varicella zoster virus infection in inflammatory bowel disease in an Asian population: a nationwide population-based cohort study. Int J Clin Pract. 2015 Feb;69(2):228-34.
6. Forbes HJ, Bhaskaran K, Thomas SL, Smeeth L, Clayton T, Langan SM. Quantification of risk factors for herpes zoster: population based case-control study. BMJ. 2014 May 13;348:g2911.
7. Long MD, Martin C, Sandler RS, Kappelman MD. Increased risk of herpes zoster among 108 604 patients with inflammatory bowel disease. Aliment Pharmacol Ther. 2013 Feb;37(4):420-9.
8. Marehbian J, Arrighi HM, Hass S, Tian H, Sandborn WJ. Adverse events associated with common therapy regimens for moderate-to-severe Crohn's disease. Am J Gastroenterol. 2009 Oct;104(10):2524-33.
9. Gupta G, Lautenbach E, Lewis JD. Incidence and risk factors for herpes zoster among patients with inflammatory bowel disease. Clin Gastroenterol Hepatol. 2006 Dec;4(12):1483-90.
10. Hanauer SB, Sandborn WJ, Feagan BG, Gasink C, Jacobstein D, Zou B, Johanns J, Adedokun OJ, Sands BE, Rutgeerts P, de Villiers WJS, Colombel JF, Ghosh S. IM-UNITI: 3 Year Efficacy, Safety, and Immunogenicity of Ustekinumab Treatment of Crohn's Disease. J Crohns Colitis. 2019 Jun 3.
11. Winthrop KL, Melmed GY, Vermeire S, Long MD, Chan G, Pedersen RD, Lawendy N, Thorpe AJ, Nduaka CI, Su C. Herpes Zoster Infection in Patients With Ulcerative Colitis Receiving Tofacitinib. Inflamm Bowel Dis. 2018 Sep 15;24(10):2258-2265.
12. Colombel JF, Sands BE, Rutgeerts P, Sandborn W, Danese S, D'Haens G, Panaccione R, Loftus EV Jr, Sankoh S, Fox I, Parikh A, Milch C, Abhyankar B, Feagan BG. The safety of vedolizumab for ulcerative colitis and Crohn's disease. Gut. 2017 May;66(5):839-851.
13. Marra F, Lo E, Kalashnikov V, Richardson K. Risk of Herpes Zoster in Individuals on Biologics, Disease-Modifying Antirheumatic Drugs, and/or Corticosteroids for Autoimmune Diseases: A Systematic Review and Meta-Analysis. Open Forum Infect Dis. 2016 Sep 28;3(4):ofw205.
14. Khan N, Trivedi C, Kavani H, Medvedeva E, Lewis J, Yang YX. Efficacy of Live Attenuated Herpes Zoster Vaccine in Patients With Inflammatory Bowel Diseases. Clin Gastroenterol Hepatol. 2019 Jun;17(7):1341-1347.

15. Zhang J, Xie F, Delzell E, Chen L, Winthrop KL, Lewis JD, Saag KG, Baddley JW, Curtis JR. Association between vaccination for herpes zoster and risk of herpes zoster infection among older patients with selected immune-mediated diseases. JAMA. 2012 Jul 4;308(1):43-9.
16. Zhang J, Delzell E, Xie F, Baddley JW, Spettell C, McMahan RM, Fernandes J, Chen L, Winthrop K, Curtis JR. The use, safety, and effectiveness of herpes zoster vaccination in individuals with inflammatory and autoimmune diseases: a longitudinal observational study. Arthritis Res Ther. 2011;13(5):R174.
17. Wasan SK, Zullow S, Berg A, Cheifetz AS, Ganley-Leal L, Farraye FA. Herpes Zoster Vaccine Response in Inflammatory Bowel Disease Patients on Low-dose Immunosuppression. Inflamm Bowel Dis. 2016 Jun;22(6):1391-6.
18. Khan N, Shah Y, Trivedi C, Lewis JD. Safety of herpes zoster vaccination among inflammatory bowel disease patients being treated with anti-TNF medications. Aliment Pharmacol Ther. 2017 Oct;46(7):668-672.
19. Lieu TA, Ray GT, Ortega-Sanchez IR, Kleinman K, Rusinak D, Prosser LA. Willingness to pay for a QALY based on community member and patient preferences for temporary health states associated with herpes zoster. Pharmacoeconomics. 2009;27(12):1005-16.
20. Eilers R, de Melker HE, Veldwijk J, Krabbe PFM. Vaccine preferences and acceptance of older adults. Vaccine. 2017 May 15;35(21):2823-2830
21. Prosser LA, Harpaz R, Rose AM, Gebremariam A, Guo A, Ortega-Sanchez IR, Zhou F, Dooling K. A Cost-Effectiveness Analysis of Vaccination for Prevention of Herpes Zoster and Related Complications: Input for National Recommendations. Ann Intern Med. 2019 Feb 19. doi: 10.7326/M18-2347. [Epub ahead of print]
22. McGirr A, Van Oorschot D, Widenmaier R, Stokes M, Ganz ML, Jung H, Varghese L, Curran D. Public Health Impact and Cost-Effectiveness of Non-live Adjuvanted Recombinant Zoster Vaccine in Canadian Adults. Appl Health Econ Health Policy. 2019 Jun 28.

Conclusion – Adults (50 years of age and older)

PICO 10A: In adult patients with IBD (50 years of age and older), should vaccination vs. no vaccination against herpes zoster (recombinant zoster vaccine) be given?

Moderate certainty of evidence

Direction – Yes (100%)

Strength – Strong (100%)

Type of recommendation	Strong recommendation against the intervention	Conditional recommendation against the intervention	Conditional recommendation for either the intervention or the comparison	Conditional recommendation for the intervention	Strong recommendation for the intervention
	○	○	○	○	○

**Recommendation**	**Statement 10A: In adult patients 50 years of age and older with IBD, we recommend recombinant zoster vaccine be given.**
**Justification**	
**Subgroup considerations**	
**Implementation considerations**	
**Monitoring and evaluation**	• Ongoing monitoring of safety and effectiveness of the recombinant zoster vaccine in IBD populations is needed.
**Research priorities**	• Assess effectiveness, safety, and duration of protection of the recombinant zoster vaccine in both younger and older IBD populations (age < 50 and >50, especially those who are on immunosuppressants). Outcomes should include serious complications related to herpes zoster such as PHN (rather than just shingles).

Evidence to Decision Table – Adults (younger than 50 years of age)

**PICO 10B**	**In adult patients with IBD (younger than 50 years of age), should vaccination vs. no vaccination against herpes zoster (recombinant zoster vaccine) be given?**
**Population**	Adult patients with IBD (50 years of age and older)
**Intervention**	Vaccination against herpes zoster (RZV, Shingrix®)
**Comparator**	No vaccination against herpes zoster
**Outcome**	Mortality, VPI (herpes zoster infection and complications), SAEs, Immunogenicity

<table>
<tr><th></th><th>Judgement</th><th>Research evidence</th><th>Additional considerations</th></tr>
<tr><td>Desirable Effects</td><td>How substantial are the desirable anticipated effects?

○ Trivial
○ Small
○ Moderate
○ Large

○ Varies
○ Don't know</td><td rowspan="2">See Evidence Profile Tables.

Risk of HZ in adult IBD patients
Nine case-control studies (considered as prognostic studies) addressed this PICO question.[1-9] All nine studies found an increased risk of herpes zoster (HZ) in IBD patients compared to the general population (1.2-1.8 times). The GRADE rating started at high as these studies provided prognostic evidence about the likelihood of HZ in patients with IBD when compared to non-IBD controls. The rating was eventually downgraded to <u>low</u> due to study limitations (detection bias, residual confounding factors) and indirectness (HZ as main outcome). In particular, IBD patients may have more frequent outpatient visits and/or hospitalization than non-IBD patients. This may lead to over-estimation of the risk of HZ in IBD patients. As well, all studies reported HZ as the main outcome, but did not report on more severe complications related to HZ (e.g. post-hepatic neuralgia, herpes zoster ophthalmicus, herpes zoster oticus, necrotizing retinitis, cranial and peripheral nerve palsies, myelopathy, meningoencephalitis, cerebellitis, and visceral involvement including pneumonitis, hepatitis, and acute retinal necrosis), which are more important to patients than a self-limited rash (shingles). In summary, there is <u>low</u> certainty evidence that adult IBD patients have an increased risk of HZ infection compared to non-IBD patients.

Six case-control studies (considered as prognostic studies) provided data on the risks of HZ stratified by age.[2-5,7,9] The only low risk of bias study (Khan 2018) suggested that the incidence of HZ among the younger IBD patients (age < 50) exceeded that of older control patients (age > 50) for whom the recombinant zoster vaccine (RZV, Shingrix®) is recommended by ACIP as the preferred vaccine for prevention of HZ and related complications. The rating of evidence for this subgroup analysis was further downgraded to very low due to imprecision since only a small number of IBD patients age < 50 had HZ. In summary, there is <u>low</u> certainty evidence that adult IBD patients older than age 50 have an increased risk of HZ infection compared to non-IBD patients older than age 50. There is <u>very low</u> certainty evidence that adult IBD patients younger than age 50 have an increased risk of HZ infection compared to non-IBD patients older than age 50.

Five case-control studies and one systematic review of RCTs and observational studies (considered as prognostic studies) provided data on the risks of HZ stratified by medication groups.[2-3,7-9,13] The use of steroids, thiopurines, anti-TNF, and combination therapy were associated with increased risks of HZ among IBD patients compared to IBD patients on no treatment / 5ASA or to the general population. The rating of evidence for this subgroup analysis was further downgraded to <u>very low</u> due to imprecision of effect estimates for anti-TNF and thiopurines with wide CIs, inconsistency with some studies suggesting no increased risks for HZ with anti-TNF and</td><td></td></tr>
<tr><td>Undesirable Effects</td><td>How substantial are the undesirable anticipated effects?

○ Large
○ Moderate
○ Small
○ Trivial

○ Varies
○ Don't know</td><td></td></tr>
</table>

thiopurines, and residual confounding with no adjustment for disease flare or severity in most studies. **In summary, there is <u>very low</u> certainty evidence that steroids, combination therapy (steroids + thiopurines or anti-TNF, steroids + thiopurines + anti-TNF, thiopurines + anti-TNF), thiopurines alone, and anti-TNF alone were associated with increased risks of HZ among IBD patients.**

Three single-arm RCTs (considered as prognostic studies) provided data on the incidence of HZ associated with the use of vedolizumab, ustekinumab, and tofacinib.[10-12] **There is <u>very low</u> certainty evidence that tofacinib is associated with an increased incidence of HZ in IBD patients, but vedolizumab and ustekinumab are not.**

Effectiveness and safety of HZV in adult IBD patients
There was no RCT comparing recombinant (or live attenuated) herpes zoster vaccine (HZV) with placebo or no treatment in adult patients with IBD to address this PICO question.

One case-control study in IBD patients, two case-control studies in patients with selected immune-mediated diseases including IBD, and a before-and-after study in IBD patients addressed this PICO question.[14-17] The two larger case-control studies[14,15] that included a larger number of vaccinated patients showed a significant reduction in the risk of HZ (39-46%) following vaccination with the live-attenuated zoster vaccine (LZV, Zostavax®). In a large case-control study, no significant risk reduction of herpes zoster infection was seen among patients vaccinated while on thiopurines compared with patients not vaccinated while on thiopurines (AHR 0.63, 95% CI 0.30-1.33).[14] There were too few patients who were vaccinated while on anti-TNF alone or in combination with a thiopurine to assess the association of their use with effectiveness of the vaccine.[14] The before-and-after study showed that IBD patients can mount an immune response to the live-attenuated zoster vaccine (LZV, Zostavax®) with a significant increase in HZ immunoglobulin G 2 weeks post vaccination, but the response was lower in patiens on low dose immunomodulators (methotrexate $\leq$ 0.4mg/kg/week, azathioprine $\leq$ 3.0mg/kg/d, 6MP $\leq$ 1.5mg/kg/d).[17] It is uncertain if this blunted response is clinically relevant and still would afford seroprotection. No serious adverse events were reported with the live attenuated herpes zoster vaccine.[17] The GRADE rating of these studies started as low due to their observational designs. The rating was downgraded to **<u>very low</u>** due to study limitations (selection bias, residual confounding) and indirectness (patient population, intervention, outcome). In particular, HZV may be selectively given to healthier patients (healthy vaccinee effect). This may have led to over-estimation of the protective effect of the vaccine and underestimation of adverse effects. As well, the PICO question pertains to recombinant zoster vaccine (not live attenuated zoster vaccine which was assessed in these studies). The studies reported HZ as the main outcome, but did not report on more severe complications related to HZ (e.g. post-hepatic neuralgia, herpes zoster ophthalmicus, herpes zoster oticus, necrotizing retinitis, cranial and peripheral nerve palsies, myelopathy, meningoencephalitis, cerebellitis, and visceral involvement including pneumonitis, hepatitis, and acute retinal necrosis), which are more important to patients than a self-limited rash (shingles). The incidence of HZ within 42 days following vaccination with

		live attenuated zoster vaccine in IBD patients was very low.<br><br>The CDC ACIP recommends herpes zoster recombinant vaccine in immunocompetent adults aged 50 years and older for the prevention of HZ and related complications based on **high level** of evidence for both safety and effectiveness. Since there were studies on clinical effectiveness, safety, and immunogenicity on live attenuated zoster vaccines in age-specific IBD populations that supported the findings in the general populations, the evidence was not downgraded for indirectness related to patient population (general population vs. IBD population). As per CDC, recombinant zoster vaccine is preferred over live attenuated vaccine due to higher effectiveness. Therefore, the evidence is also not downgraded for indirectness related to intervention. The evidence of adult IBD patients aged 50 years and older is therefore anchored to the general population. However, it is possible that herpes zoster vaccine may not be as effective in IBD patients on immunosuppressive medications. As well, only a small number of IBD patients on different types of immunosuppressive medications were included in these studies. Therefore, the evidence was downgraded 1 level to moderate for adult IBD patients on immunosuppressive medications. **Overall, there is high certainty evidence that herpes zoster vaccine is effective in adult IBD patients aged 50 years and older not on immunosuppressive medications. There is moderate certainty evidence that herpes zoster is effective in adult IBD patients aged 50 years and older on immunosuppressive medications. As there is serious imprecision with the estimate of serious adverse events related to use of zoster vaccine in IBD patients and all included studies assessed live attenuated vaccines (not recombinant vaccines), the evidence was downgraded to moderate for safety.**[15-18]<br><br>**Overall, there is moderate certainty evidence that recombinant zoster vaccine is safe and effective in adult IBD patients aged 50 and older on or not on immunosuppressives.**<br><br>As there were very few **younger IBD patients (age < 50)** who received the herpes zoster vaccine in the included studies, the benefits vs. risks of the recombinant zoster vaccine in this patient population are very uncertain. As well, no long-term studies on the duration of vaccine protection have been performed for patients younger than 50 years old. It remains unclear whether adults receiving the vaccine before age 50 will continue to be protected as they age. **If we extrapolate the evidence from older IBD patients (age > 50), the evidence would have to be downgraded to low for both safety and effectiveness.**	

**Certainty of evidence**	**What is the overall certainty of the evidence of effects?**<br><br>○ Very low<br>○ Low (for patients age < 50 on or not on IS)<br>○ Moderate<br>○ High<br><br>○ No included studies		
**Values and Preferences**	**Is there important uncertainty about or variability in how much people value the main outcomes?**<br><br>○ Important uncertainty or variability<br>○ Possibly important uncertainty or variability<br>○ Probably no important uncertainty or variability<br>○ No important uncertainty or variability	A survey was conducted to evaluate willingness to pay (WTP) for a quality-adjusted life-year (QALY) based on community member and patient preferences for temporary health states associated with herpes zoster.[19] The study showed that patients and community members gave mean WTP per QALY values that **varied significantly** based on age, sex, socioeconomic status, experience with shingles and duration of the health state evaluated. The WTP per QALY ranged from a trimmed mean of $US 26,000 to US 45,000 (year 2005 values). In multivariate analyses, the mean WTP per QALY was higher among respondents who were younger, male or had higher educational or income levels. After adjusting for these demographic variables, patients who had experienced shingles gave responses with the highest WTP per QALY values. Patients who had experienced PHN gave the lowest values, and community members gave values intermediate to the shingles and PHN groups. In multivariate models that evaluated the effects of pain and duration of the hypothetical zoster scenario, lower duration was associated with higher WTP per QALY. This effect appeared to be due to people increasing the amounts of time they would be willing to trade as duration increased, without proportional increases in the amounts of money they would be willing to pay. **The high variability in responses underscored the fact that preferences at the individual level may vary substantially.**<br><br>A discrete choice experiment was conducted to determine the relative importance of vaccine and disease specific characteristics and acceptance for Dutch older adults (age > 50).[20] The results suggest that older adults are most likely to accept pneumococcal vaccination of the 4 vaccines evaluated (pneumococcal, herpes zoster, pertussis, influenza). Potential vaccination rates of older adults were estimated at 58.1% for herpes zoster.	

<table>
<tr>
<td>Balance of effects</td>
<td>Does the balance between desirable and undesirable effects favor the intervention or the comparison?

o Favors the comparison
o Probably favors the comparison
o Does not favor either the intervention or the comparison
o Probably favors the intervention
o Favors the intervention

o Varies
o Don't know</td>
<td></td>
<td></td>
</tr>
<tr>
<td>Resources required</td>
<td>How large are the resource requirements (costs)?

o Large costs
o Moderate costs
o Negligible costs and savings
o Moderate savings
o Large savings

o Varies
o Don't know</td>
<td>CDC vaccine price list last reviewed/updated: July 1, 2019

<table><tr><th>Brandname</th><th>CDC cost/dose</th><th>Private sector cost/dose</th></tr><tr><td>Shingrix®</td><td>$102.90</td><td>$144.20</td></tr></table>
Shingrix is given as a 2-dose vaccine.</td>
<td></td>
</tr>
<tr>
<td>Certainty of Evidence of Required Resources</td>
<td>What is the certainty of the evidence of resource requirements (costs)?

o Very low
o Low
o Moderate
o High

o No included studies</td>
<td>The costs of delivering routine immunization services may vary widely across countries and different health system settings. See Immunization Costing Action Network (ICAN) Immunization Delivery Cost Catalogue. http://immunizationeconomics.org/ican-idcc.</td>
<td></td>
</tr>
</table>

**Cost effectiveness**	**Does the cost-effectiveness of the intervention favor the intervention or the comparison?**<br><br>○ Favors the comparison<br>○ Probably favors the comparison<br>○ Does not favor either the intervention or the comparison<br>○ Probably favors the intervention<br>○ Favors the intervention<br><br>○ Varies<br>○ No included studies	A cost-effectiveness analysis compared vaccination with recombinant herpes zoster vaccine (RZV) vs. zoster vaccine live (ZVL) vs. no vaccination at age 50 or older.[21] **For vaccination with RZV compared with no vaccination, ICERs ranged by age from $10,000 to $47,000 per quality-adjusted life-year (QALY)**, using a societal perspective and assuming 100% completion of the 2-dose RZV regimen. For persons aged 60 years or older, ICERs were less than $60,000 per QALY. Vaccination with ZVL was dominated by vaccination with RZV for all age groups 60 years or older. **For those aged 50 to 59 years, RZV had an economically attractive ICER of $46,000 per QALY compared with no vaccination.**<br><br>A cost-utility analysis suggested that recombinant herpes zoster vaccine in persons aged ≥60 would be cost effective in the Canadian population compared with no vaccination and vaccination with live attenuated herpes zoster vaccine with an ICER of $28,360 per QALY.[22]	Most decision makers in the US consider cost < $50,000 to $60,000 per QALY gained as reasonably efficient.
**Acceptability**	**Is the intervention acceptable to key stakeholders?**<br><br>○ No<br>○ Probably no<br>○ Probably yes<br>○ Yes<br><br>○ Varies<br>○ Don't know		
**Feasibility**	**Is the intervention feasible to implement?**<br><br>○ No<br>○ Probably no<br>○ Probably yes<br>○ Yes<br><br>○ Varies<br>○ Don't know	In the US, demands exceed the supplies for Zoster vaccine.	

Conclusion – Adults (younger than 50 years of age)

PICO 10B: In adult patients with IBD (younger than 50 years of age), should vaccination vs. no vaccination against herpes zoster (recombinant zoster vaccine) be given?

Direction – Yes (89%), Uncertain (11%)
Strength – Conditional
Low certainty of evidence

**Type of recommendation**	**Strong recommendation against the intervention**	**Conditional recommendation against the intervention**	**Conditional recommendation for either the intervention or the comparison**	**Conditional recommendation for the intervention**	**Strong recommendation for the intervention**
	o	o	o	o (highlighted)	o
**Recommendation**	**Statement 10B: In adult patients with IBD younger than 50 years of age, we suggest recombinant zoster vaccine be given.**				
**Justification**					
**Subgroup considerations**					
**Implementation considerations**					
**Monitoring and evaluation**	• Ongoing monitoring of safety and effectiveness of the recombinant zoster vaccine in IBD populations is needed.				
**Research priorities**	• Assess effectiveness, safety, and duration of protection of the recombinant zoster vaccine in				

	both younger and older IBD populations (age < 50 and >50, especially those who are on immunosuppressants). Outcomes should include serious complications related to herpes zoster such as PHN (rather than just shingles).

Hepatitis B

Background

Hepatitis B virus (Hep B) is transmitted via blood or sexual contact. Hep B can cause both acute and chronic illness. Initial infection may be asymptomatic in up to 50% of adults and 90% of children. The risk of fulminant hepatitis is increased with age and in pregnant women and has a case fatality rate of 1% to 2%. Although the majority of individuals infected will spontaneously clear the infection after 4 to 8 weeks, the risk of becoming a chronic carrier varies inversely with the age at which infection occurs. Infants have an 80% to 90% chance of becoming chronic carriers; children over one years and less than 5 years of age 25% to 50% chance; and adolescents and adults 3% to 10% chance.[1,2] The risk of becoming a chronic carrier is higher in immunocompromised individuals and in adults with diabetes or receiving hemodialysis. Chronic carriers are at risk of developing liver cirrhosis and hepatocellular carcinoma. The highest risk of transmission is in infants exposed during childbirth to their mothers who are carriers of Hep B. Other high-risk groups include injection drug users, household contacts of Hep B carriers, and people at risk of sexually transmitted diseases. In Canada, most cases of acute Hep B occur in unimmunized household contacts of Hep B carriers, and people 25 years of age and older who acquire infection through unprotected sexual activity, sharing drug injection equipment, or procedures with percutaneous exposure.[1]

It is estimated that more than 300 million individuals worldwide are Hep B carriers of whom approximately 500,000 to 1.2 million die annually from Hep B-related liver disease.[1] Canada is considered an area of low Hep B endemicity with an estimated less than 5% of Canadian residents having markers of past infection and less than 0.5% being carriers.[1] In the US, the incidence rate for new Hep B infection for 2016 was 1.0 cases per 100,000 population. The incidence of infection in all age groups has declined by up to 88.5% coinciding with increased vaccination usage.[2]

Both CDC ACIP and NACI recommend universal vaccination of infants and children.[1,2] The CDC ACIP recommends routine administration of 3 doses of Hep B vaccine in infants with the first dose being administered within 24 hours of birth. It also

recommends Hep B vaccination for all unvaccinated children and adolescents aged <19 years. NACI recommends routine vaccination of Hep B in children with age variable from jurisdiction to jurisdiction.

Both CDC ACIP and NACI recommend Hep B vaccine for unvaccinated adults at risk for Hep B infection using the standard 3 dose schedule:

- People who have immigrated to Canada from areas where there is a high prevalence of Hep B (NACI)
- Populations or communities in which Hep B is highly endemic
- People with lifestyle risks for infection, including
    - People whose sex partners have hepatitis B
    - Sexually active persons who are not in a long-term monogamous relationship
    - Persons seeking evaluation or treatment for a sexually transmitted disease
    - Men who have sexual contact with other men
    - People who share needles, syringes, or other drug-injection equipment
- People who have household contact with someone infected with the Hep B virus
- Health care and public safety workers at risk for exposure to blood or body fluids
- Residents and staff of facilities for developmentally disabled persons
- Persons in correctional facilities
- Travelers to regions with increased rates of hepatitis B
- People with chronic liver disease, kidney disease, HIV infection, infection with hepatitis C, or diabetes
- Anyone who wants to be protected from hepatitis B

The Infectious Diseases Society of America recommends that Hep B vaccination should *not* be withheld in patients with chronic inflammatory disorders on immunosuppressive medications because of concerns about exacerbation of chronic immune-mediated inflammatory illness (*strong recommendation, moderate quality evidence*).[3] ACG recommends vaccination against Hep B be administered as per ACIP guidelines (*conditional recommendation, very low level of evidence*).[4] The European Crohn's and Colitis Organization (ECCO) recommends Hep B vaccination in all HBV anti-HBcAb seronegative patients with IBD, and anti-HBs response be measured after vaccination.[5] Higher doses of the immunizing antigen may be required to provide protection.[5] As well, ECCO recommends maintenance of HBs antibody be monitored in patients at risk.[5]

Routine serologic testing before vaccination of infants, children, and adolescents is not recommended. However, the CDC endorses serologic testing prior to vaccination in persons receiving immunosuppressive therapy. Routine serologic testing after vaccination is

also not recommended except in people whose medical care depends on knowledge of their response to vaccine (e.g. immunocompromised people). The presence of anti-Hep B surface (anti-HBs) antibodies of at least 10 IU/L following completion of a recommended schedule in immunocompetent children and adults is considered seroprotection for life. The 3 dose vaccine series produces a protective antibody response in approximately 95% of healthy infants overall and >90% of healthy adults aged <40 years.[1,2] Exceptions are some immunocompromised persons (defined as congenital immunodeficiency, hematopoietic stem cell transplant, solid organ transplant recipients, HIV-infected) and people with chronic renal disease or on dialysis, who may require higher vaccine dose or periodic booster doses if their anti-HBs titer falls below 10 IU/L.[1,2] In immunocompetent individuals, although anti-HBs titers may become non-detectable over time, immune memory persists.

Serious adverse effects with Hep B vaccines are rare.[6] According to the CDC ACIP, Hepatitis B vaccines have been demonstrated to be safe when administered to infants, children, adolescents, and adults. The safety of hepatitis B vaccine is assessed continuously through ongoing monitoring of data from the Vaccine Safety Datalink (VSD), the Vaccine Adverse Events Reporting System (VAERS), and other post marketing surveillance systems. The estimated incidence of anaphylaxis among children and adolescents who received hepatitis B vaccine is one case per 1.1 million vaccine doses distributed (95% confidence interval 0.1-3.9). There are no other known causal safety concerns.

There have been reports of reactivation of HBV in chronic carriers while on infliximab.[7] Reactivation of HBV may manifest in different patterns ranging from abnormal liver enzymes to acute liver failure leading to death. In a large study assessing liver dysfunction related to HBV in patients with IBD (REPENTINA 2 study), 36% of HBsAg positive patients demonstrated evidence for liver dysfunction, including 6 patients who developed liver failure.[8] Using two or more immunosuppressants was an independent predictor HBV reactivation.[8]

References:

1. Public Health Agency of Canada. https://www.canada.ca/en/public-health/services/publications/healthy-living/canadian-immunization-guide-part-4-active-vaccines/page-7-hepatitis-b-vaccine.html#tab3
2. Schillie S, Vellozzi C, Reingold A, et al. Prevention of Hepatitis B Virus Infection in the United States: Recommendations of the Advisory Committee on Immunization Practices (ACIP). *MMWR* 2018; 67(1):1-31.
3. Rubin LG, Levin MJ, Ljungman P et al. 2013 IDSA Clinical Practice Guideline for Vaccination of the Immunocompromised Host. *Clinical Infectious Disease.* 2014; 58(3): e44-e100.
4. Farraye FA, Melmed GY, Lichtenstein GR, Kane SV. ACG Clinical Guideline: Preventive Care in Inflammatory Bowel Disease. Am J Gastroenterol. 2017 Feb;112(2):241-258.
5. Rahier JF, Magro F, Abreu C, Armuzzi A, Ben-Horin S, Chowers Y, Cottone M, de Ridder L, Doherty G, Ehehalt R, Esteve M, Katsanos K, Lees CW, Macmahon E, Moreels T, Reinisch W, Tilg H, Tremblay L, Veereman-Wauters G, Viget N, Yazdanpanah Y, Eliakim R, Colombel JF; European Crohn's and

Colitis Organisation (ECCO). Second European evidence-based consensus on the prevention, diagnosis and management of opportunistic infections in inflammatory bowel disease. J Crohns Colitis. 2014 Jun;8(6):443-68.
6. André FE. Summary of safety and efficacy data on a yeast-derived hepatitis B vaccine. *Am J Med* 1989;87(3a):S14–20.
7. Loomba R, Liang TJ. Hepatitis B Reactivation Associated With Immune Suppressive and Biological Modifier Therapies: Current Concepts, Management Strategies, and Future Directions. Gastroenterology. 2017 May;152(6):1297-1309.
8. Loras C, Gisbert JP, Mínguez M, Merino O, Bujanda L, Saro C, Domenech E, Barrio J, Andreu M, Ordás I, Vida L, Bastida G, González-Huix F, Piqueras M, Ginard D, Calvet X, Gutiérrez A, Abad A, Torres M, Panés J, Chaparro M, Pascual I, Rodriguez-Carballeira M, Fernández-Bañares F, Viver JM, Esteve M; REPENTINA study; GETECCU (Grupo Español de Enfermedades de Crohn y Colitis Ulcerosa) Group. Liver dysfunction related to hepatitis B and C in patients with inflammatory bowel disease treated with immunosuppressive therapy. Gut. 2010 Oct;59(10):1340-6.

Risk of hepatitis B infection in IBD patients

PICO: What is the risk of Hep B infection in people with IBD compared to people without IBD?

Summary

Adults

Ten cross-sectional observational studies addressed this PICO question.[1-10] Older observational studies in the Western countries showed higher prevalence of past HBV infection among IBD patients compared to the general population. However, more recent observational studies in Western countries suggested that the prevalence of present and past HBV infection in IBD patients is similar to that in the general population perhaps due to more satisfactory preventative measures in hospitals, better decontamination of surgical and endoscopic equipment, more effective screening of blood products, increased vaccination coverage, and IBD patients' avoidance of risk-associated behavior. In Eastern countries where HBV is endemic, the prevalence rates of past HBV infection (not present infection) in IBD populations appear to be higher than in the general population. It is however difficult to compare prevalence of HBV infection across studies because of variations in prevalence of HBV infection, policy of infection control, implementation of vaccination programs over time in different countries and also within the same country, and differences in the mean age of patients included in each of these studies. Therefore, the studies were not pooled together in a meta-analysis.

The GRADE rating started as high as these were considered prognostic studies (providing evidence that the likelihood of HBV infection in patients with IBD). The rating was further downgraded to **very low** due to study limitations (residual confounding factors, detection bias, admission bias), indirectness (populations and outcomes), and inconsistency. Hospitalized patients or highly

selected patients in tertiary care centers were included, and not all patients with IBD. Patients with IBD may be more likely to be screened for Hepatitis B infection due to more outpatient visits and hospitalization compared to the general population (detection bias related to increased health care utilization). As well, serologic outcomes were used to estimate patient-important outcomes such as chronic active infection, liver cirrhosis, and/or hepatocellular carcinoma. Present and past HBV infection in the included studies was defined according to the terminology adopted by the National Institutes of Health Conferences on management of Hepatitis B. Present HBV infection is defined by positive HBsAg and included chronic hepatitis B and inactive HBsAg carrier state. However, inactive carrier state carries a very good prognosis in the spectrum of chronic HBV infection, with low rates of reactivation, hepatocellular carcinoma and progression of disease to cirrhosis. In contrast, chronic hepatitis B infection (HBeAg positive or HBeAg negative) has a higher risk of progression to liver cirrhosis and/or hepatocellular carcinoma. Cross-sectional designs cannot distinguish these two entities as inactive HBsAg carrier state is diagnosed by absence of HBeAg and presence of anti-HBe, undetectable levels of HBV DNA in PCR, repeatedly normal ALT, and minimal or no necroinflammation, slight fibrosis, or even histology on biopsy (although biopsy is not indicated to make the diagnosis in these patients). A minimum- follow-up of 1 year with ALT levels at least every 3- 4months and serum HBV DNA level is required before classifying a patient as inactive HBV carrier. As well, past HBV infection included resolved hepatitis B defined by presence of anti-HBc with or without anti-HBs. HBV DNA levels were not measured in HBsAg negative patients with anti-HBc, therefore, the level of occult HBV infection is unknown. Anti-HBc positive patients with occult infection may have reactivation of infection during treatment with immunosuppressives. **In summary, there is <u>very low</u> certainty evidence that adult IBD patients have a comparable (or increased risk) of HBV infection compared to non-IBD patients.**

Pediatric

Literature search did not identify any study on the risk of Hep B infection in pediatric IBD patients.

Risk of Bias Table

Prognostic studies							
**Study**	**Study sample adequately represents the population of**	**Study data available adequately represent the**	**Prognostic factor measured in a similar and**	**Outcome of interest is measured in a similar and**	**Important potential confounding factors are appropriately accounted for**	**Statistical analysis is appropriate, and all primary**	**Comments**

	interest	study sample (>80% follow-up)	valid way for all participants	valid way for all participants		outcomes are reported	
Chen 2017 (China)	Study included only hospitalized IBD patients who were screened for Hep B or Hep C (not consecutive patients), but most first time suspected IBD patients were hospitalized. IBD patients who were not screened were excluded. Possible selection bias	OK	OK	Data were reliant on administrative discharge diagnoses. Possible misclassification errors due to errors of miscoding, and the codes have not been previously validated.	Possible residual confounding factors: Only surgery and blood transfusions were accounted for. Other risk factors for hepatitis B such as risky sexual behavior and drug abuse were not accounted for. Healthy controls who attended routine health examinations may be prognostically different than IBD patients who were hospitalized and screened for Hep B infection (different degree of health seeking behavior; healthy volunteer effect). This may have over-estimated the risk of Hep B infection in IBD populations.<br><br>Detection and Admission bias: Patients with IBD may be more likely to be admitted to hospital and	OK	• Retrospective cohort study in 2 centers in Shanghai, China, looking at prevalence of Hep B infection amongst IBD patients<br>• Cases: 980 newly diagnosed IBD between January 2006 and December 2015.<br>• Controls: 2488 age and sex matched individuals attending the 2 hospitals for routine health examinations<br>• **IBD patients were found to have a higher rate of past Hep B infection defined as anti-HBc positive (41.22% vs 35.85%, p = 0.003) but no differences in present infection defined as HBsAg positive (7.86% vs 6.59%, p = 0.187)**<br>• **The prevalence of past infection was higher in Ulcerative Colitis than in Crohn Disease (OR 0.62, 95% CI: 0.46-0.84)**

					therefore screened for Hep B thus leading to an overestimate in the IBD cohort. Patients with comorbidities and Hep B may be sicker and thus more likely to be admitted to hospital.		
Huang 2014 (China)	Study included only hospitalized IBD patients. All patients were screened for HBV and HCV.	OK	OK	Data were reliant on administrative discharge diagnoses. Possible misclassification errors due to errors of miscoding, and the codes have not been previously validated.	Possible residual confounding factors: Other risk factors for hepatitis B such as risky sexual behavior and drug abuse were not accounted for. Healthy controls who attended routine health examinations may be prognostically different than IBD patients who were hospitalized and screened for Hep B infection (different degree of health seeking behavior; healthy volunteer effect). This may have over-estimated the risk of Hep B infection in IBD populations.<br><br>Detection and	OK	• Retrospective single center cohort study in Shanghai, China between January 2001 and August 2012<br>• Cases: 714 inpatients with IBD admitted to hospital, who were then screened for Hep B infection<br>• Controls: 22,373 age and sex matched healthy controls presenting for routine health examinations<br>• **IBD patients were found to have a higher rate of past Hep B infection defined as anti-HBc positive +/- anti-HBs (40.62% vs 27.58%), but no differences in present infection defined as HBsAg positive (5.46% vs 5.52%).**

					<u>Admission bias:</u> Patients with IBD may be more likely to be admitted to hospital and therefore screened for Hep B thus leading to an overestimate in the IBD cohort. Patients with comorbidities and Hep B may be sicker and thus more likely to be admitted to hospital.		
Loras 2009 (Spain)	Study included only consecutively hospitalized IBD patients. All patients were screened for HBV and HCV.	OK	OK	OK	<u>Detection and Admission bias:</u> Patients with IBD may be more likely to be admitted to hospital and therefore screened for Hep B thus leading to an overestimate in the IBD cohort. Patients with comorbidities and Hep B may be sicker and thus more likely to be admitted to hospital.	OK	• Prospective cross-sectional multicenter cohort study in 17 Spanish hospitals<br>• <u>Cases</u>: 2,076 consecutively recruited IBD patients visiting those hospitals<br>• <u>Controls</u>: No controls<br>• **Crohn Disease patients had a prevalence of 0.6% HepBsAg and 7.1% anti-HepBc. Ulcerative Colitis patients had a rate of 0.8% HepBsAg and 8% anti-HepBc. This compared with that found in the general population in Spain from other publications (1996-2007), which ranged from 0.7 to 1.7% for HepBsAg and 8.7 to 10.6% for anti-HepBc**

Kim 2014 (Korea)	Unclear how patients were selected but HepBsAg and anti HepBsAb were assessed in all hospitalized patients. Anti-HepBcAb was assessed in a subset of patients (357 of 513)	OK	OK	Unclear	Possible residual confounding factors: High risk sexual behavior and parenteral drug use were risk factors not accounted for. Healthy controls who attended routine health examinations may be prognostically different than IBD patients who were hospitalized and screened for Hep B infection (different degree of health seeking behavior; healthy volunteer effect).	OK	• Cross-sectional multicenter observational study that was conducted at 5 tertiary referral hospitals in southeastern Korea<br>• Cases: 513 IBD cases with >6 months disease duration between 2009-2011.<br>• Controls: 1040 age and sex matched controls who attended the hospital for routine medical check-up.<br>• **Overall, IBD patients did not differ significantly from controls in terms of Hep B infection though patients with Crohn Disease had lower rates of infection than controls (HepBsAg 4.4% in gen population compared to UC: 3.3%, CD: 4.1%; HepBcAb 35.9% gen population vs UC: 35.2%; CD: 23.8%).**<br>• **Age <30 yrs was associated with risk of non-immunity despite national vaccination program since 1991 (OR 2.5, 1.23-5.07)**
	Single center with large outpatient IBD clinic. Screening for Hep B was performed in	OK	OK	OK	Possible residual confounding factors: Hep B vaccination status, history of drug use and high-risk sexual behavior not accounted for.	OK	• Single center retrospective cohort study<br>• Cases: 482 outpatient IBD clinic patients were screened for Hep B as they presented for routine IBD clinic follow up.

Katsanos 2010 (Balkans)	all patients presenting for routine clinic follow up rather than all IBD. Possible selection bias as highly selected IBD patients were seen in this tertiary referral center.				More health conscious, low risk behavior patients may be more likely to present for follow up than high risk behavior IBD patients and thus have lower rates of infection than control patients.		• Controls: No controls<br>• The IBD population had a prevalence of 2.3% of HBV infection, comparable to that of the local population of Northwest Greece which was estimated at "never exceeding 3%".<br>• Of note, routine vaccination of infants started in the early 90's in parallel with screening of all pregnant women. Vaccination of 11-year olds started in 1998 with 90% estimated coverage
Biancone 2001 (Italy)	It is unclear if cases included only hospitalized IBD patients or outpatients as well. Possible selection bias as patients were recruited from tertiary referral centers.	OK	OK	OK	Possible residual confounding factors: Other risk factors for hepatitis B such as risky sexual behavior and drug abuse were not accounted for.<br><br>Detection and Admission bias: Patients with IBD may be more likely to be admitted to hospital and therefore screened for Hep B thus leading to an overestimate in the IBD cohort. Patients with comorbid Hep		• Cases: 332 patients with Crohn Disease attending seven Gastrointestinal Units from different areas of Italy were prospectively recruited<br>• Controls: 374 subjects with no known risk factors for HBV or HCV infections attending as in- or outpatients at Endocrinologic or Cardiologic Units from the same institutions were tested. A disease control group consisted of 162 Ulcerative Colitis patients recruited over the same time period from the same GI units as the CD patients.

					B may be been sicker and thus more likely to be admitted to hospital.		• **Hep BsAg was 2.1% amongst this group as well as controls and no differences were found between Crohn Disease and Ulcerative Colitis. However, evidence of past infection was higher in the IBD group compared to the general population (CD 10.9% vs UC 11.5% vs Controls 5.1%; p=0.01 and 0.02 respectively).**
Chevaux 2010 (France)	Some patients at higher risk for Hep B or Hep C may not have consented for the study. Possible selection bias. Patients were from a tertiary care center and were part of a global ongoing study aimed at determining the environmental and genetic risk factors associated with IBD.	OK	OK	OK	Possible residual confounding factors: Other risk factors for hepatitis B such as risky sexual behavior, drug abuse, tattoos and piercings comorbidities, medication, blood transfusion and disease activity were not accounted for.<br><br>Vaccination rates may have been influenced by education, socioeconomic status and higher health resource utilization compared to general population.	OK	• Prospective cohort study from 2005-2009.<br>• Cases: 315 consecutive inpatient and outpatients seen in single center hospital.<br>• Controls: regional prevalence estimates based on national epidemiologic study on the prevalence of HBV and HCV performed by the French Institute of Health survey, 2004<br>• **Overall, there was no difference between the IBD cohort and the general population in the prevalence of HBsAg (0.95% vs 1.12%). Prevalence of anti-HBc in IBD was significantly lower than in the general population (2.54% vs.**

					The IBD population may be more health conscious, low risk behavior patients lower rates of infection than control patients).		**8.3%)**<br>• **Hep B infection (HBsAg positive) was 1.59% in UC and 0.79% in CD. Past infection (anti-HBc) was 1.59% in UC and 2.78% in CD.**<br>• Effective vaccination was detected in 48.9% of IBD patients
Tolentino 2008 (Brazil)	Only included outpatients.<br><br>"Patients were selected, weekly according to their order of arrival for the medical interview"<br><br>Some patients at higher risk for Hep B or Hep C may not have consented for the study.<br><br>Possible selection bias as patients were from a referral center for IBD.	OK	OK	OK	<u>Possible residual confounding factors:</u> Other risk factors for hepatitis B such as risky sexual behavior and drug abuse were not accounted for.<br><br><u>Detection bias:</u> Patients with IBD may be more likely to be seen in clinic and have more regular follow-up, and therefore more likely to be screened for Hep B thus leading to an overestimate in the IBD cohort. l	OK	• Prospective cohort study of outpatients in large university referral hospital<br>• <u>Cases:</u> 176 consecutively recruited patients attending IBD clinic<br>• <u>Controls</u>: General Brazilian population estimates<br>• Risk factors including gender, endoscopy, blood transfusion, surgeries, duration of disease, age, tattoos and piercings, sexual lifestyle, drug use and dialysis were examined<br>• **17% of IBD patients had evidence of past infection (anti-HBc) and 2.3 % present infection (HBsAg).**<br>• **Prevalence of past infection (anti-HBc) was higher in IBD patients than the overall Brazil population (17% vs. 7.9%) based on 2005 data**<br>• **Prevalence of present**

							**infection (HBsAg) was higher in IBD patients vs. general population (2.3% vs. 0.5%)**<br>• **Older age at testing was associated with higher rates of anti-HepBcAb (mean age 47.7 vs 39, p=0.001)**<br>• **Patients who underwent IBD-related surgeries were exposed to more blood transfusions (43.6 vs 22.6%, P=0.015) but transfusion was not found to be an independent risk factor for infection.**
Ardesia 2017 (Italy)	Only included outpatients.<br><br>Possible selection bias as patients were from a referral center for IBD.	OK	OK	OK	Possible residual confounding factors: Other risk factors for hepatitis B such as risky sexual behavior, drug abuse, tattoos and piercings comorbidities, medication, blood transfusion and disease activity were not accounted for and may have led to an overestimation of Hep B infection in the IBD population.	OK	• Retrospective cohort study<br>• Cases: 509 IBD patients undergoing baseline infectious screening prior to starting thiopurines or biologics<br>• Controls: No comparator group was provided.<br>• In the nonvaccinated population, that is, patients aged >37 years, past Hep B infection (HepBcAb) was found in 9.6% and 8.4% in CD and UC, respectively. In this age group, present infection (HepBsAg) was found in 2% and 1.6% in CD and UC, respectively.<br>• In the vaccinated group,

							age ≤37 yrs, HepBcAb was found in 2.2% and 2.9% in CD and UC, respectively. HepBsAg was present in 2% and 1.4% respectively.
He 2015 (China)	Does not describe if patients were ambulatory or inpatients. Data taken from database at large university hospital. Possible selection bias.	OK	OK	OK	Possible residual confounding factors: Other risk factors for hepatitis B such as risky sexual behavior, drug abuse, tattoos and piercings comorbidities, medication, blood transfusion and disease activity were not accounted for.	OK	• Retrospective cohort study from July 2006-July 2012.<br>• Cases: 675 consecutive IBD patients<br>• Controls: General population data from physical examination center at university hospital.<br>• Present infection (HBsAg) was not different amongst the groups (13.6% CD, 16.8% UC and 13.8% in general population).<br>• Past infection: 25.4% CD, 30.1% UC<br>• Male gender, older age, family history of HepB infection were associated with HepBsAg positivity.<br>• No differences in IBD therapies, blood transfusions or previous surgeries were found.

Certainty Assessment								Summary of Findings	
Studies	Risk of bias	Inconsistency	Indirectness	Imprecision	Other considerations	Certainty of Evidence	Overall Certainty of evidence	Study Event Rates	Relative Effect (95% CI)
**VPI (Hepatitis B infection) - Critical**									
10 Observational studies[1-10] (prognostic studies)	Serious[a]	Serious[b]	Serious[c]	Not serious	None	⊕⊖⊖⊖ VERY LOW	⊕⊖⊖⊖ VERY LOW	See **Summary of Risk of Hepatitis B Infection in IBD Populations.** Recent studies from Western countries did not find an increased prevalence of past or present Hep B infection compared to the general population. Increased prevalence of past infection (but not present infection) in high endemic areas (Asia).	

Footnotes:

a. Downgraded for study limitations. High risk for detection and admission bias with 4 studies included only hospitalized patients. 2 studies did not detail where patients were recruited from. 3 studies recruited only outpatients. 6 studies were based in large tertiary referral centers introducing possible selection bias. Most studies did not account for possible residual confounding factors for Hep B infection risk or vaccination use. Did not account for health service utilization. It is possible patients with IBD were screened more often for Hep B than the general population due to regular follow-up and hospitalization.

b. Downgraded for inconsistency. More recent Asian studies and older Western studies showed increased risk or prevalence of past Hep B infection. More recent Western studies did not show increased risk of past or present hepatitis B infection. No serious inconsistency for present infection likely due to introduction of national immunization program.

c. Downgraded for indirectness of outcome. Most studies reported serological positivity as the primary outcome and did not address patient-important outcomes related to fulminant hepatitis, cirrhosis, hepatocellular carcinoma and death related to hepatitis B infection.

Summary of the Risk of Hepatitis B Infection in IBD Populations

Study	Present infection in GP	Present infection in IBD populations	Past infection in GP	Past infection in IBD populations	Risk factors for infection
Chen 2017[1] (China)	6.59%	7.86%	35.85%	41.22%	• Age at sampling for UC and previous surgery were independent risk factors for HBV infection.
Ardesia 2017[2]	Not reported	≤37 yrs age 1.7%	Southern Italy 11.2%	≤37 yrs age 2.5%	• Older age at sampling, but

(Italy)		>37 yrs age 1.8%		>37 yrs age 9.0%	influenced by introduction of compulsory vaccination in 1979
He 2015[3] (China)	13.8%	UC: 16.8%<br>CD: 13.6%	Not reported	UC: 30.1%<br>CD: 25.4%	• Male gender, older age, family history of Hep B infection, were associated with HepBsAg positivity
Huang 2014[4] (China)	5.52%	5.46%	27.58%	40.62%	• Age, family history of hepatitis, and a previous IBD-related admission were independent risk factors for HBV infection.
Kim 2014[5] (Korea)	4.4%	UC: 3.3%<br>CD: 4.1%	35.9%	UC: 35.2%<br>CD: 23.8%<br>(lower in CD than GP)	• Age <30 yrs at sampling was a risk factor for non-immunity.
Chevaux 2010[6] (France)	0.95-1.12%	UC: 1.59%<br>CD: 0.79%	8.3%	CD: 2.78%<br>UC: 1.59%<br>(lower in IBD than GP)	• Not investigated
Katsanos 2010[7] (Balkans)	3%	2.3%	Not reported	Not reported	• Not investigated
Loras 2009[8] (Spain)	0.7-1.7%	UC: 0.8%<br>CD: 0.6%<br>Indeterminate: 0%	8.7-10.6%	UC: 8%<br>CD: 7.1%<br>Indeterminate: 5.3%	• Age, family history of hepatitis, and moderate-to-severe disease were independent risk factors for HBV infection.
Tolentino 2008[9] (Brazil)	0.5%	2.3%	Brazil: 7.9%<br>Rio de Janeiro state: 2.5%	17%	• Older age at testing was associated with higher rates of anti-HBcAb
Biancone 2001[10] (Italy)	2.1%	2.1%	5.1%	UC: 11.5%<br>CD: 10.9%	• HBcAb positivity was associated with age, southern area, female gender in CD and to UC duration.

GP: general population

As defined by primary studies:

- Present infection: HBsAg positive
- Past infection: Anti-HBc positive with or without anti-HBs

Compared to healthy people, risks were significantly higher (yellow shading) or lower (green shading)

References:

1. Chen D, Luo S, Ben q et al. Prevalence of hepatitis B and C and factors for infection and nonimmune in inflammatory bowel disease patients in China. Eur J Gastro & Hep. 2017 29(5): 509-515
2. Ardesia M, Constantino G, Mondello P et al. Serology of Viral Infections and Tuberculosis Screening in an IBD Population Referred to a Tertiary Centre of Southern Italy. Gastroenterol Res Pract. 2017;2017:4139656.
3. He Y, Xu P, Chen Y et al. Prevalence and influences of hepatitis B virus infection on inflammatory bowel disease: a retrospective study in southern China. Int J Clin Exp Med. 2015, 8(5): 8078-8085.
4. Huang ML, Xu XT, Shen J et al. Prevalence and factors related to hepatitis B and C infection in inflammatory bowel disease patients in China: A retrospective study. J Crohns Colitis. 2014, 8(4); 282-287.
5. Kim ES, Cho KB, Park KS et al. Prevalence of Hepatitis B Viral Markers in patients with Inflammatory Bowel Disease in a Hepatitis B Endemic Area: Inadequate protective antibody levels in young patients. J Clin Gastroenterol. 2014, 48: 553-558.
6. Chevaux JB, Nani A, Oussalah A et al. Prevalence of Hepatitis B and C and Risk Factors for Nonvaccination in Inflammatory Bowel Disease Patients in Northeast France. Inflamm Bowel Dis. 2010, 16: 916-924.
7. Katsanos KH, Tsianos VE, Zois CD et al. Inflammatory bowel disease and hepatitis B and C in Western Balkans: a referral centre study and review of the literature. J Crohns Colitis 2010, 4(4): 450-65.
8. Loras C, Saro C, Gonzalez-Huix F et al. Prevalence and factors related to hepatitis B and C in Inflammatory Bowel Disease patients in Spain: A nationwide, multicenter study. Am J Gastro. 2009, 104(1): 57-63.
9. Tolentino YFM, Fogaca HS, Zaltman C et al. Hepatitis B virus prevalence and transmission risk factors in inflammatory bowel disease patients at Clementino Fraga Filho university hospital. World J Gastroenterol. 2008, 14(20): 3201-3206.
10. Biancone L, Pavia M, Del Vecchio Blanco G et al. Hepatitis B and C virus in Crohn's Disease. Inflamm Bowel Dis. 2001, 7(4): 287-94.

Effectiveness and Safety of hepatitis B vaccine in IBD patients

Summary – Pediatric

**PICO 11**	**In pediatric patients with IBD, should vaccination vs. no vaccination against hepatitis B be given?**
**Population**	Pediatric patients with IBD

Intervention	Vaccination against hepatitis B
Comparator	No vaccination against hepatitis B
Outcome	Mortality, VPI (hepatitis B infection), SAEs, Immunogenicity

There were no RCTs or observational studies comparing Hep B vaccination versus placebo or no vaccination in pediatric patients with IBD to address this PICO question.

One systematic review of 4 RCTs assessed the effectiveness and safety of HBV vaccination in infants born to hepatitis B surface antigen-positive mothers.[1] Compared with placebo or no intervention, vaccine reduced hepatitis B occurrence (RR 0.28, 95% CI 0.20-0.40). Most trials were unclear risk of bias for allocation concealment.[1] The evidence was graded as high for effectiveness, but downgraded 1 level due to study limitations. Vaccination programmes against hepatitis B was shown to be very effective in 2 large observational studies with long follow-up, as evident by a dramatic decrease in the incidence of hepatocellular carcinoma (60.1%), mortality due to fulminant hepatic failure (76.3%), and mortality due to chronic liver diseases (92.0%), as observed among vaccinated persons in Taiwan over the decades since vaccine introduction.[2,3] The evidence started as low, but upgraded 2 levels due to large effect size.[2,3]

Four cross-sectional studies and one case-control study assessed seroprotection (defined as anti-HBs > 10 IU/L) in children with IBD.[4-8] Among the cross-sectional studies, the seroconversion rates against HBV vaccines ranged from 28% to 71.3% in pediatric IBD patients.[4-7] **Due to the cross-sectional nature of these studies, they cannot differentiate between lack of primary antibody response to HBV vaccine vs. loss of antibody levels with time from vaccination**. Yet, the clinical significance of loss of anti-HBs titers in patients with IBD is unknown. Anti-HBs titers frequently become undetectable over time in healthy persons. A number of long-term studies performed in different epidemiological contexts have confirmed that clinical HBV infection rarely occurs among successfully vaccinated people, even though anti-HBs titers decline to < 10 IU/L. Therefore, protection against breakthrough HBV infection may be dependent on immunologic memory rather than on anti-HBs levels. However, clinically significant HBV infection has been documented in immunocompromised responders (HIV and those undergoing hemodialysis) who do not maintain anti-HBs concentration > 10 IU/L**.** Therefore, the CDC recommends annual anti-HBs testing for these patients and a booster dose be administered when anti-HBs levels decrease to < 10 IU/L. However, for other immunocompromised patients (e.g. IBD), the need for booster is uncertain (risks for contracting HBV may not be as high as patients with HIV or on hemodialysis; no study or report on the risks of breakthrough HBV infection in previously vaccinated IBD patients with low anti-HBs level). As well, there were different proportions of pateints on different types of immunosuppressive medications across studies. Three cross-sectional studies reported no significant association between the use of immunosuppressive medications and the seroconversion rates among pediatric IBD

patients, whereas one did not assess this association.[4-7] One case-control study found lower seroconversion rates in children with IBD vs. healthy control after primary vaccination against HBV (70.2% vs. 90%, P = 0.02).[8] The overall seroprotection rates after administering single dose booster to non-responders were not statistically different between the 2 groups (85.1% in IBD patients vs. 96% healthy controls, P = 0.08).[8] No significant association was found between treatment and vaccination response.[8] No serious adverse reactions or exacerbation of IBD was reported.[8]

The evidence suggests that HBV vaccine can induce seroconversion or seroresponse in a significant proportion of pediatric IBD patients (although the response appears to be reduced compared to the general population). Use of immunosuppressive medications may not affect the immunologic response to HBV vaccination in pediatric IBD patients. The seroconversion rates may wane over time with reduction in anti-HBs titer. However, it is uncertain if this reduced immunologic response is clinically relevant/important and would still afford clinical protection as no studies have assessed patient-important clinical outcomes. No serious adverse events including disease exacerbation was reported. The GRADE rating started at low due to the observational designs of the studies. The rating was downgraded to very low due to study limitations (selection bias, residual confounding), indirectness (use of surrogate outcomes), and imprecision. **The evidence for effectiveness was anchored to the general population (high certainty), and was downgraded to moderate due to indirectness as immunogenicity studies suggested that HBV vaccine may be less immunogenic (and therefore less effective) in pediatric IBD patients.**

In terms of safety, the CDC assessed the evidence of HBV vaccine in adult persons with diabetes, although most of the included trials were high risk adults with no history of diabetes.[9-14] No serious adverse events were reported by any of the included trials.[9-14] CDC rated the certainty of evidence as high for safety.[9-14] The evidence was downgraded to moderate due to indirectness (adult vs. pediatric population; sample sizes in IBD studies were not sufficient to detect rare adverse events).[9-14] The systematic review that included infants born to hepatitis B surface antigen-positive mothers showed HBV vaccine to be safe, but few trials reported on serious adverse events.[1] The evidence was downgraded to low due to study limitations and indirectness (non-IBD population).[1] The Vaccine Adverse Event Reporting System (VAERS) received 2588/20,231 reports following HBV vaccination in persons 2-18 years from 2005-2015.[15] 6.8% of the reports were serious, including 45 deaths.[15] Most commonly reported case of death was Sudden Infant Death Syndrome. Most common non-death serious reports among infants aged < 1 month were nervous system disorders among children aged 1-23 months, and infections among persons age 2- 18 years.[15] No causal link has been established between HBV vaccines and these adverse events.[15] The evidence was downgraded to very low due to study limitations and indirectness (non-IBD population).[15] In the case-control study involving pediatric IBD patients (n = 47), no serious adverse events were reported after HBV vaccination.[8] The evidence was downgraded to very low due to study limitations and imprecision.[8] **The evidence for safety was**

anchored to the general population, and was downgraded to <u>moderate</u> due to indirectness (sample size in the IBD study was insufficient to detect rare adverse events).

Overall, there is <u>moderate</u> certainty evidence that HBV vaccine is safe and effective in pediatric IBD populations.

Revaccination following primary vaccination failure

Two cross-sectional studies and one case-conrol study assessed revaccination with a single booster dose following primary vaccination failure in pediatric IBD patients.[6-8] No studies assessed repeat vaccination with 3-dose series. The response rate of revaccination by single booster dose ranged from 50-76%.[6-8] The GRADE rating started at low due to the observational nature of these studies. The rating was downgraded to very low due to study limitations, indirectness (surrogate outcomes) and imprecision. **In summary, there is <u>very low</u> certainty evidence that repeat vaccination with a booster dose is safe and effective in reducing the risks of HBV infection in pediatric IBD patients following primary vaccination failure.**

Risk of Bias Table – Pediatric

Cohort studies							
**Study**	**Valid methods to ascertain exposure**	**Prognostic factors (other than exposure of interest) similar among cohorts – or cohorts were adjusted adequately for confounders**	**Demonstration that outcome of interest was not present at the start of the study**	**Outcome detection methods valid and similar among cohorts**	**Follow-up complete and similar among cohorts**	**Free of other bias**	**Comments**
deBruyn 2018 (Canada)	Vaccination records and baseline serology were used to determine immunity against vaccine preventable diseases including	IBD subtype, current immunosuppressive medication use, age at diagnosis, and age at serum collection were adjusted for in a multivariate analysis.	OK	OK	OK	Possible selection bias. Patients attending a tertiary referral center may differ systematically from other	• Cross sectional study in children examining the serologic status of childhood vaccinatable diseases<br>• 156 children with IBD at a Canadian tertiary referral IBD unit.<br>• Vaccination coverage for

	HepB.	Disease activity at time of vaccination, duration of disease and nutritional status were not accounted for.				patients. Patients who agreed to participate in a study where serologic assays were measured may be different than patients who did not agree to participate.	Hep B was up to date in 75.8% of patients.<br>• **Seroconversion among participants with complete vaccine series (n = 115; anti-HBs titer ≥ 10 IU/L): 71.3%**<br>• **Seroconversion among immunosuppressed (93): 61.3%**<br>• Cannot differentiate between lack of primary antibody response to HBV vaccine vs. loss of antibody levels with time from vaccination (clinical relevance of waning titer over time is unclear)<br>• Older age at diagnosis was associated with seroprotection among subjects with complete HBV vaccination (OR 1.20, 95%CI 1.03–1.39)<br>• No difference in seroprotection in subjects who completed HBV before IBD diagnosis vs. after diagnosis<br>• Among those who received HBV series after IBD diagnosis (n = 25), **no difference in seroprotection in subjects who completed HBV while on IS vs. subjects not on IS at the time of HBV vaccination**

Watts 2017 (USA)	Consecutively recruited IBD patients Administration of HepB vaccination obtained through from medical records or from Immunization Registry Program	Adjusted for age, sex, race, disease phenotype, surgery medications<br><br>Did not adjust for disease activity or duration, nutritional status.	OK	OK	OK	Patients attending a tertiary referral center may differ systematically from other patients	• Cross sectional study<br>• Cohort: 116 patients with IBD aged 5-18 years who had received a full series of HBV vaccine (15 steroids, 66 on IM, 53 on biologic)<br>• Seroprotection defined as anti-HBs ab > 10mIU/mL<br>• **Seroprotection in only 28%**<br>• **Higher seroprotection in younger patients 5-10 year age group vs. older groups** (60% vs. 22-27%, P = 0.04)<br>• **Children younger than 10 were more likely to have seroprotection (OR 4.56, 95% CI 1.08-19.28)**<br>• Cannot differentiate between lack of primary antibody response to HBV vaccine vs. loss of antibody levels with time from vaccination (clinical relevance of waning titer over time is unclear)<br>• **Use of IS was not associated with serological response**
Nguyen 2017 (US)	Chart review	Did not account for concurrent medications, disease activity, duration, phenotype, nutritional status, or other confounding factors	OK	Serological outcomes not available for some patients due to the retrospective nature of the	Unclear	Possible selection bias. Cases were selected from tertiary center. Patients who agreed to or selected to be	• Retrospective cohort study<br>• 51 patients diagnosed with IBD prior to age 10 receiving anti-TNF<br>• 67% (27/44) with documented serology were non-responders to primary HBV vaccination

				study		vaccinated were likely to be prognostically different than those who did not have vaccination.	• 22% (6/27) non-responders received booster<br>• 67% (4/6) seroprotection following booster
Moses 2012 (USA)	Patients were consecutively enrolled from a large US tertiary referral center receiving infliximab	Adjusted for age, sex, disease location, subtype, BMI, albumin, medications.<br><br>Did not adjust for disease activity, duration, or nutritional status	OK	OK	OK	Patients who agreed to vaccination were likely to be prognostically different than patients who did not agree.	• Prospective cross-sectional, single-center study<br>• 100 pediatric IBD patients aged 5-18 on infliximab at a large tertiary pediatric center (53% AZA, 14% 6MP, 35% MTX)<br>• All assessed for serologic markers of HBV: HBsAg, anti-HBc, anti-HBs<br>• Immunity was defined as anti-HBs > 10mIU/mL<br>• Booster dose given to non-immune patients and anamnestic response measured after 4 weeks<br>• **87 patients were vaccinated against HBV and only 56% had immunity to HBV**<br>• 38 patients non-immune after full series of HBV vaccine, **34 received booster immunization and 76% had an anamnestic response**.<br>• Older age, lower albumin levels, and pancolitis were associated with the absence of protective antibodies

							• **IFX dose, frequency, duration, and concurrent use of IM were not associated with the absence of protective antibodies**<br>• Loss of antibody levels with time from vaccination (clinical relevance of waning titer over time is unclear)

6MP – 6 mercaptopurine
AZA - azathioprine
IFX – infliximab
MTX – methotrexate

Case Control Studies						
**Study**	**Cases and controls similar for risk of exposure (or adjusted adequately for confounders)**	**Methods to determine exposure valid and similar for cases and controls**	**Methods to ascertain outcome of interest valid and similar for cases and controls**	**Missing data**	**Other bias**	**Comments**
Urganci 2013 (Turkey)	Accounted for age, sex, BMI, IBD phenotype, treatment<br><br>Did not account for disease activity, duration, or nutritional status	OK	OK	OK	Possible selecton bias. Cases were selected from tertiary research hospital and controls were recruited from hospital clinics.	• Prospective case-control study conducted at pediatric hospital in Turkey.<br>• Cases: 47 patients with IBD ages 3 to 17 years. All on 5ASA, 13 steroid, 8 AZA for steroid dependent IBD<br>• Controls: 50 healthy age- and sex-matched controls recruited from hospital outpatient clinics. Lack of immunity by screening<br>• All received 20mg of HBV vaccine 0, 1, and 6 months<br>• Seroprotection defined as anti-HBs $\geq$ 10 mIU/mL at 1 month<br>• Those with anti-HBs <10 mIU/mL received

						a booster dose.<br>• **Lower seroconversion in IBD patients vs controls after primary vaccination** (70.2% vs. 90%, P = 0.02)<br>• Lower seroconversion in IBD non-responders vs. controls after single booster (60% vs. 50%, ns)<br>• Overall seroconversion in IBD vs. controls (85.1 vs. 96%, ns)<br>• **No significant association between treatment and vaccination response**<br>• No severe adverse reactions or exacerbation of IBD

Evidence Profile Table – Pediatric

HBV vaccine in the Pediatric IBD Population

**Certainty Assessment**								**Summary of Findings**	
Studies	Risk of bias	Inconsistency	Indirectness	Imprecision	Other considerations	Certainty of Evidence	Overall Certainty of evidence	Study Event Rates	Relative Effect (95% CI)
**VPI (Hepatitis B infection) - CRITICAL**							⊕⊕⊕⊖ MODERATE		
1 SR of 4 RCTs[1]<br><br>**Infants born to HBs antigen positive mothers**<br><br>*Adapted from WHO position paper*	Serious[a]	Not serious	Serious[b]	Not serious	None	⊕⊕⊖⊖ LOW		13.1% vs. 50.1%<br><br>(vaccinated vs. controls)	RR 0.28 (0.20-0.40)

2 Observational studies[2,3]<br><br>**General population**<br><br>*Adpated from WHO position paper*	Not serious	Not serious	Serious[b]	Not serious	Upgraded 2 levels due to large effect size	⊕⊕⊕⊖<br>MODERATE		• Vaccination programmes against HBV are very effective, as evident by a dramatic decrease in the incidence of HCC (60.1%), mortality due to fulminant hepatic failure (76.3%), and mortality due to chronic liver diseases (92.0%), as observed among vaccinated persons in Taiwan over the decades since vaccine introduction.
**Immunogenicity (Seroresponse defined as anti-HBs antibody > 10 U/L) - IMPORTANT**								
4 cross-sectional studies[4-7]<br>1 case-control study[8]<br><br>**IBD populations**	Serious[c]	Not serious	Serious[d]	Serious[e]	None	⊕⊖⊖⊖<br>VERY LOW		• Response rates among IBD patients ranged from 28% to 71.3% (cannot differentiate lack of primary antibody response vs. loss of antibody level with times in cross-sectional studies)<br>• No significant association between the use of immunosuppressive treatment and serological response
**Serious adverse effects - CRITICAL**								
1 SR of 4 RCTs[1]<br><br>**Infants born to HBs antigen positive mothers**<br><br>*Adapted from WHO position paper*	Serious[a]	Not serious	Serious[b]	Not serious	None	⊕⊕⊖⊖<br>LOW		• HBV vaccine seems safe, but few trials reported on adverse events
6 RCTs[9-14]<br><br>**Adults in high risk populations**<br><br>Adapted from CDC Grade Evidence Profile	Not serious	Not serious	Serious[b]	Not serious	None	⊕⊕⊕⊖<br>MODERATE		• No serious vaccine-related adverse events (0% in vaccinated)
1 Observational study[15]<br><br>**General population**	Serious[f]	Not serious	Serious[b]	Not serious	None	⊕⊖⊖⊖<br>VERY LOW		• 2588/20,231 (13%) reports were in persons 2-18 years.<br>• 6.8% serious adverse events including 45 deaths. Most commonly reported cause of death was Sudden Infant Death Syndrome.<br>• Most commonly non-death serious reports following HBV vaccines among infants aged < 1 month, were nervous system disorders among children aged 1-23 months; infections and infestation among persons age 2 – 18 years and lymphatic systemic disorders
1 case-control study[8]<br><br>**IBD populations**	Serious[c]	Not serious	Not serious	Serious[g]	None	⊕⊖⊖⊖<br>VERY LOW		• No serious adverse reactions or exacerbation of IBD

Footnotes:

a. Downgraded for study limitations as all studies were unclear risk of bias for allocation concealment. Few trials reported on adverse events.
b. Downgraded for indirectness as these were not IBD patients. Immunogenicity studies suggested that the seroresponse rates in IBD patients may not be as high as in the general population. Sample sizes in IBD studies were not sufficient to detect rare adverse events.

c. Downgraded for study limitations. Possible Selection bias: unclear how patients were selected for most studies. Vaccines may be selectively given to healthier patients (healthy vaccinee effect) or sicker patients (confounding by indication). Most of these studies were conducted in tertiary care centers. Patients who did not complete the vaccination series or refuse to be vaccinated were excluded. This may have led to over-estimation or underestimation of the protective effect of the vaccine. Possible residual confounding factors: did not adjust for disease activity or severity, comorbidities, obesity and nutritional status for most studies.
d. Downgraded for indirectness as surrogate outcomes of immunogenicity (not patient-important outcomes) were used in these studies.
e. Downgraded for imprecision due to small sample size (n = 457 pediatric IBD patients) with very small number of patients on different subgroups of immunosuppressive medications (e.g. anti-TNF, immunomodulator, steroids). If we include the adult IBD populations, we would not need to downgrade for imprecision, but this will not change the overall GRADE rating – very low.
f. Downgraded for study limitations as this is data based on a national spontaneous reporting system (VAERS). May overestimate or underestimate reporting of adverse events.
g. Downgraded for imprecision due to small sample size (n = 47 pediatric IBD patients). If we include the adult IBD populations, we would not need to downgrade for imprecision, but this will not change the overall GRADE rating – very low.

Revaccination following primary vaccination failure in the Pediatric IBD Population

**Certainty Assessment**								**Summary of Findings**	
Studies	Risk of bias	Inconsistency	Indirectness	Imprecision	Other considerations	Certainty of Evidence	Overall Certainty of evidence	Study Event Rates	Relative Effect (95% CI)
**Immunogenicity (Seroresponse defined as anti-HBs antibody > 10 U/L) - IMPORTANT**							⊕⊖⊖⊖ VERY LOW		
3 Observational studies[6-8]<br>**IBD populations**	Serious[a]	Not serious	Serious[b]	Serious[c]	None	⊕⊖⊖⊖ VERY LOW		• See **Response rates after revaccination following primary HBV vaccination failure**<br>• Response rate of revaccination by single booster dose in primary HBV vaccination non-responders is about 50-76%	
**Serious adverse effects - CRITICAL**									
1 case-control study[8]<br>**IBD populations**	Serious[a]	Not serious	Not serious	Serious[c]	None	⊕⊖⊖⊖ VERY LOW		• **No serious vaccine-related adverse events**	

Footnotes:

a. Downgraded for study limitations. Possible Selection bias: only a proportion of patients who failed primary vaccination were revaccinated. Unclear how these patients were selected. Residual confounding factors: did not adjust or account for disease activity or severity, nutritional status, medications use etc.
b. Downgraded for indirectness as surrogate outcomes of immunogenicity (not patient-important outcomes) were used in these studies.
c. Downgraded for imprecision due to small sample sizes.

Response rates after revaccination following primary HBV vaccination failure

	Number of patients (n)	Mean age (years)	Use of IS	Definition of response	Vaccine dose	Response after 1st vaccination	Response after 2nd vaccination	Cumulative response after 1st and 2nd vaccination
Nguyen 2013	51	< 10	100% anti-TNF	Not defined	"primary vaccination series"<br><br>"booster vaccine"	33%<br>(? Lack of primary antibody response vs. loss of protective antibody level over time)	67%	?
Urganci 2013	47	11.6 +/- 3.74	100% 5ASA<br>28% steroids<br>17% AZA	Anti-HBs > 10 IU/L	First: 20mcg 0, 1, 3-6 mos<br><br>Second: single booster dose	70.2%	50%	85.1%
Moses 2012	100	17.9 +/- 4.0	53% AZA<br>14% 6MP<br>36% MTX<br>100% IFX	Anti-HBs > 10 IU/L	First: "full series of HBV vaccine with 3 or more doses"<br><br>Second: single booster dose	53%<br><br>(? Lack of primary antibody response vs. loss of protective antibody level over time)	76%	86%

6MP – 6 mercaptopurine
AZA – azathioprine
IFX – infliximab
MTX - methotrexate

References:

1. Lee C, Gong Y, Brok J, Boxall EH, Gluud C. Hepatitis B immunisation for newborn infants of hepatitis B surface antigen-positive mothers. Cochrane Database Syst Rev. 2006 Apr 19;(2):CD004790. Review.
2. Chien YC, Jan CF, Chiang CJ, Kuo HS, You SL, Chen CJ. Incomplete hepatitis B immunization, maternal carrier status, and increased risk of liver diseases: a 20-year cohort study of 3.8 million vaccinees. Hepatology. 2014 Jul;60(1):125-32.
3. Chiang CJ, Yang YW, You SL, Lai MS, Chen CJ. Thirty-year outcomes of the national hepatitis B immunization program in Taiwan. JAMA. 2013 Sep 4;310(9):974-6.
4. deBruyn JCC, Soon IS, Fonseca K, Feng S, Purtzki M, Goedhart C, Kuhn S, Vanderkooi OG, Wrobel I. Serologic Status of Routine Childhood Vaccines, Cytomegalovirus, and Epstein-Barr Virus in Children With Inflammatory Bowel Disease. Inflamm Bowel Dis. 2019 Jun 18;25(7):1218-1226.
5. Watts A, Bennett WE, Molleston JP, Gupta SK, Croffie JM, Waseem S, McFerron BA, Steiner SJ, Kumar S, Vanderpool CP, Hon EC, Bozic MA, Subbarao GC, Pfefferkorn MD. Incidence of Low Seroimmunity to Hepatitis B Virus in Children With Inflammatory Bowel Disease. J Pediatr Gastroenterol Nutr. 2017 Nov;65(5):551-554.
6. Nguyen HT, Minar P, Jackson K, Fulkerson PC. Vaccinations in immunosuppressive-dependent pediatric inflammatory bowel disease. World J Gastroenterol. 2017 Nov 14;23(42):7644-7652.
7. Moses J, Alkhouri N, Shannon A, Raig K, Lopez R, Danziger-Isakov L, Feldstein AE, Zein NN, Wyllie R, Carter-Kent C. Hepatitis B immunity and response to booster vaccination in children with inflammatory bowel disease treated with infliximab. Am J Gastroenterol. 2012 Jan;107(1):133-8.
8. Urganci N, Kalyoncu D. Immunogenecity of hepatitis A and B vaccination in pediatric patients with inflammatory bowel disease. J Pediatr Gastroenterol Nutr. 2013 Apr;56(4):412-15.
9. Coutinho RA, Lelie PN, Albrecht-van Lent P, Stoutjesdijk L, Huisman J, Kuipers H, Schut LJ, Reerink-Brongers EE, Reesink HW, van Aken WG. Efficacy trial of heat-inactivated hepatitis B vaccine (CLB) in male homosexuals in the Netherlands. Dev Biol Stand. 1983;54:287-92.
10. Crosnier J, Jungers P, Courroucé AM, Laplanche A, Benhamou E, Degos F, Lacour B, Prunet P, Cerisier Y, Guesry P. Randomised placebo-controlled trial of hepatitis B surface antigen vaccine in french haemodialysis units: II, Haemodialysis patients. Lancet. 1981 Apr 11;1(8224):797-800.
11. Szmuness W, Stevens CE, Harley EJ, Zang EA, Alter HJ, Taylor PE, DeVera A, Chen GT, Kellner A. Szmuness W, Stevens CE, Harley EJ, Zang EA, Alter HJ, Taylor PE, DeVera A, Chen GT, Kellner A. N Engl J Med. 1982 Dec 9;307(24):1481-6.
12. Francis DP, Hadler SC, Thompson SE, Maynard JE, Ostrow DG, Altman N, Braff EH, O'Malley P, Hawkins D, Judson FN, Penley K, Nylund T, Christie G, Meyers F, Moore JN Jr, Gardner A, Doto IL, Miller JH, Reynolds GH, Murphy BL, Schable CA, Clark BT, Curran JW, Redeker AG. The prevention of hepatitis B with vaccine. Report of the centers for disease control multi-center efficacy trial among homosexual men. Ann Intern Med. 1982 Sep;97(3):362-6.
13. Szmuness W, Stevens CE, Harley EJ, Zang EA, Oleszko WR, William DC, Sadovsky R, Morrison JM, Kellner A. Hepatitis B vaccine: demonstration of efficacy in a controlled clinical trial in a high-risk population in the United States. N Engl J Med. 1980 Oct 9;303(15):833-41.
14. Dienstag JL, Werner BG, Polk BF, Snydman DR, Craven DE, Platt R, Crumpacker CS, Ouellet-Hellstrom R, Grady GF. Hepatitis B vaccine in health care personnel: safety, immunogenicity, and indicators of efficacy. Ann Intern Med. 1984 Jul;101(1):34-40.
15. Haber P, Moro PL, Ng C, Lewis PW, Hibbs B, Schillie SF, Nelson NP, Li R, Stewart B, Cano MV. Safety of currently licensed hepatitis B surface antigen vaccines in the United States, Vaccine Adverse Event Reporting System (VAERS), 2005-2015. Vaccine. 2018 Jan 25;36(4):559-564.

Evidence to Decision Table – Pediatric

PICO 11	**In pediatric patients with IBD, should vaccination vs. no vaccination against hepatitis B be given?**
**Population**	Pediatric patients with IBD
**Intervention**	Vaccination against hepatitis B
**Comparator**	No vaccination against hepatitis B
**Outcome**	Mortality, VPI (hepatitis B infection), SAEs, Immunogenicity

	Judgement	Research evidence	Additional considerations
**Desirable Effects**	**How substantial are the desirable anticipated effects?**<br><br>o Trivial<br>o Small<br>o Moderate<br>o Large<br><br>o Varies<br>o Don't know	**Risks of Hepatitis B infection in IBD Population**<br><br>Literature search did not identify any study on the risk of hepatitis B infection in pediatric IBD patients.<br><br>**Effectiveness and safety of HBV vaccine in pediatric IBD patients**<br><br>There were no RCTs or observational studies comparing Hep B vaccination versus placebo or no vaccination in pediatric patients with IBD to address this PICO question.<br><br>One systematic review of 4 RCTs assessed the effectiveness and safety of HBV vaccination in infants born to hepatitis B surface antigen-positive mothers.[1] Compared with placebo or no intervention, vaccine reduced hepatitis B occurrence (RR 0.28, 95% CI 0.20-0.40). Most trials were unclear risk of bias for allocation concealment.[1] The evidence was graded as high for effectiveness, but downgraded 1 level due to study limitations. Vaccination programmes against hepatitis B was shown to be very effective in 2 large observational studies with long follow-up, as evident by a dramatic decrease in the incidence of hepatocellular carcinoma (60.1%), mortality due to fulminant hepatic failure (76.3%), and mortality due to chronic liver diseases (92.0%), as observed among vaccinated persons in Taiwan over the decades since vaccine introduction.[2,3] The evidence started as low, but upgraded 2 levels due to large effect size.[2,3]<br><br>Four cross-sectional studies and one case-control study assessed seroprotection (defined as anti-HBs > 10 IU/L) in children with IBD.[4-8] Among the cross-sectional studies, the seroconversion rates against HBV vaccines ranged from 28% to 71.3% in pediatric IBD patients.[4-7] **Due to the cross-sectional nature of these studies, they cannot differentiate between lack of primary antibody response to HBV vaccine vs. loss of antibody levels with time from vaccination**. Yet, the clinical significance of loss of anti-HBs titers in patients with IBD is unknown. Anti-HBs titers frequently become undetectable over time in healthy persons. A number of long-term studies performed in different epidemiological contexts have confirmed that clinical HBV infection rarely	
**Undesirable Effects**	**How substantial are the undesirable anticipated effects?**<br><br>o Large<br>o Moderate<br>o Small<br>o Trivial<br><br>o Varies<br>o Don't know		

occurs among successfully vaccinated people, even though anti-HBs titers decline to < 10 IU/L. Therefore, protection against breakthrough HBV infection may be dependent on immunologic memory rather than on anti-HBs levels. However, clinically significant HBV infection has been documented in immunocompromised responders (HIV and those undergoing hemodialysis) who do not maintain anti-HBs concentration > 10 IU/L. Therefore, the CDC recommends annual anti-HBs testing for these patients and a booster dose be administered when anti-HBs levels decrease to < 10 IU/L. However, for other immunocompromised patients (e.g. IBD), the need for booster is uncertain (risks for contracting HBV may not be as high as patients with HIV or on hemodialysis; no study or report on the risks of breakthrough HBV infection in previously vaccinated IBD patients with low anti-HBs level). As well, there were different proportions of pateints on different types of immunosuppressive medications across studies. Three cross-sectional studies reported no significant association between the use of immunosuppressive medications and the seroconversion rates among pediatric IBD patients, whereas one did not assess this association.[4-7] One case-control study found lower seroconversion rates in children with IBD vs. healthy control after primary vaccination against HBV (70.2% vs. 90%, P = 0.02).[8] The overall seroprotection rates after administering single dose booster to non-responders were not statistically different between the 2 groups (85.1% in IBD patients vs. 96% healthy controls, P = 0.08).[8] No significant association was found between treatment and vaccination response.[8] No serious adverse reactions or exacerbation of IBD was reported.[8]

The evidence suggests that HBV vaccine can induce seroconversion or seroresponse in a significant proportion of pediatric IBD patients (although the response appears to be reduced compared to the general population). Use of immunosuppressive medications may not affect the immunologic response to HBV vaccination in pediatric IBD patients. The seroconversion rates may wane over time with reduction in anti-HBs titer. However, it is uncertain if this reduced immunologic response is clinically relevant/important and would still afford clinical protection as no studies have assessed patient-important clinical outcomes. No serious adverse events including disease exacerbation was reported. The GRADE rating started at low due to the observational designs of the studies. The rating was downgraded to very low due to study limitations (selection bias, residual confounding), indirectness (use of surrogate outcomes), and imprecision. **The evidence for effectiveness was anchored to the general population (high certainty), and was downgraded to <u>moderate</u> due to indirectness as immunogenicity studies suggested that HBV vaccine may be less immunogenic (and therefore less effective) in pediatric IBD patients.**

In terms of safety, the CDC assessed the evidence of HBV vaccine in adult persons with diabetes, although most of the included trials were high risk adults with no history of diabetes.[9-14] No serious adverse events were reported by any of the included trials.[9-14] CDC rated the certainty of evidence as high for safety.[9-14] The evidence was downgraded to moderate due to indirectness (adult vs. pediatric population; sample sizes in IBD studies were not sufficient to detect rare adverse events).[9-14] The systematic review that included infants born to hepatitis B surface antigen-positive mothers showed HBV vaccine to be safe, but few trials reported on serious adverse events.[1] The evidence was downgraded to low due to study limitations and indirectness (non-IBD

		population).[1] The Vaccine Adverse Event Reporting System (VAERS) received 2588/20,231 reports following HBV vaccination in persons 2-18 years from 2005-2015.[15] 6.8% of the reports were serious, including 45 deaths.[15] Most commonly reported case of death was Sudden Infant Death Syndrome. Most common non-death serious reports among infants aged < 1 month were nervous system disorders among children aged 1-23 months, and infections among persons age 2- 18 years.[15] No causal link has been established between HBV vaccines and these adverse events.[15] The evidence was downgraded to very low due to study limitations and indirectness (non-IBD population).[15] In the case-control study involving pediatric IBD patients (n = 47), no serious adverse events were reported after HBV vaccination.[8] The evidence was downgraded to very low due to study limitations and imprecision.[8] **The evidence for safety was anchored to the general population, and was downgraded to <u>moderate</u> due to indirectness (sample size in the IBD study was insufficient to detect rare adverse events).**<br><br>**Overall, there is <u>moderate</u> certainty evidence that HBV vaccine is safe and effective in pediatric IBD populations.**	
**Certainty of evidence**	**What is the overall certainty of the evidence of effects?**<br><br>○ Very low<br>○ Low<br>○ Moderate<br>○ High<br><br>○ No included studies		
**Values and Preferences**	**Is there important uncertainty about or variability in how much people value the main outcomes?**<br><br>○ Important uncertainty or variability<br>○ Possibly important uncertainty or variability<br>○ Probably no important uncertainty or variability<br>○ No important uncertainty or variability	Patients likely value patient-important outcomes (mortality, chronic active hepatitis, cirrhosis, hepatocellular cancer, adverse effects) more than surrogate outcomes (immunogenicity).	

**Balance of effects**	**Does the balance between desirable and undesirable effects favor the intervention or the comparison?**<br><br>o Favors the comparison<br>o Probably favors the comparison<br>o Does not favor either the intervention or the comparison<br>o Probably favors the intervention<br>o Favors the intervention<br><br>o Varies<br>o Don't know		
**Resources required**	**How large are the resource requirements (costs)?**<br><br>o Large costs<br>o Moderate costs<br>o Negligible costs and savings<br>o Moderate savings<br>o Large savings<br><br>o Varies<br>o Don't know	CDC vaccine price list last reviewed/updated: July 1, 2019<br><br>(see price table below)<br><br>Engerix-Bis given as a 3-dose vaccine typically at 0, 1, 6 months.	
**Certainty of Evidence of Required Resources**	**What is the certainty of the evidence of resource requirements (costs)?**<br><br>o Very low<br>o Low<br>o Moderate<br>o High<br><br>o No included studies	The costs of delivering routine immunization services may vary widely across countries and different health system settings. See Immunization Costing Action Network (ICAN) Immunization Delivery Cost Catalogue. http://immunizationeconomics.org/ican-idcc.	

Brandname	CDC cost/dose	Private sector cost/dose
Engerix-B (adult)	$29.73	$58.95
Engerix B (ped/adolescent)	$16.02	$23.72

**Cost effectiveness**	**Does the cost-effectiveness of the intervention favor the intervention or the comparison?**<br><br>○ Favors the comparison<br>○ Probably favors the comparison<br>○ Does not favor either the intervention or the comparison<br>○ Probably favors the intervention<br>○ Favors the intervention<br><br>○ Varies<br>○ No included studies	There are no studies that addressed this question specifically in the IBD population.<br><br>A cost-effectiveness study of HBV vaccination strategies found that vaccination (with or without screening) prevents more disease at somewhat increased cost than no vaccination for the neonatal, adolescent and adult populations.[16] Vaccination (with or without screening) is a dominant strategy in adult high-risk populations (those with HBV incidence > 5%; lower cost and greater benefit than no vaccination).[16] When HBV vaccine is administered to all children at age 10 and again 10 years later (incremental cost-per-year-of-life-saved relative to the "no vaccination" strategy is $375).[16] A strategy of universal newborn vaccination alone leads to an incremental cost-per-year-of-life saved of $3332.[16] If adolescents are vaccinated at age 10, incremental cost-per-year-of-life saved is $13,938; for the general adult population, the incremental cost-per-year-of-life saved of universal vaccination is $54,524.[16]	
**Acceptability**	**Is the intervention acceptable to key stakeholders?**<br><br>○ No<br>○ Probably no<br>○ Probably yes<br>○ Yes<br><br>○ Varies<br>○ Don't know	There are no studies that addressed this question specifically in the IBD population.<br><br>In a qualitative study using semi-structured focus group discussions conducted in the UK, the majority of students aged 12-13 years (n = 50) and nearly all parents (n = 39) were in favor of universal HBV vaccination.[17]	
**Feasibility**	**Is the intervention feasible to implement?**<br><br>○ No<br>○ Probably no<br>○ Probably yes<br>○ Yes<br><br>○ Varies<br>○ Don't know		

References:

1. Lee C, Gong Y, Brok J, Boxall EH, Gluud C. Hepatitis B immunisation for newborn infants of hepatitis B surface antigen-positive mothers. Cochrane Database Syst Rev. 2006 Apr 19;(2):CD004790. Review.
2. Chien YC, Jan CF, Chiang CJ, Kuo HS, You SL, Chen CJ. Incomplete hepatitis B immunization, maternal carrier status, and increased risk of liver diseases: a 20-year cohort study of 3.8 million vaccinees. Hepatology. 2014 Jul;60(1):125-32.

3. Chiang CJ, Yang YW, You SL, Lai MS, Chen CJ. Thirty-year outcomes of the national hepatitis B immunization program in Taiwan. JAMA. 2013 Sep 4;310(9):974-6.
4. deBruyn JCC, Soon IS, Fonseca K, Feng S, Purtzki M, Goedhart C, Kuhn S, Vanderkooi OG, Wrobel I. Serologic Status of Routine Childhood Vaccines, Cytomegalovirus, and Epstein-Barr Virus in Children With Inflammatory Bowel Disease. Inflamm Bowel Dis. 2019 Jun 18;25(7):1218-1226.
5. Watts A, Bennett WE, Molleston JP, Gupta SK, Croffie JM, Waseem S, McFerron BA, Steiner SJ, Kumar S, Vanderpool CP, Hon EC, Bozic MA, Subbarao GC, Pfefferkorn MD. Incidence of Low Seroimmunity to Hepatitis B Virus in Children With Inflammatory Bowel Disease. J Pediatr Gastroenterol Nutr. 2017 Nov;65(5):551-554.
6. Nguyen HT, Minar P, Jackson K, Fulkerson PC. Vaccinations in immunosuppressive-dependent pediatric inflammatory bowel disease. World J Gastroenterol. 2017 Nov 14;23(42):7644-7652.
7. Moses J, Alkhouri N, Shannon A, Raig K, Lopez R, Danziger-Isakov L, Feldstein AE, Zein NN, Wyllie R, Carter-Kent C. Hepatitis B immunity and response to booster vaccination in children with inflammatory bowel disease treated with infliximab. Am J Gastroenterol. 2012 Jan;107(1):133-8.
8. Urganci N, Kalyoncu D. Immunogenecity of hepatitis A and B vaccination in pediatric patients with inflammatory bowel disease. J Pediatr Gastroenterol Nutr. 2013 Apr;56(4):412-15.
9. Coutinho RA, Lelie PN, Albrecht-van Lent P, Stoutjesdijk L, Huisman J, Kuipers H, Schut LJ, Reerink-Brongers EE, Reesink HW, van Aken WG. Efficacy trial of heat-inactivated hepatitis B vaccine (CLB) in male homosexuals in the Netherlands. Dev Biol Stand. 1983;54:287-92.
10. Crosnier J, Jungers P, Courroucé AM, Laplanche A, Benhamou E, Degos F, Lacour B, Prunet P, Cerisier Y, Guesry P. Randomised placebo-controlled trial of hepatitis B surface antigen vaccine in french haemodialysis units: II, Haemodialysis patients. Lancet. 1981 Apr 11;1(8224):797-800.
11. Szmuness W, Stevens CE, Harley EJ, Zang EA, Alter HJ, Taylor PE, DeVera A, Chen GT, Kellner A. Szmuness W, Stevens CE, Harley EJ, Zang EA, Alter HJ, Taylor PE, DeVera A, Chen GT, Kellner A. N Engl J Med. 1982 Dec 9;307(24):1481-6.
12. Francis DP, Hadler SC, Thompson SE, Maynard JE, Ostrow DG, Altman N, Braff EH, O'Malley P, Hawkins D, Judson FN, Penley K, Nylund T, Christie G, Meyers F, Moore JN Jr, Gardner A, Doto IL, Miller JH, Reynolds GH, Murphy BL, Schable CA, Clark BT, Curran JW, Redeker AG. The prevention of hepatitis B with vaccine. Report of the centers for disease control multi-center efficacy trial among homosexual men. Ann Intern Med. 1982 Sep;97(3):362-6.
13. Szmuness W, Stevens CE, Harley EJ, Zang EA, Oleszko WR, William DC, Sadovsky R, Morrison JM, Kellner A. Hepatitis B vaccine: demonstration of efficacy in a controlled clinical trial in a high-risk population in the United States. N Engl J Med. 1980 Oct 9;303(15):833-41.
14. Dienstag JL, Werner BG, Polk BF, Snydman DR, Craven DE, Platt R, Crumpacker CS, Ouellet-Hellstrom R, Grady GF. Hepatitis B vaccine in health care personnel: safety, immunogenicity, and indicators of efficacy. Ann Intern Med. 1984 Jul;101(1):34-40.
15. Haber P, Moro PL, Ng C, Lewis PW, Hibbs B, Schillie SF, Nelson NP, Li R, Stewart B, Cano MV. Safety of currently licensed hepatitis B surface antigen vaccines in the United States, Vaccine Adverse Event Reporting System (VAERS), 2005-2015. Vaccine. 2018 Jan 25;36(4):559-564.
16. Bloom BS, Hillman AL, Fendrick AM, Schwartz JS. A reappraisal of hepatitis B virus vaccination strategies using cost-effectiveness analysis. Ann Intern Med. 1993 Feb 15;118(4):298-306.
17. Hinds A, Cameron JC. Acceptability of universal hepatitis B vaccination among school pupils and parents. Commun Dis Public Health. 2004 Dec;7(4):278-82.

Conclusion – Pediatric

PICO 11: In pediatric patients with IBD, should vaccination vs. no vaccination against hepatitis B be given?

Direction – Yes (100%)
Strength – Strong (100%)

Type of recommendation	Strong recommendation against the intervention	Conditional recommendation against the intervention	Conditional recommendation for either the intervention or the comparison	Conditional recommendation for the intervention	Strong recommendation for the intervention
	○	○	○	○	○

**Recommendation**	**Statement 11: In pediatric patients with IBD, we recommend hepatitis B vaccine be given.**
**Justification**	
**Subgroup considerations**	
**Implementation considerations**	
**Monitoring and evaluation**	• Ongoing monitoring of safety and effectiveness of the HBV vaccine in IBD populations is needed.
**Research priorities**	• RCT is needed to determine the effectiveness, safety, and serological response of double dose standard or accelerated schedule vs. standard dose standard schedule in IBD patients<br>• Research is needed to determine the clinical relevance/importance of waning anti-HBs antibody titer in IBD patients (especially those who are immunocompromised), and the benefits vs risks of periodic monitoring of titers and administration of booster dose.

Summary – Adults

PICO 12A	**In unimmunized adult patients with IBD (with a risk factor for hepatitis B infection), should vaccination vs. no vaccination against hepatitis B be given?**
**Population**	Adult patients with IBD with documented or presumed lack of immunity against hepatitis B (with a risk factor for hepatitis B infection)
**Intervention**	Vaccination against hepatitis B
**Comparator**	No vaccination against hepatitis B
**Outcome**	Mortality, VPI (hepatitis B infection), SAEs, Immunogenicity

PICO 12B	**In unimmunized adult patients with IBD (without a risk factor for hepatitis B infection), should vaccination vs. no vaccination against hepatitis B be given?**
**Population**	Adult patients with IBD with documented or presumed lack of immunity against hepatitis B (without a risk factor for hepatitis B infection)
**Intervention**	Vaccination against hepatitis B
**Comparator**	No vaccination against hepatitis B
**Outcome**	Mortality, VPI (hepatitis B infection), SAEs, Immunogenicity

There were no RCTs or observational studies comparing HBV vaccination versus placebo or no vaccination in adult patients with IBD to address this PICO question.

CDC ACIP has assessed the evidence of effectiveness and safety of HBV vaccine among adults with diabetes. They included 6 RCTs which assessed the risk of hepatitis B infection (mostly in high risk non-diabetic adults including health care personnel, homosexuals, and patients on hemodialysis).[1-6] HBV vaccine was found to reduce the risk of hepatitis B infection by 63% (RR 0.37, 95% CI 0.29-0.48, NNT 261). Seroprotection was achieved in 91.6% (95% CI 87.6-94.4%) among vaccinated persons. The evidence was rated as **high** for both effectiveness and safety by the CDC, but downgraded to moderate when applied to persons with diabetes.

One systematic review of 13 observational studies (observational data from 1 RCT, 6 prospective cohort and 6 retrospective cohort studies) assessed the response rate of HBV vaccination in patients with IBD using the surrogate outcome of anti-HBs antibody

threshold > 10 IU/L.[7] Most studies used the standard HBV vaccine dose of 20ug at 0-, 1-, and 6-month schedule. The pooled rate of an immune response among all IBD patients was 61% (95% CI 53-69%).[7] There was considerable heterogeneity (I^2 = 92%) which could not be accounted for by the subgroup analyses based on study design, published state, vaccine dose, or IBD drug use. Younger age and vaccination during remission were identified as positive predictors of a serological response to vaccination.[7] A low response rate was seen amongst IBD patients receiving immunosuppressive therapies (corticosteroids, immunomodulators, anti-TNF). The authors concluded that the lowest response was seen in those receiving anti-TNF therapies. However, the confidence intervals of all subgroups of medications overlap.[7] Hence, all immunosuppressive medications were associated with a reduced serologic response to HBV vaccine in IBD patients. In one large prospective study of 389 IBD patients starting anti-TNF therapy, 254 patients were found to have anti-HBs < 100 IU/L.[12] They were vaccinated with accelerated double 40ug HBV vaccine dose at 0-, 1-, and 2-month schedule. Effective vaccination and seroprotection were achieved in 26.4% and 43.5% of patients, and for revaccination 31.3% and 44.4%, respectively.[12] At the end of the vaccination, a total of 56.7% of patients achieved seroprotection.[12] Age $\leq$ 30 years and the use of anti-TNF monotherapy were the only predictive factors for seroprotection.[12]

The evidence suggests that HBV vaccine can induce seroconversion or seroresponse in a significant proportion of adult IBD patients (although the response appears to be reduced compared to the general population). Young age and vaccination during remission are associated with improved serologic response to HBV vaccination. Use of immunosuppressive medications (e.g. immunomodulators, anti-TNF, steroids) is associated with a reduced immunologic response to HBV vaccination in IBD patients. However, it is uncertain if this reduced immunologic response is clinically relevant/important and would still afford clinical protection as no studies have assessed patient-important clinical outcomes. No serious adverse events including disease exacerbation was reported. The GRADE rating started at low due to the observational designs of the studies. The rating was downgraded to **very low** due to study limitations (selection bias, residual confounding), inconsistency, and indirectness (use of surrogate outcomes).

A standard 3-dose HBV vaccination confers protective antibody formation in more than 95% of healthy infants, children and young adults. According to the WHO, an anti-HBs concentration of > 10 IU/L (seroconversion) measured 1-3 months after administration of the last dose of the primary vaccination is considered a reliable marker of protection against infection. However, **the protective antibody titer induced by vaccination is under debate**. Increasingly, an effective immune response (complete response according to the standard definition of efficacy) is defined as anti-HBs > 100 IU/mL. **Due to lack of studies with clinical outcomes comparing the 2 different definitions of serologic protection, it is therefore uncertain what the correlates of vaccine-induced protection should be in IBD patients.**

One RCT assessed the effects of vedolizumab on the serological response to HBV vaccine in healthy participants (not IBD patients).[8] 127 healthy participants were randomized to receive either a single dose of vedolizumab 750mg IV or placebo. After 4 days, they were given HBV vaccine days 4, 32, and 60. Vedolizumab did not alter the response to HBV vaccination among healthy participants. The response rate defined as anti-HBs antibody $\geq$ 10 U/L was 90.3% in placebo group vs. 88.5% in the vedolizumab group. The GRADE rating started as high, but was downgraded to **very low** due to indirectness (healthy participants, surrogate outcomes) and imprecision. **In summary, there is <u>very low</u> certainty evidence that vedolizumab is not associated with a reduced serologic response to HBV vaccination in patients with IBD.**

The overall evidence was anchored to the general population (individuals with high risk for contracting hepatitis B infection). Although there were studies on safety and immunogenicity on HBV vaccine in adult IBD populations, the evidence suggests that the vaccine may not be as immunogenic (and therefore as effective) in the IBD populations compared to the general population. Therefore, the evidence for effectiveness was downgraded to moderate for HBV vaccine in adult iBD populations. For safety, the sample sizes in the IBD studies were insufficient to detect rare adverse events. Therefore, the evidence for safety was also downgraded to moderate for HBV vaccine in adult IBD populations. **Overall, there is <u>moderate</u> certainty evidence that HBV vaccine is safe and effective in adult IBD patients (with a risk factor for hepatitis B infection). The evidence was down graded to low in adult IBD patients (without a risk factor for hepatitis B infection) due to indirectness (studies in the general population were done in individuals at high risk for hepatitis B infection).**

Revaccination following primary vaccination failure

Four observational studies all conducted in Spain assessed revaccination with repeat vaccination series of 3 additional doses of HBV (20mcg or 40mcg).[9-12] The response rate of revaccination by repeat 3-dose vaccination series is about 50% (range 42-68%).[9-12] The GRADE rating started at low due to the observational nature of these studies. The rating was downgraded to very low due to study limitations, indirectness (surrogate outcomes) and imprecision. **In summary, there is <u>very low</u> certainty evidence that repeat vaccination 3-dose series is safe and effective in reducing the risks of HBV infection in IBD patients following primary vaccination failure.**

There is one retrospective cohort study by Pratt et al. that was published outside the literature search comparing 3 vs. 1 or 2 additional HBV doses following primary vaccination failure in adult patients with IBD.[13] This study cannot be included in the evidentiary base as it is outside our search date. The study showed that in immunocompromised patients with IBD who failed primary HBV vaccination, 3 additional doses of vaccine were more likely to achieve seroprotective HBsAb levels than patients who received 1 or 2 doses (62.9% vs. 40.2%; OR 1.77, P = 0.01; OR 1.9, P = 0.03, respectively, after adjusting for age, sex, race,

immunosuppressive medication exposure, time between vaccine/titer).[13] Due to the retrospective nature of this study, it cannot reliably distinguish between primary HBV vaccination non-responders and initial responders with waning antibody titers but ability to mount an anamnestic response once re-challenged with a booster vaccination.

Among immunocompetent patients who do not respond to an initial 3-dose HBV vaccination schedule, meta-analyses have reported that between 25-50% will respond to an additional booster dose, while between 44-100% will respond to a repeat 3-dose vaccine series.[14]

Double dose vs. standard dose HBV vaccination in IBD patients
PICO 8C: In unimmunized adult patients with IBD, should double dose vs. standard dose of HBV vaccination be given?
Two observational studies (1 conducted in Spain and 1 in Turkey) compared double dose vs. standard dose HBV vaccination.[15,16] One study included patients with a variety of autoimmune conditions (15 patients with IBD).[16] The other study included only IBD patients.[15] Two cohort studies conducted in Spain assessed serological response to double dose HBV vaccination in IBD patients without a comparison group.[11,12] There was inconsistency in the results with one study suggesting no difference in serological response between double dose vs. standard dose HBV vaccination administered as per standard schedule in patients with autoimmune conditions, and the other study suggesting higher serological response with accelerated schedule of double dose HBV vaccination in IBD patients.[15,16] Nevertheless, both cohort studies with no comparison group suggested that the serological response was still low with accelerated schedule of double dose HBV vaccination in IBD patients.[11,12] The GRADE rating started at low due to the observational nature of these studies. The rating was downgraded to very low due to study limitations, indirectness (surrogate outcomes) and imprecision. **In summary, there is <u>very low</u> certainty evidence that double dose HBV vaccination is associated with a higher or comparable serological response as standard dose HBV vaccination in IBD patients.**

Check titers periodically and administer booster doses as required in IBD patients who responded to HBV vaccination?
There is no RCT or observational studies that addressed this question (comparing measuring vs. not measuring anti-HBs titer periodically and giving vs. not giving booster doses when anti-HBs titer is low).

One prospective observational study included 100 IBD patients who responded to HBV vaccination (anti-HBs > 10 IU/L at 1 – 3 months). The anti-HBs titers were measured at 6 and 12 months.[17] The cumulative incidence of loss of anti-HBs titer was 2% after 6 months and 15% at 12 months.[17] The incidence rate of loss of protective anti-HBs titers was 18% per patient-year. Treatment with anti-TNFs was associated with a higher risk of loss of anti-HBs (HR 3.1, 95% CI 1.1-8.8).[17] In another prospective observational study that included 99 IBD patients starting ani-TNF with previous effective vaccination (anti-HBs > 100 IU/L), 90% maintained titers 4 months after the beginning of anti-TNF treatment, and 81% of patients maintained the titers after a mean follow-up of 29 months.[12]

Yet, the clinical significance of loss of anti-HBs titers in patients with IBD is unknown. Anti-HBs titers frequently become undetectable over time in healthy persons. A number of long-term studies performed in different epidemiological contexts have confirmed that clinical HBV infection rarely occurs among successfully vaccinated people, even though anti-HBs titers decline to < 10 IU/L. Therefore, protection against breakthrough HBV infection may be dependent on immunologic memory rather than on anti-HBs levels. However, clinically significant HBV infection has been documented in immunocompromised responders (HIV and those underlying hemodialysis) who do not maintain anti-HBs concentration > 10 IU/L**.**[18] Therefore, the CDC recommends annual anti-HBs testing for these patients and a booster dose be administered when anti-HBs levels decrease to < 10 IU/L. However, for other immunocompromised patients (e.g. IBD), the need for booster is uncertain (risks for contracting HBV may not be as high as patients with HIV or on hemodialysis; no study or report on the risks of breakthrough HBV infection in previously vaccinated IBD patients). The GRADE rating started at low due to the observational design of this study.[17] The rating was downgraded to **very low** due to study limitations (residual confounding, selection bias), indirectness (surrogate outcomes), and imprecision.[17]

In summary, there is <u>very low</u> certainty evidence that IBD patients have loss of protective anti-HBs titers over time. Yet, the potential benefits and harms of periodically measuring anti-HBs titers and giving booster when titer is low are highly uncertain. In particular, there are no studies with long term follow-up assessing the safety and effectiveness of repeated administration of booster in patients with autoimmune diseases. It is also uncertain what the target anti-HBs titers should be for IBD patients especially for those who are on immunosuppressive therapies.

References:

1. Coutinho RA, Lelie PN, Albrecht-van Lent P, Stoutjesdijk L, Huisman J, Kuipers H, Schut LJ, Reerink-Brongers EE, Reesink HW, van Aken WG. Efficacy trial of heat-inactivated hepatitis B vaccine (CLB) in male homosexuals in the Netherlands. Dev Biol Stand. 1983;54:287-92.
2. Crosnier J, Jungers P, Couroucé AM, Laplanche A, Benhamou E, Degos F, Lacour B, Prunet P, Cerisier Y, Guesry P. Randomised placebo-controlled trial of hepatitis B surface antigen vaccine in french haemodialysis units: II, Haemodialysis patients. Lancet. 1981 Apr 11;1(8224):797-800.
3. Szmuness W, Stevens CE, Harley EJ, Zang EA, Alter HJ, Taylor PE, DeVera A, Chen GT, Kellner A. Szmuness W, Stevens CE, Harley EJ, Zang EA, Alter HJ, Taylor PE, DeVera A, Chen GT, Kellner A. N Engl J Med. 1982 Dec 9;307(24):1481-6.
4. Francis DP, Hadler SC, Thompson SE, Maynard JE, Ostrow DG, Altman N, Braff EH, O'Malley P, Hawkins D, Judson FN, Penley K, Nylund T, Christie G, Meyers F, Moore JN Jr, Gardner A, Doto IL, Miller JH, Reynolds GH, Murphy BL, Schable CA, Clark BT, Curran JW, Redeker AG. The prevention of hepatitis B with vaccine. Report of the centers for disease control multi-center efficacy trial among homosexual men. Ann Intern Med. 1982 Sep;97(3):362-6.
5. Szmuness W, Stevens CE, Harley EJ, Zang EA, Oleszko WR, William DC, Sadovsky R, Morrison JM, Kellner A. Hepatitis B vaccine: demonstration of efficacy in a controlled clinical trial in a high-risk population in the United States. N Engl J Med. 1980 Oct 9;303(15):833-41.
6. Dienstag JL, Werner BG, Polk BF, Snydman DR, Craven DE, Platt R, Crumpacker CS, Ouellet-Hellstrom R, Grady GF. Hepatitis B vaccine in health care personnel: safety, immunogenicity, and indicators of efficacy. Ann Intern Med. 1984 Jul;101(1):34-40.

7. Jiang H, Wang S, Deng M et al. Immune response to hepatitis B vaccination among people with inflammatory bowel diseases: A systematic review and meta-analysis. *Vaccine.* 2017, 35: 2633-2641.
8. Wyant T, Leach T, Sankoh S, et al. Vedolizumab affects antibody responses to immunisation selectively in the gastrointestinal tract: randomised controlled trial results *Gut*. 2015;64:77–83
9. Cossio-Gil Y, Martínez-Gómez X, Campins-Martí M, Rodrigo-Pendás JÁ, Borruel-Sainz N, Rodríguez-Frías F, Casellas-Jordà F. Immunogenicity of hepatitis B vaccine in patients with inflammatory bowel disease and the benefits of revaccination. J Gastroenterol Hepatol. 2015 Jan;30(1):92-8.
10. Sempere L, Almenta I, Barrenengoa J, Gutiérrez A, Villanueva CO, de-Madaria E, García V, Sánchez-Payá J. Factors predicting response to hepatitis B vaccination in patients with inflammatory bowel disease. Vaccine. 2013 Jun 26;31(30):3065-71.
11. Gisbert JP, Villagrasa JR, Rodríguez-Nogueiras A, Chaparro M. Efficacy of hepatitis B vaccination and revaccination and factors impacting on response in patients with inflammatory bowel disease. Am J Gastroenterol. 2012 Oct;107(10):1460-6.
12. Loras C, Gisbert JP, Saro MC, Piqueras M, Sánchez-Montes C, Barrio J, Ordás I, Montserrat A, Ferreiro R, Zabana Y, Chaparro M, Fernández-Bañares F, Esteve M; REPENTINA study, GETECCU group (Grupo Español de trabajo de Enfermedades de Crohn y Colitis Ulcerosa). Impact of surveillance of hepatitis b and hepatitis c in patients with inflammatory bowel disease under anti-TNF therapies: multicenter prospective observational study (REPENTINA 3). J Crohns Colitis. 2014 Nov;8(11):1529-38.
13. Pratt PK Jr, Nunes D, Long MT, Farraye FA. Improved Antibody Response to Three Additional Hepatitis B Vaccine Doses Following Primary Vaccination Failure in Patients with Inflammatory Bowel Disease. Dig Dis Sci. 2019 Jul;64(7):2031-2038.
14. Schillie S, Vellozzi C, Reingold A, Harris A, Haber P, Ward JW, Nelson NP. Prevention of Hepatitis B Virus Infection in the United States: Recommendations of the Advisory Committee on Immunization Practices. MMWR Recomm Rep. 2018 Jan 12;67(1):1-31.
15. Gisbert JP, Menchén L, García-Sánchez V, Marín I, Villagrasa JR, Chaparro M. Comparison of the effectiveness of two protocols for vaccination (standard and double dosage) against hepatitis B virus in patients with inflammatory bowel disease. Aliment Pharmacol Ther. 2012 Jun;35(12):1379-85.
16. Haykir Solay A, Eser F. High dose hepatitis B vaccine is not effective in patients using immunomodulatory drugs: a pilot study. Hum Vaccin Immunother. 2019;15(5):1177-1182.
17. Gisbert JP, Villagrasa JR, Rodríguez-Nogueiras A, Chaparro M. Kinetics of anti-hepatitis B surface antigen titers after hepatitis B vaccination in patients with inflammatory bowel disease. Inflamm Bowel Dis. 2013 Mar;19(3):554-8.
18. Stevens CE, Alter HJ, Taylor PE, Zang EA, Harley EJ, Szmuness W. Hepatitis B vaccine in patients receiving hemodialysis. Immunogenicity and efficacy. N Engl J Med. 1984 Aug 23;311(8):496-501.

Risk of Bias Table – Adults

**SR of Observational Studies and RCTs**		
**Study**	**Quality Assessment**	**Comments**
Yanny 2019 (select	• No risk of bias assessment was provided<br>• **This does not appear to be a systematic review.**	• SR of 71 studies defined as "adequate quality" with a total of 2354 patients. Examined management approaches to HBV vaccination non-response in select populations

populations with decreased response to HBV vaccine including IBD and celiac disease)	• **Errors in identifying studies that assessed celiac disease and IBD when references were checked:** included Jiang 2017 (SR that did not address this question), Marin 2015 (review article), Chyuan 2015 (mouse model), Filippelli 2014 (review article), Lin 2008 (not relevant), Walkiewicz-Jedrzejczak 2014 (celiac), Feng 2017 (hemodialysis), Das 2017 (case report), Chen 2014 (not relevant), Feng 2017 (drug users). Sempere 2013 and Gisbert 2012 were the only eligible IBD studies.<br>• **Uncertain how the results were pooled together**	(general population, HIV, HCV, ESRD and dialysis dependence, hypoalbuminemia, diabetes, celiac disease and IBD, advanced age).<br>• HBV vaccine response defined as anti-HBs antibody > 10 IU/L<br>• Seroconversion for general population (10 studies, n = 282) primary non-responders: repeat vaccination series with the same dose vs. a single-dose booster (85.7% vs. 73.2%, p < 0.01)<br>• Seroconversion for celiac disease and IBD primary non-responders (12 studies, n = 282): repeat vaccination series with the same dose of 10ug to 20ug (67.5%)<br>• Seroconversion for general population loss of immunity: repeat vaccination series with the same dose vs. a single-dose booster (95.7% vs. 83.2%, P < 0.01)<br>• Seroconversion for celiac disease and IBD loss of immunity: repeat vaccination series (77.5%)<br>• Single dose booster with the same vaccination dose or a higher dose was not studied in celiac disease or IBD
Jiang 2017 (Adult IBD populations)	• Quality assessment was done with an instrument produced by Udina et al (not valid)<br>• Most studies were at moderate or high risk of bias for most domains<br>• Moderate to high levels of statistical heterogeneity (inconsistency) for most meta-analyses<br>• Downgraded for residual confounding factors including different disease activity states at time of study, comorbidities, smoking, obesity and nutritional status, and selection bias. Most of these studies were conducted in tertiary care centers. Patients who did not complete the vaccination series or refuse to be vaccinated were excluded.<br>• Short duration of follow up for most studies	• SR and MA included 13 studies with 1688 IBD patients (1 RCT treated as observational data, 6 prospective studies and 6 retrospective studies) assessing response rate to HBV vaccination<br>• Anti-HBs level > 10 U/L was considered an effective immune response.<br>• Most studies used 0-, 1- and 6-month schedule. Vaccine dosage 10-40ug<br>• **Pooled response rate among patients with IBD 61% (95% CI 53-69%), range 34.1-78.6%,** significant heterogeneity (I^2 value 92%)<br>• **Young age and vaccination during disease remission (RR 1.62, 95% CI 1.15-2.29) were associated with a positive response to HBV vaccination**<br>• **No use of immunosuppressive therapy was predictive of an immune response** compared to immunosuppressive (RR 1.31, 95% CI 1.13-1.59), immunomodulator (RR 1.33, 95% CI 1.08-1.63) or anti-TNF (RR 1.57, 95% CI 1.19-2.08). CIs of these comparisons all overlap. Therefore, cannot support the authors' conclusion that anti-TNF had a greater negative effective on the immunologic response to HBV vaccination than immunomodulatory therapy.<br>• No comparison of different vaccine dosing<br>• One study (Gisbert 2013) looked at long term follow up and found loss of protective antibodies over time in most IBD patients<br>• **No serious vaccine-related adverse events**

	Cohort studies						
**Study**	**Valid methods to ascertain exposure**	**Prognostic factors (other than exposure of interest) similar among cohorts – or cohorts were adjusted adequately for confounders**	**Demonstration that outcome of interest was not present at the start of the study**	**Outcome detection methods valid and similar among cohorts**	**Follow-up complete and similar among cohorts**	**Free of other bias**	**Comments**
Ardesia 2017 (Italy)	OK<br><br>Serologic status and interview	Did not adjust for duration of disease, medication use, disease activity, smoking, and nutritional status which may be confounding factors	OK	OK	OK	OK	• Retrospective nested case-control study<br>• Cases: 509 IBD patients undergoing baseline infectious screening prior to starting thiopurines or biologics (new and follow up patients). 176 Aged < 37 (patients born later than 1979) – mandatory HBV vaccination in Italy<br>• Controls: 174 healthy non-IBD controls aged $\leq$ 37<br>• Vaccinated and lost protective titer: Patients aged $\leq$ 37 and negative for HBsAg, HBcAb and HBsAb < 10 IU/mL<br>• Vaccinated and positive protective titer: Patients aged $\leq$ 37 with HBsAb > 10 IU/mL<br>• Lower seroprotection rate in IBD compared to controls. HBsAb positivity of 55.9% in IBD patients and of 85.1% in controls. **Significant loss of HBsAb titer among patients with IBD**<br>• No safety concerns
Chang 2018 (Korea)	OK	"Disease type, duration, activity, and type of treatment at the time of vaccination were not significant factors" in immunogenicity	OK	OK	OK	Possible selection bias as uncertain how patients were recruited in the study. Patients who agreed to vaccination were likely to be prognostically different than	• Prospective observational cohort study<br>• 330 patients with IBD were included from a hospital IBD clinic. Ages were 29.9 $\pm$ 12.3 years.<br>• 87 patients did not have immunity (26%). 44 patients with previous complete HBV vaccination received a booster. 29 patients without prior vaccination or an unknown history of vaccination received a full 3-dse vaccination.<br>• Optimal response anti-HBs $\geq$ 10 IU/L $\geq$ 1 mo<br>• Booster group: 70.5% response. Full vaccination after failed booster: optimal response 76.9%

						patients who did not agree.	(10/13)<br>• Full vaccination group: 89.7% response<br>• Younger age at initial vaccination dose (< 26 years) was a positive predictor for optimal vaccine response (OR 6.01, 95% CI 1.15-31.32)<br>• Previous complete vaccination history (OR 0.15, 95% CI 0.03-0.80) was a negative predictive factor for optimal vaccine response<br>• No serious adverse events
Loras 2014 (Spain)	OK	Adjusted for age, sex, previous vaccines, type of treatment, established vaccination schedule, IS, IBD subtype, disease duration.<br><br>Did not adjust for duration of disease, disease activity, smoking, and nutritional status.	OK	OK	OK	Possible selection bias as patients who agreed to vaccinate and revaccinate may be prognostically different than those who refused to or who were not selected to be vaccinated.	• Multicenter prospective study of 389 IBD patients starting IFX therapy (248 IFX, 138 ADA, 3 CZP, 74% on IM) with 4 interventions:<br>• (1) anti-HBs < 100 IU/L (n = 254): accelerated double dose 40ug HBV vaccine at 0-1-2 mos, and revaccinate same schedule if titer < 100 IU/L after 2 mos<br>• (2) anti-HBs > 100 IU/L: monitoring titer at 4 mos after starting anti-TNF and at the end of f/u<br>• (3) anti-HBc and/or HCV+: analysis q 2 mos<br>• (4) HBsAg+: start antivirals<br>• Seroprotection defined as anti-HBs 10-100 IU/L and effective vaccination anti-HBs > 100 IU/L<br>• Non-immune patients: seroprotection (43.5%) and effective vaccination (26.4%) after 1st vaccination. Seroprotection (44.4%) and effective vaccination (31.3%) after revaccination.
Gisbert 2013 (Spain)	OK	Did not adjust for duration of disease, disease activity, smoking, and nutritional status which may be confounding factors	OK	OK	OK	Possible selection bias as uncertain how patients were recruited in the study. Patients who were prospectively followed after	• Prospective cohort study of 100 patients with IBD (49% thiopurines, 14% anti-TNF) with a response (anti-HBS > 10 IU/L at 1-3 mos) to HBV vaccination<br>• Anti-HBs titers measured at 6 and 12 mos<br>• Cumulative incidence of loss of anti-HBs titers was 2% at 6 months and 15% after 12 months<br>• The projected incidence rate of loss of protective anti-HBs titers was 18% per patient-year<br>• Treatment with anti-TNF was the only factor

						vaccination may be different than those who were not followed	associated with a higher risk of loss of anti-HBs (HR 3.1, 95% CI 1.1-8.8).

ADA – adaliumumab
CZP - certolizumab
IFX - infliximab

Before-After (Pre-Post) Studies									
Study	Was there a concurrent comparator group that did not receive the intervention	If a concurrent comparator group was used, was it similar to the intervention group (or adequately adjusted) for prognostic factors	If no concurrent comparator group was used: If each participant served as his/her own control (assessed before vs. after the intervention), are there compelling arguments that the outcome was not influenced by historic events / underlying secular trends	If no concurrent comparator group was used: If two different consecutive cohorts of participants were assessed (before vs. after implementation of the intervention), are there (a) compelling arguments that the outcome was not influenced by historic events / underlying secular trends and (b) evidence that the two groups were similar (or adequately	Outcome detection methods valid and similar among compared groups / periods	Incomplete outcome data assessed	Selective outcome reporting	Other bias	Comments

				adjusted) for prognostic factors					
Haykir Solay 2019 (Turkey)	No – but this does not affect the risk of bias as the only explanation for increase in titer is the vaccine (no other confounding factors)	No adjustment of potential confounding factors such as disease activity	OK	NA	OK	OK	OK	Possible selection bias as unclear how patients were recruited into the study.	• Cohort study assessing pre- and post vaccination titers in 109 HepB seronegative with a variety of autoimmune diseases including 15 patients with IBD (Crohn's 12, UC 3)<br>• Engerix-B was administered on the standard schedule in 3 doses of 20 (73 patients) or 40 µg/ml<br>• Response defined as anti-HBs $\geq$ 10IU/mL<br>• Response rate 49.3% in the standard dose group and 61.1% in the high dose group (ns).<br>• The IM drugs used by the patients were adalimumab (n = 62), ustekinumab (n = 25), infliximab (n = 12), etanercept (n

									= 9) and golimumab (n = 1). The antibody response to vaccination was low in infliximab users (p = 0.007), higher response rates in ustekinumab and etanercept users (p = 0.032 and 0.035 respectively)<br>• There was no statistically significant difference between the standard and high dose vaccine among the drug groups
Cossio-Gil 2015 (Spain)	No – but this does not affect the risk of bias as the only explanation for increase in titer is the vaccine (no other confounding factors)	No	OK	NA	OK	OK	OK	Possible selection bias as only 53/85 patients who did not respond to the first vaccination attempt were revaccinated	• 172 IBD patients vaccine with 10mcg or 20mcg HBV vaccine 0, 1, 3-6 mos<br>• 53/85 patients with anti-HBs < 10 IU/L were revaccinated with HBV same dose schedule

Sempere 2012 (Spain)	No – but this does not affect the risk of bias as the only explanation for increase in titer is the vaccine (no other confounding factors)	No	OK	NA	OK	OK	OK	Possible selection bias as only 44/55 patients who did not respond to the first vaccination attempt were revaccinated.	• 105 IBD patients vaccinated with HBV vaccine 20mg 0, 1, 6 mos<br>• 44/55 patients with anti-HBs < 10 IU/L were revaccinated with 40mg HBV 0, 1, 6 mos
Gisbert 2012a (Spain)	No – but this does not affect the risk of bias as the only explanation for increase in titer is the vaccine (no other confounding factors	No – More patients on standard dose group on IS, anti-TNF, and combined thiopurine and anti-TNF. After adjustment for possible confounding variables, vaccination protocol was the only factor associated with a response to the vaccine.	OK	NA	OK	OK	OK	Possible selection bias as vaccination protocol used is dependent on hospital site.	• Cohort study of 148 IBD patients from 3 hospitals with 2 different vaccination protocols (22% thiopurines, 23% anti-TNF, 25% both)<br>• Standard protocol (Engerix-B single dose 20ug 0, 1 and 6 mos) in 46% of patients<br>• Faster protocol (Engerix-B double dose 40ug 0, 1, and 2 mos) in 54% of patients<br>• Response defined as anti-HBS titer > 10 IU/L at 1-3 mos<br>• Seroconversion was higher with double dose vs.

									standard dose (75% vs. 41%, P< 0.001)<br>• No serious adverse events
Gisbert 2012b (Spain)	No – but this does not affect the risk of bias as the only explanation for increase in titer is the vaccine (no other confounding factors)	No	OK	NA	OK	OK	OK	Possible selection bias as only 95/148 patients who did not respond to the first vaccination attempt were revaccinated.	• Cohort study of 241 IBD patients vaccinated against HBV with accelerated double-dose schedule (Engerix B 40ug 0, 1, 2 mos)<br>• 95/148 patients with anti-HBs < 100 IU/L were revaccinated with the same dose and schedule<br>• Response (anti-HBs>100 IU/L) after second vaccination 42% (29-54%)

RCTs							
**Study**	**Adequate sequence generation**	**Allocation concealment**	**Blinding**	**Incomplete outcome data addressed**	**Free of selective reporting**	**Free of other bias**	**Comments**
Wyant 2015 (USA) Healthy participants	Block randomization using a centralized	OK	OK	OK	OK	Participants were healthy volunteers rather than	• A phase I double blinded, placebo controlled randomized non-inferiority trial was conducted among **healthy** male and female participants aged 18-39 years.

	voice response system.					IBD patients which impacts the generalizability of the immune response effect	• 127 participants were randomized 1:1 to receive a dose of vedolizumab or placebo and were then vaccinated with Hep B (HBvaxPRO; Sanofi Pasteur MSD, Maidenhead, Berkshire, UK) and oral cholera vaccine.<br>• Seroconversion defined as anti-HBs antibody $\geq$ 10 IU/L on day 74.<br>• A total of 56 (90.3%) placebo-treated and 54 (88.5%) vedolizumab-treated participants showed HepBsAb seroconversion (absolute difference, −1.8%; 95% CI −12.7% to 9.1%).<br>• One serious adverse event (spontaneous abortion) in a placebo patient

Evidence Profile Table – Adults

HBV vaccine in the Adult IBD Population

Certainty Assessment								Summary of Findings	
Studies	Risk of bias	Inconsistency	Indirectness	Imprecision	Other considerations	Certainty of Evidence	Overall Certainty of evidence	Study Event Rates	Relative Effect (95% CI)
**VPI (Hepatitis B infection) - CRITICAL**							⊕⊕⊕⊖ MODERATE With risk factor<br><br>⊕⊕⊖⊖ LOW		
6 RCTs[1-6]<br><br>**Adults in high risk populations**<br><br>Adapted from CDC Grade Evidence Profile	Not serious	Not serious	Serious[a] With risk factor<br><br>Very serious[a] Without risk factor	Not serious	None	⊕⊕⊕⊖ MODERATE<br>⊕⊕⊖⊖<br><br>LOW Without risk factor		4.1% vs. 10.7%<br><br>(vaccinated vs. controls)	RR 0.37 (0.29-0.48)<br>NNT 261

**Immunogenicity (Seroresponse defined as anti-HBs antibody > 10 U/L) - IMPORTANT**							Without risk factor	
1 SR Observational studies[7]<br><br>1 prospective cohort study[12]<br><br>**IBD populations**	Serious[b]	Serious[c]	Serious[d]	Not serious	None	⊕⊖⊖⊖<br>VERY LOW		• **Pooled seroresponse rate among IBD patients 61% (95% CI 53-69%)[7]**<br>• Young age and vaccination during disease remission (RR 1.62, 95% CI 1.15-2.29) were associated with a positive response to HBV vaccination[7]<br>• **No use of immunosuppressive therapy was predictive of an immune response compared to immunosuppressive** (RR 1.31, 95% CI 1.13-1.59), **immunomodulator** (RR 1.33, 95% CI 1.08-1.63) or **anti-TNF** (RR 1.57, 95% CI 1.19-2.08).[7]
1 RCT[8]<br><br>**General Population**	Not serious	Not serious	Very serious[e]	Serious[f]	None	⊕⊖⊖⊖<br>VERY LOW		• **Vedolizumab did not alter the serologic response to HBV vaccine.**
**Serious adverse effects - CRITICAL**								
6 RCTs[1-6]<br><br>**Adults in high risk populations**<br><br>Adapted from CDC Grade Evidence Profile	Not serious	Not serious	Serious[a]	Not serious	None	⊕⊕⊕⊖<br>MODERATE		• **No serious vaccine-related adverse events (0% in vaccinated)**
1 SR Observational studies[7]<br><br>**IBD populations**	Serious[b]	Not serious	Not serious	Not serious	None	⊕⊖⊖⊖<br>VERY LOW		• **No serious vaccine-related adverse events**

Footnotes:

a. Downgraded for serious indirectness for IBD patients with risk factors for hepatitis B. Populations were not IBD patients, and included high risk populations such as homosexuals, patients on hemodialysis, health care personnel. Immunogenicity studies in IBD populations suggested that the Hep B vaccine may not be as immunogenic as in the general population. Downgraded for very serious indirectness for IBD patients without risk factor for hepatitis B as risk of hepatitis B would be lower compared to those with risk factors. Therefore, the absolute effects would be expected to be lower from hepatitis B vaccination. Sample sizes in IBD studies were not sufficient to detect rare adverse events: rate of anaphylaxis estimated 1.1 per million doses (95% CI 0.1-3.9) per million doses.

b. Downgraded for study limitations. Possible Selection bias: unclear how patients were selected for most studies. Vaccines may be selectively given to healthier patients (healthy vaccinee effect) or sicker patients (confounding by indication). Most of these studies were conducted in tertiary care centers. Patients who did not complete the vaccination series or refuse to be vaccinated were excluded. This may have led to over-estimation or underestimation of the protective effect of the vaccine. Possible residual confounding factors: did not adjust for disease activity or severity, smoking, comorbidities, obesity and nutritional status for most studies.

c. Downgraded for inconsistency (significant heterogeneity I^2 = 92%)
d. Downgraded for indirectness as surrogate outcomes of immunogenicity (not patient-important outcomes) were used in these studies. All studies were done in Spain. Uncertain if results would be generalizable to IBD populations in other countries.
e. Downgraded for indirectness as the study included healthy participants (not IBD patients). Therefore, their response to HBV after receiving Vedolizumab may not be generalizable to the IBD populations. Use of surrogate outcomes of immunogenicity
f. Downgraded for imprecision due to small sample size (n = 127 with only 64 participants treated with 1 dose of vedolizumab).

Revaccination following primary vaccination failure in the Adult IBD Population

Certainty Assessment								Summary of Findings	
Studies	Risk of bias	Inconsistency	Indirectness	Imprecision	Other considerations	Certainty of Evidence	Overall Certainty of evidence	Study Event Rates	Relative Effect (95% CI)
**Immunogenicity (Seroresponse defined as anti-HBs antibody > 10 U/L) - IMPORTANT**							⊕⊝⊝⊝ VERY LOW		
4 Observational studies[9-12]<br>**IBD populations**	Serious[a]	Not serious	Serious[b]	Serious[c]	None	⊕⊝⊝⊝ VERY LOW		• See **Response rates after revaccination following primary HBV vaccination failure**<br>• Response rate of revaccination by repeat vaccination series (3 additional doses) in primary HBV vaccination non-responders ranged from 42-68%	
**Serious adverse effects - CRITICAL**									
1 Observational study[10]<br>**IBD populations**	Serious[a]	Not serious	Not serious	Serious[c]	None	⊕⊝⊝⊝ VERY LOW		• **No serious vaccine-related adverse events**	

Footnotes:

a. Downgraded for study limitations. Possible Selection bias: only a proportion of patients who failed primary vaccination were revaccinated. Unclear how these patients were selected.
b. Downgraded for indirectness as surrogate outcomes of immunogenicity (not patient-important outcomes) were used in these studies.
c. Downgraded for imprecision due to small sample sizes.

Response rates after revaccination following primary HBV vaccination failure

	Number of patients (n)	Mean age (years)	Use of IS	Definition of response	Vaccine dose	Response after 1st vaccination	Response after 2nd vaccination	Cumulative response after 1st and 2nd vaccination
Cossio-Gil[9] 2015	172	44.5	53% AZA<br>13% biologics<br>8% 5ASA	Anti-HBs > 10 IU/L	First and Second: 10mcg or 20mcg<br>0, 1, 3-6 mos	50.6%<br>(42.9-58.3%)	52.8%<br>(38.6-66.7%)	66.8%<br>(59.3-73.8%)
Loras 2014[12]	254	Age 40 +/- 0.7	100% anti-TNF<br>74% IM	Seroprotection: anti-HBs 10-100 IU/L<br><br>Effective vaccination: Anti-HBs > 100 IU/L	First and Second: Double dose (40ug)<br>Engerix B<br>0, 1, 2 mos	Seroprotection: 43.5%<br><br>Effective vaccination: 26.4%	Seroprotetion: 44.4%<br><br>Effective vaccination: 31.3%	Seroprotection: 56.7%<br>(50-62%)
Sempere 2013[10]	105	41 +/-11	73.3% AZA<br>2.9% MTX<br>47.6% steroids<br>23.8% anti-TNF	Anti-HBs > 10 IU/L	First: 20mg<br>0, 1 and 6 mos<br><br>Second:40mg<br>0, 1 and 6 mos	47.6%<br>(37.6-57.6%)	68%	85.1%<br>(77.4-92.8%)
Gisbert 2012b[15]	241	44+/-14	30% IM<br>9% anti-TNF<br>10% combined<br>51% no IS	Anti-HBs > 100 IU/L	First and Second: Double dose (40ug)<br>Engerix B<br>0, 1, 2 mos	39%<br>(23-45%)	42%<br>(29-54%)	65%

AZA: Azathioprine
MTX: Methotrexate
IM: immunomodulators
IS: immunosuppressants

Double dose vs. Standard dose HBV vaccination in IBD patients

Certainty Assessment								Summary of Findings	
Studies	Risk of bias	Inconsistency	Indirectness	Imprecision	Other considerations	Certainty of Evidence	Overall Certainty of evidence	Study Event Rates	Relative Effect (95% CI)
**Immunogenicity (Seroresponse defined as anti-HBs antibody > 10 U/L) - IMPORTANT**							⊕⊖⊖⊖ VERY LOW		
4 Observational studies[11,12,15,16] **IBD populations**	Serious[a]	Serious[b]	Serious[c]	Serious[d]	None	⊕⊖⊖⊖ VERY LOW		• See **Response rates with double dose vs. standard dose of HBV vaccine** • Results are inconsistent, but double dose may be associated with higher serological response than standard dose in IBD patients	
**Serious adverse effects - CRITICAL**									
1 Observational study[15] **IBD populations**	Serious[a]	Not serious	Not serious	Serious[d]	None	⊕⊖⊖⊖ VERY LOW		• **No serious vaccine-related adverse events**	

Footnotes:

a. Downgraded for study limitations. Possible residual confounding factors for the 2 studies that compared standard vs. high dose vaccination dose between 2 groups of patients. In 1 study (Gisbert 2012a), more patients who received standard dose of vaccination were on any immunosuppressant, any anti-TNF, and concomitant thiopurine and anti-TNF therapy. Possible selection bias as it is unclear how patients were selected to receive standard vs. high dose vaccination.
b. Downgraded for inconsistency. One study (Haykir Solay 2019) showed no difference in serological response between standard vs. double dose HBV vaccine in patients with a variety of autoimmune conditions (no subgroup data provided for the 15 IBD patients). Another study (Gisbert 2012a) suggested accelerated schedule of double dose was associated with higher serological response than standard dose at standard schedule. The cohort study (Gisbert 2012b) suggested even with accelerated schedule of double dose vaccination, the serological response was still low in IBD patients.
c. Downgraded for indirectness as surrogate outcomes of immunogenicity (not patient-important outcomes) were used in these studies. 1 study (Haykir Solay 2019) also included autoimmune diseases other than IBD. Two studies (Gisbert 2012a and Gibsert 2012b) used accelerated protocol at 0, 1, 2 months for double dose, the other study (Haykir Solay 2019) used routine schedule at 0, 1, and 6 months for both standard and double dose.
d. Downgraded for imprecision due to small sample sizes.

Response rates with double dose vs. standard dose of HBV vaccine

	Number of patients (n)	Mean age (years)	Use of IS	Definition of response	Vaccine dose	Serological Response
Haykir Solay 2019[10]	109 (83 psoriasis, 6 RA, 3 hidradenitis supurativa, 1 Behcet's, 1 AS, 12 CD, 3 UC)	44.8 +/-10.3	62 ADA<br>25 Ustekinumab<br>12 Infliximab<br>9 Etanercept<br>1 Golimumab	Anti-HBs > 10 IU/L	Standard:<br>Engerix-B 20ug<br>0, 4, 24 weeks<br><br>Double dose:<br>Engerix-B 40ug<br>0, 4, 24 weeks	Standard: 49.3%<br><br>Double: 61.1%<br><br>(P > 0.05)
Loras 2014[6]	254	Age 40 +/- 0.7	100% anti-TNF<br>74% IM	Seroprotection: anti-HBs 10-100 IU/L<br><br>Effective vaccination: Anti-HBs > 100 IU/L	Double dose:<br>Engerix B 40ug<br>0, 1, 2 mos	Anti-HBs > 10 IU/L<br>Double: 43.5%<br><br>Anti-HBs > 100 IU/L<br>Double: 26.4%
Gisbert 2012a[9]	148	40	22% thiopurines<br>23% Anti-TNF<br>25% combined<br>30% no IS<br><br>(more patients on IS received standard dose)	Anti-HBs > 10 IU/L<br><br>Anti-HBs > 100 IU/L	Standard:<br>Engerix-B 20ug<br>0, 1, 6 mos<br><br>Double dose:<br>Engerix-B 40ug<br>0, 1, 2 mos	Anti-HBs > 10 IU/L<br>Standard: 41% (29-54%)<br>Double: 75% (65-85%)<br>P < 0.001<br><br>Anti-HBs > 100 IU/L<br>Standard: 22% (11-33%)<br>Double: 55% (43-66%)<br>P < 0.001

Gisbert 2012b[5]	241	44+/-14	30% IM<br>9% anti-TNF<br>10% combined<br>51% no IS	Seroconversion:<br>Anti-HBs > 10 IU/L<br><br>Complete response:<br>Anti-HBs > 100 IU/L	Double dose:<br>Engerix B 40ug<br>0, 1, 2 mos	Anti-HBs > 10 IU/L<br>Double: 59% (52-65%)<br><br>Anti-HBs > 100 IU/L<br>Double: 39% (23-45%)

IM = immunomodulators
IS = immunosuppressives

Check titers periodically and administer booster doses as required in IBD patients who responded to HBV vaccination?

Certainty Assessment								Summary of Findings	
Studies	Risk of bias	Inconsistency	Indirectness	Imprecision	Other considerations	Certainty of Evidence	Overall Certainty of evidence	Study Event Rates	Relative Effect (95% CI)
**Immunogenicity (Loss of seroresponse defined as anti-HBs antibody < 10 U/L) - IMPORTANT**							⊕⊖⊖⊖<br>VERY LOW		
2 Observational studies[12,17]	Serious[a]	Not serious	Serious[b]	Serious[c]	None	⊕⊖⊖⊖<br>VERY LOW		• In IBD patients with a response to HBV vaccination, cumulative incidence of loss of anti-HBs titers was 2% after 6 months and 15% after 12 months[16]<br>• Anti-TNF was the only factor associated with a higher risk of loss of anti-HBs antibody (HR 3.1, 95% CI 1.1-8.8)[16]	

Footnotes:

a. Downgraded for study limitations. Possible residual confounding factors as the study did not adjust or account for disease activity / duration, smoking, nutritional status. Possible selection bias as patients who responded to HBV vaccination and were selected to follow were likely to be different than those who were not selected (? sicker patients with more comorbidities may be selected into the study).
b. Downgraded for indirectness as surrogate outcomes of immunogenicity (not patient-important outcomes) were used in this study.
c. Downgraded for imprecision due to small sample size.

References:

1. Coutinho RA, Lelie PN, Albrecht-van Lent P, Stoutjesdijk L, Huisman J, Kuipers H, Schut LJ, Reerink-Brongers EE, Reesink HW, van Aken WG. Efficacy trial of heat-inactivated hepatitis B vaccine (CLB) in male homosexuals in the Netherlands. Dev Biol Stand. 1983;54:287-92.
2. Crosnier J, Jungers P, Courroucé AM, Laplanche A, Benhamou E, Degos F, Lacour B, Prunet P, Cerisier Y, Guesry P. Randomised placebo-controlled trial of hepatitis B surface antigen vaccine in french haemodialysis units: II, Haemodialysis patients. Lancet. 1981 Apr 11;1(8224):797-800.
3. Szmuness W, Stevens CE, Harley EJ, Zang EA, Alter HJ, Taylor PE, DeVera A, Chen GT, Kellner A. Szmuness W, Stevens CE, Harley EJ, Zang EA, Alter HJ, Taylor PE, DeVera A, Chen GT, Kellner A. N Engl J Med. 1982 Dec 9;307(24):1481-6.
4. Francis DP, Hadler SC, Thompson SE, Maynard JE, Ostrow DG, Altman N, Braff EH, O'Malley P, Hawkins D, Judson FN, Penley K, Nylund T, Christie G, Meyers F, Moore JN Jr, Gardner A, Doto IL, Miller JH, Reynolds GH, Murphy BL, Schable CA, Clark BT, Curran JW, Redeker AG. The prevention of hepatitis B with vaccine. Report of the centers for disease control multi-center efficacy trial among homosexual men. Ann Intern Med. 1982 Sep;97(3):362-6.
5. Szmuness W, Stevens CE, Harley EJ, Zang EA, Oleszko WR, William DC, Sadovsky R, Morrison JM, Kellner A. Hepatitis B vaccine: demonstration of efficacy in a controlled clinical trial in a high-risk population in the United States. N Engl J Med. 1980 Oct 9;303(15):833-41.
6. Dienstag JL, Werner BG, Polk BF, Snydman DR, Craven DE, Platt R, Crumpacker CS, Ouellet-Hellstrom R, Grady GF. Hepatitis B vaccine in health care personnel: safety, immunogenicity, and indicators of efficacy. Ann Intern Med. 1984 Jul;101(1):34-40.
7. Jiang H, Wang S, Deng M et al. Immune response to hepatitis B vaccination among people with inflammatory bowel diseases: A systematic review and meta-analysis. *Vaccine.* 2017, 35: 2633-2641.
8. Wyant T, Leach T, Sankoh S, et al. Vedolizumab affects antibody responses to immunisation selectively in the gastrointestinal tract: randomised controlled trial results *Gut*. 2015;64:77–83
9. Cossio-Gil Y, Martínez-Gómez X, Campins-Martí M, Rodrigo-Pendás JÁ, Borruel-Sainz N, Rodríguez-Frías F, Casellas-Jordà F. Immunogenicity of hepatitis B vaccine in patients with inflammatory bowel disease and the benefits of revaccination. J Gastroenterol Hepatol. 2015 Jan;30(1):92-8.
10. Sempere L, Almenta I, Barrenengoa J, Gutiérrez A, Villanueva CO, de-Madaria E, García V, Sánchez-Payá J. Factors predicting response to hepatitis B vaccination in patients with inflammatory bowel disease. Vaccine. 2013 Jun 26;31(30):3065-71.
11. Gisbert JP, Villagrasa JR, Rodríguez-Nogueiras A, Chaparro M. Efficacy of hepatitis B vaccination and revaccination and factors impacting on response in patients with inflammatory bowel disease. Am J Gastroenterol. 2012 Oct;107(10):1460-6.
12. Loras C, Gisbert JP, Saro MC, Piqueras M, Sánchez-Montes C, Barrio J, Ordás I, Montserrat A, Ferreiro R, Zabana Y, Chaparro M, Fernández-Bañares F, Esteve M; REPENTINA study, GETECCU group (Grupo Español de trabajo de Enfermedades de Crohn y Colitis Ulcerosa). Impact of surveillance of hepatitis b and hepatitis c in patients with inflammatory bowel disease under anti-TNF therapies: multicenter prospective observational study (REPENTINA 3). J Crohns Colitis. 2014 Nov;8(11):1529-38.
13. Pratt PK Jr, Nunes D, Long MT, Farraye FA. Improved Antibody Response to Three Additional Hepatitis B Vaccine Doses Following Primary Vaccination Failure in Patients with Inflammatory Bowel Disease. Dig Dis Sci. 2019 Jul;64(7):2031-2038.
14. Schillie S, Vellozzi C, Reingold A, Harris A, Haber P, Ward JW, Nelson NP. Prevention of Hepatitis B Virus Infection in the United States: Recommendations of the Advisory Committee on Immunization Practices. MMWR Recomm Rep. 2018 Jan 12;67(1):1-31.
15. Gisbert JP, Menchén L, García-Sánchez V, Marín I, Villagrasa JR, Chaparro M. Comparison of the effectiveness of two protocols for vaccination (standard and double dosage) against hepatitis B virus in patients with inflammatory bowel disease. Aliment Pharmacol Ther. 2012 Jun;35(12):1379-85.

16. Haykir Solay A, Eser F. High dose hepatitis B vaccine is not effective in patients using immunomodulatory drugs: a pilot study. Hum Vaccin Immunother. 2019;15(5):1177-1182.
17. Gisbert JP, Villagrasa JR, Rodríguez-Nogueiras A, Chaparro M. Kinetics of anti-hepatitis B surface antigen titers after hepatitis B vaccination in patients with inflammatory bowel disease. Inflamm Bowel Dis. 2013 Mar;19(3):554-8.
18. Stevens CE, Alter HJ, Taylor PE, Zang EA, Harley EJ, Szmuness W. Hepatitis B vaccine in patients receiving hemodialysis. Immunogenicity and efficacy. N Engl J Med. 1984 Aug 23;311(8):496-501.

Evidence to Decision Table – Adults (with a risk factor for hepatitis B infection)

**PICO 12A**	**In unimmunized adult patients with IBD (with a risk factor for hepatitis B infection), should vaccination vs. no vaccination against hepatitis B be given?**
**Population**	Adult patients with IBD with documented or presumed lack of immunity against hepatitis B (with a risk factor for hepatitis B infection)
**Intervention**	Vaccination against hepatitis B
**Comparator**	No vaccination against hepatitis B
**Outcome**	Mortality, VPI (hepatitis B infection), SAEs, Immunogenicity

	Judgement	Research evidence	Additional considerations
**Desirable Effects**	**How substantial are the desirable anticipated effects?**<br><br>○ Trivial<br>○ Small<br>○ Moderate<br>○ Large<br><br>○ Varies<br>○ Don't know	**Risks of Hepatitis B infection in IBD Population**<br><br>Ten cross-sectional observational studies addressed this PICO question.[1-10] Older observational studies in the Western countries showed higher prevalence of past HBV infection among IBD patients compared to the general population. However, more recent studies in Western countries suggested that the prevalence of present and past HBV infection in IBD patients is similar to that in the general population perhaps due to more satisfactory preventative measures in hospitals, better decontamination of surgical and endoscopic equipment, more effective screening of blood products, increased vaccination coverage, and IBD patients' avoidance of risk-associated	

Undesirable Effects	**How substantial are the undesirable anticipated effects?**<br><br>○ Large<br>○ Moderate<br>○ Small<br>○ Trivial<br><br>○ Varies<br>○ Don't know	behavior. In Eastern countries where HBV is endemic, the prevalence rates of past HBV infection (not present infection) in IBD populations appear to be higher than in the general population. It is however difficult to compare prevalence of HBV infection across studies because of variations in background prevalence of HBV infection, policy of infection control, implementation of vaccination programs over time in different countries and also within the same country (studies), and differences in the mean age of patients included in each of these studies. Therefore, the studies were not pooled together in a meta-analysis.<br><br>The GRADE rating started as high as these were considered prognostic studies (providing evidence that the likelihood of HBV infection in patients with IBD). The rating was further downgraded to very low due to study limitations (residual confounding factors, detection bias, admission bias), indirectness (populations and outcomes), and inconsistency. Hospitalized patients or highly selected patients in tertiary care centers were included, and they may not be representative of all patients with IBD. Patients with IBD may be more likely to be screened for Hepatitis B infection due to more outpatient visits and hospitalization compared to the general population. As well, serologic outcomes were used to estimate patient-important outcomes such as chronic active infection, liver cirrhosis, and/or hepatocellular carcinoma. Present and past HBV infection in the included studies was defined according to the terminology adopted by the National Institutes of Health Conferences on management of Hepatitis B. Present HBV infection is defined by positive HBsAg and included chronic hepatitis B and inactive HBsAg carrier state. However, inactive carrier state carries a very good prognosis in the spectrum of chronic HBV infection, with low rates of reactivation, hepatocellular carcinoma and progression of disease to cirrhosis. In contrast, chronic hepatitis B infection (HBeAg positive or HBeAg negative) has a higher risk of progression to liver cirrhosis and/or hepatocellular carcinoma. Cross-sectional designs cannot distinguish these two entities as inactive HBsAg carrier state is diagnosed by absence of HBeAg and presence of anti-HBe, undetectable levels of HBV DNA in PCR, repeatedly normal ALT, and minimal or no necroinflammation, slight fibrosis, or even histology on biopsy (although biopsy is not indicated to make the diagnosis in these patients). A minimum follow-up of 1 year with ALT levels at least every 3- 4months and serum HBV DNA level is required before classifying a patient as inactive HBV carrier. As well, past HBV infection included resolved hepatitis B defined by presence of anti-HBc with or without anti-HBs. HBV DNA levels were not measured in HBsAg negative patients with anti-HBc, therefore, the level of occult HBV infection is unknown. Anti-HBc positive patients with occult infection may have reactivation of infection during treatment with immunosuppressives. **In summary, there is very low certainty evidence that adult IBD patients have a comparable or increased risk of HBV infection compared to non-IBD patients.**<br><br>**Effectiveness and safety of HBV vaccine in adult IBD patients**<br><br>There were no RCTs or observational studies comparing HBV vaccination versus placebo or no vaccination in adult patients with IBD to address this PICO question.<br><br>CDC ACIP has assessed the evidence of effectiveness and safety of HBV vaccine among	

		adults with diabetes. They included 6 RCTs which assessed the risk of hepatitis B infection (mostly in high risk non-diabetic adults including health care personnel, homosexuals, and patients on hemodialysis).[11-16] HBV vaccine was found to reduce the risk of hepatitis B infection by 63% (RR 0.37, 95% CI 0.29-0.48, NNT 261).[11-16] Seroprotection was achieved in 91.6% (95% CI 87.6-94.4%) among vaccinated persons. The evidence was rated as high for both effectiveness and safety by the CDC, but downgraded to moderate when applied to persons with diabetes.<br><br>One systematic review of 13 observational studies (observational data from 1 RCT, 6 prospective cohort and 6 retrospective cohort studies) assessed the response rate of HBV vaccination in patients with IBD using the surrogate outcome of anti-HBs antibody threshold > 10 IU/L.[17] Most studies used the standard HBV vaccine dose of 20ug at 0-, 1-, and 6-month schedule. The pooled rate of an immune response among all IBD patients was 61% (95% CI 53-69%).[17] There was considerable heterogeneity (I^2 = 92%) which could not be accounted for by the subgroup analyses based on study design, published state, vaccine dose, or IBD drug use. Younger age and vaccination during remission were identified as positive predictors of a serological response to vaccination.[17] A low response rate was seen amongst IBD patients receiving immunosuppressive therapies (corticosteroids, immunomodulators, anti-TNF). The authors concluded that the lowest response was seen in those receiving anti-TNF therapies. However, the confidence intervals of all subgroups of medications overlap.[17] Hence, all immunosuppressive medications were associated with a reduced serologic response to HBV vaccine in IBD patients. In one large prospective study of 389 IBD patients starting anti-TNF therapy, 254 patients were found to have anti-HBs < 100 IU/L.[22] They were vaccinated with accelerated double 40ug HBV vaccine dose at 0-, 1-, and 2-month schedule. Effective vaccination and seroprotection were achieved in 26.4% and 43.5% of patients, and for revaccination 31.3% and 44.4%, respectively.[22] At the end of the vaccination, a total of 56.7% of patients achieved seroprotection.[22] Age ≤ 30 years and the use of anti-TNF monotherapy were the only predictive factors for seroprotection.[22]<br><br>**The evidence suggests that HBV vaccine can induce seroconversion or seroresponse in a significant proportion of adult IBD patients (although the response appears to be reduced compared to the general population). Young age and vaccination during remission are associated with improved serologic response to HBV vaccination. Use of immunosuppressive medications (e.g. immunomodulators, anti-TNF, steroids) is associated with a reduced immunologic response to HBV vaccination in IBD patients.** However, it is uncertain if this reduced immunologic response is clinically relevant/important and would still afford clinical protection as no studies have assessed patient-important clinical outcomes. No serious adverse events including disease exacerbation was reported. The GRADE rating started at low due to the observational designs of the studies. The rating was downgraded to very low due to study limitations (selection bias, residual confounding), inconsistency, and indirectness (use of surrogate outcomes).<br><br>A standard 3-dose HBV vaccination confers protective antibody formation in more than 95% of healthy infants, children and young adults. According to the WHO, an anti-HBs	

		concentration of > 10 IU/L (seroconversion) measured 1-3 months after administration of the last dose of the primary vaccination is considered a reliable marker of protection against infection. However, **the protective antibody titer induced by vaccination is under debate**. Increasingly, an effective immune response (complete response according to the standard definition of efficacy) is defined as anti-HBs > 100 IU/mL. **Due to lack of studies with clinical outcomes comparing the 2 different definitions of serologic protection, it is therefore uncertain what the correlates of vaccine-induced protection should be in IBD patients.**<br><br>One RCT assessed the effects of vedolizumab on the serological response to HBV vaccine in healthy participants (not IBD patients).[18] 127 healthy participants were randomized to receive either a single dose of vedolizumab 750mg IV or placebo. After 4 days, they were given HBV vaccine days 4, 32, and 60. Vedolizumab did not alter the response to HBV vaccination among healthy participants. The response rate defined as anti-HBs antibody $\geq$ 10 U/L was 90.3% in placebo group vs. 88.5% in the vedolizumab group. The GRADE rating started as high, but was downgraded to very low due to indirectness (healthy participants, surrogate outcomes) and imprecision. **In summary, there is <u>very low</u> certainty evidence that vedolizumab is not associated with a reduced serologic response to HBV vaccination in patients with IBD.**<br><br>The overall evidence was anchored to the general population (individuals with high risk for contracting hepatitis B infection). Although there were studies on safety and immunogenicity on HBV vaccine in adult IBD populations, the evidence suggests that the vaccine may not be as immunogenic (and therefore as effective) in the IBD populations compared to the general population. Therefore, the evidence for effectiveness was downgraded to moderate for HBV vaccine in adult iBD populations. For safety, the sample sizes in the IBD studies were insufficient to detect rare adverse events. Therefore, the evidence for safety was also downgraded to moderate for HBV vaccine in adult IBD populations. **Overall, there is moderate certainty evidence that HBV vaccine is safe and effective in adult IBD patients (with a risk factor for hepatitis B infection). The evidence was downgraded to low in adult IBD patients (without a risk factor for hepatitis B infection) due to indirectness (studies in the general population were done in individuals at high risk for hepatitis B infection).**<br><br>**Revaccination following primary vaccination failure**<br>There is no RCT to address this question. Four observational studies all conducted in Spain assessed revaccination with repeat vaccination series of 3 additional doses of HBV (20mcg or 40mcg).[19-22] The response rate of revaccination by repeat 3-dose vaccination series is about 50% (range 42-68%).[19-22] The GRADE rating started at low due to the observational nature of these studies. The rating was downgraded to very low due to study limitations, indirectness (surrogate outcomes) and imprecision. **In summary, there is <u>very low</u> certainty evidence that repeat vaccination 3-dose series is safe and effective in reducing the risks of HBV infection in IBD patients following primary vaccination failure.**<br><br>There is one retrospective cohort study by Pratt et al. that was published outside the literature search comparing 3 vs. 1 or 2 additional HBV doses following primary	

vaccination failure in adult patients with IBD.[23] This study cannot be included in the evidentiary base as it is outside our search date. The study showed that in immunocompromised patients with IBD who failed primary HBV vaccination, 3 additional doses of vaccine were more likely to achieve seroprotective HBsAb levels than patients who received 1 or 2 doses (62.9% vs. 40.2%; OR 1.77, P = 0.01; OR 1.9, P = 0.03, respectively, after adjusting for age, sex, race, immunosuppressive medication exposure, time between vaccine/titer).[23] Due to the retrospective nature of this study, it cannot reliably distinguish between primary HBV vaccination non-responders and initial responders with waning antibody titers but ability to mount an anamnestic response once re-challenged with a booster vaccination.

Among immunocompetent patients who do not respond to an initial 3-dose HBV vaccination schedule, meta-analyses have reported that between 25-50% will respond to an additional booster dose, while between 44-100% will respond to a repeat 3-dose vaccine series.[24]

Double dose vs. standard dose HBV vaccination in IBD patients

There is no RCT to address this question. Two observational studies (1 conducted in Spain and 1 in Turkey) compared double dose vs. standard dose HBV vaccination.[25,26] One study included patients with a variety of autoimmune conditions (15 patients with IBD).[26] The other study included only IBD patients.[25] Two cohort studies conducted in Spain assessed serological response to double dose HBV vaccination in IBD patients without a comparison group.[21,22] There was inconsistency in the results with one study suggesting no difference in serological response between double dose vs. standard dose HBV vaccination administered as per standard schedule in patients with autoimmune conditions, and the other study suggesting higher serological response with accelerated schedule of double dose HBV vaccination in IBD patients.[25,26] Nevertheless, both cohort studies with no comparison group suggested that the serological response was still low with accelerated schedule of double dose HBV vaccination in IBD patients.[21,22] The GRADE rating started at low due to the observational nature of these studies. The rating was downgraded to very low due to study limitations, indirectness (surrogate outcomes) and imprecision. **In summary, there is <u>very low</u> certainty evidence that double dose HBV vaccination is associated with a higher or comparable serological response as standard dose HBV vaccination in IBD patients.**

Check titers periodically and administer booster doses as required in IBD patients who responded to HBV vaccination?

There is no RCT or observational studies that addressed this question (comparing measuring vs. not measuring anti-HBs titer periodically and giving vs. not giving booster doses when anti-HBs titer is low).

One prospective observational study included 100 IBD patients who responded to HBV vaccination (anti-HBs > 10 IU/L at 1 – 3 months). The anti-HBs titers were measured at 6 and 12 months.[27] The cumulative incidence of loss of anti-HBs titer was 2% after 6 months and 15% at 12 months.[27] The incidence rate of loss of protective anti-HBs titers was 18% per patient-year. Treatment with anti-TNFs was associated with a higher risk

		of loss of anti-HBs (HR 3.1, 95% CI 1.1-8.8).[27] In another prospective observational study that included 99 IBD patients starting ani-TNF with previous effective vaccination (anti-HBs > 100 IU/L), 90% maintained titers 4 months after the beginning of anti-TNF treatment, and 81% of patients maintained the titers after a mean follow-up of 29 months.[22] Yet, the clinical significance of loss of anti-HBs titers in patients with IBD is unknown. Anti-HBs titers frequently become undetectable over time in healthy persons. A number of long-term studies performed in different epidemiological contexts have confirmed that clinical HBV infection rarely occurs among successfully vaccinated people, even though anti-HBs titers decline to < 10 IU/L. Therefore, protection against breakthrough HBV infection may be dependent on immunologic memory rather than on anti-HBs levels. However, clinically significant HBV infection has been documented in immunocompromised responders (HIV and those underlying hemodialysis) who do not maintain anti-HBs concentration > 10 IU/L.[28] Therefore, the CDC recommends annual anti-HBs testing for these patients and a booster dose be administered when anti-HBs levels decrease to < 10 IU/L. However, for other immunocompromised patients (e.g. IBD), the need for booster is uncertain (risks for contracting HBV may not be as high as patients with HIV or on hemodialysis; no study or report on the risks of breakthrough HBV infection in previously vaccinated IBD patients). The GRADE rating started at low due to the observational design of this study. The rating was downgraded to very low due to study limitations (residual confounding, selection bias), indirectness (surrogate outcomes), and imprecision.<br><br>**In summary, there is <u>very low</u> certainty evidence that IBD patients have loss of protective anti-HBs titers over time. Yet, the potential benefits and harms of periodically measuring anti-HBs titers and giving booster when titer is low are highly uncertain. In particular, there are no studies with long term follow-up assessing the safety and effectiveness of repeated administration of booster in patients with autoimmune diseases. It is also uncertain what the target anti-HBs titers should be for IBD patients especially for those who are on immunosuppressive therapies.**	
**Certainty of evidence**	**What is the overall certainty of the evidence of effects?**<br><br>○ Very low<br>○ Low<br>○ Moderate<br>○ High<br><br>○ No included studies		
**Values and Preference s**	**Is there important uncertainty about or variability in how much people value the main outcomes?**<br><br>○ Important uncertainty or variability	Patients likely value patient-important outcomes (mortality, chronic active hepatitis, cirrhosis, hepatocellular cancer, adverse effects) more than surrogate outcomes (immunogenicity).	

<table>
<tr>
<td></td>
<td>○ Possibly important uncertainty or variability
○ Probably no important uncertainty or variability
○ No important uncertainty or variability</td>
<td></td>
<td></td>
</tr>
<tr>
<td>Balance of effects</td>
<td>Does the balance between desirable and undesirable effects favor the intervention or the comparison?

○ Favors the comparison
○ Probably favors the comparison
○ Does not favor either the intervention or the comparison
○ Probably favors the intervention
○ Favors the intervention

○ Varies
○ Don't know</td>
<td></td>
<td></td>
</tr>
<tr>
<td>Resources required</td>
<td>How large are the resource requirements (costs)?

○ Large costs
○ Moderate costs
○ Negligible costs and savings
○ Moderate savings
○ Large savings

○ Varies
○ Don't know</td>
<td>CDC vaccine price list last reviewed/updated: July 1, 2019

<table>
<tr><th>Brandname</th><th>CDC cost/dose</th><th>Private sector cost/dose</th></tr>
<tr><td>Engerix-B (adult)</td><td>$29.73</td><td>$58.95</td></tr>
<tr><td>Engerix B (ped/adolescent)</td><td>$16.02</td><td>$23.72</td></tr>
</table>
Engerix-Bis given as a 3-dose vaccine typically at 0, 1, 6 months.</td>
<td></td>
</tr>
<tr>
<td>Certainty of Evidence of Required Resources</td>
<td>What is the certainty of the evidence of resource requirements (costs)?

○ Very low
○ Low
○ Moderate
○ High

○ No included studies</td>
<td>The costs of delivering routine immunization services may vary widely across countries and different health system settings. See Immunization Costing Action Network (ICAN) Immunization Delivery Cost Catalogue. http://immunizationeconomics.org/ican-idcc.</td>
<td></td>
</tr>
</table>

**Cost effectiveness**	**Does the cost-effectiveness of the intervention favor the intervention or the comparison?**<br><br>○ Favors the comparison<br>○ Probably favors the comparison<br>○ Does not favor either the intervention or the comparison<br>○ Probably favors the intervention<br>○ Favors the intervention<br><br>○ Varies<br>○ No included studies	There are no studies that addressed this question specifically in the IBD population.<br><br>A cost-effectiveness study of HBV vaccination strategies found that vaccination (with or without screening) prevents more disease at somewhat increased cost than no vaccination for the neonatal, adolescent and adult populations.[29] Vaccination (with or without screening) is a dominant strategy in adult high-risk populations (those with HBV incidence > 5%; lower cost and greater benefit than no vaccination).[29] When HBV vaccine is administered to all children at age 10 and again 10 years later (incremental cost-per-year-of-life-saved relative to the "no vaccination" strategy is $375).[29] A strategy of universal newborn vaccination alone leads to an incremental cost-per-year-of-life saved of $3332. If adolescents are vaccinated at age 10, incremental cost-per-year-of-life saved is $13,938**; for the general adult population, the incremental cost-per-year-of-life saved of universal vaccination is $54,524.**[29]<br><br>A cost-effectiveness study of college-based vaccination against HBV and Hepatitis A was performed.[30] The authors developed epidemiologic models to consider infection risks and disease progression and then compared the cost of vaccination with economic, longevity, and quality of life benefits. Immunization of 100,000 students would prevent 1,403 acute cases of hepatitis A, 929 cases of hepatitis B, and 144 cases of chronic hepatitis B. Hepatitis B vaccination would cost the health system $7,600 per quality-adjusted life year (QALY) gained but would reduce societal costs by 6%.[30] HepatitisA/B vaccination would cost the health system $8,500 per QALY but would reduce societal costs by 12%.[30]<br><br>A study amongst a type 1 diabetes population looked at the cost effectiveness of vaccinating non-immune patients 20-59 years of age.[31] Using a 10% uptake rate, the intervention would vaccinate 528,047 people and prevent 4,271 acute and 256 chronic hepatitis B infections.[31] Net health care costs were estimated to increase by $91.4 million, and 1,218 QALYs would be gained, producing a cost-effectiveness ratio of $75,094 per QALY gained.[31] This is a moderately cost-effective strategy. As diabetes is a chronic illness similar to IBD, some parallels can be drawn with this study and the IBD population.	
**Acceptability**	**Is the intervention acceptable to key stakeholders?**<br><br>○ No<br>○ Probably no<br>○ Probably yes<br>○ Yes<br><br>○ Varies<br>○ Don't know	There are no studies that addressed this question specifically in the adult IBD population.	

Feasibility	**Is the intervention feasible to implement?**<br><br>○ No<br>○ Probably no<br>○ Probably yes<br>○ Yes<br><br>○ Varies<br>○ Don't know		

References:

1. Chen D, Luo S, Ben q et al. Prevalence of hepatitis B and C and factors for infection and nonimmune in inflammatory bowel disease patients in China. Eur J Gastro & Hep. 2017 29(5): 509-515
2. Ardesia M, Constantino G, Mondello P et al. Serology of Viral Infections and Tuberculosis Screening in an IBD Population Referred to a Tertiary Centre of Southern Italy. Gastro Res and Practice. 2017, Article ID 4139656: 1- 9.
3. He Y, Xu P, Chen Y et al. Prevalence and influences of hepatitis B virus infection on inflammatory bowel disease: a retrospective study in southern China. Int J Clin Exp Med. 2015, 8(5): 8078-8085.
4. Huang ML, Xu XT, Shen J et al. Prevalence and factors related to hepatitis B and C infection in inflammatory bowel disease patients in China: A retrospective study. J Crohns Colitis. 2014, 8(4); 282-287.
5. Kim ES, Cho KB, Park KS et al. Prevalence of Hepatitis B Viral Markers in patients with Inflammatory Bowel Disease in a Hepatitis B Endemic Area: Inadequate protective antibody levels in young patients. J Clin Gastroenterol. 2014, 48: 553-558.
6. Chevaux JB, Nani A, Oussalah A et al. Prevalence of Hepatitis B and C and Risk Factors for Nonvaccination in Inflammatory Bowel Disease Patients in Northeast France. Inflamm Bowel Dis. 2010, 16: 916-924.
7. Konstaninos HK, Tsianos VE, Zois CD et al. Inflammatory bowel disease and hepatitis B and C in Western Balkans: a referral centre study and review of the literature. J Crohns Colitis 2010, 4(4): 450-65.
8. Loras C, Saro C, Gonzalez-Huix F et al. Prevalence and factors related to hepatitis B and C in Inflammatory Bowel Disease patients in Spain: A nationwide, multicenter study. Am J Gastro. 2009, 104(1): 57-63.
9. Tolentino YFM, Fogaca HS, Zaltman C et al. Hepatitis B virus prevalence and transmission risk factors in inflammatory bowel disease patients at Clementino Fraga Filho university hospital. World J Gastroenterol. 2008, 14(20): 3201-3206.
10. Biancone L, Pavia M, Del Vecchio Blanco G et al. Hepatitis B and C virus in Crohn's Disease. Inflamm Bowel Dis. 2001, 7(4): 287-94.
11. Coutinho RA, Lelie PN, Albrecht-van Lent P, Stoutjesdijk L, Huisman J, Kuipers H, Schut LJ, Reerink-Brongers EE, Reesink HW, van Aken WG. Efficacy trial of heat-inactivated hepatitis B vaccine (CLB) in male homosexuals in the Netherlands. Dev Biol Stand. 1983;54:287-92.
12. Crosnier J, Jungers P, Couroucé AM, Laplanche A, Benhamou E, Degos F, Lacour B, Prunet P, Cerisier Y, Guesry P. Randomised placebo-controlled trial of hepatitis B surface antigen vaccine in french haemodialysis units: II, Haemodialysis patients. Lancet. 1981 Apr 11;1(8224):797-800.
13. Szmuness W, Stevens CE, Harley EJ, Zang EA, Alter HJ, Taylor PE, DeVera A, Chen GT, Kellner A. Szmuness W, Stevens CE, Harley EJ, Zang EA, Alter HJ, Taylor PE, DeVera A, Chen GT, Kellner A. N Engl J Med. 1982 Dec 9;307(24):1481-6.
14. Francis DP, Hadler SC, Thompson SE, Maynard JE, Ostrow DG, Altman N, Braff EH, O'Malley P, Hawkins D, Judson FN, Penley K, Nylund T, Christie G, Meyers F, Moore JN Jr, Gardner A, Doto IL, Miller JH, Reynolds GH, Murphy BL, Schable CA, Clark BT, Curran JW, Redeker AG. The prevention of

hepatitis B with vaccine. Report of the centers for disease control multi-center efficacy trial among homosexual men. Ann Intern Med. 1982 Sep;97(3):362-6.

15. Szmuness W, Stevens CE, Harley EJ, Zang EA, Oleszko WR, William DC, Sadovsky R, Morrison JM, Kellner A. Hepatitis B vaccine: demonstration of efficacy in a controlled clinical trial in a high-risk population in the United States. N Engl J Med. 1980 Oct 9;303(15):833-41.
16. Dienstag JL, Werner BG, Polk BF, Snydman DR, Craven DE, Platt R, Crumpacker CS, Ouellet-Hellstrom R, Grady GF. Hepatitis B vaccine in health care personnel: safety, immunogenicity, and indicators of efficacy. Ann Intern Med. 1984 Jul;101(1):34-40.
17. Jiang H, Wang S, Deng M et al. Immune response to hepatitis B vaccination among people with inflammatory bowel diseases: A systematic review and meta-analysis. *Vaccine.* 2017, 35: 2633-2641.
18. Wyant T, Leach T, Sankoh S, et al. Vedolizumab affects antibody responses to immunisation selectively in the gastrointestinal tract: randomised controlled trial results *Gut*. 2015;64:77–83
19. Cossio-Gil Y, Martínez-Gómez X, Campins-Martí M, Rodrigo-Pendás JÁ, Borruel-Sainz N, Rodríguez-Frías F, Casellas-Jordà F. Immunogenicity of hepatitis B vaccine in patients with inflammatory bowel disease and the benefits of revaccination. J Gastroenterol Hepatol. 2015 Jan;30(1):92-8.
20. Sempere L, Almenta I, Barrenengoa J, Gutiérrez A, Villanueva CO, de-Madaria E, García V, Sánchez-Payá J. Factors predicting response to hepatitis B vaccination in patients with inflammatory bowel disease. Vaccine. 2013 Jun 26;31(30):3065-71.
21. Gisbert JP, Villagrasa JR, Rodríguez-Nogueiras A, Chaparro M. Efficacy of hepatitis B vaccination and revaccination and factors impacting on response in patients with inflammatory bowel disease. Am J Gastroenterol. 2012 Oct;107(10):1460-6.
22. Loras C, Gisbert JP, Saro MC, Piqueras M, Sánchez-Montes C, Barrio J, Ordás I, Montserrat A, Ferreiro R, Zabana Y, Chaparro M, Fernández-Bañares F, Esteve M; REPENTINA study, GETECCU group (Grupo Español de trabajo de Enfermedades de Crohn y Colitis Ulcerosa). Impact of surveillance of hepatitis b and hepatitis c in patients with inflammatory bowel disease under anti-TNF therapies: multicenter prospective observational study (REPENTINA 3). J Crohns Colitis. 2014 Nov;8(11):1529-38.
23. Pratt PK Jr, Nunes D, Long MT, Farraye FA. Improved Antibody Response to Three Additional Hepatitis B Vaccine Doses Following Primary Vaccination Failure in Patients with Inflammatory Bowel Disease. Dig Dis Sci. 2019 Jul;64(7):2031-2038.
24. Schillie S, Vellozzi C, Reingold A, Harris A, Haber P, Ward JW, Nelson NP. Prevention of Hepatitis B Virus Infection in the United States: Recommendations of the Advisory Committee on Immunization Practices. MMWR Recomm Rep. 2018 Jan 12;67(1):1-31.
25. Gisbert JP, Menchén L, García-Sánchez V, Marín I, Villagrasa JR, Chaparro M. Comparison of the effectiveness of two protocols for vaccination (standard and double dosage) against hepatitis B virus in patients with inflammatory bowel disease. Aliment Pharmacol Ther. 2012 Jun;35(12):1379-85.
26. Haykir Solay A, Eser F. High dose hepatitis B vaccine is not effective in patients using immunomodulatory drugs: a pilot study. Hum Vaccin Immunother. 2019;15(5):1177-1182.
27. Gisbert JP, Villagrasa JR, Rodríguez-Nogueiras A, Chaparro M. Kinetics of anti-hepatitis B surface antigen titers after hepatitis B vaccination in patients with inflammatory bowel disease. Inflamm Bowel Dis. 2013 Mar;19(3):554-8.
28. Stevens CE, Alter HJ, Taylor PE, Zang EA, Harley EJ, Szmuness W. Hepatitis B vaccine in patients receiving hemodialysis. Immunogenicity and efficacy. N Engl J Med. 1984 Aug 23;311(8):496-501.
29. Bloom BS, Hillman AL, Fendrick AM, Schwartz JS. A reappraisal of hepatitis B virus vaccination strategies using cost-effectiveness analysis. Ann Intern Med. 1993 Feb 15;118(4):298-306.
30. Jacobs RJ, Saab S, Meyerhoff AS. The cost effectiveness of hepatitis immunization for US college students. J Am Coll Health. 2003 May;51(6):227-36.

31. Hoerger TJ, Schillie S, Wittenborn JS, Bradley CL, Zhou F, Byrd K, Murphy TV. Cost-effectiveness of hepatitis B vaccination in adults with diagnosed diabetes. Diabetes Care. 2013 Jan;36(1):63-9.

Conclusion – Adults (with a risk factor for hepatitis B infection)

PICO 12A: **In unimmunized adult patients with IBD (with a risk factor for hepatitis B infection), should vaccination vs. no vaccination against hepatitis B be given?**
Moderate certainty of evidence
Direction – Yes (100%)
Strength – Strong (100%)

Type of recommendation	Strong recommendation against the intervention	Conditional recommendation against the intervention	Conditional recommendation for either the intervention or the comparison	Conditional recommendation for the intervention	Strong recommendation for the intervention
	○	○	○	○	○
**Recommendation**	**Statement 12A: In unimmunized adult patients with IBD with a risk factor for hepatitis B infection, we recommend hepatitis B vaccine be given.**				
**Justification**					
**Subgroup considerations**					
**Implementation considerations**					

**Monitoring and evaluation**	• Ongoing monitoring of safety and effectiveness of the HBV vaccine in IBD populations is needed.
**Research priorities**	• Population-based studies to assess the effectiveness, safety, and duration of protection of HBV vaccine in IBD patients, especially those who are on immunosuppressants. Outcomes should include serologic response as well as patient-important outcomes.<br>• RCT is needed to determine the effectiveness, safety, and serological response of double dose standard or accelerated schedule vs. standard dose standard schedule in IBD patients<br>• Research is needed to determine the clinical relevance/importance of waning anti-HBs antibody titer in IBD patients (especially those who are immunocompromised), and the benefits vs risks of periodic monitoring of titers and administration of booster dose.

Evidence to Decision Table – Adults (without a risk factor for hepatitis B infection)

**PICO 12B**	**In unimmunized adult patients with IBD (without a risk factor for hepatitis B infection), should vaccination vs. no vaccination against hepatitis B be given?**
**Population**	Adult patients with IBD with documented or presumed lack of immunity against hepatitis B (without a risk factor for hepatitis B infection)
**Intervention**	Vaccination against hepatitis B
**Comparator**	No vaccination against hepatitis B
**Outcome**	Mortality, VPI (hepatitis B infection), SAEs, Immunogenicity

Judgement	Research evidence	Additional considerations

**Desirable Effects**	**How substantial are the desirable anticipated effects?**<br><br>○ Trivial<br>○ Small<br>○ Moderate<br>○ Large<br><br>○ Varies<br>○ Don't know	**Risks of Hepatitis B infection in IBD Population**<br><br>Ten cross-sectional observational studies addressed this PICO question.[1-10] Older observational studies in the Western countries showed higher prevalence of past HBV infection among IBD patients compared to the general population. However, more recent studies in Western countries suggested that the prevalence of present and past HBV infection in IBD patients is similar to that in the general population perhaps due to more satisfactory preventative measures in hospitals, better decontamination of surgical and endoscopic equipment, more effective screening of blood products, increased vaccination coverage, and IBD patients' avoidance of risk-associated behavior. In Eastern countries where HBV is endemic, the prevalence rates of past HBV infection (not present infection) in IBD populations appear to be higher than in the general population. It is however difficult to compare prevalence of HBV infection across studies because of variations in background prevalence of HBV infection, policy of infection control, implementation of vaccination programs over time in different countries and also within the same country (studies), and differences in the mean age of patients included in each of these studies. Therefore, the studies were not pooled together in a meta-analysis.<br><br>The GRADE rating started as high as these were considered prognostic studies (providing evidence that the likelihood of HBV infection in patients with IBD). The rating was further downgraded to very low due to study limitations (residual confounding factors, detection bias, admission bias), indirectness (populations and outcomes), and inconsistency. Hospitalized patients or highly selected patients in tertiary care centers were included, and they may not be representative of all patients with IBD. Patients with IBD may be more likely to be screened for Hepatitis B infection due to more outpatient visits and hospitalization compared to the general population. As well, serologic outcomes were used to estimate patient-important outcomes such as chronic active infection, liver cirrhosis, and/or hepatocellular carcinoma. Present and past HBV infection in the included studies was defined according to the terminology adopted by the National Institutes of Health Conferences on management of Hepatitis B. Present HBV infection is defined by positive HBsAg and included chronic hepatitis B and inactive HBsAg carrier state. However, inactive carrier state carries a very good prognosis in the spectrum of chronic HBV infection, with low rates of reactivation, hepatocellular carcinoma and progression of disease to cirrhosis. In contrast, chronic hepatitis B infection (HBeAg positive or HBeAg negative) has a higher risk of progression to liver cirrhosis and/or hepatocellular carcinoma. Cross-sectional designs cannot distinguish these two entities as inactive HBsAg carrier state is diagnosed by absence of HBeAg and presence of anti-HBe, undetectable levels of HBV DNA in PCR, repeatedly normal ALT, and minimal or no necroinflammation, slight fibrosis, or even histology on biopsy (although biopsy is not indicated to make the diagnosis in these patients). A minimum follow-up of 1 year with ALT levels at least every 3- 4months and serum HBV DNA level is required before classifying a patient as inactive HBV carrier. As well, past HBV infection included resolved hepatitis B defined by presence of anti-HBc with or without anti-HBs. HBV DNA levels were not measured in HBsAg negative patients with anti-HBc, therefore, the level of occult HBV infection is unknown. Anti-HBc positive patients with occult infection may have reactivation of infection during treatment with	
**Undesirable Effects**	**How substantial are the undesirable anticipated effects?**<br><br>○ Large<br>○ Moderate<br>○ Small<br>○ Trivial<br><br>○ Varies<br>○ Don't know		

		immunosuppressives. **In summary, there is very low certainty evidence that adult IBD patients have a comparable or increased risk of HBV infection compared to non-IBD patients.**<br><br>**Effectiveness and safety of HBV vaccine in adult IBD patients**<br><br>There were no RCTs or observational studies comparing HBV vaccination versus placebo or no vaccination in adult patients with IBD to address this PICO question.<br><br>CDC ACIP has assessed the evidence of effectiveness and safety of HBV vaccine among adults with diabetes. They included 6 RCTs which assessed the risk of hepatitis B infection (mostly in high risk non-diabetic adults including health care personnel, homosexuals, and patients on hemodialysis).[11-16] HBV vaccine was found to reduce the risk of hepatitis B infection by 63% (RR 0.37, 95% CI 0.29-0.48, NNT 261).[11-16] Seroprotection was achieved in 91.6% (95% CI 87.6-94.4%) among vaccinated persons. The evidence was rated as high for both effectiveness and safety by the CDC, but downgraded to moderate when applied to persons with diabetes.<br><br>One systematic review of 13 observational studies (observational data from 1 RCT, 6 prospective cohort and 6 retrospective cohort studies) assessed the response rate of HBV vaccination in patients with IBD using the surrogate outcome of anti-HBs antibody threshold > 10 IU/L.[17] Most studies used the standard HBV vaccine dose of 20ug at 0-, 1-, and 6-month schedule. The pooled rate of an immune response among all IBD patients was 61% (95% CI 53-69%).[17] There was considerable heterogeneity (I^2 = 92%) which could not be accounted for by the subgroup analyses based on study design, published state, vaccine dose, or IBD drug use. Younger age and vaccination during remission were identified as positive predictors of a serological response to vaccination.[17] A low response rate was seen amongst IBD patients receiving immunosuppressive therapies (corticosteroids, immunomodulators, anti-TNF). The authors concluded that the lowest response was seen in those receiving anti-TNF therapies. However, the confidence intervals of all subgroups of medications overlap.[17] Hence, all immunosuppressive medications were associated with a reduced serologic response to HBV vaccine in IBD patients. In one large prospective study of 389 IBD patients starting anti-TNF therapy, 254 patients were found to have anti-HBs < 100 IU/L.[22] They were vaccinated with accelerated double 40ug HBV vaccine dose at 0-, 1-, and 2-month schedule. Effective vaccination and seroprotection were achieved in 26.4% and 43.5% of patients, and for revaccination 31.3% and 44.4%, respectively.[22] At the end of the vaccination, a total of 56.7% of patients achieved seroprotection.[22] Age ≤ 30 years and the use of anti-TNF monotherapy were the only predictive factors for seroprotection.[22]<br><br>**The evidence suggests that HBV vaccine can induce seroconversion or seroresponse in a significant proportion of adult IBD patients (although the response appears to be reduced compared to the general population). Young age and vaccination during remission are associated with improved serologic response to HBV vaccination. Use of immunosuppressive medications (e.g. immunomodulators, anti-TNF, steroids) is associated with a reduced immunologic response to HBV vaccination in IBD patients.**	

		However, it is uncertain if this reduced immunologic response is clinically relevant/important and would still afford clinical protection as no studies have assessed patient-important clinical outcomes. No serious adverse events including disease exacerbation was reported. The GRADE rating started at low due to the observational designs of the studies. The rating was downgraded to very low due to study limitations (selection bias, residual confounding), inconsistency, and indirectness (use of surrogate outcomes).<br><br>A standard 3-dose HBV vaccination confers protective antibody formation in more than 95% of healthy infants, children and young adults. According to the WHO, an anti-HBs concentration of > 10 IU/L (seroconversion) measured 1-3 months after administration of the last dose of the primary vaccination is considered a reliable marker of protection against infection. However, **the protective antibody titer induced by vaccination is under debate**. Increasingly, an effective immune response (complete response according to the standard definition of efficacy) is defined as anti-HBs > 100 IU/mL. **Due to lack of studies with clinical outcomes comparing the 2 different definitions of serologic protection, it is therefore uncertain what the correlates of vaccine-induced protection should be in IBD patients.**<br><br>One RCT assessed the effects of vedolizumab on the serological response to HBV vaccine in healthy participants (not IBD patients).[18] 127 healthy participants were randomized to receive either a single dose of vedolizumab 750mg IV or placebo. After 4 days, they were given HBV vaccine days 4, 32, and 60. Vedolizumab did not alter the response to HBV vaccination among healthy participants. The response rate defined as anti-HBs antibody $\geq$ 10 U/L was 90.3% in placebo group vs. 88.5% in the vedolizumab group. The GRADE rating started as high, but was downgraded to very low due to indirectness (healthy participants, surrogate outcomes) and imprecision. **In summary, there is <u>very low</u> certainty evidence that vedolizumab is not associated with a reduced serologic response to HBV vaccination in patients with IBD.**<br><br>The overall evidence was anchored to the general population (individuals with high risk for contracting hepatitis B infection). Although there were studies on safety and immunogenicity on HBV vaccine in adult IBD populations, the evidence suggests that the vaccine may not be as immunogenic (and therefore as effective) in the IBD populations compared to the general population. Therefore, the evidence for effectiveness was downgraded to moderate for HBV vaccine in adult iBD populations. For safety, the sample sizes in the IBD studies were insufficient to detect rare adverse events. Therefore, the evidence for safety was also downgraded to moderate for HBV vaccine in adult IBD populations. **Overall, there is moderate certainty evidence that HBV vaccine is safe and effective in adult IBD patients (with a risk factor for hepatitis B infection). The evidence was downgraded to low in adult IBD patients (without a risk factor for hepatitis B infection) due to indirectness (studies in the general population were done in individuals at high risk for hepatitis B infection).**<br><br>**Revaccination following primary vaccination failure**<br>There is no RCT to address this question. Four observational studies all conducted in Spain assessed revaccination with repeat vaccination series of 3 additional doses of	

HBV (20mcg or 40mcg).[19-22] The response rate of revaccination by repeat 3-dose vaccination series is about 50% (range 42-68%).[19-22] The GRADE rating started at low due to the observational nature of these studies. The rating was downgraded to very low due to study limitations, indirectness (surrogate outcomes) and imprecision. **In summary, there is very low certainty evidence that repeat vaccination 3-dose series is safe and effective in reducing the risks of HBV infection in IBD patients following primary vaccination failure.**

There is one retrospective cohort study by Pratt et al. that was published outside the literature search comparing 3 vs. 1 or 2 additional HBV doses following primary vaccination failure in adult patients with IBD.[23] This study cannot be included in the evidentiary base as it is outside our search date. The study showed that in immunocompromised patients with IBD who failed primary HBV vaccination, 3 additional doses of vaccine were more likely to achieve seroprotective HBsAb levels than patients who received 1 or 2 doses (62.9% vs. 40.2%; OR 1.77, P = 0.01; OR 1.9, P = 0.03, respectively, after adjusting for age, sex, race, immunosuppressive medication exposure, time between vaccine/titer).[23] Due to the retrospective nature of this study, it cannot reliably distinguish between primary HBV vaccination non-responders and initial responders with waning antibody titers but ability to mount an anamnestic response once re-challenged with a booster vaccination.

Among immunocompetent patients who do not respond to an initial 3-dose HBV vaccination schedule, meta-analyses have reported that between 25-50% will respond to an additional booster dose, while between 44-100% will respond to a repeat 3-dose vaccine series.[24]

Double dose vs. standard dose HBV vaccination in IBD patients

There is no RCT to address this question. Two observational studies (1 conducted in Spain and 1 in Turkey) compared double dose vs. standard dose HBV vaccination.[25,26] One study included patients with a variety of autoimmune conditions (15 patients with IBD).[26] The other study included only IBD patients.[25] Two cohort studies conducted in Spain assessed serological response to double dose HBV vaccination in IBD patients without a comparison group.[21,22] There was inconsistency in the results with one study suggesting no difference in serological response between double dose vs. standard dose HBV vaccination administered as per standard schedule in patients with autoimmune conditions, and the other study suggesting higher serological response with accelerated schedule of double dose HBV vaccination in IBD patients.[25,26] Nevertheless, both cohort studies with no comparison group suggested that the serological response was still low with accelerated schedule of double dose HBV vaccination in IBD patients.[21,22] The GRADE rating started at low due to the observational nature of these studies. The rating was downgraded to very low due to study limitations, indirectness (surrogate outcomes) and imprecision. **In summary, there is very low certainty evidence that double dose HBV vaccination is associated with a higher or comparable serological response as standard dose HBV vaccination in IBD patients.**

Check titers periodically and administer booster doses as required in IBD patients

		**who responded to HBV vaccination?**<br>There is no RCT or observational studies that addressed this question (comparing measuring vs. not measuring anti-HBs titer periodically and giving vs. not giving booster doses when anti-HBs titer is low).<br><br>One prospective observational study included 100 IBD patients who responded to HBV vaccination (anti-HBs > 10 IU/L at 1 – 3 months). The anti-HBs titers were measured at 6 and 12 months.[27] The cumulative incidence of loss of anti-HBs titer was 2% after 6 months and 15% at 12 months.[27] The incidence rate of loss of protective anti-HBs titers was 18% per patient-year. Treatment with anti-TNFs was associated with a higher risk of loss of anti-HBs (HR 3.1, 95% CI 1.1-8.8).[27] In another prospective observational study that included 99 IBD patients starting ani-TNF with previous effective vaccination (anti-HBs > 100 IU/L), 90% maintained titers 4 months after the beginning of anti-TNF treatment, and 81% of patients maintained the titers after a mean follow-up of 29 months.[22] Yet, the clinical significance of loss of anti-HBs titers in patients with IBD is unknown. Anti-HBs titers frequently become undetectable over time in healthy persons. A number of long-term studies performed in different epidemiological contexts have confirmed that clinical HBV infection rarely occurs among successfully vaccinated people, even though anti-HBs titers decline to < 10 IU/L. Therefore, protection against breakthrough HBV infection may be dependent on immunologic memory rather than on anti-HBs levels. However, clinically significant HBV infection has been documented in immunocompromised responders (HIV and those underlying hemodialysis) who do not maintain anti-HBs concentration > 10 IU/L.[28] Therefore, the CDC recommends annual anti-HBs testing for these patients and a booster dose be administered when anti-HBs levels decrease to < 10 IU/L. However, for other immunocompromised patients (e.g. IBD), the need for booster is uncertain (risks for contracting HBV may not be as high as patients with HIV or on hemodialysis; no study or report on the risks of breakthrough HBV infection in previously vaccinated IBD patients). The GRADE rating started at low due to the observational design of this study. The rating was downgraded to very low due to study limitations (residual confounding, selection bias), indirectness (surrogate outcomes), and imprecision.<br><br>**In summary, there is <u>very low</u> certainty evidence that IBD patients have loss of protective anti-HBs titers over time. Yet, the potential benefits and harms of periodically measuring anti-HBs titers and giving booster when titer is low are highly uncertain. In particular, there are no studies with long term follow-up assessing the safety and effectiveness of repeated administration of booster in patients with autoimmune diseases. It is also uncertain what the target anti-HBs titers should be for IBD patients especially for those who are on immunosuppressive therapies.**	

**Certainty of evidence**	**What is the overall certainty of the evidence of effects?**<br><br>○ Very low<br>○ Low<br>○ Moderate<br>○ High<br><br>○ No included studies		
**Values and Preferences**	**Is there important uncertainty about or variability in how much people value the main outcomes?**<br><br>○ Important uncertainty or variability<br>○ Possibly important uncertainty or variability<br>○ Probably no important uncertainty or variability<br>○ No important uncertainty or variability	Patients likely value patient-important outcomes (mortality, chronic active hepatitis, cirrhosis, hepatocellular cancer, adverse effects) more than surrogate outcomes (immunogenicity).	
**Balance of effects**	**Does the balance between desirable and undesirable effects favor the intervention or the comparison?**<br><br>○ Favors the comparison<br>○ Probably favors the comparison<br>○ Does not favor either the intervention or the comparison<br>○ Probably favors the intervention<br>○ Favors the intervention<br><br>○ Varies<br>○ Don't know		

Resources required

How large are the resource requirements (costs)?

- o Large costs
- o Moderate costs
- o Negligible costs and savings
- o Moderate savings
- o Large savings

- o Varies
- o Don't know

CDC vaccine price list last reviewed/updated: July 1, 2019

Brandname	CDC cost/dose	Private sector cost/dose
Engerix-B (adult)	$29.73	$58.95
Engerix B (ped/adolescent)	$16.02	$23.72

Engerix-Bis given as a 3-dose vaccine typically at 0, 1, 6 months.

Certainty of Evidence of Required Resources

What is the certainty of the evidence of resource requirements (costs)?

- o Very low
- o Low
- o Moderate
- o High

- o No included studies

The costs of delivering routine immunization services may vary widely across countries and different health system settings. See Immunization Costing Action Network (ICAN) Immunization Delivery Cost Catalogue. http://immunizationeconomics.org/ican-idcc.

Cost effectiveness

Does the cost-effectiveness of the intervention favor the intervention or the comparison?

- o Favors the comparison
- o Probably favors the comparison
- o Does not favor either the intervention or the comparison
- o Probably favors the intervention
- o Favors the intervention

- o Varies
- o No included studies

There are no studies that addressed this question specifically in the IBD population.

A cost-effectiveness study of HBV vaccination strategies found that vaccination (with or without screening) prevents more disease at somewhat increased cost than no vaccination for the neonatal, adolescent and adult populations.[29] Vaccination (with or without screening) is a dominant strategy in adult high-risk populations (those with HBV incidence > 5%; lower cost and greater benefit than no vaccination).[29] When HBV vaccine is administered to all children at age 10 and again 10 years later (incremental cost-per-year-of-life-saved relative to the "no vaccination" strategy is $375).[29] A strategy of universal newborn vaccination alone leads to an incremental cost-per-year-of-life saved of $3332. If adolescents are vaccinated at age 10, incremental cost-per-year-of-life saved is $13,938; **for the general adult population, the incremental cost-per-year-of-life saved of universal vaccination is $54,524.**[29]

A cost-effectiveness study of college-based vaccination against HBV and Hepatitis A was performed.[30] The authors developed epidemiologic models to consider infection risks and disease progression and then compared the cost of vaccination with economic, longevity, and quality of life benefits. Immunization of 100,000 students would prevent

		1,403 acute cases of hepatitis A, 929 cases of hepatitis B, and 144 cases of chronic hepatitis B. Hepatitis B vaccination would cost the health system $7,600 per quality-adjusted life year (QALY) gained but would reduce societal costs by 6%.[30] HepatitisA/B vaccination would cost the health system $8,500 per QALY but would reduce societal costs by 12%.[30]<br><br>A study amongst a type 1 diabetes population looked at the cost effectiveness of vaccinating non-immune patients 20-59 years of age.[31] Using a 10% uptake rate, the intervention would vaccinate 528,047 people and prevent 4,271 acute and 256 chronic hepatitis B infections.[31] Net health care costs were estimated to increase by $91.4 million, and 1,218 QALYs would be gained, producing a cost-effectiveness ratio of $75,094 per QALY gained.[31] This is a moderately cost-effective strategy. As diabetes is a chronic illness similar to IBD, some parallels can be drawn with this study and the IBD population.	
**Acceptability**	**Is the intervention acceptable to key stakeholders?**<br><br>○ No<br>○ Probably no<br>○ Probably yes<br>○ Yes<br><br>○ Varies<br>○ Don't know	There are no studies that addressed this question specifically in the adult IBD population.	
**Feasibility**	**Is the intervention feasible to implement?**<br><br>○ No<br>○ Probably no<br>○ Probably yes<br>○ Yes<br><br>○ Varies<br>○ Don't know		

Conclusion – Adults (without a risk factor for hepatitis B infection)

PICO 12B: **In unimmunized adult patients with IBD (without a risk factor for hepatitis B infection), should vaccination vs. no vaccination against hepatitis B be given?**

Low certainty of evidence

Direction – Yes (100%)

Strength – conditional

Type of recommendation	Strong recommendation against the intervention	Conditional recommendation against the intervention	Conditional recommendation for either the intervention or the comparison	Conditional recommendation for the intervention	Strong recommendation for the intervention
	○	○	○	○	○
**Recommendation**	**Statement 12B: In unimmunized adult patients with IBD without a risk factor for hepatitis B infection, we suggest hepatitis B vaccine be given.**				
**Justification**					
**Subgroup considerations**					
**Implementation considerations**					
**Monitoring and evaluation**	• Ongoing monitoring of safety and effectiveness of the HBV vaccine in IBD populations is needed.				
**Research priorities**	• Population-based studies to assess the effectiveness, safety, and duration of protection of HBV vaccine in IBD patients, especially those who are on immunosuppressants. Outcomes should				

	include serologic response as well as patient-important outcomes.<br>• RCT is needed to determine the effectiveness, safety, and serological response of double dose standard or accelerated schedule vs. standard dose standard schedule in IBD patients<br>• Research is needed to determine the clinical relevance/importance of waning anti-HBs antibody titer in IBD patients (especially those who are immunocompromised), and the benefits vs risks of periodic monitoring of titers and administration of booster dose.

Evidence to Decision Table – Adults (double dose vs. standard dose HBV vaccination)

Double dose vs. standard dose HBV vaccination in IBD patients

PICO: Should double dose vs. standard dose for HBV vaccination be used in IBD patients on immunosuppressive therapy?

	Judgement	Research evidence	Additional considerations
**Desirable Effects**	**How substantial are the desirable anticipated effects?**<br><br>○ Trivial<br>○ Small<br>○ Moderate<br>○ Large<br><br>○ Varies<br>○ Don't know	**Risks of Hepatitis B infection in IBD Population**<br><br>Ten cross-sectional observational studies addressed this PICO question.[1-10] Older observational studies in the Western countries showed higher prevalence of past HBV infection among IBD patients compared to the general population. However, more recent studies in Western countries suggested that the prevalence of present and past HBV infection in IBD patients is similar to that in the general population perhaps due to more satisfactory preventative measures in hospitals, better decontamination of surgical and endoscopic equipment, more effective screening of blood products, increased vaccination coverage, and IBD patients' avoidance of risk-associated behavior. In Eastern countries where HBV is endemic, the prevalence rates of past HBV infection (not present infection) in IBD populations appear to be higher than in the general population. It is however difficult to compare prevalence of HBV infection across studies because of variations in background prevalence of HBV infection, policy of infection control, implementation of vaccination programs over time in different countries and also within the same country (studies), and differences in the mean age of patients included in each of these studies. Therefore, the studies were not pooled together in a meta-analysis.<br><br>The GRADE rating started as high as these were considered prognostic studies (providing evidence that the likelihood of HBV infection in patients with IBD). The rating was further downgraded to very low due to study limitations (residual confounding factors, detection bias, admission bias), indirectness (populations and outcomes), and	
**Undesirable Effects**	**How substantial are the undesirable anticipated effects?**<br><br>○ Large<br>○ Moderate<br>○ Small<br>○ Trivial<br><br>○ Varies<br>○ Don't know		

inconsistency. Hospitalized patients or highly selected patients in tertiary care centers were included, and they may not be representative of all patients with IBD. Patients with IBD may be more likely to be screened for Hepatitis B infection due to more outpatient visits and hospitalization compared to the general population. As well, serologic outcomes were used to estimate patient-important outcomes such as chronic active infection, liver cirrhosis, and/or hepatocellular carcinoma. Present and past HBV infection in the included studies was defined according to the terminology adopted by the National Institutes of Health Conferences on management of Hepatitis B. Present HBV infection is defined by positive HBsAg and included chronic hepatitis B and inactive HBsAg carrier state. However, inactive carrier state carries a very good prognosis in the spectrum of chronic HBV infection, with low rates of reactivation, hepatocellular carcinoma and progression of disease to cirrhosis. In contrast, chronic hepatitis B infection (HBeAg positive or HBeAg negative) has a higher risk of progression to liver cirrhosis and/or hepatocellular carcinoma. Cross-sectional designs cannot distinguish these two entities as inactive HBsAg carrier state is diagnosed by absence of HBeAg and presence of anti-HBe, undetectable levels of HBV DNA in PCR, repeatedly normal ALT, and minimal or no necroinflammation, slight fibrosis, or even histology on biopsy (although biopsy is not indicated to make the diagnosis in these patients). A minimum follow-up of 1 year with ALT levels at least every 3- 4months and serum HBV DNA level is required before classifying a patient as inactive HBV carrier. As well, past HBV infection included resolved hepatitis B defined by presence of anti-HBc with or without anti-HBs. HBV DNA levels were not measured in HBsAg negative patients with anti-HBc, therefore, the level of occult HBV infection is unknown. Anti-HBc positive patients with occult infection may have reactivation of infection during treatment with immunosuppressives. **In summary, there is <u>very low</u> certainty evidence that adult IBD patients have a comparable or increased risk of HBV infection compared to non-IBD patients.**

Effectiveness and safety of HBV vaccine in adult IBD patients

There were no RCTs or observational studies comparing HBV vaccination versus placebo or no vaccination in adult patients with IBD to address this PICO question.

CDC ACIP has assessed the evidence of effectiveness and safety of HBV vaccine among adults with diabetes. They included 6 RCTs which assessed the risk of hepatitis B infection (mostly in high risk non-diabetic adults including health care personnel, homosexuals, and patients on hemodialysis).[11-16] HBV vaccine was found to reduce the risk of hepatitis B infection by 63% (RR 0.37, 95% CI 0.29-0.48, NNT 261).[11-16] Seroprotection was achieved in 91.6% (95% CI 87.6-94.4%) among vaccinated persons. The evidence was rated as high for both effectiveness and safety by the CDC, but downgraded to moderate when applied to persons with diabetes.

One systematic review of 13 observational studies (observational data from 1 RCT, 6 prospective cohort and 6 retrospective cohort studies) assessed the response rate of HBV vaccination in patients with IBD using the surrogate outcome of anti-HBs antibody threshold > 10 IU/L.[17] Most studies used the standard HBV vaccine dose of 20ug at 0-, 1-, and 6-month schedule. The pooled rate of an immune response among all IBD

		patients was 61% (95% CI 53-69%).[17] There was considerable heterogeneity (I^2 = 92%) which could not be accounted for by the subgroup analyses based on study design, published state, vaccine dose, or IBD drug use. Younger age and vaccination during remission were identified as positive predictors of a serological response to vaccination.[17] A low response rate was seen amongst IBD patients receiving immunosuppressive therapies (corticosteroids, immunomodulators, anti-TNF). The authors concluded that the lowest response was seen in those receiving anti-TNF therapies. However, the confidence intervals of all subgroups of medications overlap.[17] Hence, all immunosuppressive medications were associated with a reduced serologic response to HBV vaccine in IBD patients. In one large prospective study of 389 IBD patients starting anti-TNF therapy, 254 patients were found to have anti-HBs < 100 IU/L.[22] They were vaccinated with accelerated double 40ug HBV vaccine dose at 0-, 1-, and 2-month schedule. Effective vaccination and seroprotection were achieved in 26.4% and 43.5% of patients, and for revaccination 31.3% and 44.4%, respectively.[22] At the end of the vaccination, a total of 56.7% of patients achieved seroprotection.[22] Age ≤ 30 years and the use of anti-TNF monotherapy were the only predictive factors for seroprotection.[22]<br><br>**The evidence suggests that HBV vaccine can induce seroconversion or seroresponse in a significant proportion of adult IBD patients (although the response appears to be reduced compared to the general population). Young age and vaccination during remission are associated with improved serologic response to HBV vaccination. Use of immunosuppressive medications (e.g. immunomodulators, anti-TNF, steroids) is associated with a reduced immunologic response to HBV vaccination in IBD patients.** However, it is uncertain if this reduced immunologic response is clinically relevant/important and would still afford clinical protection as no studies have assessed patient-important clinical outcomes. No serious adverse events including disease exacerbation was reported. The GRADE rating started at low due to the observational designs of the studies. The rating was downgraded to very low due to study limitations (selection bias, residual confounding), inconsistency, and indirectness (use of surrogate outcomes).<br><br>A standard 3-dose HBV vaccination confers protective antibody formation in more than 95% of healthy infants, children and young adults. According to the WHO, an anti-HBs concentration of > 10 IU/L (seroconversion) measured 1-3 months after administration of the last dose of the primary vaccination is considered a reliable marker of protection against infection. However, **the protective antibody titer induced by vaccination is under debate**. Increasingly, an effective immune response (complete response according to the standard definition of efficacy) is defined as anti-HBs > 100 IU/mL. **Due to lack of studies with clinical outcomes comparing the 2 different definitions of serologic protection, it is therefore uncertain what the correlates of vaccine-induced protection should be in IBD patients.**<br><br>One RCT assessed the effects of vedolizumab on the serological response to HBV vaccine in healthy participants (not IBD patients).[18] 127 healthy participants were randomized to receive either a single dose of vedolizumab 750mg IV or placebo. After 4 days, they were given HBV vaccine days 4, 32, and 60. Vedolizumab did not alter the	

response to HBV vaccination among healthy participants. The response rate defined as anti-HBs antibody ≥ 10 U/L was 90.3% in placebo group vs. 88.5% in the vedolizumab group. The GRADE rating started as high, but was downgraded to very low due to indirectness (healthy participants, surrogate outcomes) and imprecision. **In summary, there is very low certainty evidence that vedolizumab is not associated with a reduced serologic response to HBV vaccination in patients with IBD.**

The overall evidence was anchored to the general population (individuals with high risk for contracting hepatitis B infection). Although there were studies on safety and immunogenicity on HBV vaccine in adult IBD populations, the evidence suggests that the vaccine may not be as immunogenic (and therefore as effective) in the IBD populations compared to the general population. Therefore, the evidence for effectiveness was downgraded to moderate for HBV vaccine in adult iBD populations. For safety, the sample sizes in the IBD studies were insufficient to detect rare adverse events. Therefore, the evidence for safety was also downgraded to moderate for HBV vaccine in adult IBD populations. **Overall, there is moderate certainty evidence that HBV vaccine is safe and effective in adult IBD patients.**

Revaccination following primary vaccination failure
There is no RCT to address this question. Four observational studies all conducted in Spain assessed revaccination with repeat vaccination series of 3 additional doses of HBV (20mcg or 40mcg).[19-22] The response rate of revaccination by repeat 3-dose vaccination series is about 50% (range 42-68%).[19-22] The GRADE rating started at low due to the observational nature of these studies. The rating was downgraded to very low due to study limitations, indirectness (surrogate outcomes) and imprecision. **In summary, there is very low certainty evidence that repeat vaccination 3-dose series is safe and effective in reducing the risks of HBV infection in IBD patients following primary vaccination failure.**

There is one retrospective cohort study by Pratt et al. that was published outside the literature search comparing 3 vs. 1 or 2 additional HBV doses following primary vaccination failure in adult patients with IBD.[23] This study cannot be included in the evidentiary base as it is outside our search date. The study showed that in immunocompromised patients with IBD who failed primary HBV vaccination, 3 additional doses of vaccine were more likely to achieve seroprotective HBsAb levels than patients who received 1 or 2 doses (62.9% vs. 40.2%; OR 1.77, P = 0.01; OR 1.9, P = 0.03, respectively, after adjusting for age, sex, race, immunosuppressive medication exposure, time between vaccine/titer).[23] Due to the retrospective nature of this study, it cannot reliably distinguish between primary HBV vaccination non-responders and initial responders with waning antibody titers but ability to mount an anamnestic response once re-challenged with a booster vaccination.

Among immunocompetent patients who do not respond to an initial 3-dose HBV vaccination schedule, meta-analyses have reported that between 25-50% will respond to an additional booster dose, while between 44-100% will respond to a repeat 3-dose vaccine series.[24]

Double dose vs. standard dose HBV vaccination in IBD patients

PICO: Should double dose accelerated schedule vs. standard dose and schedule for HBV vaccination be used in IBD patients?

There is no RCT to address this question. Two observational studies (1 conducted in Spain and 1 in Turkey) compared double dose vs. standard dose HBV vaccination.[25,26] One study included patients with a variety of autoimmune conditions (15 patients with IBD).[26] The other study included only IBD patients.[25] Two cohort studies conducted in Spain assessed serological response to double dose HBV vaccination in IBD patients without a comparison group.[21,22] There was inconsistency in the results with one study suggesting no difference in serological response between double dose vs. standard dose HBV vaccination administered as per standard schedule in patients with autoimmune conditions, and the other study suggesting higher serological response with accelerated schedule of double dose HBV vaccination in IBD patients.[25,26] Nevertheless, both cohort studies with no comparison group suggested that the serological response was still low with accelerated schedule of double dose HBV vaccination in IBD patients.[21,22] The GRADE rating started at low due to the observational nature of these studies. The rating was downgraded to very low due to study limitations, indirectness (surrogate outcomes) and imprecision. **In summary, there is <u>very low</u> certainty evidence that double dose HBV vaccination is associated with a higher or comparable serological response as standard dose HBV vaccination in IBD patients.**

Check titers periodically and administer booster doses as required in IBD patients who responded to HBV vaccination?

There is no RCT or observational studies that addressed this question (comparing measuring vs. not measuring anti-HBs titer periodically and giving vs. not giving booster doses when anti-HBs titer is low).

One prospective observational study included 100 IBD patients who responded to HBV vaccination (anti-HBs > 10 IU/L at 1 – 3 months). The anti-HBs titers were measured at 6 and 12 months.[27] The cumulative incidence of loss of anti-HBs titer was 2% after 6 months and 15% at 12 months.[27] The incidence rate of loss of protective anti-HBs titers was 18% per patient-year. Treatment with anti-TNFs was associated with a higher risk of loss of anti-HBs (HR 3.1, 95% CI 1.1-8.8).[27] In another prospective observational study that included 99 IBD patients starting ani-TNF with previous effective vaccination (anti-HBs > 100 IU/L), 90% maintained titers 4 months after the beginning of anti-TNF treatment, and 81% of patients maintained the titers after a mean follow-up of 29 months.[22] Yet, the clinical significance of loss of anti-HBs titers in patients with IBD is unknown. Anti-HBs titers frequently become undetectable over time in healthy persons. A number of long-term studies performed in different epidemiological contexts have confirmed that clinical HBV infection rarely occurs among successfully vaccinated people, even though anti-HBs titers decline to < 10 IU/L. Therefore, protection against breakthrough HBV infection may be dependent on immunologic memory rather than on anti-HBs levels. However, clinically significant HBV infection has been documented in immunocompromised responders (HIV and those underlying hemodialysis) who do not maintain anti-HBs concentration > 10 IU/L**.**[28] Therefore, the

		CDC recommends annual anti-HBs testing for these patients and a booster dose be administered when anti-HBs levels decrease to < 10 IU/L. However, for other immunocompromised patients (e.g. IBD), the need for booster is uncertain (risks for contracting HBV may not be as high as patients with HIV or on hemodialysis; no study or report on the risks of breakthrough HBV infection in previously vaccinated IBD patients). The GRADE rating started at low due to the observational design of this study. The rating was downgraded to very low due to study limitations (residual confounding, selection bias), indirectness (surrogate outcomes), and imprecision.<br><br>**In summary, there is very low certainty evidence that IBD patients have loss of protective anti-HBs titers over time. Yet, the potential benefits and harms of periodically measuring anti-HBs titers and giving booster when titer is low are highly uncertain. In particular, there are no studies with long term follow-up assessing the safety and effectiveness of repeated administration of booster in patients with autoimmune diseases. It is also uncertain what the target anti-HBs titers should be for IBD patients especially for those who are on immunosuppressive therapies.**	
**Certainty of evidence**	**What is the overall certainty of the evidence of effects?**<br><br>o Very low<br>o Low<br>o Moderate<br>o High<br><br>o No included studies		
**Values and Preferences**	**Is there important uncertainty about or variability in how much people value the main outcomes?**<br><br>o Important uncertainty or variability<br>o Possibly important uncertainty or variability<br>o Probably no important uncertainty or variability<br>o No important uncertainty or variability	Patients likely value patient-important outcomes (mortality, chronic active hepatitis, cirrhosis, hepatocellular cancer, adverse effects) more than surrogate outcomes (immunogenicity).	

**Balance of effects**	**Does the balance between desirable and undesirable effects favor the intervention or the comparison?**<br><br>o Favors the comparison<br>o Probably favors the comparison<br>o Does not favor either the intervention or the comparison<br>o Probably favors the intervention<br>o Favors the intervention<br><br>o Varies<br>o Don't know		
**Resources required**	**How large are the resource requirements (costs)?**<br><br>o Large costs<br>o Moderate costs<br>o Negligible costs and savings<br>o Moderate savings<br>o Large savings<br><br>o Varies<br>o Don't know	CDC vaccine price list last reviewed/updated: July 1, 2019<br><br>**Brandname** / **CDC cost/dose** / **Private sector cost/dose**<br>Engerix-B (adult) / $29.73 / $58.95<br>Engerix B (ped/adolescent) / $16.02 / $23.72<br><br>Engerix-Bis given as a 3-dose vaccine typically at 0, 1, 6 months.	
**Certainty of Evidence of Required Resources**	**What is the certainty of the evidence of resource requirements (costs)?**<br><br>o Very low<br>o Low<br>o Moderate<br>o High<br><br>o No included studies	The costs of delivering routine immunization services may vary widely across countries and different health system settings. See Immunization Costing Action Network (ICAN) Immunization Delivery Cost Catalogue. http://immunizationeconomics.org/ican-idcc.	

Brandname	CDC cost/dose	Private sector cost/dose
Engerix-B (adult)	$29.73	$58.95
Engerix B (ped/adolescent)	$16.02	$23.72

**Cost effectiveness**	**Does the cost-effectiveness of the intervention favor the intervention or the comparison?**<br><br>○ Favors the comparison<br>○ Probably favors the comparison<br>○ Does not favor either the intervention or the comparison<br>○ Probably favors the intervention<br>○ Favors the intervention<br><br>○ Varies<br>○ No included studies	There are no studies that addressed this question specifically in the IBD population.<br><br>A cost-effectiveness study of HBV vaccination strategies found that vaccination (with or without screening) prevents more disease at somewhat increased cost than no vaccination for the neonatal, adolescent and adult populations.[29] Vaccination (with or without screening) is a dominant strategy in adult high-risk populations (those with HBV incidence > 5%; lower cost and greater benefit than no vaccination).[29] When HBV vaccine is administered to all children at age 10 and again 10 years later (incremental cost-per-year-of-life-saved relative to the "no vaccination" strategy is $375).[29] A strategy of universal newborn vaccination alone leads to an incremental cost-per-year-of-life saved of $3332. If adolescents are vaccinated at age 10, incremental cost-per-year-of-life saved is $13,938; for the general adult population, the incremental cost-per-year-of-life saved of universal vaccination is $54,524.[29]<br><br>A cost-effectiveness study of college-based vaccination against HBV and Hepatitis A was performed.[30] The authors developed epidemiologic models to consider infection risks and disease progression and then compared the cost of vaccination with economic, longevity, and quality of life benefits. Immunization of 100,000 students would prevent 1,403 acute cases of hepatitis A, 929 cases of hepatitis B, and 144 cases of chronic hepatitis B. Hepatitis B vaccination would cost the health system $7,600 per quality-adjusted life year (QALY) gained but would reduce societal costs by 6%.[30] HepatitisA/B vaccination would cost the health system $8,500 per QALY but would reduce societal costs by 12%.[30]<br><br>A study amongst a type 1 diabetes population looked at the cost effectiveness of vaccinating non-immune patients 20-59 years of age.[31] Using a 10% uptake rate, the intervention would vaccinate 528,047 people and prevent 4,271 acute and 256 chronic hepatitis B infections.[31] Net health care costs were estimated to increase by $91.4 million, and 1,218 QALYs would be gained, producing a cost-effectiveness ratio of $75,094 per QALY gained.[31] This is a moderately cost-effective strategy. As diabetes is a chronic illness similar to IBD, some parallels can be drawn with this study and the IBD population.	
**Acceptability**	**Is the intervention acceptable to key stakeholders?**<br><br>○ No<br>○ Probably no<br>○ Probably yes<br>○ Yes<br><br>○ Varies<br>○ Don't know	There are no studies that addressed this question specifically in the adult IBD population.	

Feasibility	Is the intervention feasible to implement?<br><br>○ No<br>○ Probably no<br>○ Probably yes<br>○ Yes<br><br>○ Varies<br>○ Don't know		

Conclusion – Adults (double dose vs. standard dose HBV vaccination)

PICO: Should double dose vs. standard dose for HBV vaccination be used in unimmunized adult IBD patients on immunosuppressive therapy?

Direction – Yes (11%), No (22%), Uncertain (67%)

**Type of recommendation**	**Strong recommendation against the intervention**<br>○	**Conditional recommendation against the intervention**<br>○	**Conditional recommendation for either the intervention or the comparison**<br>○	**Conditional recommendation for the intervention**<br>○	**Strong recommendation for the intervention**<br>○
**Recommendation**	**No recommendation – In unimmunized adult patients with IBD on immunosuppressive therapy, the consensus group could not make a recommendation for or against giving double-dose hepatitis B vaccine.**				
**Justification**					

**Subgroup considerations**	
**Implementation considerations**	
**Monitoring and evaluation**	• Ongoing monitoring of safety and effectiveness of the HBV vaccine in IBD populations is needed.
**Research priorities**	• Population-based studies to assess the effectiveness, safety, and duration of protection of HBV vaccine in IBD patients, especially those who are on immunosuppressants. Outcomes should include serologic response as well as patient-important outcomes.<br>• RCT is needed to determine the effectiveness, safety, and serological response of double dose standard or accelerated schedule vs. standard dose standard schedule in IBD patients<br>• Research is needed to determine the clinical relevance/importance of waning anti-HBs antibody titer in IBD patients (especially those who are immunocompromised), and the benefits vs risks of periodic monitoring of titers and administration of booster dose.

Influenza

Background

Influenza is an acute respiratory infection caused primarily by influenza A and B viruses. The most common symptoms are fever, myalgia, headache, cough and fever. Most people will recover within a week, but some are at greater risk of more severe complications, such as viral pneumonia, secondary bacterial pneumonia, worsening of underlying chronic respiratory disease, febrile convulsions, Reye’s syndrome, myocarditis, and death.

Influenza occurs globally with an annual attack rate estimated at 5-10% in adults and 20-30% in children. It should be noted that the incidence of influenza is often underreported since the illness may be confused with other viral illnesses and many people with influenza-like illness do not seek medical care or have viral diagnostic testing done.

Both CDC ACIP and NACI recommend routine annual influenza vaccination of all persons aged $\geq$ 6months without contraindications (noting product-specific age indications and contraindications), with particular focus on people at high risk for influenza-related complications or hospitalization, including:[1,2]

- All children aged 6 through 59 months
- All persons aged $\geq$ 50
- Adults and children who have chronic pulmonary or cardiovascular, renal, hepatic, neurologic, hematologic, or metabolic disorders
- Residents of nursing homes and other chronic care facilities
- Persons who are immunosuppressed due to any cause (medical condition or medications)
- Women who are or will be pregnant during the influenza season
- Children and adolescents who are receiving aspiring or salicylate-containing medications and who might be at risk for experiencing Reye syndrome after influenza infection
- Indigenous peoples; and
- Persons who are extremely obese

Guidelines from the European Crohn's and Colitis Organization and the ACG recommend routine influenza vaccination of all IBD patients, including those on immunosuppressants (*very low level evidence, conditional recommendation*).[3,4] Previous studies in IBD populations have found low rates of influenza vaccination (28%), and common reasons cited for non-immunization with influenza vaccines were lack of awareness of increased infection risk, absence of recommendations from the physician, concerns for lack of effectiveness due to immunosuppressive medications, and potential side effects including exacerbation of disease.[5]

The World Health Organization (WHO) recommends that, when available, seasonal quadrivalent influenza vaccines contain the recommended three viruses for the trivalent vaccine as well as the influenza B virus lineage that is not included in the trivalent vaccine. Inactivated seasonal influenza vaccines contain representative strains of the two human influenza A subtypes (H3N2 and H1NA) and either one (for trivalent vaccines) or both (for quadrivalent vaccines) of the two influenza B lineages (Yamagata or Victoria). An age appropriate inactivated influenza vaccine (IIV) or recombinant influenza vaccine (RIV4) or live attenuated influenza

vaccine (LAIV4) is recommended for persons in all risk groups without contraindications by both ACIP and NACI. However, live attenuated influenza vaccine (LAIV) is recommended not be used for immunocompromised persons because of the uncertain but biologically plausible risk for disease attributable to the vaccine virus, the paucity of safety data for LAIV in most of these populations, and the availability of alternative vaccines. Annual vaccination is required because the body's immune response from vaccination diminishes within a year. Also, because influenza viruses change often, the specific strains in the vaccine are reviewed each year by WHO and updated as necessary so that there is the greatest probability of matching circulating viruses.

It is generally accepted that a single inactivated influenza vaccine has a sufficient protective effect in healthy individuals and no booster vaccinations need to be administered. However, in individuals less than 9 years of age who have not previously receive the seasonal influenza require two doses of influenza vaccine. As well, the antibody response after vaccination depends on several factors, including the age of the recipient, prior and subsequent exposure to antigens, and the presence of immune compromising conditions. Serologic testing is not considered necessary before or after receiving the seasonal influenza vaccine.

References:

1. Centers for Disease Control and Prevention. https://www.cdc.gov/flu/professionals/vaccination/index.htm
2. Public Health Agency of Canada. https://www.canada.ca/en/public-health/services/publications/healthy-living/canadian-immunization-guide-statement-seasonal-influenza-vaccine-2018-2019.html#t1
3. Farraye FA, Melmed GY, Lichtenstein GR, Kane SV. ACG Clinical Guideline: Preventive Care in Inflammatory Bowel Disease. Am J Gastroenterol. 2017 Feb;112(2):241-258.
4. Rahier JF, Magro F, Abreu C, Armuzzi A, Ben-Horin S, Chowers Y, Cottone M, de Ridder L, Doherty G, Ehehalt R, Esteve M, Katsanos K, Lees CW, Macmahon E, Moreels T, Reinisch W, Tilg H, Tremblay L, Veereman-Wauters G, Viget N, Yazdanpanah Y, Eliakim R, Colombel JF; European Crohn's and Colitis Organisation (ECCO). Second European evidence-based consensus on the prevention, diagnosis and management of opportunistic infections in inflammatory bowel disease. J Crohns Colitis. 2014 Jun;8(6):443-68.
5. Melmed GY, Ippoliti AF, Papadakis KA, Tran TT, Birt JL, Lee SK, Frenck RW, Targan SR, Vasiliauskas EA. Patients with inflammatory bowel disease are at risk for vaccine-preventable illnesses. Am J Gastroenterol 2006;101(8): 1834-1840.

Risk of Influenza infection in IBD patients

PICO: What is the risk of influenza infection in people with IBD compared to people without IBD?

Summary

Adults

Two observational studies addressed this PICO question.[1,2]

One was a cross-sectional case-control study that used an US administrative database (Nationwide Inpatient Sample) to compare the risks of hospitalization for influenza pneumonia among adult IBD patients vs. non-IBD controls.[1] It is important to note that influenza pneumonia patients treated as outpatients were excluded. After adjusting for various factors including comorbidities, risk factors for pneumonia, as well as patient and hospital characteristics, IBD patients did not demonstrate an increased odd of hospitalization for pneumonia due to influenza virus. However, low income UC patients had an increased odd of hospitalization for pneumonia due to influenza virus (aOR 1.86, CI 1.46-2.37). Mortality during these admissions among IBD patients was not significantly higher than the control population.

The other case-control study used a US commercial administrative database containing inpatient, outpatient, and pharmacy data to assess the risks of influenza and its related complications among adult IBD patients vs. non-IBD controls.[2] This study provided more direct evidence as both outpatients and inpatients (population of interest) treated for influenza infection were captured in this study. After adjusting for health care utilization and comorbid illnesses, IBD patients had an increased risk for influenza infection compared with non-IBD controls (aHR 1.28, CI 1.19-1.37).[2] IBD patients also had significantly more hospitalizations within 30 days of an influenza diagnosis compared with non-IBD controls (5.4% vs. 1.85%, $P < 0.001$).[2] As this study provided more direct evidence than the previous study, the GRADE rating was anchored to this study.

The GRADE rating started at high as it was considered a prognostic study (providing evidence about the likelihood of influenza infection in patients with IBD). The rating was further downgraded to **low** due to study limitations (residual confounding factors, detection bias, admission bias, and misclassification bias). In particular, patients with IBD and respiratory symptoms may be more likely to be tested for, diagnosed with, and admitted for influenza than non-IBD controls, thus creating an overestimate of the risk of influenza among IBD patients. **In summary, there is low certainty evidence that adult IBD patients have an increased risk of influenza infection compared to non-IBD patients.**

Pediatric

Literature search did not identify any study on the risk of influenza infection in pediatric IBD patients.

Risk of Bias Table

Prognostic studies							
**Study**	**Study sample adequately represents the population of interest**	**Study data available adequately represent the study sample (>80% follow-up)**	**Prognostic factor measured in a similar and valid way for all participants**	**Outcome of interest is measured in a similar and valid way for all participants**	**Important potential confounding factors are appropriately accounted for**	**Statistical analysis is appropriate, and all primary outcomes are reported**	**Comments**
Tinsley 2019 (IBD patients)	Study included patients from a US commercial health claims database containing inpatient, outpatient, and pharmacy data on > 50 million participants (large employers and health plans).<br><br>Findings may not be generalizable to Medicare, Medicaid, and uninsured populations.	OK	Data were reliant on administrative diagnoses. Possible misclassification errors due to errors of miscoding, and the codes have not been previously validated.	Data were reliant on administrative diagnoses. Possible misclassification errors due to errors of miscoding, and the codes have not been previously validated.<br><br>Variances in how influenza was diagnosed (e.g. clinical only, laboratory proven, etc).<br><br>Detection bias and admission rate bias: patients with IBD and respiratory	Each IBD patient was matched 1:1 to a non-IBD patient based on age, sex, and date of entry into the cohort.<br><br>Adjusted for health care utilization and comorbid illnesses.<br><br>Possible residual confounding factors: influenza vaccination status, smoking, severity and activity of underlying disease (e.g. IBD patients may be more likely to be admitted or presented to outpatient care than non-IBD patients).	OK	• Case-control study (Jan 2008 to Dec 2011) using the MarketScan Database to assess the incidence of influenza and risk of related complications in **adult IBD patients** vs. non-IBD patients. Nested case-control study to evaluate the effects of IBD medications on influenza risk.<br>• Cases: 140,480 adult IBD patients<br>• Controls:140,480 non-IBD cohort<br>• **Increased risk of influenza among**

				illnesses may be more likely to be tested and admitted for influenza than non-IBD controls, thus creating an overestimate of the incidence of influenza and hospitalization for influenza-related complications among IBD patients.			**IBD patients vs. non-IBD patients (IRR 1.54, 95% CI 1.49-1.63) and (AHR 1.28, 95% CI 1.19-1.37)**<br>• **Higher rate of hospitalizations among IBD patients vs. non-IBD patients (5.4% vs. 1.85%, P < 0.001)**<br>• **Steroids were independently associated with influenza (OR 1.22, 95% CI 1.08-1.38)**
Stobaugh 2013 (US) (IBD patients)	Study included only hospitalized patients, and did not capture influenza infection treated as outpatients. <u>Prevalence-incidence (Neyman) bias</u>: Exclusion of individuals with severe (fatal prior to admission) or mild influenza infection (not requiring admission) may	OK	Data were reliant on administrative discharge diagnoses. Possible <u>misclassification errors</u> due to errors of miscoding, and the codes have not been previously validated.	Data were reliant on administrative discharge diagnoses. Possible <u>misclassification errors</u> due to errors of miscoding, and the codes have not been previously validated.<br><br><u>Detection bias and admission rate bias:</u> patients with	Case-mix adjustment was performed using the updated Elixhauser Agency for Health-care Research and Quality-Web ICD-9-CM comorbidity algorithms, well-described risk factors for pneumonia, as well as patient and hospital characteristics.<br><br><u>Possible residual confounding factors:</u> medication use,	OK	• Cross-sectional case-control study (6-year analysis) on the Nationwide Inpatient Sample to assess the risk of hospitalizations for vaccine preventable pneumonias (Influenza virus) among adult IBD patients vs. non-IBD patients<br>• <u>Cases:</u> All **adult patients** hospitalized with

	result in a systematic error in the estimated association or effect of IBD on the risk of hospitalization for influenza.			IBD and pneumonia may be more likely to be tested and admitted for influenza than controls, thus creating an overestimate of the prevalence of influenza pneumonia among admitted IBD patients.	influenza vaccination status, severity and activity of underlying disease (e.g. sicker IBD patients on immunosuppressives may be more likely to be admitted than less sick IBD patients).		a secondary diagnosis of IBD<br>• Control: random sample of hospitalized adult patients without a primary or secondary diagnosis of IBD<br>• **Increased odds for hospitalization for pneumonias due to influenza virus were seen among UC patients in the bottom quartile of income (1.86, CI 1.46-2.37) vs. non-IBD control.**

AHR: adjusted hazard ratio
IRR: incidence rate ratio

Evidence Profile Table

**Certainty Assessment**								**Summary of Findings**	
Studies	Risk of bias	Inconsistency	Indirectness	Imprecision	Other considerations	Certainty of Evidence	Overall Certainty of evidence	Study Event Rates	Relative Effect (95% CI)
**Mortality – CRITICAL**							⨁⨁⊖⊖ LOW[d]		

1<br>Observational study[1]	Very serious[a]	Not serious	Serious[b]	Not serious	None	⊕⊖⊖⊖<br>VERY LOW		16 deaths during hospitalization for Hib among all patients	No difference in mortality among IBD patients vs. non-IBD control
**VPI (Influenza) - CRITICAL**									
1<br>Observational study[2]	Very serious[c]	Not serious	Not serious	Not serious	None	⊕⊕⊖⊖<br>LOW		Incidence:<br>709.5/100,000 in IBD patients vs. 459.7/100,000 in non-IBD control	**aHR 1.28 (1.19-1.37)**
**VPI (Admission for Influenza) - CRITICAL**									
1<br>observational study[1]	Very serious[a]	Not serious	Serious[b]	Not serious	None	⊕⊖⊖⊖<br>VERY LOW		Prevalence:<br>41.2/100,000 in IBD patients vs. 39.5/100,000 in non-IBD control	CD: aOR 1.08 (0.95-1.23)<br>UC: aOR 1.05 (0.89-1.25)<br>**Low income UC patients (those in the bottom quartile): aOR 1.86 (1.46-2.37)**
1<br>Observational study[2]	Very serious[c]	Not serious	Not serious	Not serious	None	⊕⊕⊖⊖<br>LOW		Incidence:<br>5.4% in IBD patients vs. 1.85% in non-IBD control<br>(P < 0.001)	**IBD patients had significantly more hospitalizations within 30 days of an influenza diagnosis compared with non-IBD controls**

Footnotes:

a. Downgraded two levels for study limitations. Possible residual confounding factors including medication use (e.g. immunosuppressives or biologics), influenza vaccination status, smoking, as well as severity and activity of IBD may over-estimate the risk of hospitalization for influenza virus pneumonia in IBD patients compared to controls. High risk for detection and admission bias as patients with IBD and pneumonia may be more likely to be tested for, diagnosed with, and admitted for influenza virus than controls, thus creating an overestimate of the prevalence of influenza virus pneumonia among admitted IBD patients. Data were reliant on administrative discharge diagnoses. Possible misclassification errors due to errors of miscoding, and the codes have not been previously validated.

b. Downgraded for indirectness. Study included only a highly selected population (hospitalized patients), and did not capture influenza infection treated as outpatients. Hence, the risk of influenza infection among all IBD patients (population of interest) vs. non-IBD patients is unknown.

c. Downgraded two levels for study limitations. Possible residual confounding factors including medication use, influenza vaccination status, smoking, as well as severity and activity of underlying disease may over-estimate the risk of influenza in IBD patients compared to controls. High risk for detection bias as patients with IBD and respiratory symptoms may be more likely to be tested for, diagnosed with, and admitted for influenza than non-IBD controls, thus creating an overestimate of the incidence of influenza and hospitalization for influenza-related complications among IBD patients. Data were reliant on administrative discharge diagnoses. Possible misclassification errors due to errors of miscoding, and the codes have not been previously validated

d. The overall GRADE rating was anchored to the more direct evidence (Tinsley 2019).[2]

References:

1. Stobaugh DJ, Deepak P, Ehrenpreis ED. Hospitalizations for vaccine preventable pneumonias in patients with inflammatory bowel disease: a 6-year analysis of the Nationwide Inpatient Sample. Clin Exp Gastroenterol. 2013 May 6;6:43-9.

2. Tinsley A, Navabi S, Williams ED, Liu G, Kong L, Coates MD, Clarke K. Increased Risk of Influenza and Influenza-Related Complications Among 140,480 Patients With Inflammatory Bowel Disease. Inflamm Bowel Dis. 2019 Jan 10;25(2):369-376.

Effectiveness and Safety of Influenza vaccine in IBD patients

Summary – Pediatric

PICO 13	In pediatric patients with IBD, should vaccination vs. no vaccination against influenza be given?
**Population**	Pediatric patients with IBD
**Intervention**	Vaccination against influenza
**Comparator**	No vaccination against influenza
**Outcome**	Mortality, VPI (influenza infection), SAEs, Immunogenicity

There was no RCT comparing influenza vaccine with placebo or no treatment in pediatric patients with IBD to address this PICO question.

One Cochrane systematic review assessed the effectiveness of inactivated influenza vaccine and included 5 RCTs on 1628 healthy children aged 2 to 16 years.[1] Compared with placebo or no vaccination, inactivated vaccines reduce the risk of influenza in healthy children from 30% to 11% (RR 0.36, 95% CI 0.28-0.48; NNT = 5).[1] The certainty of evidence was **high.**

There are four observational studies that addressed this PICO question using surrogate outcomes of immunogenicity (seroprotection, seroconversion, GMT fold rise in titer) in pediatric IBD patients.[2-5] The assessed vaccines include trivalent inactivated influenza vaccines. In two of these studies, children < 8 or 9 years of age received two doses of the influenza vaccines if they were receiving the influenza vaccine for the first time, or if they had received only one dose during the previous influenza season as their first dose.[2,5] According to the criteria defined by the European Union Committee for Medicinal Products for Human Use (CHMP) for the evaluation of seasonal influenza vaccine immunogenicity in immunocompetent adults aged 18-60 years, at least one of the following serological criteria for Haemagglutination inhibition (HI) antibody response should be achieved: seroprotection (HI titer ≥ 40) > 70% (or > 60% in age > 60), seroconversion (at least a 4-fold increase in titer) > 40% (or > 30% in age > 60), or geometric mean titer (GMT) fold rise > 2.5 (or > 2 in age > 60). For pandemic vaccines, all three of the criteria had to be met.

However, CHMP criteria for serological response to vaccination are based on healthy volunteers aged 18 to 60 years with attenuated strains, thus may not reflect expected rates of clinical protection observed in other populations (e.g. children, older adults, adults with underlying comorbidities, vaccinated immunocompromised populations). Furthermore, methods of standardization of antibody titres are lacking.

The evidence suggests that influenza vaccination can induce seroprotection (33-100% achieving HI titer $\geq$ 40), seroconversion (33-88.9% achieving at least a 4-fold increase in titer), and GMT fold rise (1.5-22.1) in a significant proportion of pediatric IBD patients. The evidence also suggests that pediatric IBD patients can mount appropriate immunologic response to influenza A components as per the CHMP criteria. However, pediatric IBD patients may be less likely to mount appropriate immunologic response to the B component of the influenza vaccine. As influenza A subtypes exhibit greater cross-reactivity, individuals may be more prime to develop immunogenicity due to previous exposure to not only the same, but also to similar influenza A subtypes by vaccination or infection; this may explain the higher rates of immunogenicity against influenza A compared to influenza B. Immunosuppressive medications (e.g. immunomodulators, anti-TNF, steroids) may further reduce the immunologic response to influenza vaccination in pediatric IBD patients, particularly when multiple immunosuppressive medications are used. However, it is uncertain if this reduced immunologic response is clinically relevant/important and would still afford clinical protection. No serious adverse events including disease exacerbation was reported. In one observational study, no increase in health services event rates including hospitalizations and emergency visits in the post-vaccine risk period was found in IBD patients, and there may be evidence for a protective effect of influenza immunization against IBD-related health services use.[6] The GRADE rating started at low due to the observational designs of the studies. The rating was downgraded to **very low** due to study limitations (selection bias, residual confounding), indirectness (surrogate outcomes), and imprecision. In particular, IBD patients who agreed to vaccination were likely to be systematically different than those who did not agree or seek vaccination (healthy vaccinee effect or confounding by indication). This may lead to selection bias confounding the vaccine’s effect on the outcomes (e.g. mortality, infection, adverse events, and even immunologic response).

The evidence for effectiveness was anchored to the general population. As immunogenicity studies suggested that inactivated influenza vaccines may be less immunogenic (and therefore less effective) in pediatric IBD patients (particularly to the B component), the evidence for effectiveness of inactivated influenza vaccine in pediatric IBD patients was downgraded to moderate in certainty.

One systematic review of 6001 articles including RCTs and large population-based epidemiologic studies found inactivated influenza vaccines to be generally safe with rare serious adverse events.[8] Fever and febrile seizures are more common in children than adults.[8]

The certainty of evidence for safety of inactivated influenza vaccine was judged to be high. Five observational studies in pediatric IBD patients found no serious adverse events following the administration of inactivated influenza vaccines.[2-6] **The evidence for safety was anchored to the general population, but was downgraded to moderate when the evidence was applied to pediatric IBD patients due to indirectness (sample sizes in the IBD studies were insufficient to detect rare adverse events).**

Overall, there is moderate certainty evidence that inactivated influenza is safe and effective in pediatric IBD patients.

There was no RCT comparing the effectiveness and safety of single vs. booster influenza vaccination in pediatric IBD patients.

One RCT addressed the question of effectiveness and safety related to timing of influenza vaccination in IBD patients on maintenance infliximab therapy.[7] However, only 16% (22/137) of the participants were pediatric IBD patients. Therefore, this study was included in the adult evidence profile table (not the pediatric evidence profile table). The study suggested no significant difference in immunogenicity (seroprotection and seroconversion) between vaccination given at the time of infliximab infusion vs. midway between infusions. No serious adverse events were reported. If the evidence was included for pediatric populations, the evidence would need to be further downgraded to **very low** due to indirectness (patient populations).

Risk of Bias Table – Pediatric

SR of RCTs		
**Study**	**Quality Assessment**	**Comments**
Jefferson 2018 (Healthy Children under 16)	• High certainty of evidence for the outcome of influenza (laboratory confirmation)<br>• Downgraded to moderate certainty of evidence due to risk of bias (analysis based on studies at high or unclear risk of bias for multiple domains) for the outcome of influenza-like illness (subjective report).	• SR and MA to assess the evidence for influenza vaccination **in healthy children under 16**<br>• Included 41 RCTs (>200,000 people) conducted in US, Western Europe, Russia, and Bangladesh 1984-2013<br>• **Inactivated influenza vaccines significantly reduce the risk of influenza in children aged 2 to 16 years from 30% to 11% (RR 0.36, 95% CI 0.28-0.48, NNT = 5) compared with placebo, high certainty evidence**<br>• **Inactivated influenza vaccines significantly reduce the risk of influenza-like illness from 28% to 20% (RR 0.72, 95% CI 0.65-0.79, NNT = 12), moderate certainty evidence**<br>• Evidence of serious harms was sparse

Agarwal 2012 (Immune-mediated diseases including RA, IBD on Immunosuppressants)	• No risk of bias assessment	• SR of response to routine vaccines (immunogenicity) in patients with immune-mediated diseases on immunosuppressives<br>• **2 studies assessed TIV in children with IBD (Mamula 2007, Lu 2009). IBD patients on combined immunosuppression with immunomodulators and anti-TNF, or anti-TNF alone, post-vaccination GMTs were significantly lower than control, although response rates were similar to controls**

TIV: trivalent inactivated influenza vaccine

RCTs							
**Study**	**Adequate sequence generation**	**Allocation concealment**	**Blinding**	**Incomplete outcome data addressed**	**Free of selective reporting**	**Free of other bias**	**Comments**
Debruyn 2016 (Canada) IBD patients	"randomly assigned" "using a computer-generated list of small, variable blocks"	No	No	OK	OK	Patients with a history of influenza vaccination are more likely to consent to the trial (76%) than those without a history of influenza vaccination. This may reduce the immunologic response, but should not affect the serologic protection.	• 137 subjects with **IBD on maintenance IFX (age 9-60) randomized to 2012/13 inactivated influenza vaccine** at the time of IFX infusion vs. midway between infusions<br>• **22/137 were pediatric patients (16%) – no pediatric subgroup data. Included only for adult evidence profile table.**<br>• **Did not report subgroup data on pediatric patients**<br>• 50% of patients were on concomitant IM<br>• Serologic protection defined as postvaccine titer $\geq$ 1:40, immunologic response was defined by a 4-fold or greater increase between pre- and post -vaccination HAI titers.<br>• Outcomes were assessed at baseline and 3-5 weeks after vaccination<br>• Serologic protection achieved in only 45-80% of IBD patients.<br>• No significant difference for serologic protection / immunologic response between the 2 groups<br>• Vaccine timing relative to IFX infusion does not

						This may also reduce the adverse events rate.	affect the achievement of serologic protection<br>• Concomitant IM decreased odds of achieving serologic protection to H1N1 only (AOR 0.45, 95% CI 0.2-0.9)<br>• Longer duration of IFX decreased odds of mounting an immugenic response to H1N1 only (AOR 0.5, 95% CI 0.3-0.9)<br>• No serious adverse events

Case Control Studies						
**Study**	**Cases and controls similar for risk of exposure (or adjusted adequately for confounders)**	**Methods to determine exposure valid and similar for cases and controls**	**Methods to ascertain outcome of interest valid and similar for cases and controls**	**Missing data**	**Other bias**	**Comments**
Benchimol 2013 (Canada) (IBD patients)	Each IBD case was matched to 5 controls according to gender, provincial administrative health region, date of birth. Did not adjust for disease severity / activity.	Outpatient physician visits and immunization codes were obtained from OHIP database, including influenza immunization by either a physician or nurse practitioner. Compared to self-reported vaccination in 12-19-year-olds, these codes are	Administrative databases (Discharge Abstract Database for Hospitalization, OHIP for ED and outpatient physician visits).<br><br>Safety of influenza vaccine was assessed with the use of Vaccine and Immunization Surveillance in Ontario, an analysis infrastructure created with the use of data from the ICES to monitor vaccine safety and	OK	Washout period surrounding the time of vaccination was asymmetrically divided: 30 days prior to vaccination, and 2 days after. This washout period was discounted from the control period. As the healthy vaccinee effect	• Retrospective case control study among pediatric IBD patients in Ontario (age < 19)<br>• 4916 IBD patients diagnosed between 1999-2010 vs. 21,686 non-IBD controls<br>• Higher immunization rates in IBD patients vs. controls (25.3% vs. 13.2%, P<0.001)<br>• **Safety outcomes (indirect using health services use as proxies of adverse events)**:<br>• **In IBD patients: no increase in health services use (including hospitalizations, ED visits, or physician office visit for any reason) in any risk period after immunization (3-180 days after vaccination) compared with the control period** (end of the risk intervals up to 30

		55% sensitive and 96% specific with positive and negative predictive values of 94% and 68%. **The low sensitivity is due to the availability of influenza vaccination from public health clinics (not captured in OHIP).**	efficacy.<br><br>In IBD cases, all-cause and IBD-related physician visits, hospitalizations, and ED visits were used as health services proxies for adverse events.		has been described as symmetrically distributed around the time of vaccination, this would lead to over-estimation of the risk of health services utilization for the control period.	days before the following influenza vaccination date; from IBD diagnosis up to 30 days before the initial influenza vaccination). Pooled RI of any health services utilization during the risk period 0.95 (0.84-1.07)<br>• **Significantly lower IBD-related health sciences utilization in the combined postvaccine periods compared with the control period (RI 0.81, 95% CI 0.68-0.96)**<br>• **From 15 to 180 days, IBD cases had a lower RI relative to controls (pooled RIR: 0.85, 95% CI 0.74-0.97)**
Debruyn 2012 (Canada) (IBD patients)	Multivariate logistic regression was performed to evaluate the effect of age, IBD type, and vaccine type on immunogenic response and serologic protection. Pre-vaccine disease activity did not impact the relationship between impaired serologic protection and immunosuppression for children with IBD.	OK	OK	OK	Patients who agreed to vaccination were likely to be prognostically different than patients who did not agree. Therefore, the seroprotection or seroresponse could be over-estimated in a "more healthy" patient population.	• Case-control studies of 60 children with IBD and 53 healthy sibling controls (age 2 – 17), most children are > age 9<br>• IBD patients classified into 2 groups: NIS vs. immunosuppressed (steroids, IM, biologics)<br>• 70% IBD patients were on immunosuppressives (2 steroids, 32 AZA, 6MP or MTX, 8 biologic including 2 combination)<br>• All received **2008 inactivated TIV**. Children age < 9 required 2 doses given ≥ 4 weeks apart if first time, or received only 1 dose during the previous season as the 1st dose<br>• Seroprotection HI titer ≥ 1:40 and seroresponse > 4 fold increase in titer post immunization<br>• Outcomes were assessed at base line and at 3 – 5 weeks after vaccine<br>• **No difference in proportion achieving seroresponse between IBD patients vs.**

						**controls**. Seroresponse to H3N2 (70%), H1N1 (72%), B (53%) in IBD patients. However, **a lower proportion of IBD patients developed seroresponse to B vs. controls (53% vs. 81%, P = 0.002**). **No significant difference in seroresponse between NIS vs. immunosuppressed.**<br>• **No difference in proportion achieving seroprotection between IBD patients vs. controls.** Seroprotection to H3N2 (95%), H1N1 (98%), B (85%). **No significant difference in seroprotection between NIS vs. immunosuppressed.** However, a **lower proportion of immunosuppressed vs. NIS IBD patients** achieved seroprotection to B (79% vs. 100%, P = 003)<br>• **CHPA criteria met for both seroresponse and seroprotection in IBD patients**<br>• **Safety outcomes: no significant difference between pre-vaccine vs. post-vaccine disease activity**<br>• **No serious adverse events causally related to vaccine**
Mamula 2007 (US) (IBD patients)	No adjustment for made for disease activity or severity	OK	OK	OK	Patients who agreed to vaccination were likely to be prognostically different than patients who did not agree. Therefore, the seroprotection or seroresponse could be over-	• Case control study with 51 IBD patients vs. 29 healthy controls (age 9 – 17)<br>• Divided into 3 subgroups: Group A IFX and IM (16), Group B IM (20), and Group C anti-inflammatory therapy (14)<br>• Received TIV 2002-2003 or 2003-2004<br>• Seroprotection HI titer > 40<br>• Outcomes were assessed at baseline and 4 weeks post vaccine<br>• **Seroprotection achieved for 2 strains among all IBD patients: 78% H1N1, 90% H3N2, but not B (64%)**<br>• **Statistically significant decrease in**

					estimated in a "more healthy" patient population.	**seroprotection against B in IBD patients when compared with healthy controls (p = 0.0125)**<br>• **Group A (IFX + IM) were less likely to respond to 2 influenza vaccine (H1N1 and B) when compared with healthy controls (p = 0.018 and P = 0.0002)**<br>• Seroprotection achieved for all strains for all groups except Group A (IFX + IM) with 38% against B<br>• **Seroconversion achieved for all strains among all IBD patients: 76% for H1N1, 79% H3N2, and 62% B**<br>• Seroconversion achieved for all strains for all groups except Group A (IFX + IM) with 33% seroconversion for B<br>• **No serious adverse events**<br>• **No change in disease activity**
Romanowska 2010 (Poland) (IBD patients)	Small study. Not adjusted for age, IBD type, disease severity / activity.	OK	OK	OK	Patients who agreed to vaccination were likely to be prognostically different than patients who did not agree. Therefore, the seroprotection or seroresponse could be over-estimated in a "more healthy" patient population.	• Case control study of pediatric patients with IBD (age 6 – 18)<br>• Group A (9) on anti-inflammatory meds such as 5ASA, antibiotics, Group B (21) on 5ASA and an IM (azathioprine, encorton, infliximab) vs Group C (34) healthy children control<br>• Vaccinated with the **"split" type TIV**<br>• Seroprotection anti-HA antibody titer $\geq$ 40. Seroresponse 4-fold increase in anti-HA antibody titers after vaccination<br>• Outcomes were assessed at baseline, 1 and 6 mos<br>• **Seroprotection: > 70% in H1N1 and H3N2 for all groups at 1 and 6 month post vaccination**; **For B stain, only 44.4% Group A and 33.3% Group B at 1 month; only 66.7% Group A and 47.6% Group B at 6 mos post vaccination**.

						• **Seroresponse: > 40% in H1N1 and H3N2 for all groups at 1 and 6 month post vaccination; For B strain, only 28.6% Group B at 1 month and 47.6% Group B at 6 mos. All groups achieved seroresponse at 6 mos**<br>• **GMT titre > 2.5 fold 1 month post-vaccination for H1NA and H3N2. For B, MFI was only 1.5 for Group B at 1 month and 2.4 at 6 mos.**<br>• CHMP criteria fulfilled for H1N1, H3N2 and B at 6 mos.<br>• **Safety outcome: no severe adverse effects**

GMT: geometric mean titers
HA: hemagglutinin antibodies
ICES: Institute for Clinical Evaluate Sciences
IFX: infliximab
MFI: mean fold increase
NA: neuraminidase
NIS: non-immunosuppressed
OHIP: Ontario Health Insurance Plan

Cohort studies							
**Study**	**Valid methods to ascertain exposure**	**Prognostic factors (other than exposure of interest) similar among cohorts – or cohorts were adjusted adequately for confounders**	**Demonstration that outcome of interest was not present at the start of the study**	**Outcome detection methods valid and similar among cohorts**	**Follow-up complete and similar among cohorts**	**Free of other bias**	**Comments**
Lu 2009 (US) (IBD patients0	OK	No adjustment for made for disease activity or severity	OK	OK	OK	Patients who agreed to vaccination were likely to be	• Cohort study of 146 children (age $\geq$ 5) with IBD divided into 3 groups: NIS (20) vs. IS (126). IS were subcategorized as tacrolimus, anti-TNF, IM, and

						prognostically different than patients who did not agree. Therefore, the seroprotection or seroresponse could be over-estimated in a “more healthy” patient population.	steroids only.<br>• All received **TIV**<br>• Seroprotection HI titer $\geq$ 40<br>• Outcomes were assessed at baseline and 3-9 weeks post vaccination<br>• **Seroprotection was achieved for H1N1, H3N2 but not for B (21-80%)**<br>• Statistically significant higher proportion seroprotected in A strains than in B strains irrespective of whether patients were receiving immunosuppressives ($p < 0.02$)<br>• **Proportion of seroprotection was similar between NIS and IS for all 3 strains**. No significant differences in the proportion seroprotected between any medication subgroup and the NIS group, although lower % (21%) responded to B in patients on anti-TNF<br>• **Proportion of seroprotection for H1N1 and H3N2 were similar between historical controls and the NIS/IS group**. Strain B was less immunogenic – 57% controls vs. 42% NIS and 39% IS for seroprotection.<br>• **Significantly lower proportion seroprotection for B among patients on anti-TNF compared to NIS (14% vs. 39%, p = 0.025) who did not**

							**have seroprotective levels at entry**<br>• No difference in post-vaccination GMT between NIS and IS groups for all 3 strains, but steroids had a significantly higher post-vaccination GMT for strain B compared to NIS group and other medication except tacrolimus<br>• **No serious adverse events**<br>• **No significant difference between pre- and post-vaccine disease activity**

HI – hemagglutination-inhibition
IM - immunomodulators
NIS – non-immunosuppressed
TIV – trivalent inactivated influenza vaccine

Evidence Profile Table – Pediatric

Inactivated Influenza Vaccines in the Pediatric IBD Population

**Certainty Assessment**								**Summary of Findings**	
Studies	Risk of bias	Inconsistency	Indirectness	Imprecision	Other considerations	Certainty of Evidence	Overall Certainty of evidence	Study Event Rates	Relative Effect (95% CI)
**VPI (Influenza, assessed by laboratory confirmation) - CRITICAL**							⊕⊕⊕⊖<br>MODERATE		
1 SR of 5 RCTs[1]<br><br>**Healthy children age > 2**	Not serious	Not serious	Serious[a]	Not serious	None	⊕⊕⊕⊖<br>MODERATE		11% vs. 30%<br>(vaccinated vs. controls)	**RR 0.36 (0.28-0.48)**<br>**NNT = 5**

**Immunogenicity (Seroprotection with HI titer > 40) - IMPORTANT**								
4<br>Observational studies[2-5]<br><br>**IBD populations**	Serious[b]	Not serious	Serious[c]	Serious[d]	None	⊕⊖⊖⊖<br>VERY LOW		• See **Summary of observational studies assessing the seroprotection rates of influenza vaccine in Pediatric IBD patients**<br>• Seroprotection rates range from 33.3-100%
**Immunogenicity (Seroconversion with > 4-fold increase in titer between pre- and post-vaccination titers) - IMPORTANT**								
3<br>Observational studies[2-4]<br><br>**IBD populations**	Serious[b]	Not serious	Serious[c]	Serious[d]	None	⊕⊖⊖⊖<br>VERY LOW		• See **Summary of observational studies assessing the seroconversion rates of influenza vaccine in IBD patients**<br>• Seroconversion rates range from 33-88.9%
**Immunogenicity (GMT fold rise > 2.5) - IMPORTANT**								
3<br>Observational studies[3-5]<br><br>**IBD populations**	Serious[b]	Not serious	Serious[c]	Serious[d]	None	⊕⊖⊖⊖<br>VERY LOW		• See **Summary of observational studies assessing the fold increases in GMT of influenza vaccine in IBD patients**<br>• GMT fold rise range from 1.5-22.1
**Adverse events - CRITICAL**								
1 SR of 6001 studies (RCTs, population based epidemiologic studies, and other observational studies)[8]<br><br>**Healthy children**	Not serious	Not serious	Serious[a]	Not serious	None	⊕⊕⊕⊖<br>MODERATE		• Most influenza vaccies are generally safe with rare serious adverse events<br>• Fever and febrile seizures are more common in children than adults
5<br>Observational studies[2-6]<br><br>**IBD populations**	Serious[b]	Not serious	Not serious	Serious[d]	None	⊕⊖⊖⊖<br>VERY LOW		• No serious adverse events or vaccine induced disease exacerbation

Footnotes:

a. Downgraded for indirectness. Immunogenicity studies suggest that pediatric IBD patients can mount appropriate immunologic response to influenza A components as per the CHMP criteria. However, pediatric IBD patients may be less likely to mount appropriate immunologic response to the B component of the influenza vaccine. Sample sizes in the IBD studies were insufficient to detect rare adverse events.
b. Downgraded for study limitations. IBD patients who agreed to vaccination were likely to be systematically different than those who did not agree or seek vaccination (healthy vaccinee effect or confounding by indication). This may lead to selection bias confounding the vaccine's effect on the outcomes (e.g. mortality, infection, and even immunologic response).
c. Downgraded for indirectness. Surrogate outcomes were used to estimate clinical effectiveness of influenza vaccine. CHMP criteria for serological response to vaccination are based on healthy volunteers aged 18 to 60 years with attenuated strains, thus may not reflect expected rates of clinical protection observed in other populations (e.g. children, vaccinated immunocompromised populations etc.).

d. Small sample size with varying subgroups of patients on no medications or different combinations of medications.
e. Downgraded for study limitations. Lack of standardized approaches to the definition, ascertainment, and reporting of adverse events in trials.

Summary of observational studies assessing the seroprotection rates (HI titer ≥ 1:40) of influenza vaccine in Pediatric IBD patients

Study	Age group	Number of patients	Types of vaccine	Weeks post vaccination	H1N1	H3N2	B
Debruyn 2012	2-17	60 IBD<br>53 controls	TIV<br>2008	3-5	IS: 100%<br>No IS: 94%<br>Control: 98%	IS: 95%<br>No IS: 94%<br>Control: 96%	IS: 79%<br>No IS:100%<br>Control: 94%
Romanowska 2010	6-18	30 IBD<br>34 controls	TIV split type	4	No IS: 77.8%<br>IM: 76.2%<br>Control: 82.4%	No IS: 88.9%<br>IM: 85.7%<br>Control: 94.1%	No IS: 44.4%<br>IM:33.3%<br>Control: 76.5%
Mamula 2007	9-17	51 IBD<br>29 controls	TIV<br>2002/2003<br>2003/2004	4	Total IBD: 78%<br>No IS: 86%<br>IM: 85%<br>IFX + IM: 63%<br>Control: 97%	Total IBD: 90%<br>No IS: 93%<br>IM: 85%<br>IFX + IM: 94%<br>Control: 100%	Total IBD: 64%<br>No IS: 85%<br>IM: 70%<br>IFX + IM: 38%<br>Control: 90%
Lu 2009	5-26<br>Mean age: 14.5	146 IBD<br>No IS: 19<br>IS: 118	TIV	3-9	No IS: 100%<br>IS: 95%	No IS: 84%<br>IS: 89%	No IS: 42%<br>IS: 39%

IFX: infliximab
IM: immunomodulators
IS: immunosuppressants including steroids, immunomodulators, or biologics

European Union Committee for Medicinal Products for Human Use (CHMP) criteria (Hi titer ≥ 40) > 70% for the evaluation of seasonal influenza vaccine immunogenicity met (yellow shading)

Summary of observational studies assessing the seroconversion rates (≥ 4-fold increase in titer) of influenza vaccine in IBD patients

Study	Age group	Number of patients	Types of vaccine	Weeks post vaccination	H1N1	H3N2	B

Debruyn 2012	2-17	60 IBD<br>53 controls	TIV<br>2008	3-5	IS: 76%<br>No IS: 61%<br>Control: 76%	IS: 71%<br>No IS: 67%<br>Control: 83%	IS: 55%<br>No IS: 50%<br>Control: 81%
Romanowska 2010	6-18	30 IBD<br>34 controls	TIV split type	4	No IS: 77.8%<br>IM: 76.2%<br>Control: 61.8%	No IS: 88.9%<br>IM: 76.2%<br>Control: 50.0%	No IS: 66.7%<br>IM: 47.6%<br>Control: 58.8%
Mamula 2007	9-17	51 IBD<br>29 controls	TIV<br>2002/2003<br>2003/2004	4	Total IBD: 76%<br>No IS: 83%<br>IM: 83%<br>IFX + IM: 63%<br>Control: 95%	Total IBD: 79%<br>No IS: 83%<br>IM: 75%<br>IFX + IM: 83%<br>Control: 100%	Total IBD: 62%<br>No IS: 85%<br>IM: 68%<br>IFX + IM: 33%<br>Control: 89%

IFX: infliximab
IM: immunomodulators
IS: immunosuppressants
TIV: trivalent influenza vaccine

European Union Committee for Medicinal Products for Human Use (CHMP) criteria (at least a 4-fold increase in titer) > 40% for the evaluation of seasonal influenza vaccine immunogenicity met (yellow shading)

Summary of observational studies assessing the fold increases in GMT of influenza vaccine in IBD patients

Study	Age group	Number of patients	Types of vaccine	Weeks post vaccination	H1N1	H3N2	B
Romanowska 2010	6-18	30 IBD<br>34 controls	TIV split type	4	No IS: 8.6<br>IM: 5.0<br>Control: 4.7	No IS: 21.8<br>IM: 5.6<br>Control: 4.4	No IS: 2.9<br>IM: 1.5<br>Control: 3.8
Mamula 2007	9-17	51 IBD<br>29 controls	TIV<br>2002/2003<br>2003/2004	4	Total IBD: -<br>No IS: 22.1<br>IM: 24.8<br>IFX + IM: 8.8<br>Control: 29.1	Total IBD: -<br>No IS: 3.3<br>IM: 7.9<br>IFX + IM: 2.9<br>Control: 3.0	Total IBD: -<br>No IS: 9.9<br>IM: 6.9<br>IFX + IM: 2.1<br>Control: 9.6
Lu 2009	5-26<br>Mean age: 14.5	146 IBD<br>No IS: 19<br>IS: 118	TIV	3-9	No IS: 5.9<br>IS: 4.4	No IS: 2.6<br>IS: 2.8	No IS: 1.8<br>IS: 1.8

GMT: geometric mean titer
IFX: infliximab

IM: immunomodulators
IS: immunosuppressants
TIV: trivalent influenza vaccine

European Union Committee for Medicinal Products for Human Use (CHMP) criteria (geometric mean fold rise > 2.5) for the evaluation of seasonal influenza vaccine immunogenicity met (yellow shading)

References:

1. Jefferson T, Rivetti A, Di Pietrantonj C, Demicheli V. Vaccines for preventing influenza in healthy children. Cochrane Database Syst Rev. 2018 Feb 1;2:CD004879.
2. deBruyn JC, Hilsden R, Fonseca K, Russell ML, Kaplan GG, Vanderkooi O, Wrobel I. Immunogenicity and safety of influenza vaccination in children with inflammatory bowel disease. Inflamm Bowel Dis. 2012 Jan;18(1):25-33.
3. Romanowska M1, Banaszkiewicz A, Nowak I, Radzikowski A, Brydak LB. Immunization against influenza during the 2005/2006 epidemic season and the humoral response in children with diagnosed inflammatory bowel disease (IBD). Med Sci Monit. 2010 Sep;16(9):CR433-9.
4. Mamula P, Markowitz JE, Piccoli DA, Klimov A, Cohen L, Baldassano RN. Immune response to influenza vaccine in pediatric patients with inflammatory bowel disease. Clin Gastroenterol Hepatol. 2007 Jul;5(7):851-6.
5. Lu Y, Jacobson DL, Ashworth LA, Grand RJ, Meyer AL, McNeal MM, Gregas MC, Burchett SK, Bousvaros A. Immune response to influenza vaccine in children with inflammatory bowel disease. Am J Gastroenterol. 2009 Feb;104(2):444-53.
6. Benchimol EI, Hawken S, Kwong JC, Wilson K. Safety and utilization of influenza immunization in children with inflammatory bowel disease. Pediatrics. 2013 Jun;131(6):e1811-20.
7. deBruyn J, Fonseca K, Ghosh S, Panaccione R, Gasia MF, Ueno A, Kaplan GG, Seow CH, Wrobel I. Immunogenicity of Influenza Vaccine for Patients with Inflammatory Bowel Disease on Maintenance Infliximab Therapy: A Randomized Trial. Inflamm Bowel Dis. 2016 Mar;22(3):638-47.
8. Halsey NA, Talaat KR, Greenbaum A, Mensah E, Dudley MZ, Proveaux T, Salmon DA. The safety of influenza vaccines in children: An Institute for Vaccine Safety white paper. Vaccine. 2015 Dec 30;33 Suppl 5:F1-F67.

Evidence to Decision Table – Pediatric

**PICO 13**	**In pediatric patients with IBD, should vaccination vs. no vaccination against influenza be given?**
**Population**	Pediatric patients with IBD
**Intervention**	Vaccination against influenza
**Comparator**	No vaccination against influenza
**Outcome**	Mortality, VPI (influenza infection), SAEs, Immunogenicity

	Judgement	Research evidence	Additional considerations
**Desirable Effects**	**How substantial are the desirable anticipated effects?**<br><br>○ Trivial<br>○ Small<br>○ Moderate<br>○ Large<br><br>○ Varies<br>○ Don't know	See Evidence Profile Tables.<br><br>**Risk of Influenza infection in pediatric IBD patients**<br><br>Literature search did not identify any study on the risk of influenza infection in pediatric IBD patients.<br><br>**Effectiveness and safety of Inactivated Influenza Vaccine in pediatric IBD patients**<br><br>There was no RCT comparing influenza vaccine with placebo or no treatment in pediatric patients with IBD to address this PICO question.<br><br>One Cochrane systematic review assessed the effectiveness of inactivated influenza vaccine and included 5 RCTs on 1628 healthy children aged 2 to 16 years.[1] Compared with placebo or no vaccination, inactivated vaccines reduce the risk of influenza in healthy children from 30% to 11% (RR 0.36, 95% CI 0.28-0.48; NNT = 5).[1] The certainty of evidence was high.<br><br>There are four observational studies that addressed this PICO question using surrogate outcomes of immunogenicity (seroprotection, seroconversion, GMT fold rise in titer) in pediatric IBD patients.[2-5] The assessed vaccines include trivalent inactivated influenza vaccines. In two of these studies, children < 8 or 9 years of age received two doses of the influenza vaccines if they were receiving the influenza vaccine for the first time, or if they had received only one dose during the previous influenza season as their first dose.[2,5] According to the criteria defined by the European Union Committee for Medicinal Products for Human Use (CHMP) for the evaluation of seasonal influenza vaccine immunogenicity in immunocompetent adults aged 18-60 years, at least one of the following serological criteria for Haemagglutination inhibition (HI) antibody response should be achieved: seroprotection (HI titer $\geq$ 40) > 70% (or > 60% in age > 60), seroconversion (at least a 4-fold increase in titer) > 40% (or > 30% in age > 60), or geometric mean titer (GMT) fold rise > 2.5 (or > 2 in age > 60). For pandemic vaccines, all three of the criteria had to be met. However, CHMP criteria for serological response to vaccination are based on healthy volunteers aged 18 to 60 years with attenuated strains, thus may not reflect expected rates of clinical protection observed in other populations (e.g. children, older adults, adults with underlying comorbidities, vaccinated immunocompromised populations). Furthermore, methods of standardization of antibody titres are lacking.<br><br>The evidence suggests that influenza vaccination can induce seroprotection (33-100% achieving HI titer $\geq$ 40), seroconversion (33-88.9% achieving at least a 4-fold increase in titer), and GMT fold rise (1.5-22.1) in a significant proportion of pediatric IBD patients.	
**Undesirable Effects**	**How substantial are the undesirable anticipated effects?**<br><br>○ Large<br>○ Moderate<br>○ Small<br>○ Trivial<br><br>○ Varies<br>○ Don't know		

		The evidence also suggests that pediatric IBD patients can mount appropriate immunologic response to influenza A components as per the CHMP criteria. However, pediatric IBD patients may be less likely to mount appropriate immunologic response to the B component of the influenza vaccine. As influenza A subtypes exhibit greater cross-reactivity, individuals may be more prime to develop immunogenicity due to previous exposure to not only the same, but also to similar influenza A subtypes by vaccination or infection; this may explain the higher rates of immunogenicity against influenza A compared to influenza B. Immunosuppressive medications (e.g. immunomodulators, anti-TNF, steroids) may further reduce the immunologic response to influenza vaccination in pediatric IBD patients, particularly when multiple immunosuppressive medications are used. However, it is uncertain if this reduced immunologic response is clinically relevant/important and would still afford clinical protection. No serious adverse events including disease exacerbation was reported. In one observational study, no increase in health services event rates including hospitalizations and emergency visits in the post-vaccine risk period was found in IBD patients, and there may be evidence for a protective effect of influenza immunization against IBD-related health services use.[6] The GRADE rating started at low due to the observational designs of the studies. The rating was downgraded to very low due to study limitations (selection bias, residual confounding), indirectness (surrogate outcomes), and imprecision. In particular, IBD patients who agreed to vaccination were likely to be systematically different than those who did not agree or seek vaccination (healthy vaccinee effect or confounding by indication). This may lead to selection bias confounding the vaccine's effect on the outcomes (e.g. mortality, infection, adverse events, and even immunologic response).<br><br>**The evidence for effectiveness was anchored to the general population. As immunogenicity studies suggested that inactivated influenza vaccines may be less immunogenic (and therefore less effective) in pediatric IBD patients (particularly to the B component), the evidence for effectiveness of inactivated influenza vaccine in pediatric IBD patients was downgraded to moderate in certainty.**<br><br>One systematic review of 6001 articles including RCTs and large population-based epidemiologic studies found inactivated influenza vaccines to be generally safe with rare serious adverse events.[8] Fever and febrile seizures are more common in children than adults.[8] The certainty of evidence for safety of inactivated influenza vaccine was judged to be high. Five observational studies in pediatric IBD patients found no serious adverse events following the administration of inactivated influenza vaccines.[2-6] **The evidence for safety was anchored to the general population, but was downgraded to <u>moderate</u> when the evidence was applied to pediatric IBD patients due to indirectness (sample sizes in the IBD studies were insufficient to detect rare adverse events).**<br><br>**Overall, there is <u>moderate</u> certainty evidence that inactivated influenza is safe and effective in pediatric IBD patients.**<br><br>There was no RCT comparing the effectiveness and safety of single vs. booster influenza vaccination in pediatric IBD patients.	

		One RCT addressed the question of effectiveness and safety related to timing of influenza vaccination in IBD patients on maintenance infliximab therapy.[7] However, only 16% (22/137) of the participants were pediatric IBD patients. Therefore, this study was included in the adult evidence profile table (not the pediatric evidence profile table). The study suggested no significant difference in immunogenicity (seroprotection and seroconversion) between vaccination given at the time of infliximab infusion vs. midway between infusions. No serious adverse events were reported. If the evidence was included for pediatric populations, the evidence would need to be further downgraded to **very low** due to indirectness (patient populations).	
**Certainty of evidence**	**What is the overall certainty of the evidence of effects?**<br><br>○ Very low<br>○ Low<br>○ Moderate<br>○ High<br><br>○ No included studies		
**Values and Preferences**	**Is there important uncertainty about or variability in how much people value the main outcomes?**<br><br>○ Important uncertainty or variability<br>○ Possibly important uncertainty or variability<br>○ Probably no important uncertainty or variability<br>○ No important uncertainty or variability	Patients likely value patient-important outcomes (mortality, VPI, adverse effects) more than surrogate outcomes (immunogenicity).	
**Balance of effects**	**Does the balance between desirable and undesirable effects favor the intervention or the comparison?**<br><br>○ Favors the comparison<br>○ Probably favors the comparison<br>○ Does not favor either the intervention or the comparison<br>○ Probably favors the intervention<br>○ Favors the intervention<br><br>○ Varies<br>○ Don't know		

<table>
<tr><td>Resources required</td><td>How large are the resource requirements (costs)?

o Large costs
o Moderate costs
o Negligible costs and savings
o Moderate savings
o Large savings

o Varies
o Don't know</td><td>CDC vaccine price list last reviewed/updated: July 1, 2019

<table><tr><th>Brandname</th><th>CDC cost/dose</th><th>Private sector cost/dose</th></tr><tr><td>Fluzone® Quadrivalent</td><td>$12.808</td><td>$16.939</td></tr><tr><td>Fluarix® Quadrivalent</td><td>$12.22</td><td>$16.82</td></tr><tr><td>FluLaval® Quadrivalent</td><td>$11.94</td><td>$15.77</td></tr><tr><td>Flucelvax® Quadrivalent</td><td>$15.00</td><td>$22.758</td></tr><tr><td>Afluria® Quadrivalent</td><td>$11.35</td><td>$15.871</td></tr></table></td><td></td></tr>
<tr><td>Certainty of Evidence of Required Resources</td><td>What is the certainty of the evidence of resource requirements (costs)?

o Very low
o Low
o Moderate
o High

o No included studies</td><td>The costs of delivering routine immunization services may vary widely across countries and different health system settings. See Immunization Costing Action Network (ICAN) Immunization Delivery Cost Catalogue. http://immunizationeconomics.org/ican-idcc.</td><td></td></tr>
<tr><td>Cost effectiveness</td><td>Does the cost-effectiveness of the intervention favor the intervention or the comparison?

o Favors the comparison
o Probably favors the comparison
o Does not favor either the intervention or the comparison
o Probably favors the intervention
o Favors the intervention

o Varies
o No included studies</td><td>There are no studies that addressed this question specifically in the IBD population.

In a systematic review of economic evaluations of childhood influenza vaccination, the majority of the studies found that childhood influenza vaccination was cost effective.[9] The studies differed widely in terms of costs and benefits that were included and the methodologies used.

In another systematic review of the cost-effectiveness of influenza immunization programs with inclusion of 41 studies, vaccinating all versus only high-risk children found vaccinating all to be dominant (less costly and more effective) to $47,000 per QALY gained (societal), and dominant to $18,000 per QALY gained (healthcare system).[10]</td><td></td></tr>
</table>

**Acceptability**	**Is the intervention acceptable to key stakeholders?**<br><br>o No<br>o Probably no<br>o Probably yes<br>o Yes<br><br>o Varies<br>o Don't know		
**Feasibility**	**Is the intervention feasible to implement?**<br><br>o No<br>o Probably no<br>o Probably yes<br>o Yes<br><br>o Varies<br>o Don't know		

References:

1. Jefferson T, Rivetti A, Di Pietrantonj C, Demicheli V. Vaccines for preventing influenza in healthy children. Cochrane Database Syst Rev. 2018 Feb 1;2:CD004879.
2. deBruyn JC, Hilsden R, Fonseca K, Russell ML, Kaplan GG, Vanderkooi O, Wrobel I. Immunogenicity and safety of influenza vaccination in children with inflammatory bowel disease. Inflamm Bowel Dis. 2012 Jan;18(1):25-33.
3. Romanowska M1, Banaszkiewicz A, Nowak I, Radzikowski A, Brydak LB. Immunization against influenza during the 2005/2006 epidemic season and the humoral response in children with diagnosed inflammatory bowel disease (IBD). Med Sci Monit. 2010 Sep;16(9):CR433-9.
4. Mamula P, Markowitz JE, Piccoli DA, Klimov A, Cohen L, Baldassano RN. Immune response to influenza vaccine in pediatric patients with inflammatory bowel disease. Clin Gastroenterol Hepatol. 2007 Jul;5(7):851-6.
5. Lu Y, Jacobson DL, Ashworth LA, Grand RJ, Meyer AL, McNeal MM, Gregas MC, Burchett SK, Bousvaros A. Immune response to influenza vaccine in children with inflammatory bowel disease. Am J Gastroenterol. 2009 Feb;104(2):444-53.
6. Benchimol EI, Hawken S, Kwong JC, Wilson K. Safety and utilization of influenza immunization in children with inflammatory bowel disease. Pediatrics. 2013 Jun;131(6):e1811-20.
7. deBruyn J, Fonseca K, Ghosh S, Panaccione R, Gasia MF, Ueno A, Kaplan GG, Seow CH, Wrobel I. Immunogenicity of Influenza Vaccine for Patients with Inflammatory Bowel Disease on Maintenance Infliximab Therapy: A Randomized Trial. Inflamm Bowel Dis. 2016 Mar;22(3):638-47.
8. Halsey NA, Talaat KR, Greenbaum A, Mensah E, Dudley MZ, Proveaux T, Salmon DA. The safety of influenza vaccines in children: An Institute for Vaccine Safety white paper. Vaccine. 2015 Dec 30;33 Suppl 5:F1-F67.

9. Newall AT, Jit M, Beutels P. Economic evaluations of childhood influenza vaccination: a critical review. Pharmacoeconomics. 2012 Aug 1;30(8):647-60.
10. Ting EEK, Sander B, Ungar WJ. Systematic review of the cost-effectiveness of influenza immunization programs. Vaccine. 2017 Apr 4;35(15):1828-1843

Conclusion – Pediatric

PICO 13: In pediatric patients with IBD, should vaccination vs. no vaccination against influenza be given?
Moderate certainty of evidence
Direction – Yes (100%)
Strength – Strong (100%)

**Type of recommendation**	**Strong recommendation against the intervention**	**Conditional recommendation against the intervention**	**Conditional recommendation for either the intervention or the comparison**	**Conditional recommendation for the intervention**	**Strong recommendation for the intervention**
	○	○	○	○	○
**Recommendation**	**Statement 13: In pediatric patients with IBD, we recommend influenza vaccine be given.**				
**Justification**					
**Subgroup considerations**					
**Implementation considerations**					
**Monitoring and evaluation**	• Ongoing monitoring of serious adverse events associated with influenza vaccine in IBD patients				

**Research priorities**	• Observational or RCTs to determine the clinical effectiveness of influenza vaccine in pediatric IBD patients with assessment of patient-important outcomes (i.e. influenza, influenza-like illness etc.)<br>• More RCTs are needed to compare single vs. booster vaccination strategies in pediatric IBD patients on immunosuppressive medications (particularly for the B component)<br>• RCTs are needed to compare standard vs. high dose influenza vaccine products in pediatric IBD patients on immunosuppressive medications (particularly for the B component)

Summary – Adults

**PICO 14A**	**In adult patients with IBD (65 years of age and younger), should vaccination vs. no vaccination against influenza be given?**
**Population**	Adult patients with IBD **(65 years of age and younger)**
**Intervention**	Vaccination against influenza
**Comparator**	No vaccination against influenza
**Outcome**	Mortality, VPI (influenza infection), SAEs, Immunogenicity

**PICO 14B**	**In adult patients with IBD (older than 65 years of age), should vaccination vs. no vaccination against influenza be given?**
**Population**	Adult patients with IBD with **(older than 65 years of age)**
**Intervention**	Vaccination against influenza
**Comparator**	No vaccination against influenza
**Outcome**	Mortality, VPI (influenza infection), SAEs, Immunogenicity

There was no RCT comparing influenza vaccine with placebo or no treatment in adult patients with IBD to address these 2 PICO questions.

There are 2 Cochrane systematic reviews assessing the safety and effectiveness of inactivated influenza vaccine in healthy adults aged 16-65 and in the elderly aged > 65.[1,2] In **healthy adult**s, inactivated influenza vaccines reduce the risk of influenza (RR 0.41, 95% CI 0.36-0.47; NNT = 71) and influenza-like illness (RR 0.84, 95% CI 0.75-0.95; NNT = 29).[1] The certainty of evidence was **moderate** for the outcome of influenza due to indirectness for outcome with uncertainty over definition, surveillance and testing of influenza in older trials.[1] The certainty of evidence was also **moderate** for the outcome of influenza-like illness due to inconsistency. In **elderly aged > 65**, inactivated influenza vaccines also reduce the risk of influenza (RR 0.42, 95% CI 0.27-0.66; NNT = 30) and influenza-like illess (RR 0.59, 95% CI 0.47-0.73; NNT = 42).[2] However, the certainty of evidence was l**ow** for influenza due to indirectness of outcome and study limitations with most of the evidence coming from studies with high or unclear risk of bias for more than one risk of bias domain, but **moderate** for influenza-like illness due to study limitations.[2]

There are six cohort studies and four RCTs (observational data) that addressed this PICO question using surrogate outcomes of immunogenicity (seroprotection, seroconversion, GMT fold rise in titer) in adult IBD patients.[3-12] The assessed vaccines include trivalent and quadrivalent inactivated influenza vaccines, as well as the 2009 pandemic H1N1 vaccine. According to the criteria defined by the European Union Committee for Medicinal Products for Human Use (CHMP) for the evaluation of seasonal influenza vaccine immunogenicity in immunocompetent adults aged 18-60 years, <u>at least one</u> of the following serological criteria for Haemagglutination inhibition (HI) antibody response should be achieved: seroprotection (HI titer $\geq$ 40) > 70% (or > 60% in age > 60), seroconversion (at least a 4-fold increase in titer) > 40% (or > 30% in age > 60), or geometric mean titer (GMT) fold rise > 2.5 (or > 2 in age > 60). For pandemic vaccines, all three of the criteria had to be met. However, CHMP criteria for serological response to vaccination are based on healthy volunteers aged 18 to 60 years with attenuated strains, thus may not reflect expected rates of clinical protection observed in other populations (e.g. children, older adults, adults with underlying comorbidities, vaccinated immunocompromised populations). Furthermore, methods of standardization of antibody titres are lacking.

The evidence suggests that influenza vaccination can induce seroprotection (43-100% achieving HI titer $\geq$ 40), seroconversion (23-76% achieving at least a 4-fold increase in titer), and GMT fold rise (1.77-20.4) in a significant proportion of adult IBD patients. Immunosuppressive medications (e.g. immunomodulators, anti-TNF, steroids) may reduce the immunologic response to influenza vaccination in IBD patients, particularly when multiple immunosuppressive medications are used. However, it is uncertain if this reduced immunologic response is clinically relevant/important and would still afford clinical protection. The GRADE rating started at low due to the observational designs of the studies. The rating was downgraded to **very low** due to study limitations (selection bias, residual confounding), indirectness (surrogate outcome), inconsistency, and imprecision. In particular, IBD patients who agreed to vaccination were likely to be systematically different than those who did not agree or seek vaccination (healthy vaccinee effect or

confounding by indication). This may lead to selection bias confounding the vaccine's effect on the outcomes (e.g. mortality, infection, adverse events, and even immunologic response). As well, the response to influenza vaccine was quite variable, and comparisons between different studies are difficult to make because of annual reformulation of vaccine resulting in differing antigenicity of each year's vaccine. Protective effect of a vaccine is also dependent on whether the circulating strain is included in the vaccine. Additionally, in some years, strains can remain unchanged from previous years. Thus, a study participant who had been vaccinated against the same strain the previous year might have a different response than a participant naïve to that strain (depending on pre-vaccination titers). Patient populations were also quite variable across studies with different proportions of patients being on different immunosuppressive medications. This may have contributed to the inconsistency in results across studies. It is important to note that most patients included in the studies were age 18-65. Very few elderly patients (age > 65) were included.

The evidence for effectiveness was anchored to the general population. As immunogenicity studies suggested that inactivated vaccines may be less immunogenic (and therefore less effective) in adult IBD patients, we would also need to need to consider downgrading for indirectness. However, the degree of indirectness was not judged to be severe enough to warrant further downgrading as most studies showed the CHMP criteria for immunogenicity was met in adult IBD populations. Therefore, **the overall certainty of evidence for effectiveness of inactivated influenza vaccine was judged to remain <u>moderate</u> in adult IBD patients aged 16-65 and <u>moderate</u> in elderly IBD patients over age 65.**

The two Cochrane systematic reviews in healthy adults and elderly adults also showed no serious adverse events associated with the use of inactivated influenza vaccine.[1,2] The certainty of evidence for safety was high for healthy adults and for elderly adults.[1,2] No serious adverse events including disease exacerbation was reported in the 10 observational studies in adult IBD patients.[3-12] The certainty of evidence for safety was anchored to the general population, and downgraded for indirectness as sample sizes in the IBD studies were insufficient to detect rare adverse events. **Therefore, the overall certainty of evidence for safety of inactivated influenza vaccine was judged to be <u>moderate</u> in adult IBD patients aged 16-65 and <u>moderate</u> in elderly IBD patients over age 65.**

Overall, there is <u>moderate</u> certainty evidence that inactivated influenza is safe and effective in adult IBD patients aged 16-65 and elderly IBD patients age > 65.

Single vs. booster influenza vaccination:

Two RCTs addressed the question of effectiveness and safety between single vs. booster influenza vaccination in IBD patients.[3,5] Both studies suggested no significant difference in immunogenicity (seroprotection, seroconversion, GMT titer rise) between single vs. booster vaccination strategies in IBD patients. No serious adverse events including disease exacerbation was reported. The GRADE rating started at high. The rating was downgraded to **very low** due to study limitations, indirectness (surrogate outcomes, patient populations), and imprecision. Both studies were conducted in Japan with different formulations of vaccines (2015/2016 seasonal QIV and 2012-2013 seasonal TIV with different vaccine strains). Therefore, results may not be generalizable to other IBD patient populations. As well, surrogate outcomes were used. **In summary, there is very low certainty evidence that there is no significant difference in effectiveness and safety between single vs. booster influenza vaccination in adult IBD patients.**

Timing of influenza vaccination relative to anti-TNF therapy:

One RCT addressed the question of effectiveness and safety related to timing of influenza vaccination in IBD patients on maintenance infliximab therapy.[4] The study suggested no significant difference in immunogenicity (seroprotection and seroconversion) between vaccination given at the time of infliximab infusion vs. midway between infusions. No serious adverse events were reported. The GRADE rating started at high. The rating was downgraded to **low** due to study limitations and imprecision. **In summary, there is low certainty evidence that timing of influenza vaccination relative to infliximab infusion does not affect the effectiveness and safety of influenza in adult IBD patients.**

Risk of Bias Table – Adults

SR of RCTs		
**Study**	**Quality Assessment**	**Comments**
Demicheli 2018 **(Healthy Adults aged 16-65)**	• Most studies were at high or unclear risk of bias for all domains<br>• Downgraded to moderate certainty of evidence due to indirectness (uncertainty over definition, surveillance and testing of influenza in older trials) for the outcome of influenza (laboratory confirmation).<br>• Downgraded to moderate certainty of evidence due	• SR and MA to assess the evidence for influenza vaccination in healthy individuals aged 16-65 years<br>• Included 52 RCTs (>80,000 people) conducted in North America, South America, and Europe 1969-2009<br>• **Inactivated influenza vaccines significantly reduce the risk of influenza from 2.3% to 0.9% (RR 0.41, 95% CI 0.36-0.47, NNT = 71) compared with placebo, moderate certainty evidence**

	to inconsistency (variation in event rates across control arms) for the outcome of influenza-like illness (subjective report)<br>• Industry funding of influenza vaccine studies determines publication in high-prestige journals and higher citations than other types of funding. Industry funding is associated with optimistic conclusions	• **Inactivated influenza vaccines significantly reduce the risk of influenza-like illness from 21.5% to 18.1% (RR 0.84, 95% CI 0.75-0.95, NNT = 29), moderate certainty evidence**<br>• No serious adverse events with vaccines
Demicheli 2018c **(elderly age > 65)**	• Downgraded to low certainty of evidence due to serious risk of bias and indirectness (uncertainty over the definition, testing, and surveillance of influenza in older trials) for the outcome of influenza (laboratory confirmation)<br>• Downgraded to moderate certainty of evidence due to serious risk of bias for the outcome of influenza-like illness (subjective report)<br>• Downgrade to very low certainty of evidence due to serious risk of bias and imprecision for the outcome of mortality<br>• Lack of detail regarding the methods used to confirm the diagnosis of influenza limits the applicability of the results<br>• Insufficient, poor quality, old evidence relating to complications	• SR and MA to assess the evidence for influenza vaccination in elderly individuals aged > 65<br>• Included 8 RCTs (> 5000 people) conducted in community and residential care settings in Europe and US 1965-2000<br>• **Influenza vaccine significantly reduce the risk of influenza from 6% to 2.4% (RR 0.42, 95% CI 0.27-0.66, NNT = 30) compared with placebo, low certainty of evidence**<br>• **Influenza vaccine significantly reduce the risk of influenza-like illness from 6% to 3.5% (RR 0.59, 95% CI 0.47-0.73, NNT = 42) compared with no vaccine, moderate certainty evidence**<br>• **No difference in mortality (RR 1.02, 95% CI 0.11-9.72), very low certainty evidence**<br>• Insufficient data on complications
Agarwal 2012 **(Immune-mediated diseases including RA, IBD on Immunosuppressants)**	• No risk of bias assessment	• SR of response to routine vaccines (immunogenicity) in patients with immune-mediated diseases on immunosuppressives<br>• 3/5 studies of TIV administered to patients with rheumatic diseases showed that vaccine responses were generally robust across all immunosuppressive subgroups (DMARDs, anti-TNF) and appeared to confer at least some degree of immunity (defined as HI > 1:40). Patients in 2 studies still developed seroprotection at equivalent rates as controls, but those receiving rituximab or anti-TNF had a lower GMT as compared to controls.<br>• **2 studies assessed TIV in children with IBD (Mamula 2007, Lu 2009). IBD patients on combined immunosuppression with immunomodulators and anti-TNF, or anti-TNF alone, post-vaccination GMTs were significantly lower than control, although response rates were similar to controls**

TIV: trivalent inactivated influenza vaccine

SR of Observational Studies and RCTs		
**Study**	**Quality Assessment**	**Comments**
Beck 2012 **(Patients with HIV, cancer, transplants, autoimmune diseases - SLE)**	• Majority of studies (n = 137) were non-randomized trials. Small number of RCTs (n = 23)<br>• Most studies were at unclear or high risk of bias across most domains with residual confounding and selection bias<br>• Moderate to high levels of statistical heterogeneity (inconsistency) for most meta-analyses	• SR and MA to assess the evidence for influenza vaccination in immunocompromised patients based on etiology<br>• Significantly lower odds of influenza-like illness after vaccination in patients with HIV, patients with cancer, and transplant recipients compared with patients receiving placebo or no vaccination.<br>• Pooled odds of seroconversion and seroprotection were lower in HIV patients, patients with cancer, and transplant recipients, compared with immunocompetent controls<br>• **5 studies in patients with autoimmune disease treated with immunosuppressants (adult SLE) suggested that influenza vaccination is beneficial resulting in low rates of influenza-like illness, possibly comparable to rates in immunocompetent controls. The serological response (mean geometric mean titer) after vaccination was commonly greater than the CHMP criteria of 2.5, although less than in immunocompetent controls.**<br>• **No IBD patients included**<br>• Vaccination was well tolerated with no serious adverse effects

CHMP: Committee for Human Medicinal Products
MA: meta-analyses
SLE: systemic lupus erythematosus
SR: systematic review

RCTs							
**Study**	**Adequate sequence generation**	**Allocation concealment**	**Blinding**	**Incomplete outcome data addressed**	**Free of selective reporting**	**Free of other bias**	**Comments**
Shirai 2018 (Japan) (IBD patients)	"The participants whose birthdays were on even days	No	Unblinded	"Several patients were diagnosed as serologically infected	OK	OK	• 132 adults with CD or UC on **immunosuppressive therapy**, randomized to **single vaccination** (83) vs. **booster group with QIV** (49)<br>• 32 patients on immunomodulatory monotherapy, 16 anti-TNF monotherapy, 15

	were assigned to the single vaccination group, and those whose birthdays were on odd days to the booster vaccination group”			with influenza and excluded from the analysis after the end of the season”			combination<br>• 27 healthy controls randomized to single vs. booster<br>• Immunogenicity was evaluated (seroconversion rate >40%, mean GMT increase > 2.5 or seroprotection rate > 70%) and FDA criteria (lower limit of 95% CI of seroconversion to exceed 40% and the lower limit of 95% CI of seroprotection to exceed 70%).<br>• Outcomes were assessed at baseline, 4 weeks after vaccination, and after the influenza season in the single vaccination group; and 4 weeks after the second vaccination and after the influenza season in the booster group<br>• **Each vaccine strain showed immunogenicity (European Medicines Agency criteria) with a single inoculation. Booster did not induce additional response.** No significant difference in the GMT, seroconversion, seroprotection rates ($\geq$ 1:40) between single vs. booster after vaccination and after the end of the season. No significant difference in seroprotection between IBD patients vs. healthy controls<br>• Lower immunogenicity (seroprotection and seroconversion rates) in patients on infliximab (serum IFX > 0.1ug/mL compared with patients without biological therapy) (seroprotection: H1N1: OR 0.37, 95% CI 0.11-1.21; H3N2: OR 0.22, 95% CI 0.07-0.68; seroconversion: H1N1: OR 0.23, 95% CI 0.06-0.91; H3N2: OR 0.19, 95% CI 0.06-0.56)<br>• **No serious adverse effects including exacerbation of disease**
Debruyn 2016 (Canada) IBD patients	“randomly assigned” “using a	No	No	OK	OK	Patients with a history of influenza	• 137 subjects with IBD on **maintenance IFX** (age 9-60) randomized to 2012/13 **inactivated influenza vaccine (TIV) at the time of IFX**

	computer-generated list of small, variable blocks”					vaccination are more likely to consent to the trial (76%) than those without a history of influenza vaccination. This may reduce the immunologic response, but should not affect the serologic protection. This may also reduce the adverse event rates.	**infusion vs. midway between infusions**<br>• 50% of patients were on concomitant IM<br>• Serologic protection defined as postvaccine titer ≥ 1:40, immunologic response was defined by a 4-fold or greater increase between pre- and post -vaccination HAI titers.<br>• Seroprotection 67% vs. 55% for H1N1, 43% vs. 49% for H3N2, 69% vs. 79% to B<br>• Outcomes were assessed at baseline and 3-5 weeks after vaccination.<br>• **Serologic protection achieved in only 45-80% of IBD patients**.<br>• **No significant difference for serologic protection / immunologic response between the 2 groups (all below 40%)**<br>• Vaccine timing relative to IFX infusion does not affect the achievement of serologic protection<br>• Concomitant IM , longer duration of IFX use, decreased odds of achieving serologic protection to H1N1 only (AOR 0.45, 95% CI 0.2-0.9; AOR 0.5, 95% CI 0.3-0.9)<br>• **No serious adverse events**<br>• **6% had clinically significant increase in disease activity score, but not impacted by timing.**
Balint 2015 (Hungary) (IBD patients)	“The type of vaccine (whole virion or split virion vaccine) was randomly selected” Uneven number of	Unclear	Unclear	OK	OK	Patients who refused vaccination are likely to be prognostically different than those who agreed to vaccination.	• 156 IBD patients (age ≥ 18) randomized to **whole vs. split virion vaccine**. 53 patients refused the vaccine served as control.<br>• Majority of patients were treated with IM and biological therapy<br>• Outcomes were assessed at baseline and 5-6 weeks after vaccination<br>• Every patient had pre-existing protective levels of antibody to influenza viruses (previous vaccinations)

	randomized patients: whole virion (57), split virion (99)					Therefore, the comparison with controls may over-estimate the response to vaccines.	• **Post-immunization antibody titers of influenza A and B significantly increased in patients immunized with split virion compared with control**<br>• Post-immunization antibody tires significantly increased after the administration of split compared with whole virion<br>• **No serious adverse events**<br>• **No difference in influenza like symptoms between vaccinated vs. control (8.3% vs. 7.5%)**<br>• Relapse of disease occurred in 10%, more common in vaccinated than control
Matsumoto 2015 (Japan) (IBD patients)	Unclear	Unlikely as "open-label"	"Open-label"	OK	OK	OK	• 78 IBD patients on immunosuppressive therapy and 11 healthy individuals were randomized to **single vaccination vs. two vaccination booster with TIV** aged $\geq$ 20<br>• 29 patients received IM; 21 anti-TNF, 28 combination<br>• Seroprotection (HI titer $\geq$ 1:40), Seroresponse ($\geq$ 4-fold rise), GMT titer<br>• Outcomes were assessed at baseline, 3 weeks post-vaccination, and after the flu season in the single group; 3 weeks post-second vaccination and after the flu season in the booster group<br>• **No significant differences between single vs. booster in immunogenicity** (differences in GMTs, lower seroprotection rate for H1N1 strain after booster than single vaccination, similar seroprotection rates for other strains) .<br>• Second booster did not result in an additional immune response in patients who had an insufficient immune response<br>• **Seroprotection rate > 70% for every strain in the single group** (H1N1:85%, H3N2: 82%, B:100%)

							• No association between the types of immunosuppressive therapy or the immune responses<br>• Higher pre-vaccination titers were associated with sufficient immune response<br>• No differences between single vs. booster in immunogenicity among controls<br>• **No serious adverse events**

CD: Crohn's
EMA: European Medicines Agency
FDA: United States Food and Drug Administration
GMT: geometric mean titer
IFX: infliximab
IM: immunomodulators
QIV: quadrivalent inactivated influenza vaccine
TIV: trivalent inactivated influenza vaccine
UC: ulcerative colitis

**Cohort studies**							
**Study**	**Valid methods to ascertain exposure**	**Prognostic factors (other than exposure of interest) similar among cohorts – or cohorts were adjusted adequately for confounders**	**Demonstration that outcome of interest was not present at the start of the study**	**Outcome detection methods valid and similar among cohorts**	**Follow-up complete and similar among cohorts**	**Free of other bias**	**Comments**
Launay 2015 (France) (IBD patients)	OK	Did not adjust for disease severity or pre-vaccination titer which may be a confounding factor	OK	OK	31% of patients had missing data at the end of 2 years	Possible selection bias as uncertain how patients were recruited in the study. Patients who agreed to vaccination were likely to be prognostically	• 225 IBD adults aged 18-64 received the TIV in 2009-2010 and 2010-2011<br>• Group A: 31 no IS, Group B 77 IS, Group C 117 anti-TNF +/- IS<br>• Seroconversion defined as post-vaccination titer ≥ 1:40 and at least a 4-fold increase between post-vaccination and pre-

						different than patients who did not agree.	vaccination titers.<br>• Seroprotection defined as post-vaccination HI titer > 1:40<br>• Outcomes were assessed at baseline, 3 weeks and 6 mos after vaccination.<br>• **High seroprotection rates in IBD patients > 70%**<br>• No difference in seroconversion rates among groups (> 40%)<br>• **CHMP criteria were satisfied**<br>• **Anti-TNF +/- IS reduced the immune responses and the persistence of seroprotection rates at 6 mos after vaccination**<br>• Most patients did not develop influenza-like episodes (93%,80%, 83%)<br>• **No major adverse events**, disease exacerbation related to vaccine in 2/92 with CD on anti-TNF
Hagihara 2014 (Japan) (IBD patients)	OK	Adjusted for age, disease activity, and pre-vaccination titer.	OK	OK	OK	Possible selection bias as uncertain how patients were recruited in the study. Patients who agreed to vaccination were likely to be prognostically different patients who did not agree	• Prospective cohort study of 91 IBD patients aged > 20 who received a **single dose of 2010 TIV**<br>• Seroresponse defined as > 4-fold rise and seroprotection post-vaccination tier > 1:40<br>• Outcomes were assessed at baseline, 3 weeks post vaccine, and after flu season.<br>• Seroprotection was 81% H1N1, 61% for H3N2, and 86% for B.<br>• Seroresponse 73% for H1N1, 67% for H3N2, 53% for B<br>• GMT increased by a mean fold rise of 7.7 (H1N1), 6.4 (H3N2), and 4.6 (B)

							• **CHMP criteria were satisfied**<br>• **Immunosuppressants or anti-TNF alone or combination therapy independently reduced the immune response to the influenza vaccine for at least 1/3 strains.**<br>• **No serious adverse events including disease exacerbation**
Andrisani 2013 (Italy) (IBD patients)	OK	"No correlation was found between seroconversion rate, GMT and factor increase with age, gender, duration of disease and treatment, type of anti-TNF, disease activity, Montreal classification, and flu vaccination in the last 3 years"<br><br>"GMT was correlated with previous or concomitant vaccination for 2009 seasonal influenza and with GMT at baseline"	OK	OK	OK	OK	• Prospective cohort study with 62 consecutive IBD patients on **maintenance treatment with anti-TNF** for at least 4 mos and 31 healthy controls.<br>• Anti-TNF monotherapy (47), anti-TNF + IS (AZA, MTX) or steroids (15)<br>• All patients were vaccinated with **H1N1 vaccine**<br>• Seroprotection (HI titer $\geq$ 1:40), seroconversion ($\geq$ 4 fold increase in titer), GMT titer (> 2.5 factor increase) – all 3 criteria for pandemics vaccine<br>• Outcomes were assessed at baseline and 4 weeks post vaccine<br>• **Seroprotection rates comparable between IBD patients on anti-TNF vs. healthy controls (> 70%)**<br>• **Seroconversion rates lower among IBD patients on anti-TNF vs. healthy controls**<br>• **Patients on combined therapy (Anti-TNF + IS): the seroconversion rate and GMT factor increase did not reach the CHMA criteria.**

							• **Patients on anti-TNF alone met all 3 CHMA criteria.**<br>• **No disease fare or significant adverse events**
Cullen 2012<br>(US)<br>(IBD patients)	OK	"Age, gender, type of IBD, disease activity, and time from vaccination to assessment of serological response were not associated with lower immunogenicity"	OK	OK	OK	Possible selection bias as Patients who agreed to vaccination were likely to be prognostically different than patients who did not agree	• Prospective cohort study of 105 adult (age $\geq$ 18) patients with IBD vaccinated for the **2009 H1N1 influenza virus**<br>• 77/105 (73%) were on IS, 28 were not on IS<br>• Seroprotection defined as HI titer $\geq$ 40)<br>• Outcomes were assessed at baseline, 4-10 weeks post vaccine and after 6 mos<br>• Overall seroprotection was 50%, 64% in the NIS group, and 44% in the IS group (P = NS)<br>• **Lower proportion of seroprotection in those taking combined IS** (2 or more of steroid, thiopurine, MTX, or a biological drug) **than in those on monotherapy IS (36% vs. 42-47%, p = NS) or no IS (36% vs. 64%, p = 0.02)**<br>• Overall 11.4 fold increase of GMT post vaccination. Fold increase in GMT was higher in the NIS group than in the IS group (20.4 vs. 9.3, p = NS). **Fold increase was significantly lower in those taking combined IS than in those on monotherapy IS (3.5 vs. 11.5, p = 0.03)**<br>• **CHPA criteria not met for seroprotection (need 3 criteria**

							**met for pandemic vaccine)**<br>• **No serious adverse events**<br>• 6% (6/105) had influenza-lie illness during 6-month after vaccination<br>• 11% had increase in disease activity
Rahier 2011 (European multi-center study) (IBD patients)	OK	NA	OK	OK Assessment of disease activity by phone 4 weeks after the vaccine (may underestimate or overestimate the clinical scores)	6% missing data	Possible selection bias as Patients who agreed to vaccination were likely to be prognostically different than patients who did not agree	• Multi-center prospective study to evaluate local and systemic symptoms associated with influenza H1N1 vaccination in 575 patients with IBD receiving IM and/or biological therapy<br>• 41.7% monotherapy, 58.3% combined therapy<br>• 499 received adjuvanted, 76 non-adjuvanted vaccine<br>• 57% received seasonal influenza vaccine as well<br>• **Safety outcomes: No severe adverse side effects**<br>• Local and systemic symptoms in 34.6% and 15.5%. No difference in rates of local / systemic reactions in patients receiving monotherapy or combined therapy. Reactions are similar to those expected in the general population and are not influenced by either the disease itself or any treatment.<br>• **Absence of flare 4 weeks after vaccination in 96% of patients**
Gelinck 2008 (Netherlands) (autoimmune diseases including IBD)	OK	Did not adjust for disease severity or pre-vaccination titer which may be confounding factors	OK	OK	OK	Possible selection bias as Patients who agreed to vaccination	• Prospective cohort study of 112 patients with autoimmune diseases treated with anti-TNF (64), no anti-TNF (48), and healthy controls (18). Majority patients

						were likely to be prognostically different than patients who did not agree	had rheumatic diseases. IBD patients (18%).<br>• All received TIV to H3N2, H1N1, and B<br>• Seroprotection defined as HI titer $\geq$ 40). Seroresponse ($\geq$ 4 fold increase in titer),<br>• Outcomes were assessed at baseline and 4 weeks post vaccine<br>• **Seroprotection was high (80-94%) to H3N2, H1N1, and B in all 3 groups. CHMA criteria achieved.**<br>• Post vaccination GMT against H1N1, H3N2, and B were significantly lower in patients on anti-TNF compared with patients not on anti-TNF and healthy controls.

AZA: Azathioprine
GMT: Geometric mean titer
HI: haemagglutinin inhibition
IM: immunomodulator
IS: Immunosuppressants
MTX: methotrexate
NIS: non-immunosuppressed
TIV: trivalent inactivated influenza vaccine

Evidence Profile Table – Adults

Inactivated Influenza Vaccines in the Adult IBD Population

Certainty Assessment								Summary of Findings	
Studies	Risk of bias	Inconsistency	Indirectness	Imprecision	Other considerations	Certainty of Evidence	Overall Certainty of evidence	Study Event Rates	Relative Effect (95% CI)
**VPI (Influenza, assessed by laboratory confirmation) - CRITICAL**							⊕⊕⊕⊖ MODERATE For aged 16-65<br><br>⊕⊕⊕⊖ MODERATE For aged > 65		
1 SR 25 RCTs[1]<br><br>**Healthy adults aged 16-65**	Not serious	Not serious	Serious[a]	Not serious	None	⊕⊕⊕⊖ MODERATE		0.9% vs. 2.3% (vaccinated vs. controls)	RR 0.41 (0.36-0.47) NNT = 71
1 SR 3 RCTs[2]<br><br>**Adults aged > 65**	Serious[b]	Not serious	Serious[a]	Not serious	None	⊕⊕⊖⊖ LOW		2.4% vs. 6% (vaccinated vs. controls)	RR 0.42 (0.27-0.66) NNT = 30
**VPI (influenza-like illness assessed with: subjective report) - CRITICAL**									
1 SR 16 RCTs[1]<br><br>**Healthy adults aged 16-65**	Not serious	Serious[c]	Not serious	Not serious	None	⊕⊕⊕⊖ MODERATE		18.1% vs. 21.5% (vaccinated vs. controls)	RR 0.84 (0.75-0.95) NNT = 29
1 SR 4 RCTs[2]<br><br>**Adults aged > 65**	Serious[b]	Not serious	Not serious	Not serious	None	⊕⊕⊕⊖ MODERATE		3.5% vs. 5% (vaccinated vs. controls)	RR 0.59 (0.47-0.73) NNT = 42
**Immunogenicity (Seroprotection with HI titer ≥ 40) - IMPORTANT**									
8 Observational studies[3–10] (cohort studies and observational data from RCTs)<br><br>**IBD Populations**	Serious[d]	Serious[e]	Serious[f]	Serious[g]	None	⊕⊖⊖⊖ VERY LOW		• See **Summary of cohort and RCTs (observational data) assessing the seroprotection rates of influenza vaccine in IBD patients**<br>• Seroprotection rates range from 43-100%	
**Immunogenicity (Seroconversion with ≥ 4-fold increase in titer between pre- and post-vaccination titers) - IMPORTANT**									
5 Observational studies[3,4,6-8] (cohort studies and observational data from RCTs)	Serious[d]	Serious[e]	Serious[f]	Serious[g]	None	⊕⊖⊖⊖ VERY LOW		• See **Summary of cohort and RCTs (observational data) assessing the seroconversion rates of influenza vaccine in IBD patients**<br>• Seroconversion rates range from 23-76%	

**IBD Populations**								
**Immunogenicity (GMT fold rise > 2.5) - IMPORTANT**								
6<br>Observational studies[3,5-9]<br>(cohort studies and observational data from RCTs)<br><br>**IBD Populations**	Serious[d]	Not serious	Serious[f]	Serious[g]	None	⊕⊖⊖⊖<br>VERY LOW		• See **Summary of cohort studies and RCTs (observational data) assessing the fold increases in GMT of influenza vaccine in IBD patients**<br>• GMT fold rise range from 1.77-20.4
**Adverse events - CRITICAL**								
1 SR 13 RCTs and observational studies[1]<br><br>**Healthy adults aged 16-65**	Not serious	Not serious	Serious[h]	Not serious	None	⊕⊕⊕⊖<br>MODERATE		• No evidence of an association between exposure to inactivated influenza vaccine and serious adverse events (e.g. Guillain-Barre' syndrome, multiple sclerosis, optic neuritis, and immune thrombocytopenia)<br>• Increased fever with inactivated vaccine (RR 1.55, 1.26-1.91)
1 SR 3 RCTs[2]<br><br>**Adults aged > 65**	Not serious	Not serious	Serious[h]	Not serious	None	⊕⊕⊕⊖<br>MODERATE		• No serious adverse events
10<br>Observational studies[3-12]<br>(cohort studies and observational data from RCTs)<br><br>**IBD Populations**	Serious[d]	Not serious	Not serious	Serious[g]	None	⊕⊖⊖⊖<br>VERY LOW		• No serious adverse events or vaccine induced disease exacerbation

GMT: geometric mean titer
HI: Haemagglutination inhibition

Footnotes:

a. Downgraded for indirectness. Uncertainty over definition, surveillance and testing of influenza in older trials. Immunogenicity studies in IBD patients suggested that the influenza vaccine may not be as immunogenic (and therefore as effective) as in the general population.
b. Downgraded for study limitations. Most of the evidence summarized in the meta-analysis comes from studies with high or unclear risk of bias for more than one 'Risk of bias' domain.
c. Downgraded for inconsistent results. There is discordance between the direction and size of effects across studies. Different definitions of influenza-like illness across the studies could explain why there is variation in the event rates across the control arms.
d. Downgraded for study limitations. IBD patients who agreed to vaccination were likely to be systematically different than those who did not agree or seek vaccination (healthy vaccinee effect or confounding by indication). This may lead to selection bias confounding the vaccine's effect on the outcomes (e.g. mortality, infection, and even immunologic response).

e. Downgraded for inconsistency. Immunologic response to different strains (H1N1, H3N2, B) were variable among studies. The response to influenza vaccine varies and comparisons between different studies are difficult to make because of annual reformulation of vaccine resulting in differing antigenicity of each year's vaccine. Additionally, in some years, strains can remain unchanged from previous years. Thus, a study participant who had been vaccinated against the same strain the previous year might have a different response than a participant naïve to that strain. As well, patient populations were heterogeneous across studies with different disease severity / activity, previous vaccination history (pre-vaccination titer), use of immunosuppressive medications, and risk factors for influenza infection.
f. Downgraded for indirectness. Surrogate outcomes were used to estimate clinical effectiveness of influenza vaccine. CHMP criteria for serological response to vaccination are based on healthy volunteers aged 18 to 60 years with attenuated strains, thus may not reflect expected rates of clinical protection observed in other populations (e.g. vaccinated immunocompromised populations).
g. Small sample size with varying subgroups of patients on no medications or different combinations of medications.
h. Downgraded for indirectness. Sample sizes in the IBD studies were insufficient to detect rare adverse events.

Summary of cohort studies and RCTs (observational data) assessing the seroprotection rates (HI titer ≥ 1:40) of influenza vaccine in IBD patients

Study	Age group	Number of patients	Types of vaccine	Weeks post vaccination	H1N1	H3N2	B
Shirai 2018	Mean age 42.6	83	QIV single	4	66%	77%	80%, 84%
	Mean age 42.3	49	QIV double	4	69%	69%	84%, 80%
Debruyn 2016	9-60	69	TIV 2012/2013 (at infusion of IFX)	3-5	67.2%	43.3%	68.7%
	9-60	68	TIV 2012/2013 (midway between infusions of IFX)	3-5	64.7%	48.5%	79.4%
Matsumoto 2015	Mean age 45.3	46	TIV 2012-2013 single	3	85%	82%	100%
	Mean age 42.4	43	TIV 2012-2013	3	64%	77%	100%

			double				
Launay 2015	18-64	255	TIV 2009-2010 and 2010-2011	3	No IS: 77%<br>IM: 75%<br>Anti-TNF +/- IM: 66%<br>P = 0.35	No IS: 77%<br>IM: 68%<br>Anti-TNF +/- IM: 52%<br>P = 0.014	No IS: 97%<br>IM: 96%<br>Anti-TNF +/- IM: 95%<br>P = 0.99
Hagihara 2014	$\geq$ 20	91	TIV 2010/2011	3	81%	61%	86%
Andrisani 2013	18-75	62	2009 pandemic H1N1	4	Anti-TNF: 91%<br>Anti-TNF + IM: 80%	-	-
Cullen 2012	20-68	108	2009 pandemic H1N1	4-10	Overall: 50%<br>No IS: 64%<br>IS: 44%<br>P = 0.06	-	-
Gelinck 2008	18 – 85	112 autoimmune diseases 18% IBD	TIV 2003/2004	4	89-94%	89-94%	89-94%

IFX: infliximab
IM: immunomodulators
IS: immunosuppressants including steroids, immunomodulators, or biologics
QIV: quadrivalent inactivated influenza vaccine
TIV: trivalent influenza vaccine
European Union Committee for Medicinal Products for Human Use (CHMP) criteria (Hi titer $\geq$ 40) > 70% for the evaluation of seasonal influenza vaccine immunogenicity met (yellow shading)

Summary of cohort studies and RCTs (observational data) assessing the seroconversion rates ($\geq$ 4-fold increase in titer) of influenza vaccine in IBD patients

Study	Age group	Number of patients	Types of vaccine	Weeks post vaccination	H1N1	H3N2	B
Shirai 2018	Mean age 42.6	83	QIV single	4	36%	58%	52%, 51%
	Mean age	49	QIV double	4	43%	59%	41%, 43%

	42.3						
Debruyn 2016	9-60	69	TIV 2012/2013 (at infusion of IFX)	3-5	27.7%	27.7%	34.9%
	9-60	68	TIV 2012/2013 (midway between infusions of IFX)	3-5	36.7%	23.3%	40.0%
Launay 2015	18-64	255	TIV 2009-2010 and 2010-2011	3	No IS: 67%<br>IM: 64%<br>Anti-TNF +/- IM: 54%<br>P = 0.28	No IS: 63%<br>IM: 50%<br>Anti-TNF +/- IM: 41%<br>P = 0.074	No IS: 63%<br>IM: 76%<br>Anti-TNF +/- IM: 60%<br>P = 0.078
Hagihara 2014	$\geq$20	91	TIV 2010/2011	3	73%	67%	53%
Andrisani 2013	18-75	62	2009 pandemic H1N1	4	Anti-TNF: 49%<br>Anti-TNF + IM: 33%	-	-

IFX: infliximab
IM: immunomodulators
IS: immunosuppressants including steroids, immunomodulators, or biologics
QIV: quadrivalent inactivated influenza vaccine
TIV: trivalent influenza vaccine
European Union Committee for Medicinal Products for Human Use (CHMP) criteria *(at least a 4-fold increase in titer) > 40%* for the evaluation of seasonal influenza vaccine immunogenicity met (yellow shading)

Summary of cohort studies and RCTs (observational data) assessing the fold increases in GMT of influenza vaccine in IBD patients

Study	Age group	Number of patients	Types of vaccine	Weeks post vaccination	H1N1	H3N2	B
Shirai 2018	Mean age 42.6	83	QIV single	4	3.50	5.15	3.82, 4.06
	Mean age 42.3	49	QIV double	4	2.69	5.46	3.38, 3.67

Matsumoto 2015	Mean age 45.3	46	TIV 2012-2013 single	3	6.35	4.95	2.48
	Mean age 42.4	43	TIV 2012-2013 double	3	4.22	3.86	1.77
Launay 2015	18-64	255	TIV 2009-2010 and 2010-2011	3	No IS: 7.9<br>IM: 7.7<br>Anti-TNF +/- IM: 6.8<br>P = 0.82	No IS: 6.5<br>IM: 4.1<br>Anti-TNF +/- IM: 3.2<br>P = 0.0348	No IS: 7.0<br>IM: 6.5<br>Anti-TNF +/- IM: 5.0<br>P = 0.21
Hagihara 2014	$\geq$20	91	TIV 2010/2011	3	7.7	6.4	4.6
Andrisani 2013	18-75	62	2009 pandemic H1N1	4	Anti-TNF: 3.5<br>Anti-TNF + IM: 1.74	-	-
Cullen 2012	20-68	108	2009 pandemic H1N1	4-10	Overall: 11.4<br>No IS: 20.4<br>IS: 9.3<br>P = 0.06		

GMT: geometric mean titer
IM: immunomodulators
IS: immunosuppressants including steroids, immunomodulators, or biologics
QIV: quadrivalent inactivated influenza vaccine
TIV: trivalent influenza vaccine
European Union Committee for Medicinal Products for Human Use (CHMP) criteria (*geometric mean fold rise > 2.5)* for the evaluation of seasonal influenza vaccine immunogenicity met (yellow shading)

Single vs Double (Booster) Influenza Vaccination in Adult IBD Population

Certainty Assessment	Summary of Findings		Comments
	No of patients (ITT)	Effect	

Studies	Risk of bias	Inconsistency	Indirectness	Imprecision	Other considerations	Certainty of Evidence	Overall certainty of evidence	Booster	Single	Relative (95%CI)	Absolute (95%CI)	
**Immunogenicity (Seroprotection with HI titer $\geq$ 40) – IMPORTANT**												
2 RCTs[3,5]	Serious[a]	Not serious	Serious[b]	Serious[c]	Publication bias cannot be assessed (< 10 studies)	⊕⊖⊖⊖ VERY LOW		• See **Summary of cohort and RCTs (observational data) assessing the seroprotection rates of influenza vaccine in IBD patients**<br>• No significant difference in seroprotection rates between single vs. booster vaccination groups				Results cannot be combined in a meta-analysis as different vaccine preparations were assessed (QIV, TIV)
**Immunogenicity (Seroconversion with $\geq$ 4-fold increase in titer between pre- and post-vaccination titers) – IMPORTANT**												
1 RCTs[3]	Serious[d]	Not serious	Serious[b]	Serious[e]	Publication bias cannot be assessed (< 10 studies)	⊕⊖⊖⊖ VERY LOW	⊕⊖⊖⊖ VERY LOW	• See **Summary of cohort and RCTs (observational data) assessing the seroconversion rates of influenza vaccine in IBD patients**<br>• No significant difference in seroconversion rates between single vs. booster vaccination groups.				Results cannot be combined in a meta-analysis as different vaccine preparations were assessed (QIV, TIV)
**Immunogenicity (GMT fold rise > 2.5) – IMPORTANT**												
2 RCTs[3,5]	Serious[a]	Not serious	Serious[b]	Serious[c]	Publication bias cannot be assessed (< 10 studies)	⊕⊖⊖⊖ VERY LOW		• See **Summary of cohort studies and RCTs (observational data) assessing the fold increases in GMT of influenza vaccine in IBD patients**<br>• No significant difference in GMT rise between single vs. booster vaccination groups				Results cannot be combined in a meta-analysis as different vaccine preparations were assessed (QIV, TIV)
**Adverse events – CRITICAL**												

2<br>RCTs[3,5]	Serious[a]	Not serious	Serious[f]	Serious[c]	Publication bias cannot be assessed (< 10 studies)	⊕⊝⊝⊝<br>VERY LOW		• No serious adverse effects including exacerbation of disease				Results cannot be combined in a meta-analysis as different vaccine preparations were assessed (QIV, TIV)

Footnotes:

a. Downgraded for study limitations. Both studies were considered high or unclear risk of bias for random sequence generation, allocation concealment and blinding.
b. Downgraded for indirectness. Both studies were conducted in Japan with different formulations of vaccines (2015/2016 seasonal QIV and 2012-2013 seasonal TIV with different vaccine strains). Results may not be generalizable to other IBD patient populations. Surrogate outcomes (immunogenicity) were assessed.
c. Downgraded for imprecision with small sample sizes (n = 210).
d. Downgraded for study limitations. Study was considered high risk of bias for random sequence generation, allocation concealment and blinding.
e. Downgraded for imprecision with small sample size (n = 132).
f. Downgraded for indirectness. Both studies were conducted in Japan with different formulations of vaccines (2015/2016 seasonal QIV and 2012-2013 seasonal TIV with different vaccine strains). Results may not be generalizable to other IBD patient populations.

Timing of Influenza Vaccination in Adult IBD patients on Maintenance Infliximab Therapy

Certainty Assessment								Summary of Findings				Comments
								No of patients (ITT)		Effect		
Studies	Risk of bias	Inconsistency	Indirectness	Imprecision	Other considerations	Certainty of Evidence	Overall certainty of evidence	Vaccine Mid-way Between Infusions	Vaccine at Time of Infusion	Relative (95%CI)	Absolute (95%CI)	
**Immunogenicity (Seroprotection with HI titer ≥ 40) - IMPORTANT**							⊕⊕⊝⊝<br>LOW					
1<br>RCT[4]	Serious[a]	Not serious	Serious[b]	Serious[c]	Publication bias cannot be assessed (< 10 studies)	⊕⊝⊝⊝<br>VERY LOW		• See **Summary of cohort and RCTs (observational data) assessing the seroprotection rates of influenza vaccine in IBD patients**<br>• No significant difference in seroprotection rates				

								between vaccination at time of infusion vs. midway between infusions.	
**Immunogenicity (Seroconversion with ≥ 4-fold increase in titer between pre- and post-vaccination titers) - IMPORTANT**									
1<br>RCT[4]	Serious[a]	Not serious	Serious[b]	Serious[c]	Publication bias cannot be assessed (< 10 studies)	⊕⊖⊖⊖<br>VERY LOW		• See **Summary of cohort and RCTs (observational data) assessing the seroconversion rates of influenza vaccine in IBD patients**<br>• No significant difference in seroconversion rates between vaccination at time of infusion vs. midway between infusions.	
**Adverse events - CRITICAL**									
1<br>RCT[4]	Serious[a]	Not serious	Not serious	Serious[c]	Publication bias cannot be assessed (< 10 studies)	⊕⊕⊖⊖<br>LOW		• No serious adverse effects<br>• 6% of subjects had a clinically significant increase in disease activity score, not impacted by vaccine timing.	

Footnotes:

a. Downgraded for study limitations. High risk of bias for allocation concealment and blinding.
b. Downgraded for indirectness. Surrogate outcomes were used to estimate effectiveness of vaccine. 16% of participants were pediatric IBD patients.
c. Downgraded for imprecision. Small sample size (n = 137).

References:

1. Demicheli V, Jefferson T, Ferroni E, Rivetti A, Di Pietrantonj C. Vaccines for preventing influenza in healthy adults.Cochrane Database Syst Rev. 2018 Feb 1;2:CD001269.
2. Demicheli V, Jefferson T, Di Pietrantonj C, Ferroni E, Thorning S, Thomas RE, Rivetti A. Vaccines for preventing influenza in the elderly. Cochrane Database Syst Rev. 2018 Feb 1;2:CD004876.
3. Shirai S, Hara M, Sakata Y, Tsuruoka N, Yamamoto K, Shimoda R, Gomi Y, Yoshii H, Fujimoto K, Iwakiri R. Immunogenicity of Quadrivalent Influenza Vaccine for Patients with Inflammatory Bowel Disease Undergoing Immunosuppressive Therapy. Inflamm Bowel Dis. 2018 Apr 23;24(5):1082-1091.
4. deBruyn J, Fonseca K, Ghosh S, Panaccione R, Gasia MF, Ueno A, Kaplan GG, Seow CH, Wrobel I. Immunogenicity of Influenza Vaccine for Patients with Inflammatory Bowel Disease on Maintenance Infliximab Therapy: A Randomized Trial. Inflamm Bowel Dis. 2016 Mar;22(3):638-47.
5. Matsumoto H, Ohfuji S, Watanabe K, Yamagami H, Fukushima W, Maeda K, Kamata N, Sogawa M, Shiba M, Tanigawa T, Tominaga K, Watanabe T, Fujiwara Y, Hirota Y, Arakawa T. Booster influenza vaccination does not improve immune response in adult inflammatory bowel disease patients treated with immunosuppressives: a randomized controlled trial. J Gastroenterol. 2015 Aug;50(8):876-86.
6. Launay O, Abitbol V, Krivine A, Slama LB, Bourreille A, Dupas JL, Hébuterne X, Savoye G, Deplanque D, Bouhnik Y, Pelletier AL, Galtier F, Laharie D, Nachury M, Zerbib F, Allez M, Bommelaer G, Duclos B, Lucht F, Gougeon ML, Tartour E, Rozenberg F, Hanslik T, Beaugerie L, Carrat F; MICIVAX Study Group. Immunogenicity and Safety of Influenza Vaccine in Inflammatory Bowel Disease Patients Treated or not with Immunomodulators and/or Biologics: A Two-year Prospective Study. J Crohns Colitis. 2015 Dec;9(12):1096-107.

7. Hagihara Y, Ohfuji S, Watanabe K, Yamagami H, Fukushima W, Maeda K, Kamata N, Sogawa M, Shiba M, Tanigawa T, Tominaga K, Watanabe T, Fujiwara Y, Hirota Y, Arakawa T. Infliximab and/or immunomodulators inhibit immune responses to trivalent influenza vaccination in adults with inflammatory bowel disease. J Crohns Colitis. 2014 Mar;8(3):223-33.
8. Andrisani G, Frasca D, Romero M, Armuzzi A, Felice C, Marzo M, Pugliese D, Papa A, Mocci G, De Vitis I, Rapaccini GL, Blomberg BB, Guidi L. Immune response to influenza A/H1N1 vaccine in inflammatory bowel disease patients treated with anti TNF-α agents: effects of combined therapy with immunosuppressants. J Crohns Colitis. 2013 May;7(4):301-7.
9. Cullen G, Bader C, Korzenik JR, Sands BE. Serological response to the 2009 H1N1 influenza vaccination in patients with inflammatory bowel disease. Gut. 2012 Mar;61(3):385-91.
10. Gelinck LB, van der Bijl AE, Beyer WE, Visser LG, Huizinga TW, van Hogezand RA, Rimmelzwaan GF, Kroon FP. The effect of anti-tumour necrosis factor alpha treatment on the antibody response to influenza vaccination. Ann Rheum Dis. 2008 May;67(5):713-6.
11. Bálint A, Farkas K, Éva PK, Terhes G, Urbán E, Szucs M, Nyári T, Bata Z, Nagy F, Szepes Z, Miheller P, Lorinczy K, Lakatos PL, Lovász B, Tamás S, Kulcsár A, Berényi A, Törocsik D, Daróczi T, Saródi Z, Wittmann T, Molnár T. Antibody and cell-mediated immune response to whole virion and split virion influenza vaccine in patients with inflammatory bowel disease on maintenance immunosuppressive and biological therapy. Scand J Gastroenterol. 2015 Feb;50(2):174-81.
12. Rahier JF, Papay P, Salleron J, Sebastian S, Marzo M, Peyrin-Biroulet L, Garcia-Sanchez V, Fries W, van Asseldonk DP, Farkas K, de Boer NK, Sipponen T, Ellul P, Louis E, Peake ST, Kopylov U, Maul J, Makhoul B, Fiorino G, Yazdanpanah Y, Chaparro M; European Crohn's and Colitis Organisation (ECCO). H1N1 vaccines in a large observational cohort of patients with inflammatory bowel disease treated with immunomodulators and biological therapy. Gut. 2011 Apr;60(4):456-62.

Evidence to Decision Table – Adults

**PICO 14A**	**In adult patients with IBD (65 years of age and younger), should vaccination vs. no vaccination against influenza be given?**
**Population**	Adult patients with IBD **(65 years of age and younger)**
**Intervention**	Vaccination against influenza
**Comparator**	No vaccination against influenza
**Outcome**	Mortality, VPI (influenza infection), SAEs, Immunogenicity

**PICO 14B**	**In adult patients with IBD (older than 65 years of age), should vaccination vs. no vaccination against influenza be given?**
**Population**	Adult patients with IBD with **(older than 65 years of age)**
**Intervention**	Vaccination against influenza
**Comparator**	No vaccination against influenza

Outcome	Mortality, VPI (influenza infection), SAEs, Immunogenicity

	Judgement	Research evidence	Additional considerations
**Desirable Effects**	**How substantial are the desirable anticipated effects?**<br><br>○ Trivial<br>○ Small<br>○ Moderate<br>○ Large<br><br>○ Varies<br>○ Don't know	See Evidence Profile Tables.<br><br>**Risk of Influenza infection in adult IBD patients**<br><br>Two observational studies addressed this PICO question.[1,2]<br><br>One was a cross-sectional case-control study that used an US administrative database (Nationwide Inpatient Sample) to compare the risks of hospitalization for influenza pneumonia among adult IBD patients vs. non-IBD controls.[1] It is important to note that influenza pneumonia patients treated as outpatients were excluded. After adjusting for various factors including comorbidities, risk factors for pneumonia, as well as patient and hospital characteristics, IBD patients did not demonstrate an increased odd of hospitalization for pneumonia due to influenza virus. However, low income UC patients had an increased odd of hospitalization for pneumonia due to influenza virus (aOR 1.86, CI 1.46-2.37). Mortality during these admissions among IBD patients was not significantly higher than the control population.<br><br>The other case-control study used a US commercial administrative database containing inpatient, outpatient, and pharmacy data to assess the risks of influenza and its related complications among adult IBD patients vs. non-IBD controls.[2] This study provided more direct evidence as both outpatients and inpatients (population of interest) treated for influenza infection were captured in this study. After adjusting for health care utilization and comorbid illnesses, IBD patients had an increased risk for influenza infection compared with non-IBD controls (aHR 1.28, CI 1.19-1.37). IBD patients also had significantly more hospitalizations within 30 days of an influenza diagnosis compared with non-IBD controls (5.4% vs. 1.85%, $P < 0.001$). As this study provided more direct evidence than the previous study, the GRADE rating was anchored at this study.<br><br>The GRADE rating started at high as it was considered a prognostic study (providing evidence about the likelihood of influenza infection in patients with IBD). The rating was further downgraded to **low** due to study limitations (residual confounding factors, detection bias, admission bias, and misclassification bias). In particular, patients with IBD and respiratory symptoms may be more likely to be tested for, diagnosed with, and admitted for influenza than non-IBD controls, thus creating an overestimate of the risk of influenza among IBD patients. **In summary, there is low certainty evidence that adult IBD patients have an increased risk of influenza infection compared to non-IBD patients.**	
**Undesirable Effects**	**How substantial are the undesirable anticipated effects?**<br><br>○ Large<br>○ Moderate<br>○ Small<br>○ Trivial<br><br>○ Varies<br>○ Don't know		

Effectiveness and safety of Inactivated Influenza Vaccine in adult IBD patients

There was no RCT comparing influenza vaccine with placebo or no treatment in adult patients with IBD to address this PICO question.

There are 2 Cochrane systematic reviews assessing the safety and effectiveness of inactivated influenza vaccine in healthy adults aged 16-65 and in the elderly aged > 65.[3,4] In healthy adults, inactivated influenza vaccines reduce the risk of influenza (RR 0.41, 95% CI 0.36-0.47).[3] The certainty of evidence was moderate due to indirectness for outcome with uncertainty over definition, surveillance and testing of influenza in older trials.[3] The certainty of evidence was also moderate for the outcome of influenza-like illness due to inconsistency. In elderly aged > 65, inactivated influenza vaccines also reduce the risk of influenza (RR 0.42, 95% CI 0.27-0.66).[4] However, the certainty of evidence was low due to indirectness for outcome and study limitations with most of the evidence coming from studies with high or unclear risk of bias for more than one risk of bias domain, but moderate for influenza-like illness due to study limitations.[4]

There are six cohort studies and four RCTs (observational data) that addressed this PICO question using surrogate outcomes of immunogenicity (seroprotection, seroconversion, GMT fold rise in titer).[5-14] The assessed vaccines include trivalent and quadrivalent inactivated influenza vaccines, as well as the 2009 pandemic H1N1 vaccine. According to the criteria defined by the European Union Committee for Medicinal Products for Human Use (CHMP) for the evaluation of seasonal influenza vaccine immunogenicity in immunocompetent adults aged 18-60 years, at least one of the following serological criteria for Haemagglutination inhibition (HI) antibody response should be achieved: seroprotection (HI titer $\geq$ 40) > 70% (or > 60% in age > 60), seroconversion (at least a 4-fold increase in titer) > 40% (or > 30% in age > 60), or geometric mean titer (GMT) fold rise > 2.5 (or > 2 in age > 60). For pandemic vaccines, all three of the criteria had to be met. However, CHMP criteria for serological response to vaccination are based on healthy volunteers aged 18 to 60 years with attenuated strains, thus may not reflect expected rates of clinical protection observed in other populations (e.g. children, older adults, adults with underlying comorbidities, vaccinated immunocompromised populations). Furthermore, methods of standardization of antibody titres are lacking.

The evidence suggests that influenza vaccination can induce seroprotection (43-100% achieving HI titer $\geq$ 40), seroconversion (23-76% achieving at least a 4-fold increase in titer), and GMT fold rise (1.77-20.4) in a significant proportion of adult IBD patients. Immunosuppressive medications (e.g. immunomodulators, anti-TNF, steroids) may reduce the immunologic response to influenza vaccination in IBD patients, particularly when multiple immunosuppressive medications are used. However, it is uncertain if this reduced immunologic response is clinically relevant/important and would still afford clinical protection. The GRADE rating started at low due to the observational designs of the studies. The rating was downgraded to very low due to study limitations (selection bias, residual confounding), indirectness (surrogate outcome), inconsistency, and imprecision. In particular, IBD patients who agreed to vaccination were likely to be systematically different than those who did not agree or seek vaccination (healthy vaccinee effect or confounding by indication). This may lead to selection bias

		confounding the vaccine's effect on the outcomes (e.g. mortality, infection, adverse events, and even immunologic response). As well, the response to influenza vaccine was quite variable, and comparisons between different studies are difficult to make because of annual reformulation of vaccine resulting in differing antigenicity of each year's vaccine. Protective effect of a vaccine is also dependent on whether the circulating strain is included in the vaccine. Additionally, in some years, strains can remain unchanged from previous years. Thus, a study participant who had been vaccinated against the same strain the previous year might have a different response than a participant naïve to that strain (depending on pre-vaccination titers). Patient populations were also quite variable across studies with different proportions of patients being on different immunosuppressive medications. This may have contributed to the inconsistency in results across studies. It is important to note that most patients included in the studies were age 18-65. Very few elderly patients (age > 65) were included.<br><br>The evidence for effectiveness was anchored to the general population. As immunogenicity studies suggested that inactivated vaccines may be less immunogenic (and therefore less effective) in adult IBD patients, we would also need to need to consider downgrading for indirectness. However, the degree of indirectness was not judged to be severe enough to warrant further downgrading as most studies showed the CHMP criteria for immunogenicity was met in adult IBD populations. Therefore, **the overall certainty of evidence for effectiveness of inactivated influenza vaccine was judged to remain <u>moderate</u> in adult IBD patients aged 16-65 and <u>moderate</u> in elderly IBD patients over age 65.**<br><br>The two Cochrane systematic reviews in healthy adults and elderly adults also showed no serious adverse events associated with the use of inactivated influenza vaccine.[3,4] The certainty of evidence for safety was high for healthy adults and moderate for elderly adults.[3,4] No serious adverse events including disease exacerbation was reported in the 10 observational studies in adult IBD patients.[5-14] The certainty of evidence for safety was anchored to the general population, and downgraded for indirectness as sample sizes in the IBD studies were insufficient to detect rare adverse events. **Therefore, the overall certainty of evidence for safety of inactivated influenza vaccine was judged to be <u>moderate</u> in adult IBD patients aged 16-65 and <u>moderate</u> in elderly IBD patients over age 65.**<br><br>**Overall, there is <u>moderate</u> certainty evidence that inactivated influenza is safe and effective in adult IBD patients aged 16-65 and elderly IBD patients age > 65.**<br><br>**Single vs. booster influenza vaccination:**<br><br>Two RCTs addressed the question of effectiveness and safety between single vs. booster influenza vaccination in IBD patients.[5,7] Both studies suggested no significant difference in immunogenicity (seroprotection, seroconversion, GMT titer rise) between single vs. booster vaccination strategies in IBD patients. No serious adverse events including disease exacerbation was reported. The GRADE rating started at high. The rating was downgraded to **<u>very low</u>** due to study limitations, indirectness (surrogate	

		outcomes, patient populations), and imprecision. Both studies were conducted in Japan with different formulations of vaccines (2015/2016 seasonal QIV and 2012-2013 seasonal TIV with different vaccine strains). Therefore, results may not be generalizable to other IBD patient populations. As well, surrogate outcomes were used. **In summary, there is very low certainty evidence that there is no significant difference in effectiveness and safety between single vs. booster influenza vaccination in adult IBD patients.**<br><br>**Timing of influenza vaccination relative to anti-TNF therapy:**<br><br>One RCT addressed the question of effectiveness and safety related to timing of influenza vaccination in IBD patients on maintenance infliximab therapy.[6] The study suggested no significant difference in immunogenicity (seroprotection and seroconversion) between vaccination given at the time of infliximab infusion vs. midway between infusions. No serious adverse events were reported. The GRADE rating started at high. The rating was downgraded to **low** due to study limitations and imprecision. **In summary, there is low certainty evidence that timing of influenza vaccination relative to infliximab infusion does not affect the effectiveness and safety of influenza in adult IBD patients.**	
Certainty of evidence	**What is the overall certainty of the evidence of effects?**<br><br>o Very low<br>o Low<br>o Moderate for IBD patients aged 16-65 and age > 65<br>o High<br><br>o No included studies		
Values and Preferences	**Is there important uncertainty about or variability in how much people value the main outcomes?**<br><br>o Important uncertainty or variability<br>o Possibly important uncertainty or variability<br>o Probably no important uncertainty or variability<br>o No important uncertainty or variability	Patients likely value patient-important outcomes (mortality, VPI, adverse effects) more than surrogate outcomes (immunogenicity).	

**Balance of effects**	**Does the balance between desirable and undesirable effects favor the intervention or the comparison?**<br><br>o Favors the comparison<br>o Probably favors the comparison<br>o Does not favor either the intervention or the comparison<br>o Probably favors the intervention<br>o Favors the intervention<br><br>o Varies<br>o Don't know		
**Resources required**	**How large are the resource requirements (costs)?**<br><br>o Large costs<br>o Moderate costs<br>o Negligible costs and savings<br>o Moderate savings<br>o Large savings<br><br>o Varies<br>o Don't know	CDC vaccine price list last reviewed/updated: July 1, 2019	
**Certainty of Evidence of Required Resources**	**What is the certainty of the evidence of resource requirements (costs)?**<br><br>o Very low<br>o Low<br>o Moderate<br>o High<br><br>o No included studies	The costs of delivering routine immunization services may vary widely across countries and different health system settings. See Immunization Costing Action Network (ICAN) Immunization Delivery Cost Catalogue. http://immunizationeconomics.org/ican-idcc.	

Brandname	CDC cost/dose	Private sector cost/dose
Fluzone® Quadrivalent	$12.808	$16.939
Fluarix® Quadrivalent	$12.22	$16.82
FluLaval® Quadrivalent	$11.94	$15.77
Flucelvax® Quadrivalent	$15.00	$22.758
Afluria® Quadrivalent	$11.35	$15.871

**Cost effectiveness**	**Does the cost-effectiveness of the intervention favor the intervention or the comparison?**<br><br>○ Favors the comparison<br>○ Probably favors the comparison<br>○ Does not favor either the intervention or the comparison<br>○ Probably favors the intervention<br>○ Favors the intervention<br><br>○ Varies<br>○ No included studies	There are no studies that addressed this question specifically in the IBD population.<br><br>In a systematic review of cost-effectiveness of adult influenza vaccination, the cost-effectiveness of influenza vaccination ranged from $8000 to $39,000 per QALY.[15] Assessments for adults aged $\geq$ 65 yielded lower CE ratios, ranging from being cost-saving to $15,300 per QALY. Influenza vaccination in adults appears to have a similar CE profile as other commonly utilized preventative services for adults (e.g. colorectal cancer screening, breast cancer screening etc.).[15]<br><br>In a systematic review of cost-effectiveness of adult vaccinations, influenza vaccine was found to have favorable cost-effectiveness profiles.[16] For outcomes assessing age-based vaccinations, the percent indicating any cost-effectiveness estimates equal to or below $50,000/QALY were 100 for influenza.[16]<br><br>In another systematic review of the cost-effectiveness of influenza immunization programs with inclusion of 41 studies, vaccinating high-risk adults (cancer patients, elderly adults, underlying chronic diseases – asthma, cardiovascular disease, diabetes, HIV, hypertension, stroke) was found to be highly cost-effective.[17]	
**Acceptability**	**Is the intervention acceptable to key stakeholders?**<br><br>○ No<br>○ Probably no<br>○ Probably yes<br>○ Yes<br><br>○ Varies<br>○ Don't know		
**Feasibility**	**Is the intervention feasible to implement?**<br><br>○ No<br>○ Probably no<br>○ Probably yes<br>○ Yes<br><br>○ Varies<br>○ Don't know		

References:

1. Stobaugh DJ, Deepak P, Ehrenpreis ED. Hospitalizations for vaccine preventable pneumonias in patients with inflammatory bowel disease: a 6-year analysis of the Nationwide Inpatient Sample. Clin Exp Gastroenterol. 2013 May 6;6:43-9.
2. Tinsley A, Navabi S, Williams ED, Liu G, Kong L, Coates MD, Clarke K. Increased Risk of Influenza and Influenza-Related Complications Among 140,480 Patients With Inflammatory Bowel Disease. Inflamm Bowel Dis. 2019 Jan 10;25(2):369-376.
3. Demicheli V, Jefferson T, Ferroni E, Rivetti A, Di Pietrantonj C. Vaccines for preventing influenza in healthy adults.Cochrane Database Syst Rev. 2018 Feb 1;2:CD001269.
4. Demicheli V, Jefferson T, Di Pietrantonj C, Ferroni E, Thorning S, Thomas RE, Rivetti A. Vaccines for preventing influenza in the elderly. Cochrane Database Syst Rev. 2018 Feb 1;2:CD004876.
5. Shirai S, Hara M, Sakata Y, Tsuruoka N, Yamamoto K, Shimoda R, Gomi Y, Yoshii H, Fujimoto K, Iwakiri R. Immunogenicity of Quadrivalent Influenza Vaccine for Patients with Inflammatory Bowel Disease Undergoing Immunosuppressive Therapy. Inflamm Bowel Dis. 2018 Apr 23;24(5):1082-1091.
6. deBruyn J, Fonseca K, Ghosh S, Panaccione R, Gasia MF, Ueno A, Kaplan GG, Seow CH, Wrobel I. Immunogenicity of Influenza Vaccine for Patients with Inflammatory Bowel Disease on Maintenance Infliximab Therapy: A Randomized Trial. Inflamm Bowel Dis. 2016 Mar;22(3):638-47.
7. Matsumoto H, Ohfuji S, Watanabe K, Yamagami H, Fukushima W, Maeda K, Kamata N, Sogawa M, Shiba M, Tanigawa T, Tominaga K, Watanabe T, Fujiwara Y, Hirota Y, Arakawa T. Booster influenza vaccination does not improve immune response in adult inflammatory bowel disease patients treated with immunosuppressives: a randomized controlled trial. J Gastroenterol. 2015 Aug;50(8):876-86.
8. Launay O, Abitbol V, Krivine A, Slama LB, Bourreille A, Dupas JL, Hébuterne X, Savoye G, Deplanque D, Bouhnik Y, Pelletier AL, Galtier F, Laharie D, Nachury M, Zerbib F, Allez M, Bommelaer G, Duclos B, Lucht F, Gougeon ML, Tartour E, Rozenberg F, Hanslik T, Beaugerie L, Carrat F; MICIVAX Study Group. Immunogenicity and Safety of Influenza Vaccine in Inflammatory Bowel Disease Patients Treated or not with Immunomodulators and/or Biologics: A Two-year Prospective Study. J Crohns Colitis. 2015 Dec;9(12):1096-107.
9. Hagihara Y, Ohfuji S, Watanabe K, Yamagami H, Fukushima W, Maeda K, Kamata N, Sogawa M, Shiba M, Tanigawa T, Tominaga K, Watanabe T, Fujiwara Y, Hirota Y, Arakawa T. Infliximab and/or immunomodulators inhibit immune responses to trivalent influenza vaccination in adults with inflammatory bowel disease. J Crohns Colitis. 2014 Mar;8(3):223-33.
10. Andrisani G, Frasca D, Romero M, Armuzzi A, Felice C, Marzo M, Pugliese D, Papa A, Mocci G, De Vitis I, Rapaccini GL, Blomberg BB, Guidi L. Immune response to influenza A/H1N1 vaccine in inflammatory bowel disease patients treated with anti TNF-α agents: effects of combined therapy with immunosuppressants. J Crohns Colitis. 2013 May;7(4):301-7.
11. Cullen G, Bader C, Korzenik JR, Sands BE. Serological response to the 2009 H1N1 influenza vaccination in patients with inflammatory bowel disease. Gut. 2012 Mar;61(3):385-91.
12. Gelinck LB, van der Bijl AE, Beyer WE, Visser LG, Huizinga TW, van Hogezand RA, Rimmelzwaan GF, Kroon FP. The effect of anti-tumour necrosis factor alpha treatment on the antibody response to influenza vaccination. Ann Rheum Dis. 2008 May;67(5):713-6.
13. Bálint A, Farkas K, Éva PK, Terhes G, Urbán E, Szucs M, Nyári T, Bata Z, Nagy F, Szepes Z, Miheller P, Lorinczy K, Lakatos PL, Lovász B, Tamás S, Kulcsár A, Berényi A, Törocsik D, Daróczi T, Saródi Z, Wittmann T, Molnár T. Antibody and cell-mediated immune response to whole virion and split virion influenza vaccine in patients with inflammatory bowel disease on maintenance immunosuppressive and biological therapy. Scand J Gastroenterol. 2015 Feb;50(2):174-81.
14. Rahier JF, Papay P, Salleron J, Sebastian S, Marzo M, Peyrin-Biroulet L, Garcia-Sanchez V, Fries W, van Asseldonk DP, Farkas K, de Boer NK, Sipponen T, Ellul P, Louis E, Peake ST, Kopylov U, Maul J, Makhoul B, Fiorino G, Yazdanpanah Y, Chaparro M; European Crohn's and Colitis Organisation (ECCO). H1N1 vaccines in a large observational cohort of patients with inflammatory bowel disease treated with immunomodulators and biological therapy. Gut. 2011 Apr;60(4):456-62.

15. Dabestani NM, Leidner AJ, Seiber EE, Kim H, Graitcer SB, Foppa IM, Bridges CB. A review of the cost-effectiveness of adult influenza vaccination and other preventive services. Prev Med. 2019 Sep;126:105734.
16. Leidner AJ, Murthy N, Chesson HW, Biggerstaff M, Stoecker C, Harris AM, Acosta A, Dooling K, Bridges CB. Cost-effectiveness of adult vaccinations: A systematic review. Vaccine. 2019 Jan 7;37(2):226-234.
17. Ting EEK, Sander B, Ungar WJ. Systematic review of the cost-effectiveness of influenza immunization programs. Vaccine. 2017 Apr 4;35(15):1828-1843.

Conclusion – Adults

PICO 14A: In adult patients with IBD (65 years old and younger), should vaccination vs. no vaccination against influenza be given?

Moderate certainty of evidence
Direction – Yes (100%)
Strength – Strong (100%)

**Type of recommendation**	**Strong recommendation against the intervention**	**Conditional recommendation against the intervention**	**Conditional recommendation for either the intervention or the comparison**	**Conditional recommendation for the intervention**	**Strong recommendation for the intervention**
	○	○	○	○	○ (highlighted)
**Recommendation**	**Statement 14A: In adult patients with IBD 65 years of age and younger, we recommend influenza vaccine be given.**				
**Justification**					
**Subgroup considerations**					

**Implementation considerations**	
**Monitoring and evaluation**	• Ongoing monitoring of serious adverse events associated with influenza vaccine in IBD patients
**Research priorities**	• Observational or RCTs to determine the clinical effectiveness of influenza vaccine in adult IBD patients with assessment of patient-important outcomes (i.e. influenza, influenza-like illness etc.)<br>• More RCTs are needed to compare single vs. booster vaccination strategies in adult IBD patients on immunosuppressive medications<br>• RCTs are needed to compare standard vs. high dose influenza vaccine products in adult IBD patients on immunosuppressive medications

PICO 14B: In adult patients with IBD (oolder than 65 years of age), should vaccination vs. no vaccination against influenza be given?

Moderate certainty of evidence
Direction – Yes (100%)
Strength – Strong (100%)

**Type of recommendation**	**Strong recommendation against the intervention**	**Conditional recommendation against the intervention**	**Conditional recommendation for either the intervention or the comparison**	**Conditional recommendation for the intervention**	**Strong recommendation for the intervention**
	○	○	○	○	○ (highlighted)

**Recommendation**	**Statement 14B: In adult patients with IBD older than 65 years of age, we recommend influenza vaccine be given.**
**Justification**	
**Subgroup considerations**	
**Implementation considerations**	
**Monitoring and evaluation**	• Ongoing monitoring of serious adverse events associated with influenza vaccine in IBD patients
**Research priorities**	• Observational or RCTs to determine the clinical effectiveness of influenza vaccine in adult IBD patients with assessment of patient-important outcomes (i.e. influenza, influenza-like illness etc.)<br>• More RCTs are needed to compare single vs. booster vaccination strategies in adult IBD patients on immunosuppressive medications<br>• RCTs are needed to compare standard vs. high dose influenza vaccine products in adult IBD patients on immunosuppressive medications

Evidence to Decision Table – Timing of influenza vaccination relative to anti-TNF therapy

PICO: In patients with IBD on maintenance biologic therapy, should influenza vaccine be timed in relation to the biologic therapy?

Judgement	Research evidence	Additional considerations

<table>
<tr>
<td>Desirable Effects</td>
<td>How substantial are the desirable anticipated effects?

○ Trivial
○ Small
○ Moderate
○ Large

○ Varies
○ Don't know</td>
<td rowspan="2">See Evidence Profile Tables.

Risk of Influenza infection in adult IBD patients

Two observational studies addressed this PICO question.[1,2]

One was a cross-sectional case-control study that used an US administrative database (Nationwide Inpatient Sample) to compare the risks of hospitalization for influenza pneumonia among adult IBD patients vs. non-IBD controls.[1] It is important to note that influenza pneumonia patients treated as outpatients were excluded. After adjusting for various factors including comorbidities, risk factors for pneumonia, as well as patient and hospital characteristics, IBD patients did not demonstrate an increased odd of hospitalization for pneumonia due to influenza virus. However, low income UC patients had an increased odd of hospitalization for pneumonia due to influenza virus (aOR 1.86, CI 1.46-2.37). Mortality during these admissions among IBD patients was not significantly higher than the control population.

The other case-control study used a US commercial administrative database containing inpatient, outpatient, and pharmacy data to assess the risks of influenza and its related complications among adult IBD patients vs. non-IBD controls.[2] This study provided more direct evidence as both outpatients and inpatients (population of interest) treated for influenza infection were captured in this study. After adjusting for health care utilization and comorbid illnesses, IBD patients had an increased risk for influenza infection compared with non-IBD controls (aHR 1.28, CI 1.19-1.37). IBD patients also had significantly more hospitalizations within 30 days of an influenza diagnosis compared with non-IBD controls (5.4% vs. 1.85%, $P < 0.001$). As this study provided more direct evidence than the previous study, the GRADE rating was anchored at this study.

The GRADE rating started at high as it was considered a prognostic study (providing evidence about the likelihood of influenza infection in patients with IBD). The rating was further downgraded to <u>low</u> due to study limitations (residual confounding factors, detection bias, admission bias, and misclassification bias). In particular, patients with IBD and respiratory symptoms may be more likely to be tested for, diagnosed with, and admitted for influenza than non-IBD controls, thus creating an overestimate of the risk of influenza among IBD patients. In summary, there is <u>low</u> certainty evidence that adult IBD patients have an increased risk of influenza infection compared to non-IBD patients.

Effectiveness and safety of Inactivated Influenza Vaccine in adult IBD patients

There was no RCT comparing influenza vaccine with placebo or no treatment in adult patients with IBD to address this PICO question.

There are 2 Cochrane systematic reviews assessing the safety and effectiveness of inactivated influenza vaccine in healthy adults aged 16-65 and in the elderly aged > 65.[3,4] In healthy adults, inactivated influenza vaccines reduce the risk of influenza (RR</td>
<td></td>
</tr>
<tr>
<td>Undesirable Effects</td>
<td>How substantial are the undesirable anticipated effects?

○ Large
○ Moderate
○ Small
○ Trivial

○ Varies
○ Don't know</td>
<td></td>
</tr>
</table>

		0.41, 95% CI 0.36-0.47).[3] The certainty of evidence was moderate due to indirectness for outcome with uncertainty over definition, surveillance and testing of influenza in older trials.[3] The certainty of evidence was also moderate for the outcome of influenza-like illness due to inconsistency. In elderly aged > 65, inactivated influenza vaccines also reduce the risk of influenza (RR 0.42, 95% CI 0.27-0.66).[4] However, the certainty of evidence was low due to indirectness for outcome and study limitations with most of the evidence coming from studies with high or unclear risk of bias for more than one risk of bias domain, but moderate for influenza-like illness due to study limitations.[4]<br><br>There are six cohort studies and four RCTs (observational data) that addressed this PICO question using surrogate outcomes of immunogenicity (seroprotection, seroconversion, GMT fold rise in titer).[5-14] The assessed vaccines include trivalent and quadrivalent inactivated influenza vaccines, as well as the 2009 pandemic H1N1 vaccine. According to the criteria defined by the European Union Committee for Medicinal Products for Human Use (CHMP) for the evaluation of seasonal influenza vaccine immunogenicity in immunocompetent adults aged 18-60 years, at least one of the following serological criteria for Haemagglutination inhibition (HI) antibody response should be achieved: seroprotection (HI titer $\geq$ 40) > 70% (or > 60% in age > 60), seroconversion (at least a 4-fold increase in titer) > 40% (or > 30% in age > 60), or geometric mean titer (GMT) fold rise > 2.5 (or > 2 in age > 60). For pandemic vaccines, all three of the criteria had to be met. However, CHMP criteria for serological response to vaccination are based on healthy volunteers aged 18 to 60 years with attenuated strains, thus may not reflect expected rates of clinical protection observed in other populations (e.g. children, older adults, adults with underlying comorbidities, vaccinated immunocompromised populations). Furthermore, methods of standardization of antibody titres are lacking.<br><br>The evidence suggests that influenza vaccination can induce seroprotection (43-100% achieving HI titer $\geq$ 40), seroconversion (23-76% achieving at least a 4-fold increase in titer), and GMT fold rise (1.77-20.4) in a significant proportion of adult IBD patients. Immunosuppressive medications (e.g. immunomodulators, anti-TNF, steroids) may reduce the immunologic response to influenza vaccination in IBD patients, particularly when multiple immunosuppressive medications are used. However, it is uncertain if this reduced immunologic response is clinically relevant/important and would still afford clinical protection. The GRADE rating started at low due to the observational designs of the studies. The rating was downgraded to very low due to study limitations (selection bias, residual confounding), indirectness (surrogate outcome), inconsistency, and imprecision. In particular, IBD patients who agreed to vaccination were likely to be systematically different than those who did not agree or seek vaccination (healthy vaccinee effect or confounding by indication). This may lead to selection bias confounding the vaccine's effect on the outcomes (e.g. mortality, infection, adverse events, and even immunologic response). As well, the response to influenza vaccine was quite variable, and comparisons between different studies are difficult to make because of annual reformulation of vaccine resulting in differing antigenicity of each year's vaccine. Protective effect of a vaccine is also dependent on whether the circulating strain is included in the vaccine. Additionally, in some years, strains can remain unchanged from previous years. Thus, a study participant who had been vaccinated against the same strain the previous year might have a different response	

than a participant naïve to that strain (depending on pre-vaccination titers). Patient populations were also quite variable across studies with different proportions of patients being on different immunosuppressive medications. This may have contributed to the inconsistency in results across studies. It is important to note that most patients included in the studies were age 18-65. Very few elderly patients (age > 65) were included.

The evidence for effectiveness was anchored to the general population. As immunogenicity studies suggested that inactivated vaccines may be less immunogenic (and therefore less effective) in adult IBD patients, we would also need to need to consider downgrading for indirectness. However, the degree of indirectness was not judged to be severe enough to warrant further downgrading as most studies showed the CHMP criteria for immunogenicity was met in adult IBD populations. Therefore, **the overall certainty of evidence for effectiveness of inactivated influenza vaccine was judged to remain <u>moderate</u> in adult IBD patients aged 16-65 and <u>moderate</u> in elderly IBD patients over age 65.**

The two Cochrane systematic reviews in healthy adults and elderly adults also showed no serious adverse events associated with the use of inactivated influenza vaccine.[3,4] The certainty of evidence for safety was high for healthy adults and moderate for elderly adults.[3,4] No serious adverse events including disease exacerbation was reported in the 10 observational studies in adult IBD patients.[5-14] The certainty of evidence for safety was anchored to the general population, and downgraded for indirectness as sample sizes in the IBD studies were insufficient to detect rare adverse events. **Therefore, the overall certainty of evidence for safety of inactivated influenza vaccine was judged to be <u>moderate</u> in adult IBD patients aged 16-65 and <u>moderate</u> in elderly IBD patients over age 65.**

Overall, there is <u>moderate</u> certainty evidence that inactivated influenza is safe and effective in adult IBD patients aged 16-65 and elderly IBD patients age > 65.

Single vs. booster influenza vaccination:

Two RCTs addressed the question of effectiveness and safety between single vs. booster influenza vaccination in IBD patients.[5,7] Both studies suggested no significant difference in immunogenicity (seroprotection, seroconversion, GMT titer rise) between single vs. booster vaccination strategies in IBD patients. No serious adverse events including disease exacerbation was reported. The GRADE rating started at high. The rating was downgraded to **<u>very low</u>** due to study limitations, indirectness (surrogate outcomes, patient populations), and imprecision. Both studies were conducted in Japan with different formulations of vaccines (2015/2016 seasonal QIV and 2012-2013 seasonal TIV with different vaccine strains). Therefore, results may not be generalizable to other IBD patient populations. As well, surrogate outcomes were used. **In summary, there is <u>very low</u> certainty evidence that there is no significant difference in effectiveness and safety between single vs. booster influenza vaccination in adult IBD patients.**

		**Timing of influenza vaccination relative to anti-TNF therapy:**<br><br>One RCT addressed the question of effectiveness and safety related to timing of influenza vaccination in IBD patients on maintenance infliximab therapy.[6] The study suggested no significant difference in immunogenicity (seroprotection and seroconversion) between vaccination given at the time of infliximab infusion vs. midway between infusions. No serious adverse events were reported. The GRADE rating started at high. The rating was downgraded to **low** due to study limitations and imprecision. **In summary, there is low certainty evidence that timing of influenza vaccination relative to infliximab infusion does not affect the effectiveness and safety of influenza in adult IBD patients.**	
**Certainty of evidence**	**What is the overall certainty of the evidence of effects?**<br><br>○ Very low<br>○ Low<br>○ Moderate<br>○ High<br><br>○ No included studies		
**Values and Preferences**	**Is there important uncertainty about or variability in how much people value the main outcomes?**<br><br>○ Important uncertainty or variability<br>○ Possibly important uncertainty or variability<br>○ Probably no important uncertainty or variability<br>○ No important uncertainty or variability	Patients likely value patient-important outcomes (mortality, VPI, adverse effects) more than surrogate outcomes (immunogenicity).	
**Balance of effects**	**Does the balance between desirable and undesirable effects favor the intervention or the comparison?**<br><br>○ Favors the comparison<br>○ Probably favors the comparison<br>○ Does not favor either the intervention or the comparison<br>○ Probably favors the intervention<br>○ Favors the intervention<br><br>○ Varies<br>○ Don't know		

**Resources required**	**How large are the resource requirements (costs)?**<br><br>○ Large costs<br>○ Moderate costs<br>○ Negligible costs and savings<br>○ Moderate savings<br>○ Large savings<br><br>○ Varies<br>○ Don't know	CDC vaccine price list last reviewed/updated: July 1, 2019<br><br>(see table below)	
**Certainty of Evidence of Required Resources**	**What is the certainty of the evidence of resource requirements (costs)?**<br><br>○ Very low<br>○ Low<br>○ Moderate<br>○ High<br><br>○ No included studies	The costs of delivering routine immunization services may vary widely across countries and different health system settings. See Immunization Costing Action Network (ICAN) Immunization Delivery Cost Catalogue. http://immunizationeconomics.org/ican-idcc.	
**Cost effectiveness**	**Does the cost-effectiveness of the intervention favor the intervention or the comparison?**<br><br>○ Favors the comparison<br>○ Probably favors the comparison<br>○ Does not favor either the intervention or the comparison<br>○ Probably favors the intervention<br>○ Favors the intervention<br><br>○ Varies<br>○ No included studies	There are no studies that addressed this question specifically in the IBD population.	

Brandname	CDC cost/dose	Private sector cost/dose
Fluzone® Quadrivalent	$12.808	$16.939
Fluarix® Quadrivalent	$12.22	$16.82
FluLaval® Quadrivalent	$11.94	$15.77
Flucelvax® Quadrivalent	$15.00	$22.758
Afluria® Quadrivalent	$11.35	$15.871

**Acceptability**	**Is the intervention acceptable to key stakeholders?**<br><br>○ No<br>○ Probably no<br>○ Probably yes<br>○ Yes<br><br>○ Varies<br>○ Don't know		
**Feasibility**	**Is the intervention feasible to implement?**<br><br>○ No<br>○ Probably no<br>○ Probably yes<br>○ Yes<br><br>○ Varies<br>○ Don't know		

References:

1. Stobaugh DJ, Deepak P, Ehrenpreis ED. Hospitalizations for vaccine preventable pneumonias in patients with inflammatory bowel disease: a 6-year analysis of the Nationwide Inpatient Sample. Clin Exp Gastroenterol. 2013 May 6;6:43-9.
2. Tinsley A, Navabi S, Williams ED, Liu G, Kong L, Coates MD, Clarke K. Increased Risk of Influenza and Influenza-Related Complications Among 140,480 Patients With Inflammatory Bowel Disease. Inflamm Bowel Dis. 2019 Jan 10;25(2):369-376.
3. Demicheli V, Jefferson T, Ferroni E, Rivetti A, Di Pietrantonj C. Vaccines for preventing influenza in healthy adults.Cochrane Database Syst Rev. 2018 Feb 1;2:CD001269.
4. Demicheli V, Jefferson T, Di Pietrantonj C, Ferroni E, Thorning S, Thomas RE, Rivetti A. Vaccines for preventing influenza in the elderly. Cochrane Database Syst Rev. 2018 Feb 1;2:CD004876.
5. Shirai S, Hara M, Sakata Y, Tsuruoka N, Yamamoto K, Shimoda R, Gomi Y, Yoshii H, Fujimoto K, Iwakiri R. Immunogenicity of Quadrivalent Influenza Vaccine for Patients with Inflammatory Bowel Disease Undergoing Immunosuppressive Therapy. Inflamm Bowel Dis. 2018 Apr 23;24(5):1082-1091.
6. deBruyn J, Fonseca K, Ghosh S, Panaccione R, Gasia MF, Ueno A, Kaplan GG, Seow CH, Wrobel I. Immunogenicity of Influenza Vaccine for Patients with Inflammatory Bowel Disease on Maintenance Infliximab Therapy: A Randomized Trial. Inflamm Bowel Dis. 2016 Mar;22(3):638-47.
7. Matsumoto H, Ohfuji S, Watanabe K, Yamagami H, Fukushima W, Maeda K, Kamata N, Sogawa M, Shiba M, Tanigawa T, Tominaga K, Watanabe T, Fujiwara Y, Hirota Y, Arakawa T. Booster influenza vaccination does not improve immune response in adult inflammatory bowel disease patients treated with immunosuppressives: a randomized controlled trial. J Gastroenterol. 2015 Aug;50(8):876-86.

8. Launay O, Abitbol V, Krivine A, Slama LB, Bourreille A, Dupas JL, Hébuterne X, Savoye G, Deplanque D, Bouhnik Y, Pelletier AL, Galtier F, Laharie D, Nachury M, Zerbib F, Allez M, Bommelaer G, Duclos B, Lucht F, Gougeon ML, Tartour E, Rozenberg F, Hanslik T, Beaugerie L, Carrat F; MICIVAX Study Group. Immunogenicity and Safety of Influenza Vaccine in Inflammatory Bowel Disease Patients Treated or not with Immunomodulators and/or Biologics: A Two-year Prospective Study. J Crohns Colitis. 2015 Dec;9(12):1096-107.
9. Hagihara Y, Ohfuji S, Watanabe K, Yamagami H, Fukushima W, Maeda K, Kamata N, Sogawa M, Shiba M, Tanigawa T, Tominaga K, Watanabe T, Fujiwara Y, Hirota Y, Arakawa T. Infliximab and/or immunomodulators inhibit immune responses to trivalent influenza vaccination in adults with inflammatory bowel disease. J Crohns Colitis. 2014 Mar;8(3):223-33.
10. Andrisani G, Frasca D, Romero M, Armuzzi A, Felice C, Marzo M, Pugliese D, Papa A, Mocci G, De Vitis I, Rapaccini GL, Blomberg BB, Guidi L. Immune response to influenza A/H1N1 vaccine in inflammatory bowel disease patients treated with anti TNF-α agents: effects of combined therapy with immunosuppressants. J Crohns Colitis. 2013 May;7(4):301-7.
11. Cullen G, Bader C, Korzenik JR, Sands BE. Serological response to the 2009 H1N1 influenza vaccination in patients with inflammatory bowel disease. Gut. 2012 Mar;61(3):385-91.
12. Gelinck LB, van der Bijl AE, Beyer WE, Visser LG, Huizinga TW, van Hogezand RA, Rimmelzwaan GF, Kroon FP. The effect of anti-tumour necrosis factor alpha treatment on the antibody response to influenza vaccination. Ann Rheum Dis. 2008 May;67(5):713-6.
13. Bálint A, Farkas K, Éva PK, Terhes G, Urbán E, Szucs M, Nyári T, Bata Z, Nagy F, Szepes Z, Miheller P, Lorinczy K, Lakatos PL, Lovász B, Tamás S, Kulcsár A, Berényi A, Törocsik D, Daróczi T, Saródi Z, Wittmann T, Molnár T. Antibody and cell-mediated immune response to whole virion and split virion influenza vaccine in patients with inflammatory bowel disease on maintenance immunosuppressive and biological therapy. Scand J Gastroenterol. 2015 Feb;50(2):174-81.
14. Rahier JF, Papay P, Salleron J, Sebastian S, Marzo M, Peyrin-Biroulet L, Garcia-Sanchez V, Fries W, van Asseldonk DP, Farkas K, de Boer NK, Sipponen T, Ellul P, Louis E, Peake ST, Kopylov U, Maul J, Makhoul B, Fiorino G, Yazdanpanah Y, Chaparro M; European Crohn's and Colitis Organisation (ECCO). H1N1 vaccines in a large observational cohort of patients with inflammatory bowel disease treated with immunomodulators and biological therapy. Gut. 2011 Apr;60(4):456-62.
15. Dabestani NM, Leidner AJ, Seiber EE, Kim H, Graitcer SB, Foppa IM, Bridges CB. A review of the cost-effectiveness of adult influenza vaccination and other preventive services. Prev Med. 2019 Sep;126:105734.
16. Leidner AJ, Murthy N, Chesson HW, Biggerstaff M, Stoecker C, Harris AM, Acosta A, Dooling K, Bridges CB. Cost-effectiveness of adult vaccinations: A systematic review. Vaccine. 2019 Jan 7;37(2):226-234.
17. Ting EEK, Sander B, Ungar WJ. Systematic review of the cost-effectiveness of influenza immunization programs. Vaccine. 2017 Apr 4;35(15):1828-1843.

Conclusion – Timing of influenza vaccination relative to anti-TNF therapy

PICO: In patients with IBD on maintenance biologic therapy, should seasonal influenza immunization be timed in relation to the biologic dose?
Low certainty of evidence
Direction – Yes () Uncertain (33%) No (67%)
Strength -

No consensus

Type of recommendation	Strong recommendation against the intervention	Conditional recommendation against the intervention	Conditional recommendation for either the intervention or the comparison	Conditional recommendation for the intervention	Strong recommendation for the intervention
	○	○	○	○	○

**Recommendation**	**No recommendation: In patients with IBD on maintenance biologic therapy, the consensus group could not make a recommendation for or against timing seasonal influenza immunization in relation to the biologic dose.**
**Justification**	
**Subgroup considerations**	
**Implementation considerations**	
**Monitoring and evaluation**	• Ongoing monitoring of serious adverse events associated with influenza vaccine in IBD patients
**Research priorities**	• Observational or RCTs to determine the clinical effectiveness of influenza vaccine in adult IBD patients with assessment of patient-important outcomes (i.e. influenza, influenza-like illness etc.)<br>• More RCTs are needed to compare single vs. booster vaccination strategies in adult IBD patients

	on immunosuppressive medications<br>• RCTs are needed to compare standard vs. high dose influenza vaccine products in adult IBD patients on immunosuppressive medications

We initially divided adults into 2 groups (age 65 years and younger and older than 65) when we assessed the evidence and went through the Evidence-to-Decision framework. Given that the certainty of evidence is moderate and the recommendation is strong for both groups of patients, we have combined them into 1 group for the final recommendation: In adult patients with IBD, we recommend influenza vaccine be given.

Pneumococcal Disease

Background

Streptococcus pneumoniae infections are a major cause of invasive pneumococcal disease (IPD) such as meningitis, sepsis, and pneumonia with bacteremia, as well as milder but more common non-invasive illnesses such as sinusitis and otitis media. IPD is most common in the very young, the elderly and persons at high risk due to underlying medical conditions or lifestyle factors. The case fatality rate of bacteremic pneumococcal pneumonia is 5% to 7% and is higher among elderly persons.

Medical conditions resulting in high risk of IPD include a variety of non-immunocompromising conditions such as CSF leak, chronic neurological condition, cochlear implants, chronic heart disease, diabetes mellitus, chronic kidney disease, chronic liver disease, and chronic disease. As well, **immunocompromising conditions** such as sickle cell disease, congenital immunodeficiencies, immunocompromising therapy including long-term corticosteroids, chemotherapy, radiation therapy, and post-organ transplant therapy, HIV infection, hematopoietic stem cell transplant, malignant neoplasms, nephrotic syndrome, and solid organ or islet transplant.

CDC recommends routine administration of pneumococcal conjugate vaccine (PCV13) for all children younger than 2 years of age in a series of 4 doses.[1] Children age 2 through 4 years old who are unvaccinated or received an incomplete PCV13 series should still get 1 dose of PCV13.[1] The number of doses recommended and the intervals between doses will depend on the child's age when vaccination begins. For children over the age of 2 and adults with certain medical conditions such as chronic heart disease, chronic lung disease, diabetes, cerebrospinal fluid leaks, cochlear implants, sickle cell disease or other hemoglobinopathies, congenital or acquired asplenia or splenic dysfunction, HIV infection, chronic renal failure or nephrotic syndrome, **diseases associated with**

treatment with immunosuppressive drugs or radiation therapy including malignant neoplasm, leukemia, lymphomas, and Hodgkin's disease, or solid organ transplantation, and congenital immunodeficiency, **should receive both PCV13 and pneumococcal polysaccharide (PPSV23) vaccine**.[1] For adults 65 years or older, CDC recommends PCV13 who have not previously received a dose, and PPSV23.[1] Up to 98% of the pneumococcal serotypes that cause pneumonia in the industrialized world are contained in the PPSV23 vaccine.

Similarly, NACI recommends PCV13 vaccine for infants and children up to 5 years of age routinely, children over 5 years old at high risk of IPD due to underlying medical conditions, adults with immunocompromising conditions resulting in high risk of IPD, and residents of hematopoietic stem cell transplant (HSCT).[2] PPSV23 vaccine is recommended for all individuals 24 months of age and older who are at high risk of IPD due to an underlying medical condition, who are residents of long-term care facilities, adults 65 years and older (regardless of risk factors or previous pneumococcal vaccination), and adults at high risk of IPD due to lifestyle factors.[2] PPSV23 is not approved for use in children younger than 2 years of age because children in this age group do not develop an effective immune response to capsule types contained in the polysaccharide vaccine.

Specifically, in adults with immunocompromising condition (except HSCT), immunocompetent persons who might be anticipating initiation of immunocompromising treatments, individuals on immunosuppressive therapy, NACI recommends 1 dose of Pneu-C-13 vaccine followed at least 8 weeks later by 1 dose of Pneu-P-23 vaccine, if not previously received.[2] A booster dose of Pneu-P-23 vaccine should be given at least 5 years later. The dose of Pneu-C-13 vaccine should be administered at least 1 year after any previous dose of Pneu-P-23 vaccine. The rationale of this prime-boost schedule is that PCV13 causes a T-cell dependent immune response, leading to the formation of immunological memory. Subsequent PPSV23 administration boosts the response to the serotypes that are present in both vaccines, while simultaneously broadening the serotype spectrum.

The IDSA guidelines recommend PCV13 be administered to adults and children with a chronic inflammatory illness that is being treated with immunosuppression (strong, very low-moderate). PPSV23 should be administered to patients aged > 2 years with chronic inflammatory illnesses with planned initiation of immunosuppression (strong, low), low level immunosuppression (strong, low), and high-level immunosuppression (strong, very low). A second dose of PPSV23 should be given 5 years later (strong, low).[3]

A booster dose of Pneu-C-13 is not necessary because there is currently no evidence that a booster dose is beneficial. A booster dose of Pneu-P-23 vaccine is recommended for individuals of any age in whom antibody response is decreased due to: functional or anatomic hyposplenia or asplenia, including sickle cell disease; chronic liver disease, including hepatic cirrhosis; chronic kidney failure or nephrotic syndrome; and immunosuppression related to disease or therapy (i.e. individuals at highest risk of IPD). If a

booster dose of Pneu-P-23 vaccine is recommended, it should be administered at least 5 years after any previous dose of Pneu-P-23 vaccine.

Serologic testing is not recommended before or after receiving pneumococcal vaccine.

References:

1. Centers for Disease Control and Prevention https://www.cdc.gov/vaccines/vpd/pneumo/hcp/who-when-to-vaccinate.html
2. Public Health Agency of Canada. https://www.canada.ca/en/public-health/services/publications/healthy-living/canadian-immunization-guide-part-4-active-vaccines/page-16-pneumococcal-vaccine.html
3. Rubin LG, Levin MJ, Ljungman P, Davies EG, Avery R, Tomblyn M, Bousvaros A, Dhanireddy S, Sung L, Keyserling H, Kang I; Infectious Diseases Society of America. 2013 IDSA clinical practice guideline for vaccination of the immunocompromised host. Clin Infect Dis. 2014 Feb;58(3):e44-100.

Risk of Pneumococcal Infection in IBD patients

PICO: What is the risk of pneumococcal infection in people with IBD compared to people without IBD?

Summary – Adults and Pediatrics

One systematic review and five observational studies addressed this PICO question.[1-6]

One systematic review assessed the incidence of invasive pneumococcal disease (IPD) in immunocompromised patients with inclusion of five studies on patients with chronic inflammatory disease such as SLE, Sjogren's syndrome, polymyositis/dermatomyositis, COPD, asthma, RA, and IBD.[1] No subgroup data was provided for IBD.[1] Compared to healthy control cohorts (pooled incidence rate: 10/100,000 person-years), the incidence rate of IPD was increased in patients with chronic inflammatory disease (pooled incidence rate: 65/100,000 person-years).[1] One observational study found an increased risk of IPD among patients with autoimmune diseases (RA, SLE, CD) vs. healthy adults.[2] Two other observational studies also found an increased risk of IPD among IBD patients compared to non-IBD controls.[3,4] **Compared to non-IBD controls, the risk of IPD is about 1.5- to 2-fold higher in IBD patients.**

One cross-sectional case-control study used an administrative database (Nationwide Inpatient Sample) to compare the risks of hospitalization for pneumonia due to *Streptococcus pneumoniae* among adult IBD patients vs. non-IBD controls.[5] It is important to

note that pneumonia treated as outpatients were excluded. After adjusting for various factors including comorbidities, risk factors for pneumonia, as well as patient and hospital characteristics, IBD patients did not demonstrate increased odds of hospitalization for *Streptococcus pneumoniae* compared to non-IBD controls.[5] Mortality during these admissions among IBD patients was not significantly higher than the control population.[5]

One retrospective cohort and nested case-control study found an increased risk of pneumonia among IBD patients compared to non-IBD patients after adjusting for age, disease type, health care utilization and comorbidities.[6] Use of biologic medications, corticosteroids, and PPI were significantly associated with pneumonia.[6] As the most common etiologic agent of community acquired pneumonia in the US is pneumococcal pneumonia, the evidence was not downgraded for indirectness (outcome).

The GRADE rating started at high as these were considered prognostic studies (providing evidence about the likelihood of Streptococcal infection in patients with IBD). The rating was further downgraded to **low** due to study limitations (residual confounding factors, detection bias, admission bias, and misclassification bias). In particular, patients with IBD and respiratory symptoms may be more likely to be tested for, diagnosed with, and admitted for *Streptococcus pneumoniae* infection than non-IBD controls, thus creating an overestimate of the risk of Streptococcal infection among IBD patients. **In summary, there is low certainty evidence that adult IBD patients have an increased risk of pneumococcal infection compared to non-IBD patients.**

One observational study included both adult and pediatric IBD patients found an increased risk of IPD among IBD patients compared to non-IBD controls.[3] No subgroup data was provided for pediatric patients. Compared to non-IBD controls, the risk of IPD is about 1.5- to 2-fold higher in IBD patients. **There is low certainty evidence that pediatric IBD patients have an increased risk of pneumococcal infection compared to non-IBD patients.**

Risk of Bias Table

Prognostic studies							
**Study**	**Study sample adequately represents the population of**	**Study data available adequately represent**	**Prognostic factor measured in a similar and**	**Outcome of interest is measured in a similar and**	**Important potential confounding factors are appropriately accounted for**	**Statistical analysis is appropriate, and all**	**Comments**

	interest	the study sample (>80% follow-up)	valid way for all participants	valid way for all participants		primary outcomes are reported	
Van Aalst 2018 (SR)	No<br><br>Most included patients had HIV, transplantation, and other chronic inflammatory diseases (e.g. SLE, RA). Only a minority had IBD	OK	Unlikely across studies	Unlikely across studies	No adjustment for disease severity, use of immunosuppressive medications, comorbidities, and other risk factors for IPD	OK	• SR of IPD in immunocompromised patients<br>• 5 studies included patients with chronic inflammatory disease (RA, SLE, Sjogren's, polymyositis/dermatomyositis, COPD, asthma, IBD-minority)<br>• **Pooled IPD incidence rate in the population with chronic inflammatory disease was 65 / 100,000 person years (95% CI 36.8-114.2) v**s. healthy control 10/100,000; HIV 331/100,000 person-years in non-African countries; autologous or stem cell transplant 696 and 812/100,000<br>• **Included Kantso study** – only study that provided subgroup data for IBD patients
Kantso 2015 (Denmark) Adults and Pediatric	OK<br><br>Included both outpatients and inpatients	OK	OK	IPD identified through the register for national surveillance of IPD – positive culture for S. Pneumoniae from blood, CSF, or other	Not adjusted for comorbidities or disease activity may lead to overestimation of the risk of IPD in IBD.<br><br>No adjustment for pneumococcal vaccination.	OK	• <u>Population based study</u> of 74,156 IBD patients (both hospitalized and outpatients) and 1,482,363 non-IBD controls matched by gender, age, and area of residence from 1977-2013<br>• IBD patients had a significantly higher risk of

				normally sterile sites	<u>Detection bias:</u> Patients with IBD may have more outpatient visits and hospitalization than non-IBD controls. This may lead to overestimation of the risk of IPD in IBD patients (and vice versa for the risks of IPD prior to the diagnosis of IBD).		IPD than controls. CD (HR 1.99, 95% CI 1.59-2.49). UC (HR 1.46, 95% CI 1.25-1.69)<br>• IBD medication use including anti-TNF had limited impact on the risk of IPD, except azathioprine in UC (HR 2.38, 95% CI 1.00-5.67)<br>• Up to 4 years prior to IBD diagnosis, increased risk of IPD (UC HR 1.51, 95% CI 1.05-2.17; CD HR 1.79, 95% CI 1.05-3.03)
Shea 2014 (US) Adults	OK<br><br>Included only private health claims data<br><br>Persons with public or no health insurance were not represented.	OK	OK	Data were reliant on administrative codes. (Possible <u>misclassification errors</u> due to errors of miscoding, and the codes have not been previously validated.	Not adjusted for disease activity or use of immunosuppressants may lead to overestimation of the risk of IPD in IBD.<br><br>No adjustment for pneumococcal vaccination or smoking.<br><br><u>Detection bias:</u> Patients with IBD may have more outpatient visits and hospitalization than healthy controls. This may lead to overestimation of the risk of IPD in IBD patients.	OK	• Retrospective cohort study using data from 3 private healthcare claims repositories (2006-2010 to compare rates of pneumococcal disease in immunocompetent adults with chronic medical condition (“at-risk”) and immunocompromised adults (“high-risk”) and “healthy” adults<br>• **Increased rates of all-cause pneumonia among persons with autoimmune diseases** (RA, SLE, CD): RR 4.1 (4.0-4.3) for aged 18-49, 4.0 (3.9-4.0) for aged 50-64, and 3.5 (3.4-3.5) for aged <u>></u>65<br>• **Increased rates of pneumococcal pneumonia and IPD among persons with autoimmune diseases**

Stobaugh 2013 (US) Adults	Study included only hospitalized patients, and did not capture patients treated as outpatients. <u>Prevalence-incidence (Neyman) bias</u>: Exclusion of individuals with severe (fatal prior to admission) or mild disease (not requiring admission) may result in a systematic error in the estimated association or effect of IBD on the risk of hospitalization for Streptococcus pneumoniae.	OK	Data were reliant on administrative discharge diagnoses. Possible <u>misclassification errors</u> due to errors of miscoding, and the codes have not been previously validated.	Data were reliant on administrative discharge diagnoses. Possible <u>misclassification errors</u> due to errors of miscoding, and the codes have not been previously validated.<br><br><u>Detection bias and admission rate bias:</u> patients with IBD and pneumonia may be more likely to be tested and admitted for Streptococcus pneumoniae than controls, thus creating an overestimate of the prevalence of Streptococcus pneumoniae pneumonia among admitted IBD patients.	Case-mix adjustment was performed using the updated Elixhauser Agency for Health-care Research and Quality-Web ICD-9-CM comorbidity algorithms, well-described risk factors for pneumonia, as well as patient and hospital characteristics.<br><br><u>Possible residual confounding factors:</u> medication use, vaccination status, severity and activity of underlying disease (e.g. sicker IBD patients on immunosuppressives may be more likely to be admitted than less sick IBD patients).	OK	• Cross-sectional case-control study (6-year analysis) on the Nationwide Inpatient Sample to assess the risk of hospitalizations for vaccine preventable pneumonias (Streptococcus pneumoniae) among IBD patients vs. non-IBD patients<br>• <u>Cases:</u> All adult patients hospitalized with a secondary diagnosis of IBD<br>• <u>Control:</u> random sample of hospitalized adult patients without a primary or secondary diagnosis of IBD<br>• **IBD patients did not have increased odds of being admitted for S. pneumoniae pneumonia (AOR 1.08; CI 0.99-1.17) vs. non-IBD control.**<br>• **No difference in mortality between IBD patients vs. non-IBD patients**

Long 2013 (US) Adults	OK<br><br>Included only private health claims data<br><br>Persons with public or no health insurance were not represented<br><br>Elderly (age > 65) were also not included.	OK	Data were reliant on administrative discharge diagnoses. Possible misclassification errors due to errors of miscoding, and the codes have not been previously validated	Data were reliant on administrative discharge diagnoses. Possible misclassification errors due to errors of miscoding, and the codes have not been previously validated.	Accounted for health care utilization, comorbidities, region of country, age, disease type.<br><br>Did not adjust for disease activity/severity, vaccination status, comorbidities.	OK	• Retrospective cohort and nested case-control study of 108,604 IBD patients vs. 434,416 non-IBD patients (age < 64) with median follow-up 24 mos<br>• **Increased risk of pneumonia in IBD vs. non-IBD controls** (AHR 1.54, 95% CI 1.49-1.60)<br>• **Use of biologic meds (OR 1.28, 95 %CI 1.08-1.52), steroids (OR 3.62, 95% CI 3.30-3.98), and PPI (OR 1.14, 95% CI 1.03-1.25) significantly associated with pneumonia**
Wotton 2012 (UK) Adults and Pediatric	Study included only hospitalized patients, and did not capture patients treated as outpatients. Prevalence-incidence (Neyman) bias: Exclusion of individuals with severe (fatal prior to admission) or mild disease (not requiring admission) may result in a systematic error	OK	Data were reliant on administrative discharge diagnoses. Possible misclassification errors due to errors of miscoding, and the codes have not been previously validated	Data were reliant on administrative discharge diagnoses. Possible misclassification errors due to errors of miscoding, and the codes have not been previously validated	Accounted for age, sex, calendar year of admission and district of residence.<br><br>Did not account for health care utilization, disease activity or severity, use of IS, or vaccination status.<br><br>Detection bias: Patients with IBD may have more hospitalization than healthy controls. This may lead to overestimation of the risk of	OK	• Retrospective cohort study using linked hospital data in the UK assessing the risk of IPD in patients admitted to hospital with immune-mediated diseases from 1999-2008<br>• Reference cohort was admission with various other, mainly minor, medical and surgical conditions<br>• Increased risk of IPD in patients with immune-mediated diseases. CD RR 2.2 (2.1-2.3)

	in the estimated association or effect of IBD on the risk of hospitalization for Streptococcus pneumoniae.				pneumococcal disease in IBD patients.		

CD: Crohn's disease
CSF: cerebrospinal fluid
IPD: invasive pneumococcal disease
IS: immunosuppressive medications
RA: rheumatoid arthritis
SLE: systematic lupus erythematosus

Evidence Profile Table

**Certainty Assessment**								**Summary of Findings**	
Studies	Risk of bias	Inconsistency	Indirectness	Imprecision	Other considerations	Certainty of Evidence	Overall Certainty of evidence	Study Event Rates	Relative Effect (95% CI)
**VPI (Invasive Pneumococcal Disease) - CRITICAL**							⊕⊕⊖⊖ LOW[g]		
1 SR[1]<br>3 Observational studies[2-4]	Very serious[a]	Not serious	Not serious[b]	Not serious	None	⊕⊕⊖⊖<br>LOW		• See **Summary of observational studies assessing the risks of pneumococcal infection in IBD vs. non-IBD patients**<br>• Compared to non-IBD controls, the risk of invasive pneumococcal disease (IPD) is higher in IBD patients (about 1.5- to 2-fold increase in risk)	
**VPI (hospitalization for pneumonia due to S. pneumoniae) - CRITICAL**									
1 Observational study[5]	Very serious[c]	Not serious	Serious[d]	Not serious	None	⊕⊖⊖⊖<br>VERY LOW		• See **Summary of observational studies assessing the risks of pneumococcal infection in IBD vs. non-IBD patients**<br>• Compared to non-IBD controls, there is no increased odds of	

								hospitalization for pneumonia due to S. pneumoniae
**VPI (pneumonia) - CRITICAL**								
1 Observational study[6]	Very serious[e]	Not serious	Not serious[f]	Not serious	None	⨁⨁⊝⊝ LOW		• See **Summary of observational studies assessing the risks of pneumococcal infection in IBD vs. non-IBD patients**<br>• Compared to non-IBD controls, the risk of pneumonia is higher in IBD patients (about 1.5-fold increase in risk)<br>• Among IBD patients, increased risk of pneumonia with:<br>Biologic use: OR 1.28 (1.08-1.52)<br>Steroid use: OR 3.62 (3.30-3.98)<br>PPI use: OR 1.14 (1.03-1.25)

Footnotes:

a. Downgraded for study limitations. Possible residual confounding factors including medication use (e.g. immunosuppressives or biologics), vaccination status, smoking, as well as severity and activity of IBD may over-estimate the risk of IPD in IBD patients compared to controls. High risk for detection and admission bias as patients with IBD may be more likely to be tested for and diagnosed with IPD than controls, thus creating an overestimate of the prevalence of IPD among IBD patients. Data were reliant on administrative discharge diagnoses. Possible misclassification errors due to errors of miscoding, and the codes have not been previously validated.
b. Not downgraded for indirectness although 1 observational study included patients with autoimmune diseases (no subgroup data for IBD patients).
c. Downgraded for study limitations. Possible residual confounding factors including medication use (e.g. immunosuppressives or biologics), vaccination status, as well as severity and activity of IBD may over-estimate the risk of hospitalization for pneumonia due to S. pneumoniae in IBD patients compared to controls. High risk for detection and admission bias as patients with IBD and pneumonia may be more likely to be tested and admitted for S. pneumoniae than controls, thus creating an overestimate of the prevalence of S. pneumoniae pneumonia among admitted IBD patients. Data were reliant on administrative discharge diagnoses. Possible misclassification errors due to errors of miscoding, and the codes have not been previously validated.
d. Downgraded for indirectness. Study included only a highly selected population (hospitalized patients), and did not capture pneumonia patients treated as outpatients. Hence, the risk of S. pneumoniae infection among all IBD patients (population of interest) vs. non-IBD patients is unknown.
e. Downgraded for study limitations. Possible residual confounding factors including vaccination status, smoking, severity and activity of IBD may over-estimate the risk of pneumonia in IBD patients compared to controls. High risk for detection and admission bias as patients with IBD may be more likely to be tested for and diagnosed with pneumonia than controls, thus creating an overestimate of the prevalence of pneumonia among IBD patients. Data were reliant on administrative discharge diagnoses. Possible misclassification errors due to errors of miscoding, and the codes have not been previously validated.
f. Not downgraded for indirectness. Outcome was pneumonia although the most common etiologic agent of community acquired pneumonia in the US is pneumococcal pneumonia.
g. Overall certainty of evidence was based on the outcome of VPI (invasive pneumococcal disease) due to higher methodological quality of the included studies for this outcome.

Summary of observational studies assessing the risk of pneumococcal infection in IBD vs. non-IBD patients

Study	IBD patients	Non-IBD control	Incidence rates of pneumococcal infection in IBD patients	Incidence rates of pneumococcal infection in non-IBD controls	Adjusted Incidence rate ratios (IRR) / Hazards Ratio (HR) for pneumococcal infection in IBD patients (95% CI)
Van Aalst 2018[1] (SR 5 observational studies of chronic inflammatory disease)	-	-	65 per 100,000 person-years IPD in population with chronic inflammatory disease	10 per 100,000 person-years IPD in healthy control	Increased risk of IPD in population with chronic inflammatory disease
Kantsø 2015[3] (Denmark) Adults and Pediatric	74,156	1,482,363	0.37% IPD	0.27% IPD	Increased risk of IPD in IBD patients:<br>CD: HR 1.99 (1.59-2.49)<br>UC: HR 1.46 (1.25-1.69)
Shea 2014[2] (US) Adults	Autoimmune diseases (RA, CD, SLE)<br>238,225 person-years for age 18-49<br><br>341,148 person-years for age 50-64<br><br>162,206 person-years for age $\geq$ 65	42,472,513 person-years for age 18-49<br><br>20,972,935 person-years for age 50-64<br><br>5,389,930 person-years for age > 65	13 per 100,000 person-years IPD for age 18-49<br><br>21.1 per 100,000 person-years IPD for age 50-64<br><br>33.3 per 100,000 person-years IPD for age $\geq$ 65	1.8 per 100,000 person-years IPD for age 18-49<br><br>4.5 per 100,000 person-years IPD for age 50-64<br><br>8.3 per 100,000 person-years IPD for age $\geq$ 65	Increased risk of IPD in persons with autoimmune diseases (no subgroup data for IBD):<br>RR 7.1 (4.9-10.1) for age 18-49<br><br>RR 4.7 (3.7-6.0) for age 50-64<br><br>RR 4.0 (3.0-5.3) for age $\geq$ 65
Stobaugh 2013[5] (US) Adults	918,557 patient discharges	48,087,002 patient discharges	82.6 per 100,000 Hospitalization for S. pneumoniae	69.2 per 100,000 Hospitalization for S. pneumoniae	No increased odds of hospitalization for pneumonia due to S. pneumoniae<br>CD: AOR 1.08 (0.99-1.17)<br>UC: AOR 0.93 (0.82-1.06)
Long 2013[6] (US) Adults	108,604	434,416	138 per 10,000 pneumonia	76 per 10,000 Pneumonia	Increased risk of pneumonia in IBD patients:<br>Overall: AHR 1.54 (1.49-1.60)<br>CD: AHR 1.71 (1.62-1.80)<br>UC: AHR 1.41 (1.34-1.48)

					Among IBD patients, increased risk of pneumonia:<br>Biologic use: OR 1.28 (1.08-1.52)<br>Steroid use: OR 3.62 (3.30-3.98)<br>PPI use: OR 1.14 (1.03-1.25)
Wotton 2012[4]<br>(US)<br>Adults	244,364<br>Patient admission	-	-	-	Increased risk of IPD in IBD patients:<br>CD: RR 2.25 (2.14-2.35)<br>UC: RR 1.70 (1.63-1.77)

CD: Crohn's disease
IPD: invasive pneumococcal disease
RA: rheumatoid arthritis
SLE: systematic lupus erythematosus

References:

1. van Aalst M, Lötsch F, Spijker R, van der Meer JTM, Langendam MW, Goorhuis A, Grobusch MP, de Bree GJ. Incidence of invasive pneumococcal disease in immunocompromised patients: A systematic review and meta-analysis. Travel Med Infect Dis. 2018 Jul - Aug;24:89-100.
2. Shea KM, Edelsberg J, Weycker D, Farkouh RA, Strutton DR, Pelton SI. Rates of pneumococcal disease in adults with chronic medical conditions. Open Forum Infect Dis. 2014 May 27;1(1):ofu024.
3. Kantsø B, Simonsen J, Hoffmann S, Valentiner-Branth P, Petersen AM, Jess T. Inflammatory Bowel Disease Patients Are at Increased Risk of Invasive Pneumococcal Disease: A Nationwide Danish Cohort Study 1977-2013. Am J Gastroenterol. 2015 Nov;110(11):1582-7.
4. Wotton CJ, Goldacre MJ. Risk of invasive pneumococcal disease in people admitted to hospital with selected immune-mediated diseases: record linkage cohort analyses. J Epidemiol Community Health. 2012 Dec;66(12):1177-81.
5. Stobaugh DJ, Deepak P, Ehrenpreis ED. Hospitalizations for vaccine preventable pneumonias in patients with inflammatory bowel disease: a 6-year analysis of the Nationwide Inpatient Sample. Clin Exp Gastroenterol. 2013 May 6;6:43-9.
6. Long MD, Martin C, Sandler RS, Kappelman MD. Increased risk of pneumonia among patients with inflammatory bowel disease. Am J Gastroenterol. 2013 Feb;108(2):240-8.

Effectiveness and Safety of Pneumococcal Vaccine in IBD patients

Summary - Pediatric

**PICO 15**	**In pediatric patients with IBD, should vaccination vs. no vaccination against pneumococcal disease be given?**
**Population**	Pediatric patients with IBD
**Intervention**	Vaccination against pneumococcal disease

**Comparator**	No vaccination against pneumococcal disease
**Outcome**	Mortality, VPI (pneumococcal infection), SAEs, Immunogenicity

There was no RCT comparing pneumococcal vaccine with placebo or no treatment in pediatric patients with IBD to address this PICO question.

There are two observational studies that addressed this PICO question.[1,2] One cohort study assessed the immunogenicity of PCV13 in 122 IBD patients with IBD aged 5 to 18 years.[1] No significant difference in the rate of adequate vaccine response to PCV13 was found between IBD patients (majority on immunosuppressive medications) and healthy controls.[1] However, children with IBD on anti-TNF or immunomodulators had lower geometric mean titer rises than children with IBD on no immunosuppressive therapy.[1] A small cohort study assessed the immunogenicity of PPSV23 in 18 pediatric patients with IBD.[2] 55.5% of IBD patients were found to be hypo-responsive to PPSV23.[2] It is important to note that consensus on the correlates of clinical protection is lacking. It is therefore uncertain if this reduced immunologic response is clinically relevant/important and would still afford clinical protection. The evidence suggests that PCV13 can induce a serological response in a significant proportion of pediatric IBD patients. However, vaccination response to PPSV23 may be impaired in pediatric IBD patients. Furthermore, immunosuppressive medications may reduce the immunologic response to pneumococcal vaccination in pediatric IBD patients. No serious adverse events were reported with vaccination. The GRADE rating started at low due to the observational designs of the studies. The rating was downgraded to **very low** due to study limitations (selection bias, residual confounding), indirectness (surrogate outcome), and imprecision.

A Cochrane systematic review of 6 RCTs conducted in healthy children less than 2 years of age found pneumococcal vaccines (PCV 7, 9, 11) to be effective in preventing IPD (RR 0.20, 95% CI 0.10-0.42), X-ray defined pneumonia (RR 0.81, 95% CI 0.66-1.00), and clinical pneumonia (RR 0.94, 0.91-0.98).[3] **The certainty of evidence for effectiveness of pneumococcal vaccines in healthy children less than 2 years old was high, but was downgraded to <u>moderate</u> for IBD patients less than 2 years old and <u>low</u> for IBD patients older than 2 years old.**

CDC evaluated the evidence of pneumococcal vaccines for immunocompromised children aged 6 through 18 years. Due to the limited body of evidence on vaccine efficacy and safety among persons with immunocompromising conditions, PCV13 vaccine was evaluated using data for HIV-infected children (1 RCT of PCV9 among HIV infected children in South Africa, 1 RCT of PCV7 among HIV-infected adults in Malawi), observational studies of PCV7 in children with sickle cell disease, and immunogenicity studies of PPSV23.[4] Overall, the quality of evidence was rated as **low for PCV13 among immunocompromised children**. However, the desirable consequences were deemed to clearly outweigh undesirable consequences given the very high burden of pneumococcal diseases

among immunocompromised children. There was **uncertainty regarding costs/benefits of PCV13 vs. PPSV23 in these patients.** However, **the CDC concluded that broader serotype protection can be achieved through the use of both PCV13 and PPSV23 among immunocompromised children**; 49% of IPD in this group is caused by PCV13 serotypes, and an additional 23% by serotypes in PPSV23 not included in PCV13. Evidence from immunogenicity studies demonstrate that antibody response is non-inferior or superior when PCV13 is given before PPSV23 compared to PPSV23 before PCV13. CDC recommends the use of both vaccines among immunocompromised children. This evidence was not included in the evidence profile table as patients with HIV or sickle cell disease were determined *a priori* to be significantly different than IBD patients.

In terms of safety, a Cochrane systematic review found serious adverse events causally related to vaccination (PCV7, PCV 10/11, PCV7/9, PCV6 + TIV) to be rare in children up to 12 years old, and did not differ significantly between groups.[5] **The certainty of evidence for safety of pneumococcal vaccines was high, but was downgraded to moderate for pediatric IBD patients due to indirectness (sample sizes in the IBD studies were insufficient to detect rare adverse events).**

Overall, there is <u>moderate</u> and <u>low</u> certainty evidence that pneumococcal vaccines are safe and effective in pediatric IBD patients age less than 2 and older than or equal to 2, respectively.

Risk of Bias Table – Pediatric

Before-After (Pre-Post) Studies									
Study	Was there a concurrent comparator group that did not receive the intervention	If a concurrent comparator group was used, was it similar to the intervention group (or adequately adjusted) for prognostic factors	If no concurrent comparator group was used		Outcome detection methods valid and similar among compared groups / periods	Incomplete outcome data assessed	Selective outcome reporting	Other bias	Comments
			If each participant served as his/her own control (assessed before vs. after the intervention), are there	If two different consecutive cohorts of participants were assessed (before vs. after implementation of the intervention), are there (a)					

			**compelling arguments that the outcome was not influenced by historic events / underlying secular trends**	**compelling arguments that the outcome was not influenced by historic events / underlying secular trends and (b) evidence that the two groups were similar (or adequately adjusted) for prognostic factors**					
Banaszkiewicz 2015 (Poland)	No	Did not adjust for disease type, disease activity, or treatment subgroup.	OK	NA	OK	OK	OK	Patients who consented to receive vaccination were likely to be prognostically different than patients who did not agree to participate (healthy vaccinee effect)	• Prospective cohort study of 122 IBD patients (age 5-18) on no IS , anti-TNF or IM (steroids 2mg/kg/day for ≥ 2 weeks, AZA, 6MP, cyclosporine, IFX, ADA) , and healthy controls (56)<br>• All received PCV13<br>• Seroconversion at 6-8 weeks post-vaccination<br>• Adequate vaccine response defined as post-immunization antibody of ≥ 0.35 ug/mL to all 13 serotypes as per WHO recommendation

									• **No significant difference in the rate of adequate vaccine response between IBD patients and controls**<br>• **Children on no IS had higher GMT rises than children on anti-TNF or IM**<br>• **No vaccine related serious AEs**
Fallahi 2014 (Iran)	No	Did not adjust for disease type, disease activity, or treatment subgroup.	OK	NA	OK	OK	OK	Possible selection bias. Uncertain how patients were selected into this study from a tertiary care center in Iran	• Cohort study of 18 pediatric IBD patients (mean age 10.7 +/- 4.2) vs 20 healthy controls<br>• 15/18 patients on IS (6MP, AZA, +/- steroids), 3/18 on 5ASA<br>• All received PPSV23<br>• Seroresponse defined as total IgG antibody titer =/> lower limit of 2-tailed 90% probability interval of postimmunization IgG of healthy adults (129U/mL) 28 days post-vaccination<br>• Mean increased level of IgG after

									vaccination was lower in IBD patients vs. controls<br>• 10/18 IBD patients hyporesponsive to the vaccine

6MP – 6 mercaptopurine
ADA – adalimumab
AZA - azathioprine
GMT – geometric mean titers
IFX - infliximab
IM – immunomodulators
IS – immunosuppressants

Evidence Profile Table - Pediatric

Certainty Assessment								Summary of Findings	
Studies	Risk of bias	Inconsistency	Indirectness	Imprecision	Other considerations	Certainty of Evidence	Overall Certainty of evidence	Study Event Rates	Relative Effect (95% CI)
**VPI (IPD) - CRITICAL**							⊕⊕⊕⊖ MODERATE For age < 2<br><br>⊕⊕⊖⊖ LOW For age > 2		
1 SR of 7 RCTs[3]<br><br>**Children < age 2** (PCV 7, 9, 11)	Not serious	Not serious	Serious[a]	Not serious	None	⊕⊕⊕⊖ MODERATE For age < 2<br><br>⊕⊕⊖⊖ LOW For age > 2		• Vaccine serotype IPD: RR 0.20 (95% CI 0.10-0.42)<br>• All serotypes IPD: RR 0.42 (0.25-0.71)	
**Immunogenicity (Seroresponse as defined by primary studies) - IMPORTANT**									

2 Observational studies[1,2]<br><br>**IBD populations**<br>(PCV13, PPSV23)	Serious[b]	Not serious	Serious[c]	Serious[d]	None	⊕⊖⊖⊖<br>VERY LOW		• See **Summary of observational studies assessing the seroresponse rates of pneumococcal vaccines in pediatric IBD patients**<br>• Seroresponse rates to PCV13 was 90.4%[1]<br>• Seroresponse rates to PPSV23 was 44.5%[2]
**Adverse events - CRITICAL**								
1 SR of 9 RCTs[5]<br><br>**Healthy children up to 12 years old**<br><br>(PCV 7, PCV 10/11, PCV7/9, PCV6 + TIV)	Not serious	Not serious	Serious[e]	Not serious	None	⊕⊕⊕⊖<br>MODERATE		• Serious adverse events judged causally related to vaccination (PCV 7, PCV 10/11, PCV7/9, PCV6 + TIV) were rare and did not differ significantly between groups.<br>• No fatal serious adverse event.
1 Observational study[1]<br><br>**IBD populations**<br>PCV13	Serious[b]	Not serious	Not serious	Serious[d]	None	⊕⊖⊖⊖<br>VERY LOW		• No serious adverse events

Footnotes:

a. Downgraded for indirectness. Immunogenicity studies suggested that the pneumococcal vaccines may not be as immunogenic (and therefore as effective) in the IBD populations as in the general population. Studies only included children age < 2. Downgraded 1 more level if evidence is applied to children age > 2.
b. Downgraded for study limitations. Selection bias: vaccine may be selectively given to healthier patients (healthy vaccinee effect) or sicker patients. This may have led to over- or under-estimation of the protective effect of the vaccine depending on the direction of bias. Possible residual confounding factors: did not adjust for disease activity or severity, comorbidities, medications.
c. Downgraded for indirectness. Definitions of seroresponse were highly variable across studies. Surrogate outcome of seroresponse was used to estimate clinical efficacy or effectiveness. However, consensus on the correlates of protection for pneumococcal vaccine is lacking. Studies also included varying proportions of patients on no immunosuppressives or different immunosuppressive medications.
d. Downgraded for imprecision as small sample sizes for each subgroup of patients on different immunosuppressive medications
e. Downgraded for indirectness. Healthy children up to 12 years old. Not IBD patients

Summary of observational studies assessing the seroresponse rates of Pneumococcal vaccines in Pediatric IBD patients

Study	Age group	Number of patients	Types of vaccine	Definition of seroresponse	Weeks post vaccination	Seroresponse rates				
						All IBD	IM	Anti-TNF	Combination therapy	Controls
Banaszkiewicz 2015	Median 15	122	PCV13	Post-immunization	6-8	90.4%	No subgroup	No subgroup	No subgroup data	96.5% Healthy

(Poland)	(5-18)			antibody ≥ 3.5 ug/mL for ≥ all 13 serotypes			data reported 67% on IM	data reported 9.8% on anti-TNF	reported	controls
Fallahi 2014 (Iran)	Mean 10.7 +/- 4.2 yrs	18	PPSV23	Overall antibody titers =/> lower limit of 2 tailed 90% probability interval of post-immunization IgG of healthy adults	28 days	**44.5%**	No subgroup data 83% on IM	-	-	-

IM – immunomodulator
IS - immunosuppressants
BOLD – significant reduction in serological response compared to controls (red shading)

References:

1. Banaszkiewicz A, Targońska B, Kowalska-Duplaga K, Karolewska-Bochenek K, Sieczkowska A, Gawrońska A, Grzybowska-Chlebowczyk U, Krzesiek E, Łazowska-Przeorek I, Kotowska M, Sienkiewicz E, Walkowiak J, Gregorek H, Radzikowski A, Albrecht P. Immunogenicity of 13-Valent Pneumococcal Conjugate Vaccine in Pediatric Patients with Inflammatory Bowel Disease. Inflamm Bowel Dis. 2015 Jul;21(7):1607-14.
2. Fallahi G, Aghamohammadi A, Khodadad A, Hashemi M, Mohammadinejad P, Asgarian-Omran H, Najafi M, Farhmand F, Motamed F, Soleimani K, Soheili H, Parvaneh N, Darabi B, Nasiri Kalmarzi R, Pourhamdi S, Abolhassani H, Mirminachi B, Rezaei N. Evaluation of antibody response to polysaccharide vaccine and switched memory B cells in pediatric patients with inflammatory bowel disease. Gut Liver. 2014 Jan;8(1):24-8.
3. Lucero MG, Dulalia VE, Nillos LT, Williams G, Parreño RA, Nohynek H, Riley ID, Makela H. Pneumococcal conjugate vaccines for preventing vaccine-type invasive pneumococcal disease and X-ray defined pneumonia in children less than two years of age. Cochrane Database Syst Rev. 2009 Oct 7;(4):CD004977.
4. Centers for Disease Control and Prevention. https://www.cdc.gov/vaccines/acip/recs/grade/table-refs.html
5. Fortanier AC, Venekamp RP, Boonacker CW, Hak E, Schilder AG, Sanders EA, Damoiseaux RA. Pneumococcal conjugate vaccines for preventing acute otitis media in children. Cochrane Database Syst Rev. 2019 May 28;5:CD001480.

Evidence to Decision Table - Pediatric

PICO 15	In pediatric patients with IBD, should vaccination vs. no vaccination against pneumococcal disease be given?
**Population**	Pediatric patients with IBD
**Intervention**	Vaccination against pneumococcal disease
**Comparator**	No vaccination against pneumococcal disease
**Outcome**	Mortality, VPI (pneumococcal infection), SAEs, Immunogenicity

	Judgement	Research evidence	Additional considerations
**Desirable Effects**	**How substantial are the desirable anticipated effects?**<br><br>○ Trivial<br>○ Small<br>○ Moderate<br>○ Large<br><br>○ Varies<br>○ Don't know	**Risk of Pneumococcal infection in pediatric IBD patients**<br><br>One observational study included both adult and pediatric IBD patients found an increased risk of IPD among IBD patients compared to non-IBD controls.[1] No subgroup data was provided for pediatric patients. Compared to non-IBD controls, the risk of IPD is about 1.5- to 2-fold higher in IBD patients. **There is low certainty evidence that pediatric IBD patients have an increased risk of pneumococcal infection compared to non-IBD patients.**<br><br>**Effectiveness and safety of Pneumococcal vaccines in pediatric IBD patients**<br><br>There was no RCT comparing pneumococcal vaccine with placebo or no treatment in pediatric patients with IBD to address this PICO question.<br><br>There are two observational studies that addressed this PICO question.[2,3] One cohort study assessed the immunogenicity of PCV13 in 122 IBD patients with IBD aged 5 to 18 years.[2] No significant difference in the rate of adequate vaccine response to PCV13 was found between IBD patients (majority on immunosuppressive medications) and healthy controls.[2] However, children with IBD on anti-TNF or immunomodulators had lower geometric mean titer rises than children with IBD on no immunosuppressive therapy.[2] A small cohort study assessed the immunogenicity of PPSV23 in 18 pediatric patients with IBD.[3] 55.5% of IBD patients were found to be hypo-responsive to PPSV23.[3] It is important to note that consensus on the correlates of clinical protection is lacking. It is therefore uncertain if this reduced immunologic response is clinically relevant/important and would still afford clinical protection. The evidence suggests that PCV13 can induce a serological response in a significant proportion of pediatric IBD patients. However, vaccination response to PPSV23 may be impaired in pediatric IBD patients. Furthermore, immunosuppressive medications may reduce the immunologic response to pneumococcal vaccination in pediatric IBD patients. No serious adverse events were reported with vaccination. The GRADE rating started at low due to the observational designs of the studies. The rating was downgraded to very low due to	
**Undesirable Effects**	**How substantial are the undesirable anticipated effects?**<br><br>○ Large<br>○ Moderate<br>○ Small<br>○ Trivial<br><br>○ Varies<br>○ Don't know		

		study limitations (selection bias, residual confounding), indirectness (surrogate outcome), and imprecision.<br><br>A Cochrane systematic review of 6 RCTs conducted in healthy children less than 2 years of age found pneumococcal vaccines (PCV 7, 9, 11) to be effective in preventing IPD (RR 0.20, 95% CI 0.10-0.42), X-ray defined pneumonia (RR 0.81, 95% CI 0.66-1.00), and clinical pneumonia (RR 0.94, 0.91-0.98).[4] **The certainty of evidence for effectiveness of pneumococcal vaccines in healthy children less than 2 years old was high, but was downgraded to moderate for IBD patients less than 2 years old and low for IBD patients older than 2 years old.**<br><br>CDC evaluated the evidence of pneumococcal vaccines for immunocompromised children aged 6 through 18 years. Due to the limited body of evidence on vaccine efficacy and safety among persons with immunocompromising conditions, PCV13 vaccine was evaluated using data for HIV-infected children (1 RCT of PCV9 among HIV infected children in South Africa, 1 RCT of PCV7 among HIV-infected adults in Malawi, observational studies of PCV7 in children with sickle cell disease, and immunogenicity studies of PPSV23).[5] Overall, the quality of evidence was rated as **low for PCV13 among immunocompromised children**. However, the desirable consequences were deemed to clearly outweigh undesirable consequences given the very high burden of pneumococcal diseases among immunocompromised children. There was uncertainty regarding costs/benefits of PCV13 vs. PPSV23 in these patients. However, the CDC concluded that broader serotype protection can be achieved through the use of both PCV13 and PPSV23 among immunocompromised children; 49% of IPD in this group is caused by PCV13 serotypes, and an additional 23% by serotypes in PPSV23 not included in PCV13. Evidence from immunogenicity studies demonstrate that antibody response is non-inferior or superior when PCV13 is given before PPSV23 compared to PPSV23 before PCV13. CDC recommends the use of both vaccines among immunocompromised children. This evidence was not included in the evidence profile table as patients with HIV or sickle cell disease were determined *a priori* to be significantly different than IBD patients.<br><br>In terms of safety, a Cochrane systematic review found serious adverse events causally related to vaccination (PCV7, PCV 10/11, PCV7/9, PCV6 + TIV) to be rare in children up to 12 years old, and did not differ significantly between groups.[6] **The certainty of evidence for safety of pneumococcal vaccines was high, but was downgraded to moderate for pediatric IBD patients due to indirectness (sample sizes in the IBD studies were insufficient to detect rare adverse events).**<br><br>**Overall, there is moderate and low certainty evidence that pneumococcal vaccines are safe and effective in pediatric IBD patients age less than 2 (PCV13) and older than 2 (PCV13 and PPSV23), respectively.**	

**Certainty of evidence**	**What is the overall certainty of the evidence of effects?**<br><br>o Very low<br>o Low for IBD patients age > 2 for both PCV 13 and PPSV23<br>o Moderate for IBD patients age < 2 for PCV13<br>o High<br><br>o No included studies		
**Values and Preferences**	**Is there important uncertainty about or variability in how much people value the main outcomes?**<br><br>o Important uncertainty or variability<br>o Possibly important uncertainty or variability<br>o Probably no important uncertainty or variability<br>o No important uncertainty or variability	Patients likely value patient-important outcomes (mortality, VPI, adverse effects) more than surrogate outcomes (immunogenicity).	
**Balance of effects**	**Does the balance between desirable and undesirable effects favor the intervention or the comparison?**<br><br>o Favors the comparison<br>o Probably favors the comparison<br>o Does not favor either the intervention or the comparison<br>o Probably favors the intervention<br>o Favors the intervention<br><br>o Varies<br>o Don't know		
**Resources required**	**How large are the resource requirements (costs)?**<br><br>o Large costs<br>o Moderate costs<br>o Negligible costs and savings<br>o Moderate savings<br>o Large savings<br><br>o Varies<br>o Don't know	CDC vaccine price list last reviewed/updated: July 1, 2019 (see table below)	

Brandname	CDC cost/dose	Private sector cost/dose
Prevnar 13 TM	$137.01	$188.26
Pneumovax®23	$56.30	$105.194

**Certainty of Evidence of Required Resources**	**What is the certainty of the evidence of resource requirements (costs)?**<br><br>○ Very low<br>○ Low<br>○ Moderate<br>○ High<br><br>○ No included studies	The costs of delivering routine immunization services may vary widely across countries and different health system settings. See Immunization Costing Action Network (ICAN) Immunization Delivery Cost Catalogue. http://immunizationeconomics.org/ican-idcc.	
**Cost effectiveness**	**Does the cost-effectiveness of the intervention favor the intervention or the comparison?**<br><br>○ Favors the comparison<br>○ Probably favors the comparison<br>○ Does not favor either the intervention or the comparison<br>○ Probably favors the intervention<br>○ Favors the intervention<br><br>○ Varies<br>○ No included studies	There are no studies that addressed this question specifically in the IBD population.<br><br>A global modelling cost-effectiveness analysis of pneumococcal conjugate vaccination found that PCV13 use was probably cost-effective in all six UN regions.[7] The ICER for PCV introduction is less than GDP per capita in almost all regions and countries[7]. The GDP per capita threshold has been traditionally used as an indication of cost-effectiveness. The analysis showed large benefits of PCV use worldwide in terms of lives saved and disability averted, and in terms of cost-effectiveness, particularly in Africa and Asia.[7]	
**Acceptability**	**Is the intervention acceptable to key stakeholders?**<br><br>○ No<br>○ Probably no<br>○ Probably yes<br>○ Yes<br><br>○ Varies<br>○ Don't know	In a study that assessed parents' and other adults' values for preventing disease associated with pneumococcal infection, both parents and community members assigned relatively high values to preventing meningitis, pneumonia, and complex otitis media.[8] When the value of preventing pneumococcal disease is incorporated into economic analyses, pneumococcal conjugate vaccine has a cost-effectiveness ratio in the range of other widely used health interventions (< 10,000 dollars per QALY at a vaccine cost of 58 dollars per dose).[8]	
**Feasibility**	**Is the intervention feasible to implement?**<br><br>○ No<br>○ Probably no<br>○ Probably yes<br>○ Yes<br><br>○ Varies		

	○ Don't know		

References:

1. Kantsø B, Simonsen J, Hoffmann S, Valentiner-Branth P, Petersen AM, Jess T. Inflammatory Bowel Disease Patients Are at Increased Risk of Invasive Pneumococcal Disease: A Nationwide Danish Cohort Study 1977-2013. Am J Gastroenterol. 2015 Nov;110(11):1582-7.
2. Banaszkiewicz A, Targońska B, Kowalska-Duplaga K, Karolewska-Bochenek K, Sieczkowska A, Gawrońska A, Grzybowska-Chlebowczyk U, Krzesiek E, Łazowska-Przeorek I, Kotowska M, Sienkiewicz E, Walkowiak J, Gregorek H, Radzikowski A, Albrecht P. Immunogenicity of 13-Valent Pneumococcal Conjugate Vaccine in Pediatric Patients with Inflammatory Bowel Disease. Inflamm Bowel Dis. 2015 Jul;21(7):1607-14.
3. Fallahi G, Aghamohammadi A, Khodadad A, Hashemi M, Mohammadinejad P, Asgarian-Omran H, Najafi M, Farhmand F, Motamed F, Soleimani K, Soheili H, Parvaneh N, Darabi B, Nasiri Kalmarzi R, Pourhamdi S, Abolhassani H, Mirminachi B, Rezaei N. Evaluation of antibody response to polysaccharide vaccine and switched memory B cells in pediatric patients with inflammatory bowel disease. Gut Liver. 2014 Jan;8(1):24-8.
4. Lucero MG, Dulalia VE, Nillos LT, Williams G, Parreño RA, Nohynek H, Riley ID, Makela H. Pneumococcal conjugate vaccines for preventing vaccine-type invasive pneumococcal disease and X-ray defined pneumonia in children less than two years of age. Cochrane Database Syst Rev. 2009 Oct 7;(4):CD004977.
5. Centers for Disease Control and Prevention. https://www.cdc.gov/vaccines/acip/recs/grade/table-refs.html
6. Fortanier AC, Venekamp RP, Boonacker CW, Hak E, Schilder AG, Sanders EA, Damoiseaux RA. Pneumococcal conjugate vaccines for preventing acute otitis media in children. Cochrane Database Syst Rev. 2019 May 28;5:CD001480.
7. Chen C, Cervero Liceras F, Flasche S, Sidharta S, Yoong J, Sundaram N, Jit M. Effect and cost-effectiveness of pneumococcal conjugate vaccination: a global modelling analysis. Lancet Glob Health. 2019 Jan;7(1):e58-e67.
8. Prosser LA, Ray GT, O'Brien M, Kleinman K, Santoli J, Lieu TA. Preferences and willingness to pay for health states prevented by pneumococcal conjugate vaccine. Pediatrics. 2004 Feb;113(2):283-90.

Conclusion – Pediatric

PICO 15: In pediatric patients with IBD, should age appropriate pneumococcal vaccines be given?

Moderate certainty of evidence (age $\leq$ 2)

Low certainty of evidence (age > 2)

Direction – Yes (100%)

Strength – strong (100%)

Type of recommendation	Strong recommendation against the intervention	Conditional recommendation against the intervention	Conditional recommendation for either the intervention or the comparison	Conditional recommendation for the intervention	Strong recommendation for the intervention
	○	○	○	○	○

**Recommendation**	**Statement 15: In pediatric patients with IBD, we recommend age-appropriate pneumococcal vaccines be given.**
**Justification**	
**Subgroup considerations**	
**Implementation considerations**	
**Monitoring and evaluation**	• Ongoing monitoring of serious adverse events associated with pneumococcal vaccine in IBD patients
**Research priorities**	• Observational or RCTs to determine the clinical effectiveness of pneumococcal vaccine in IBD patients with assessment of patient-important outcomes (i.e. pneumococcal infection etc.)<br>• Observational studies to establish correlates of seroprotection against pneumococcal disease in IBD patients<br>• More RCTs are needed to compare single vs. booster vaccination strategies for pneumococcal vaccine (PPSV23) in pediatric IBD patients on immunosuppressive medications

Summary – Adults

**PICO 16A**	**In adult patients with IBD (not on immunosuppressive therapy), with a risk factor for pneumococcal disease, should vaccination vs. no vaccination against pneumococcal disease be given?**
**Population**	Adult patients with IBD (not on immunosuppressive therapy), with a risk factor for pneumococcal disease
**Intervention**	Vaccination against pneumococcal disease
**Comparator**	No vaccination against pneumococcal disease
**Outcome**	Mortality, VPI (pneumococcal infection), SAEs, Immunogenicity

**PICO**	**In adult patients with IBD (not on immunosuppressive therapy), without a risk factor for pneumococcal disease, should vaccination vs. no vaccination against pneumococcal disease be given?**
**Population**	Adult patients with IBD (not on immunosuppressive therapy), without a risk factor for pneumococcal disease
**Intervention**	Vaccination against pneumococcal disease
**Comparator**	No vaccination against pneumococcal disease
**Outcome**	Mortality, VPI (pneumococcal infection), SAEs, Immunogenicity

**PICO 16B**	**In adult patients with IBD (on immunosuppressive therapy), should vaccination vs. no vaccination against pneumococcal disease be given?**
**Population**	Adult patients with IBD (on immunosuppressive therapy)
**Intervention**	Vaccination against pneumococcal disease
**Comparator**	No vaccination against pneumococcal disease

Outcome	Mortality, VPI (pneumococcal infection), SAEs, Immunogenicity

There was no RCT comparing pneumococcal vaccine with placebo or no treatment in adult patients with IBD to address this PICO question.

There are six observational studies that addressed this PICO question.[1-6] In one cross-sectional observational study utilizing administrative data extracted from the Veterans Health Administration in the US, one-year mortality was lower for those vaccinated against pneumococcal infection relative to the unvaccinated (2.1% vs. 4.5%, $P < 0.001$).[1] However, only 20% of the cohort received pneumococcal vaccination.[1] Five observational studies assessed serological response to pneumococcal vaccination in IBD patients on immunosuppressive medications compared to healthy controls or IBD patients not on immunosuppressive medications.[2-6] One study assessed the immunogenicity of sequential vaccination schedule of PCV13 followed by PPSV23.[2] The other four studies assessed the immunogenicity of PPSV23.[3-6] It is important to note that consensus on the correlates of clinical protection is lacking. The cut-off value of serotype-specific IgG antibody titers $\geq$ 0.35ug/mL was recommended by the WHO as protective for invasive pneumococcal disease (IPD). This cut-off is based on 3 clinical studies in children, who received PCV7. However, this cut-off is not serotype-specific. Comparisons across studies are difficult as studies compared different serotypes with different definitions of vaccination response. Some studies performed serotype specific assays of the individual antibody responses to the serotypes included in the vaccine, whereas others only provided overall response rates. The evidence suggest that pneumococcal vaccination can induce a serological response in a significant proportion of adult IBD patients. Immunosuppressive medications (e.g. immunomodulators, anti-TNF, combination therapy) may reduce the immunologic response to pneumococcal vaccination in IBD patients, particularly when combination immunosuppressive medications are used. However, it is uncertain if this reduced immunologic response is clinically relevant/important and would still afford clinical protection. No serious adverse events including disease exacerbation was reported.

Interestingly, one study suggested that patients on immunosuppressive medications had a lower seroconversion rates to serotypes present in both PCV13 and PPSV23 (50%) compared with the seroconversion to the serotypes exclusive to PPSV23 (70%).[2] The absence of a PPSV23 booster effect for PCV13 serotypes in immunocompromised patients has been described previously in recipients of allogenic hematopoietic stem cell transplant patients.[7] Multiple priming doses of PCV13 may be necessary.

The GRADE rating started at low due to the observational designs of these studies. The rating was downgraded to **very low** due to study limitations (selection bias, residual confounding, misclassification errors), indirectness (surrogate outcome), and imprecision. In particular, IBD patients who agreed to vaccination were likely to be systematically different than those who did not agree or seek vaccination (healthy vaccinee effect or confounding by indication). This may lead to selection bias confounding the vaccine's effect

on the outcomes (e.g. mortality, infection, adverse events, and even immunologic response). It is important to note that most patients included in the studies were age < 65. Very few elderly patients (age > 65) were included. Therefore, the evidence in elderly IBD patients was even more uncertain.

A Cochrane systematic review of 18 RCTs found pneumococcal polysaccharide vaccines to be effective in reducing IPD (OR 0.26, 95% CI 0.14-0.45) and all-cause pneumonia (OR 0.72, 95% CI 0.56-0.93) in adults.[8] In subgroup analyses, there was evidence of protective efficacy against IPD in healthy adults in low-income countries (OR 0.14, 95% CI 0.03-0.61), healthy adults in high-income countries (OR 0.20, 95% CI 0.10-0.39), but not in adults with chronic disease in high-income countries with wide confidence interval (OR 1.56, 95% CI 0.35-6.94).[8] **Results of RCTs are consistent with a protective effect against IPD and all-cause pneumonia among generally healthy adults. Such trials have not demonstrated that PPSV23 is efficacious against either IPD or all-cause pneumonia in populations at higher risk (due to imprecision), such as adults and children with underlying conditions that increase their risk of pneumococcal disease or highly immunosuppressed individuals of any age.[8]** The evidence for effectiveness was moderate due to study limitations. **The evidence for effectiveness was anchored to the general population (moderate for IBD patients not on immunosuppressive medications), but was downgraded to <u>low</u> due to indirectness as the vaccine may be less immunogenic in IBD patients on immunosuppressive medications than in the general population**.

CDC evaluated the evidence of pneumococcal vaccines for immunocompromised adults. Due to the limited body of evidence on vaccine efficacy and safety among persons with immunocompromising conditions, both PCV13 and PPSV23 vaccines were evaluated using data for HIV-infected adults (1 RCT of PCV7 among HIV infected adults in Malawi, 1 RCT of PPSV23 among HIV-infected adults in Uganda as well as observational studies in US and Europe).[9] Overall, the quality of evidence was rated as **very low for PPSV23 and low for PCV13 among immunocompromised adults**. However, the desirable consequences were deemed to clearly outweigh undesirable consequences given the extremely high burden of pneumococcal diseases among immunocompromised adults. Therefore, the CDC recommends the use of both vaccines among immunocompromised adults.

In terms of safety, the Cochrane systematic review did not assess adverse events related to the use of pneumococcal vaccines.[8] In 6 RCTs (from CDC profile) on PCV13, no serious adverse events were identified.[10] The evidence was downgraded to moderate due to indirectness. In a systematic review of 18 studies (RCTs and observational studies) of anti-pneumococcal vaccine in 601 systemic lupus erythematosus (SLE) patients, no serious adverse events were reported.[11] The evidence was downgraded to low due to imprecision and indirectness (not IBD patients).[11] In 4 observational studies, no serious adverse events were reported after administration of pneumococcal vaccine in IBD patients.[2-5] **The certainty of evidence for safety of pneumococcal vaccines in adult IBD patients was <u>moderate.</u>**

Overall, there is <u>moderate</u> certainty evidence that pneumococcal vaccines are safe and effective in adult IBD patients <u>not on immunosuppressive medications</u>. For IBD patients <u>on immunosuppressive medications</u>, the overall certainty of evidence was <u>low</u>.

Risk of Bias Table – Adults

SR of RCTs		
**Study**	**Quality Assessment**	**Comments**
Morberley 2013 **(Healthy Adults in low income countries / high income countries, adults with chronic illnesses in high-income countries)**	• Most RCTs scored poorly across domains and only 4 trials were overall low risk of bias. The poor scores were common in the earlier trials and were largely due to inadequate reporting rather than known inadequate methods. RCTs contributing data were conducted over a long period of time (1947-2000) and within distinct population groups, utilizing various valencies of the vaccine with differing amounts of antigen content.	• Cochrane SR of 18 RCTs (64,852 adults) assessing the effectiveness of pneumococcal vaccines (any type including 2, 6, 14, 23-valent) in healthy adults and adults with chronic illnesses such as COPD or bronchogenic carcinoma<br>• **Overall, pneumococcal polysaccharide vaccine reduced the risk of all IPD (OR 0.26, 95% CI 0.14-0.45, I^2 = 0%) and all-cause pneumonia (OR 0.72, 95% CI 0.56-0.93, I^2 = 85%), but not mortality (OR 0.90, 95% CI 0.74-1.09, I^2 = 69%)**<br>• Healthy adults in low income countries (5373 participants): OR 0.14 (95% CI 0.03-0.61)<br>• Healthy adults in high income countries (27,886 participants): OR 0.20 (95% CI 0.10-0.39), I^2 = 0%<br>• Adults with chronic disease in high-income countries (3230 participants): OR 1.56 (0.35-6.94), I^2 = 0%<br>• **Results of RCTs are consistent with a protective effect against IPD and all-cause pneumonia among generally healthy adults. Such trials have not demonstrated that PPSV23 is efficacious against either IPD or all-cause pneumonia in populations at higher risk, such as adults and children with underlying conditions that increase their risk of pneumococcal disease or highly immunosuppressed individuals of an age.**<br>• Observational studies of PPSV23 generally have found the vaccine is 50-80% effective in preventing IPD among immunocompetent adults and individuals with various underlying illnesses who are not severely immunosuppressed.<br>• No report of AEs

Nguyen 2015<br>**(IBD patients)**	• Inappropriate to pool these studies together in a MA given the heterogeneity in study designs, control groups used (HC vs. IBD patients not on IS), interventions (different vaccines), and different definitions of outcomes (seroresponse or seroprotection for the same vaccine and also among different vaccines)<br>• Data extraction errors -> included a hepatitis B vaccine study under pneumococcal vaccine<br>• Used Effective Public Health Practice Project model" to assess quality and rated all studies as "strong"	• SR of 9 cohort studies (n = 1474) comparing IBD patients on IS (anti-TNF, IM, and/or prednisone $\geq$ 20mg/day) vs IBD patients not on IS or HC<br>• Different vaccines were included: Hep B (2), Hep A (1), Influenza (2), Pneumococcal (4)<br>• Included 4 studies that assessed pneumococcal vaccines (Fiorino 2012, Lee 2014 , Melmed 2010), but 1 (Gisbert 2012) was erroneously included as it assessed hepatitis B vaccine (not pneumococcal vaccine)<br>• IBD patients on IS have a significantly lower response to routine vaccinations. The great effect is seen among those on anti-TNF and combination IS.
Agarwal 2012<br>**(Immune-mediated diseases including RA, IBD on Immunosuppressants)**	• No risk of bias assessment	• SR of response to routine vaccines (immunogenicity) in patients with immune-mediated diseases on immunosuppressives<br>• 4 studies of PPSV23 administered to patients with rheumatic diseases showed that vaccine responses were reduced with Methotrexate alone or in combination with anti-TNF, but anti-TNF alone may not diminish the vaccine response.<br>• **1 study in IBD patients (Melmed 2010) showed reduced vaccine response in patients on combination treatment (anti-TNF + IM)**

HC: healthy controls
IS: immunosuppressive medications

**Before-After (Pre-Post) Studies**									
**Study**	**Was there a <u>concurrent</u> comparator group that did not receive the intervention**	**If a concurrent comparator group was used, was it <u>similar</u> to the intervention group (or adequately adjusted) for prognostic factors**	**If <u>no</u> concurrent comparator group was used**		**Outcome detection methods valid and similar among compared groups / periods**	**Incomplete outcome data assessed**	**Selective outcome reporting**	**Other bias**	**Comments**
			**If each participant served as his/her own control (assessed before vs. after the intervention), are there compelling**	**If two different consecutive cohorts of participants were assessed (before vs. after implementation of the intervention), are there (a) compelling**					

			**arguments that the outcome was not influenced by historic events / underlying secular trends**	**arguments that the outcome was not influenced by historic events / underlying secular trends and (b) evidence that the two groups were similar (or adequately adjusted) for prognostic factors**					
Van Aalst 2019 (Netherlands)	No	Adjusted for sex, age, disease type, treatment subgroup, smoking, alcohol, body mass index, use of low-dose prednisolone and topical steroids.<br><br>Only use of IS was significantly associated with seroconversion (OR 0.32, 0.10-0.98)	OK	NA	OK	OK	OK	Patients who consented to receive vaccination were likely to be prognostically different than patients who did not agree to participate (healthy vaccinee effect)	• Prospective cohort study of 141 IBD patients (age ≥ 18) on IM (35), anti-TNF (40), combination therapy (29), no IS (37)<br>• PCV 13 then PPSV 23 2 mos later<br>• Seroconversion at 4-8 weeks post-vaccination<br>• Seroconversion defined as post-immunization antibody of ≥ 1.3ug/mL for ≥ 70% of all measured serotypes<br>• **Seroconversion**

									**rates for all 23 serotypes among patients using IS 59% vs. 81% controls (OR 0.33, 95% CI 0.13-0.82)**<br>• **No vaccine related serious AEs**
Lee 2019 (Korea)	No	Adjusted for age, gender, type of therapy, baseline disease activity, disease duration, duration of IS therapy	OK	NA	OK	OK	OK	Possible selection bias as uncertain how patients were selected into the study.	• Multi-center, prospective observational study of 197 adult CD patients<br>• All received PPSV23<br>• Seroresponse defined as % subjects achieving a 2-fold increase in overall IgG antibody titer<br>• **Overall serological response 67%**<br>• **Serological response significantly lower in patients on anti-TNF (50%), anti-TNF + IM (58%) than patients on 5ASA (78.4%). IM did not affect the immunologic response to vaccine (78.6%)**<br>• Subset of patients

									on anti-TNF, no significant difference in serological response rate regarding timing of vaccination relative to IFX infusion cycle.<br>• **No serious AEs**
Dotan 2012 (US and Israel)	No	No	OK	NA	OK	19% (10/53) either withdrew due to thiopurine side effects or were lost to follow-up<br><br>Reported outcomes only on 53% (28/53) who were started on thiopurine. Unclear if the other patients received vaccine or not	OK	Patients who consented to receive vaccination were likely to be prognostically different than patients who did not agree to participate (healthy vaccinee effect)	• Prospective cohort study of 53 IBD patients (35 CD, 15 UC, 3 IC) who were starting on thiopurine treatment<br>• Patients were administered PPSV23 vaccine before thiopurine therapy<br>• Post-therapy average 6-MP dose: 1.05 +/- 0.30mg/kg<br>• Response to vaccine was defined as $\geq$ 2-fold increase in antibody titer to at least 4/14 serotypes within the vaccine.<br>• **75% patients had seroresponse to PPSV23**

									• **No vaccine induced disease exacerbation**
Fiorino 2012 (Italy)	No	Adjusted for age, sex, baseline antibody titer, disease activity, time interval between baseline and post-vaccination assessment, and form of therapy.	OK	NA	OK	OK	OK	Patients who consented to receive vaccination were likely to be prognostically different than patients who did not agree to participate (healthy vaccinee effect)	• Prospective study of 96 consecutive patients with IBD (35 on 5ASA, 19 on thiopurine, 26 IFX, 16 combined IS)<br>• All received PPSV23 vaccine<br>• Seroresponse was defined as at least a 2 fold increase in anti-pneumococcal antibodies at least 3 weeks after vaccination<br>• **Patients on IFX or combination IS had significantly lower response rates (57.6% and 62.5%) vs. 5ASA (88.6%)**<br>• AZA alone did not influence the response rate (78.9%)<br>• **No serious AEs**
Melmed 2010 (US)	No	Adjusted for age, gender, disease duration, baseline CRP, disease activity	OK	NA	OK	OK	OK	Patients who consented to receive vaccination were likely to be prognostically	• Cohort study of 45 adult IBD patients (20 combination treatment, 25 5ASA only, 19 healthy controls)<br>• All received

		Significant differences in disease types between groups with 85% of patients on combination treatment had CD vs 52% of patients on 5ASA had CD. When disease type was added as a covariate to the regression model, the odds of response was not significantly different between the 2 groups of IBD patients.						different than patients who did not agree to participate (healthy vaccinee effect)	PPSV23<br>• Seroresponse defined as two fold or greater increase in antibody titer and ≥ 1 ug post vaccination GMT in the majority of antibodies (3 or more out of 5 serotypes tested) at 4 weeks<br>• **Overall vaccine response was lower in IBD patients on combination treatment than those not on IS or healthy controls (45%, 80%, 85%, P = 0.01)**

AEs: adverse events
CD: Crohn's disease
IFX: infliximab
IM: immunomodulator therapy
IS: immunosuppressive therapy

Cohort studies							
**Study**	**Valid methods to ascertain exposure**	**Prognostic factors (other than exposure of interest) similar among**	**Demonstration that outcome of interest was**	**Outcome detection methods**	**Follow-up complete and similar**	**Free of other bias**	**Comments**

		cohorts – or cohorts were adjusted adequately for confounders	not present at the start of the study	valid and similar among cohorts	among cohorts		
Case 2015 (US)	Data were reliant on administrative codes (pneumococcal vaccination). Possible misclassification errors due to errors of miscoding, and the codes have not been previously validated.<br><br>Receipt of vaccine outside the VA was not accounted for.	Adjusted for demographic covariates (age, sex, marital status), VHA priority (disabled) status, region of treatment, immunosuppressive medication, and comorbidity burden.<br><br>Did not adjust for disease activity or severity.	OK	OK - mortality	OK	Selection bias: vaccine may be selectively given to healthier patients. Healthy vaccinee effect. This may have led to over-estimation of the protective effect of the vaccine.	• Cross sectional observational study of 49,350 IBD patients in the VHA system from 2005-2009 (mean age 62, range age 19-98; 94% male), 10% on IS in any year<br>• 20% received pneumococcal vaccination (5% prior to IBD diagnosis, 2% on the date of IBD diagnosis, 13% after IBD diagnosis)<br>• **1-year mortality was lower for those vaccinated vs. unvaccinated (2.1% vs. 4.5%, P < 0.001)**<br>• 1-year mortality for patients with IBD vaccinated before diagnosis (OR 0.71, 95% CI 0.58-0.86), vaccinated at diagnosis (OR 0.54, 95% CI 0.36-0.82), after diagnosis (OR 0.14, 95% CI 0.10-.0.19)<br>• Being married, living outside of the Northeast, and having more comorbidities were associated with vaccination before IBD diagnosis<br>• Models of vaccination at or after diagnosis poor fit: little better than chance.

IS: immunosuppressive medication
VA: Veterans Health Administration

Evidence Profile Table - Adults

**Certainty Assessment**								**Summary of Findings**	
Studies	Risk of bias	Inconsistency	Indirectness	Imprecision	Other considerations	Certainty of Evidence	Overall Certainty of evidence	Study Event Rates	Relative Effect (95% CI)
**Mortality - CRITICAL**									
1 SR 18 RCTs[8]<br><br>**General population**	Serious[a]	Not serious[b]	Not serious<br>For not on IS<br><br>Serious[c]<br>For on IS	Not serious	None	⊕⊕⊕⊖<br>MODERATE<br>For not on IS<br><br>⊕⊕⊖⊖<br>LOW<br>For on IS	⊕⊕⊕⊖<br>MODERATE<br>For not on IS<br><br>⊕⊕⊖⊖<br>LOW<br>For on IS	• No reduction in mortality with pneumococcal polysaccharide vaccine (OR 0.90, 95% CI 0.74-1.09)	
1 Observational study[1]<br><br>**IBD populations**	Serious[d]	Not serious	Serious[e]	Not serious	None	⊕⊖⊖⊖<br>VERY LOW		• **Mortality was lower for those vaccinated** vs. unvaccinated (2.1% vs. 4.5%, P < 0.001)<br>• 1-year mortality for patients with IBD vaccinated before diagnosis (OR 0.71, 95% CI 0.58-0.86), vaccinated at diagnosis (OR 0.54, 95% CI 0.36-0.82), after diagnosis (OR 0.14, 95% CI 0.10-0.19)	
**VPI (IPD) - CRITICAL**									
1 SR 18 RCTs[8]<br><br>**General population**	Serious[a]	Not serious[b]	Not serious<br>For not on IS<br><br>Serious[c]<br>For on IS	Not serious	None	⊕⊕⊕⊖<br>MODERATE<br>For not on IS<br><br>⊕⊕⊖⊖<br>LOW<br>For on IS		• **Reduced risk of IPD with pneumococcal vaccine (OR 0.26, 95% CI 0.14-0.45)**<br>• **3 fewer per 1000 (from 2 fewer to 3 fewer)**	
**Immunogenicity (Seroresponse as defined by primary studies) - IMPORTANT**									
5 Observational studies[2-6]<br><br>**IBD populations**	Serious[f]	Not serious	Serious[g]	Serious[h]	None	⊕⊖⊖⊖<br>VERY LOW		• See **Summary of observational studies assessing the seroresponse rates of pneumococcal vaccines in adult IBD patients**<br>• Seroresponse ranged from 41-89%.<br>• Seroresponse appeared to be reduced in patients on immunosuppressive medications	

Adverse events - CRITICAL								
6 RCTs[10]<br><br>**General population**<br>(from CDC GRADE profile)	Not serious	Not serious	Serious[i]	Not serious	None	⊕⊕⊕⊖<br>MODERATE		• No serious adverse events
1 SR 18 studies (RCTs and observational studies)[11]<br><br>**SLE patients**	Not serious	Not serious	Serious[j]	Serious[k]	None	⊕⊕⊖⊖<br>LOW		• No serious adverse events<br>• Up to one third of cases reported mild/low-grade complaints
4 Observational studies[2-5]<br><br>**IBD populations**	Serious[f]	Not serious	Not serious	Serious[h]	None	⊕⊖⊖⊖<br>VERY LOW		• No serious adverse events or vaccine induced disease exacerbation

Footnotes:

a. Downgraded for study limitations. 39% of trials described an adequate method of sequence generation and allocation concealment. 44% trials reported adequate blinding of participants and personnel.
b. Not downgraded for statistical inconsistency as this could be explained by differences in efficacy in low-income vs. high-income countries and general population vs. adults with chronic illness.
c. Downgraded for indirectness. Immunogenicity studies suggested that pneumococcal vaccines may be less immunogenic (and therefore less effective) in IBD populations than in the general population.
d. Downgraded for study limitations. Selection bias: vaccine may be selectively given to healthier patients (healthy vaccinee effect). This may have led to over-estimation of the protective effect of the vaccine. Data were reliant on administrative codes (pneumococcal vaccination). Possible misclassification errors due to errors of miscoding, and the codes have not been previously validated. Receipt of vaccine outside the VA was not accounted for. Possible residual confounding factors: did not adjust for smoking, disease activity or severity.
e. Downgraded for indirectness. Included patients from Veterans Health Administration system (predominantly older male, about 10% of cohort was on immunosuppressive drugs in any year). Results may not be generalizable to all IBD populations.
f. Downgraded for study limitations. Selection bias: vaccine may be selectively given to healthier or sicker patients. This may have led to over- or under- estimation of the protective effect of the vaccine depending on the direction of bias.
g. Downgraded for indirectness. Definitions of seroresponse were highly variable across studies. Surrogate outcome of seroresponse was used to estimate clinical efficacy or effectiveness. However, consensus on the correlates of protection for pneumococcal vaccine is lacking. Studies also included varying proportions of patients on no immunosuppressives or different immunosuppressive medications.
h. Downgraded for imprecision as small sample sizes for each subgroup of patients on different immunosuppressive medications.
i. Downgraded for indirectness. Included general population and not IBD patients. Small sample sizes of IBD studies cannot detect rare adverse effects.
j. Downgraded for indirectness. Included SLE patients and not IBD patients.
k. Downgraded for imprecision as small sample sizes to detect rare adverse events.

Summary of observational studies assessing the seroresponse rates of Pneumococcal vaccines in Adult IBD patients

Study	Age group	Number of patients	Types of vaccine	Definition of seroresponse	Weeks post vaccination	Seroresponse rates			
						IM	Anti-TNF	Combination therapy	Controls
Van Aalst 2019 (Netherlands)	Median 45 (29-56)	141	PCV13	Post-immunization antibody ≥ 1.3ng/mL for ≥ 70% of all 23 measured serotypes	4-8	49% (31-64%) **OR 0.18 (0.06-0.55)**	58% (42-73%) **OR 0.26 (0.09-0.77)**	41% (23-60%) **OR 0.14 (0.04-0.43)**	84% (71-94%) IBD patients not on IS
			PPSV 23			74% (60-88%) OR 0.67 (0.22-2.06)	78% (64-91%) OR 0.80 (0.27-2.43)	55% (37-74%) **OR 0.29 (0.10-0.86)**	81% (67-92%) IBD patients not on IS
			All 23 serotypes			60% (42-75%) OR 0.35 (0.12-1.02)	63% (46-78%) OR 0.39 (0.14-1.10)	52% (33-71%) **OR 0.25 (0.08-0.75)**	81% (68-93%) IBD patients not on IS
Lee 2014 (Korea)	Mean 32.4	197	PPSV 23	2-fold increase in overall IgG anti-pneumococcal antibody titer	4	78.6% *(dose of AZA used was relatively low 75.5mg/day)*	**50.0%**	**58.0%**	78.4% IBD patients on 5ASA
Dotan 2012 (US and Israel)	Mean 34.35	28	PPSV 23	2-fold or greater increase in antibody titer to least 4/14 serotypes	3	75%	-	-	-
Fiorino 2012 (Italy)	Mean 42 (19-70)	96	PPSV 23	2-fold or greater increase in overall antibody titer	At least 3	78.9% OR 0.48 (0.10-0.82)	**57.7% OR 0.17 (0.04-0.64)**	**62.5% OR 0.21 (0.05-0.91)**	88.6% IBD patients on 5ASA
Melmed 2010 (US)	Median 36.5 On	45	PPSV23	2-fold or greater increase in	4	-	-	**45%**	80% IBD patients on 5ASA

	combination<br><br>Median 40<br>On 5ASA only			antibody titer and $\geq$ 1 ug post vaccination GMT in the majority of antibodies (3 or more out of 5 serotypes tested)					84% Healthy controls

AZA - azathioprine
IM – immunomodulator
IS – immunosuppressants
BOLD – significant reduction in serological response compared to controls (red shading)

References:

1. Case DJ, Copeland LA, Stock EM, Herrera HR, Pfanner TP. Pneumococcal vaccination rates in VHA patients with inflammatory bowel disease. Medicine (Baltimore). 2015 Feb;94(6):e417
2. van Aalst M, Garcia Garrido HM, van der Leun J, Meek B, van Leeuwen EMM, Löwenberg M, D'Haens GR, Ponsioen CYI, Grobusch MP, Goorhuis A. Immunogenicity of the currently recommended pneumococcal vaccination schedule in patients with inflammatory bowel disease. Clin Infect Dis. 2019 Mar 22.
3. Lee CK, Kim HS, Ye BD, Lee KM, Kim YS, Rhee SY, Kim HJ, Yang SK, Moon W, Koo JS, Lee SH, Seo GS, Park SJ, Choi CH, Jung SA, Hong SN, Im JP, Kim ES; Korean Association for the Study of Intestinal Diseases (KASID) Study. Patients with Crohn's disease on anti-tumor necrosis factor therapy are at significant risk of inadequate response to the 23-valent pneumococcal polysaccharide vaccine. J Crohns Colitis. 2014 May;8(5):384-91.
4. Dotan I, Werner L, Vigodman S, Agarwal S, Pfeffer J, Horowitz N, Malter L, Abreu M, Ullman T, Guzner-Gur H, Halpern Z, Mayer L. Normal response to vaccines in inflammatory bowel disease patients treated with thiopurines. Inflamm Bowel Dis. 2012 Feb;18(2):261-8.
5. Fiorino G, Peyrin-Biroulet L, Naccarato P, Szabò H, Sociale OR, Vetrano S, Fries W, Montanelli A, Repici A, Malesci A, Danese S. Effects of immunosuppression on immune response to pneumococcal vaccine in inflammatory bowel disease: a prospective study. Inflamm Bowel Dis. 2012 Jun;18(6):1042-7.
6. Melmed GY, Agarwal N, Frenck RW, Ippoliti AF, Ibanez P, Papadakis KA, Simpson P, Barolet-Garcia C, Ward J, Targan SR, Vasiliauskas EA. Immunosuppression impairs response to pneumococcal polysaccharide vaccination in patients with inflammatory bowel disease. Am J Gastroenterol. 2010 Jan;105(1):148-54.
7. Cordonnier C, Ljungman P, Juergens C, Maertens J, Selleslag D, Sundaraiyer V, Giardina PC, Clarke K, Gruber WC, Scott DA, Schmoele-Thoma B; 3003 Study Group. Immunogenicity, safety, and tolerability of 13-valent pneumococcal conjugate vaccine followed by 23-valent pneumococcal polysaccharide vaccine in recipients of allogeneic hematopoietic stem cell transplant aged ≥2 years: an open-label study. Clin Infect Dis. 2015 Aug 1;61(3):313-23
8. Moberley S, Holden J, Tatham DP, Andrews RM. Vaccines for preventing pneumococcal infection in adults. Cochrane Database Syst Rev. 2013 Jan 31;(1):CD000422.
9. Centers for Disease Control and Prevention. https://www.cdc.gov/vaccines/acip/recs/grade/table-refs.html

10. Adawi M, Bragazzi NL, McGonagle D, Watad S, Mahroum N, Damiani G, Conic R, Bridgewood C, Mahagna H, Giacomelli L, Eggenhöffner R, Mahamid M, Pigatto PDM, Amital H, Watad A. Immunogenicity, safety and tolerability of anti-pneumococcal vaccination in systemic lupus erythematosus patients: An evidence-informed and PRISMA compliant systematic review and meta-analysis. Autoimmun Rev. 2019 Jan;18(1):73-92.
11. Food and Drug Administration. FDA expands use of Prevnar 13 vaccine for people ages 50 and older. Silver Spring, MD: US Department of Health and Human Services, Food and Drug Administration. 2011. Available at http://www.fda.gov/newsevents/newsroom/pressannoucements/ucm 285431.htm? source=govdelivery. Accessed May 21, 2012.

Evidence to Decision Table – Adults (not on immunosuppressive therapy)

**PICO 16A**	**In adult patients with IBD (not on immunosuppressive therapy), with a risk factor for pneumococcal disease, should vaccination vs. no vaccination against pneumococcal disease be given?**
**Population**	Adult patients with IBD (not on immunosuppressive therapy), with a risk factor for pneumococcal disease
**Intervention**	Vaccination against pneumococcal disease
**Comparator**	No vaccination against pneumococcal disease
**Outcome**	Mortality, VPI (pneumococcal infection), SAEs, Immunogenicity

**PICO**	**In adult patients with IBD (not on immunosuppressive therapy), without a risk factor for pneumococcal disease, should vaccination vs. no vaccination against pneumococcal disease be given?**
**Population**	Adult patients with IBD (not on immunosuppressive therapy), without a risk factor for pneumococcal disease
**Intervention**	Vaccination against pneumococcal disease
**Comparator**	No vaccination against pneumococcal disease
**Outcome**	Mortality, VPI (pneumococcal infection), SAEs, Immunogenicity

	**Judgement**	**Research evidence**	**Additional considerations**
**able Effects**	**How substantial are the desirable anticipated**	**Risk of Pneumococcal infection in adult IBD patients**	Vaccination titers may wane over time in immunosuppressed populations. It is unclear

	**effects?**<br><br>○ Trivial<br>○ Small without RF<br>○ Moderate<br>○ Large with RF<br><br>○ Varies<br>○ Don't know	One systematic review and five observational studies addressed this PICO question.[1-6]<br><br>One systematic review assessed the incidence of invasive pneumococcal disease (IPD) in immunocompromised patients.[1] Five studies included patients with chronic inflammatory disease such as SLE, Sjogren's syndrome, polymyositis/dermatomyositis, COPD, asthma, RA, and IBD. No subgroup data was provided for IBD. Compared to healthy control cohorts (pooled incidence rate: 10/100,000 person-years), the incidence rate of IPD was increased in patients with chronic inflammatory disease (pooled incidence rate: 65/100,000 person-years).[1] One observational study found an increased risk of IPD among patients with autoimmune diseases (RA, SLE, CD) vs. healthy adults.[2] Two other observational studies also found an increased risk of IPD among IBD patients compared to non-IBD controls.[3,4] **Compared to non-IBD controls, the risk of IPD is about 1.5- to 2-fold higher in IBD patients.**<br><br>One cross-sectional case-control study used an administrative database (Nationwide Inpatient Sample) to compare the risks of hospitalization for pneumonia due to Streptococcus pneumoniae among adult IBD patients vs. non-IBD controls.[5] It is important to note that pneumonia treated as outpatients were excluded. After adjusting for various factors including comorbidities, risk factors for pneumonia, as well as patient and hospital characteristics, IBD patients did not demonstrate increased odds of hospitalization for Streptococcus pneumoniae compared to non-IBD controls.[5] Mortality during these admissions among IBD patients was not significantly higher than the control population.[5]<br><br>One retrospective cohort and nested case-control study found an increased risk of pneumonia among IBD patients compared to non-IBD patients after adjusting for age, disease type, health care utilization and comorbidities.6 Use of biologic medications, corticosteroids, and PPI were significantly associated with pneumonia.[6] As the most common etiologic agent of community acquired pneumonia in the US is pneumococcal pneumonia, the evidence was not downgraded for indirectness (outcome).<br><br>The GRADE rating started at high as these were considered prognostic studies (providing evidence about the likelihood of Streptococcal infection in patients with IBD). The rating was further downgraded to **low** due to study limitations (residual confounding factors, detection bias, admission bias, and misclassification bias). In particular, patients with IBD and respiratory symptoms may be more likely to be tested for, diagnosed with, and admitted for Streptococcus pneumoniae infection than non-IBD controls, thus creating an overestimate of the risk of Streptococcal infection among IBD patients**. In summary, there is low certainty evidence that adult IBD patients have an increased risk of pneumococcal infection compared to non-IBD patients.**<br><br>**Effectiveness and safety of Pneumococcal vaccines in adult IBD patients**<br><br>There was no RCT comparing pneumococcal vaccine with placebo or no treatment in adult patients with IBD to address this PICO question.<br><br>There are six observational studies that addressed this PICO question.[7-12] In one cross-	which of the antigens should be assessed, what levels of titers are to be considered protective, and whether and when to revaccinate patients with low titers. There may also be differences in response between serotypes. The clinical significance of decline in detectable antibodies is not clear since immune correlates of protection for pneumococcal polysaccharide vaccine have not been established and relatively limited clinical data are available regarding the duration of vaccine-induced protection against IPD. It is unclear if regular revaccination is needed because the incidence of pneumococcal infection in adults increases with age.
**Undesirable Effects**	**How substantial are the undesirable anticipated effects?**<br><br>○ Large<br>○ Moderate<br>○ Small<br>○ Trivial<br><br>○ Varies<br>○ Don't know		

		sectional observational study utilizing administrative data extracted from the Veterans Health Administration in the US, one-year mortality was lower for those vaccinated against pneumococcal infection relative to the unvaccinated (2.1% vs. 4.5%, P < 0.001).[7] However, only 20% of the cohort received pneumococcal vaccination.[7] Five observational studies assessed serological response to pneumococcal vaccination in IBD patients on immunosuppressive medications compared to healthy controls or IBD patients not on immunosuppressive medications.[8-12] One study assessed the immunogenicity of sequential vaccination schedule of PCV13 followed by PPSV23.[8] The other four studies assessed the immunogenicity of PPSV23.[9-12] It is important to note that consensus on the correlates of clinical protection is lacking. The cut-off value of serotype-specific IgG antibody titers ≥ 0.35ug/mL was recommended by the WHO as protective for invasive pneumococcal disease (IPD). This cut-off is based on 3 clinical studies in children, who received PCV7. However, this cut-off is not serotype-specific. Comparisons across studies are difficult as studies compared different serotypes with different definitions of vaccination response. Some studies performed serotype specific assays of the individual antibody responses to the serotypes included in the vaccine, whereas others only provided overall response rates. The evidence suggest that pneumococcal vaccination can induce a serological response in a significant proportion of adult IBD patients. Immunosuppressive medications (e.g. immunomodulators, anti-TNF, combination therapy) may reduce the immunologic response to pneumococcal vaccination in IBD patients, particularly when combination immunosuppressive medications are used. However, it is uncertain if this reduced immunologic response is clinically relevant/important and would still afford clinical protection. No serious adverse events including disease exacerbation was reported.<br><br>Interestingly, one study suggested that patients on immunosuppressive medications had a lower seroconversion rates to serotypes present in both PCV13 and PPSV23 (50%) compared with the seroconversion to the serotypes exclusive to PPSV23 (70%).[8] The absence of a PPSV23 booster effect for PCV13 serotypes in immunocompromised patients has been described previously in recipients of allogenic hematopoietic stem cell transplant patients.[13] Multiple priming doses of PCV13 may be necessary.<br><br>The GRADE rating started at low due to the observational designs of these studies. The rating was downgraded to very low due to study limitations (selection bias, residual confounding, misclassification errors), indirectness (surrogate outcome), and imprecision. In particular, IBD patients who agreed to vaccination were likely to be systematically different than those who did not agree or seek vaccination (healthy vaccinee effect or confounding by indication). This may lead to selection bias confounding the vaccine’s effect on the outcomes (e.g. mortality, infection, adverse events, and even immunologic response). It is important to note that most patients included in the studies were age < 65. Very few elderly patients (age > 65) were included. Therefore, the evidence in elderly IBD patients was even more uncertain.<br><br>A Cochrane systematic review of 18 RCTs found pneumococcal polysaccharide vaccines to be effective in reducing IPD (OR 0.26, 95% CI 0.14-0.45) and all-cause pneumonia (OR 0.72, 95% CI 0.56-0.93) in adults.[14] In subgroup analyses, there was evidence of protective efficacy against IPD in healthy adults in low-income countries (OR 0.14, 95%	

		CI 0.03-0.61), healthy adults in high-income countries (OR 0.20, 95% CI 0.10-0.39), but not in adults with chronic disease in high-income countries with wide confidence interval (OR 1.56, 95% CI 0.35-6.94).[14] **Results of RCTs are consistent with a protective effect against IPD and all-cause pneumonia among generally healthy adults. Such trials have not demonstrated that PPSV 23 is efficacious against either IPD or all-cause pneumonia in populations at higher risk (due to imprecision), such as adults and children with underlying conditions that increase their risk of pneumococcal disease or highly immunosuppressed individuals of any age.** The evidence for effectiveness was moderate due to study limitations. **The evidence for effectiveness was anchored to the general population (moderate for IBD patients not on immunosuppressive medications), but was downgraded to low due to indirectness as the vaccine may be less immunogenic in IBD patients on immunosuppressive medications than in the general population.**<br><br>CDC evaluated the evidence of pneumococcal vaccines for immunocompromised adults. Due to the limited body of evidence on vaccine efficacy and safety among persons with immunocompromising conditions, both PCV13 and PPSV23 vaccines were evaluated using data for HIV-infected adults (1 RCT of PCV7 among HIV infected adults in Malawi, 1 RCT of PPSV23 among HIV-infected adults in Uganda as well as observational studies in US and Europe).[15] Overall, the quality of evidence was rated as **low or very low for PPSV23 and moderate or low for PCV13 among immunocompromised adults**. However, the desirable consequences were deemed to clearly outweigh undesirable consequences given the extremely high burden of pneumococcal diseases among immunocompromised adults. Therefore, the CDC recommends the use of both vaccines among immunocompromised adults.<br><br>In terms of safety, the Cochrane systematic review did not assess adverse events related to the use of pneumococcal vaccines.[14] In a systematic review of 18 studies (RCTs and observational studies) of anti-pneumococcal vaccine in 601 systemic lupus erythematosus (SLE) patients, no serious adverse events were reported.[16] The evidence was downgraded to low due to imprecision and indirectness (not IBD patients).[16] In 4 observational studies, no serious adverse events were reported after administration of pneumococcal vaccine in IBD patients.[8-11] **The certainty of evidence for safety of pneumococcal vaccines in adult IBD patients was moderate.**<br><br>**Overall, there is moderate certainty evidence that pneumococcal vaccines are safe and effective in adult IBD patients not on immunosuppressive medications. For IBD patients on immunosuppressive medications, the overall certainty of evidence was low.**	

**Certainty of evidence**	**What is the overall certainty of the evidence of effects?**<br><br>o Very low<br>o Low<br>o Moderate<br>o High<br><br>o No included studies		
**Values and Preferences**	**Is there important uncertainty about or variability in how much people value the main outcomes?**<br><br>o Important uncertainty or variability<br>o Possibly important uncertainty or variability<br>o Probably no important uncertainty or variability<br>o No important uncertainty or variability	Patients likely value patient-important outcomes (mortality, VPI, adverse effects) more than surrogate outcomes (immunogenicity).	
**Balance of effects**	**Does the balance between desirable and undesirable effects favor the intervention or the comparison?**<br><br>o Favors the comparison<br>o Probably favors the comparison<br>o Does not favor either the intervention or the comparison<br>o Probably favors the intervention no RF<br>o Favors the intervention with RF<br><br>o Varies<br>o Don't know		
**Resources required**	**How large are the resource requirements (costs)?**<br><br>o Large costs<br>o Moderate costs<br>o Negligible costs and savings<br>o Moderate savings<br>o Large savings<br><br>o Varies<br>o Don't know	CDC vaccine price list last reviewed/updated: July 1, 2019<br><br>**Brandname** / **CDC cost/dose** / **Private sector cost/dose**<br>Prevnar 13 TM / $137.01 / $188.26<br>Pneumovax®23 / $56.30 / $105.194	

**Certainty of Evidence of Required Resources**	**What is the certainty of the evidence of resource requirements (costs)?**<br><br>○ Very low<br>○ Low<br>○ Moderate<br>○ High<br><br>○ No included studies	The costs of delivering routine immunization services may vary widely across countries and different health system settings. See Immunization Costing Action Network (ICAN) Immunization Delivery Cost Catalogue. http://immunizationeconomics.org/ican-idcc.	
**Cost effectiveness**	**Does the cost-effectiveness of the intervention favor the intervention or the comparison?**<br><br>○ Favors the comparison<br>○ Probably favors the comparison<br>○ Does not favor either the intervention or the comparison<br>○ Probably favors the intervention<br>○ Favors the intervention with RF<br><br>○ Varies<br>○ No included studies no RF	There are no studies that addressed this question specifically in the IBD population.<br><br>In a cost effectiveness analysis, the addition of one dose of PCV 13 to the previously recommended PPSV23 doses for adults with selected immunocompromised conditions (HIV/AIDS, hematologic cancer, solid organ transplants, and end stage renal disease) was found to potentially reduce both disease and cost.[18] This model would cost $16 million (in 2009 US$) but provide off-setting savings of $21 million per cohort from the societal perspective.[18] However, assumptions about PPSV23 and PCV13 vaccine effectiveness were based on 2 RCTs and several observational studies conducted among HIV-infected adults. **Because no such studies have been conducted among other immunocompromised populations, further assumptions had to be made about the relative vaccine effectiveness in those groups.**<br><br>In another cost-effectiveness analysis with consideration of childhood PCV13 herd immunity, a single dose of PCV13 was found to be more cost-effective in immunocompromised individuals than other vaccination recommendations (combination of PCV13 and PPSV23).[19] A single PCV13 cost $70,937 per QALY gained compared to no vaccination, whereas combination of vaccinations cost $136,724/QALY.[17]	Cost effectiveness in IBD patients would depend on childhood herd immunity and effectiveness of vaccines (no data on clinical efficacy so far in IBD populations).
**Acceptability**	**Is the intervention acceptable to key stakeholders?**<br><br>○ No<br>○ Probably no<br>○ Probably yes<br>○ Yes<br><br>○ Varies<br>○ Don't know	A discrete choice experiment was conducted to determine the relative importance of vaccine and disease specific characteristics and acceptability for Dutch older adults (age > 50), including pneumococcal disease, herpes zoster, pertussis vaccination, and influenza vaccination.[20] Older adults are most likely to accept pneumococcal vaccination of the 4 vaccines (68.1%).[20]<br><br>In a study that assessed parents' and other adults' values for preventing disease associated with pneumococcal infection, both parents and community members assigned relatively high values to preventing meningitis, pneumonia, and complex otitis media.[21] When the value of preventing pneumococcal disease is incorporated into economic analyses, pneumococcal conjugate vaccine has a cost-effectiveness ratio in the range of other widely used health interventions (< 10,000 dollars per QALY at a vaccine cost of 58 dollars per dose).[21]	

Feasibility	Is the intervention feasible to implement?<br><br>○ No<br>○ Probably no<br>○ Probably yes<br>○ Yes<br><br>○ Varies<br>○ Don't know		

Conclusion – Adults (not on immunosuppressive therapy)

PICO 16A: In adult patients with IBD (not on immunosuppressive therapy) with a risk factor for pneumococcal disease, should pneumococcal vaccines be given?

moderate certainty of evidence

Direction – Yes (88%), Uncertain (12%)

Strength – Strong (78%)

Type of recommendation	Strong recommendation against the intervention	Conditional recommendation against the intervention	Conditional recommendation for either the intervention or the comparison	Conditional recommendation for the intervention	Strong recommendation for the intervention
	○	○	○	○	■
**Recommendation**	**Statement 16A: In adult patients with IBD not on immunosuppressive therapy with a risk-factor for pneumococcal disease, we recommend pneumococcal vaccines be given.**				
**Justification**					

**Subgroup considerations**	
**Implementation considerations**	
**Monitoring and evaluation**	• Ongoing monitoring of serious adverse events associated with pneumococcal vaccine in IBD patients
**Research priorities**	• Observational or RCTs to determine the clinical effectiveness of pneumococcal vaccine in IBD patients with assessment of patient-important outcomes (i.e. pneumococcal infection etc.)<br>• Observational studies to establish correlates of seroprotection against pneumococcal disease in IBD patients<br>• More RCTs are needed to compare single vs. booster vaccination strategies for pneumococcal vaccine (PCV13) in IBD patients on immunosuppressive medications

PICO: In adult patients with IBD (not on immunosuppressive therapy) without a risk factor for pneumococcal disease, should pneumococcal vaccines be given?
moderate certainty of evidence
Direction – Yes (12%), Uncertain (88%)
No recommendation

**Type of recommendation**	**Strong recommendation against the intervention**	**Conditional recommendation against the intervention**	**Conditional recommendation for either the intervention or the comparison**	**Conditional recommendation for the intervention**	**Strong recommendation for the intervention**

	○	○	○	○	○
**Recommendation**	**No recommendation. In adult patients with IBD not on immunosuppressive therapy without a risk-factor for pneumococcal disease, the consensus group could not make a recommendation for or against giving pneumococcal vaccines.**				
**Justification**					
**Subgroup considerations**					
**Implementation considerations**					
**Monitoring and evaluation**	• Ongoing monitoring of serious adverse events associated with pneumococcal vaccine in IBD patients				
**Research priorities**	• Observational or RCTs to determine the clinical effectiveness of pneumococcal vaccine in IBD patients with assessment of patient-important outcomes (i.e. pneumococcal infection etc.)<br>• Observational studies to establish correlates of seroprotection against pneumococcal disease in IBD patients<br>• More RCTs are needed to compare single vs. booster vaccination strategies for pneumococcal vaccine (PCV13) in IBD patients on immunosuppressive medications				

Evidence to Decision Table – Adults (on immunosuppressive therapy)

**PICO 16B**	**In adult patients with IBD (on immunosuppressive therapy), should**

	vaccination vs. no vaccination against pneumococcal disease be given?
**Population**	Adult patients with IBD (on immunosuppressive therapy)
**Intervention**	Vaccination against pneumococcal disease
**Comparator**	No vaccination against pneumococcal disease
**Outcome**	Mortality, VPI (pneumococcal infection), SAEs, Immunogenicity

	Judgement	Research evidence	Additional considerations
**Desirable Effects**	**How substantial are the desirable anticipated effects?**<br><br>○ Trivial<br>○ Small<br>○ Moderate<br>○ Large<br><br>○ Varies<br>○ Don't know	**Risk of Pneumococcal infection in adult IBD patients**<br><br>One systematic review and five observational studies addressed this PICO question.[1-6]<br><br>One systematic review assessed the incidence of invasive pneumococcal disease (IPD) in immunocompromised patients.[1] Five studies included patients with chronic inflammatory disease such as SLE, Sjogren's syndrome, polymyositis/dermatomyositis, COPD, asthma, RA, and IBD. No subgroup data was provided for IBD. Compared to healthy control cohorts (pooled incidence rate: 10/100,000 person-years), the incidence rate of IPD was increased in patients with chronic inflammatory disease (pooled incidence rate: 65/100,000 person-years).[1] One observational study found an increased risk of IPD among patients with autoimmune diseases (RA, SLE, CD) vs. healthy adults.[2] Two other observational studies also found an increased risk of IPD among IBD patients compared to non-IBD controls.[3,4] **Compared to non-IBD controls, the risk of IPD is about 1.5- to 2-fold higher in IBD patients.**<br><br>One cross-sectional case-control study used an administrative database (Nationwide Inpatient Sample) to compare the risks of hospitalization for pneumonia due to Streptococcus pneumoniae among adult IBD patients vs. non-IBD controls.[5] It is important to note that pneumonia treated as outpatients were excluded. After adjusting for various factors including comorbidities, risk factors for pneumonia, as well as patient and hospital characteristics, IBD patients did not demonstrate increased odds of hospitalization for Streptococcus pneumoniae compared to non-IBD controls.[5] Mortality during these admissions among IBD patients was not significantly higher than the control population.[5]<br><br>One retrospective cohort and nested case-control study found an increased risk of pneumonia among IBD patients compared to non-IBD patients after adjusting for age, disease type, health care utilization and comorbidities.6 Use of biologic medications, corticosteroids, and PPI were significantly associated with pneumonia.[6] As the most common etiologic agent of community acquired pneumonia in the US is pneumococcal pneumonia, the evidence was not downgraded for indirectness (outcome).	Vaccination titers may wane over time in immunosuppressed populations. It is unclear which of the antigens should be assessed, what levels of titers are to be considered protective, and whether and when to revaccinate patients with low titers. There may also be differences in response between serotypes. The clinical significance of decline in detectable antibodies is not clear since immune correlates of protection for pneumococcal polysaccharide vaccine have not been established and relatively limited clinical data are available regarding the duration of vaccine-induced protection against IPD. It is unclear if regular revaccination is needed because the incidence of pneumococcal infection in adults increases with age.
**Undesirable Effects**	**How substantial are the undesirable anticipated effects?**<br><br>○ Large<br>○ Moderate<br>○ Small<br>○ Trivial<br><br>○ Varies<br>○ Don't know		

		The GRADE rating started at high as these were considered prognostic studies (providing evidence about the likelihood of Streptococcal infection in patients with IBD). The rating was further downgraded to **low** due to study limitations (residual confounding factors, detection bias, admission bias, and misclassification bias). In particular, patients with IBD and respiratory symptoms may be more likely to be tested for, diagnosed with, and admitted for Streptococcus pneumoniae infection than non-IBD controls, thus creating an overestimate of the risk of Streptococcal infection among IBD patients**. In summary, there is low certainty evidence that adult IBD patients have an increased risk of pneumococcal infection compared to non-IBD patients.**<br><br>**Effectiveness and safety of Pneumococcal vaccines in adult IBD patients**<br><br>There was no RCT comparing pneumococcal vaccine with placebo or no treatment in adult patients with IBD to address this PICO question.<br><br>There are six observational studies that addressed this PICO question.[7-12] In one cross-sectional observational study utilizing administrative data extracted from the Veterans Health Administration in the US, one-year mortality was lower for those vaccinated against pneumococcal infection relative to the unvaccinated (2.1% vs. 4.5%, P < 0.001).[7] However, only 20% of the cohort received pneumococcal vaccination.[7] Five observational studies assessed serological response to pneumococcal vaccination in IBD patients on immunosuppressive medications compared to healthy controls or IBD patients not on immunosuppressive medications.[8-12] One study assessed the immunogenicity of sequential vaccination schedule of PCV13 followed by PPSV23.[8] The other four studies assessed the immunogenicity of PPSV23.[9-12] It is important to note that consensus on the correlates of clinical protection is lacking. The cut-off value of serotype-specific IgG antibody titers $\geq$ 0.35ug/mL was recommended by the WHO as protective for invasive pneumococcal disease (IPD). This cut-off is based on 3 clinical studies in children, who received PCV7. However, this cut-off is not serotype-specific. Comparisons across studies are difficult as studies compared different serotypes with different definitions of vaccination response. Some studies performed serotype specific assays of the individual antibody responses to the serotypes included in the vaccine, whereas others only provided overall response rates. The evidence suggest that pneumococcal vaccination can induce a serological response in a significant proportion of adult IBD patients. Immunosuppressive medications (e.g. immunomodulators, anti-TNF, combination therapy) may reduce the immunologic response to pneumococcal vaccination in IBD patients, particularly when combination immunosuppressive medications are used. However, it is uncertain if this reduced immunologic response is clinically relevant/important and would still afford clinical protection. No serious adverse events including disease exacerbation was reported.<br><br>Interestingly, one study suggested that patients on immunosuppressive medications had a lower seroconversion rates to serotypes present in both PCV13 and PPSV23 (50%) compared with the seroconversion to the serotypes exclusive to PPSV23 (70%).[8] The absence of a PPSV23 booster effect for PCV13 serotypes in immunocompromised patients has been described previously in recipients of allogenic hematopoietic stem cell transplant patients.[13] Multiple priming doses of PCV13 may be necessary.	

		The GRADE rating started at low due to the observational designs of these studies. The rating was downgraded to very low due to study limitations (selection bias, residual confounding, misclassification errors), indirectness (surrogate outcome), and imprecision. In particular, IBD patients who agreed to vaccination were likely to be systematically different than those who did not agree or seek vaccination (healthy vaccinee effect or confounding by indication). This may lead to selection bias confounding the vaccine's effect on the outcomes (e.g. mortality, infection, adverse events, and even immunologic response). It is important to note that most patients included in the studies were age < 65. Very few elderly patients (age > 65) were included. Therefore, the evidence in elderly IBD patients was even more uncertain.<br><br>A Cochrane systematic review of 18 RCTs found pneumococcal polysaccharide vaccines to be effective in reducing IPD (OR 0.26, 95% CI 0.14-0.45) and all-cause pneumonia (OR 0.72, 95% CI 0.56-0.93) in adults.[14] In subgroup analyses, there was evidence of protective efficacy against IPD in healthy adults in low-income countries (OR 0.14, 95% CI 0.03-0.61), healthy adults in high-income countries (OR 0.20, 95% CI 0.10-0.39), but not in adults with chronic disease in high-income countries with wide confidence interval (OR 1.56, 95% CI 0.35-6.94).[14] **Results of RCTs are consistent with a protective effect against IPD and all-cause pneumonia among generally healthy adults. Such trials have not demonstrated that PPSV 23 is efficacious against either IPD or all-cause pneumonia in populations at higher risk (due to imprecision), such as adults and children with underlying conditions that increase their risk of pneumococcal disease or highly immunosuppressed individuals of any age.** The evidence for effectiveness was moderate due to study limitations. **The evidence for effectiveness was anchored to the general population (moderate for IBD patients not on immunosuppressive medications), but was downgraded to low due to indirectness as the vaccine may be less immunogenic in IBD patients on immunosuppressive medications than in the general population.**<br><br>CDC evaluated the evidence of pneumococcal vaccines for immunocompromised adults. Due to the limited body of evidence on vaccine efficacy and safety among persons with immunocompromising conditions, both PCV13 and PPSV23 vaccines were evaluated using data for HIV-infected adults (1 RCT of PCV7 among HIV infected adults in Malawi, 1 RCT of PPSV23 among HIV-infected adults in Uganda as well as observational studies in US and Europe).[15] Overall, the quality of evidence was rated as **low or very low for PPSV23 and moderate or low for PCV13 among immunocompromised adults**. However, the desirable consequences were deemed to clearly outweigh undesirable consequences given the extremely high burden of pneumococcal diseases among immunocompromised adults. Therefore, the CDC recommends the use of both vaccines among immunocompromised adults.<br><br>In terms of safety, the Cochrane systematic review did not assess adverse events related to the use of pneumococcal vaccines.[14] In 6 RCTs (from CDC profile) on PCV13, no serious adverse events were identified. The evidence was downgraded to moderate due to indirectness. In a systematic review of 18 studies (RCTs and observational studies) of anti-pneumococcal vaccine in 601 systemic lupus erythematosus (SLE)	

		patients, no serious adverse events were reported.[16] The evidence was downgraded to low due to imprecision and indirectness (not IBD patients).[16] In 4 observational studies, no serious adverse events were reported after administration of pneumococcal vaccine in IBD patients.[8-11] **The certainty of evidence for safety of pneumococcal vaccines in adult IBD patients was moderate.**<br><br>**Overall, there is moderate certainty evidence that pneumococcal vaccines are safe and effective in adult IBD patients not on immunosuppressive medications. For IBD patients on immunosuppressive medications, the overall certainty of evidence was low.**	
**Certainty of evidence**	**What is the overall certainty of the evidence of effects?**<br><br>o Very low<br>o Low On IS<br>o Moderate<br>o High<br><br>o No included studies		
**Values and Preferences**	**Is there important uncertainty about or variability in how much people value the main outcomes?**<br><br>o Important uncertainty or variability<br>o Possibly important uncertainty or variability<br>o Probably no important uncertainty or variability<br>o No important uncertainty or variability	Patients likely value patient-important outcomes (mortality, VPI, adverse effects) more than surrogate outcomes (immunogenicity).	
**Balance of effects**	**Does the balance between desirable and undesirable effects favor the intervention or the comparison?**<br><br>o Favors the comparison<br>o Probably favors the comparison<br>o Does not favor either the intervention or the comparison<br>o Probably favors the intervention<br>o Favors the intervention<br><br>o Varies<br>o Don't know		

<table>
<tr>
<td>Resources required</td>
<td>How large are the resource requirements (costs)?

○ Large costs
○ Moderate costs
○ Negligible costs and savings
○ Moderate savings
○ Large savings

○ Varies
○ Don't know</td>
<td>CDC vaccine price list last reviewed/updated: July 1, 2019

<table><tr><th>Brandname</th><th>CDC cost/dose</th><th>Private sector cost/dose</th></tr><tr><td>Prevnar 13 TM</td><td>$137.01</td><td>$188.26</td></tr><tr><td>Pneumovax®23</td><td>$56.30</td><td>$105.194</td></tr></table></td>
<td></td>
</tr>
<tr>
<td>Certainty of Evidence of Required Resources</td>
<td>What is the certainty of the evidence of resource requirements (costs)?

○ Very low
○ Low
○ Moderate
○ High

○ No included studies</td>
<td>The costs of delivering routine immunization services may vary widely across countries and different health system settings. See Immunization Costing Action Network (ICAN) Immunization Delivery Cost Catalogue. http://immunizationeconomics.org/ican-idcc.</td>
<td></td>
</tr>
<tr>
<td>Cost effectiveness</td>
<td>Does the cost-effectiveness of the intervention favor the intervention or the comparison?

○ Favors the comparison
○ Probably favors the comparison
○ Does not favor either the intervention or the comparison
○ Probably favors the intervention
○ Favors the intervention

○ Varies
○ No included studies</td>
<td>There are no studies that addressed this question specifically in the IBD population.

In a cost effectiveness analysis, the addition of one dose of PCV 13 to the previously recommended PPSV23 doses for adults with selected immunocompromised conditions (HIV/AIDS, hematologic cancer, solid organ transplants, and end stage renal disease) was found to potentially reduce both disease and cost.[18] This model would cost $16 million (in 2009 US$) but provide off-setting savings of $21 million per cohort from the societal perspective.[18] However, assumptions about PPSV23 and PCV13 vaccine effectiveness were based on 2 RCTs and several observational studies conducted among HIV-infected adults. Because no such studies have been conducted among other immunocompromised populations, further assumptions had to be made about the relative vaccine effectiveness in those groups.

In another cost-effectiveness analysis with consideration of childhood PCV13 herd immunity, a single dose of PCV13 was found to be more cost-effective in immunocompromised individuals than other vaccination recommendations (combination of PCV13 and PPSV23).[19] A single PCV13 cost $70,937 per QALY gained compared to no vaccination, whereas combination of vaccinations cost $136,724/QALY.[17]</td>
<td>Cost effectiveness in IBD patients would depend on childhood herd immunity and effectiveness of vaccines (no data on clinical efficacy so far in IBD populations).</td>
</tr>
</table>

**Acceptability**	**Is the intervention acceptable to key stakeholders?**<br><br>○ No<br>○ Probably no<br>○ Probably yes<br>○ Yes<br><br>○ Varies<br>○ Don't know	A discrete choice experiment was conducted to determine the relative importance of vaccine and disease specific characteristics and acceptability for Dutch older adults (age > 50), including pneumococcal disease, herpes zoster, pertussis vaccination, and influenza vaccination.[20] Older adults are most likely to accept pneumococcal vaccination of the 4 vaccines (68.1%).[20]<br><br>In a study that assessed parents' and other adults' values for preventing disease associated with pneumococcal infection, both parents and community members assigned relatively high values to preventing meningitis, pneumonia, and complex otitis media.[21] When the value of preventing pneumococcal disease is incorporated into economic analyses, pneumococcal conjugate vaccine has a cost-effectiveness ratio in the range of other widely used health interventions (< 10,000 dollars per QALY at a vaccine cost of 58 dollars per dose).[21]	
**Feasibility**	**Is the intervention feasible to implement?**<br><br>○ No<br>○ Probably no<br>○ Probably yes<br>○ Yes<br><br>○ Varies<br>○ Don't know		

References:

1. van Aalst M, Lötsch F, Spijker R, van der Meer JTM, Langendam MW, Goorhuis A, Grobusch MP, de Bree GJ. Incidence of invasive pneumococcal disease in immunocompromised patients: A systematic review and meta-analysis. Travel Med Infect Dis. 2018 Jul - Aug;24:89-100.
2. Shea KM, Edelsberg J, Weycker D, Farkouh RA, Strutton DR, Pelton SI. Rates of pneumococcal disease in adults with chronic medical conditions. Open Forum Infect Dis. 2014 May 27;1(1):ofu024.
3. Kantsø B, Simonsen J, Hoffmann S, Valentiner-Branth P, Petersen AM, Jess T. Inflammatory Bowel Disease Patients Are at Increased Risk of Invasive Pneumococcal Disease: A Nationwide Danish Cohort Study 1977-2013. Am J Gastroenterol. 2015 Nov;110(11):1582-7.
4. Wotton CJ, Goldacre MJ. Risk of invasive pneumococcal disease in people admitted to hospital with selected immune-mediated diseases: record linkage cohort analyses. J Epidemiol Community Health. 2012 Dec;66(12):1177-81.
5. Stobaugh DJ, Deepak P, Ehrenpreis ED. Hospitalizations for vaccine preventable pneumonias in patients with inflammatory bowel disease: a 6-year analysis of the Nationwide Inpatient Sample. Clin Exp Gastroenterol. 2013 May 6;6:43-9.
6. Long MD, Martin C, Sandler RS, Kappelman MD. Increased risk of pneumonia among patients with inflammatory bowel disease. Am J Gastroenterol. 2013 Feb;108(2):240-8.
7. Case DJ, Copeland LA, Stock EM, Herrera HR, Pfanner TP. Pneumococcal vaccination rates in VHA patients with inflammatory bowel disease. Medicine (Baltimore). 2015 Feb;94(6):e417

8. van Aalst M, Garcia Garrido HM, van der Leun J, Meek B, van Leeuwen EMM, Löwenberg M, D'Haens GR, Ponsioen CYI, Grobusch MP, Goorhuis A. Immunogenicity of the currently recommended pneumococcal vaccination schedule in patients with inflammatory bowel disease. Clin Infect Dis. 2019 Mar 22.
9. Lee CK, Kim HS, Ye BD, Lee KM, Kim YS, Rhee SY, Kim HJ, Yang SK, Moon W, Koo JS, Lee SH, Seo GS, Park SJ, Choi CH, Jung SA, Hong SN, Im JP, Kim ES; Korean Association for the Study of Intestinal Diseases (KASID) Study. Patients with Crohn's disease on anti-tumor necrosis factor therapy are at significant risk of inadequate response to the 23-valent pneumococcal polysaccharide vaccine. J Crohns Colitis. 2014 May;8(5):384-91.
10. Dotan I, Werner L, Vigodman S, Agarwal S, Pfeffer J, Horowitz N, Malter L, Abreu M, Ullman T, Guzner-Gur H, Halpern Z, Mayer L. Normal response to vaccines in inflammatory bowel disease patients treated with thiopurines. Inflamm Bowel Dis. 2012 Feb;18(2):261-8.
11. Fiorino G, Peyrin-Biroulet L, Naccarato P, Szabò H, Sociale OR, Vetrano S, Fries W, Montanelli A, Repici A, Malesci A, Danese S. Effects of immunosuppression on immune response to pneumococcal vaccine in inflammatory bowel disease: a prospective study. Inflamm Bowel Dis. 2012 Jun;18(6):1042-7.
12. Melmed GY, Agarwal N, Frenck RW, Ippoliti AF, Ibanez P, Papadakis KA, Simpson P, Barolet-Garcia C, Ward J, Targan SR, Vasiliauskas EA. Immunosuppression impairs response to pneumococcal polysaccharide vaccination in patients with inflammatory bowel disease. Am J Gastroenterol. 2010 Jan;105(1):148-54.
13. Cordonnier C, Ljungman P, Juergens C, Maertens J, Selleslag D, Sundaraiyer V, Giardina PC, Clarke K, Gruber WC, Scott DA, Schmoele-Thoma B; 3003 Study Group. Immunogenicity, safety, and tolerability of 13-valent pneumococcal conjugate vaccine followed by 23-valent pneumococcal polysaccharide vaccine in recipients of allogeneic hematopoietic stem cell transplant aged ≥2 years: an open-label study. Clin Infect Dis. 2015 Aug 1;61(3):313-23
14. Moberley S, Holden J, Tatham DP, Andrews RM. Vaccines for preventing pneumococcal infection in adults. Cochrane Database Syst Rev. 2013 Jan 31;(1):CD000422.
15. Centers for Disease Control and Prevention. https://www.cdc.gov/vaccines/acip/recs/grade/table-refs.html
16. Adawi M, Bragazzi NL, McGonagle D, Watad S, Mahroum N, Damiani G, Conic R, Bridgewood C, Mahagna H, Giacomelli L, Eggenhöffner R, Mahamid M, Pigatto PDM, Amital H, Watad A. Immunogenicity, safety and tolerability of anti-pneumococcal vaccination in systemic lupus erythematosus patients: An evidence-informed and PRISMA compliant systematic review and meta-analysis. Autoimmun Rev. 2019 Jan;18(1):73-92.
17. Centers for Disease Control and Prevention. https://www.cdc.gov/vaccines/acip/recs/grade/table-refs.html
18. Cho BH, Stoecker C, Link-Gelles R, Moore MR. Cost-effectiveness of administering 13-valent pneumococcal conjugate vaccine in addition to 23-valent pneumococcal polysaccharide vaccine to adults with immunocompromising conditions. Vaccine. 2013 Dec 5;31(50):6011-21.
19. Smith KJ, Nowalk MP, Raymund M, Zimmerman RK. Cost-effectiveness of pneumococcal conjugate vaccination in immunocompromised adults. Vaccine. 2013 Aug 20;31(37):3950-6.
20. Eilers R, de Melker HE, Veldwijk J, Krabbe PFM. Vaccine preferences and acceptance of older adults. Vaccine. 2017 May 15;35(21):2823-2830.
21. Prosser LA, Ray GT, O'Brien M, Kleinman K, Santoli J, Lieu TA. Preferences and willingness to pay for health states prevented by pneumococcal conjugate vaccine. Pediatrics. 2004 Feb;113(2):283-90.

Conclusion – Adults (on immunosuppressive therapy)

PICO 16B: In adult patients with IBD (on immunosuppressive therapy), should pneumococcal vaccines be given?
Low certainty of evidence
Direction – Yes (100%)
Strength - Conditional

Type of recommendation	Strong recommendation against the intervention	Conditional recommendation against the intervention	Conditional recommendation for either the intervention or the comparison	Conditional recommendation for the intervention	Strong recommendation for the intervention
	○	○	○	○	○
**Recommendation**	**Statement 16B: In adult patients with IBD on immunosuppressive therapy, we suggest pneumococcal vaccines be given.**				
**Justification**					
**Subgroup considerations**					
**Implementation considerations**					
**Monitoring and evaluation**	• Ongoing monitoring of serious adverse events associated with pneumococcal vaccine in IBD patients				
**Research priorities**	• Observational or RCTs to determine the clinical effectiveness of pneumococcal vaccine in IBD patients with assessment of patient-important outcomes (i.e. pneumococcal infection etc.)				

	• Observational studies to establish correlates of seroprotection against pneumococcal disease in IBD patients<br>• More RCTs are needed to compare single vs. booster vaccination strategies for pneumococcal vaccine (PCV13) in IBD patients on immunosuppressive medications

Meningococcal Disease

Background

Meningococcal disease is an infection caused by *Neisseria meningitidis*. Most meningococcal infections involve meningitis and meningococcal septicemia. In particular, meningococcal meningitis is associated with high fatality (up to 50% when untreated) and high frequency (10-20%) of severe long-term sequelae including seizure disorders, motor deficits, hearing or vision loss, orthopedic complications such as digit or limb amputations, intellectual and cognitive impairment. Almost all invasive meningococcal disease (IMD) is associated with serogroups A, B, C, Y, and W-135.

Worldwide, the incidence of endemic meningococcal disease is low (0.5 to 5 per 100,000 persons), with the incidence being highest in children younger than 1 year old, followed by adolescence (16 through 23 years old) and older individuals (> age 60). Rates of meningococcal disease have been declining in the US. In 2017, there were about 350 total cases of meningococcal disease reported (incidence rate 0.11 per 100,000 persons). The Canadian Enhanced Meningococcal Surveillance System detected 154 to 229 cases of IMD annually from 2006 through 2011, for an incidence of 0.55 cases per 100,000 persons per year. The prevalence may increase with concomitant viral upper respiratory tract infection, for persons living in crowded living conditions (college students, military recruits, persons of low socioeconomic status), and with both passive and active smoking.[1] Outbreaks tend to occur in semi-closed communities (e.g. military camps, college residences, schools, day-care centers).

There are 2 types of meningococcal vaccines available in the US: meningococcal conjugate vaccines (or MenACWY) and serogroup B meningococcal vaccines (MenB). In addition, the monovalent conjugate meningococcal vaccine (Men-C-C) is available in Canada. CDC ACIP recommends routine administration of meningococcal conjugate vaccine (MenACWY) to all 11 to 12 year olds, and a booster dose at age 16.[2] Adolescents and young adults (16 through 23 years old) may also receive a serogroup B meningococcal vaccine (MenB).[2] **In the US, CDC does not recommend routine meningococcal vaccination to infants due to low burden of disease**

and low proportion of meningococcal cases that are preventable with vaccines that do not protect against serogroup B disease. On the other hand, **NACI recommends routine administration of Men-C-C vaccine to healthy children at 12 months of age (or earlier), and to all adolescents and young adults (11 or 12 years of age, 12 to 24 years of age) with either Men-C-C or MenACWY as a booster**.[3] If not previously immunized as infants or toddlers, children less than 11 years of age should also receive the Men-C-C vaccine.[3] In addition, the multicomponent meningococcal (4CMenB) vaccine may be considered on an individual basis for children, adolescents, and young adults to protect against serogroup B strains.[3]

Both CDC and NCI[2,3] recommend routine meningococcal conjugate and serogroup B meningococcal vaccination for children and adults who are at increased risk for invasive meningococcal disease (IMD):

Persons at higher risks of IMD include:

- Persons with functional or anatomic asplenia, including sickle cell disease. In patients with hyposplenism, the ability to produce antibodies against polysaccharides is diminished and may contribute to the increased risk of infection by encapsulated organisms.
- Persons with congenital complement, properdin, factor D or primary antibody deficiencies
- Persons with acquired complement deficiency due to receipt of terminal complement inhibitor eculizumab
- HIV positive individuals
- Travelers to areas with high rates of endemic meningococcal disease or transmission
- Research, industrial and clinical laboratory personnel who may be at risk of exposure to *Neisseria meningitidis*
- Military personnel during recruit training and on certain deployments
- Persons at increased risk because of disease outbreak

NACI also recommends that individuals at high risk of developing meningococcal disease due to underlying medical conditions receive periodic booster doses every 3 – 5 years.

The ACG recommends that adolescents with IBD receive meningococcal vaccination in accordance with routine vaccination recommendations (*conditional recommendation, very low level evidence*).[4]

Men-C-C and Men-C-ACYW vaccines are immunogenic in infants, toddlers and adolescents, but available data suggest a waning immune response over time (within 5 years). Available data on MenB vaccines suggest that protective antibodies also decrease

quickly (within 1 to 2 years) after vaccination. Serologic testing is not recommended before or after receiving meningococcal vaccine. No significant increased risk for serious adverse events has been identified in clinical trials.

References:

1. Gardner P. Prevention of meningococcal disease. N Engl J Med. 2006;355:1466-1474.
2. Centers for Disease Control and Prevention. https://www.cdc.gov/flu/professionals/vaccination/index.htm
3. Public Health Agency of Canada. https://www.canada.ca/en/public-health/services/publications/healthy-living/canadian-immunization-guide-part-4-active-vaccines/page-13-meningococcal-vaccine.html
4. Farraye FA, Melmed GY, Lichtenstein GR, Kane SV. ACG Clinical Guideline: Preventive Care in Inflammatory Bowel Disease. Am J Gastroenterol. 2017 Feb;112(2):241-258.

Risk of Meningococcal infection in IBD patients

PICO: What is the risk of meningococcal infection in people with IBD compared to people without IBD?

Summary – Adults and Pediatric

Literature search did not identify any study on the risk of meningococcal infection in adult or pediatric IBD patients.

Since 1974, there have been several reports of the association between IBD and hyposplenism based on indirect measurements of splenic function (e.g. heated red cell clearance, pitted erythrocyte counts e.g. Howell-Jolly bodies, differential interference contrast microscopy, splenic size from ultrasound).[1-8] However, **the prevalence of functional hyposplenism is uncertain in IBD populations due to paucity of evidence**. Of note, functional hyposplenism has also been reported to occur in patients with other GI conditions such as celiac disease, Whipple's disease, idiopathic ulcerative enteritis, tropical sprue, alcoholic liver disease, autoimmune atrophic gastritis, and long-term parenteral nutrition, as well as other autoimmune conditions such as systemic lupus erythematosus, thyroid disease, rheumatoid arthritis, sarcoidosis, and amyloidosis. However, the exact mechanisms of hyposplenia in these conditions are poorly understood. Furthermore, **the clinical significance of functional hyposplenism is unclear as there are no studies assessing the risks of meningococcal infection in IBD patients.**

One case report described a 42 year-old woman who presented with fulminant meningococcal sepsis with hyposplenism that was diagnosed on computed tomography (CT) scan.[9] The hyposplenism was proposed to be possibly related to IBD. Another case report described meningococcal meningoencephalitis after certolizumab pegol treatment in a 51 year-old woman with Crohn's disease.[10]

Due to the designs of these studies (case series/reports), the GRADE rating already started at **very low** for higher risks of functional hyposplenism in IBD patients compared to the general population. It is even more uncertain whether this finding translates into higher risks of infection by encapsulated organisms such as Streptococcus pneumoniae, Neisseria meningitis, Haemophilus influenzae.

References:

1. Rameh BS, Stevens FM, McCarthy CF. Hyposplenism in inflammatory bowel disease. Ir J Med Sci. 1988 Jan;157(1):8-9.
2. Muller AF, Cornford E, Toghill PJ. Splenic function in inflammatory bowel disease: assessment by differential interference microscopy and splenic ultrasound. Q J Med. 1993 May;86(5):333-40.
3. Ryan FP, Smart RC, Holdsworth CD, Preston FE. Hyposplenism in inflammatory bowel disease. Gut. 1978 Jan;19(1):50-5.
4. Ryan FP, Smart R, Preston FE, Holdsworth CD. Hyposplenism in ulcerative colitis. Lancet. 1974 Aug 10;2(7876):318-20.
5. Palmer KR, Sherriff SB, Holdsworth CD, Ryan FP. Further experience of hyposplenism in inflammatory bowel disease. Q J Med. 1981 Autumn;50(200):463-71.
6. Jewell DP, Berney JJ, Pettit JE. Splenic phagocytic function in patients with inflammatory bowel disease. Pathology. 1981 Oct;13(4):717-23.
7. Di Sabatino A, Rosado MM, Ciccocioppo R, Cazzola P, Morera R, Corazza GR, Carsetti R. Depletion of immunoglobulin M memory B cells is associated with splenic hypofunction in inflammatory bowel disease. Am J Gastroenterol. 2005 Aug;100(8):1788-95.
8. Muller AF, Toghill PJ. Hyposplenism in gastrointestinal disease. Gut. 1995 Feb;36(2):165-7.
9. Shah A, Lettieri CJ. Fulminant meningococcal sepsis in a woman with previously unknown hyposplenism. Medscape J Med. 2008 Feb 13;10(2):36.
10. Majumder S, Kumar A. Meningococcal meningoencephalitis after certolizumab pegol treatment in a patient with Crohn's disease. J Crohns Colitis. 2013 Feb;7(1):e19.

Effectiveness and Safety of Meningococcal vaccine in IBD patients

Summary – Pediatric

**PICO 17**	**In pediatric patients with IBD, should age appropriate vaccination vs. no vaccination against meningococcal disease be given?**
**Population**	Pediatric patients with IBD
**Intervention**	Age appropriate vaccination against meningococcal disease

Comparator	No age appropriate vaccination against meningococcal disease
Outcome	Mortality, VPI (meningococcal infection), SAEs, Immunogenicity

There was no RCT or observational studies comparing meningococcal vaccine with placebo or no treatment in pediatric patients with IBD to address this PICO question.

A Cochrane systematic review with inclusion of 8 RCTs (n = 480,068) found **polysaccharide serogroup A vaccine** to be strongly protective for serogroup A meningococcal meningitis for first year in children over five and adults with summary vaccine efficacy of 95% (95% CI 87-99%).[1] Protection extended into the second and third year after vaccination, but the results did not reach statistical significance.[1] The vaccine was protective in Finnish children aged 3 months to 5 years.[1] The latter was also the only trial that assessed the effect of a booster dose in children under 2 years of age, but lacked power to yield statistically significant results. The vaccine was protective in one- to five-year old children in developing countries (Nigeria and Sudan).[1] The quality of the trials was considered to be high.[1]

The relatively low incidence of meningococcal C disease has so far precluded any RCTs assessing the efficacy of **meningococcal serogroup C conjugate vaccines** against clinical endpoints. A Cochrane systematic review found meningococcal serogroup C conjugate vaccines to be highly immunogenic in infants for preventing meningococcal C meningitis and septicaemia.[2] The effectiveness of meningococcal serogroup C conjugate vaccine was described only in observational studies.[2] The introduction of meningococcal C vaccines into routine immunization programs in Europe, Canada, and Australia have proven to be effective, with dramatic reduction in the incidence of meningococcal serogroup C disease.[2] The overall quality of the trials was considered good.[2] The WHO has evaluated the evidence for the use of meningococcal serogroup C conjugate vaccines and quadrivalent meningococcal vaccines in children using GRADE methodology. There is **moderate** certainty evidence that meningococcal serogroup C conjugate vaccines protects children aged $\geq$ 2 months to < 5 years and individuals aged $\geq$ 5 years against meningococcal disease. In addition, there is **moderate** certainty evidence that quadrivalent meningococcal conjugate vaccines protects children aged $\geq$ 12 months to < 5 years against meningococcal disease, and **low** certainty evidence that quadrivalent meningococcal conjugate vaccines protects individuals aged $\geq$ 5 years against meningococcal disease. There is **moderate** certainty evidence that meningococcal vaccines are not associated with serious adverse effects.

The low incidence of meningococcal B disease has also precluded any RCTs assessing the efficacy of serogroup B meningococcal vaccines against clinical endpoints. Vaccine effectiveness of serogroup B meningococcal vaccines was therefore inferred based on surrogate outcomes of immunogenicity. CDC considered the quality of evidence **low** for the use of meningococcal serogroup B

vaccines in adolescents and young adults (persistence in immunogenicity 11-24 months), and also in persons at increased risk of serogroup B meningococcal disease.

It is important to note that the evidence was not downgraded due to indirectness related to patient population (general population vs. IBD patients) since there is no reason to suspect that IBD patients are at lower risks for developing meningococcal infection than non-IBD patients. On the contrary, there is reason to suspect that IBD patients may be at higher risks for developing meningococcal infection than non-IBD patients due to the very small number of case reports of functional hyposplenism in these patients, and the use of immunosuppressive medications. There is also no evidence to suggest that the meningococcal vaccines are harmful or less effective in IBD patients. Therefore, the evidence was anchored at the general population since there is no reason to deviate from country-specific immunization guidelines for the general population with protocols based on local epidemiologic, programmatic, resource, policy, disease control objectives and strategies.

In summary, there is moderate certainty evidence that meningococcal serogroup C conjugate vaccines are safe and effective in reducing the risk of meningococcal disease in pediatric IBD patients.

There is moderate certainty evidence that quadrivalent meningococcal conjugate vaccines are safe and effective in pediatric IBD patients aged ≥ 12 months to < 5 years, and low certainty evidence that quadrivalent meningococcal conjugate vaccines are safe and effective in pediatric IBD patients aged ≥ 5 years.

There is low certainty evidence that serogroup B meningococcal vaccines are safe and effective in reducing the risk of serogroup B meningococcal disease in pediatric IBD patients (adolescents and young adults).

References:

1. Patel M, Lee CK. Polysaccharide vaccines for preventing serogroup A meningococcal meningitis. Cochrane Database Syst Rev. 2005 Jan 25;(1):CD001093.
2. Conterno LO, Silva Filho CR, Rüggeberg JU, Heath PT. Conjugate vaccines for preventing meningococcal C meningitis and septicaemia. Cochrane Database Syst Rev. 2006 Jul 19;(3):CD001834.

Evidence Profile Table – Pediatric

The evidence profile tables from the WHO and CDC for the general population are included below:

Meningococcal serogroup C conjugate vaccines in children (from WHO Evidence Profile Tables)

https://www.who.int/immunization/policy/position_papers/meningococcal/en/

WHO rating of quality of evidence:
1 – very low
2 – low
3 – moderate
4 – high

Table II a. Efficacy of MenC conjugate vaccines. Do conjugated MC group C-vaccines protect children aged ≥2 months to <5 years against invasive meningococcal disease?				
			Rating	Adjustment to level
Quality Assessment	No of Studies/Quality starting level		1 RCT	4
	Factors decreasing confidence	Limitation in study design	None serious	0
		Inconsistency	None serious	0
		Indirectness	Serious[1]	-1
		Imprecision	None serious	0
		Publication bias	None serious	0
	Factors increasing confidence	Large effect	Not applicable	0
		Dose-response	Not applicable	0
		Mitigated bias and confounding	Not applicable	0
	Final rating of quality of evidence			3
Summary of Findings	Statement on quality of evidence			We are moderately confident in the estimate of effect on the health outcome
	Conclusion			Conjugated MC group C-vaccines protects children aged ≥2 months to <5 years against meningococcal disease.

[1]Immunogenicity rather than clinical protection is used as an endpoint. Serum bactericidal activity at titres ≥1:4 (tests using human complement, hSBA) or 1:8 (tests using rabbit complement, rSBA) are considered reliable immunologic correlates of protection. References providing the rationale for this conclusion include *Borrow RP et al 2005; Adrews NR et al 2003*; and *Goldschneider IEC 1969*.

Table II b. Efficacy of MenC conjugate vaccines. Do conjugated MC group C-vaccines protect individuals aged ≥5 years against invasive meningococcal disease?				
			Rating	Adjustment to level
Quality Assessment	No of Studies/Starting quality starting level		2 RCTs	4
	Factors decreasing confidence	Limitation in study design	None serious	0
		Inconsistency	None serious	0
		Indirectness	Serious[1]	-1
		Imprecision	None serious	0
		Publication bias	None serious	0
	Factors increasing confidence	Large effect	Not applicable	0
		Dose-response	Not applicable	0
		Mitigated bias and confounding	Not applicable	0
	Final rating of quality of evidence			3
Summary of Findings	Statement on quality of evidence			We are moderately confident in the estimate of effect on the health outcome
	Conclusion			Conjugated MC group C-vaccines protect individuals aged ≥5 years against meningococcal disease.

[1]Immunogenicity rather than clinical protection is used as an endpoint. Serum bactericidal activity (SBA) at titres ≥1:4 (tests using human complement, hSBA) or 1:8 (tests using rabbit complement, rSBA) are considered reliable immunologic correlates of protection. (For references providing the rationale for this conclusion, see Table IIIa).

Quadrivalent meningococcal conjugate vaccines in children (from WHO Evidence Profile Tables)

https://www.who.int/immunization/policy/position_papers/meningococcal/en/

Table VI a. Efficacy of quadrivalent meningococcal conjugate vaccines.
Do combined conjugated MC vaccines protect children aged ≥12 months to <5 years against invasive meningococcal disease?

			Rating	Adjustment to level
Quality Assessment	No of Studies/Starting quality level		1 RCT + 1 observational	4
	Factors decreasing confidence	Limitation in study design	None serious	0
		Inconsistency	None serious	0
		Indirectness	Serious[1]	-1
		Imprecision	None serious	0
		Publication bias	None serious	0
	Factors increasing confidence	Large effect	Not applicable	0
		Dose-response	Not applicable	0
		Mitigated bias and confounding	Not applicable	0
	Final rating of quality of evidence			3
Summary of Findings	Statement on quality of evidence			We are moderately confident in the estimate of effect on the health outcome.
	Conclusion			Combined conjugated MC vaccines protect against meningococcal disease in children ≥12 months to <5 years of age.

[1]Immunogenicity rather than clinical protection is used as an endpoint. Serum bactericidal activity (SBA) at titres ≥1:4 (tests using human complement, hSBA) or 1:8 (tests using rabbit complement, rSBA) are considered reliable immunologic correlates of protection. References providing the rationale for this conclusion include *Borrow RP et al 2005; Andrews NR et al 2003*; and *Goldschneider IEC 1969*.

Table VI b. Efficacy of quadrivalent meningococcal conjugate vaccines.
Do combined conjugated MC vaccines protect individuals aged ≥5 years against invasive meningococcal disease?

			Rating	Adjustment to level
Quality Assessment	No of Studies/Starting quality level		1 RCT + 1 observational	4
	Factors decreasing confidence	Limitation in study design	None serious	0
		Inconsistency	None serious	0
		Indirectness	Serious[1]	-1
		Imprecision	None serious	0
		Publication bias	Serious[2]	-1
	Factors increasing confidence	Large effect	Not applicable	0
		Dose-response	Not applicable	0
		Mitigated bias and confounding	Not applicable	0
	Final rating of quality of evidence			2
Summary of Findings	Statement on quality of evidence			Our confidence in the estimate of the effect on the health outcome is limited.
	Conclusion			Limited evidence that vaccination with combined conjugated MC vaccines protect individuals aged ≥5years against invasive meningococcal disease..

[1]Immunogenicity rather than clinical protection is used as an endpoint. Serum bactericidal activity (SBA) at titres ≥1:4 (tests using human complement, hSBA) or 1:8 (tests using rabbit complement, rSBA) are considered reliable immunologic correlates of protection. (For references providing the rationale for this conclusion, see Table Va).

[2]Studies conducted by the manufacturer. Internationally published evidence so far limited.

Serogroup B Meningococcal Vaccines in adolescents and young adults (from CDC Evidence Profile Tables)

CDC rating of quality of evidence:
1 – high
2 – moderate
3 – low
4 – very low

Table 1a: Use of MenB-4C (Bexsero®) in adolescents and young adults (including college students): Evidence Table

Outcomes	Design (# studies)	Initial Evidence	Risk of Bias	Inconsistency	Indirectness	Imprecision	Publication Bias	Others	Final Evidence	Overall Evidence Type
**Benefits**										
Short-term immunogenicity	3 RCTs	1	Not Serious	Serious** (-1)	Serious*** (-1)	Not Serious	Unable to assess	Yes## (+1)	2	2
	1 Obs	3	Not serious	Not serious	Serious*** (-1)	Not Serious	Unable to assess	None	4	
Persistence of immunogenicity (11-24 months)	2 RCTs	1	Serious* (-1)	Not serious	Serious*** (-1)	Not Serious	Unable to assess	None	3	3
MenB Immunogenicity with concomitant vaccination	No available studies									
**Harms**										
Serious Adverse Events	3 RCTs	1	Not serious	Not serious	Not Serious	Serious# (-1)	Unable to assess	None	2	2
Safety with Concomitant vaccination (SAEs)	No available studies									

Footnotes:
* No formal statistical hypothesis testing or sample size calculation planned in the protocol for one study. Potential selection bias for participants in the other study – downgraded by 1
** High heterogeneity, I-squared > 90% across all strains – downgraded by 1
*** Studies assessed correlate of protection and not directly efficacy – downgraded by 1
# The CI around the effect estimate includes both effect and non-effect – downgraded by 1
Strong strength of association. RR ranges between 4.44 and 5.19 – upgraded by 1

Table 1b: Considerations for Vaccine Use: MenB-4C (Bexsero®)

Key Factors	Comments
Balance between benefits and harms	Among healthy adolescents and young adults (including college students), the vaccine is immunogenic in the short-term and persists 1-2 years after vaccination. Low disease burden lowers overall benefits.
**Evidence type for benefits and harms**	
MenB-4C vaccine use among healthy adolescents and young adults (including college students)	**Benefits:**<br>Short-term immunogenicity: **Evidence Type 2**<br>Persistence in immunogenicity (11-24 months): **Evidence Type 3**<br>MenB immunogenicity with concomitant vaccination: **Not assessed**<br><br>**Harms:**<br>Serious Adverse Events: **Evidence Type 2**<br>SAEs following concomitant vaccination: **Not assessed**

Table 2a: Use of MenB-FHbp (Trumenba®) in adolescents and young adults (including college students): Evidence Table

Outcome	Design (#studies)	Initial Evidence	Risk of Bias	Inconsistency	Indirectness	Imprecision	Publication Bias	Others	Final Evidence Type	Overall Evidence Type
**Benefits**										
Short-term Immunogenicity	2 RCTs	1	Not serious	Serious** (-1)	Serious *** (-1)	Not serious	Unable to assess	Yes## (+1)	2	2
	1 Obs	3	Not serious	Not applicable	Serious *** (-1)	Not serious	Unable to assess	None	4	
Persistence in Immunogenicity 48 months post vaccination	1 Obs	3	Serious* (-1)	Not applicable	Serious *** (-1)	Minor *#	Unable to assess	None	4	4
MenB immunogenicity with concomitant vaccination (Non-inferiority) +	2 RCTs	1	Not serious	Not serious	Serious *** (-1)	Not serious	Unable to assess	None	2	2
**Harms**										
Serious Adverse Events (SAEs)	5 RCTs	1	Not serious	Not serious	Not serious	Serious # (-1)	Unable to assess	None	2	2
Safety with Concomitant vaccination (SAEs)	2 RCTs	1	Not serious	Not serious	Not serious	Serious # (-1)	Unable to assess	None	2	2

Footnotes:
+ Concomitant administration with Tdap/IPV or 4vHPV
* Very small sample size
**Significant heterogeneity; I-square ranges between 43-81% — Downgraded 1
*** Studies assessed correlate of protection and not directly efficacy – downgraded by 1
*# The CI around the effect estimate includes both effect and non-effect in two strains not common in the U.S.
The CI around the effect estimate includes both effect and non-effect – downgraded by 1
Very strong strength of association: relative risk ranges between 4.64 between 12.26 – upgraded by 1

Table 2b: Considerations for Vaccine Use: MenB-FHbp (Trumenba®)

Key Factors	Comments
Balance between benefits and harms	Among healthy adolescents and young adults (including college students), the vaccine is immunogenic in the short-term and persists up to 4 years after vaccination. MenB-FHbp is safe for concomitant vaccination with 4vHPV, MenACWY, Tdap and Tdap/IPV. Low disease burden lowers overall benefits.
**Evidence type for benefits and harms**	
**MenB-FHbp vaccine use among healthy adolescent and young adults (including college students)**	**Benefits:**<br>Short term immunogenicity: **Evidence Type 2**<br>Persistence in Immunogenicity (48 months): **Evidence Type 4**<br>MenB immunogenicity with concomitant vaccination: **Evidence Type 2**<br>**Harms:**<br>Serious Adverse Events: **Evidence Type 2**<br>SAEs following concomitant vaccination: **Evidence Type 2**

Serogroup B Meningococcal Vaccines in Persons at Increased Risk for Serogroup B Meningococcal Disease (from CDC Evidence Profile Tables)

Table 1a: Use of MenB-4C (Bexsero®) in Persons at Increased Risk: Evidence Table

Outcome	Design (# studies)	Initial Evidence	Risk of Bias	Inconsistency	Indirectness	Imprecision	Publication Bias	Others	Final Evidence	Overall Evidence Type
					**Benefits**					
**Short-term immunogenicity**	3 RCTs	1	Not Serious	Serious[*] (-1)	Serious[**] (-2)	Not Serious	Unable to assess	Yes[##] (+1)	3	3
	2 Obs	3	Not serious	Not serious	Serious[#] (-2)	Not Serious	Unable to assess	None	4	
**Persistence of immunogenicity (11-24 months)**	2 RCTs	1	Serious[#*] (-1)	Not serious	Serious[**] (-2)	Not Serious	Unable to assess	None	4	4
					**Harms**					
**Serious Adverse Events**	3 RCTs	1	Not serious	Not serious	Serious[>] (-1)	Serious[***] (-1)	Unable to assess	None	3	3

Footnotes:

*** High heterogeneity, I-squared > 90% across all strains – downgraded by 1**

**** Focused on healthy adolescents and young adults, not persons at increased risk of serogroup B meningococcal disease; studies assessed correlate of protection and not directly efficacy – downgraded by 2**

## Strong strength of association. RR ranges between 2.46 and 4.25 – upgraded by 1

# One study focused on laboratory workers only, other groups at increased risk (e.g., persons with compliment deficiencies or asplenia) not considered; studies assessed correlate of protection and not directly efficacy – downgraded by 2

#* No formal statistical hypothesis testing or sample size calculation planned in the protocol for one study. Potential selection bias for participants in the other study – downgraded by 1

> Focused on healthy adolescents and young adults, not persons at increased risk of serogroup B meningococcal disease – downgraded by 1

*****The CI around the effect estimate includes both effect and non-effect – downgraded by 1**

Table 1c: Considerations for Vaccine Use: MenB-4C (Bexsero®)

Key Factors	Comments
**Balance between benefits and harms**	Vaccine is immunogenic in the short-term, and immunogenicity persists (1-2 years) for healthy adolescents and adults, and is safe. Low disease burden lowers overall benefits.
**Evidence type for benefits and harms**	
**Use of MenB-4C in persons at increased risk**	Benefits: Evidence Type: 3<br>Harms: Evidence Type: 3<br>**Overall: Evidence Type: 3**
**Use of MenB-4C during outbreaks**	Benefits: Evidence Type: 2<br>Harms: Evidence Type: 2<br>**Overall: Evidence Type: 2**

Table 2a: Use of MenB-FHbp (Trumenba®) in Persons at Increased Risk: Evidence Table

Outcome	Design (#studies)	Initial Evidence	Risk of Bias	Inconsistency	Indirectness	Imprecision	Publication Bias	Others	Final Evidence Type	Overall Evidence Type
**Benefits**										
Short-term Immunogenicity	2 RCTs	1	Not serious	Serious[*] (-1)	Serious[**] (-2)	Not serious	Unable to assess	Yes[***] (+1)	3	3
	3 Obs	3	Serious[>] (-1)	Minor	Serious[**] (-2)	Not serious	Unable to assess	None	4	
**Harms**										
Serious Adverse Events	5 RCTs	1	Not serious	Not serious	Serious[#] (-1)	Serious[##] (-1)	Unable to assess	None	3	3

Footnotes:
* Significant heterogeneity; I-square ranges between 40-91% – Downgraded 1
** Studies focused on healthy adolescents and young adults, not persons at increased risk of serogroup B meningococcal disease; studies assessed correlate of protection and not directly efficacy – downgraded by 2
*** Very strong strength of association: relative risk ranges between 4 between 9 – upgraded by 1
> In one study, no statistical consideration taken into account when determining sample size; sample size was small in a second study – downgraded by 1
With the exception of one study, all other studies did not focus on persons at increased risk of serogroup B meningococcal disease – downgraded by 1
The CI around the effect estimate includes both effect and non-effect – downgraded by 1

Table 2c: Considerations for Vaccine Use: MenB-FHbp (Trumenba®)

Key Factors	Comments
**Balance between benefits and harms**	Vaccine is immunogenic in the short-term for healthy adolescent and adults, and is safe. Low disease burden lowers overall benefits.
**Evidence type for benefits and harms**	
**Use of MenB-FHbp in persons at increased risk**	Benefits: Evidence Type: 3<br>Harms: Evidence Type: 3<br>**Overall: Evidence Type: 3**
**Use of MenB-FHbp during outbreaks**	Benefits: Evidence Type: 2<br>Harms: Evidence Type: 2<br>**Overall: Evidence Type: 2**

Evidence to Decision Table - Pediatric

**PICO 17**	**In pediatric patients with IBD, should age appropriate vaccination vs. no vaccination against meningococcal disease be given?**
**Population**	Pediatric patients with IBD
**Intervention**	Age appropriate vaccination against meningococcal disease
**Comparator**	No age appropriate vaccination against meningococcal disease
**Outcome**	Mortality, VPI (meningococcal infection), SAEs, Immunogenicity

	Judgement	Research evidence	Additional considerations
**Desirable Effects**	**How substantial are the desirable anticipated effects?**<br><br>○ Trivial<br>○ Small<br>○ Moderate<br>○ Large<br><br>○ Varies	See Evidence Profile Tables.<br><br>**Risk of Meningococcal Infection in IBD Patients**<br><br>Literature search did not identify any study on the risk of meningococcal infection in adult or pediatric IBD patients.<br><br>Since 1974, there have been several reports of the association between IBD and hyposplenism based on indirect measurements of splenic function (e.g. heated red cell	

<table>
<tr><td></td><td>○ Don't know</td><td rowspan="2">clearance, pitted erythrocyte counts e.g. Howell-Jolly bodies, differential interference contrast microscopy, splenic size from ultrasound).[1-8] However, the prevalence of functional hyposplenism is uncertain in IBD populations due to paucity of evidence. Of note, functional hyposplenism has also been reported to occur in patients with other GI conditions such as celiac disease, Whipple's disease, idiopathic ulcerative enteritis, tropical sprue, alcoholic liver disease, autoimmune atrophic gastritis, and long term parenteral nutrition, as well as other autoimmune conditions such as systemic lupus erythematosus, thyroid disease, rheumatoid arthritis, sarcoidosis, and amyloidosis. However, the exact mechanisms of hyposplenia in these conditions are poorly understood. Furthermore, the clinical significance of functional hyposplenism is unclear as there are no studies assessing the risks of meningococcal infection in IBD patients.

One case report described a 42-year-old woman who presented with fulminant meningococcal sepsis with hyposplenism that was diagnosed on computed tomography (CT) scan.[9] The hyposplenism was proposed to be possibly related to IBD. Another case report described meningococcal meningoencephalitis after certolizumab pegol treatment in a 51-year-old woman with Crohn's disease.[10]

Due to the designs of these studies (case series/reports), the GRADE rating already started at very low for higher risks of functional hyposplenism in IBD patients compared to the general population. It is even more uncertain whether this finding translates into higher risks of infection by encapsulated organisms such as Streptococcus pneumoniae, Neisseria meningitis, Haemophilus influenzae.

Effectiveness and Safety of Meningococcal Vaccine in Pediatric IBD Patients

There was no RCT or observational studies comparing meningococcal vaccine with placebo or no treatment in pediatric patients with IBD to address this PICO question.

A Cochrane systematic review with inclusion of 8 RCTs (n = 480,068) found polysaccharide serogroup A vaccine to be strongly protective for serogroup A meningococcal meningitis for first year in children over five and adults with summary vaccine efficacy of 95% (95% CI 87-99%).[11] Protection extended into the second and third year after vaccination, but the results did not reach statistical significance.[1] The vaccine was protective in Finnish children aged 3 months to 5 years.[11] The latter was also the only trial that assessed the effect of a booster dose in children under 2 years of age, but lacked power to yield statistically significant results. The vaccine was protective in one- to five-year old children in developing countries (Nigeria and Sudan).[1] The quality of the trials was considered to be high.[11]

The relatively low incidence of meningococcal C disease has so far precluded any RCTs assessing the efficacy of meningococcal serogroup C conjugate vaccines against clinical endpoints. A Cochrane systematic review found meningococcal serogroup C conjugate vaccines to be highly immunogenic in infants for preventing meningococcal C meningitis and septicaemia.[12] The effectiveness of meningococcal serogroup C conjugate vaccine was described only in observational studies.[12] The introduction of meningococcal C vaccines into routine immunization programs in Europe, Canada, and</td><td></td></tr>
<tr><td>Undesirable Effects</td><td>How substantial are the undesirable anticipated effects?

○ Large
○ Moderate
○ Small
○ Trivial

○ Varies
○ Don't know</td><td></td></tr>
</table>

		Australia have proven to be effective, with dramatic reduction in the incidence of meningococcal serogroup C disease.[12] The overall quality of the trials was considered good.[2]<br><br>The WHO has evaluated the evidence for the use of **meningococcal serogroup C conjugate vaccines and quadrivalent meningococcal vaccines** in children using GRADE methodology. There is **moderate** certainty evidence that meningococcal serogroup C conjugate vaccines protects children aged ≥ 2 months to < 5 years and individuals aged ≥ 5 years against meningococcal disease. In addition, there is **moderate** certainty evidence that quadrivalent meningococcal conjugate vaccines protects children aged ≥ 12 months to < 5 years against meningococcal disease, and **low** certainty evidence that quadrivalent meningococcal conjugate vaccines protects individuals aged ≥ 5 years against meningococcal disease. There is **moderate** certainty evidence that meningococcal vaccines are not associated with serious adverse effects.<br><br>The low incidence of meningococcal B disease has also precluded any RCTs assessing the efficacy of **serogroup B meningococcal vaccines** against clinical endpoints. Vaccine effectiveness of serogroup B meningococcal vaccines was therefore inferred based on surrogate outcomes of immunogenicity. CDC considered the quality of evidence **low** for the use of meningococcal serogroup B vaccines in adolescents and young adults (persistence in immunogenicity 11-24 months), and also in persons at increased risk of serogroup B meningococcal disease.<br><br>It is important to note that the evidence was not downgraded due to indirectness related to patient population (general population vs. IBD patients) since there is no reason to suspect that IBD patients are at lower risks for developing meningococcal infection than non-IBD patients. On the contrary, there is reason to suspect that IBD patients may be at higher risks for developing meningococcal infection than non-IBD patients due to the very small number of case reports of functional hyposplenism in these patients, and the use of immunosuppressive medications. There is also no evidence to suggest that the meningococcal vaccines are harmful or less effective in IBD patients. Therefore, the evidence was anchored at the general population since there is no reason to deviate from country-specific immunization guidelines for the general population with protocols based on local epidemiologic, programmatic, resource, policy, disease control objectives and strategies.<br><br>**In summary, there is moderate certainty evidence that meningococcal serogroup C conjugate vaccines are safe and effective in reducing the risk of meningococcal disease in pediatric IBD patients.**<br><br>**There is moderate certainty evidence that quadrivalent meningococcal conjugate vaccines are safe and effective in pediatric IBD patients aged ≥ 12 months to < 5 years, and low certainty evidence that quadrivalent meningococcal conjugate vaccines are safe and effective in pediatric IBD patients aged ≥ 5 years.**<br><br>**There is low certainty evidence that serogroup B meningococcal vaccines are safe and effective in reducing the risk of serogroup B meningococcal disease in pediatric IBD**	

		**patients (adolescents and young adults).**	
**Certainty of evidence**	**What is the overall certainty of the evidence of effects?**<br><br>○ Very low<br>○ Low for meningococcal serogroup B<br>○ Moderate for meningococcal serogroup C quadrivalent meningococcal conjugate vaccines<br>○ High<br><br>○ No included studies		
**Values and Preferences**	**Is there important uncertainty about or variability in how much people value the main outcomes?**<br><br>○ Important uncertainty or variability<br>○ Possibly important uncertainty or variability<br>○ Probably no important uncertainty or variability<br>○ No important uncertainty or variability	Patients likely value patient-important outcomes (mortality, VPI, adverse effects) more than surrogate outcomes (immunogenicity).	
**Balance of effects**	**Does the balance between desirable and undesirable effects favor the intervention or the comparison?**<br><br>○ Favors the comparison<br>○ Probably favors the comparison<br>○ Does not favor either the intervention or the comparison<br>○ Probably favors the intervention<br>○ Favors the intervention<br><br>○ Varies<br>○ Don't know		

<table>
<tr><td>Resources required</td><td>How large are the resource requirements (costs)?

o Large costs
o Moderate costs
o Negligible costs and savings
o Moderate savings
o Large savings

o Varies
o Don't know</td><td>CDC vaccine price list last reviewed/updated: July 1, 2019

<table><tr><th>Brandname</th><th>CDC cost/dose</th><th>Private sector cost/dose</th></tr><tr><td>Menactra® Meningococcal conjugate (ACYW)</td><td>$93.45</td><td>$122.31</td></tr><tr><td>Menveo® Meningococcal conjugate (ACYW)</td><td>$76.02</td><td>$130.75</td></tr><tr><td>Trumemba® Meningococcal B</td><td>$108.95</td><td>$139.52</td></tr><tr><td>Bexsero® Meningococcal B</td><td>$108.53</td><td>$170.75</td></tr></table></td><td></td></tr>
<tr><td>Certainty of Evidence of Required Resources</td><td>What is the certainty of the evidence of resource requirements (costs)?

o Very low
o Low
o Moderate
o High

o No included studies</td><td>The costs of delivering routine immunization services may vary widely across countries and different health system settings. See Immunization Costing Action Network (ICAN) Immunization Delivery Cost Catalogue. http://immunizationeconomics.org/ican-idcc.</td><td></td></tr>
<tr><td>Cost effectiveness</td><td>Does the cost-effectiveness of the intervention favor the intervention or the comparison?

o Favors the comparison
o Probably favors the comparison
o Does not favor either the intervention or the comparison
o Probably favors the intervention
o Favors the intervention

o Varies</td><td>There are no studies that addressed this question specifically in the IBD population.

A cost-effectiveness analysis of conjugate meningococcal vaccination strategies in the US found that routine meningococcal conjugate (ACYW-135) vaccination of US children would reduce the burden of disease in vaccinated cohorts, but at a relatively high societal cost (adolescent vaccination would cost society $633,000 per meningococcal case prevented and $121,000 per life-year saved).[13] Routine vaccination of US 11 year olds would prevent 270 cases and 36 deaths in this cohort over 22 years. The results were sensitive to variations in disease incidence, case-fatality ratio, and cost per vaccination.[13] The cost-effectiveness of toddler vaccination is essentially equivalent to adolescent vaccination, whereas infant vaccination would be much less cost-effective (cost > 3 times per case prevented).[13] However, herd immunity was not assumed. The</td><td></td></tr>
</table>

	○ No included studies	projected cost-effectiveness of adolescent vaccination approaches that of adopted childhood vaccines under conditions of above-average meningococcal disease incidence or at a lower cost per vaccination.<br><br>Cost-effectiveness analyses has not found universal meningococcal serogroup B to be cost effective in infants or college-aged young adults.[14,15]	
**Acceptability**	**Is the intervention acceptable to key stakeholders?**<br><br>○ No<br>○ Probably no<br>○ Probably yes<br>○ Yes<br><br>○ Varies<br>○ Don't know		
**Feasibility**	**Is the intervention feasible to implement?**<br><br>○ No<br>○ Probably no<br>○ Probably yes<br>○ Yes<br><br>○ Varies<br>○ Don't know		

References:

1. Rameh BS, Stevens FM, McCarthy CF. Hyposplenism in inflammatory bowel disease. Ir J Med Sci. 1988 Jan;157(1):8-9.
2. Muller AF, Cornford E, Toghill PJ. Splenic function in inflammatory bowel disease: assessment by differential interference microscopy and splenic ultrasound. Q J Med. 1993 May;86(5):333-40.
3. Ryan FP, Smart RC, Holdsworth CD, Preston FE. Hyposplenism in inflammatory bowel disease. Gut. 1978 Jan;19(1):50-5.
4. Ryan FP, Smart R, Preston FE, Holdsworth CD. Hyposplenism in ulcerative colitis. Lancet. 1974 Aug 10;2(7876):318-20.

5. Palmer KR, Sherriff SB, Holdsworth CD, Ryan FP. Further experience of hyposplenism in inflammatory bowel disease. Q J Med. 1981 Autumn;50(200):463-71.
6. Jewell DP, Berney JJ, Pettit JE. Splenic phagocytic function in patients with inflammatory bowel disease. Pathology. 1981 Oct;13(4):717-23.
7. Di Sabatino A, Rosado MM, Ciccocioppo R, Cazzola P, Morera R, Corazza GR, Carsetti R. Depletion of immunoglobulin M memory B cells is associated with splenic hypofunction in inflammatory bowel disease. Am J Gastroenterol. 2005 Aug;100(8):1788-95.
8. Muller AF, Toghill PJ. Hyposplenism in gastrointestinal disease. Gut. 1995 Feb;36(2):165-7.
9. Shah A, Lettieri CJ. Fulminant meningococcal sepsis in a woman with previously unknown hyposplenism. Medscape J Med. 2008 Feb 13;10(2):36.
10. Majumder S, Kumar A. Meningococcal meningoencephalitis after certolizumab pegol treatment in a patient with Crohn's disease. J Crohns Colitis. 2013 Feb;7(1):e19.
11. Patel M1, Lee CK. Polysaccharide vaccines for preventing serogroup A meningococcal meningitis. Cochrane Database Syst Rev. 2005 Jan 25;(1):CD001093.
12. Conterno LO, Silva Filho CR, Rüggeberg JU, Heath PT. Conjugate vaccines for preventing meningococcal C meningitis and septicaemia. Cochrane Database Syst Rev. 2006 Jul 19;(3):CD001834.
13. Shepard CW, Ortega-Sanchez IR, Scott RD 2nd, Rosenstein NE; ABCs Team. Cost-effectiveness of conjugate meningococcal vaccination strategies in the United States. Pediatrics. 2005 May;115(5):1220-32.
14. Tu HA, Deeks SL, Morris SK, Strifler L, Crowcroft N, Jamieson FB, Kwong JC, Coyte PC, Krahn M, Sander B. Economic evaluation of meningococcal serogroup B childhood vaccination in Ontario, Canada. Vaccine. 2014 Sep 22;32(42):5436-46.
15. Leeds IL, Namasivayam V, Bamogo A, Sankhla P, Thayer WM. Cost Effectiveness of Meningococcal Serogroup B Vaccination in College-Aged Young Adults. Am J Prev Med. 2019 Feb;56(2):196-204.

Conclusion – Pediatric

PICO 17: In pediatric patients with IBD, should age appropriate meningococcal vaccine be given?
Moderate certainty of evidence
Direction – 100%
Strength – 89% Strong

**Type of recommendation**	**Strong recommendation against the intervention**	**Conditional recommendation against the intervention**	**Conditional recommendation for either the intervention or the comparison**	**Conditional recommendation for the intervention**	**Strong recommendation for the intervention**

	○	○	○	○	○

**Recommendation**	**Statement 17: In pediatric patients with IBD, we recommend age-appropriate menincogoccal vaccine be given.**
**Justification**	
**Subgroup considerations**	
**Implementation considerations**	
**Monitoring and evaluation**	• Ongoing monitoring of serious adverse events associated with meningococcal vaccines in IBD patients
**Research priorities**	• Observational studies to determine the risks of meningococcal infection in pediatric IBD patients compared to the general population<br>• RCTs or observational studies to determine the clinical effectiveness and immunogenicity of meningococcal vaccines in pediatric IBD patients with assessment of patient-important outcomes (i.e. meningococcal infection)

Summary – Adults

**PICO 18**	**In adult patients with IBD with a risk factor for invasive meningococcal disease, should vaccination vs. no vaccination against meningococcal disease be given?**
**Population**	Adult patients with IBD with a risk factor for invasive meningococcal disease

**Intervention**	Vaccination against meningococcal disease
**Comparator**	No vaccination against meningococcal disease
**Outcome**	Mortality, VPI (meningococcal infection), SAEs, Immunogenicity

**PICO**	**In adult patients with IBD without a risk factor for invasive meningococcal disease, should vaccination vs. no vaccination against meningococcal disease be given?**
**Population**	Adult patients with IBD **without a risk factor for invasive meningococcal disease**
**Intervention**	Vaccination against meningococcal disease
**Comparator**	No vaccination against meningococcal disease
**Outcome**	Mortality, VPI (meningococcal infection), SAEs, Immunogenicity

There was no RCT or observational studies comparing meningococcal vaccine with placebo or no treatment in adult patients with IBD to address this PICO question.

CDC and NACI do not recommend routine meningococcal vaccines for adults except in individuals with high-risk medical conditions. It is important to note that most studies included pediatric patients (healthy adolescents and young adults) and not persons at increased risks of meningococcal infections. As well, immunogenicity was used as surrogate outcomes of clinical effectiveness. According to WHO, there is **moderate** certainty evidence that meningococcal serogroup C conjugate vaccines protects individuals aged $\geq$ 5 years against meningococcal disease. There is **low** certainty evidence that quadrivalent meningococcal conjugate vaccines protects individuals aged $\geq$ 5 years against meningococcal disease. There is **moderate** certainty evidence that meningococcal vaccines are not associated with serious adverse effects. CDC considered the quality of evidence **low** for the use of meningococcal serogroup B vaccines in persons at increased risk of serogroup B meningococcal disease.

It is important to note that the evidence was not downgraded due to indirectness related to patient population (general population vs. IBD patients) since there is no reason to suspect that IBD patients are at lower risks for developing meningococcal infection than non-IBD patients. On the contrary, there is reason to suspect that IBD patients may be at higher risks for developing meningococcal infection than non-IBD patients due to functional hyposplenism or immunosuppressive medications. There is also no evidence to suggest that the meningococcal vaccines are harmful or less effective in IBD patients. Therefore, there is no reason to deviate from

country-specific immunization guidelines with protocols based on local epidemiologic, programmatic, resource, policy, disease control objectives and strategies.

In summary, there is moderate certainty evidence that meningococcal serogroup C conjugate vaccines are safe and effective in adult IBD patients. There is low certainty evidence that quadrivalent meningococcal conjugate vaccines are safe and effective in adult IBD patients. There is low certainty evidence that meningococcal vaccines are safe and effective in reducing the risk of meningococcal serogroup B disease in adult IBD patients.

Evidence Profile Table – Adults

See Evidence Profile Table - Pediatric

Evidence to Decision Table – Adults

**PICO 18**	**In adult patients with IBD with a risk factor for invasive meningococcal disease, should vaccination vs. no vaccination against meningococcal disease be given?**
**Population**	Adult patients with IBD with a risk factor for invasive meningococcal disease
**Intervention**	Vaccination against meningococcal disease
**Comparator**	No vaccination against meningococcal disease
**Outcome**	Mortality, VPI (meningococcal infection), SAEs, Immunogenicity

**PICO**	**In adult patients with IBD without a risk factor for invasive meningococcal disease, should vaccination vs. no vaccination against meningococcal disease be given?**
**Population**	Adult patients with IBD **without a risk factor for invasive meningococcal disease**
**Intervention**	Vaccination against meningococcal disease
**Comparator**	No vaccination against meningococcal disease

Outcome	Mortality, VPI (meningococcal infection), SAEs, Immunogenicity

	Judgement	Research evidence	Additional considerations
**Desirable Effects**	**How substantial are the desirable anticipated effects?**<br><br>o Trivial without RF<br>o Small<br>o Moderate with RF<br>o Large<br><br>o Varies<br>o Don't know	See Evidence Profile Tables.<br><br>**Risk of Meningococcal Infection in IBD Patients**<br><br>Literature search did not identify any study on the risk of meningococcal infection in adult or pediatric IBD patients.<br><br>Since 1974, there have been several reports of the association between IBD and hyposplenism based on indirect measurements of splenic function (e.g. heated red cell clearance, pitted erythrocyte counts e.g. Howell-Jolly bodies, differential interference contrast microscopy, splenic size from ultrasound).[1-8] However, the prevalence of functional hyposplenism is uncertain in IBD populations due to paucity of evidence. Of note, functional hyposplenism has also been reported to occur in patients with other GI conditions such as celiac disease, Whipple's disease, idiopathic ulcerative enteritis, tropical sprue, alcoholic liver disease, autoimmune atrophic gastritis, and long-term parenteral nutrition, as well as other autoimmune conditions such as systemic lupus erythematosus, thyroid disease, rheumatoid arthritis, sarcoidosis, and amyloidosis. However, the exact mechanisms of hysplenia in these conditions are poorly understood. Furthermore, the clinical significance of functional hyposplenism is unclear as there are no studies assessing the risks of meningococcal infection in IBD patients.<br><br>One case report described a 42-year-old woman who presented with fulminant meningococcal sepsis with hyposplenism that was diagnosed on computed tomography (CT) scan.[9] The hyposplenism was proposed to be possibly related to IBD. Another case report described meningococcal meningoencephalitis after certolizumab pegol treatment in a 51-year-old woman with Crohn's disease.[10]<br><br>Due to the designs of these studies (case series/reports), the GRADE rating already started at very low for higher risks of functional hyposplenism in IBD patients compared to the general population. It is even more uncertain whether this finding translates into higher risks of infection by encapsulated organisms such as Streptococcus pneumoniae, Neisseria meningitis, Haemophilus influenzae.<br><br>**Effectiveness and Safety of Meningococcal Vaccine in Adult IBD Patients**<br><br>There was no RCT or observational studies comparing meningococcal vaccine with placebo or no treatment in adult patients with IBD to address this PICO question.<br><br>CDC and NACI do not recommend routine meningococcal vaccines for adults except in individuals with high-risk medical conditions. It is important to note that most studies	Elaborate in the text what high risk conditions are (IBD is not considered a high-risk condition). There is no clinical evidence to suggest that it is thus far.
**Undesirable Effects**	**How substantial are the undesirable anticipated effects?**<br><br>o Large<br>o Moderate<br>o Small<br>o Trivial<br><br>o Varies<br>o Don't know		

		included pediatric patients (healthy adolescents and young adults) and not persons at increased risks of meningococcal infections. As well, immunogenicity was used as surrogate outcomes of clinical effectiveness. According to WHO, there is **moderate** certainty evidence that meningococcal serogroup C conjugate vaccines protects individuals aged > 5 years against meningococcal disease. There is **low** certainty evidence that quadrivalent meningococcal conjugate vaccines protects individuals aged > 5 years against meningococcal disease. There is **moderate** certainty evidence that meningococcal vaccines are not associated with serious adverse effects. CDC considered the quality of evidence **low** for the use of meningococcal serogroup B vaccines in persons at increased risk of serogroup B meningococcal disease.<br><br>It is important to note that the evidence was not downgraded due to indirectness related to patient population (general population vs. IBD patients) since there is no reason to suspect that IBD patients are at lower risks for developing meningococcal infection than non-IBD patients. On the contrary, there is reason to suspect that IBD patients may be at higher risks for developing meningococcal infection than non-IBD patients due to functional hyposplenism or immunosuppressive medications. There is also no evidence to suggest that the meningococcal vaccines are harmful or less effective in IBD patients. Therefore, there is no reason to deviate from country-specific immunization guidelines with protocols based on local epidemiologic, programmatic, resource, policy, disease control objectives and strategies.<br><br>**In summary, there is moderate certainty evidence that meningococcal serogroup C conjugate vaccines are safe and effective in adult IBD patients. There is low certainty evidence that quadrivalent meningococcal conjugate vaccines are safe and effective in adult IBD patients. There is low certainty evidence that meningococcal serogroup B vaccines are safe and effective in reducing the risk of serogroup B meningococcal disease in adult IBD patients.**	
Certainty of evidence	**What is the overall certainty of the evidence of effects?**<br><br>○ Very low<br>○ Low for meningococcal serogroup B, quadrivalent<br>○ Moderate meningococcal serogroup C conjugate vaccines<br>○ High<br><br>○ No included studies		
Values and Preferences	**Is there important uncertainty about or variability in how much people value the main outcomes?**<br><br>○ Important uncertainty or variability<br>○ Possibly important uncertainty or variability	Patients likely value patient-important outcomes (mortality, VPI, adverse effects) more than surrogate outcomes (immunogenicity).	

	○ Probably no important uncertainty or variability<br>○ No important uncertainty or variability		
**Balance of effects**	**Does the balance between desirable and undesirable effects favor the intervention or the comparison?**<br><br>○ Favors the comparison<br>○ Probably favors the comparison<br>○ Does not favor either the intervention or the comparison No RF<br>○ Probably favors the intervention with RF<br>○ Favors the intervention<br><br>○ Varies<br>○ Don't know		
**Resources required**	**How large are the resource requirements (costs)?**<br><br>○ Large costs<br>○ Moderate costs without RF<br>○ Negligible costs and savings with RF<br>○ Moderate savings<br>○ Large savings<br><br>○ Varies<br>○ Don't know	CDC vaccine price list last reviewed/updated: July 1, 2019 (see table below)	

CDC vaccine price list last reviewed/updated: July 1, 2019

Brandname	CDC cost/dose	Private sector cost/dose
Menactra® Meningococcal conjugate (ACYW)	$93.45	$122.31
Menveo® Meningococcal conjugate (ACYW)	$76.02	$130.75
Trumemba® Meningococcal B	$108.95	$139.52
Bexsero® Meningococcal B	$108.53	$170.75

**Certainty of Evidence of Required Resources**	**What is the certainty of the evidence of resource requirements (costs)?**<br><br>o Very low<br>o Low<br>o Moderate<br>o High<br><br>o No included studies	The costs of delivering routine immunization services may vary widely across countries and different health system settings. See Immunization Costing Action Network (ICAN) Immunization Delivery Cost Catalogue. http://immunizationeconomics.org/ican-idcc.	
**Cost effectiveness**	**Does the cost-effectiveness of the intervention favor the intervention or the comparison?**<br><br>o Favors the comparison without RF<br>o Probably favors the comparison<br>o Does not favor either the intervention or the comparison<br>o Probably favors the intervention with RF<br>o Favors the intervention<br><br>o Varies<br>o No included studies	There is a paucity of cost-effectiveness analysis of meningococcal vaccination in adult patients (or in those at increased risks for infection).<br><br>In one cost effectiveness analysis, meningococcal vaccination during an outbreak of invasive meningococcal disease among men who have sex with men with or without HIV infection was found to be cost-effective in 97% of simulations with herd immunity (at a cost-effectiveness threshold of $100,000/QALY).[11] Variables that exerted the greatest influence on results were the magnitude of herd immunity, case fatality ratio, and incidence of invasive meningococcal disease.<br><br>A cost-effectiveness analysis found universal meningococcal serogroup B not cost effective in college-aged young adults.[12]	
**Acceptability**	**Is the intervention acceptable to key stakeholders?**<br><br>o No<br>o Probably no<br>o Probably yes<br>o Yes<br><br>o Varies<br>o Don't know		
**Feasibility**	**Is the intervention feasible to implement?**<br><br>o No<br>o Probably no<br>o Probably yes<br>o Yes<br><br>o Varies		

	○ Don't know		

References:

1. Rameh BS, Stevens FM, McCarthy CF. Hyposplenism in inflammatory bowel disease. Ir J Med Sci. 1988 Jan;157(1):8-9.
2. Muller AF, Cornford E, Toghill PJ. Splenic function in inflammatory bowel disease: assessment by differential interference microscopy and splenic ultrasound. Q J Med. 1993 May;86(5):333-40.
3. Ryan FP, Smart RC, Holdsworth CD, Preston FE. Hyposplenism in inflammatory bowel disease. Gut. 1978 Jan;19(1):50-5.
4. Ryan FP, Smart R, Preston FE, Holdsworth CD. Hyposplenism in ulcerative colitis. Lancet. 1974 Aug 10;2(7876):318-20.
5. Palmer KR, Sherriff SB, Holdsworth CD, Ryan FP. Further experience of hyposplenism in inflammatory bowel disease. Q J Med. 1981 Autumn;50(200):463-71.
6. Jewell DP, Berney JJ, Pettit JE. Splenic phagocytic function in patients with inflammatory bowel disease. Pathology. 1981 Oct;13(4):717-23.
7. Di Sabatino A, Rosado MM, Ciccocioppo R, Cazzola P, Morera R, Corazza GR, Carsetti R. Depletion of immunoglobulin M memory B cells is associated with splenic hypofunction in inflammatory bowel disease. Am J Gastroenterol. 2005 Aug;100(8):1788-95.
8. Muller AF, Toghill PJ. Hyposplenism in gastrointestinal disease. Gut. 1995 Feb;36(2):165-7.
9. Shah A, Lettieri CJ. Fulminant meningococcal sepsis in a woman with previously unknown hyposplenism. Medscape J Med. 2008 Feb 13;10(2):36.
10. Majumder S, Kumar A. Meningococcal meningoencephalitis after certolizumab pegol treatment in a patient with Crohn's disease. J Crohns Colitis. 2013 Feb;7(1):e19.
11. Simon MS, Weiss D, Geevarughese A, Kratz MM, Cutler B, Gulick RM, Zucker JR, Varma JK, Schackman BR. Cost-Effectiveness of Meningococcal Vaccination Among Men Who Have Sex With Men in New York City. J Acquir Immune Defic Syndr. 2016 Feb 1;71(2):146-54.
12. Leeds IL, Namasivayam V, Bamogo A, Sankhla P, Thayer WM. Cost Effectiveness of Meningococcal Serogroup B Vaccination in College-Aged Young Adults. Am J Prev Med. 2019 Feb;56(2):196-204.

Conclusion – Adults

PICO 18: In adult patients with IBD with a risk factor for invasive meningococcal disease, should meningococcal vaccine be given?
Moderate certainty of evidence
Direction – Yes (100%)
Strength – Strong (100%)

Type of recommendation	Strong recommendation against the intervention	Conditional recommendation against the intervention	Conditional recommendation for either the intervention or the comparison	Conditional recommendation for the intervention	Strong recommendation for the intervention
	○	○	○	○	○

**Recommendation**	**Statement 18: In adult patients with IBD with a risk factor for invasive meningococcal disease, we recommend meningococcal vaccines be given.**
**Justification**	
**Subgroup considerations**	
**Implementation considerations**	
**Monitoring and evaluation**	• Ongoing monitoring of serious adverse events associated with meningococcal vaccines in IBD patients
**Research priorities**	• Observational studies to determine the risks of meningococcal infection in adult IBD patients compared to the general population

PICO: In adult patients with IBD without a risk factor for invasive meningococcal disease, should meningococcal vaccine be given?
Moderate certainty of evidencce
Direction – Uncertain (78%), No (22%)

Type of recommendation	Strong recommendation against the intervention	Conditional recommendation against the intervention	Conditional recommendation for either the intervention or the comparison	Conditional recommendation for the intervention	Strong recommendation for the intervention
	○	○	○	○	○
**Recommendation**	**No recommendation. In adult patients with IBD without a risk factor for invasive meningococcal disease, the consensus group could not make a recommendation for or against giving pneumococcal vaccines.**				
**Justification**					
**Subgroup considerations**					
**Implementation considerations**					
**Monitoring and evaluation**	• Ongoing monitoring of serious adverse events associated with meningococcal vaccines in IBD patients				
**Research priorities**	• Observational studies to determine the risks of meningococcal infection in adult IBD patients compared to the general population				

Diphtheria, Tetanus, Pertussis

Background

Diphtheria is caused by exotoxin-producing strains of the bacterium *Corynebacterium diphtheriae*. The organism produces a toxin that inhibits cellular protein synthesis which results in local tissue destruction and pseudomembrane formation.[1] Most complications are attributable to the toxin which can affect almost any mucous membrane in the body. The most frequent complications are myocarditis and neuritis. Whilst infection is rare in Canada it remains endemic in many developing countries and case fatality rates are 5-10%.[1] Inadequately immunized or unimmunized travellers to areas with endemic diphtheria are at higher risk of acquiring disease.

Tetanus is caused by a neurotoxin produced by the bacterium *Clostridium tetani*. It is characterized by rigidity and convulsive spasms of skeletal muscles, usually starting in the jaw and neck before becoming generalized.[1] *Clostridium tetani* usually enters the body through a wound. In the presence of anaerobic conditions, the spores germinate, and the toxins produced are disseminated via blood and lymphatics. Case fatality is about 11% and those at most risk include persons 60 years of age and older (18%) and unvaccinated persons (22%). In about 20% of tetanus deaths, no obvious pathology is identified, and death is attributed to the direct effects of tetanus toxin.[1,2]

Pertussis, or whooping cough, is a highly communicable respiratory illness caused by the bacterium *Bordatella pertussis*. Primarily a toxin mediated disease, Pertussis causes paralysis of respiratory epithelium which interferes with clearance of pulmonary secretions. The most common complication of Pertussis is secondary bacterial pneumonia, also the cause of most pertussis related deaths.[1] Its severity is greatest among infants who are too young to be protected by a complete vaccine series.[1,2] In Canada, pertussis vaccine is only available as an acellular preparation in a combination vaccine. Acellular pertussis vaccines are subunit vaccines that contain purified, inactivated components of B. pertussis cells.[1]

Nine combination vaccine preparations are available in Canada with the most common being the DTaP-IPV (inactivated polio vaccine)-Hib vaccine (INFANRIX®-IPV/Hib (GlaxoSmithKline); INFANRIX®-IPV/Hib (Sanofi Pasteur Ltd.)) which is recommended by both NACI and the CDC ACIP at 2, 4, 6 and 12 to 23 months of age (generally given at 18 months of age).[2,3] A reduced Pertussis booster (Tdap) vaccine should be administered to adolescents at 14 to 16 years of age as the first 10-year booster dose, to unimmunized adults, and to pregnant women during each pregnancy regardless of previous Tdap exposure. After receipt of Tdap, adolescents and adults are recommended to receive a booster tetanus and diphtheria toxoids (Td) vaccine every 10 years to assure ongoing protection against tetanus and diphtheria.[2,3]

References:

1. Centers for Disease Control and Prevention. Epidemiology and Prevention of Vaccine-Preventable Diseases. Hamborsky J, Kroger A, Wolfe S, eds. 13th ed. Washington D.C. Public Health Foundation, 2015.
2. Public Health Agency of Canada. Accessed online Sept 28 2019 at https://www.canada.ca/en/public-health/services/publications/healthy-living/canadian-immunization-guide-part-4-active-vaccines/page-4-diphtheria-toxoid.html
3. Liang JL, Tiwari T, Moro P, et al. Prevention of Pertussis, Tetanus, and Diphtheria with Vaccines in the United States: Recommendations of the Advisory Committee on Immunization Practices (ACIP). *MMWR Recomm Rep* 2018;67(No. RR-2):1–44.

Risk of Diphtheria, Tetanus and Pertussis Infection in IBD patients

PICO: What is the risk of tetanus, diphtheria and pertussis infection in people with IBD compared to people without IBD?

Summary – Adults and Pediatric

The literature search did not identify any study on the risk of tetanus, diphtheria or pertussis infection in adult or pediatric IBD patients.

Effectiveness and Safety of Diphtheria, Tetanus and Pertussis Vaccine in IBD Patients

Summary – Pediatric and Adults

**PICO 19**	**In pediatric patients with IBD, should vaccination vs. no vaccination against tetanus, diphtheria, and pertussis be given?**
**Population**	Pediatric patients with IBD
**Intervention**	Vaccination against tetanus, diphtheria, and pertussis
**Comparator**	No vaccination against tetanus, diphtheria, and pertussis
**Outcome**	Mortality, VPI (tetanus, diphtheria, pertussis), SAEs, Immunogenicity

**PICO 20**	**In adult patients with IBD, should vaccination vs. no vaccination against**

	tetanus, diphtheria, and pertussis with TdAP/Td be given?
**Population**	Adult patients with IBD
**Intervention**	Vaccination against tetanus, diphtheria, and pertussis with TdAP/Td
**Comparator**	No vaccination against tetanus, diphtheria, and pertussis with TdAP/Td
**Outcome**	Mortality, VPI (tetanus, diphtheria, pertussis), SAEs, Immunogenicity

There is no RCT comparing diphtheria, tetanus and pertussis vaccines with placebo in patients with IBD to address this PICO question. However, there are immunogenicity studies supporting the safety and effectiveness of these vaccines in both pediatric and adult IBD populatons. As observational studies have not shown any significant difference in immunogencity of these vaccines between pediatric IBD populatoins vs. adult IBD populations vs. healthy controls, the results were presented together in the evidence profile table.

Effectiveness of DTap vaccines and Tdap/Td/tetanus booster
The WHO and CDC have assessed the evidence for effectiveness of diphtheria, tetanus and acellular pertussis vaccines in healthy children and adults. For pertussis, a Cochrane systematic review of 6 RCTs found the efficacy of multi-component vaccines varied from 84-85% in preventing pertussis.[1] Although no RCT of the efficacy of diphtheria or tetanus toxoid in preventing disease has ever been conducted, strong evidence from observational studies supports the effectiveness of vaccination.[2-11] The WHO rated the certainty of evidence as **high.** When the evidence is applied to IBD patient populations, the evidence was not downgraded for effectiveness as immunogenicity studies showed that there was no significant difference in seroresponse or seroprotection between IBD patients vs. healthy controls. It is important to note that there are no established guidelines or standard correlates of protection against diphtheria, tetanus and pertussis. The true correlation between antibody level and clinical protection has not been determined. Despite the fact that immunogenicity studies in IBD populations have used different definitions of seroresponse or seroprotection, there was no significant difference found between IBD patients and healthy controls.[12-17] One cross sectional study in adults suggests that IBD patients may have lower sustained diphtheria and pertussis antibody concentrations compard to healthy controls who had received an adult Tdap booster (median 60 months since immunization), and those on anti-TNF monotherapy or combination had lower antibody concentrations compared to those on thiopurine monotherapy.[17] However, the clinical significance of these findings is uncertain given the anamnestic response was not assessed in these patients.[17] **The evidence was therefore anchored to the general population. In summary, there is <u>high</u> certainty evidence that DTap vaccines are effective in pediatric patients with IBD; and Td, Tdap and tetanus boosters are effective in adult patients with IBD. Immunosuppressive medications are not associated with a reduced serological response to these vaccines.**

Safety of DTap vaccines and Tdap/Td/tetanus booster

The WHO and CDC have also assessed the evidence of safety of diphtheria, tetanus and acellular pertussis vaccine in healthy children and adults. For pertussis, a Cochrane systematic review of 52 RCTs found no significant risk of serious adverse events following administration of acellular pertussis vaccines.[1] A safety analysis using the data from the Vaccine Adverse Event Reporting System (VAERS) found no serious adverse events related to the DTap vaccines and no increased risk for neurologic disorders.[18] The WHO rated the certainty of evidence as high. When the evidence is applied to IBD patient populations, the evidence was downgraded 1 level for imprecision given that only 1 study had assessed the safety of pertussis vaccine in pediatric IBD population and 2 studies had assessed the safety of Tdap and Td booster in adult IBD populations. In summary, **there is moderate certainty evidence that DTap are safe in pediatric patients with IBD, and Td / Tdap / tetanus booster are safe in adult patients with IBD.**

Overall, the evidence is anchored to the critical outcome of safety (adverse events). There is moderate certainty evidence that diptheria, tetanus and pertussis vaccines are safe and effective in pediatric and adult patients with IBD.

Risk of Bias Table – Adults and Pediatric

Before-After (Pre-Post) Studies									
Study	Was there a concurrent comparator group that did not receive the intervention	If a concurrent comparator group was used, was it similar to the intervention group (or adequately adjusted) for prognostic factors	If no concurrent comparator group was used		Outcome detection methods valid and similar among compared groups / periods	Incomplete outcome data assessed	Selective outcome reporting	Other bias	Comments
			If each participant served as his/her own control (assessed before vs. after the intervention), are there compelling arguments	If two different consecutive cohorts of participants were assessed (before vs. after implementation of the intervention), are there (a) compelling arguments that					

			that the outcome was not influenced by historic events / underlying secular trends	the outcome was not influenced by historic events / underlying secular trends and (b) evidence that the two groups were similar (or adequately adjusted) for prognostic factors					
Banaszkiewic 2017 (Poland) Pediatric IBD	No	OK<br><br>Adequate vaccine response was statistically lower in patients with remission compared with those with mild disease (P = 0.024), but no difference in vaccine response rates between mild vs. moderate disease	OK	NA	No established guidelines or standard correlates of protection.<br><br>An adequate vaccine response, defined as post vaccination specific IgG antibody concentration >11 Virotech units	OK	OK	Possible selection bias. Patients attending a tertiary referral center may differ systematically from other patients. Patients who agreed to participate in a study where serologic assays were measured may be different than patients who did not agree to	• Multicenter, open labelled prospective case control study in 6 pediatric university hospitals in Poland between 2013 and 2015.<br>• Cases: 109 IBD patients (age 11 and 18) with similar disease activity stratified into 3 groups (no IS, thiopurine, thiopurines and anti-TNF)<br>• Controls: 29 healthy volunteers<br>• All subjects were previously immunized

		activity (P = 0.56)						participate.	against pertussis<br>• All subjects received one dose of **DtP booster vaccine Boostrix**<br>• Serum samples were collected before vaccination and 6 to 8 weeks after vaccination.<br>• An adequate vaccine response **to pertussis**, defined as post vaccination specific IgG antibody concentration >11 Virotech units<br>• **No significant difference in adequate vaccine response rates among IBD patients 88%, 91%, 90%, 72% (p=0.11) vs. healthy controls**<br>• **No differences in adequate vaccine response rates between IBD patients on and not on IS**

									• **No serious adverse events including disease flare**
Dezfoli 2015 (US) Adult IBD	No	Did not adjust for disease activity or severity or duration.	OK	NA	No established guidelines or standard correlates of protection.<br><br>Booster response rates defined as tetanus titer > 0.4 IU/mL or greater than 4-fold increase if the baseline was > 0.1 IU/mL; pertussis titer > 20 endotoxin units EU/mL or a 4-fold increase if the baseline between 5-20 EU/mL and a 2-fold rise in those with a baseline titer > 20 EU/mL.	16.7% did not complete the study because of lack of follow-up for the post-vaccination serology.	OK	Possible selection bias. Patients attending a tertiary referral center may differ systematically from other patients. Patients who agreed to participate in a study where serologic assays were measured may be different than patients who did not agree to participate.	• Prospective case control study<br>• Cases: 76 Adult IBD subjects stratified into 4 groups (no treatment or 5-ASA alone; biologic monotherapy; IM; combined IM and biologic; healthy control<br>• Controls: 8 aged matched healthy controls<br>• All immunized with the Boostrix **Tdap booster vaccine**<br>• Serum antibody levels against tetanus toxoid (TT), pertussis toxoid (PT), and filamentous hemagglutinin (FHA) were drawn just before and approximately 4 weeks after vaccination.

									• **No statistically significant difference in booster response rates for tetanus and pertussis across all groups, but lower response rates in patients on IM and combination therapy (ns).**<br>• Booster tetanus vaccine was immunogenic with a response rate for TT between 27-56% vs 63% in healthy controls<br>• Booster pertussis vaccine was immunogenic with a response rate for PT between 45-72% and for FHA between 64-86% vs. 75% in healthy controls<br>• **No serious adverse events including flare of IBD activity.**

Dotan 2012 (US and Israel) Adult IBD	No	OK	OK	OK	No established guidelines or standard correlates of protection.<br><br>Response to tetanus was defined as > 2-fold increase in antibody titer over baseline	19% (10/53) either withdrew due to thiopurine side effects or were lost to follow-up. Reported outcomes only on 36% (19/53) who were started on thiopurine. Unclear if the other patients received vaccine or not	OK	Possible selection bias. Patients attending a tertiary referral center may differ systematically from other patients. Patients who agreed to participate in a study where serologic assays were measured may be different than patients who did not agree to participate.	• Prospective cohort study of 53 IBD patients (35 CD, 15 UC, 3 IC) who were starting on thiopurine treatment<br>• Patients were administered **diphtheria and tetanus vaccine** just before initiating thiopurine therapy<br>• Post-therapy average 6-MP dose: 1.05 +/- 0.30mg/kg<br>• Response to tetanus was defined as > 2-fold increase in antibody titer over baseline<br>• **73% (27/37) had > 2-fold rise in antibody titer**<br>• No vaccine induced disease exacerbation
Nielsen 2001 (Denmark) Adult IBD	No	OK<br><br>Included inactive disease	OK	NA	No established guidelines or standard correlates of protection.	OK	OK	Possible selection bias. Patients attending a tertiary	• Prospective case control study<br>• Cases: 10 patients with inactive CD (CRP

		patients on no medications.			Anti-tetanus antibody concentrations were determined using ELISA method.			referral center may differ systematically from other patients. Patients who agreed to participate in a study where serologic assays were measured may be different than patients who did not agree to participate.	< 10mg/L) and no inflammatory medication for ≥ 2 mos<br>• Controls: 12 age- and gender-matched healthy volunteers<br>• All had received tetanus vaccine previously with anti-tetanus titer < 0.1 IU/mL<br>• All inoculated with 1mL of **tetanus toxoid booster vaccine**<br>• Antibody titers measured at baseline, and after 7, 14, and 28 days<br>• **The anti-tetanus antibody levels were similar in patients and healthy volunteers 28 days after inoculation**, but increased more rapidly in healthy volunteers than in patients at day 7 and day 14 after inoculation.

IM - immunomodulator

Cohort studies							
**Study**	**Valid methods to ascertain exposure**	**Prognostic factors (other than exposure of interest) similar among cohorts – or cohorts were adjusted adequately for confounders**	**Demonstration that outcome of interest was not present at the start of the study**	**Outcome detection methods valid and similar among cohorts**	**Follow-up complete and similar among cohorts**	**Free of other bias**	**Comments**
deBruyn 2018 (Canada) Pediatric IBD	Vaccination records and baseline serology were used to determine immunity against vaccine preventable diseases including Varicella.	IBD subtype, current immunosuppressive medication use, age at diagnosis, and age at serum collection were adjusted for in a multivariate analysis.<br><br>Disease activity at time of vaccination, duration of disease and nutritional status were not accounted for.	OK	No established guidelines or standard correlates of protection.<br><br>Serologic protection was defined for qualitative assays as positive detection of tetanus IgG > 0.1 IU/mL; diphtheria IgG $\geq$ 0.01 IU/mL	OK	Possible selection bias. Patients attending a tertiary referral center may differ systematically from other patients. Patients who agreed to participate in a study where serologic assays were measured may be different than patients who did not agree to participate.	• Cross sectional study in children examining the serologic status of childhood vaccinatable diseases<br>• 156 children with IBD at a Canadian tertiary referral IBD unit.<br>• **Among 74 subjects who received the complete diphtheria and tetanus vaccine series, serologic protection was present for 85.1% for diphtheria and 90.5% for tetanus.**<br>• **Current IS therapy, IBD type, age at enrollment, and age at diagnosis were not associated with serologic protection.**<br>• Cannot distinguish between waning titers vs. primary vaccination failure

Caldera 2018 (US) Adult IBD	Tdap administration confirmed in the Wisconsin Immunization Registry	Disease activity at time of vaccination, duration of disease and nutritional status were not accounted for.	OK	No established guidelines or standard correlates of protection.<br><br>Seroprotection was defined as tetanus or diphtheria antibody ≥ 0.10 IU/mL. Tetanus and diphtheria IgG antibody titers were measured using ELISA. Pertussis toxin IgG, FHA, PRN IgG were measured using ELISA and adjusted to the WHO pertussis standard 1st IS 06/140.	OK	Possible selection bias. Patients attending a tertiary referral center may differ systematically from other patients. Patients who agreed to participate in a study where serologic assays were measured may be different than patients who did not agree to participate	• Cross sectional study evaluating responses to Tdap vaccine among 90 IBD patients stratified into 3 groups (thiopurine; biologic monotherapy; combination) vs 20 healthy controls<br>• All patients received Tdap dose > 4 weeks prior to entering study and in the 10-year booster interval. No differences in time since Tdap.<br>• **Pertussis antibody concentration were significantly lower in IBD patients vs. healthy controls (0.021) and those on anti-TNF (monotherapy or combination) had lower antibody concentrations compared to those on thiopurine monotherap**y (P = 0.028)<br>• **Diphtheria Pertactin antibody concentrations were lower in IBD patients (P < 0.001), and those on anti-TNF (monotherapy or combination) had lower antibody concentration compared to thiopurine monotherapy group (P < 0.001).** No difference for Pertusus toxin or FHA.<br>• **No difference in tetanus antibody concentration**s were found between IBD vs. healthy controls

							• Cannot distinguish between waning titers vs. primary vaccination failure

Evidence Profile Table – Pediatric and Adults

Certainty Assessment								Summary of Findings	
Studies	Risk of bias	Inconsistency	Indirectness	Imprecision	Other considerations	Certainty of Evidence	Overall Certainty of evidence	Study Event Rates	Relative Effect (95% CI)
**VPI (Diphtheria, Tetanus, Pertussis) - CRITICAL**							⊕⊕⊕⊖ MODERATE		
**Pertussis**<br>1 SR of 6 RCTs[1]<br>**Healthy Children**<br><br>**Diphtheria**<br>8 Observational studies[2-9]<br>**Healthy Children**<br><br>**Tetanus**<br>2 Observational studies[10,11]<br>**Healthy Adults**<br><br>*Adapted from WHO and CDC Review of Evidence*	Not serious	Not serious	Not serious[a]	Not Serious	Upgraded by 2 levels as evidence from RCT and observational studies of vaccine effectiveness of 80% or higher across multiple studies (high magnitude).	⊕⊕⊕⊕ HIGH		• **Pertusus**: efficacy of multi-component vaccines varied from 84-85% in preventing pertussis (> 21 consecutive days of paroxysmal cough with confirmation of *B. pertussis* infection by cuture, appropriate serology or contact with a household member who has culture-confirmed pertusus)[1]<br><br>• **Diphtheria**: effectiveness of diphtheria toxoid is high (96.9% with CI 94.3-98.4%),[9] although not 100%<br><br>• **Tetanus**: Incidence of tetanus among US army personnel declined from 13.4 / 100,000 during WWI (when personnel was unvaccinated) to 0.44/100,000 during WWII (when personnel routinely were vaccinated).[10] Similar observations were made among British army personnel during the same periods.[11] Effetiveness of tetanus toxoid is very high, although not 100%.	
**Immunogenicity (Diphtheria, Tetanus, Pertussis) - IMPORTANT**									
**Pertussis**<br>1 Before-After study[12]<br><br>**Pediatric IBD populations**	Serious[b]	Not serious	Serious[c]	Serious[d]	None	⊕⊖⊖⊖ VERY LOW		• No significant difference in adequate vaccine response rates to **Pertussis** among IBD patients (88-91%) vs. healthy controls (72%)<br>• No association between use of IS and vaccine response	
**Diphtheria, Tetanus**<br>1 Cross-sectional study[13]<br><br>**Pediatric IBD populations**	Serious[e]	Not serious	Serious[f]	Serious[d]	None	⊕⊖⊖⊖ VERY LOW		• Serologic protection 85.1% for **diphtheria** and 90.5% for **tetanus**.<br>• Current IS therapy, IBD type, age at enrollment, and age at diagnosis were not associated with serologic protection.<br>• Cannot distinguish between waning titers vs. primary vaccination	

								failure.
**Tdap, Td, tetanus booster**<br>3 Before-after studies[14-16]<br><br>**Adult IBD populations**	Serious[b]	Not serious	Serious[g]	Serious[d]	None	⊕⊖⊖⊖<br>VERY LOW		• No significant difference in adequate vaccine response rates among IBD patients vs. healthy controls<br>• No association between use of IS and vaccine response
**DTap**<br>1 cross-sectional study[17]<br><br>**Adult IBD populations**	Serious[e]	Not serious	Serious[h]	Serious[d]	None	⊕⊖⊖⊖<br>VERY LOW		• Lower **pertussis** and **diphtheria** concentrations in IBD patients vs. healthy controls, and those on anti-TNF (monotherapy or combination) had lower antibody concentrations compared to those on thiopurine monotherapy.<br>• No difference in **tetanus** concentrations in IBD patients vs. healthy controls.<br>• Uncertain clinical importance of waning titers (anamnestic response was not assessed)
**Adverse events - CRITICAL**								
**Pertussis**<br>1 SR of 52 RCTs[1]<br>**Healthy Children**<br><br>**DTap vaccines**<br>1 Observational study[18]<br>**Healthy Children**<br><br>*Adapted from WHO and CDC Review of Evidence*	Not serious	Not serious	Serious[i]	Not Serious	None	⊕⊕⊕⊖<br>MODERATE		• No significant risk of serious adverse events following administration of acellular **pertussis** vaccines[1]<br><br>• DTap studies among the Vaccine Adverse Event Reporting System (VAERsS) found no serious adverse events related to the **DTap** vaccine and no increased risk for neurologic disorders[18]
**Pertussis**<br>1 Observational study[12]<br><br>**Pediatric IBD populations**	Serious[b]	Not serious	Not serious	Serious[d]	None	⊕⊖⊖⊖<br>VERY LOW		• No serious adverse events including flare of disease with the **pertussis** vaccine
**Tdap, Td booster**<br>2 Before-after studies[14-16]<br><br>**Adult IBD populations**	Serious[b]	Not serious	Not serious	Serious[d]	None	⊕⊖⊖⊖<br>VERY LOW		• No serious adverse events including flare of disease with the **Tdap or Td booster**

IS – immunosuppressive therapy

Footnotes:

a. Not downgraded for indirectness as effectiveness of the vaccines is supported by immunogenicity studies in IBD patient populations.
b. Downgraded for study limitations. Possible selection bias. Patients attending a tertiary referral center may differ systematically from other patients. Patients who agreed to participate in a study where serologic assays were measured may be different than patients who did not agree to participate.
c. Downgraded for indirectness. Surrogate outcomes were used. No established guidelines or standard correlates of protection for DTap. An adequate vaccine response to Pertussis vaccine was defined as post vaccination specific IgG antibody concentration >11 Virotech units.

d. Downgraded for imprecision. Small sample size.
e. Downgraded for study limitations. Possible selection bias. Patients attending a tertiary referral center may differ systematically from other patients. Patients who agreed to participate in a study where serologic assays were measured may be different than patients who did not agree to participate. Residual confounding factors: disease activity at time of vaccination, duration of disease and nutritional status were not accounted for.
f. Downgraded for indirectness. Surrogate outcomes were used. No established guidelines or standard correlates of protection for DTap. Serologic protection was defined for qualitative assays as positive detection of tetanus IgG > 0.1 IU/mL; diphtheria IgG $\geq$ 0.01 IU/mL.
g. Downgraded for indirectness. Surrogate outcomes were used. Various definitions were used for seroprotection for different components of the vaccine.
h. Downgraded for indirectness. Surrogate outcomes were used. No established guidelines or standard correlates of protection for DTap. Seroprotection was defined as tetanus or diphtheria antibody $\geq$ 0.10 IU/mL. Tetanus and diphtheria IgG antibody titers were measured using ELISA. Pertussis toxin IgG, FHA, PRN IgG were measured using ELISA and adjusted to the WHO pertussis standard 1st IS 06/140.
i. Downgraded for indirectness. No serious adverse events with pertussis vaccines was noted in the 1 study on pediatric IBD patients. No serious adverse events were noted with Tdap and Td booster in 2 studies on adult IBD patients. Given the small sample size of these studies with very small number of patients on different types of immunosuppressive medications, we downgraded the certainty of evidence by 1 level.

References:

1. Zhang L, Prietsch SO, Axelsson I, Halperin SA. Acellular vaccines for preventing whooping cough in children. Cochrane Database Syst Rev. 2012 Mar 14;(3):CD001478.
2. Wheeler SM, Morton AR. Epidemiological Observations in the Halifax Epidemic. Am J Public Health Nations Health. 1942 Sep;32(9):947-56.
3. Stuart G. Diphtheria Incidence in European Countries. Br Med J. 1945 Nov 3;2(4426):613-5.
4. Miller LW, Older JJ, Drake J, Zimmerman S. Diphtheria immunization. Effect upon carriers and the control of outbreaks. Am J Dis Child. 1972 Mar;123(3):197-9.
5. Marcuse EK, Grand MG. Epidemiology of diphtheria in San Antonio, Tex., 1970. JAMA. 1973 Apr 16;224(3):305-10.
6. Jones EE, Kim-Farley RJ, Algunaid M, Parvez MA, Ballad YA, Hightower AW, Orenstein WA, Broome CV. Diphtheria: a possible foodborne outbreak in Hodeida, Yemen Arab Republic. Bull World Health Organ. 1985;63(2):287-93.
7. Markina SS, Maksimova NM, Vitek CR, Bogatyreva EY, Monisov AA. Diphtheria in the Russian Federation in the 1990s. J Infect Dis. 2000 Feb;181 Suppl 1:S27-34.
8. Chen RT, Hardy IR, Rhodes PH, Tyshchenko DK, Moiseeva AV, Marievsky VF. Ukraine, 1992: first assessment of diphtheria vaccine effectiveness during the recent resurgence of diphtheria in the Former Soviet Union. J Infect Dis. 2000 Feb;181 Suppl 1:S178-83.
9. Bisgard KM, Rhodes P, Hardy IR, Litkina IL, Filatov NN, Monisov AA, Wharton M. Diphtheria toxoid vaccine effectiveness: A case-control study in Russia. J Infect Dis. 2000 Feb;181 Suppl 1:S184-7.
10. Long AP. Tetanus Toxoid, Its Use in the United States Army. Am J Public Health Nations Health. 1943 Jan;33(1):53-7.
11. Boyd JS. Tetanus in the African and European theatres of war, 1939-1945. Lancet. 1946 Jan 26;1(6387):113-9.
12. Banaszkiewicz A, Gawronska A, Klincewicz B, Kofla-Dłubacz A, Grzybowska-Chlebowczyk U, Toporowska-Kowalska E, Malecka I, Stryczynska-Kazubska J, Feleszko W, Lazowska-Przeorek I, Karolewska-Bochenek K, Walkowiak J, Slusarczyk J, Radzikowski A, Demkow U, Albrecht P. Immunogenicity of Pertussis Booster Vaccination in Children and Adolescents with Inflammatory Bowel Disease: A Controlled Study. Inflamm Bowel Dis. 2017 May;23(5):847-852.

13. deBruyn JCC, Soon IS, Fonseca K, Feng S, Purtzki M, Goedhart C, Kuhn S, Vanderkooi OG, Wrobel I. Serologic Status of Routine Childhood Vaccines, Cytomegalovirus, and Epstein-Barr Virus in Children With Inflammatory Bowel Disease. Inflamm Bowel Dis. 2019 Jun 18;25(7):1218-1226.
14. Dezfoli S, Horton HA, Thepyasuwan N, Berel D, Targan SR, Vasiliauskas EA, Dubinsky M, Shih DQ, Kaur M, McGovern DP, Ippoliti A, Feldman EJ, Melmed GY. Combined Immunosuppression Impairs Immunogenicity to Tetanus and Pertussis Vaccination Among Patients with Inflammatory Bowel Disease. Inflamm Bowel Dis. 2015 Aug;21(8):1754-60.
15. Dotan I, Werner L, Vigodman S, Agarwal S, Pfeffer J, Horowitz N, Malter L, Abreu M, Ullman T, Guzner-Gur H, Halpern Z, Mayer L. Normal response to vaccines in inflammatory bowel disease patients treated with thiopurines. Inflamm Bowel Dis. 2012 Feb;18(2):261-8.
16. Nielsen HJ, Mortensen T, Holten-Andersen M, Brünner N, Sørensen S, Rask-Madsen J. Increased levels of specific leukocyte- and platelet-derived substances during normal anti-tetanus antibody synthesis in patients with inactive Crohn disease. Scand J Gastroenterol. 2001 Mar;36(3):265-9.
17. Caldera F, Saha S, Wald A, Garmoe CA, McCrone S, Megna B, Ley D, Reichelderfer M, Hayney MS. Lower Sustained Diphtheria and Pertussis Antibody Concentrations in Inflammatory Bowel Disease Patients. Dig Dis Sci. 2018 Jun;63(6):1532-1540.
18. Moro PL, Perez-Vilar S, Lewis P, Bryant-Genevier M, Kamiya H, Cano M. Safety Surveillance of Diphtheria and Tetanus Toxoids and Acellular Pertussis (DTaP) Vaccines. Pediatrics. 2018 Jul;142(1). pii: e20174171.

Evidence to Decision Table – Adults

**PICO 19**	**In pediatric patients with IBD, should vaccination vs. no vaccination against tetanus, diphtheria, and pertussis be given?**
**Population**	Adult patients with IBD
**Intervention**	Vaccination against tetanus, diphtheria, and pertussis
**Comparator**	No vaccination against tetanus, diphtheria, and pertussis
**Outcome**	Mortality, VPI (tetanus, diphtheria, pertussis), SAEs, Immunogenicity

**PICO 20**	**In adult patients with IBD, should vaccination vs. no vaccination against tetanus, diphtheria, and pertussis with TdAP/Td be given?**
**Population**	Adult patients with IBD
**Intervention**	Vaccination against tetanus, diphtheria, and pertussis with TdAP/Td
**Comparator**	No vaccination against tetanus, diphtheria, and pertussis with TdAP/Td
**Outcome**	Mortality, VPI (tetanus, diphtheria, pertussis), SAEs, Immunogenicity

	Judgement	Research evidence	Additional considerations
**Desirable Effects**	**How substantial are the desirable anticipated effects?**<br><br>○ Trivial<br>○ Small<br>○ Moderate<br>○ Large<br><br>○ Varies<br>○ Don't know	**Risk of Diphtheria, Tetanus, and Pertusis infection in IBD patients**<br><br>The literature search did not identify any study on the risk of tetanus, diphtheria or pertussis infection in adult or pediatric IBD patients.<br><br>There is no RCT comparing diphtheria, tetanus and pertussis vaccines with placebo in patients with IBD to address this PICO question. However, there are immunogenicity studies supporting the safety and effectiveness of these vaccines in both pediatric and adult IBD populatons. As observational studies have not shown any significant difference in immunogencity of these vaccines between pediatric IBD populatoins vs. adult IBD populations vs. healthy controls, the results were presented together in the evidence profile table.<br><br>**Effectiveness of DTap vaccines and Tdap/Td/tetanus booster**<br>The WHO and CDC have assessed the evidence for effectiveness of diphtheria, tetanus and acellular pertussis vaccines in healthy children and adults. For pertussis, a Cochrane systematic review of 6 RCTs found the efficacy of multi-component vaccines varied from 84-85% in preventing pertussis.[1] Although no RCT of the efficacy of diphtheria or tetanus toxoid in preventing disease has ever been conducted, strong evidence from observational studies supports the effectiveness of vaccination.[2-11] The WHO rated the certainty of evidence as **high.** When the evidence is applied to IBD patient populations, the evidence was not downgraded for effectiveness as immunogenicity studies showed that there was no significant difference in seroresponse or seroprotection between IBD patients vs. healthy controls. It is important to note that there are no established guidelines or standard correlates of protection against diphtheria, tetanus and pertussis. The true correlation between antibody level and clinical protection has not been determined. Despite the fact that immunogenicity studies in IBD populations have used different definitions of seroresponse or seroprotection, there was no significant difference found between IBD patients and healthy controls.[12-17] One cross sectional study in adults suggests that IBD patients may have lower sustained diphtheria and pertussis antibody concentrations compard to healthy controls who had received an adult Tdap booster (median 60 months since immunization), and those on anti-TNF monotherapy or combination had lower antibody concentrations compared to those on thiopurine monotherapy.[17] However, the clinical significance of these findings is uncertain given the anamnestic response was not assessed in these patients.[17] **The evidence was therefore anchored to the general population. In summary, there is high certainty evidence that DTap vaccines are effective in pediatric patients with IBD; and Td, Tdap and tetanus boosters are effective in adult patients with IBD. Immunosuppressive medications are not associated with a reduced serological response to these vaccines.**<br><br>**Safety of DTap vaccines and Tdap/Td/tetanus booster**<br>The WHO and CDC have also assessed the evidence of safety of diphtheria, tetanus and acellular pertussis vaccine in healthy children and adults. For pertussis, a Cochrane	
**Undesirable Effects**	**How substantial are the undesirable anticipated effects?**<br><br>○ Large<br>○ Moderate<br>○ Small<br>○ Trivial<br><br>○ Varies<br>○ Don't know		

		systematic review of 52 RCTs found no significant risk of serious adverse events following administration of acellular pertussis vaccines.[1] A safety analysis using the data from the Vaccine Adverse Event Reporting System (VAERS) found no serious adverse events related to the DTap vaccines and no increased risk for neurologic disorders.[18] The WHO rated the certainty of evidence as high. When the evidence is applied to IBD patient populations, the evidence was downgraded 1 level for imprecision given that only 1 study had assessed the safety of pertussis vaccine in pediatric IBD population and 2 studies had assessed the safety of Tdap and Td booster in adult IBD populations. In summary, **there is moderate certainty evidence that DTap are safe in pediatric patients with IBD, and Td / Tdap / tetanus booster are safe in adult patients with IBD.**<br><br>**Overall, the evidence is anchored to the critical outcome of safety (adverse events). There is moderate certainty evidence that diptheria, tetanus and pertussis vaccines are safe and effective in pediatric and adult patients with IBD.**	
**Certainty of evidence**	**What is the overall certainty of the evidence of effects?**<br><br>○ Very low<br>○ Low<br>○ Moderate<br>○ High<br><br>○ No included studies		
**Values and Preferences**	**Is there important uncertainty about or variability in how much people value the main outcomes?**<br><br>○ Important uncertainty or variability<br>○ Possibly important uncertainty or variability<br>○ Probably no important uncertainty or variability<br>○ No important uncertainty or variability	Patients likely value patient-important outcomes (mortality, VPI, adverse effects) more than surrogate outcomes (immunogenicity).	
**Balance of effects**	**Does the balance between desirable and undesirable effects favor the intervention or the comparison?**<br><br>○ Favors the comparison<br>○ Probably favors the comparison<br>○ Does not favor either the intervention or the comparison<br>○ Probably favors the intervention<br>○ Favors the intervention<br><br>○ Varies		

	○ Don't know		
**Resources required**	**How large are the resource requirements (costs)?**<br><br>○ Large costs<br>○ Moderate costs<br>○ Negligible costs and savings<br>○ Moderate savings<br>○ Large savings<br><br>○ Varies<br>○ Don't know	CDC vaccine price list last reviewed/updated: July 1, 2019<br><br><table><tr><th>Brandname</th><th>CDC cost/dose</th><th>Private sector cost/dose</th></tr><tr><td>Tdap<br><br>Boostrix®</td><td>$24.49</td><td>$41.19</td></tr><tr><td>Adacel®</td><td>$24.49</td><td>$45.50</td></tr></table>	
**Certainty of Evidence of Required Resources**	**What is the certainty of the evidence of resource requirements (costs)?**<br><br>○ Very low<br>○ Low<br>○ Moderate<br>○ High<br><br>○ No included studies	The costs of delivering routine immunization services may vary widely across countries and different health system settings. See Immunization Costing Action Network (ICAN) Immunization Delivery Cost Catalogue. http://immunizationeconomics.org/ican-idcc	

**Cost effectiveness**	**Does the cost-effectiveness of the intervention favor the intervention or the comparison?**<br><br>○ Favors the comparison<br>○ Probably favors the comparison<br>○ Does not favor either the intervention or the comparison<br>○ Probably favors the intervention<br>○ Favors the intervention<br><br>○ Varies<br>○ No included studies	There is no cost-effectiveness study of the diptheria, tetanus, or pertussis vaccines in the IBD populations.<br><br>An economic evaluation showed that routine childhood immunization program in the United States including diphtheria and tetanus and acellular pertussis, Haemophilus influenzae type b conjugate, inactivated poliovirus, MMR, hepatitis B, varicella, 7-valent pneumococcal conjugate, hepatitis A, and rotavirus vaccines will prevent 42,000 early deaths and 20 million cases of disease, with net savings of $13.5 billion in direct cost and $68.8 billion in total societal costs, respectively.[19]<br><br>In another economic evaluation, vaccination with DTaP or DTwP in the US resulted in substantial savings, regardless of the perspective taken and for all sensitivity analyses conducted.[20] Without a vaccination program, diphtheria, tetanus, and pertussis disease caused more than 3 million cases and more than 28,000 deaths, at a cost of $23.6 billion. From the societal perspective, net savings because of the use of DTaP and DTwP were $22.510 million and $22.623 million, respectively.[20]<br><br>A decision tree model cost-effectiveness study estimated the cost-effectiveness of implementing the 4 Pillars Program in primary care practices compared to no program for a population of adults 18–64 years of age at high risk of illness complications over a 10 year time horizon.[21] The 4 Pillars™ Practice Transformation Program (4 Pillars Program) increases uptake of pneumococcal polysaccharide vaccine, influenza vaccine and tetanus-diphtheria-acellular pertussis vaccine by 5–10% among adults with high-risk medical conditions.[21] From a third-party payer perspective, which considers direct medical costs, the 4 Pillars Program cost $28,301 per quality-adjusted life year gained; from a societal perspective, which adds direct nonmedical and indirect costs, the program was cost saving and more effective than no intervention.[21]	
**Acceptability**	**Is the intervention acceptable to key stakeholders?**<br><br>○ No<br>○ Probably no<br>○ Probably yes<br>○ Yes<br><br>○ Varies<br>○ Don't know		
**Feasibility**	**Is the intervention feasible to implement?**<br><br>○ No<br>○ Probably no<br>○ Probably yes		

	○ Yes<br><br>○ Varies<br>○ Don't know		

References:

1. Zhang L, Prietsch SO, Axelsson I, Halperin SA. Acellular vaccines for preventing whooping cough in children. Cochrane Database Syst Rev. 2012 Mar 14;(3):CD001478.
2. Wheeler SM, Morton AR. Epidemiological Observations in the Halifax Epidemic. Am J Public Health Nations Health. 1942 Sep;32(9):947-56.
3. Stuart G. Diphtheria Incidence in European Countries. Br Med J. 1945 Nov 3;2(4426):613-5.
4. Miller LW, Older JJ, Drake J, Zimmerman S. Diphtheria immunization. Effect upon carriers and the control of outbreaks. Am J Dis Child. 1972 Mar;123(3):197-9.
5. Marcuse EK, Grand MG. Epidemiology of diphtheria in San Antonio, Tex., 1970. JAMA. 1973 Apr 16;224(3):305-10.
6. Jones EE, Kim-Farley RJ, Algunaid M, Parvez MA, Ballad YA, Hightower AW, Orenstein WA, Broome CV. Diphtheria: a possible foodborne outbreak in Hodeida, Yemen Arab Republic. Bull World Health Organ. 1985;63(2):287-93.
7. Markina SS, Maksimova NM, Vitek CR, Bogatyreva EY, Monisov AA. Diphtheria in the Russian Federation in the 1990s. J Infect Dis. 2000 Feb;181 Suppl 1:S27-34.
8. Chen RT, Hardy IR, Rhodes PH, Tyshchenko DK, Moiseeva AV, Marievsky VF. Ukraine, 1992: first assessment of diphtheria vaccine effectiveness during the recent resurgence of diphtheria in the Former Soviet Union. J Infect Dis. 2000 Feb;181 Suppl 1:S178-83.
9. Bisgard KM, Rhodes P, Hardy IR, Litkina IL, Filatov NN, Monisov AA, Wharton M. Diphtheria toxoid vaccine effectiveness: A case-control study in Russia. J Infect Dis. 2000 Feb;181 Suppl 1:S184-7.
10. Long AP. Tetanus Toxoid, Its Use in the United States Army. Am J Public Health Nations Health. 1943 Jan;33(1):53-7.
11. Boyd JS. Tetanus in the African and European theatres of war, 1939-1945. Lancet. 1946 Jan 26;1(6387):113-9.
12. Banaszkiewicz A, Gawronska A, Klincewicz B, Kofla-Dłubacz A, Grzybowska-Chlebowczyk U, Toporowska-Kowalska E, Malecka I, Stryczynska-Kazubska J, Feleszko W, Lazowska-Przeorek I, Karolewska-Bochenek K, Walkowiak J, Slusarczyk J, Radzikowski A, Demkow U, Albrecht P. Immunogenicity of Pertussis Booster Vaccination in Children and Adolescents with Inflammatory Bowel Disease: A Controlled Study. Inflamm Bowel Dis. 2017 May;23(5):847-852.
13. deBruyn JCC, Soon IS, Fonseca K, Feng S, Purtzki M, Goedhart C, Kuhn S, Vanderkooi OG, Wrobel I. Serologic Status of Routine Childhood Vaccines, Cytomegalovirus, and Epstein-Barr Virus in Children With Inflammatory Bowel Disease. Inflamm Bowel Dis. 2019 Jun 18;25(7):1218-1226.
14. Dezfoli S, Horton HA, Thepyasuwan N, Berel D, Targan SR, Vasiliauskas EA, Dubinsky M, Shih DQ, Kaur M, McGovern DP, Ippoliti A, Feldman EJ, Melmed GY. Combined Immunosuppression Impairs Immunogenicity to Tetanus and Pertussis Vaccination Among Patients with Inflammatory Bowel Disease. Inflamm Bowel Dis. 2015 Aug;21(8):1754-60.
15. Dotan I, Werner L, Vigodman S, Agarwal S, Pfeffer J, Horowitz N, Malter L, Abreu M, Ullman T, Guzner-Gur H, Halpern Z, Mayer L. Normal response to vaccines in inflammatory bowel disease patients treated with thiopurines. Inflamm Bowel Dis. 2012 Feb;18(2):261-8.
16. Nielsen HJ, Mortensen T, Holten-Andersen M, Brünner N, Sørensen S, Rask-Madsen J. Increased levels of specific leukocyte- and platelet-derived substances during normal anti-tetanus antibody synthesis in patients with inactive Crohn disease. Scand J Gastroenterol. 2001 Mar;36(3):265-9.

17. Caldera F, Saha S, Wald A, Garmoe CA, McCrone S, Megna B, Ley D, Reichelderfer M, Hayney MS. Lower Sustained Diphtheria and Pertussis Antibody Concentrations in Inflammatory Bowel Disease Patients. Dig Dis Sci. 2018 Jun;63(6):1532-1540.
18. Moro PL, Perez-Vilar S, Lewis P, Bryant-Genevier M, Kamiya H, Cano M. Safety Surveillance of Diphtheria and Tetanus Toxoids and Acellular Pertussis (DTaP) Vaccines. Pediatrics. 2018 Jul;142(1). pii: e20174171.
19. Zhou F, Shefer A, Wenger J, Messonnier M, Wang LY, Lopez A, Moore M, Murphy TV, Cortese M, Rodewald L. Economic evaluation of the routine childhood immunization program in the United States, 2009. Pediatrics. 2014 Apr;133(4):577-85
20. Ekwueme DU, Strebel PM, Hadler SC, Meltzer MI, Allen JW, Livengood JR. Economic evaluation of use of diphtheria, tetanus, and acellular pertussis vaccine or diphtheria, tetanus, and whole-cell pertussis vaccine in the United States, 1997. Arch Pediatr Adolesc Med. 2000 Aug;154(8):797-803.
21. Wateska AR, Nowalk MP, Zimmerman RK, Smith KJ, Lin CJ. Cost-effectiveness of increasing vaccination in high-risk adults aged 18-64 Years: a model-based decision analysis. BMC Infect Dis. 2018 Jan 25;18(1):52.

Conclusion - Pediatrics

PICO 19: In pediatric patients with IBD, should age-appropriate tetanus, diphtheria, and pertussis-containing vaccines be given?

Moderate certainty of evidence
Direction – Yes (100%)
Strength – Strong (100%)

**Type of recommendation**	**Strong recommendation against the intervention**	**Conditional recommendation against the intervention**	**Conditional recommendation for either the intervention or the comparison**	**Conditional recommendation for the intervention**	**Strong recommendation for the intervention**
	○	○	○	○	○
**Recommendation**	**Statement 15: In pediatric patients with IBD, we recommend age-appropriate tetanus, diphtheria, and pertussis-containing vaccines be given.**				
**Justification**					

**Subgroup considerations**	
**Implementation considerations**	
**Monitoring and evaluation**	• Ongoing monitoring of serious adverse events associated with diphtheria, tetanus, and pertussis vaccines in IBD patients
**Research priorities**	• Observational studies to determine the risks of diptheria, tetanus, and pertussis infection in IBD patients compared to the general population<br>• RCTs or observational studies to determine the clinical effectiveness and immunogenicity of diphtheria, tetanus, and pertussis vaccines in IBD patients on different types of immunosuppressive medications with assessment of patient-important outcomes (i.e. infection with diphtheria, tetanus, and pertussis)

Conclusion - Adults

PICO 20: In adult patients with IBD, should Tdap or Td vaccine be given?

Moderate certainty of evidence
Direction – Yes (100%)
Strength – Strong (89%)

**Type of recommendation**	**Strong recommendation against the intervention**	**Conditional recommendation against the intervention**	**Conditional recommendation for either the intervention or**	**Conditional recommendation for the intervention**	**Strong recommendation for the intervention**

	**the comparison**<br><br>○ ○ ○ ○ ○
**Recommendation**	**Statement 16: In adult patients with IBD, we recommend Tdap/Td vaccine be given.**
**Justification**	
**Subgroup considerations**	
**Implementation considerations**	
**Monitoring and evaluation**	• Ongoing monitoring of serious adverse events associated with diphtheria, tetanus, and pertussis vaccines in IBD patients
**Research priorities**	• Observational studies to determine the risks of diptheria, tetanus, and pertussis infection in IBD patients compared to the general population<br>• RCTs or observational studies to determine the clinical effectiveness and immunogenicity of diphtheria, tetanus, and pertussis vaccines in IBD patients on different types of immunosuppressive medications with assessment of patient-important outcomes (i.e. infection with diphtheria, tetanus, and pertussis)

Human Papillomavirus Virus (HPV)

Background

Human papillomavirus (HPV) infections are the most common sexually transmitted infections. High-risk HPV types can lead to cervical, vaginal, vulvar, penile, anal, and oropharyngeal cancers. Cervical cancer is the second leading cause of cancer mortality among women worldwide. HPV types 16 and 18 cause approximately 70% of all cervical cancers. HPV types 31, 33, 45, 52, and 58 account for approximately 15-19% of cervical cancers. Low-risk HPV types can cause anogenital warts.

CDC ACIP recommends routine HPV vaccination for girls and boys at ages 11 or 12 years to protect against cancers caused by HPV infections (vaccination can be started at age 9).[1] Catch-up vaccination has been recommended through age 26 years.[1] CDC ACIP does not recommend catch-up vaccination of adults aged 27-45 years, but recognize that some adults who are not previously vaccinated may be at risk for new HPV infection and might benefit from vaccination in this age range. Therefore, ACIP recommends HPV vaccination based on shared clinical decision making for individuals ages 27 though 45 years who are not adequately vaccinated.[1] HPV vaccines are not licensed for use in adults older than age 45 years. Previous studies have demonstrated an increased risk of cervical cancer among immunosuppressed patients in the transplant and HIV populations.[2] The above recommendations also apply to people with "immunocompromising" conditions. NACI recommends routine HPV vaccine (HPV2, HPV4 or HPV9) for females aged 9 to 26 years and <u>may</u> be used in females over 26 years of age.[3] HPV4 or HPV9 vaccine is recommended routinely for males aged 9 to 26 years, and <u>may</u> be used in males over 26 years.[3] NACI also recommends HPV vaccine for any immunocompromised individual (defined as individuals with impaired immune responsiveness, whether due to the use of immunosuppressive therapy, a genetic defect, HIV infection, or other causes) and immunocompetent HIV infected individuals.[3] However, WHO deemed the evidence supporting vaccination of HIV-infected young adolescent girls to prevent cervical cancer later in life to be of low quality.

ACG recommends women with IBD on immunosuppressive therapy undergo annual cervical cancer screening (*conditional recommendation, very low level of evidence*).[4] However, it makes no specific recommendation about HPV vaccine in IBD patients. ECCO recommends routine prophylactic HPV vaccination for females and males according to national guidelines.[5]

Three HPV vaccines are licensed for use in the US and Canada: 9-valent (9vHPV, Gardasil 9), quadrivalent (4vHPV, Gardasil), and bivalent HPV vaccines (2vHPV, Cervarix). Until October 2018, all three HPV vaccines are licensed for use in the US in persons aged 9 through 26 years. Since late 2016, only 9-valent HPV vaccine has been available in the US. In October 2018, FDA approved an expansion of the age indication through age 45 years for 9-valent HPV.

All HPV vaccines are highly immunogenic in the general population. More than 98% of recipients develop an antibody response to HPV types included in the respective vaccines 1 month after completing the 3-dose series. However, there is no known correlate of immunity and no known minimal titer determined to be protective. There is abundant evidence for safety of HPV vaccines with few serious adverse events.

References:

1. Centers for Disease Control and Prevention. https://www.cdc.gov/flu/professionals/vaccination/index.htm
2. Busnach G, Piselli P, Arbustini E, Baccarani U, Burra P, Carrieri MP, Citterio F, De Juli E, Bellelli S, Pradier C, Rezza G, Serraino D; Immunosuppression and Cancer Study Group. Immunosuppression and cancer: A comparison of risks in recipients of organ transplants and in HIV-positive individuals. Transplant Proc. 2006 Dec;38(10):3533-5.
3. Public Health Agency of Canada. https://www.canada.ca/en/public-health/services/publications/healthy-living/9-valent-hpv-vaccine-clarification-minimum-intervals-between-doses-in-hpv-immunization-schedule.html
4. Farraye FA, Melmed GY, Lichtenstein GR, Kane SV. ACG Clinical Guideline: Preventive Care in Inflammatory Bowel Disease. Am J Gastroenterol. 2017 Feb;112(2):241-258.
5. Rahier JF, Magro F, Abreu C, Armuzzi A, Ben-Horin S, Chowers Y, Cottone M, de Ridder L, Doherty G, Ehehalt R, Esteve M, Katsanos K, Lees CW, Macmahon E, Moreels T, Reinisch W, Tilg H, Tremblay L, Veereman-Wauters G, Viget N, Yazdanpanah Y, Eliakim R, Colombel JF; European Crohn's and Colitis Organisation (ECCO). Second European evidence-based consensus on the prevention, diagnosis and management of opportunistic infections in inflammatory bowel disease. J Crohns Colitis. 2014 Jun;8(6):443-68.
6. World Health Organization. https://www.who.int/immunization/policy/position_papers/hpv/en/

Risk of HPV infection in IBD patients

PICO: What is the risk of HPV infection in people with IBD compared to people without IBD?

Summary – Adults and Pediatric

Literature search did not identify any study on the risk of HPV infection in adult or pediatric IBD patients. It is important to note that cervical cancer is almost exclusively caused by HPV infection, although the majority of women infected with HPV do not develop cervical cancer. So, **a more relevant and important question is whether IBD patients have an increased risk of developing cervical cancer.**

Twelve observational studies addressed this PICO question.[1-12] A systematic review published in 2015 included 8 of the 12 studies.[13] We decided not to pool all studies together in a meta-analysis as the studies were very heterogeneous in terms of methodology and outcome selections. Most importantly, both cervical carcinoma and/or varying degrees of dysplasia or cervical abnormalities were used across studies. As spontaneous recovery of low-grade dysplasia has been observed in many patients, the clinical implication of detected dysplasia is therefore uncertain. **Overall, the studies showed conflicting results as to whether IBD patients have an increased risk of developing cervical abnormalities. However, IBD patients on immunosuppressants may have an increased risk of developing "cervical abnormalities".**

The GRADE rating started at high as these was considered prognostic studies (providing evidence about the likelihood of cervical abnormalities in patients with IBD). The rating was further downgraded to **very low** due to study limitations (residual confounding factors, detection bias, and misclassification bias), inconsistency, and indirectness (outcomes). Most studies did not adjust for known risk factors of cervical cancer including smoking, HPV status, use of oral contraceptives, behavioral characteristics such as sex at early age or multiple sexual partners, socioeconomic factors, and multi-parity. Data were reliant on administrative diagnoses in larger (more precise) studies. Possible misclassification errors due to errors of miscoding, and the codes have not been previously validated. Data in regard to medication use in most studies was based on ever versus never use of these drugs, and did not specify the actual or cumulative dose of medications. Frequency of pap smears and duration of follow-up (healthcare utilization) were not accounted for in many studies. Patients with IBD are more likely to seek health care and to require multiple physician visits as compared to the general population, and thus may have a higher rate of detection of cervical abnormalities than the general population. Finally, different outcomes were used across studies including cervical abnormalities (atypical squamous cells of undetermined significance, CIN 1 or worse, or cervical cancer), abnormal pap smears, low grade or high-grade dysplasia, and cancer. **In summary, there is very low certainty evidence that adult IBD patients on immunosuppressants have an increased risk of "cervical abnormalities" compared to non-IBD patients.**

There is one systematic review of **anal squamous cell carcinoma (SCC)** in IBD patients.[14] A total of 33 cases of anal SCC was described in the literature based on mostly case reports and case series. Although its incidence may be raised in patients with Crohn's disease compared to the general population, anal SCC is a **very rare entity**.

Risk of Bias Table

SR of Observational Studies and RCTs

Study	Quality Assessment	Comments
Allegretti 2015	• Overall quality was assessed as “good” by Newcastle-Ottawa Score (6/8 studies scored $\geq$ 8)<br>• **Possible residual confounding**: behavioral characteristics such as sex at early age or multiple sexual partners, socioeconomic factors, multi-parity, healthcare utilization, disease severity/duration, etc.<br>• Errors in data extraction of the largest study (Kim 2014): data presented were unadjusted OR in all IBD patients (not the subgroup of patients on IS). The adjusted HR for the subgroup of IBD patients on IS therapy was 1.72 (0.66-4.45) - not significant.<br>• Some studies provided adjusted data, but unadjusted data were used instead when pooling data for MA<br>• **Outcomes were highly variable among studies and in most cases cannot be combined into HGD/cancer**<br>• Possible publication bias on funnel plot	• SR and MA of 5 cohort and 3 case-control studies of 77,116 IBD patients on any immunosuppression therapy and HGD on Pap smear or CIN 2/3 or cervical cancer on biopsy (n = 955). Controls were general population with no IBD who had a pap test matched by age in 7 studies.<br>• Duration of follow-up was variable (2 – 36 years)<br>• Highly variable in the degree and duration of IS<br>• Confounders such as smoking and oral contraceptive pills were often controlled for<br>• **IBD patients on IS had an increased risk of HGD / cervical cancer compared with healthy controls (OR 1.34, 95% CI 1.23-1.46).** I^2 = 34.23 (minimal-moderate heterogeneity)<br>• Unable to stratify the data by level of IS, duration, or individual drugs
Slesser 2013	• High risk for selection bias<br>• Not included in evidence profile due to majority of studies being case reports or case series.	• SR of 11 observational studies (case report/case series, cohort, case control) of anal squamous cell carcinoma (SCC) in IBD patients<br>• A total of 33 cases of anal SCC was identified (a rare entity)<br>• Incidence 0.9/100,000 in UC and 2.0/100,000 in CD

HGD: high grade dysplasia
IS: immunosuppressive therapy

Evidence Profile Table

Certainty Assessment								Summary of Findings	
Studies	Risk of bias	Inconsistency	Indirectness	Imprecision	Other considerations	Certainty of Evidence	Overall Certainty of evidence	Study Event Rates	Relative Effect (95% CI)

VPI (Cervical dysplasia or cancer) - CRITICAL							⨁⊖⊖⊖ VERY LOW	
12 Observational studies (prognostic studies)[1-12]<br><br>1 SR of 8 Observational studies[13]	Very serious[a]	Serious[b]	Serious[c]	Not serious	Possible publication bias	⨁⊖⊖⊖ VERY LOW		• See **Summary of observational studies assessing the risks of cervical dysplasia or cancer in patients with IBD**<br>• Compared to the general population, the risks of cervical abnormalities (not cancer) may be higher in IBD patients who are on immunosuppressants.

Footnotes:

a. Downgraded two levels for study limitations. Possible residual confounding factors including smoking, HPV status, behavioral characteristics such as sex at early age or multiple sexual partners, socioeconomic factors, multi-parity, healthcare utilization (more frequent physician visits and more pap smears in IBD patients), as well as disease severity/duration may over-estimate the risk of cervical cancer and dysplasia in IBD patients compared to healthy controls. High risk for detection bias as patients with IBD may be more likely to undergo pap smears for screening. Data were reliant on administrative diagnoses in larger studies. Possible misclassification errors due to errors of miscoding, and the codes have not been previously validated.
b. Downgraded one level for inconsistency in results (more inconsistency in IBD patients overall than in IBD patients on immunosuppressants).
c. Downgraded one level for indirectness as different outcomes were used across studies including cervical abnormalities (atypical squamous cells of undetermined significance, CIN 1 or worse, or cervical cancer), abnormal pap smears, low grade or high-grade dysplasia and cancer. As spontaneous recovery of low-grade dysplasia has been observed in many patients, the clinical implication of detected dysplasia is uncertain.

Summary of observational studies assessing the risks of cervical dysplasia or cancer in patients with IBD

	Number of IBD patients	Factors adjusted	Risks for cervical HGD and cancer among all IBD patients	Risks for cervical HGD and cancer among IBD patients on IS
Jung 2017<br>Population-based<br>(Korea)	15,291	Age	CD: -<br>UC: SIR 5.65 (2.44-11.13) for cervical cancer	-

Dugue 2015<br>Population-based<br>(Denmark)	45,166	Age	CD: SIR 1.3 (0.9-1.7)<br>UC: SIR 0.9 (0.7-1.1)<br>for cervical cancer	AHR 2.2 (1.2-3.9) for high cumulative dose of Azathioprine for patients with autoimmune diseases for cervical cancer. No subgroup data for IBD.
Kim 2015<br>Population-based<br>(US)	25,176	Age, risk factors for HPV (alcoholism, smoking, substance abuse, sexually active, STD, OCP, HPV vaccine, CKD and CLD), comorbidities, medications, healthcare use factors	AHR 1.07 (0.79-1.45)<br>for cervical HGD and cancer	AHR 1.72 (0.66-4.45)<br>for cervical HGD and cancer
Rungoe 2015<br>Population-based<br>(Denmark)	27,408	Age, municipality at diagnosis of IBD, comorbidities	CD: IRR 1.53 (1.04-2.27)<br>UC: IRR 0.78 (0.53-1.13)<br>for cervical cancer<br><br>CD: IRR 1.28 (1.13-1.45)<br>UC: IRR 1.12 (1.01-1.25) for HSIL	CD on anti-TNF:<br>IRR 1.85 (1.12-3.04) for HSIL
Jess 2013[5]<br>Population-based<br>(Denmark)	2211	Age at IBD diagnosis, extent of disease, smoking, medications	CD: SIR 1.65 (1.10-2.37)<br>UC: SIR 0.71 (0.43-1.11) for cervical dysplasia including carcinoma in situ	CD on thiopurines: SIR 2.47 (1.54-3.73) for cervical dysplasia including carcinoma in situ
Singh 2009[6]<br>Population-based<br>(Canada)	525	Socioeconomic status, OCP, NSAIDs, duration of follow-up	AOR 0.98 (0.80-1.19) for cervical abnormalities (atypical squamous cells of undetermined significance, CIN 1 or worse, or cervical cancer)	Steroids: AOR 0.97 (0.93-1.02)<br>IS: AOR 1.1 (0.76-1.59)<br>Combined steroids + IS: AOR 1.41 (1.09-1.81)<br>for cervical abnormalities
Lees 2009[7]<br>Single tertiary referral center<br>(Scotland)	362	Smoking, OCP, age at diagnosis, disease duration, IS exposure	No difference<br>6.9% vs. 7.3% (p = 0.375) for HGD	No difference<br>5.7% vs. 7.7% (p = 0.654) for any IS
Marehbian 2009[8]	22,310	Age	-	Monotherapy with steroids, IS,

Population-based (US)				or anti-TNF: HR 1.5 (1.2-2.0)<br><br>Two or more IS: HR 1.8 (1.1-3.0)<br><br>For cervical dysplasia
Kane 2008[9]<br>Single tertiary referral center (US)	40	Age, race, diagnosis, smoking, family history of cervical dysplasia, marital status, parity, and sexual history	OR 3.1 (1.3-8.7)<br>"higher-risk abnormalities" including atypical squamous cells-high grade dysplasia, low- and high-grade squamous intraepithelial lesions<br><br>No cancer	IM: OR 4.5 (1.5-12.3) for abnormal Pap<br><br>No cancer
Hutfless 2008[10]<br>Population-based (US)	1254	Age, ethnicity, smoking	AOR 1.45 (0.74-2.84)<br>for cervical cancer (cervical intraepithelial neoplasia grade 3 or greater)	IM: AOR 3.45 (0.82-14.45)<br>Steroids: AOR 2.79 (0.71-11.0)<br>for cervical cancer (cervical intraepithelial neoplasia grade 3 or greater)
Bhatia 2006[11]<br>Single tertiary referral center (US)	116	No	18% vs. 5% in healthy controls (P = 0.04) for abnormal Pap including atypical squamous cells of undetermined significance, low-grade squamous intraepithelial lesion, high-grade squamous intraepithelial lesion<br><br>No cancer	No difference for steroids, IS and anti-TNF
Bernstein 2001[12]<br>Population-based (Canada)	6027	No	IRR 0.91 (0.28-2.97)<br>For cervical cancer	-

CKD: chronic kidney disease
CLD: chronic liver disease
HGD: high grade dysplasia
HSIL: moderate-to-severe cervical intraepithelial neoplasia. The latter includes carcinoma in situ.
IM: immunomodulator
IS: immunosuppressive therapy

OCP: oral contraceptive use
STD: sexually transmitted disease
Increased risk – highlighted in yellow

References:

1. Jung YS, Han M, Park S, Kim WH, Cheon JH. Cancer Risk in the Early Stages of Inflammatory Bowel Disease in Korean Patients: A Nationwide Population-based Study. J Crohns Colitis. 2017 Aug 1;11(8):954-962.
2. Dugué PA, Rebolj M, Hallas J, Garred P, Lynge E. Risk of cervical cancer in women with autoimmune diseases, in relation with their use of immunosuppressants and screening: population-based cohort study. Int J Cancer. 2015 Mar 15;136(6):E711-9.
3. Kim SC, Glynn RJ, Giovannucci E, Hernández-Díaz S, Liu J, Feldman S, Karlson EW, Schneeweiss S, Solomon DH. Risk of high-grade cervical dysplasia and cervical cancer in women with systemic inflammatory diseases: a population-based cohort study. Ann Rheum Dis. 2015 Jul;74(7):1360-7.
4. Rungoe C, Simonsen J, Riis L, Frisch M, Langholz E, Jess T. Inflammatory bowel disease and cervical neoplasia: a population-based nationwide cohort study. Clin Gastroenterol Hepatol. 2015 Apr;13(4):693-700.
5. Jess T, Horváth-Puhó E, Fallingborg J, Rasmussen HH, Jacobsen BA. Cancer risk in inflammatory bowel disease according to patient phenotype and treatment: a Danish population-based cohort study. Am J Gastroenterol. 2013 Dec;108(12):1869-76.
6. Singh H, Demers AA, Nugent Z, Mahmud SM, Kliewer EV, Bernstein CN. Risk of cervical abnormalities in women with inflammatory bowel disease: a population-based nested case-control study. Gastroenterology. 2009 Feb;136(2):451-8.
7. Lees CW, Critchley J, Chee N, Beez T, Gailer RE, Williams AR, Shand AG, Arnott ID, Satsangi J. Lack of association between cervical dysplasia and IBD: a large case-control study. Inflamm Bowel Dis. 2009 Nov;15(11):1621-9.
8. Marehbian J, Arrighi HM, Hass S, Tian H, Sandborn WJ. Adverse events associated with common therapy regimens for moderate-to-severe Crohn's disease. Am J Gastroenterol. 2009 Oct;104(10):2524-33.
9. Kane S, Khatibi B, Reddy D. Higher incidence of abnormal Pap smears in women with inflammatory bowel disease. Am J Gastroenterol. 2008 Mar;103(3):631-6.
10. Hutfless S, Fireman B, Kane S, Herrinton LJ. Screening differences and risk of cervical cancer in inflammatory bowel disease. Aliment Pharmacol Ther. 2008 Sep 1;28(5):598-605.
11. Bhatia J, Bratcher J, Korelitz B, Vakher K, Mannor S, Shevchuk M, Panagopoulos G, Ofer A, Tamas E, Kotsali P, Vele O. Abnormalities of uterine cervix in women with inflammatory bowel disease. World J Gastroenterol. 2006 Oct 14;12(38):6167-71.
12. Bernstein CN, Blanchard JF, Kliewer E, Wajda A. Cancer risk in patients with inflammatory bowel disease: a population-based study. Cancer. 2001 Feb 15;91(4):854-62.
13. Allegretti JR, Barnes EL, Cameron A. Are patients with inflammatory bowel disease on chronic immunosuppressive therapy at increased risk of cervical high-grade dysplasia/cancer? A meta-analysis. Inflamm Bowel Dis. 2015 May;21(5):1089-97.
14. Slesser AA, Bhangu A, Bower M, Goldin R, Tekkis PP. A systematic review of anal squamous cell carcinoma in inflammatory bowel disease. Surg Oncol. 2013 Dec;22(4):230-7.

Effectiveness and Safety of HPV vaccine in IBD patients

Summary

There was no RCT comparing HPV vaccines with placebo in patients with IBD to address this PICO question.

One before-and-after study[1] assessed post-vaccination titers in female IBD patients aged 9-26. All 48 patients (33 in the prospective cohort and 15 in the retrospective cohort) were on immunomodulators or anti-TNF. The results were compared to Merck reported titers of healthy females aged 9-15 and 16-26 after Gardasil quadrivalent vaccine (HPV 6, 11, 16,18) at 1, 2 and 6 months. The immunogenicity of the quadrivalent vaccine appears to be comparable to healthy controls. However, the titers decrease over time, and the seroresponse to HPV type 18 may be lower (40% using the cLIA assay and 93% using the total IgG cLIA assay) in IBD patients. Further studies will be needed to understand the pattern of immune response over time. Because of the small sample size, limited duration of follow-up, and lack of cervical sampling, it is uncertain whether the immunologic response confers protection to HPV infection or cervical cancer.

The evidentiary base of CDC ACIP recommendations for HPV vaccines were reviewed. The CDC ACIP recommends:

- Routine HPV vaccination of females at age 11 or 12 years (9vHPV: moderate level of evidence)
- Catch-up HPV vaccination of females at age 13 through 26 years (9vHPV: moderate level of evidence)
- Routine HPV vaccination of males at age 11 or 12 years (9vHPG: low level of evidence)
- Catch-up HPV vaccination of males at age 13 through 21 years (9vHPV: low level of evidence)
- The CDC ACIP did not recommend catch-up HPV vaccination of adults at age 27-45 years (9vHPV: moderate level of evidence), but recognized that some adults who are not previously vaccinated may be at risk for new HPV infection and might benefit from vaccination in this age range. Therefore, ACIP recommended shared clinical decision making regarding potential HPV vaccination for these individuals.

We decided to assess the evidence for the use of 9vHPV vaccines (not 4vHPV or 2vHPV vaccines) in IBD populations for 3 reasons: 1) Since late 2016, only 9vHPV vaccine has been available in the US; 2) CDC evidence profile tables were only available for 9vHPV vaccines; and 3) many countries are now shifting to 9vHPV (Gardasil 9). The evidence was presented according to gender and age. Since there was 1 small study done in age- and gender-specific IBD population that supported the findings in age- and gender-specific general populations, the evidence was not downgraded for indirectness for patient population (general population vs. IBD population) and the evidence would be anchored with the general population.

9vHPV Vaccine in the female IBD Population – catch-up age group from age 13 through 26 (using CDC evidence profile tables as anchor)

As per CDC, data used for the evidence review were from 9vHPV pre-licensure clinical trials as well as the efficacy trials from the 4vHPV program. The pivotal efficacy trial for 9vHPV was conducted in females aged 16 through 26 years.[2] This was a randomized trial comparing 9vHPV with 4vHPV conducted among approximately 14,000 females aged 16 through 26 years. Evidence used to evaluate efficacy of 9vHPV for prevention of HPV 31, 33, 45, 52, 58-related outcomes were directly from this trial.[2] Evidence used to evaluate efficacy of 9vHPG for prevention of HPV 6, 11, 16, 18-related outcomes was from RCTs of 4vHPV and from immunogenicity studies comparing 9vHPV and 4vHPV; these data were used to infer 9vHPV efficacy for HPV 6, 11, 16, 18-related outcomes. It is important to note that the relationship between CIN2 and cervical cancer is not clear-cut as most CIN2 lesions in women below age 30 regress spontaneously. And CIN≥2 was used as a surrogate outcome for cervical cancer in these studies. The GRADE assessment was anchored to the evidence for general population for females in the catch-up age group (≥ CIN2), the rating started at moderate for effectiveness. As one observational study in female IBD population age 9-26 suggests the immunogenicity of the quadrivalent vaccine appears to be comparable to healthy controls, we did not downgrade for indirectness for effectiveness (general population vs. female IBD population).[1] For safety, the rating started at high, but downgraded to moderate as small sample size in female IBD population cannot detect rare adverse events.[1] **Overall, there is moderate certainty evidence that 9vHPV vaccine is safe and effective in reducing the risks of CIN≥ 2 given to female IBD patients aged 13 through 26. For the outcome of cervical cancer, the certainty of evidence is low (downgraded due to indirectness of outcomes).**

If the GRADE assessment was done using only evidence on 4vHPV, we would not need to downgrade for indirectness due to immunobridging, and the certainty of evidence for efficacy would be upgraded one level. But since adverse events is a considered a critical outcome, the overall certainty of evidence will not change as this is dependent on the lowest rating of all critical outcomes.

9vHPV Vaccine in the female IBD Population - routine age group at age 9 to 12 (Using CDC evidence profile tables as anchor)

For HPV vaccination of females in the routine age group, evidence from 2 immunobridging trials was also used. One trial compared 9vHPV in females aged 9 through 15 years with females aged 16 through 26 years, and another trial compared 9vHPV with 4vHPV in females aged 9 through 15 years.[3,4] Non-inferior immunogenicity of 9vHPV compared with 4vHPV in females aged 9 through 15 years and 9vHPV in females aged 9 through 15 years compared with females aged 16 through 26 years was used to infer efficacy for

prevention of HPV 6, 11, 16, 18, 31, 33, 45, 52, 58-related outcomes. The GRADE assessment was anchored to the evidence for general population for females in the routine age group (≥ CIN2), the rating started at moderate for effectiveness. As one observational study in female IBD population age 9-26 suggests the immunogenicity of the quadrivalent vaccine appears to be comparable to healthy controls, we did not downgrade for indirectness for effectiveness (general population vs. female IBD population).[1] For safety, the rating started at high, but downgraded to moderate as small sample size in female IBD population cannot detect rare adverse events.[1] **Overall, there is moderate certainty evidence that 9vHPV vaccine is safe and effective in reducing the risks of CIN≥ 2 given to female IBD patients aged 11 or 12. For the outcome of cervical cancer, the certainty of evidence for effectiveness is low.**

9vHPV Vaccine in the male IBD Population – catch-up from age 13 through 26 and routine age group at age 9 to 12 (Using CDC evidence profile tables as anchor)

For HPV vaccination of males, evidence used to evaluate efficacy of 9vHPV for prevention of HPV 6, 11, 16, 18-related outcomes was from 1 RCT of 4vHPV among approximately 4,000 males aged 16 through 26 years, which evaluated anogenital warts; anal precancer outcomes were evaluated in a subset of approximately 600;[5] and an immunogenicity study comparing 9vHPV in males with females aged 16 through 26 years.[3] Noninferior immunogenicity of 9vHPV in males compared with females was used to infer efficacy for prevention of HPV 6, 11, 16, and 18-related outcomes. For HPV vaccination of males in the routine age group, evidence was also from an immunobridging trial, which showed non-inferior immunogenicity of 9vHPV in males aged 9 through 15 years compared to females aged 16 through 26 years.[3] These data was used to infer efficacy for prevention of HPV 6, 11, 16, 18-related outcomes. The GRADE assessment was anchored at evidence for general population for males (anal cancer), and the rating started at moderate for effectiveness. However, the rating was downgraded to **low** due to indirectness (immunogenicity data from female IBD population). For safety, the rating started as low, and was downgraded to very low due to indirectness (no data on male IBD populations and small sample size in female IBD population cannot detect rare adverse events). **Overall, there is very low certainty evidence that 9vHPV vaccine is safe and effective in reducing the risks of anal cancer in male IBD patients at age 9 to 12 and from age 13 through 26.**

9vHPV Vaccine in the female and male IBD Population – catch-up from age 27 through 45 (using CDC evidence profile tables as anchor)

Since no RCTs were conducted on use of 9vHPV in this age range, extrapolation from 4vHPV efficacy was based on immunobridging data. For men, evidence was further downgraded for each outcome for indirectness since most trials enrolled women only. The GRADE assessment was anchored at evidence for general population, the rating started at moderate for female and low for male for the **combined endpoint of persistent vaccine-type HPV infections, anogenital warts, and/or CIN $\geq$ 1**. We did not downgrade for indirectness for effectiveness for female IBD patients as 1 observational study suggested comparable immunogenicity with the general population.[1] However, the rating for effectiveness was downgraded to low for male IBD patients due to indirectness (immunogenicity data from female IBD population). For safety, the GRADE rating started at moderate, and was downgraded to low for female IBD patients as small sample size in IBD population cannot detect rare adverse events. The evidence for safety was further downgraded to very low for male IBD population as safety data was from female IBD population. **Overall, there is low and very low certainty evidence that 9vHPV vaccine is safe and effective in female IBD population (age 27 through 45) and male IBD population (age 27 through 45), respectively.**

There is a very controversial Cochrane Systematic review on HPV vaccine effects on cervical lesions in adolescent girls and women.[6] It has been criticized for missing nearly half of the eligible trials, using composite surrogate outcomes for cervical cancer (in line with World Health Organization recommendations), incompletely assessed serious and systemic adverse events, and industry trial funding of all but one of the trials, and major conflicts of interests related to HPV vaccine manufacturers for most of the Cochrane authors on the first published protocol of the review.[7] There were numerous requests for Cochrane to withdraw this review. For these reasons, we have decided not to use this review as evidentiary base. The review included 26 trials (73,438 participants). Studies involved monovalent (n = 1), bivalent (n = 18), and quadrivalent vaccines (n = 7). Most women were under 26 years of age. Three trials recruited women 25 and over. HPV vaccines reduce any CIN2+ from 559 to 391/10,000 (RR 0.70, 95% CI: 0.58-0.85, high certainty evidence) and any adenocarcinoma in situ from 17 to 5/10,000 (RR 0.32, 95% CI 0.15-0.67, high certainty evidence). No data reported for cervical cancer. In women vaccinated at 24 to 45 years of age, there is moderate-certainty evidence that the risks of CIN2+ are similar between vaccinated and unvaccinated women (RR 1.04, 95% CI 0.83-1.30). The risks of serious adverse events are similar between control and HPV vaccines in women of all ages (RR 0.98, 95% CI 0.92-1.05, high certainty evidence). The death rate was significantly increased in women above age 25 (RR 2.36, 95% CI 1.10-5.03); no absolute numbers were provided for this subgroup analysis, but the total number of deaths were 51 in the HPV vaccine groups and 39 in the comparator group.

References:

1. Jacobson DL, Bousvaros A, Ashworth L, Carey R, Shrier LA, Burchett SK, Renna H, Lu Y. Immunogenicity and tolerability to human papillomavirus-like particle vaccine in girls and young women with inflammatory bowel disease. Inflamm Bowel Dis. 2013 Jun;19(7):1441-9.
2. Joura E, Bautista O, Luxembourg A. A 9-Valent HPV Vaccine in Women. N Engl J Med. 2015 Jun 25;372(26):2568-9.

3. Luxembourg A. program summary and new 9-valent HPV vaccine trial data. Presentation before the Advisory Committee on Immunization Practices (ACIP), October 30, 2014. Atlanta, GA: US Department of Health and Human Services, CDC;2014.
4. Yang S, Miller N, Fink D, Hulse A. Original application: human papillomavirus 9-valent vaccine, recombinant. Silver Spring, MD: US Department of Health and Human Services; Food and Drug Administration; 2014.
5. Palefsky JM, Giuliano AR, Goldstone S, Moreira ED Jr, Aranda C, Jessen H, Hillman R, Ferris D, Coutlee F, Stoler MH, Marshall JB, Radley D, Vuocolo S, Haupt RM, Guris D, Garner EI. HPV vaccine against anal HPV infection and anal intraepithelial neoplasia. N Engl J Med. 2011 Oct 27;365(17):1576-85.
6. Arbyn M, Xu L, Simoens C, Martin-Hirsch PP. Prophylactic vaccination against human papillomaviruses to prevent cervical cancer and its precursors. Cochrane Database Syst Rev. 2018 May 9;5:CD009069.
7. Jørgensen L, Gøtzsche PC, Jefferson T. The Cochrane HPV vaccine review was incomplete and ignored important evidence of bias. BMJ Evid Based Med. 2018 Oct;23(5):165-168.

Risk of Bias Table

Before-After (Pre-Post) Studies									
			If no concurrent comparator group was used						
**Study**	**Was there a concurrent comparator group that did not receive the intervention**	**If a concurrent comparator group was used, was it similar to the intervention group (or adequately adjusted) for prognostic factors**	**If each participant served as his/her own control (assessed before vs. after the intervention), are there compelling arguments that the outcome was not influenced by historic**	**If two different consecutive cohorts of participants were assessed (before vs. after implementation of the intervention), are there (a) compelling arguments that the outcome was not influenced by historic events / underlying**	**Outcome detection methods valid and similar among compared groups / periods**	**Incomplete outcome data assessed**	**Selective outcome reporting**	**Other bias**	**Comments**

			**events / underlying secular trends**	**secular trends and (b) evidence that the two groups were similar (or adequately adjusted) for prognostic factors**					
Jacobson 2013 (US)	No – but this does not affect the risk of bias as the only explanation for increase in titer is the vaccine (no other confounding factors)	No – but this does not affect the risk of bias as the only explanation for increase in titer is the vaccine (no other confounding factors)	OK	OK	OK	4/37 prospective cohort did not complete the study: 2 lost to follow-up and 2 discontinued.	OK	OK	• Cohort study assessing pre- and post-vaccination titer in females aged 9 – 26 with IBD. Compared to Merck reported titers of healthy females aged 9-15 and 16-26 after Gardasil quadrivalent vaccine (HPV 6, 11, 16,18) at 1, 2 and 6 mo<br>• <u>Prospective cohort</u> **(33 females, median age 15**) on IS, 51% on anti-TNF and 49% on IM. Outcomes assessed pre- and month 7.<br>• <u>Prospective cohor</u>t: 100% seropositive to HPV 6, 11, 16. **96% seropositive to HPV 18 on cLIA assay**. GMT titer was as high or higher to all 4 HPV types compared to Merck comparison

									group within each age group.<br>• 5 SAEs (unrelated to the vaccine): 2 exacerbations, 1 pneumonia, 1 ovarian torsion due to endometriosis, 1 acute sinus pain.<br>• <u>Retrospective cohort,</u> previously vaccinated **(15 females, median age 18**) on IS, 67% on anti-TNF, 33% on IM. Outcomes assessed up to 27 mos post.<br>• <u>Retrospective cohort</u>: **100% seropositive to HPV 6, 11, 16. 40% seropositive to HPV 18 cLIA assay.** Seropositive to HPV 6, 11, 16 and 18 was 93%, 87%, 100% and 93% by total IgG cLIA. Titers decreased with time since dose 3.

IS: immunosuppressants
IM: immunomodulators

SR of Observational Studies and RCTs		
**Study**	**Quality Assessment**	**Comments**
Pellegrino 2015	• No quality assessment was done<br>• High risk for selection bias<br>• As it does not change the certainty of evidence for IBD	• SR of 5 cohort studies (3 on SLE patients, 2 on juvenile idiopathic arthritis, and 1 on IBD – Jacobson 2013). Total 194 patients<br>• HPV vaccines appear to be immunogenic and safe in <u>most</u> of the patients affected by

	populations, this study was not included in the evidence profile.	autoimmune diseases. While seroconversion rates appear similar to controls, patients receiving immunosuppressive drugs had lower anti-HPV titers after vaccination.<br>• The results do not suggest a risk of significant disease exacerbation following vaccination. However, the results were achieved on a limited number of cases, thus cannot exclude that small groups tagged by specific disease phenotypes or genetic background may develop disease flares as a result of immunization.<br>• No information on long-term antibody response or clinical outcomes.<br>• Some points of concern remain to be tackled, including the effect of concomitant therapies, the risk of disease exacerbation, and the cost-effectiveness of such immunization programmes in these populations.

References:

1. Jacobson DL, Bousvaros A, Ashworth L, Carey R, Shrier LA, Burchett SK, Renna H, Lu Y. Immunogenicity and tolerability to human papillomavirus-like particle vaccine in girls and young women with inflammatory bowel disease. Inflamm Bowel Dis. 2013 Jun;19(7):1441-9.
2. Pellegrino P, Radice S, Clementi E. Immunogenicity and safety of the human papillomavirus vaccine in patients with autoimmune diseases: A systematic review. Vaccine. 2015 Jul 9;33(30):3444-9.

Evidence Profile Table

9vHPV Vaccine in the female IBD Population – catch-up age group from age 13 through 26 (using CDC evidence profile tables as anchor)

**Certainty Assessment**								**Summary of Findings**	
Studies	Risk of bias	Inconsistency	Indirectness	Imprecision	Other considerations	Certainty of Evidence	Overall Certainty of evidence	Study Event Rates	Relative Effect (95% CI)
**≥ CIN 2 (HPV 6, 11, 16, 18 related) - CRITICAL**									
3 RCTs[1]<br>4vHPV<br>2 Observational studies[2,3]	Not serious	Not serious	Serious[a]	Not serious	Not serious	⊕⊕⊕⊖<br>MODERATE	⊕⊕⊕⊖<br>MODERATE	• Efficacy 98.2% (93.3 -99.8%) per protocol	

**General Population**								
1 Observational study[4] **IBD Population**	Not serious	Not serious	Serious[b]	Serious[c]	None	⊕⊖⊖⊖ VERY LOW		• No significant difference in seropositivity between females with IBD aged 9 – 26 years and healthy females
**Cervical cancer (HPV 6, 11, 16, 18 related) - CRITICAL**								
3 RCTs[1] 4vHPV 2 Observational studies[2,3] **General Population**	Not serious	Not serious	Very serious[a,d]	Not serious	Not serious	⊕⊕⊖⊖ LOW		• See ≥ CIN2 (HPV 6, 11, 16, 18)
**Anogenital warts (HPV 6, 11, 16, 18 related) - CRITICAL**								
1 RCT[5] 2 Observational studies[2,3] 4vHPV **General Population**	Not serious	Not serious	Serious[a]	Not serious	Not serious	⊕⊕⊕⊖ MODERATE		• Efficacy 99.0% (96-100%) per protocol
**≥ CIN 2 (HPV 31, 33, 45, 52, 58 related) - CRITICAL**								
1 RCT[6] 9vHPV **General Population**	Not serious	Not serious	Not serious	Not serious	Not serious	⊕⊕⊕⊕ HIGH		• Efficacy 96.3% (79.5-99.8%) per protocol<br>• Absolute risk difference 4 fewer per 1000 (3-5)<br>• Number needed to vaccinate 250 (200-333)
**Cervical cancer (HPV 31, 33, 45, 52, 58 related) - CRITICAL**								
1 RCT[6] 2 Observational studies[2,3] 9vHPV **General Population**	Not serious	Not serious	Serious[d]	Not serious	Not serious	⊕⊕⊕⊖ MODERATE		• See ≥ CIN2 (HPV 31, 33, 45, 52, 58)
**Adverse events - CRITICAL**								
2 RCTs[3,5] 4 Observational studies[2,3]	Not serious	Not serious	Serious[e]	Not serious	None	⊕⊕⊕⊖ MODERATE		• Few cases of serious adverse events

**General Population**								
1<br>Observational study[4]<br><br>**IBD Population**	Not serious	Not serious	Not serious	Serious[c]	None	⊕⊖⊖⊖<br>VERY LOW		• No clinically significant vaccine-associated adverse events

Footnotes:

a. Downgraded for indirectness due to immunobridging to 4vHPV
b. Downgraded for indirectness. Surrogate outcome of immunogenicity was used.
c. Downgraded for imprecision due to small sample size.
d. Downgraded for indirectness due to use of ≥ CIN2 as surrogate marker for cervical cancer.
e. Downgraded for indirectness (general population, not IBD patients). Sample size in IBD patients not large enough to detect rare adverse events.

References:

1. Kjaer SK, Sigurdsson K, Iversen OE, Hernandez-Avila M, Wheeler CM, Perez G, Brown DR, Koutsky LA, Tay EH, García P, Ault KA, Garland SM, Leodolter S, Olsson SE, Tang GW, Ferris DG, Paavonen J, Lehtinen M, Steben M, Bosch FX, Dillner J, Joura EA, Majewski S, Muñoz N, Myers ER, Villa LL, Taddeo FJ, Roberts C, Tadesse A, Bryan J, Maansson R, Lu S, Vuocolo S, Hesley TM, Saah A, Barr E, Haupt RM. A pooled analysis of continued prophylactic efficacy of quadrivalent human papillomavirus (Types 6/11/16/18) vaccine against high-grade cervical and external genital lesions. Cancer Prev Res (Phila). 2009 Oct;2(10):868-78. (3 clinical trials: Protocols 007, 013 and 015)
2. Luxembourg A. program summary and new 9-valent HPV vaccine trial data. Presentation before the Advisory Committee on Immunization Practices (ACIP), October 30, 2014. Atlanta, GA: US Department of Health and Human Services, CDC;2014.
3. Yang S, Miller N, Fink D, Hulse A. Original application: human papillomavirus 9-valent vaccine, recombinant. Silver Spring, MD: US Department of Health and Human Services; Food and Drug Administration; 2014.
4. Jacobson DL, Bousvaros A, Ashworth L, Carey R, Shrier LA, Burchett SK, Renna H, Lu Y. Immunogenicity and tolerability to human papillomavirus-like particle vaccine in girls and young women with inflammatory bowel disease. Inflamm Bowel Dis. 2013 Jun;19(7):1441-9.
5. FUTURE I/II Study Group, Dillner J, Kjaer SK, Wheeler CM, Sigurdsson K, Iversen OE, Hernandez-Avila M, Perez G, Brown DR, Koutsky LA, Tay EH, García P, Ault KA, Garland SM, Leodolter S, Olsson SE, Tang GW, Ferris DG, Paavonen J, Lehtinen M, Steben M, Bosch FX, Joura EA, Majewski S, Muñoz N, Myers ER, Villa LL, Taddeo FJ, Roberts C, Tadesse A, Bryan JT, Maansson R, Lu S, Vuocolo S, Hesley TM, Barr E, Haupt R. Four year efficacy of prophylactic human papillomavirus quadrivalent vaccine against low grade cervical, vulvar, and vaginal intraepithelial neoplasia and anogenital warts: randomised controlled trial. BMJ. 2010 Jul 20;341:c3493
6. Joura E, Bautista O, Luxembourg A. A 9-Valent HPV Vaccine in Women. N Engl J Med. 2015 Jun 25;372(26):2568-9.

9vHPV Vaccine in the female IBD Population - routine age group at age 9 to 12 (Using CDC evidence profile tables as anchor)

Certainty Assessment								Summary of Findings	
Studies	Risk of bias	Inconsistency	Indirectness	Imprecision	Other considerations	Certainty of Evidence	Overall Certainty of evidence	Study Event Rates	Relative Effect (95% CI)
**> CIN 2 (HPV 6, 11, 16, 18 related) - CRITICAL**							⊕⊕⊕⊖ MODERATE		
3 RCTs[1] 4vHPV 2 Observational studies[2,3] **General Population**	Not serious	Not serious	Serious[a]	Not serious	Not serious	⊕⊕⊕⊖ MODERATE		• Efficacy 98.2% (93.3 -99.8%) per protocol and 51.5% (40.6-60.6%) intention to treat	
1 Observational study[4] **IBD Population**	Not serious	Not serious	Serious[b]	Serious[c]	None	⊕⊖⊖⊖ VERY LOW		• No significant difference in seropositivity between females with IBD aged 9 – 26 years and healthy females	
**Cervical cancer (HPV 6, 11, 16, 18 related) - CRITICAL**									
3 RCT[1] 4vHPV 2 Observational studies[2,3] **General Population**	Not serious	Not serious	Very serious[a,d]	Not serious	Not serious	⊕⊕⊖⊖ LOW		• See > CIN2 (HPV 6, 11, 16, 18)	
**Anogenital warts (HPV 6, 11, 16, 18 related) - IMPORTANT**									
1 RCT[5] 4vHPV 2 Observational studies[2,3] **General Population**	Not serious	Not serious	Serious[a]	Not serious	Not serious	⊕⊕⊕⊖ MODERATE		• Efficacy 99.0% (96-100%) per protocol	
**> CIN 2 (HPV 31, 33, 45, 52, 58 related) - CRITICAL**									
1 RCT[6] 9vHPV 4 Observational studies[2,3] **General Population**	Not serious	Not serious	Not serious	Not serious	Not serious	⊕⊕⊕⊕ HIGH		• Efficacy 96.3% (79.5-99.8%) per protocol<br>• Absolute risk difference 4 fewer per 1000 (3-5)<br>• Number needed to vaccinate 250 (200-333)	

**Cervical cancer (HPV 31, 33, 45, 52, 58 related) - CRITICAL**								
1 RCT[6]<br>9vHPV<br>4 Observational studies[2,3]<br>9vHPV<br><br>**General Population**	Not serious	Not serious	Serious[d]	Not serious	Not serious	⊕⊕⊕⊝<br>MODERATE		• See ≥ CIN2 (HPV 31, 33, 45, 52, 58)
**Adverse events - CRITICAL**								
2 RCTs[3, 5]<br>4 Observational studies[2,3]<br><br>**General Population**	Not serious	Not serious	Serious[e]	Not serious	None	⊕⊕⊕⊝<br>MODERATE		• Few cases of serious adverse events
1<br>Observational study[4]<br><br>**IBD Population**	Not serious	Not serious	Not serious	Serious[c]	None	⊕⊝⊝⊝<br>VERY LOW		• No clinically significant vaccine-associated adverse events

Footnotes:

a. Downgraded for indirectness due to immunobridging to 4vHPV. But not downgraded due to non-inferior immunogenicity among females aged 9-15 years compared with females aged 16-26 years.
b. Downgraded for indirectness. Surrogate outcome of immunogenicity was used.
c. Downgraded for imprecision due to small sample size
d. Downgraded for indirectness due to use of ≥ CIN2 as surrogate marker for cervical cancer
e. Downgraded for indirectness (general population, not IBD patients). Sample size in IBD patients not large enough to detect rare adverse events.

References:

1. Kjaer SK, Sigurdsson K, Iversen OE, Hernandez-Avila M, Wheeler CM, Perez G, Brown DR, Koutsky LA, Tay EH, García P, Ault KA, Garland SM, Leodolter S, Olsson SE, Tang GW, Ferris DG, Paavonen J, Lehtinen M, Steben M, Bosch FX, Dillner J, Joura EA, Majewski S, Muñoz N, Myers ER, Villa LL, Taddeo FJ, Roberts C, Tadesse A, Bryan J, Maansson R, Lu S, Vuocolo S, Hesley TM, Saah A, Barr E, Haupt RM. A pooled analysis of continued prophylactic efficacy of quadrivalent human papillomavirus (Types 6/11/16/18) vaccine against high-grade cervical and external genital lesions. Cancer Prev Res (Phila). 2009 Oct;2(10):868-78. (Protocols 007, 013 and 015)
2. Luxembourg A. program summary and new 9-valent HPV vaccine trial data. Presentation before the Advisory Committee on Immunization Practices (ACIP), October 30, 2014. Atlanta, GA: US Department of Health and Human Services, CDC;2014.
3. Yang S, Miller N, Fink D, Hulse A. Original application: human papillomavirus 9-valent vaccine, recombinant. Silver Spring, MD: US Department of Health and Human Services; Food and Drug Administration; 2014.

4. Jacobson DL, Bousvaros A, Ashworth L, Carey R, Shrier LA, Burchett SK, Renna H, Lu Y. Immunogenicity and tolerability to human papillomavirus-like particle vaccine in girls and young women with inflammatory bowel disease. Inflamm Bowel Dis. 2013 Jun;19(7):1441-9.
5. FUTURE I/II Study Group, Dillner J, Kjaer SK, Wheeler CM, Sigurdsson K, Iversen OE, Hernandez-Avila M, Perez G, Brown DR, Koutsky LA, Tay EH, García P, Ault KA, Garland SM, Leodolter S, Olsson SE, Tang GW, Ferris DG, Paavonen J, Lehtinen M, Steben M, Bosch FX, Joura EA, Majewski S, Muñoz N, Myers ER, Villa LL, Taddeo FJ, Roberts C, Tadesse A, Bryan JT, Maansson R, Lu S, Vuocolo S, Hesley TM, Barr E, Haupt R. Four year efficacy of prophylactic human papillomavirus quadrivalent vaccine against low grade cervical, vulvar, and vaginal intraepithelial neoplasia and anogenital warts: randomised controlled trial. BMJ. 2010 Jul 20;341:c3493
6. Joura E, Bautista O, Luxembourg A. A 9-Valent HPV Vaccine in Women. N Engl J Med. 2015 Jun 25;372(26):2568-9.

9vHPV Vaccine in the male IBD Population – catch-up from age 13 through 26 and routine age group at age 9 to 12 (Using CDC evidence profile tables as anchor)

Certainty Assessment								Summary of Findings	
Studies	Risk of bias	Inconsistency	Indirectness	Imprecision	Other considerations	Certainty of Evidence	Overall Certainty of evidence	Study Event Rates	Relative Effect (95% CI)
**Anal cancer - CRITICAL**							⊕⊖⊖⊖ VERY LOW		
1 RCT[1]<br>4vHPV<br>1 Observational study[2]<br>**General Population**	Not serious	Not serious	Very serious[a,b]	Not serious	Not serious	⊕⊕⊖⊖ LOW		• Efficacy 74.9% (8.8-95.4%) per protocol	
1 Observational study[3]<br>**Female IBD Population**	Not serious	Not serious	Very serious[c]	Serious[d]	None	⊕⊖⊖⊖ VERY LOW		• No significant difference in seropositivity between IBD patients aged 9 – 26 years and healthy persons	
**Anogenital warts (HPV 6, 11, 16, 18 related) - IMPORTANT**									
1 RCT[4]<br>1 Observational study[2]<br>4vHPV<br>**General Population**	Not serious	Not serious	Serious[b]	Not serious	Not serious	⊕⊕⊕⊖ MODERATE		• Efficacy 99.0% (96-100%) per protocol	
**Adverse events - CRITICAL**									

2 RCTs[4,5]<br>4 Observational studies[2,5]<br><br>**General Population**	Not serious	Not serious	Very serious[e]	Serious[d]	None	⊕⊖⊖⊖<br>VERY LOW		• Few cases of serious adverse events
1<br>Observational study[3]<br><br>**Female IBD Population**	Not serious	Not serious	Serious[c]	Serious[d]	None	⊕⊖⊖⊖<br>VERY LOW		• No clinically significant vaccine-associated adverse events

Footnotes:

a. Downgraded for very serious indirectness due to the use of anal intraepithelial neoplasia grade 2 or 3 as surrogate marker for anal cancer. And supported only by immunogenicity data from female IBD population.
b. Downgraded for indirectness due to the use of immunobridging to females aged 16-26 years.
c. Downgraded for indirectness as surrogate outcome of immunogenicity was used and no data on males.
d. Downgraded for imprecision due to small sample size
e. Downgraded for indirectness as no data on male IBD populations and small sample size in female IBD population cannot detect rare adverse events.

References:

1. Palefsky JM, Giuliano AR, Goldstone S, Moreira ED Jr, Aranda C, Jessen H, Hillman R, Ferris D, Coutlee F, Stoler MH, Marshall JB, Radley D, Vuocolo S, Haupt RM, Guris D, Garner EI. HPV vaccine against anal HPV infection and anal intraepithelial neoplasia. N Engl J Med. 2011 Oct 27;365(17):1576-85.
2. Luxembourg A. program summary and new 9-valent HPV vaccine trial data. Presentation before the Advisory Committee on Immunization Practices (ACIP), October 30, 2014. Atlanta, GA: US Department of Health and Human Services, CDC;2014.
3. Jacobson DL, Bousvaros A, Ashworth L, Carey R, Shrier LA, Burchett SK, Renna H, Lu Y. Immunogenicity and tolerability to human papillomavirus-like particle vaccine in girls and young women with inflammatory bowel disease. Inflamm Bowel Dis. 2013 Jun;19(7):1441-9.
4. FUTURE I/II Study Group, Dillner J, Kjaer SK, Wheeler CM, Sigurdsson K, Iversen OE, Hernandez-Avila M, Perez G, Brown DR, Koutsky LA, Tay EH, García P, Ault KA, Garland SM, Leodolter S, Olsson SE, Tang GW, Ferris DG, Paavonen J, Lehtinen M, Steben M, Bosch FX, Joura EA, Majewski S, Muñoz N, Myers ER, Villa LL, Taddeo FJ, Roberts C, Tadesse A, Bryan JT, Maansson R, Lu S, Vuocolo S, Hesley TM, Barr E, Haupt R. Four year efficacy of prophylactic human papillomavirus quadrivalent vaccine against low grade cervical, vulvar, and vaginal intraepithelial neoplasia and anogenital warts: randomised controlled trial. BMJ. 2010 Jul 20;341:c3493
5. Yang S, Miller N, Fink D, Hulse A. Original application: human papillomavirus 9-valent vaccine, recombinant. Silver Spring, MD: US Department of Health and Human Services; Food and Drug Administration; 2014.

9vHPV Vaccine in the female and male IBD Population – catch-up from age 27 through 45 (using CDC evidence profile tables as anchor)

**Certainty Assessment**	**Summary of Findings**

Studies	Risk of bias	Inconsistency	Indirectness	Imprecision	Other considerations	Certainty of Evidence	Overall Certainty of evidence	Study Event Rates	Relative Effect (95% CI)
**Combined end point of persistent vaccine-type HPV infections, anogenital warts and/or CIN ≥ 1 - CRITICAL**									
3 RCT[1-3]<br>4vHPV<br>Observational and RCT studies of immunobridging data[3-12]<br><br>**General Population**	Not serious	Not serious	Serious[a]<br>for female<br><br>Very serious[a]<br>for male	Not serious	Not serious	⊕⊕⊕⊖<br>MODERATE<br>For female<br><br>⊕⊕⊖⊖<br>LOW<br>For male			• 87.7% (75.4-95.6%) per protocol[1]<br>• 90.5% (69.9-98.2%) per protocol[2]
**CIN ≥ 2 - CRITICAL**									
3 RCT[1-3]<br>4vHPV<br>Observational and RCT studies of immunobridging data[3-12]<br><br>**General Population**	Not serious	Not serious	Serious[a]	Serious[b]	Not serious	⊕⊕⊖⊖<br>LOW	⊕⊕⊖⊖<br>LOW<br>For female<br><br>⊕⊕⊖⊖<br>VERY LOW<br>For male		• 79.8% (-80.1-99.6%) per protocol[1]<br>• 100% (-51.0-100%) per protocol[2]
1<br>Observational study[13]<br><br>**Female IBD Population**	Not serious	Not serious	Serious[c]<br>for female<br><br>Very serious[c]<br>For male	Serious[d]	None	⊕⊕⊖⊖<br>VERY LOW			• No significant difference in seropositivity between with female IBD patients aged 9 – 26 years and healthy persons
**Vaccine-related Serious Adverse events - CRITICAL**									
3 RCT[1-3]<br>4vHPV<br>Observational and RCT studies of immunobridging data[3-12]<br><br>**General Population**	Not serious	Not serious	Serious[a]<br>for female<br><br>Very serious[a]<br>for male	Serious[d]	None	⊕⊕⊖⊖<br>LOW<br>For female<br><br>⊕⊕⊖⊖<br>VERY LOW<br>For male			• Few cases of vaccine-related serious adverse events
1<br>Observational study[3]<br><br>**Female IBD Population**	Not serious	Not serious	Serious[a]<br>for female<br><br>Very serious[e]<br>for male	Serious[d]	None	⊕⊖⊖⊖<br>VERY LOW			• No clinically significant vaccine-associated adverse events

Footnotes:

a. Downgraded for indirectness since no RCTs were conducted on use of 9vHPV in adults aged 27 through 45 years, and there are no 4HPV efficacy trials in males aged 27 through 45 years; extrapolation of efficacy (and safety) from 4vHPV across age and genders is based on bridging immunogenicity data. Downgraded for very serious indirectness for male IBD population as immunogenicity and safety data from female IBD population. Downgraded for serious indirectness for female IBD population as no female IBD patients > age 26 were included in the study. Small sample size of female IBD population cannot detect rare adverse events.
b. Downgraded for imprecision since 95% CI for efficacy includes 1
c. Downgraded for serious indirectness due to the use of surrogate outcome of immunogenicity for female IBD populations. Downgraded for very serious indirectness due to the use of surrogate outcome of immunenicity and no data on male IBD populations.
d. Downgraded for imprecision due to small sample size.
e. Downgraded for indirectness for male as safety outcome data from female IBD population.

References:

1. Castellsagué X, Muñoz N, Pitisuttithum P, Ferris D, Monsonego J, Ault K, Luna J, Myers E, Mallary S, Bautista OM, Bryan J, Vuocolo S, Haupt RM, Saah A. End-of-study safety, immunogenicity, and efficacy of quadrivalent HPV (types 6, 11, 16, 18) recombinant vaccine in adult women 24-45 years of age. Br J Cancer. 2011 Jun 28;105(1):28-37. doi: 10.1038/bjc.2011.185. Epub 2011 May 31.
2. Wei L, Xie X, Liu J, Zhao Y, Chen W, Zhao C, Wang S, Liao X, Shou Q, Qiu Y, Qiao Y, Saah AJ. Efficacy of quadrivalent human papillomavirus vaccine against persistent infection and genital disease in Chinese women: A randomized, placebo-controlled trial with 78-month follow-up. Vaccine. 2019 Jun 12;37(27):3617-3624.
3. Luxembourg A. 9vHPV immunogenicity and safety trial in mid-adult females. Advisory Committee on Immunization Practice (ACIP). Atlanta, GA. June 26 2019.
4. Hillman RJ, Giuliano AR, Palefsky JM, Goldstone S, Moreira ED Jr, Vardas E, Aranda C, Jessen H, Ferris DG, Coutlee F, Marshall JB, Vuocolo S, Haupt RM, Guris D, Garner EI. Immunogenicity of the quadrivalent human papillomavirus (type 6/11/16/18) vaccine in males 16 to 26 years old. Clin Vaccine Immunol. 2012 Feb;19(2):261-7.
5. Giuliano AR, Palefsky JM, Goldstone S, Moreira ED Jr, Penny ME, Aranda C, Vardas E, Moi H, Jessen H, Hillman R, Chang YH, Ferris D, Rouleau D, Bryan J, Marshall JB, Vuocolo S, Barr E, Radley D, Haupt RM, Guris D. Giuliano AR, Palefsky JM, Goldstone S, Moreira ED Jr, Penny ME, Aranda C, Vardas E, Moi H, Jessen H, Hillman R, Chang YH, Ferris D, Rouleau D, Bryan J, Marshall JB, Vuocolo S, Barr E, Radley D, Haupt RM, Guris D. N Engl J Med. 2011 Feb 3;364(5):401-11.
6. Palefsky JM, Giuliano AR, Goldstone S, Moreira ED Jr, Aranda C, Jessen H, Hillman R, Ferris D, Coutlee F, Stoler MH, Marshall JB, Radley D, Vuocolo S, Haupt RM, Guris D, Garner EI. HPV vaccine against anal HPV infection and anal intraepithelial neoplasia. N Engl J Med. 2011 Oct 27;365(17):1576-85.
7. Luxembourg A. 9vHPV Vaccine for Mid-Adult Persons (27-45 yo) Results from Clinical Studies. Advisory Committee on Immunization Practices (ACIP). Atlanta, GA. June 20k 2018
8. Joura E, Bautista O, Luxembourg A. A 9-Valent HPV Vaccine in Women. N Engl J Med. 2015 Jun 25;372(26):2568-9.
9. Huh WK, Joura EA, Giuliano AR, Iversen OE, de Andrade RP, Ault KA, Bartholomew D, Cestero RM, Fedrizzi EN, Hirschberg AL, Mayrand MH, Ruiz-Sternberg AM, Stapleton JT, Wiley DJ, Ferenczy A, Kurman R, Ronnett BM, Stoler MH, Cuzick J, Garland SM, Kjaer SK, Bautista OM, Haupt R, Moeller E, Ritter M, Roberts CC, Shields C, Luxembourg A. Final efficacy, immunogenicity, and safety analyses of a nine-valent human papillomavirus vaccine in women aged 16-26 years: a randomised, double-blind trial. Lancet. 2017 Nov 11;390(10108):2143-2159.
10. Van Damme P, Meijer CJLM, Kieninger D, Schuyleman A, Thomas S, Luxembourg A, Baudin M. A phase III clinical study to compare the immunogenicity and safety of the 9-valent and quadrivalent HPV vaccines in men. Vaccine. 2016 Jul 29;34(35):4205-4212.

11. Donahue J. Rapid Cycle Analysis of the 9-valent human papillomavirus vaccine (9vHPV) in the Vaccine Safety Datalink. Advisory Committee on Immunization Practices (ACIP). Atlanta, GA. February 21, 2018
12. Arana J. Adverse events following 9-valent human papillomavirus vaccine (9vHPV) reported to the Vaccine Adverse Event Reporting System (VAERS). Advisory Committee on Immunization Practices (ACIP). Atlanta, GA. February 21, 2018.
13. Jacobson DL, Bousvaros A, Ashworth L, Carey R, Shrier LA, Burchett SK, Renna H, Lu Y. Immunogenicity and tolerability to human papillomavirus-like particle vaccine in girls and young women with inflammatory bowel disease. Inflamm Bowel Dis. 2013 Jun;19(7):1441-9

Evidence to Decision Table – Females with IBD age 9 to 26

PICO 21	In female patients with IBD age 9 to 26, should vaccination vs. no vaccination against human papillomavirus (HPV) be given?
Population	Female patients with IBD age 9 to 26
Intervention	Vaccination against HPV
Comparator	No vaccination against HPV
Outcome	Mortality, VPI (HPV and HPV-related complications), SAEs, Immunogenicity

	Judgement	Research evidence	Additional considerations
Desirable Effects	**How substantial are the desirable anticipated effects?**<br><br>o Trivial<br>o Small<br>o Moderate<br>o Large<br><br>o Varies<br>o Don't know	See Evidence Profile Tables.<br><br>**Risk of HPV infection in IBD patients**<br><br>Literature search did not identify any study on the risk of HPV infection in adult or pediatric IBD patients. It is important to note that cervical cancer is almost exclusively caused by HPV infection, although the majority of women infected with HPV do not develop cervical cancer. So, **a more relevant and important question is whether IBD patients have an increased risk of developing cervical cancer due to HPV infection.**<br><br>Twelve observational studies addressed this PICO question.[1-12] A systematic review	

Undesirable Effects	How substantial are the undesirable anticipated effects?		
	**How substantial are the undesirable anticipated effects?**<br><br>○ Large<br>○ Moderate<br>○ Small<br>○ Trivial<br><br>○ Varies<br>○ Don't know	published in 2015 included 8 of the 12 studies.[13] We decided not to pool all studies together in a meta-analysis as the studies were very heterogeneous in terms of methodology and outcome selections. Most importantly, both cervical carcinoma and/or varying degrees of dysplasia were used as outcomes. As spontaneous recovery of low-grade dysplasia has been observed in many patients, the clinical implication of detected dysplasia is therefore uncertain. Overall, the studies showed conflicting results as to whether IBD patients have an increased risk of developing cervical abnormalities. However, IBD patients on immunosuppressants may have an increased risk of developing cervical abnormalities.<br><br>The GRADE rating started at high as these was considered prognostic studies (providing evidence about the likelihood of cervical abnormalities in patients with IBD). The rating was further downgraded to **very low** due to study limitations (residual confounding factors, detection bias, and misclassification bias), inconsistency, and indirectness (outcomes). Most studies did not adjust for known risk factors of cervical cancer including smoking, HPV status, use of oral contraceptives, behavioral characteristics such as sex at early age or multiple sexual partners, socioeconomic factors, and multi-parity. Data were reliant on administrative diagnoses in larger (more precise) studies. Possible misclassification errors due to errors of miscoding, and the codes have not been previously validated. Data in regard to medication use in most studies was based on ever versus never use of these drugs, and did not specify the actual or cumulative dose of medications. Frequency of pap smears and duration of follow-up (healthcare utilization) were not accounted for in many studies. Patients with IBD are more likely to seek health care and to require multiple physician visits as compared to the general population, and thus may have a higher rate of detection of cervical abnormalities than the general population. Finally, different outcomes were used across studies including cervical abnormalities (atypical squamous cells of undetermined significance, CIN 1 or worse, or cervical cancer), abnormal pap smears, low grade or high-grade dysplasia, and cancer. **In summary, there is very low certainty evidence that adult IBD patients on immunosuppressants have an increased risk of cervical abnormalities compared to non-IBD patients**.<br><br>There is one systematic review of **anal squamous cell carcinoma (SCC)** in IBD patients.[14] A total of 33 cases of anal SCC was described in the literature based on mostly case reports and case series. Although its incidence may be raised in patients with Crohn's disease compared to the general population, anal SCC is a very rare entity.<br><br>**Effectiveness and Safety of HPV Vaccine in IBD patients**<br><br>There was no RCT comparing HPV vaccines with placebo in patients with IBD to address this PICO question.<br><br>One before-and-after study[15] assessed post-vaccination titers in female IBD patients aged 9-26. All 48 patients (33 in the prospective cohort and 15 in the retrospective cohort) were on immunomodulators or anti-TNF. The results were compared to Merck reported titers of healthy females aged 9-15 and 16-26 after Gardasil quadrivalent vaccine (HPV 6, 11, 16,18) at 1, 2 and 6 months. The immunogenicity of the	

quadrivalent vaccine appears to be comparable to healthy controls. However, the titers decrease over time, and the seroresponse to HPV type 18 may be lower (40% using the cLIA assay and 93% using the total IgG cLIA assay) in IBD patients. Further studies will be needed to understand the pattern of immune response over time. Because of the small sample size, limited duration of follow-up, and lack of cervical sampling, it is uncertain whether the immunologic response confers protection to HPV infection or cervical cancer.

9vHPV Vaccine in the female IBD Population – catch-up age group from age 13 through 26 (using CDC evidence profile tables as anchor)

As per CDC, data used for the evidence review were from 9vHPV pre-licensure clinical trials as well as the efficacy trials from the 4vHPV program. The pivotal efficacy trial for 9vHPV was conducted in females aged 16 through 26 years.[16] This was a randomized trial comparing 9vHPV with 4vHPV conducted among approximately 14,000 females aged 16 through 26 years. Evidence used to evaluate efficacy of 9vHPV for prevention of HPV 31, 33, 45, 52, 58-related outcomes were directly from this trial.[16] Evidence used to evaluate efficacy of 9vHPG for prevention of HPV 6, 11, 16, 18-related outcomes was from RCTs of 4vHPV and from immunogenicity studies comparing 9vHPV and 4vHPV; these data were used to infer 9vHPV efficacy for HPV 6, 11, 16, 18-related outcomes. It is important to note that the relationship between CIN2 and cervical cancer is not clear-cut as most CIN2 lesions in women below age 30 regress spontaneously. And CIN≥2 was used as a surrogate outcome for cervical cancer in these studies. The GRADE assessment was anchored to the evidence for general population for females in the catch-up age group (≥ CIN2), the rating started at moderate for effectiveness. As one observational study in female IBD population age 9-26 suggests the immunogenicity of the quadrivalent vaccine appears to be comparable to healthy controls, we did not downgrade for indirectness for effectiveness (general population vs. female IBD population).[15] For safety, the rating started at high, but downgraded to moderate as small sample size in female IBD population cannot detect rare adverse events.[15] **Overall, there is moderate certainty evidence that 9vHPV vaccine is safe and effective in reducing the risks of CIN≥ 2 given to female IBD patients aged 13 through 26. For the outcome of cervical cancer, the certainty of evidence for efficacy is low.**

9vHPV Vaccine in the female IBD Population - routine age group at age 11 or 12 (Using CDC evidence profile tables as anchor)

For HPV vaccination of females in the routine age group, evidence from 2 immunobridging trials was also used. One trial compared 9vHPV in females aged 9 through 15 years with females aged 16 through 26 years, and another trial compared 9vHPV with 4vHPV in females aged 9 through 15 years.[17,18] Non-inferior immunogenicity of 9vHPV compared with 4vHPV in females aged 9 through 15 years and 9vHPV in females aged 9 through 15 years compared with females aged 16 through 26 years was used to infer efficacy for prevention of HPV 6, 11, 16, 18, 31, 33, 45, 52, 58-related outcomes. The GRADE assessment was anchored to the evidence for general population for females in the routine age group (≥ CIN2), the rating started at moderate for effectiveness. As one observational study in female IBD population age 9-26

		suggests the immunogenicity of the quadrivalent vaccine appears to be comparable to healthy controls, we did not downgrade for indirectness for effectiveness (general population vs. female IBD population).[15] For safety, the rating started at high, but downgraded to moderate as small sample size in female IBD population cannot detect rare adverse events.[15] **Overall, there is moderate certainty evidence that 9vHPV vaccine is safe and effective in reducing the risks of CIN≥ 2 given to female IBD patients aged 11 or 12. For the outcome of cervical cancer, the certainty of evidence for efficacy is low.**	
**Certainty of evidence**	**What is the overall certainty of the evidence of effects?**<br><br>○ Very low<br>○ Low for cervical cancer<br>○ Moderate for CIN ≥ 2<br>○ High<br><br>○ No included studies	See Evidence Profile Tables.	
**Values and Preferences**	**Is there important uncertainty about or variability in how much people value the main outcomes?**<br><br>○ Important uncertainty or variability<br>○ Possibly important uncertainty or variability<br>○ Probably no important uncertainty or variability<br>○ No important uncertainty or variability	Patients likely value the outcomes of cervical cancer and vaccine-related adverse effects more than CIN≥2.	
**Balance of effects**	**Does the balance between desirable and undesirable effects favor the intervention or the comparison?**<br><br>○ Favors the comparison<br>○ Probably favors the comparison<br>○ Does not favor either the intervention or the comparison<br>○ Probably favors the intervention<br>○ Favors the intervention<br><br>○ Varies<br>○ Don't know		

<table>
<tr><td>Resources required</td><td>How large are the resource requirements (costs)?

○ Large costs
○ Moderate costs
○ Negligible costs and savings school age
○ Moderate savings
○ Large savings

○ Varies outside school age
○ Don't know</td><td>CDC vaccine price list last reviewed/updated: July 1, 2019

<table><tr><th>Brand name</th><th>CDC cost/dose</th><th>Private sector cost/dose</th></tr><tr><td>Gardasil®9</td><td>$178.14</td><td>$227.93</td></tr></table></td><td></td></tr>
<tr><td>Certainty of Evidence of Required Resources</td><td>What is the certainty of the evidence of resource requirements (costs)?

○ Very low
○ Low
○ Moderate
○ High

○ No included studies</td><td>The costs of delivering routine immunization services may vary widely across countries and different health system settings. See Immunization Costing Action Network (ICAN) Immunization Delivery Cost Catalogue. http://immunizationeconomics.org/ican-idcc.</td><td></td></tr>
<tr><td>Cost effectiveness</td><td>Does the cost-effectiveness of the intervention favor the intervention or the comparison?

○ Favors the comparison
○ Probably favors the comparison
○ Does not favor either the intervention or the comparison
○ Probably favors the intervention
○ Favors the intervention

○ Varies
○ No included studies</td><td>There are no studies that addressed this question specifically in the IBD population.

Many economic evaluations of HPV vaccination have been published. The considerable between study heterogeneity in economic evaluations of HPV vaccination programmes makes comparisons between studies difficult, as observed differences in cost effectiveness may be driven by differences in methodology as well as by variations in funding and delivery models and estimates of model parameters.

In a recent systemic review of the cost-effectiveness of implementing an HPV vaccination programme with routine cervical cancer screening, a total of 29 studies were included (17 looked only at cervical disease outcomes, and 12 also included non-cervical disease outcomes).[19] Bivalent vaccine and quadrivalent vaccines were evaluated in these studies. The majority modelled HPV vaccination in adolescent girls, one looked at HPV vaccination in women over the age of 35 years, and one included both girls and boys in the model. While different model structures, input parameters and baseline assumptions were used, the consistent message in studies that focused on female-only vaccination programmes was that routine vaccination of females is cost effective compared with cervical screening alone. It appears the addition of boys to a vaccination programme generally exceeds traditional cost effectiveness thresholds ($US 50,000 per QALY).

In a systematic review of cost-effectiveness studies of 9-valent vaccine comparing to bi- or quadrivalent vaccine, 34 studies were included. Current evidence does not show</td><td></td></tr>
</table>

		conclusive proof of greater cost-effectiveness of 9-valent vaccinee compared to older HPV vaccines.[20]	
Acceptability	**Is the intervention acceptable to key stakeholders?**<br><br>○ No<br>○ Probably no<br>○ Probably yes (highlighted)<br>○ Yes<br><br>○ Varies<br>○ Don't know	There are no studies that addressed this question specifically in the IBD population.<br><br>In a systematic review of barriers and facilitators to HPV vaccination of young women in high-income countries, 41 studies were included.[21] Whether young women receive the HPV vaccine is strongly governed by the decisions of policy makers, health care professionals, and parents. **These decisions are shaped by: financial considerations, social norms and values relating to sexual activity, and; trust in vaccination programmes and healthcare providers**. In the healthcare setting, judgments by healthcare professionals about whether to recommend the vaccine may restrict a young woman's access to the vaccine irrespective of her own beliefs and preferences. Parents may decide not to allow their daughters to be vaccine, based on cultural or religious perceptions about sexual activity.<br><br>In a systematic review of barriers to HPV vaccination among US adolescents, 55 relevant articles were summarized by target populations: health care professionals, parents, undeserved and disadvantaged populations, and males.[22] Health care professionals cited financial concerns and parental attitudes and concerns as barriers to providing the HPV vaccine to patients. **Concerns about the vaccine's effect on sexual behaviour, low perceived risks of HPV infection, social influences, irregular preventative care, and vaccine cost were also identified as potential barriers among parent**s. Parents consistently cited health care professional recommendations as one of the most important factors in their decision to vaccinate their children.	
Feasibility	**Is the intervention feasible to implement?**<br><br>○ No<br>○ Probably no<br>○ Probably yes<br>○ Yes (highlighted)<br><br>○ Varies<br>○ Don't know		

References:
1. Jung YS, Han M, Park S, Kim WH, Cheon JH. Cancer Risk in the Early Stages of Inflammatory Bowel Disease in Korean Patients: A Nationwide Population-based Study. J Crohns Colitis. 2017 Aug 1;11(8):954-962.
2. Dugué PA, Rebolj M, Hallas J, Garred P, Lynge E. Risk of cervical cancer in women with autoimmune diseases, in relation with their use of immunosuppressants and screening: population-based cohort study. Int J Cancer. 2015 Mar 15;136(6):E711-9.
3. Kim SC, Glynn RJ, Giovannucci E, Hernández-Díaz S, Liu J, Feldman S, Karlson EW, Schneeweiss S, Solomon DH. Risk of high-grade cervical dysplasia and cervical cancer in women with systemic inflammatory diseases: a population-based cohort study. Ann Rheum Dis. 2015 Jul;74(7):1360-7.
4. Rungoe C, Simonsen J, Riis L, Frisch M, Langholz E, Jess T. Inflammatory bowel disease and cervical neoplasia: a population-based nationwide cohort study. Clin Gastroenterol Hepatol. 2015 Apr;13(4):693-700.
5. Jess T, Horváth-Puhó E, Fallingborg J, Rasmussen HH, Jacobsen BA. Cancer risk in inflammatory bowel disease according to patient phenotype and treatment: a Danish population-based cohort study. Am J Gastroenterol. 2013 Dec;108(12):1869-76.
6. Singh H, Demers AA, Nugent Z, Mahmud SM, Kliewer EV, Bernstein CN. Risk of cervical abnormalities in women with inflammatory bowel disease: a population-based nested case-control study. Gastroenterology. 2009 Feb;136(2):451-8.
7. Lees CW, Critchley J, Chee N, Beez T, Gailer RE, Williams AR, Shand AG, Arnott ID, Satsangi J. Lack of association between cervical dysplasia and IBD: a large case-control study. Inflamm Bowel Dis. 2009 Nov;15(11):1621-9.
8. Marehbian J, Arrighi HM, Hass S, Tian H, Sandborn WJ. Adverse events associated with common therapy regimens for moderate-to-severe Crohn's disease. Am J Gastroenterol. 2009 Oct;104(10):2524-33.
9. Kane S, Khatibi B, Reddy D. Higher incidence of abnormal Pap smears in women with inflammatory bowel disease. Am J Gastroenterol. 2008 Mar;103(3):631-6.
10. Hutfless S, Fireman B, Kane S, Herrinton LJ. Screening differences and risk of cervical cancer in inflammatory bowel disease. Aliment Pharmacol Ther. 2008 Sep 1;28(5):598-605.
11. Bhatia J, Bratcher J, Korelitz B, Vakher K, Mannor S, Shevchuk M, Panagopoulos G, Ofer A, Tamas E, Kotsali P, Vele O. Abnormalities of uterine cervix in women with inflammatory bowel disease. World J Gastroenterol. 2006 Oct 14;12(38):6167-71.
12. Bernstein CN, Blanchard JF, Kliewer E, Wajda A. Cancer risk in patients with inflammatory bowel disease: a population-based study. Cancer. 2001 Feb 15;91(4):854-62.
13. Allegretti JR, Barnes EL, Cameron A. Are patients with inflammatory bowel disease on chronic immunosuppressive therapy at increased risk of cervical high-grade dysplasia/cancer? A meta-analysis. Inflamm Bowel Dis. 2015 May;21(5):1089-97.
14. Slesser AA, Bhangu A, Bower M, Goldin R, Tekkis PP. A systematic review of anal squamous cell carcinoma in inflammatory bowel disease. Surg Oncol. 2013 Dec;22(4):230-7.
15. Jacobson DL, Bousvaros A, Ashworth L, Carey R, Shrier LA, Burchett SK, Renna H, Lu Y. Immunogenicity and tolerability to human papillomavirus-like particle vaccine in girls and young women with inflammatory bowel disease. Inflamm Bowel Dis. 2013 Jun;19(7):1441-9.
16. Joura E, Bautista O, Luxembourg A. A 9-Valent HPV Vaccine in Women. N Engl J Med. 2015 Jun 25;372(26):2568-9.
17. Luxembourg A. program summary and new 9-valent HPV vaccine trial data. Presentation before the Advisory Committee on Immunization Practices (ACIP), October 30, 2014. Atlanta, GA: US Department of Health and Human Services, CDC;2014.
18. Yang S, Miller N, Fink D, Hulse A. Original application: human papillomavirus 9-valent vaccine, recombinant. Silver Spring, MD: US Department of Health and Human Services; Food and Drug Administration; 2014.

19. Seto K, Marra F, Raymakers A, Marra CA. The cost effectiveness of human papillomavirus vaccines: a systematic review. Drugs. 2012 Mar 26;72(5):715-43.
20. Ng SS, Hutubessy R, Chaiyakunapruk N. Systematic review of cost-effectiveness studies of human papillomavirus (HPV) vaccination: 9-Valent vaccine, gender-neutral and multiple age cohort vaccination. Vaccine. 2018 May 3;36(19):2529-2544.
21. Ferrer HB, Trotter C, Hickman M, Audrey S. Barriers and facilitators to HPV vaccination of young women in high-income countries: a qualitative systematic review and evidence synthesis. BMC Public Health. 2014 Jul 9;14:700.
22. Holman DM, Benard V, Roland KB, Watson M, Liddon N, Stokley S. Barriers to human papillomavirus vaccination among US adolescents: a systematic review of the literature. JAMA Pediatr. 2014 Jan;168(1):76-82.

Conclusion – Females with IBD age 9 to 26

PICO 21: In female patients with IBD age 9 to 26, should HPV vaccine be given?
Moderate certainty of evidence CIN≥ 2
Direction – Yes (100%)
Strength – Strong (100%)

**Type of recommendation**	**Strong recommendation against the intervention**	**Conditional recommendation against the intervention**	**Conditional recommendation for either the intervention or the comparison**	**Conditional recommendation for the intervention**	**Strong recommendation for the intervention**
	○	○	○	○	○ (highlighted)
**Recommendation**	**Statement 21: In female patients with IBD age 9 to 26, we recommend HPV vaccine be given.**				
**Justification**					
**Subgroup considerations**					

**Implementation considerations**	
**Monitoring and evaluation**	• Ongoing monitoring of serious adverse events associated with HPV vaccines in IBD patients
**Research priorities**	• More studies to determine the immunogenicity and safety of HPV vaccines in female IBD patients on immunosuppressants<br>• Observational studies to determine the effectiveness of HPV vaccines in preventing HPV-related diseases in female IBD patients

Evidence to Decision Table – Males with IBD age 9 to 26

**PICO 22**	**In male patients with IBD age 9 to 26, should vaccination vs. no vaccination against human papillomavirus (HPV) be given?**
**Population**	Male patients with IBD age 9 to 26
**Intervention**	Vaccination against HPV
**Comparator**	No vaccination against HPV
**Outcome**	Mortality, VPI (HPV and HPV-related complications), SAEs, Immunogenicity

	Judgement	Research evidence	Additional considerations
**Desirable Effects**	**How substantial are the desirable anticipated effects?**<br><br>○ Trivial<br>○ Small<br>○ Moderate<br>○ Large	See Evidence Profile Tables.<br><br>**9vHPV Vaccine in the male IBD Population – catch-up from age 13 through 26 and routine age group at age 9 to 12 (Using CDC evidence profile tables as anchor)**<br><br>For HPV vaccination of males, evidence used to evaluate efficacy of 9vHPV for	

	○ Varies<br>○ Don't know	prevention of HPV 6, 11, 16, 18-related outcomes was from 1 RCT of 4vHPV among approximately 4,000 males aged 16 through 26 years, which evaluated anogenital warts; anal precancer outcomes were evaluated in a subset of approximately 600,[1] and an immunogenicity study comparing 9vHPV in males with females aged 16 through 26 years.[2] Noninferior immunogenicity of 9vHPV in males compared with females was used to infer efficacy for prevention of HPV 6, 11, 16, and 18-related outcomes. For HPV vaccination of males in the routine age group, evidence was also from an immunobridging trial, which showed non-inferior immunogenicity of 9vHPV in males aged 9 through 15 years compared to females aged 16 through 26 years.[2] These data was used to infer efficacy for prevention of HPV 6, 11, 16, 18-related outcomes. The GRADE assessment was anchored at evidence for general population for males (anal cancer), and the rating started at moderate for effectiveness. However, the rating was downgraded to **low** due to indirectness (immunogenicity data from female IBD population). For safety, the rating started as low, and was downgraded to very low due to indirectness (no data on male IBD populations and small sample size in female IBD population cannot detect rare adverse events). **Overall, there is very low certainty evidence that 9vHPV vaccine is safe and effective in reducing the risks of anal cancer given to male IBD patients at age 11 or 12 and from age 13 through 21.**	
**Undesirable Effects**	**How substantial are the undesirable anticipated effects?**<br><br>○ Large<br>○ Moderate<br>○ Small<br>○ Trivial<br><br>○ Varies<br>○ Don't know		
**Certainty of evidence**	**What is the overall certainty of the evidence of effects?**<br><br>○ Very low<br>○ Low<br>○ Moderate<br>○ High<br><br>○ No included studies		
**Values and Preferences**	**Is there important uncertainty about or variability in how much people value the main outcomes?**<br><br>○ Important uncertainty or variability<br>○ Possibly important uncertainty or variability<br>○ Probably no important uncertainty or variability<br>○ No important uncertainty or variability	The main outcome is anal cancer. But no anal cancer was reported in any of the studies. Anal intraepithelial neoplasia grade 2 or 3 is therefore used as a surrogate marker for anal cancer.<br><br>Patients likely value the outcomes of anal cancer and vaccine-related adverse effects more than anal intraepithelial neoplasia grade 2 or 3 as a surrogate marker for anal cancer.	

**Balance of effects**	**Does the balance between desirable and undesirable effects favor the intervention or the comparison?**<br><br>○ Favors the comparison<br>○ Probably favors the comparison<br>○ Does not favor either the intervention or the comparison<br>○ Probably favors the intervention<br>○ Favors the intervention<br><br>○ Varies<br>○ Don't know		
**Resources required**	**How large are the resource requirements (costs)?**<br><br>○ Large costs<br>○ Moderate costs outside school age<br>○ Negligible costs and savings school age<br>○ Moderate savings<br>○ Large savings<br><br>○ Varies<br>○ Don't know	CDC vaccine price list last reviewed/updated: July 1, 2019<br><br><table><tr><th>Brand name</th><th>CDC cost/dose</th><th>Private sector cost/dose</th></tr><tr><td>Gardasil®9</td><td>$178.14</td><td>$227.93</td></tr></table>	
**Certainty of Evidence of Required Resources**	**What is the certainty of the evidence of resource requirements (costs)?**<br><br>○ Very low<br>○ Low<br>○ Moderate<br>○ High<br><br>○ No included studies	The costs of delivering routine immunization services may vary widely across countries and different health system settings. See Immunization Costing Action Network (ICAN) Immunization Delivery Cost Catalogue. http://immunizationeconomics.org/ican-idcc.	

**Cost effectiveness**	**Does the cost-effectiveness of the intervention favor the intervention or the comparison?**<br><br>○ Favors the comparison<br>○ Probably favors the comparison<br>○ Does not favor either the intervention or the comparison<br>○ Probably favors the intervention<br>○ Favors the intervention<br><br>○ Varies<br>○ No included studies	There are no studies that addressed this question specifically in the IBD population.<br><br>In a recent systemic review of the cost-effectiveness of implementing an HPV vaccination programme with routine cervical cancer screening, a total of 29 studies were included (17 looked only at cervical disease outcomes, and 12 also included non-cervical disease outcomes).[3] Bivalent vaccine and quadrivalent vaccines were evaluated in these studies. The majority modelled HPV vaccination in adolescent girls, one looked at HPV vaccination in women over the age of 35 years, and one included both girls and boys in the model. While different model structures, input parameters and baseline assumptions were used, the consistent message in studies that focused on female-only vaccination programmes was that routine vaccination of females is cost effective compared with cervical screening alone. **It appears the addition of boys to a vaccination programme generally exceeds traditional cost effectiveness thresholds ($US 50,000 per QALY).**<br><br>In a systematic review of cost-effectiveness studies of 9-valent vaccine comparing to bi- or quadrivalent vaccine, 34 studies were included.[4] **The inclusion of adolescent boys in vaccination program was found to be cost-effective if vaccine price and coverage was low. When coverage for female was above 75%, gender-neutral vaccination was less cost-effective than when targeting only girls aged 9 – 18 years.** Multi-cohort immunization strategy was cost-effective in the age range 9 – 14 years, but the upper age limit at which vaccination was no longer cost-effective needs to be further investigated.	
**Acceptability**	**Is the intervention acceptable to key stakeholders?**<br><br>○ No<br>○ Probably no<br>○ Probably yes<br>○ Yes<br><br>○ Varies<br>○ Don't know	There are no studies that addressed this question specifically in the IBD population.<br><br>In a systematic review of factors associated with parents' attitudes to the HPV vaccination of their adolescent sons, 18 studies were included.[5] **Parents in the selected studies were generally supportive of HPV vaccination for boys. Parental decisions were predominantly shaped by the perceived benefits of the vaccine for preventing cancer and other diseases. The second most important attribute of the vaccine strongly associated with acceptability was parental perception about the importance of future partner protection**. Parents who perceived the importance of future partner protection were more accepting of HPV vaccination for their sons. Fear of side effects and uncertainty about vaccine effectiveness, as well as cost and lack of healthcare, were barriers to HPV vaccination. Other factors such as knowledge, family characteristics, parent-child dialogue and egalitarian values appeared to be important when deciding whether to vaccinate boys.<br><br>In a systematic review and meta-analysis of HPV vaccine acceptability among men, 22 studies (n = 8360) were identified.[6] **Weighted mean HPV vaccine acceptability was moderate = 50.4 (SD 21.5) with a wide range of acceptability (8.2-94.0) across studies (100-point scale)**. Among 16 studies included in meta-analyses**, perceived HPV vaccine effects, anticipatory regret, partner thinks one should get vaccine and healthcare provider recommendation had medium size effects**, and the following factors had	

		small effect sizes on HPV vaccine acceptability: perceived HPV vaccine effectiveness, need for multiple shots, fear of needles, fear of side effects, supportive / accepting social environment, perceived risk / susceptibility to HPV, perceived HPV severity, number of lifetime sexual partners, having a current sex partner, non-receipt of hepatitis B vaccine, smoking, history of sexually transmitted infection, HPV awareness, HPV knowledge, cost, logistical barriers, being employed and non-white ethnicity.	
**Feasibility**	**Is the intervention feasible to implement?**<br><br>o No<br>o Probably no<br>o Probably yes<br>o Yes<br><br>o Varies<br>o Don't know		

References:

1. Palefsky JM, Giuliano AR, Goldstone S, Moreira ED Jr, Aranda C, Jessen H, Hillman R, Ferris D, Coutlee F, Stoler MH, Marshall JB, Radley D, Vuocolo S, Haupt RM, Guris D, Garner EI. HPV vaccine against anal HPV infection and anal intraepithelial neoplasia. N Engl J Med. 2011 Oct 27;365(17):1576-85.
2. Luxembourg A. program summary and new 9-valent HPV vaccine trial data. Presentation before the Advisory Committee on Immunization Practices (ACIP), October 30, 2014. Atlanta, GA: US Department of Health and Human Services, CDC;2014.
3. Seto K, Marra F, Raymakers A, Marra CA. The cost effectiveness of human papillomavirus vaccines: a systematic review. Drugs. 2012 Mar 26;72(5):715-43.
4. Ng SS, Hutubessy R, Chaiyakunapruk N. Systematic review of cost-effectiveness studies of human papillomavirus (HPV) vaccination: 9-Valent vaccine, gender-neutral and multiple age cohort vaccination. Vaccine. 2018 May 3;36(19):2529-2544.
5. Radisic G, Chapman J, Flight I, Wilson C. Factors associated with parents' attitudes to the HPV vaccination of their adolescent sons : A systematic review. Prev Med. 2017 Feb;95:26-37
6. Newman PA, Logie CH, Doukas N, Asakura K. HPV vaccine acceptability among men: a systematic review and meta-analysis. Sex Transm Infect. 2013 Nov;89(7):568-74.

Conclusion – Males with IBD age 9 to 26

PICO 22: In male patients with IBD age 9 to 26, should HPV vaccine be given?
Very low certainty
Direction – Yes (100%)
Strength - Conditional

Type of recommendation	Strong recommendation against the intervention	Conditional recommendation against the intervention	Conditional recommendation for either the intervention or the comparison	Conditional recommendation for the intervention	Strong recommendation for the intervention
	○	○	○	○	○
**Recommendation**	**Statement 22: In male patients with IBD age 9 to 26, we suggest HPV vaccine be given.**				
**Justification**					
**Subgroup considerations**					
**Implementation considerations**					
**Monitoring and evaluation**	• Ongoing monitoring of serious adverse events associated with HPV vaccines in IBD patients				
**Research priorities**	• More studies to determine the immunogenicity and safety of HPV vaccines in male IBD patients on immunosuppressants<br>• Observational studies to determine the effectiveness of HPV vaccines in preventing HPV-related diseases in male IBD patients				

Evidence to Decision Table – Females and Males with IBD age 27 through 45

PICO	In females and males with IBD age 27 through 45, should vaccination vs. no vaccination against human papillomavirus (HPV) be given?
**Population**	Females and males with IBD age 27 through 45
**Intervention**	Vaccination against HPV
**Comparator**	No vaccination against HPV
**Outcome**	Mortality, VPI (HPV and HPV-related complications), SAEs, Immunogenicity

	Judgement	Research evidence	Additional considerations
**Desirable Effects**	**How substantial are the desirable anticipated effects?**<br><br>o Trivial<br>o Small<br>o Moderate<br>o Large<br><br>o Varies<br>o Don't know	See Evidence Profile Table.<br><br>**9vHPV Vaccine in the female and male IBD Population – catch-up from age 27 through 45 (using CDC evidence profile tables as anchor)**<br><br>Since no RCTs were conducted on use of 9vHPV in this age range, extrapolation from 4vHPV efficacy was based on immunobridging data. For men, evidence was further downgraded for each outcome for indirectness since most trials enrolled women only. The GRADE assessment was anchored at evidence for general population, the rating started at moderate for female and low for male for the **combined endpoint of persistent vaccine-type HPV infections, anogenital warts, and/or CIN ≥ 1**. We did not downgrade for indirectness for effectiveness for female IBD patients as 1 observational study suggested comparable immunogenicity with the general population. However, the rating was downgraded to low for male IBD patients due to indirectness (immunogenicity data from female IBD population). For safety, the GRADE rating started at moderate, and was downgraded to low for female IBD patients as small sample size in IBD population cannot detect rare adverse events. The evidence for safety was further downgraded to very low for male IBD population as safety data was from female IBD population. **Overall, there is low and very low certainty evidence that 9vHPV vaccine is safe and effective in female IBD population (age 27 through 45) and male IBD population (age 27 through 45), respectively.**	HPV vaccines are prophylactic and do not prevent progression of infection to disease, decrease time to clearance of HPV infection, or treat HPV-related disease. Since HPV is commonly acquired soon after first sex, vaccine effectiveness will be much lower in adults than among young adolescents.
**Undesirable Effects**	**How substantial are the undesirable anticipated effects?**<br><br>o Large<br>o Moderate<br>o Small<br>o Trivial<br><br>o Varies<br>o Don't know		

**Certainty of evidence**	**What is the overall certainty of the evidence of effects?**<br><br>○ Very low for male<br>○ Low for female<br>○ Moderate<br>○ High<br><br>○ No included studies		
**Values and Preferences**	**Is there important uncertainty about or variability in how much people value the main outcomes?**<br><br>○ Important uncertainty or variability<br>○ Possibly important uncertainty or variability<br>○ Probably no important uncertainty or variability<br>○ No important uncertainty or variability	Combined endpoint of persistent vaccine-type HPV infections, anogenital warts, and/or CIN $\geq$ 1 was used by CDC. Patients will likely have variability in how much they value each of the main outcomes. As well, more values will likely be placed on cancer than any of these individual outcomes.	
**Balance of effects**	**Does the balance between desirable and undesirable effects favor the intervention or the comparison?**<br><br>○ Favors the comparison<br>○ Probably favors the comparison<br>○ Does not favor either the intervention or the comparison<br>○ Probably favors the intervention<br>○ Favors the intervention<br><br>○ Varies<br>○ Don't know	The balance between desirable and undesirable consequences is closely balanced or uncertain.	
**Resources required**	**How large are the resource requirements (costs)?**<br><br>○ Large costs<br>○ Moderate costs<br>○ Negligible costs and savings<br>○ Moderate savings<br>○ Large savings<br><br>○ Varies<br>○ Don't know	CDC vaccine price list last reviewed/updated: July 1, 2019<br><br>**Brand name** / **CDC cost/dose** / **Private sector cost/dose**<br>Gardasil®9 / $178.14 / $227.93	

**Certainty of Evidence of Required Resources**	**What is the certainty of the evidence of resource requirements (costs)?**<br><br>○ Very low<br>○ Low<br>○ Moderate<br>○ High<br><br>○ No included studies	The costs of delivering routine immunization services may vary widely across countries and different health system settings. See Immunization Costing Action Network (ICAN) Immunization Delivery Cost Catalogue. http://immunizationeconomics.org/ican-idcc.	
**Cost effectiveness**	**Does the cost-effectiveness of the intervention favor the intervention or the comparison?**<br><br>○ Favors the comparison<br>○ Probably favors the comparison<br>○ Does not favor either the intervention or the comparison<br>○ Probably favors the intervention<br>○ Favors the intervention<br><br>○ Varies<br>○ No included studies	Five health economic models of HPV vaccination in the US were reviewed by the CDC. The cost-effectiveness ratio for the current HPV vaccination program for young adolescents and adults up to age 26 for females and age 21 for males ranged from cost-saving to about $35,000 per QALY. In the context of the existing program, vaccinating adults aged 30 years or older would produce relatively small additional health benefits. The incremental cost per QALY gained by also vaccinating adults through age 30 years exceeded $300,000 in four of five models. In most models, expanding vaccination to older ages would result in less favourable cost-effectiveness ratios. Variation in results across models was likely due to uncertainties about HPV natural history (e.g. burden of HPV-associated disease caused by new HPV infections after age 26 years, and prevalence of immunity following clearance of natural infections) and level of herd protection from the existing HPV vaccination program.	
**Acceptability**	**Is the intervention acceptable to key stakeholders?**<br><br>○ No<br>○ Probably no<br>○ Probably yes<br>○ Yes<br><br>○ Varies<br>○ Don't know	In a study of acceptability of HPV vaccines among women older than 26 years in the US (n = 872), the response rate was 60.8%.[1] Half the respondents indicated they would want the vaccine, even if they had to pay for it. In multivariable analyses, the only factor associated with wanting the vaccine was self-reported knowledge about HPV (RR 1.43, 1.12-1.83). A majority of participants also believed that older women in general would want the vaccine if it were covered by insurance. However, this perspective was significantly diminished if the vaccine had to be paid for out of pocket (97% vs. 22% for 26-45 years old).<br><br>In another study of HPV vaccine acceptability among a national sample of adult women in the US, **adult women had generally high levels of HPV vaccine acceptability, but were greatly influenced by cost of the vaccine**.[2] Women who had experienced negative sexual health outcomes due to HPV-specific infection rated the vaccine as more acceptable.<br><br>In a systematic review and meta-analysis of HPV vaccine acceptability among men, 22 studies (n = 8360) were identified.[3] **Weighted mean HPV vaccine acceptability was moderate = 50.4 (SD 21.5) with a wide range of acceptability (8.2-94.0) across studies (100 point scale)**. Among 16 studies included in meta-analyses**, perceived HPV vaccine effects, anticipatory regret, partner thinks one should get vaccine and healthcare provider recommendation had medium size effects**, and the following factors had	

		small effect sizes on HPV vaccine acceptability: perceived HPV vaccine effectiveness, need for multiple shots, fear of needles, fear of side effects, supportive / accepting social environment, perceived risk / susceptibility to HPV, perceived HPV severity, number of lifetime sexual partners, having a current sex partner, non-receipt of hepatitis B vaccine, smoking, history of sexually transmitted infection, HPV awareness, HPV knowledge, cost, logistical barriers, being employed and non-white ethnicity.	
**Feasibility**	**Is the intervention feasible to implement?**<br><br>○ No<br>○ Probably no<br>○ Probably yes<br>○ Yes<br><br>○ Varies<br>○ Don't know		

References:

1. Dempsey AF, Brewer SE, Pyrzanowski J, Sevick C, O'leary ST. Acceptability of human papillomavirus vaccines among women older than 26 years. Vaccine. 2015 Mar 24;33(13):1556-61.
2. Stupiansky NW, Rosenthal SL, Wiehe SE, Zimet GD. Human papillomavirus vaccine acceptability among a national sample of adult women in the USA. Sex Health. 2010 Sep;7(3):304-9.
3. Newman PA, Logie CH, Doukas N, Asakura K. HPV vaccine acceptability among men: a systematic review and meta-analysis. Sex Transm Infect. 2013 Nov;89(7):568-74.

Conclusion – Females and Males with IBD age 27 through 45

PICO: In female and male patients with IBD age 27 to 45, should HPV vaccine be given?

Direction – Yes 22% Uncertain 78%

Strength –

No consensus

**Type of recommendation**	**Strong recommendation against the intervention**	**Conditional recommendation against the intervention**	**Conditional recommendation for either the intervention or**	**Conditional recommendation for the intervention**	**Strong recommendation for the intervention**

	the comparison				
	○	○	○	○	○

**Recommendation**	**No recommendation: In female and male patients with IBD age 27 to 45, the consensus group could not make a recommendation for or against giving HPV vaccine.**
**Justification**	
**Subgroup considerations**	
**Implementation considerations**	
**Monitoring and evaluation**	• Ongoing monitoring of serious adverse events associated with HPV vaccines in IBD patients
**Research priorities**	• More studies to determine the immunogenicity and safety of HPV vaccines in adult IBD patients on immunosuppressants<br>• Observational studies to determine the effectiveness of HPV vaccines in preventing HPV-related diseases in adult IBD patients
