# Supplementary material for: Canadian Association of Gastroenterology Clinical Practice Guideline for Immunizations in Patients With Inflammatory Bowel Disease (IBD)—Part 2: Inactivated Vaccines
Source: J Can Assoc Gastroenterol. 2021 Jul 29;4(4):e72–91. doi: 10.1093/jcag/gwab016 (PMC8407486; doi:10.1093/jcag/gwab016)
Supplement: gwab016_suppl_Supplementary_Appendix_4 [file gwab016_suppl_Supplementary_Appendix_4.pdf]

Appendix 4: Immunization recommendations in IBD patients and the general population

	**Canadian Association of Gastroenterology (CAG) (IBD)**	**European Crohn's and Colitis Organization (ECCO)[1] (IBD)**	**American College of Gastroenterology (ACG)[2] (IBD)**	**Centers for Disease Control (CDC)[3] (General Population)**	**Canadian Immunization Guide (PHAC/NACI)[4] (General Population)**
Haemophilus influenzae type b (Hib)	"In pediatric patients with IBD, 5 years of age and younger, we recommend Hib vaccine be given." *Strong recommendation, moderate-certainty of evidence*	-	-	Routine Hib vaccination at age 2-6 months of age. Booster at age 12-15 months. Catch-up Hib vaccination until 59 months of age.	Primary series of Hib at age 2, 4, 6 months of age. Booster at age 12-23 months. Catch-up Hib vaccination until 5 years of age.
	"In unimmunized pediatric patients with IBD, older than 5 years of age, we suggest Hib vaccine be given." *Conditional recommendation, low-certainty of evidence*			In unimmunized adults and children age 60 months or older, Hib is recommended only for high-risk medical conditions for invasive Hib disease.	In unimmunized adults and children older than 5 years of age, Hib is recommended only for high-risk medical conditions for invasive Hib disease.
	"In unimmunized adult patients with IBD, we suggest Hib vaccine be given." *Conditional recommendation, very low-certainty of evidence*			Hib is not recommended routinely for unimmunized adults and children older than 5 years.	Hib is not recommended routinely for unimmunized adults and children older than 5 years.
Hepatitis B (HBV)	"In pediatric patients with IBD, we recommend hepatitis B vaccine be given." *Strong recommendation, moderate-certainty of evidence*	"HBV vaccination is recommended in all HBV anti-HBc Ab seronegative patients with IBD."	"Vaccination against Tdap, HAV, HBV, and HPV should be administered as per ACIP guidelines" *Conditional recommendation, very low level of evidence*	Universal HBV vaccination of infants and children	Routine HBV vaccination is recommended for all children.
	"In unimmunized adult patients with IBD with a risk factor for hepatitis B infection, we recommend hepatitis B vaccine be given." *Strong recommendation, moderate-certainty of evidence*				
	"In unimmunized adult patients with IBD without a risk factor for hepatitis B infection, we suggest hepatitis B vaccine be given." *Conditional recommendation, low-certainty of evidence*			HBV is recommended only for unimmunized adults at risk for hepatitis B infection.	People who are at increased risk of exposure or complications from hepatitis B infection should receive HBV.
	"In unimmunized adult patients with IBD on immunosuppressive therapy, the consensus group could not make a recommendation for or against giving double-dose hepatitis B vaccine." *Very low certainty of evidence*			HBV is not recommended routinely for unimmunized adults.	HBV is not recommended routinely for unimmunized adults
Human Papillomavirus (HPV)	"In female patients with IBD age 9 to 26, we recommend HPV vaccine be given." *Strong recommendation, moderate-certainty of evidence*	"Routine prophylactic HPV vaccination is recommended for females and males according to national guidelines."	"Vaccination against Tdap, HAV, HBV, and HPV should be administered as per ACIP guidelines" *Conditional recommendation, very low level of evidence*	Routine HPV vaccination for girls and boys at age 11 or 12.	Routine HPV vaccination for girls and women age 9 to less than 27 years. Routine HPV vaccination for boys and men age 9 to less than 27 years.
	"In male patients with IBD age 9 to 26, we suggest HPV vaccine be given." *Conditional recommendation, very low-certainty of evidence*			Catch-up HPV vaccination through age 26 years.	
	"In female and male patients with IBD age 27 to 45, the consensus group could not make a recommendation for or against giving HPV			No catch-up HPV vaccination for adults age 27-45, but adults who are not previously vaccinated may	HPV may be considered for adults older than 27 years with ongoing risk of exposure.

	vaccine."<br>*Low certainty of evidence (females)*<br>*Very low certainty of evidence (males)*			be at risk for new HPV infection and might benefit from vaccination in this age range.	HPV is recommended for immunocompromised and immunocompetent HIV-infected persons.
Influenza	"In pediatric patients with IBD, we recommend influenza vaccine be given."<br>*Strong recommendation, moderate-certainty of evidence*	"Annual vaccination with trivalent inactivated influenza vaccine is an effective strategy to prevent influenza."<br>"Routine influenza vaccination of patients on immunomodulators is recommended in accordance with national guidelines."	"All adult patients with IBD should undergo annual vaccination against influenza"<br>*Conditional recommendation, very low level of evidence*	Annual influenza vaccination for all age 6 months and older without contraindications.	Annual influenza vaccination for all age 6 months and older without contraindications
	"In adult patients with IBD 65 years of age and younger, we recommend influenza vaccine be given."<br>*Strong recommendation, moderate -certainty of evidence*				
	"In adult patients with IBD older than 65 years of age, we recommend influenza vaccine be given."<br>*Strong recommendation, moderate -certainty of evidence*				
Meningococcal	"In pediatric patients with IBD, we recommend age-appropriate meningococcal vaccine be given."<br>*Strong recommendation, moderate-certainty of evidence*	-	"Adolescents with IBD should receive meningococcal vaccination in accordance with routine vaccination recommendations."<br>*Conditional recommendation, very low level of evidence*	No routine meningococcal vaccine to infants.<br><br>All 11 to 12 year olds should get a meningococcal conjugate vaccine, with a booster dose at 16 years old. Teens and young adults (16 through 23 year olds) also may get a serogroup B meningococcal vaccine.	Routine meningococcal conjugate vaccine to infants at 12 months.<br><br>Infants at higher risk of invasive meningococcal disease should receive meningococcal vaccines starting at 2 months of age.<br><br>Routine meningococcal conjugate vaccines to adolescents and young adults (12-24 years) even if previously vaccinated as infant or toddler. In addition, serogroup B meningococcal vaccine may be considered on an individual basis.
	"In adult patients with IBD with a risk-factor for invasive meningococcal disease, we recommend meningococcal vaccines be given."<br>*Strong recommendation, moderate-certainty of evidence*			Routine meningococcal vaccines for children and adults who are at increased risk for invasive meningococcal disease.	Routine meningococcal vaccines for children and adults who are at increased risk for invasive meningococcal disease.
	"In adult patients with IBD without a risk-factor for invasive meningococcal disease, the consensus group could not make a recommendation for or against giving meningococcal vaccines."<br>*Moderate certainty of evidence*				
Pneumococcal	"In pediatric patients with IBD, we recommend age-appropriate pneumococcal vaccines be given."<br>*Strong recommendation, moderate-certainty of evidence*	"Pneumococcal vaccination should be given shortly before initiation of immunomodulators."	"Adult patients with IBD receiving immunosuppressive therapy should receive pneumococcal vaccine	Routine PCV13 for children younger than 2 years of age.	Routine PCV13 for infants 2 months to less than 12 months of age.<br><br>Older children who did not receive the complete schedule should be

	"In adult patients with IBD not on immunosuppressive therapy, with a risk-factor for pneumococcal disease, we recommend pneumococcal vaccines be given."<br>*Strong recommendation, moderate-certainty of evidence*		with both the PCV13 and PPSV13, in accordance with national guidelines."<br>*Conditional recommendation, very low level of evidence*		vaccinated (see CIG guide for catch-up schedule).
	"In adult patients with IBD on immunosuppressive therapy, we suggest pneumococcal vaccines be given."<br>*Conditional recommendation, low-certainty of evidence*			For children over the age of 2 and adults with certain high-risk medical conditions, both PCV13 and PPSV23 vaccines are recommended.	Children age 2 months to less than 18 years at high risk of invasive pneumococcal disease should receive both PCV13 and PPSV23 vaccines: PCV13 in infancy (3+1 schedule); PPSV23 at 24 months; PPSV23 in older children and adolescents.<br><br>For adults older than 18 years of age at high risk for invasive pneumococcal disease, both PCV13 and PPSV23 are recommended.
	"In adult patients with IBD not on immunosuppressive therapy and without a risk-factor for pneumococcal disease, the consensus group could not make a recommendation for or against giving pneumococcal vaccines"<br>*Moderate certainty of evidence*			For adults age 65 years or older, PCV13 (who have not previously received a dose) and PPSV 23 are recommended.	For adults age 65 years or older, PPSV23 (one dose) is recommended. PCV13 may be considered for vaccine-naïve adults age 65 years or older.
Diphtheria, tetanus, pertussis	"In pediatric patients with IBD, we recommend age-appropriate tetanus, diphtheria, and pertussis-containing vaccines be given."<br>*Strong recommendation, moderate -certainty of evidence*	-	"Vaccination against Tdap, HAV, HBV, and HPV should be administered as per ACIP guidelines"<br>*Conditional recommendation, very low level of evidence*	Routine DTap vaccination at 2, 4, and 6 months, at 15-18 months, and at 4-6 years. A single dose of Tdap at 11-12 years of age.	Routine DTap vaccination at 2, 4, and 6 months, at 12-23 months, and at 4-6 years. A booster dose of Tdap at 14-16 years of age.
	"In adult patients with IBD, we recommend Tdap/Td vaccine be given."<br>*Strong recommendation, moderate-certainty of evidence*			1 dose of Tdap in unimmunized adults. Td or Tdap booster every 10 years.	One dose of Tdap and two doses of Td in unimmunized adults. Td booster every 10 years, with at least one dose of Tdap in adulthood.
Herpes Zoster (HZV)	"In adult patients with IBD 50 years of age and older, we recommend recombinant zoster vaccine be given."<br>*Strong recommendation, moderate-certainty of evidence*	-	"Adults with IBD over the age of 50 should consider vaccination against herpes zoster, including certain subgroups of immunosuppressed patients"<br>*Strong recommendation, low level of evidence*	Two-dose recombinant HZV (RZV) for immunocompetent adults age 50 years and older. Not recommended in immunocompromised persons.<br><br>A single dose of live HZV (LZV) for immunocompetent adults age 60 years and older. Not recommended in immunocompromised persons	Two-dose recombinant HZV (RZV) for immunocompetent adults age 50 years and older. RZV may be considered for immunocompromised adults age 50 years and older on a case-by-case basis.<br><br>If RZV is not available, consider one dose of live HZV (LZV) in adults age 50 years and older who are immunocompetent.
	"In adult patients with IBD younger than 50 years of age, we suggest recombinant zoster vaccine be given."<br>*Conditional recommendation, low-certainty of evidence*				
Measles, Mumps, Rubella (MMR)	"In MMR-susceptible pediatric patients with IBD not on immunosuppressive therapy, we recommend MMR vaccine be given."<br>*Strong recommendation, moderate-certainty of evidence*	"Susceptible individuals should ideally be vaccinated prior to initiation of any immunomodulator therapy"	No specific recommendation in IBD patients, but refer to the CDC, ACIP and IDSA guidance.<br><br>"Indicated if unknown	Routine vaccination at 12-15 months, and the second dose at 4 through 6 years of age.<br>Catch-up vaccination for children and adolescents age 12 months through 12 years of age.	Routine vaccination at 12-15 months and at 18 months of age, no later than around school entry.<br>Catch-up vaccination for children and adolescents age 12 months to less than 13 years.
	"In MMR-susceptible pediatric patients with IBD on immunosuppressive therapy, we				

	suggest against giving MMR vaccine.”<br>*Conditional recommendation, very low-certainty of evidence*		vaccination history”<br><br>“Contraindicated in patient already on immunosuppressive therapy”		
	“In MMR-susceptible adult patients with IBD not on immunosuppressive therapy, we recommend MMR vaccine be given.”<br>*Strong recommendation, moderate-quality evidence*			Susceptible adults with no evidence of immunity to MMR.<br><br>Contraindicated in persons with “impaired immune function”	Susceptible adults should receive MMR vaccine depending on risk category (e.g. health care workers, military personnel, travelers, students in post-secondary educational settings) and whether born before or after 1970.<br><br>When considering immunization of an immunocompromised person with a live vaccine, approval from the individual's attending physician should be obtained before vaccination. For complex cases, referral to a physician with expertise in immunization or immunodeficiency is advised.
	“In MMR-susceptible adult patients with IBD on immunosuppressive therapy, we suggest against giving MMR vaccine.”<br>*Conditional recommendation, very low-certainty of evidence*				
Varicella (VZV)	“In varicella-susceptible pediatric patients with IBD not on immunosuppressive therapy, we recommend varicella vaccine be given”<br>*Strong recommendation, moderate-quality evidence*	“Where possible, seronegative patients should complete the two dose course of varicella vaccine at least 3 weeks prior to commencement of immunomodulator therapy.”	“Adults with IBD should be assessed for prior exposure to varicella and vaccinated if naïve before initiation of immunosuppressive therapy when possible”<br>*Conditional recommendation, very low level of evidence*	Routine childhood vaccination for children age older than 12 months, adolescents, and adults without evidence of immunity.<br><br>Contraindicated in immunocompromised patients.	Routine childhood vaccination for children age older than 12 months, adolescents, and adults age 50 years or younger without evidence of immunity.<br><br>Also recommended for adults older than 50 years known to be serologically susceptible to varicella based on laboratory testing.<br><br>When considering immunization of an immunocompromised person with a live vaccine, approval from the individual's attending physician should be obtained before vaccination. For complex cases, referral to a physician with expertise in immunization or immunodeficiency is advised. In cases in which, in the opinion of the physician, the benefits of immunization outweigh the risks, any of the univalent varicella vaccines can be used.
	“In varicella-susceptible pediatric patients with IBD on immunosuppressive therapy, we suggest against giving varicella vaccine be given.”<br>*Conditional recommendation, very low-certainty of evidence*				
	“In varicella-susceptible adult patients with IBD not on immunosuppressive therapy, we suggest varicella vaccine be given.”<br>*Conditional recommendation, very low-certainty of evidence*				
	“In varicella-susceptible adult patients with IBD on immunosuppressive therapy, we suggest against giving varicella vaccine.”<br>*Conditional recommendation, very low-certainty of evidence*				

ACIP, Advisory Committee on Immunization Practice; ACG, American College of Gastroenterology; CAG, Canadian Association of Gastroenterology; CDC, Centers for Disease Control and Prevention; DTaP or Tdap, Diphtheria, Tetanus, Pertussis vaccine; ECCO, European Crohn’s and Colitis Organization; HBV, hepatitis B vaccine; Hib, Haemophilus influenzae type b; HPV, human papillomavirus vaccine; HZV, herpes zoster vaccine; LZV, live zoster vaccine; MMR, mumps, measles, rubella; NACI, National Advisory Committee on Immunization; PCV13, Pneumococcal conjugate vaccine; PHAC, Public Health Agency of Canada; PPSV23, Pneumococcal polysaccharide vaccine; RZV, recombinant zoster vaccine; VZV, varicella vaccine

References:

1. Rahier JF, Magro F, Abreu C, et al. Second European evidence-based consensus on the prevention, diagnosis and management of opportunistic infections in inflammatory bowel disease. J Crohns Colitis 2014;8:443-68.
2. Farraye FA, Melmed GY, Lichtenstein GR, et al. ACG clinical guideline: Preventive care in inflammatory bowel disease. Am J Gastroenterol 2017;112:241-258.
3. Centers for Disease Control and Prevention. Advisory Committee on Immunization Practices (ACIP) vaccine recommendations and guidelines. Available at: https://www.cdc.gov/vaccines/hcp/acip-recs/index.html. Accessed November 5, 2019.
4. Public Health Agency of Canada (PHAC) and National Advisory Committee on Immunizations (NACI). Canadian Immunization Guide. Last update 2020-05-13. Available at: https://www.canada.ca/en/public-health/services/canadian-immunization-guide.html . Accessed December 1, 2020.
